# Pyrinap ligands for enantioselective syntheses of amines

**DOI:** 10.1038/s41467-020-20205-0

**Published:** 2021-01-04

**Authors:** Qi Liu, Haibo Xu, Yuling Li, Yuan Yao, Xue Zhang, Yinlong Guo, Shengming Ma

**Affiliations:** 1 State Key Laboratory of Organometallic Chemistry, Shanghai Institute of Organic Chemistry, Chinese Academy of Sciences, 345 Lingling Lu, 200032 Shanghai, People’s Republic of China; 2 University of Chinese Academy of Sciences, 100049 Beijing, People’s Republic of China; 3 Research Center for Molecular Recognition and Synthesis, Department of Chemistry, Fudan University, 220 Handan Road, 200433 Shanghai, People’s Republic of China

**Keywords:** Asymmetric catalysis, Catalytic mechanisms, Synthetic chemistry methodology

## Abstract

Amines are a class of compounds of essential importance in organic synthesis, pharmaceuticals and agrochemicals. Due to the importance of chirality in many practical applications of amines, enantioselective syntheses of amines are of high current interest. Here, we wish to report the development of (*R*,*R*_*a*_)-*N*-Nap-Pyrinap and (*R*,*S*_*a*_)-*N*-Nap-Pyrinap ligands working with CuBr to catalyze the enantioselective A^3^-coupling of terminal alkynes, aldehydes, and amines affording optically active propargylic amines, which are platform molecules for the effective derivatization to different chiral amines. With a catalyst loading as low as 0.1 mol% even in gram scale reactions, this protocol is applied to the late stage modification of some drug molecules with highly sensitive functionalities and the asymmetric synthesis of the tubulin polymerization inhibitor (*S*)-(-)-*N*-acetylcolchinol in four steps. Mechanistic studies reveal that, unlike reported catalysts, a monomeric copper(I) complex bearing a single chiral ligand is involved in the enantioselectivity-determining step.

## Abstract (web summary)

Chiral amines find use as chiral ligands, resolving reagents and versatile building blocks as well as in pharmaceuticals and agrochemicals. Here, the authors developed Pyrinap ligands for the copper-catalyzed enantioselective A^3^-coupling of terminal alkynes, aldehydes, and amines affording optically active propargylic amines.

## Introduction

**Fig. 1:**
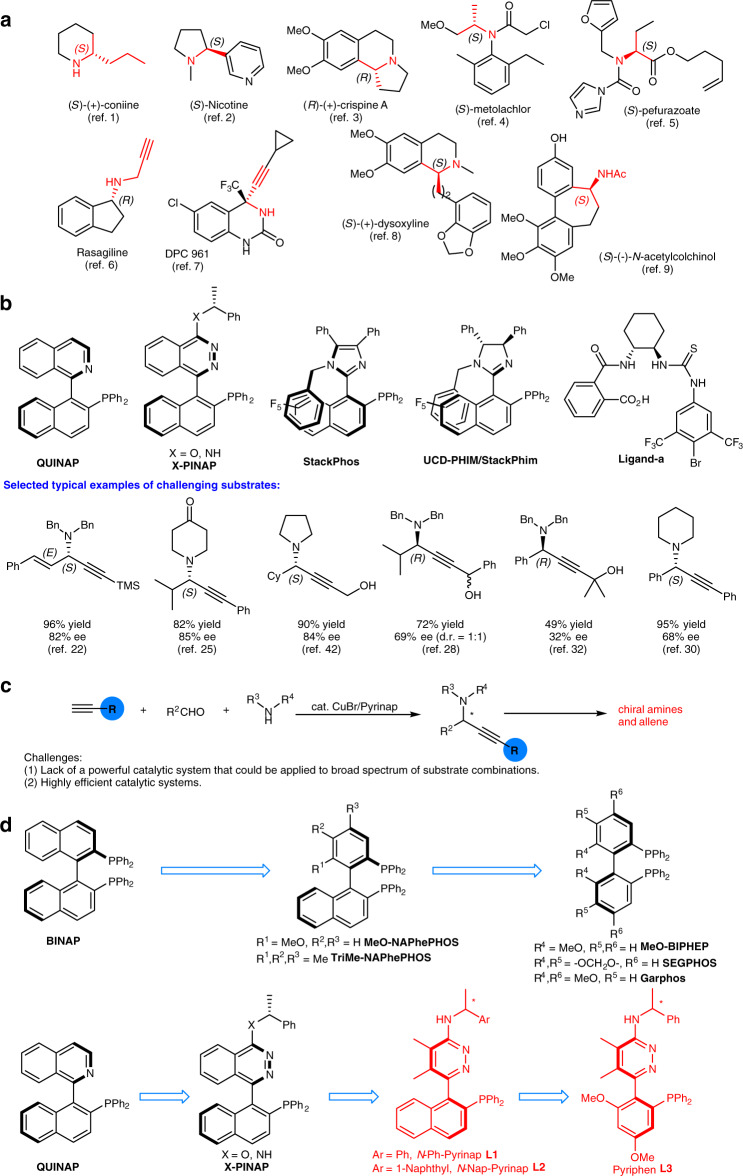
Background and concept design. **a** Selected biologically active chiral amines. **b** Known ligands for catalytic enantioselective A^3^-coupling reactions. **c** The method developed in this study. **d** Conceptual advance: evolution of binaphthyl to phenyl-naphthyl to biphenyl bisphosphines and the design of Pyrinap and Pyriphen.

Chiral amines have been not only used as resolving reagents, chiral ligands, and versatile building blocks in organic synthesis but also demonstrated wide applications in pharmaceuticals and agrochemicals (Fig. 1a)^1–9^. Thus, the development of highly efficient and enantioselective methods for syntheses of amines is of fundamental interest^10,11^. Due to the presence of a synthetically versatile carbon–carbon triple bond, propargylic amines are a very important class of compounds commonly used as precursors for other amines and diversified organic motifs. Consequently, attention has been paid to the synthesis of this type of compounds^12–14^. Enantioselective three-component coupling reaction of terminal alkynes, aldehydes, and amines provides one of the most straightforward approaches to propargylic amines due to the easy availability and diversity of the three starting materials. Chiral ligands listed in Fig. 1b have been developed or applied for this reaction by Brown^15^, Knochel^16–23^, Carreira^24,25^, Aponick^26–28^, Naeimi^29^, Seidel^30^, and Guiry^31,32^. However, challenges still remain: (1) Lack of a powerful catalytic system that could be applied to broad spectrum of very challenging combinations for three types of substrates of terminal alkynes, aldehydes, and amines. (2) More practical and efficient catalytic systems are highly desirable. Developing ligands should be the solution.

It is well known that for atropisomeric diphosphine ligands the backbone skeletons greatly affect their catalytic performance in terms of both reactivity and enantioselectivity. For example, when binaphthyl ligand BINAP (2,2′-bis(diphenylphosphino)-1,1′-binaphthyl) was replaced with the phenyl-naphthyl ligands (MeO-NAPhePHOS and TriMe-NAPhePHOS) and biphenyl ligands (MeO-BIPHEP, SEGPHOS, and Garphos), some of the challenges in enantioselective hydrogenation reactions have been properly addressed^33–39^.

In this work, inspired by such backbone effect on catalytic activity and previous studies on axially chiral P,N ligands^24,40^, we report the development of the ligands phenyl-naphthyl-type ligand *N*-Ph-Pyrinap **L1**, *N*-Nap-Pyrinap **L2**, and the diphenyl-type ligand **L3** to address the challenges with respect to the scope of the combination of alkynes, aldehydes, and amines (Fig. 1d)

## Results

### Synthesis of Pyrinap ligands

**Fig. 2:**
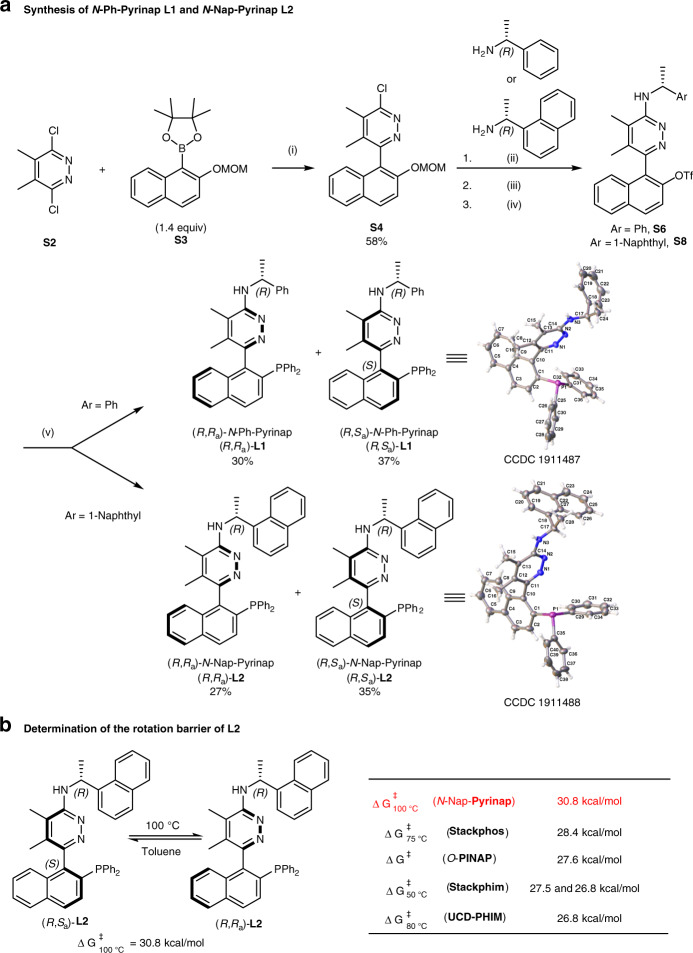
Synthesis of Pyrinap ligands and determination of rotation barrier between (*R*,*S*_*a*_)-L2 and (*R*,*R*_*a*_)-L2. Reagents and conditions: (i) Pd(OAc)_2_ (5 mol%), PPh_3_ (20 mol%), Na_2_CO_3_ (2 equiv), DME/H_2_O = 3:1, reflux, (58%); (ii) (*R*)-1-phenylethyl amine or (*R*)-1-(1-naphthyl)ethyl amine (1.3 equiv), Pd(OAc)_2_ (5 mol%), *rac*-Binap (7.5 mol%), Cs_2_CO_3_ (1.4 equiv), toluene, reflux; (iii) HCl (3 M in MeOH/H_2_O), r.t.; (iv) PhNTf_2_ (1.0 equiv), Et_3_N (1.0 equiv), DMAP (10 mol%), DCM, r.t. (for Ar = Ph, 83% yield in step (ii) and 90% yield over 2 steps (iii and iv); for Ar = 1-naphthyl, 86% yield over 2 steps (ii and iii) and 90% yield in step (iv).) (v) NiCl_2_(dppe) (10 mol%), HPPh_2_ (2 equiv), DABCO (4 equiv), DMF, 120 °C, 12 h. DME 1,2-dimethoxyethane, DMAP 4-dimethylaminopyridine, DCM dichloromethane, DABCO triethylenediamine, DMF *N*,*N*-dimethylformamide.

At first, we tried to synthesize Pyriphen **L3**. But the transformation from triflate **S1** to **L3** could not be realized by the Ni-catalyzed phosphorylation reaction. The reaction gave only a complex mixture (see Supplementary information for the details). Subsequently, we turned our attention to synthesize *N*-Ph-Pyrinap **L1** or *N*-Nap-Pyrinap **L2**. The synthesis of Pyrinap was successfully realized as shown in Fig. 2b: the Suzuki coupling reaction of 3,6-dichloro-4,5-dimethylpyridazine **S2** with boronate **S3** produced biaryl compound **S4** in 58% yield. Subsequent amination reaction with (*R*)-1-phenylethyl amine or (*R*)-1-(1-naphthyl)ethyl amine, deprotection, and triflation afforded corresponding triflates **S6** or **S8**, respectively. Finally, Ni-catalyzed coupling of **S6** or **S8** with HPPh_2_ provided a mixture of diastereomers of **L1** or **L2**, respectively, which may be separated easily via column chromatography separation on silica gel. The absolute configurations of (*R*,*S*_a_)-**L1** and (*R*,*S*_a_)-**L2** were firmly established by X-ray single-crystal analysis, respectively. (*S*,*R*_a_)-**L2** and (*S*,*S*_a_)-**L2** could also be easily prepared by the same synthetic procedure with (*S*)-1-(1-naphthyl)ethyl amine (see the Supplementary Fig. 3 for the details). In order to understand the nature of these ligands, we first determined the rotational barrier between **(*R***,***S***_***a***_**)**-**L2** and **(*R***,***R***_***a***_**)**-**L2** in toluene at 100 °C to be 30.8 kcal/mol, which is higher than those for *O*-PINAP (27.6 kcal/mol)^41^, at 75 °C for Stackphos (28.4 kcal/mol)^26^, at 50 °C for StackPhim (26.8 and 27.5 kcal/mol)^28^, and at 80 °C for UCD-PHIM (26.8 kcal/mol)^31^ (Fig. 2b). Thus, the ligand **L2** is configurationally more stable under ambient conditions.

### Optimization of reaction conditions

With these two ligands in hand, we tried the enantioselective A^3^-coupling of the most challenging propargyl alcohol **1a** with a much smaller steric hindrance, benzaldehyde **2a**, and pyrrolidine **3a**. After some screenings, it was observed that (*R*,*R*_a_)-**L2** ligand gave the highest yield (84%) and enantiomeric excess (ee) (85%) at room temperature (r.t.) (Table 1, entries 1–3). The reaction at 0 °C afforded the product in 90% ee (Table 1, entry 4). When 1.2 equivalent (equiv) each of **2a** and **3a** were applied, the yield was improved (Table 1, entry 5). The catalyst loading could be reduced to 2.5 mol% with the same level of enantioselectivity (Table 1, entry 6). Following the same conditions reported in ref. ^32^ (Table 1, entry 7), (*S*,*S*,*R*_*a*_)-UCD-PHIM provided the product in 86% yield with −85% ee (Table 1, entry 8).

**Table 1:** Optimization of the reaction conditions.


Entry	Ligand	x (mol%)	Yield of 4aaa^a^	ee of 4aaa (%)^b^
1	(*R*,*S*_a_)-**L1**	5	68	−78
2	(*R*,*S*_a_)-**L2**	5	75	−84
3	(*R*,*R*_a_)-**L2**	5	84	85
4^c^	(*R*,*R*_a_)-**L2**	5	74	90
5^c,d^	(*R*,*R*_a_)-**L2**	5	79	90
6^c,d,e^	(*R*,*R*_a_)-**L2**	2.5	73 (70^f^)	90
7^g^	(*S*,*S*,*R*_*a*_)-UCD-PHIM	1	84	−98
8^h^	(*S*,*S*,*R*_*a*_)-UCD-PHIM	1	86	−85

^a^Determined by ^1^H NMR analysis with CH_2_Br_2_ as the internal standard.

^b^Determined by chiral HPLC analysis of the isolated product.

^c^The reaction was carried out at 0 °C.

^d^1.2 equivalents of **2a** and **3a** were used.

^e^The reaction was conducted on 0.5 mmol scale.

^f^Isolated yield.

^g^Data taken from ref. ^32^.

^h^Data produced in this laboratory using (*S*,*S*,*R*_*a*_)-UCD-PHIM prepared in this laboratory.

### Substrate scope

**Fig. 3:**
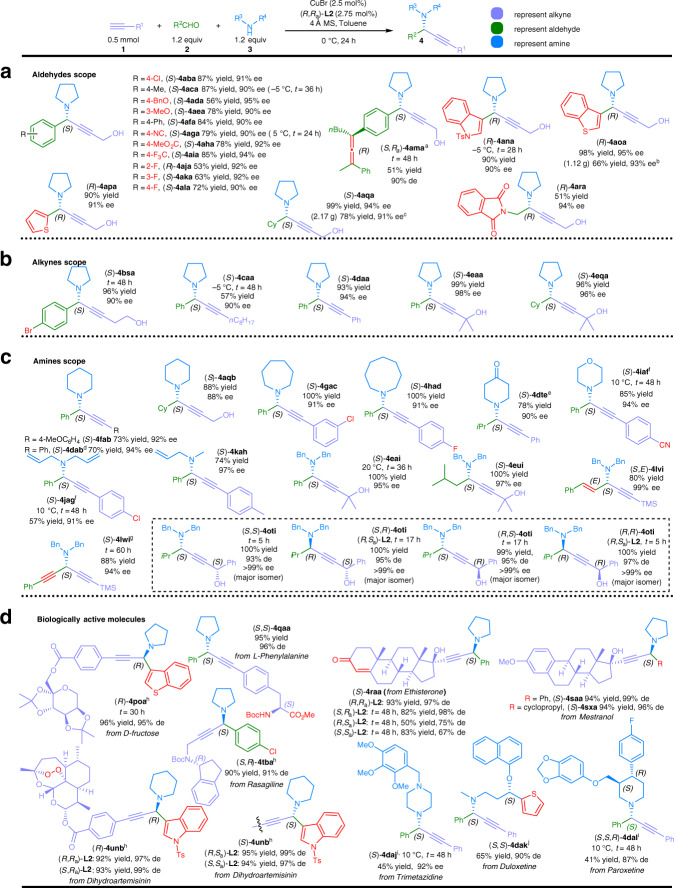
Substrate scope of the enantioselective A^3^-coupling reaction. **a** Aldehydes scope, **b** alkynes scope, **c** amines scope, and **d** biologically active molecules. Reaction conditions: ^a^the reaction was carried out using **1a** (1.2 equiv), **2m** (0.15 mmol), and **3a** (1.2 equiv). ^b^The reaction was carried out using **1a** (6.25 mmol), **2o** (1.05 equiv), **3a** (1.05 equiv), CuBr (0.1 mol%), (*R*,*R*_a_)-**L2** (0.11 mol%), and 4 Å MS (1.9 g) in toluene (16 mL) at 0 °C for 4 d. ^c^The reaction was carried out using **1a** (12.5 mmol), **2p** (1.05 equiv), **3a** (1.05 equiv), CuBr (0.1 mol%), (*R*,*R*_a_)-**L2** (0.11 mol%), and 4 Å MS (1.9 g) in toluene (31 mL) at 0 °C for 2 days. ^d^The reaction was carried out using **1d** (1.2 equiv), **2a** (0.5 mmol), and **3b** (1.2 equiv). ^e^The reaction was carried out using **1d** (0.5 mmol), **2t** (2 equiv), **3e**·HCl (1.2 equiv), NEt_3_ (2.2 equiv), CuBr (2.5 mol%), (*R*,*R*_a_)-**L2** (2.75 mol%), and 4 Å MS (150.3 mg) in DCM (1.25 mL) at 0 °C for 24 h. ^f^DMC (1.25 mL) was used as solvent. ^g^The reaction was carried out using trimethylsilylacetylene **1l** (1.5 equiv), 3-phenylpropiolaldehyde **2w** (0.5 mmol), **3i** (1.0 equiv), CuBr (2.5 mol%), (*R*,*R*_a_)-**L2** (2.75 mol%), and 4 Å MS (150.6 mg) in DCM (1.25 mL) at 0 °C for 2.5 days. ^h^The reaction was carried out on a 0.25 mmol scale. ^i^The reaction was carried out using **1** (0.5 mmol), **2** (1.2 equiv), **3**·HCl (1.2 equiv), and NEt_3_ (2.2 equiv) in DMC (1.25 mL). ^j^The reaction was carried out using **1** (0.5 mmol), **2** (1.2 equiv), **3**·HCl (1.2 equiv), and NEt_3_ (2.2 equiv) in DCM (1.25 mL). The absolute configuration before the compound no. refers to the newly generated propargylic chiral center.

With the optimized reaction conditions in hand, we firstly tested the reactivity and enantioselectivity of various aromatic aldehydes^22,25^: in general, decent yields and over 90% ee were obtained regardless of the electronic properties of the phenyl groups and the position of substituents (Fig. 3a). A wide range of synthetically useful functional groups, such as halogen (**2b**, **2j**, **2k**, **2l**), alkoxy (**2d**, **2e**), cyano (**2g**), ester (**2h**), trifluoromethyl group (**2i**), and chiral allene (**2m**) were intact under the optimal reaction conditions (products (*S*)-**4aba**, (*R*)-**4aja**, (*S*)-**4aka**, (*S*)-**4ala**, (*S*)-**4ada**, (*S*)-**4aea**, (*S*)-**4aga**, (*S*)-**4aha**, (*S*)-**4aia**, and (*S*,*R*_a_)-**4ama**). Heteroaromatic aldehydes such as Ts-protected indolecarbaldehyde (**2n**), 2-benzo[*b*]thiophenecarbaldehyde (**2o**), and 2-thiophenecarboxaldehyde (**2p**) all delivered the corresponding propargylic amines (*R*)-**4ana**, (*R*)-**4aoa**, and (*R*)-**4apa** with good yields and high ee. In addition, aliphatic aldehydes also reacted efficiently to afford the desired products (*S*)-**4aqa** and (*S*)-**4ara** in high ee.

The scope of terminal alkynes^42^ was subsequently examined (Fig. 3b): non-sterically hindered homopropargyl alcohol **1b** could produce (*S*)-**4bsa** in 96% yield with 90% ee. Even the reaction of 1°-alkyl-substituted terminal alkyne **1c** without the hydroxyl group afforded the corresponding propargylic amine (*S*)-**4caa** in 90% ee at −5 °C. As for aryl-substituted alkyne **1d**, high enantioselectivity was also obtained for the product (*S*)-**4daa**^30^. As expected, excellent yields and ee for products (*S*)-**4eaa** and (*S*)-**4eqa** were observed for tertiary propargylic alcohol **1e** with aromatic aldehyde **2a** or aliphatic aldehyde **2q**.

Encouraged by the above results, we turned to explore the scope of amines (Fig. 3c)^30^: a range of amines with different types of aldehydes and alkynes were tested (Fig. 3c). The ring size of 6- to 8-membered cyclic amines had no obvious effect on the enantiocontrol of the reaction (products (*S*)-**4fab**, (*S*)-**4dab**, (*S*)-**4aqb**, (*S*)-**4gac**, and (*S*)-**4had**). For 4-piperidone **3e**, which was used as an ammonia equivalent^25^, chiral (*S*)-**4dte** was obtained in 76% yield and 90% ee under a further modified conditions. Morpholine **3f** could also furnish the product (*S*)-**4iaf** in 85% yield with 94% ee by using dimethyl carbonate instead of toluene as the solvent. Meanwhile, the reaction could be extended to acyclic amines with good yields and excellent ee for products (*S*)-**4jag** and (*S*)-**4kah**. As we know that dibenzyl amine **3i** is an important amine due to the potential of debenzylation for further possible functionalization of the nitrogen atom. It should be noted that the known ligands for the reactions with dibenzyl amine **3i** afforded (*S*)-**4eai** and (*S,E*)-**4lvi** in 49% yield with an ee of merely 32% and 96% yield with 82% ee, respectively^22,32^. Thus, the scope of this transformation with dibenzyl amine **3i** was investigated: both aromatic and aliphatic aldehydes could achieve excellent yields and ee with tertiary propargylic alcohol **1e** (products (*S*)-**4eai** and (*S*)-**4eui**). Even an alk-2-enal or 2-alkynal, which may readily undergo conjugate addition with the amine, worked with an excellent selectivity: cinnamaldehyde *E*-**2v** smoothly yielded the corresponding product (*S*,*E*)-**4lvi** in 80% yield and 99% ee; the reaction of 3-phenylpropiolaldehyde **2w** under standard conditions was very sluggish in toluene, producing (*S*)-**4lwi** in merely 6% nuclear magnetic resonance (NMR) yield. However, 88% yield and 94% ee of (*S*)-**4lwi** could be obtained by using dichloromethane (DCM) as solvent with 1.5 equiv of trimethylsilylacetylene **1l**^27^. Importantly, all four different stereoisomers of **4oti** may be obtained in excellent yields, ee, and diastereomeric excess (d.e.) by starting from optically active propargylic alcohol (*R*)-**1o**^43^ or (*S*)-**1o**^43^ and chiral ligand (*R*,*R*_a_)-**L2** or (*R*,*S*_a_)-**L2**, respectively.

Having illustrated the broad substrate scope and efficient enantiocontrol ability of this catalytic system, late-stage modification of biologically active or drug molecules were further performed (Fig. 3d): alkynes derivatives of carbohydrate d-fructose **1p** and amino acid l-phenylalanine (*S*)-**1q**^44^ provided the corresponding products (*R*)-**4poa** and (*S*,*S*)-**4qaa** smoothly in high yields and d.e.; the terminal alkyne groups in commercial drugs, ethisterone **1r**, mestranol **1s**, and Boc-protected Rasagiline (*R*)-**1t**^45^, may be readily converted to corresponding chiral propargylic amines (*S*)-**4raa**, (*S*)-**4saa**, (*S*)-**4sxa**, and (*S*,*R*)-**4tba**, without affecting other functionalities. For ethisterone **1r**, cases of match and mismatch between the substrate chirality and the ligand chirality were observed: the reaction with (*R*,*R*_a_)-**L2** or (*S*,*R*_a_)-**L2** yielded (*S*)-**4raa** in 93% yield with 97% d.e. or 82% yield with 98% d.e., respectively. As a comparison, with (*R*,*S*_a_)-**L2** or (*S*,*S*_a_)-**L2**, the same product was produced in 50% yield with 75% d.e. or 83% yield with 67% d.e., respectively. The reaction of the terminal alkyne derivative of dihydroartemisinin, indole carboaldehyde, and piperidine afforded (*R*)-**4unb** and (*S*)-**4unb** successfully via the current protocol: even the fragile bridged peroxide group in the dihydroartemisinin, which plays an important role in antimalarial activity^46^, was tolerated. In this case, the absolute configuration of the newly formed propargylic chiral center was completely controlled by the axial chirality of the chiral ligand, regardless of the substrate chirality or the central chirality of the chiral ligand. This may be explained by the fact that the chirality in dihydroartemisinin is far away from the terminal *sp* carbon atom. Moreover, amine-containing drug molecules, trimetazine (**3j**), duloretine ((*S*)-**3k**), and paroxetine ((*S*,*R*)-**3l**), could be used directly in this reaction to deliver products (*S*)-**4daj**, (*S*,*S*)-**4dak**, and (*S*,*S*,*R*)-**4dal** in high enantioselectivity under slightly modified conditions (Fig. 3d).

To demonstrate the practical utility, the reaction of simple propargyl alcohol **1a**, cyclohexanecarbaldehyde **2q**, and pyrrolidine **3a** was performed on 12.5 mmol scale with only 0.1 mol% of CuBr and 0.11 mol% of (*R*,*R*_a_)-**L2** affording chiral propargylic amine (*S*)-**4aqa**^42^ in 78% yield (2.17g) and 91% ee after a simply acid–base extraction. Heteroaromatic aldehyde **2o** could also produce 1.12 g of (*R*)-**4aoa** in 66% yield and 93% ee on 6.25 mmol scale with 0.1 mol% of catalyst (Fig. 3a).

### Synthetic applications

**Fig. 4:**
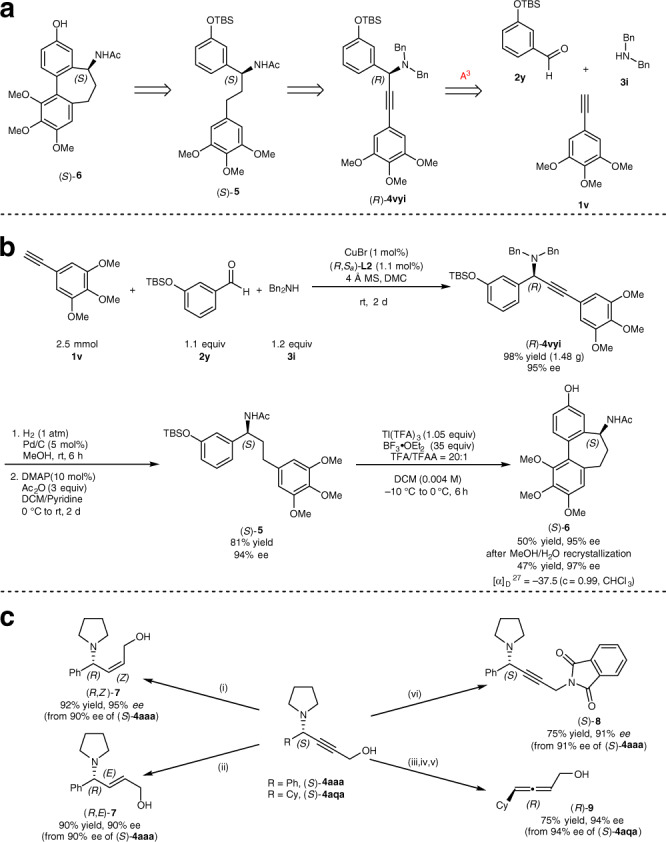
Synthetic applications. **a** Retrosynthetic analysis of (*S*)-**6**. **b** A catalytic enantioselective synthesis of (*S*)-(-)-*N*-acetylcolchinol (*S*)-**6**. **c** Transformation of propargylic amines (*S*)-**4aaa** and (*S*)-**4aqa**. Reagents and conditions: (i) Ni(OAc)_2_·4H_2_O (1 equiv), NaBH_4_ (1 equiv), ethylenediamine (3.5 equiv), EtOH, H_2_ (1 atm), r.t., 3 h; (ii) LiAlH_4_ (2 equiv), THF, 0 °C to r.t., 3 h; (iii) TBSCl (1.2 equiv), imidazole (2 equiv), DCM, r.t., 12 h; (iv) ZnI_2_ (50 mol%), toluene, 110 °C, 8 h; (v) TBAF·3H_2_O (1 equiv), THF, 0 °C to r.t., 12 h; (vi) phthalimide (1 equiv), DEAD (1.1 equiv), PPh_3_ (1 equiv), THF, 0 °C to r.t., 12 h. DCM dichloromethane, DEAD diethyl azodicarboxylate, DMAP 4-dimethylaminopyridine, DMC dimethyl carbonate, TBAF tetrabutylammonium fluoride, TBSCl *tert*-butyldimethylsilyl chloride, TFA trifluoroacetic acid, TFAA trifluoroacetic anhydride, THF tetrahydrofuran.

The colchicine degradation product (*S*)-(−)-*N*-acetylcolchinol (*S*)-**6** is the tubulin polymerization inhibitor^47–49^. Previously reported enantioselective synthesis of (*S*)-**6** suffered from using stoichiometric amounts of chiral reagents and step-economy^9,50–58^. We reasoned that our methodology could be applied to the highly efficient enantioselective synthesis of (*S*)-**6** as outlined in Fig. 4a. The key step would be the enantioselective A^3^ reaction with the above-mentioned challenging dibenzyl amine **3i** (Fig. 4b): at first, the enantioselective A^3^-coupling reaction of alkyne **1v**, aromatic aldehyde **2y**, and dibenzyl amine **3i** under standard conditions at r.t. was very sluggish, affording the desired propargylic amine (*R*)-**4vyi** in 81% yield and 96% ee with 19% of **1v** being recovered even after 7 days. Fortunately, this targeted transformation could be efficiently achieved at r.t. with 1.0 mol% of CuBr and 1.1 mol% of (*R*,*S*_a_)-**L2** in *dimethyl carbonate* producing (*R*)-**4vyi** in 98% yields and 95% ee within 48 h. Followed by Pd/C-catalyzed hydrogenation/debenzylation and acetylation, the key intermediate (*S*)-**5** was furnished in 81% yield and 94% ee. Finally, after intramolecular oxidative coupling of (*S*)-**5**, (*S*)-(-)-*N*-acetylcolchinol (*S*)-**6** was obtained in 50% yield and 95% ee (47% yield and 97% ee after recrystallization from MeOH/H_2_O; $[\alpha ]_{\mathrm{D}}^{27} = - 37.5$ (*c* = 0.99, CHCl_3_), reported value: 94% ee, $[\alpha ]_{\mathrm{D}}^{27} = - 34.0$ (*c* = 1, CHCl_3_)^56^; $[\alpha ]_{\mathrm{D}}^{20} = - 55.8$ (*c* = 0.135, CHCl_3_)^57^.

As stated in the introduction, the unique structure of chiral propargylic amine offers opportunities for further synthetic elaboration for the asymmetric syntheses of different amines (Fig. 4c): partial reduction of the C≡C triple bond in (*S*)-**4aaa** using “P-2 nickel” in the presence of ethylenediamine or LiAlH_4_ provided highly selectively (*R*,*Z*)-**7** and (*R*,*E*)-**7** in excellent yields and ee^59,60^; the Mitsunobu reaction^61^ of (*S*)-**4aaa** with phthalimide afforded 1,4-butynyl diamine (*S*)-**8** in 74% yield and 91% ee; primary α-allenols (*R*)-**9** may also be prepared with 75% yield and 94% ee by TBS protection, ZnI_2_-mediated allenation reaction, and deprotection^62,63^.

### Mechanistic studies

**Fig. 5:**
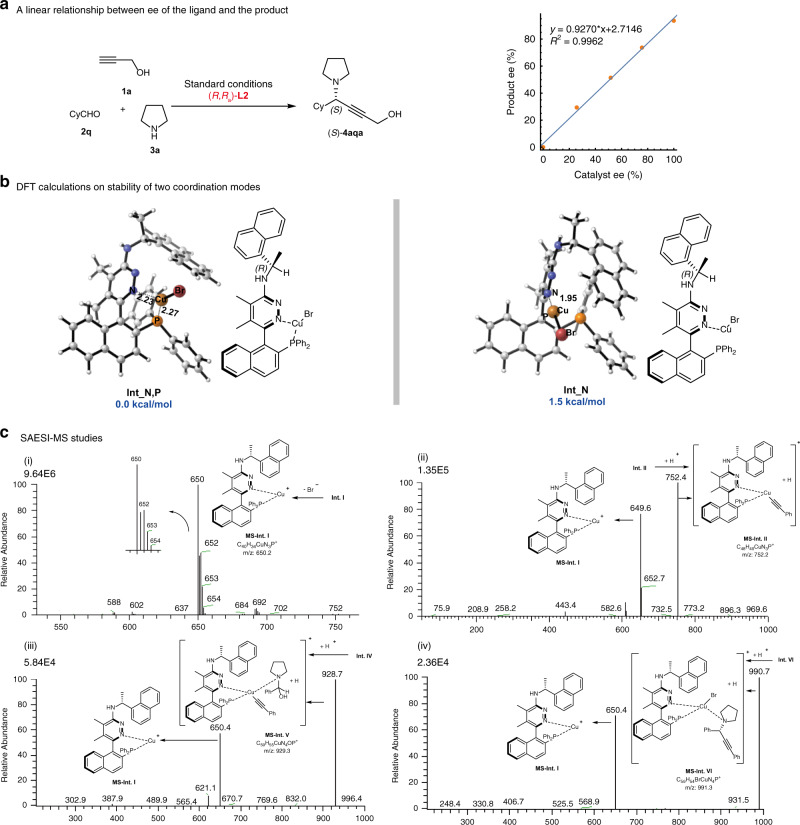
Studies on reaction mechanism. **a** The linear effect. **b** DFT calculations on the stability of two coordination modes. The calculations were performed at the M06/SDD-6-311++G(2d,p)/SMD(toluene)//M06/LANL2DZ-6-31G(d,p) level of theory at 273.15 K. The free energies Δ*G* are given with respect of **Int_N,P**. Bond lengths are given in angstroms. **c** SAESI-MS studies. (i) Expanded SAESI-MS spectrum showing the signal from *m*/*z* 540–760. (ii) SAESI-MS/MS spectrum showing the signal of **MS-Int. II** at *m*/*z* 752. (iii) SAESI-MS/MS spectrum showing the signal of **MS-Int. V** at *m*/*z* 929. (iv) SAESI-MS/MS spectrum showing the signal of **MS-Int. VI** at *m*/*z* 991.

It has been reported that Quinap demonstrated a strong positive nonlinear effect^16^, while a weak positive nonlinear effect was observed for StackPhos (see the Supplementary information file of ref. ^27^). Interestingly, a perfect linear effect was observed between the enantiopurity of (*R*,*R*_a_)-**L2** and product (*S*)-**4aqa** for the current reaction shown in Fig. 5a, which indicated that the catalytically active species most likely involves a monomeric copper(I) complex bearing a single chiral ligand. In order to acquire more information of the catalyst species in this reaction, we tried to isolate the Cu(I)-(*R*,*R*_a_)-**L2** complex^64^, but failed. We then performed ^31^P-NMR experiment to probe the coordination of CuBr with (*R*,*R*_a_)-**L2** in *d*_6_-toluene. A broad resonance at δ = −4.7 p.p.m. was observed in the ^31^P-NMR spectrum of the resulting mixture, while the resonance at δ = −12.7 p.p.m. corresponding to the free ligand (*R*,*R*_a_)-**L2** disappeared (for the details on ^31^P-NMR experiment, see Supplementary Figs. 5 and 6). The reported ^31^P-NMR chemical shifts for 1,3-bis(diphenylphosphanyl)propane (dppp) and [Cu(dppp)_2_]BF_4_ are δ = −17.2 p.p.m. and δ = −8.5 p.p.m., respectively^65^. The change in ^31^P-NMR chemical shift suggested that the P atom should coordinate to the Cu(I) atom in the solution, but may not be as strong as in the reported case. Moreover, DFT calculations were carried out to investigate the stability of two coordination modes: N,P-coordinated intermediate **Int_N,P** and N-coordinated intermediate **Int_N** (Fig. 5c, see  Supplementary information, pp 87–92 for the detailed information of computational methods). The calculated results showed that **Int_N,P** was more stable than **Int_N** by 1.5 kcal/mol.

Solvent-assisted electrospray ionization mass spectrometric experiment (SAESI-MS) was further applied to unveil the nature of catalytically active species in this reaction^66^. First, a solution of CuBr (6.25 μmol), (*R*,*R*_a_)-**L2** (6.88 μmol), and alkyne **1d** (0.25 mmol) in toluene (1 mL) was stirred at r.t. under Ar atmosphere. After 10 min, a signal with *m*/*z* of 650, which matched the *m*/*z* of mono-ligated species [Cu((*R*,*R*_a_)-**L2**)]^+^ (**MS**-**Int. I**, Fig. 5c(i), calcd for C_40_H_34_^63^CuN_3_P^+^: 650.2) was observed. Meanwhile, an alkyne-coordinated intermediate **MS-Int. II** was confirmed by a SAESI-MS/MS experiment (Fig. 5c(ii)). Then, aldehyde **2a** (0.3 mmol) and pyrroline **3a** (0.3 mmol) were added. The signal *m*/*z* of 928.7 and 990.7 were attributed to mono-ligated intermediates **MS-Int. V** (Fig. 5c(iii), calcd for C_59_H_55_^63^CuN_4_OP^+^: 929.3) and **MS-Int. VI** (Fig. 5c(iv), calcd for C_59_H_54_^79^Br^63^CuN_4_P^+^: 991.3), respectively. Their identities were further confirmed by the SAESI-MS/MS experiment (for details on MS study, see Supplementary Figs. 7–14).

**Fig. 6:**
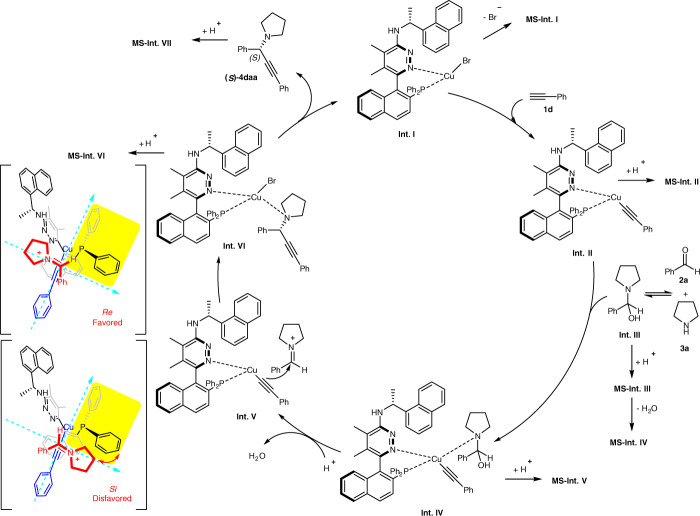
A proposed mechanism of the current A^3^-coupling reaction. A mechanism involving a monomeric copper(I) complex bearing a single chiral ligand was proposed under the instruction of the SAESI-MS studies.

Based on these experimental data, we proposed a mechanism shown in Fig. 6: the mono-ligated **Int. I** would interact with the terminal alkyne **1d** to generate **Int. II**, in which the chiral amine in the ligand may act as a proton shuttle. The reaction of amine with aldehyde generated **Int. III**, which would coordinate with the Cu atom in **Int. II** to form **Int. IV**. H^+^-mediated elimination of water formed the iminium species in **Int. V**. Enantioselective 1,2-addition would afford **Int. VI** (*Re* face attack is more favored), which underwent disassociation to release the product-propargylic amine (*S*)-**4daa** and regenerate the catalytically active **Int. I** to finish the catalytic cycle.

In conclusion, we have developed axially chiral P,N-ligands Pyrinap for the highly efficient catalytic enantioselective A^3^-coupling reaction of readily available alkynes, aldehydes, and amines to provide a variety of chiral amine-synthesis platform molecules, chiral propargylic amines, in high yields and enantioselectivity. Compared to known ligands, the salient features of this work are: (a) a general catalytic system that could be applied to a variety of challenging substrate combinations with high enantioselectivity; (b) monomeric copper(I) complex bearing a single chiral ligand has been identified as the catalytically active species; (c) the reaction has been successfully applied to the late-stage modification of some drug molecules with the sensitive functionalities survived; (d) synthetic potential has further been demonstrated by enantioselective synthesis of (*S*)-(-)-*N*-acetylcolchinol in four steps. Further studies on applications of Pyrinap and the development of diphenyl-type ligand Pyriphen **L3** are being actively pursued in this laboratory.

## Methods

### General procedure for the catalytic enantioselective A^3^-coupling reaction

To a flame-dried Schlenk tube were added CuBr (1.8 mg, 0.0125 mmol), (*R*,*R*_a_)-**L2** (8.1 mg, 0.01375 mmol), 4 Å molecular sieves (150.5 mg), and toluene (0.75 mL) sequentially under Ar atmosphere. After being stirred at room temperature for 30 min, **1a** (28.0 mg, 0.5 mmol) and **2a** (63.7 mg, 0.6 mmol)/toluene (0.5 mL) were added sequentially under Ar atmosphere. The resulting mixture was stirred at 0 °C for another 10 min, followed by the addition of pyrrolidine **3a** (42.7 mg, 0.6 mmol). After being stirred at 0 °C for 24 h, the reaction was complete as monitored by thin layer chromatography. The resulting mixture was filtrated through a short pad of basic aluminum oxide (200–300 mesh) eluted with dichloromethane/MeOH (10:1, 44 mL). After evaporation, the residue was purified by chromatography on silica gel (eluent: petroleum ether/ethyl acetate = 2:1) to afford (*S*)-**4aaa** (74.9 mg, 70%) as a liquid: 90% ee (high-performance liquid chromatography conditions: Chiralcel OD-H column, hexane/*i*-PrOH = 95/5, 1.2 mL/min, *λ* = 214 nm, *t*_R_(major) = 10.1 min, *t*_R_(minor) = 7.7 min); $[\alpha ]_{\mathrm{D}}^{31} = - 28.1$ (*c* = 1.05, CHCl_3_); ^1^H NMR (400 MHz, CDCl_3_) δ 7.51 (d, *J* = 7.2 Hz, 2H, ArH), 7.33 (t, *J* = 7.2 Hz, 2H, ArH), 7.27 (t, *J* = 6.4 Hz, 1H, ArH), 4.62 (s, 1H, CH), 4.36 (d, *J* = 1.6 Hz, 2H, OCH_2_), 2.67–2.51 (m, 4H, 2 × NCH_2_), 2.04 (s, 1 H, OH), 1.83–1.71 (m, 4H, 2 × CH_2_); ^13^C NMR (100 MHz, CDCl_3_) δ 138.9, 128.24, 128.21, 127.7, 85.0, 82.7, 58.9, 50.9, 50.5, 23.2; MS (EI) *m/z* (%) 215 (M^+^, 20.49), 138 (100); IR (neat): *ν* = 3065, 2960, 2924, 2871, 2841, 2729, 1489, 1455, 1372, 1345, 1309, 1270, 1233, 1206, 1121, 1087, 1074, 1033, 1024 cm^−1^; HRMS calcd for C_14_H_18_NO ([M + H]^+^): 216.1383, found: 216.1381.

## Supplementary information

Supplementary Information

## Data Availability

All data that support the findings of this study are available in the online version of this paper in the accompanying Supplementary information (including experimental procedures, compound characterization data, and spectra). The X-ray crystallographic coordinates for structures of (*R*,*S*_a_)-*N*-Ph-Pyrinap and (*R*,*S*_a_)-*N*-Nap-Pyrinap reported in this Article have been deposited at the Cambridge Crystallographic Data Centre (CCDC) under deposition numbers CCDC 1911487 ((*R*,*S*_a_)-*N*-Ph-Pyrinap) and 1911488 ((*R*,*S*_a_)-*N*-Nap-Pyrinap). These data can be obtained free of charge from http://www.ccdc.cam.ac.uk/data_request/cif.
