# Supplementary material for: Pyrinap ligands for enantioselective syntheses of amines
Source: Nat Commun. 2021 Jan 4;12:19. doi: 10.1038/s41467-020-20205-0 (PMC7782703; doi:10.1038/s41467-020-20205-0)
Supplement: Supplementary file 1 — Supplementary Information [file 41467_2020_20205_MOESM1_ESM.pdf]

Pyrinap Ligands for Enantioselective Syntheses of Amines

Qi Liu,[a,b] Haibo Xu,[a,b] Yuling Li,[a] Yuan Yao,[c] Xue Zhang,[a] Yinlong Guo,[a] and Shengming Ma[a,c*]

[a] State Key Laboratory of Organometallic Chemistry, Shanghai Institute of Organic Chemistry, Chinese Academy of Sciences, 345 Lingling Lu, Shanghai 200032, P. R. China

[b] University of Chinese Academy of Sciences, Beijing 100049, P. R. China

[c] Research Center for Molecular Recognition and Synthesis, Department of Chemistry, Fudan University, 220 Handan Lu, Shanghai 200433, P. R. China

[*]Correspondence and requests for materials should be addressed to S. Ma (email: masm@sioc.ac.cn)

Supplementary Information

Table of Contents

1. General Information	2
2. Synthesis of the ligands	2-18
3. Attempted synthesis of Pyriphen **L3**	18
4. Synthesis of the terminal alkynes **1p** and **1u**	18-20
5. Synthesis of chiral propargylic amines	21-70
6. Synthesis of (*S*)-(-)-*N*-acetylcolchinol (*S*)-**6**	71-74
7. Synthetic transformations	75-78
8. Determination of the rotation barrier for **L2**	78-81
9. The effect of the ee of the ligand on the ee of (*S*)-**4aqa**	81-82
10. ^{31}P-NMR experiments	82
11. SAESI-MS Experiments	83-87
12. Computational studies	87-92
NMR and HPLC spectra	93-398
Supplementary References	399-401

1. General Information

All reactions were carried out under Ar in dried Schlenk tubes or flasks. CuBr (98%) was purchased from Aladdin. $NiCl_2$(dppe) was purchased from J&K. 4 Å molecular sieves were purchased from Alfa Aesar and kept in a glove box after activation (heated at 450 °C for 10 h in a Muffle furnace, taken out after cooling to 200 °C and then kept in a glove box to allow to cool to room temperature). Aldehydes were redistilled right before use. Toluene was dried over sodium wire with benzophenone as the indicator and distilled freshly before use. Dichloromethane was dried with CaH_2 and distilled freshly before use. Dimethyl carbonate was dried with 4 Å molecular sieves and distilled freshly before use. Other reagents were used without further treatment. Petroleum ether (60 ~ 90 °C) was used for chromatography. All the temperatures were referred to the oil baths used. $CFCl_3$ was used as the internal standard for the ^{19}F NMR analysis. 85% H_3PO_4 in D_2O was used as the external standard for the ^{31}P NMR analysis.

2. Synthesis of the ligands

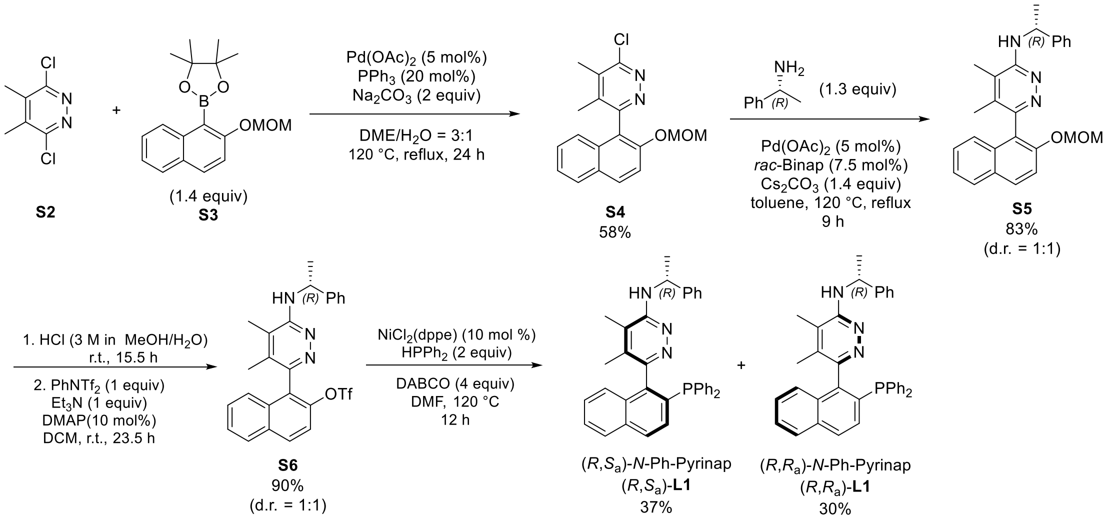

Supplementary Figure 1. Synthetic route for (*R*,*S*a)-L1 and (*R*,*R*a)-L1. DME = 1,2-dimethoxyethane, DMAP = 4-dimethylaminopyridine, DCM = dichloromethane, DABCO = triethylenediamine, DMF = *N*,*N*-dimethylformamide.

1. 3-Chloro-6-(2-(methoxymethoxy)naphthyl)-4,5-dimethylpyridazine **S4** (xhb-1-038)

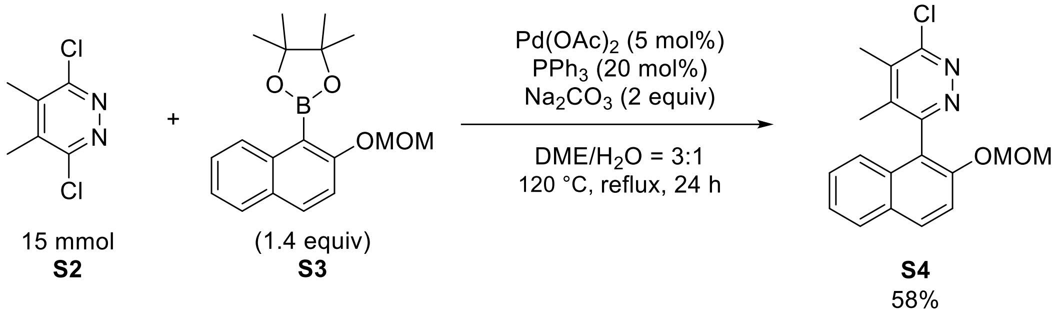

To a flask were added **S2** (2.7111 g, 15 mmol), **S3**[1] (6.6024 g, 21 mmol), $Pd(OAc)_2$ (170.5 mg, 0.75 mmol), PPh_3 (787.5 mg, 3 mmol), and Na_2CO_3 (3.2155 g, 30 mmol) sequentially. The flask was evacuated and refilled with Ar for three times and equipped with a reflux condenser. Then 44 mL of degassed mixed solvent (DME:H_2O = 3:1) were added. The resulting solution was stirred and refluxed with an oil bath preheated at 120 °C for 24 h as monitored by TLC. After cooling to room temperature, H_2O (18 mL) was added, the mixture was extracted with CH_2Cl_2 (3 × 40 mL). The combined organic layer was dried over anhydrous Na_2SO_4, filtrated, and concentrated under reduced pressure. The residue was purified by chromatography on silica gel (eluent: petroleum ether/ethyl acetate = 10:1 (550 mL) to 5:1 (1560 mL) to 4:1 (250 mL)) to afford the crude product (3.4521 g) as a yellow solid. The crude product was recrystallized from petroleum ether/ethyl acetate to afford **S4** (2.8810 g, 58%) as a white solid: m.p. 131.9-132.6 °C (ethyl acetate/petroleum ether); **^{1}H NMR** (400 MHz, $CDCl_3$) δ 7.96 (d, J = 9.2 Hz, 1 H, ArH), 7.86 (d, J = 9.2 Hz, 1 H, ArH), 7.53 (d, J = 8.8 Hz, 1 H, ArH), 7.42-7.32 (m, 2 H, ArH), 7.11 (d, J = 7.6 Hz, 1 H, ArH), 5.17 (d, J = 6.8 Hz, 1 H, one proton from OCH_2O), 5.15 (d, J = 6.8 Hz, 1 H, one proton from OCH_2O), 3.37 (s, 3 H, OCH_3), 2.50 (s, 3 H, CH_3), 2.06 (s, 3 H, CH_3); **^{13}C NMR** (100 MHz, $CDCl_3$) δ 158.7, 157.0, 152.2, 139.9, 135.9, 132.8, 130.9, 129.5, 128.0, 127.1, 124.3, 124.1, 120.6, 116.0, 94.9, 56.1, 15.84, 15.80; **MS** (ESI) *m/z* 331 ([M(^{37}Cl)+H]$^+$), 329 ([M(^{35}Cl)+H]$^+$); **IR** (neat): ν = 2947, 2904, 1619, 1592, 1541, 1527, 1506, 1468, 1451, 1435, 1410, 1380, 1338, 1310, 1263, 1245, 1229, 1215, 1195, 1172, 1151, 1104, 1088, 1078, 1030, 1010 cm^{-1}; Anal. Calcd. for $C_{18}H_{17}ClN_2O_2$: C 65.75, H 5.21, N 8.52;

Found: C 65.83, H 5.34, N 8.34.

2. 6-(2-(Methoxymethoxy)naphthyl)-4,5-dimethyl-*N*-((*R*)-1-phenylethyl)pyridazin-3-amine **S5** (xhb-1-054)

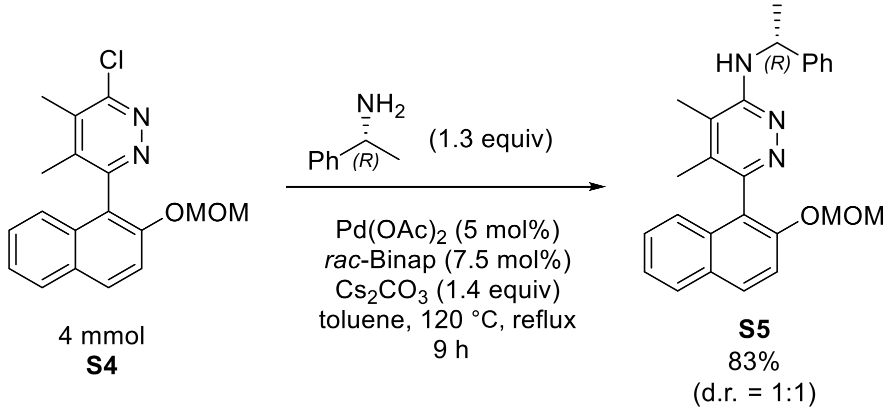

The flame-dried flask was evacuated and refilled with Ar for three times. $Pd(OAc)_2$ (46.0 mg, 0.2 mmol), *rac*-Binap (191.3 mg, 0.3 mmol), and toluene (4 mL) were added. After being stirred for 30 min at room temperature, **S4** (1.3188 g, 4 mmol), (*R*)-1-phenylethan-1-amine (0.68 mL, d = 0.940 g/mL, 0.6392 g, 5.2 mmol)/toluene (4 mL), and Cs_2CO_3 (1.8293 g, 5.6 mmol) were added. Then the flask was equipped with a reflux condenser. The resulting suspension was stirred and refluxed with an oil bath preheated at 120 °C for 9 h as monitored by TLC. After cooling to room temperature, the crude reaction mixture was filtered through a short pad of basic aluminum oxide (200-300 mesh) eluted with ethyl acetate (80 mL). The filtrate was concentrated under reduced pressure and the residue was purified by chromatography on silica gel (eluent: petroleum ether/ethyl acetate = 4:1 (750 mL) to 3:1 (1200 mL) to 2:1 (750 mL)) to afford **S5** (1.3726 g, 83%, d.r. = 1:1) (the d.r. of the product was determined via the NMR analysis) as a yellow foam: m.p. 77.7-78.7 °C (we were not able to obtain the crystal from the solvent tested, the m.p. was determined by using the solid after evaporation of the eluent); **^{1}H NMR** (400 MHz, $CDCl_3$) δ [7.86 (d, *J* = 9.2 Hz), 7.85 (d, *J* = 9.2 Hz), 1 H, ArH], 7.81-7.77 (m, 1 H, ArH), 7.54-7.42 (m, 3 H, ArH), 7.38-7.17 (m, 6 H, ArH), 5.72-5.61 (m, 1 H, NCH), [5.16 (d, *J* = 6.8 Hz, 0.47 H), 5.08 (d, *J* = 6.8 Hz, 0.50 H), 1 H, one proton from OCH_2O], [5.08 (d, *J* = 6.8 Hz, 0.50 H), 5.00 (d, *J* = 6.4 Hz, 0.47 H), 1 H, one proton from OCH_2O], [4.57 (d, *J* = 6.0 Hz, 0.49 H), 4.52 (d,

J = 6.0 Hz, 0.47 H), 1 H, NH], [3.36 (s, 1.39 H), 3.22 (s, 1.46 H), 3 H, OCH_3], 2.06 (s, 3 H, CH_3), 1.89 (s, 3 H, CH_3), [1.64 (d, J = 6.4 Hz, 1.50 H), 1.63 (d, J = 6.4 Hz, 1.50 H), 3 H, CH_3]; **MS** (ESI) *m/z* 414 ($[M+H]^+$); **IR** (neat): ν = 3297, 2966, 2898, 2824, 1622, 1592, 1579, 1556, 1509,1469, 1446, 1396, 1372, 1355, 1303, 1263, 1241, 1196, 1145, 1074, 1031, 1010 cm^{-1}; **HRMS** calcd for $C_{26}H_{28}N_3O_2$ ($[M+H]^+$): 414.2176. Found: 414.2162.

3. 1-(4,5-Dimethyl-6-(((*R*)-1-phenylethyl)amino)pyridazin-3-yl)naphth-2-yl trifluoromethanesulfonate **S6** (xhb-1-028, xhb-1-030)

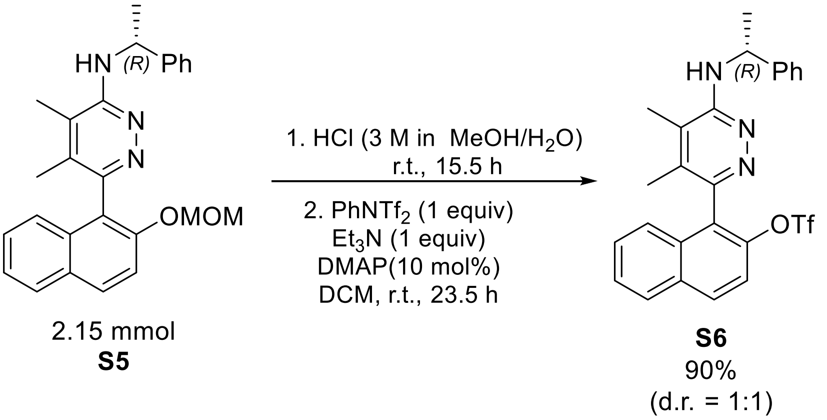

To a flask were added **S5** (887.5 mg, 2.15 mmol) and 42.9 mL of HCl (3 M in $MeOH/H_2O$ = 3:1). The resulting solution was stirred for 15.5 h at room temperature as monitored by TLC. An aqueous solution of NH_3•H_2O (12 M) was added to adjust the pH of the solution to neutral. Then H_2O (20 mL) was added, and the resulting mixture was extracted with CH_2Cl_2 (3 × 40 mL). The combined organic layer was dried over anhydrous Na_2SO_4, filtrated, and concentrated under reduced pressure to afford the residue as a yellow foam, which was used in the next step without further purification.

To a flask were added the crude product obtained above and DMAP (26.7 mg, 0.215 mmol). After being evacuated and refilled with Ar for three times, DCM (21.5 mL), Et_3N (0.30 mL, d = 0.728 g/mL, 0.2184 g, 2.16 mmol), and *N*-phenylbis(trifluoromethanesulfonimide) (784.3 mg, 2.15 mmol) were added. The resulting solution was stirred at room temperature for 23.5 h as monitored by TLC, and concentrated under reduced pressure directly. The residue was purified by

chromatography on silica gel (eluent: petroleum ether/ethyl acetate = 4:1 (750 mL) to 3:1 (1000 mL)) to afford the crude product **S6** (1.0865 g) as a yellow foam. The crude product was dissolved with 10 mL of dichloromethane and washed with a saturated aqueous solution of $NaHCO_3$ (2 × 10 mL), the organic layer was dried over anhydrous Na_2SO_4. After filtration and evaporation, the pure **S6** was obtained (0.9663 g, 90%, d.r. = 1:1) (the d.r. of the product was determined via the NMR analysis) as a yellow foam: m.p. 75.8-76.8 °C (we were not able to obtain the crystal from all the solvent tested, the m.p. value was determined by using the solid after evaporation of the solvent); **^{1}H NMR** (400 MHz, $CDCl_3$) δ 7.99 (d, *J* = 8.8 Hz, 1 H, ArH), 7.93 (d, *J* = 7.2 Hz, 1 H, ArH), 7.59-7.23 (m, 9 H, ArH), 5.70 (m, 1 H, NCH), [4.54 (s, 0.46 H), 4.52 (s, 0.45 H), 1 H, NH], 2.15 (s, 3 H, CH_3), 1.93 (s, 3 H, CH_3), [1.70 (d, *J* = 8.4 Hz, 1.48 H), 1.68 (d, *J* = 7.2 Hz, 1.56 H), 3 H, CH_3]; **^{19}F NMR** (376 MHz, $CDCl_3$) δ -74.8, -74.9; **MS** (EI) *m/z* (%) 501 (M^+, 26.99), 120 (100); **IR** (neat): ν = 3335, 2972, 1579, 1557, 1510, 1482, 1451, 1417, 1375, 1357, 1327, 1248, 1206, 1171, 1136, 1091, 1073, 1016 cm^{-1}; Anal. Calcd. for $C_{25}H_{22}F_3N_3O_3S$: C 59.87, H 4.42, N 8.38; Found: C 59.85, H 4.58, N 8.29.

4. (*R*,*S*a)-6-(2-(Diphenylphosphanyl)naphthalen-1-yl)-4,5-dimethyl-*N*-(1-phenylethyl)pyridazin-3-amine (*R*,*S*a)-**L1** and (*R*,*R*a)-6-(2-(diphenylphos-phanyl)-naphthyl)-4,5-dimethyl-*N*-(1-phenylethyl)pyridazin-3-amine (*R*,*R*a)-**L1** (xhb-1-080)

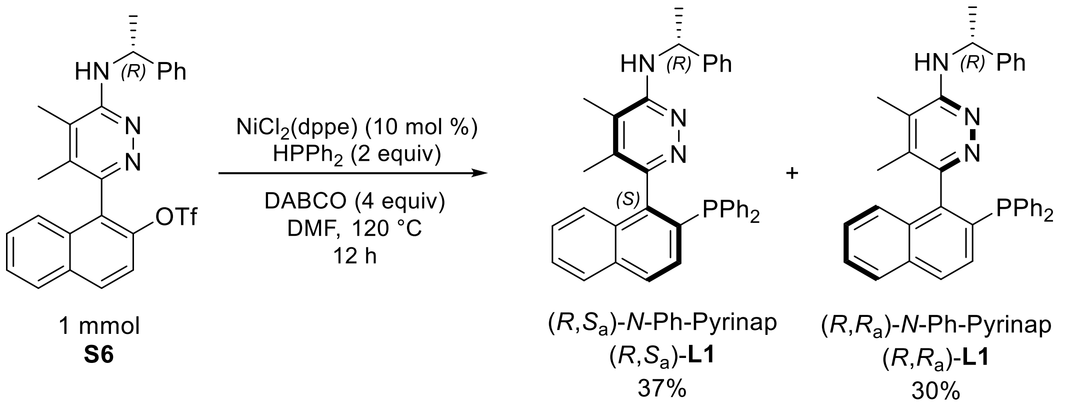

A flame-dried Schlenk tube was evacuated and refilled with Ar for three times. Then Ni(dppe)Cl_2 (53.6 mg, 0.1 mmol)/DMF (2.4 mL) and diphenylphosphine (0.36 mL, d = 1.07 g/mL, 0.3852 g, 2 mmol) were added. After being stirred in an oil bath preheated at 120 °C for 30 min, a solution of **S6** (501.6 mg, 1 mmol) and DABCO

(456.4 mg, 4 mmol) in 2.4 mL of DMF was added via a syringe. The residue in the flask was dissolved with 0.8 mL of DMF and added to Schlenk tube via a syringe. The resulting solution was stirred at 120 °C for 12 h as monitored by TLC. After being cooled to room temperature, the resulting mixture was concentrated under reduced pressure (20 mbar, bath temperature: 60 °C). The residue was filtrated through a short column (5 cm) of silica gel eluted with toluene/acetone = 10: 1 (330 mL). After evaporation, the crude residue was purified by chromatography on silica gel (eluent: toluene/ethyl acetate = 40:1 (615 mL) to 20:1 (1050 mL); then toluene/acetone = 10:1 (330 mL)) to afford (R,S_a)-**L1** (198.6 mg, 37%, top spot) as a yellow solid and (R,R_a)-**L1** (161.8 mg, 30%, bottom spot) as a yellow solid:

(R,S_a)-**L1**: m.p. 189.3-190.0 °C (toluene/hexane); $[\alpha]_D^{26}$ = -45.7 (c = 0.995, $CHCl_3$); **^{1}H NMR** (400 MHz, $CDCl_3$) δ 7.83 (d, J = 8.0 Hz, 1 H, ArH), 7.80 (d, J = 8.8 Hz, 1 H, ArH), 7.54-7.43 (m, 3 H, ArH), 7.39-7.20 (m, 16 H, ArH), 5.69 (quint, J = 6.9 Hz, 1 H, NCH), 4.41 (d, J = 7.2 Hz, 1 H, NH), 2.08 (s, 3 H, CH_3), 1.73 (s, 3 H, CH_3), 1.65 (d, J = 6.8 Hz, 3 H, CH_3); **^{13}C NMR** (100 MHz, $CDCl_3$, This compound contains many sp^2-carbons, it's difficult to recognize the C-P couplings. So we list all the signals here.) δ 155.9, 153.54, 153.48, 144.9, 143.7, 143.4, 137.8, 137.6, 137.1, 137.0, 135.25, 135.22, 134.7, 134.6, 133.9, 133.7, 133.5, 133.3, 133.1, 132.7, 132.6, 129.8, 128.4, 128.3, 128.22, 128.17, 128.1, 128.0, 127.8, 126.9, 126.7, 126.5, 126.4, 126.2, 120.3, 50.3, 22.5, 15.4, 11.7; **^{31}P NMR** (162 MHz, $CDCl_3$) δ -13.4; **MS** (ESI) *m/z* 538 ($[M+H]^+$); **IR** (neat): ν = 3352, 3055, 3025, 2971, 2925, 1583, 1558, 1480, 1452, 1433, 1395, 1374, 1356, 1322, 1273, 1208, 1139, 1090, 1069, 1048, 1025 cm^{-1}; Anal. Calcd. for $C_{36}H_{32}N_3P$: C 80.42, H 6.00, N 7.82; Found: C 80.80, H 5.91, N 7.63;

(R,R_a)-**L1**: m.p. 111.3-111.9 °C (toluene/hexane); $[\alpha]_D^{26}$ = +104.7 (c = 1.00, $CHCl_3$); **^{1}H NMR** (400 MHz, $CDCl_3$) δ 7.80 (d, J = 8.4 Hz, 1 H, ArH), 7.77 (d, J = 8.8 Hz, 1 H, ArH), 7.52-7.40 (m, 3 H, ArH), 7.37-7.15 (m, 16 H, ArH), 5.64 (quint, J = 6.5 Hz, 1 H, NCH), 4.47 (d, J = 6.8 Hz, 1 H, NH), 1.98 (s, 3 H, CH_3), 1.72 (s, 3 H, CH_3), 1.64 (d, J = 6.4 Hz, 3 H, CH_3); **^{13}C NMR** (100 MHz, $CDCl_3$, This compound contains many sp^2-carbons, it's difficult to recognize the C-P couplings. So we list all the signals here.) δ 155.9, 153.5, 153.4, 144.8, 143.5, 143.2, 137.7, 137.6, 136.7, 136.6, 135.12,

135.10, 134.9, 134.8, 133.6, 133.5, 133.44, 133.40, 133.3, 132.6, 132.5, 129.7, 128.4, 128.2, 128.11, 128.10, 128.0, 127.8, 126.9, 126.6, 126.4, 126.3, 126.05, 126.03, 120.4, 50.5, 22.2, 15.4, 11.7; **^{31}P NMR** (162 MHz, $CDCl_3$) δ -12.2; **MS** (ESI) *m/z* 538 ($[M+H]^+$); **IR** (neat): ν = 3330, 3053, 2968, 2923, 1582, 1555, 1479, 1449, 1433, 1373, 1354, 1320, 1207, 1177, 1140, 1111, 1089, 1069, 1025 cm^{-1}; Anal. Calcd. for $C_{36}H_{32}N_3P$: C 80.42, H 6.00, N 7.82; Found: C 80.27, H 6.00, N 7.79.

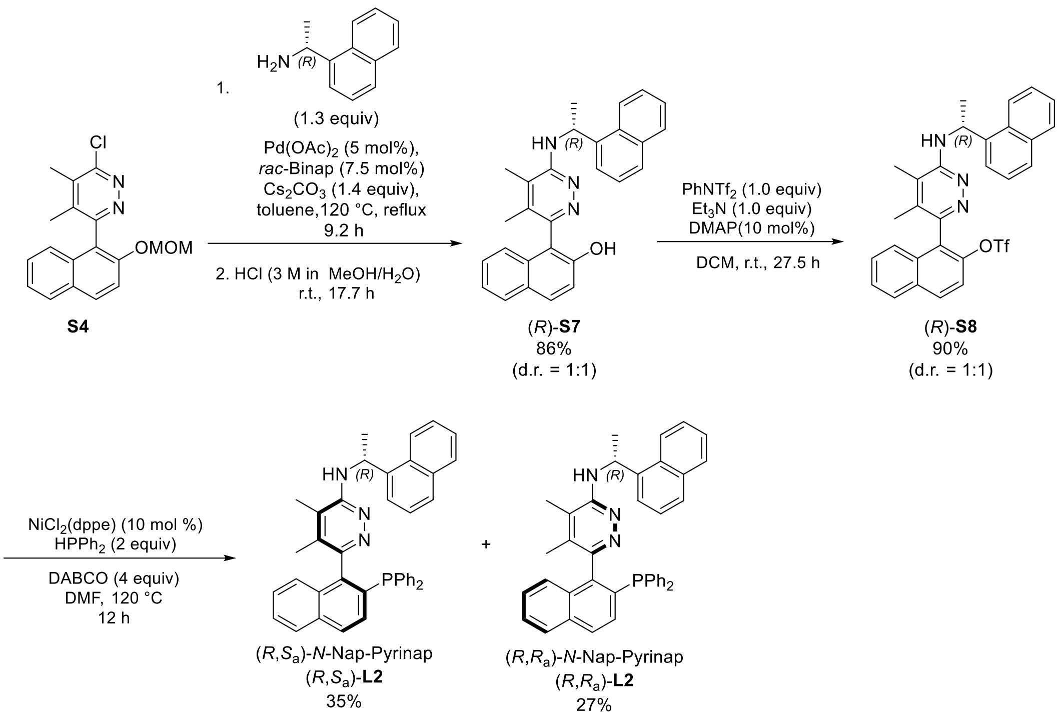

Supplementary Figure 2. Synthetic route for (*R*,*S*a)-L2 and (*R*,*R*a)-L2. DME = 1,2-dimethoxyethane, DMAP = 4-dimethylaminopyridine, DCM = dichloromethane, DABCO = triethylenediamine, DMF = *N*,*N*-dimethylformamide.

5. 1-(4,5-Dimethyl-6-(((*R*)-1-(naphthyl)ethyl)amino)pyridazin-3-yl)naphtha-2-ol (*R*)-**S7** (xhb-1-041, xhb-1-051)

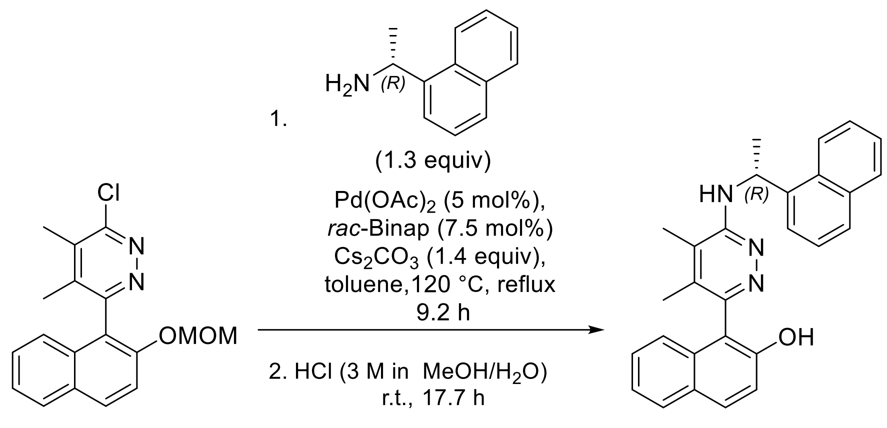

3 mmol
S4

(*R*)-**S7**
86%
(d.r. = 1:1)

The flame-dried flask was evacuated and refilled with Ar for three times. $Pd(OAc)_2$ (34.1 mg, 0.15 mmol), *rac*-Binap (143.8 mg, 0.226 mmol), and toluene (3 mL) were added. After being stirred for 30 min at room temperature, **S4** (988.3 mg, 3 mmol), (*R*)-1-(naphthyl)ethan-1-amine (0.63 mL, d = 1.067 g/mL, 0.6722 g, 3.9 mmol)/toluene (3 mL), and Cs_2CO_3 (1.3711 g, 4.2 mmol) were added. Then the flask was equipped with a reflux condenser. The resulting suspension was stirred and refluxed with an oil bath preheated at 120 °C for 9.2 h as monitored by TLC. After being cooled to room temperature, the crude reaction mixture was filtered through a short pad of basic aluminum oxide (200-300 mesh) eluted with ethyl acetate (60 mL). The filtrate was concentrated under reduced pressure and the residue was purified by chromatography on silica gel (eluent: petroleum ether/ethyl acetate = 5:1 (1200 mL) to 4:1 (750 mL) to 2:1 (300 mL)) to afford crude product as a yellow foam, which was used in the next step without further characterization.

To a flask were added the crude product obtained above and 48.7 mL of HCl (3 M in $MeOH/H_2O$ = 3:1). The resulting mixture was stirred for 17.7 h at room temperature as monitored by TLC. An aqueous solution of $NH_3•H_2O$ (12 M) was added to adjust the pH value of the resulting solution to neutral. Then H_2O (30 mL) was added, and the resulting solution was extracted with CH_2Cl_2 (3 × 50 mL). The combined organic layer was dried over anhydrous Na_2SO_4, filtrated, and concentrated under reduced pressure. The residue was purified by chromatography on silica gel (eluent: petroleum ether/ethyl acetate = 4:1 (1750 mL) to 3:1 (400 mL), then dichloromethane/methanol = 20:1 (630

mL)) to afford (*R*)-**S7** (1.0834 g, 86%, d.r. = 1:1) (the d.r. of the product was determined via the NMR analysis) as a yellow foam: m.p. 207.9-208.5 °C (ethyl acetate/petroleum ether); **^{1}H NMR** (400 MHz, $CDCl_3$) δ 8.28-8.16 (m, 1 H, ArH), 7.91-7.39 (m, 8 H, protons from ArH and OH), 7.32-7.01 (m, 5 H, protons from ArH and OH), 6.44-6.30 (m, 1 H, NCH), [4.61 (d, *J* = 6.4 Hz, 0.48 H), 4.57 (d, *J* = 6.8 Hz, 0.47 H), 1 H, NH], [1.94 (s, 1.51 H), 1.92 (s, 1.38 H), 3 H, CH_3], 1.84-1.73 (6 H, 2 × CH_3); **MS** (ESI) *m/z* 420 ($[M+H]^+$); **IR** (neat): ν = 3356, 3046, 2963, 2927, 2926, 1621, 1583, 1556, 1510, 1484, 1441, 1366, 1344, 1270, 1239, 1210, 1180, 1142, 1081, 1012 cm^{-1}; **HRMS** calcd for $C_{28}H_{26}N_3O$ ($[M+H]^+$): 420.2070. Found: 420.2071.

6. 1-(4,5-Dimethyl-6-(((*R*)-1-(naphthyl)ethyl)amino)pyridazin-3-yl)naphth-2-yl trifluoromethanesulfonate (*R*)-**S8** (xhb-1-059)

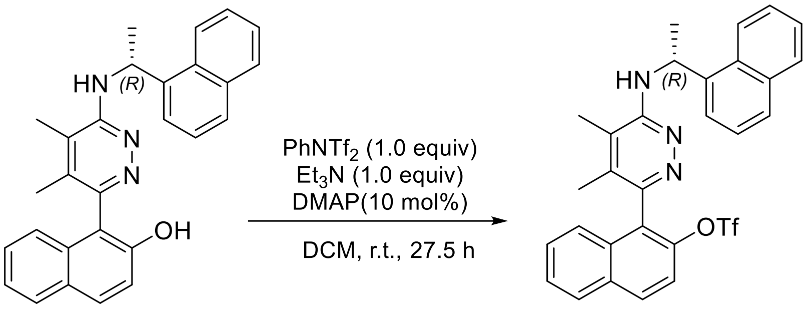

2.37 mmol
(*R*)-**S7**

(*R*)-**S8**
90%
(d.r. = 1:1)

To a flask were added (*R*)-**S7** (993.9 mg, 2.37 mmol) and DMAP (29.8 mg, 0.24 mmol). After being evacuated and refilled with Ar for three times, DCM (23.6 mL), Et_3N (0.33 mL, d = 0.728 g/mL, 0.2402 g, 2.37 mmol) and *N*-phenylbis(trifluoromethanesulfonimide) (861.7 mg, 2.36 mmol) were added. The resulting solution was stirred at room temperature for 27.5 h as monitored by TLC and concentrated under reduced pressure directly. The residue was purified by chromatography on silica gel (eluent: petroleum ether/ethyl acetate = 10:1 (330 mL) to 5:1 (1920 mL)) to afford the crude product (*R*)-**S8** (1.4254 g) as a yellow foam. The crude product was dissolved with 10 mL of dichloromethane and washed with a saturated aqueous solution of $NaHCO_3$ (2 × 10 mL). The organic layer was dried over

anhydrous Na_2SO_4. After filtration and evaporation, the pure (*R*)-**S8** was obtained (1.1909 g, 90%, d.r. = 1:1) (the d.r. of the product was determined via the NMR analysis) as a yellow foam: m.p. 103.5-104.5 °C (we were not able to obtain the crystal from all the solvent tested, the m.p. was determined by using the solid after evaporation of the solvent); **^{1}H NMR** (400 MHz, $CDCl_3$) δ [8.32 (d, *J* = 8.0 Hz, 0.49 H), 8.18-8.13 (m, 0.51 H), 1 H, ArH], 8.00 (d, *J* = 9.2 Hz, 1 H, ArH), [7.94 (d, *J* = 8.4 Hz, 0.5 H), 7.93 (d, *J* = 8.0 Hz, 0.5 H), 1 H, ArH], 7.90-7.84 (m, 1 H, ArH), 7.82 (d, *J* = 8.0 Hz, 1 H, ArH), [7.69 (d, *J* = 7.2 Hz, 0.52 H), 7.65 (d, *J* = 7.2 Hz, 0.52 H), 1 H, ArH], 7.59-7.43 (m, 7 H, ArH), 6.59-6.47 (m, 1 H, NCH), [4.57 (d, *J* = 8.0 Hz, 0.50 H), 4.55 (d, *J* = 8.4 Hz, 0.49 H), 1 H, NH], [2.07 (s, 1.48 H), 2.02 (s, 1.48 H), 3 H, CH_3], [1.92 (s, 1.50 H), 1.92 (s, 1.50 H), 3 H, CH_3], [1.88 (d, *J* = 6.4 Hz, 1.51 H), 1.84 (d, *J* = 6.4 Hz, 1.48 H), 3 H, CH_3]; **^{19}F NMR** (376 MHz, $CDCl_3$) δ -74.8, -74.9; **MS** (ESI) *m/z* 552 ($[M+H]^+$); **IR** (neat): ν = 3353, 3049, 2974, 1579, 1556, 1510, 1463, 1448, 1417, 1373, 1327, 1247, 1205, 1171, 1135, 1073, 1015 cm^{-1}; Anal. Calcd. for $C_{29}H_{24}F_3N_3O_3S$: C 63.15, H 4.39, N 7.62 cm^{-1}; Found: C 62.93, H 4.39, N 7.46.

7. (*R*,*S*a)-6-(2-(Diphenylphosphanyl)naphthalen-1-yl)-4,5-dimethyl-*N*-(1-(naphth-1-yl)ethyl)pyridazin-3-amine (*R*,*S*a)-**L2** and (*R*,*S*a)-6-(2-(diphenyl-phosphanyl)naphthalene-1-yl)-4,5-dimethyl-*N*-(1-(naphthalen-1-yl)ethyl)pyridazin-3-amine (*R*,*R*a)-**L2** (xhb-1-088)

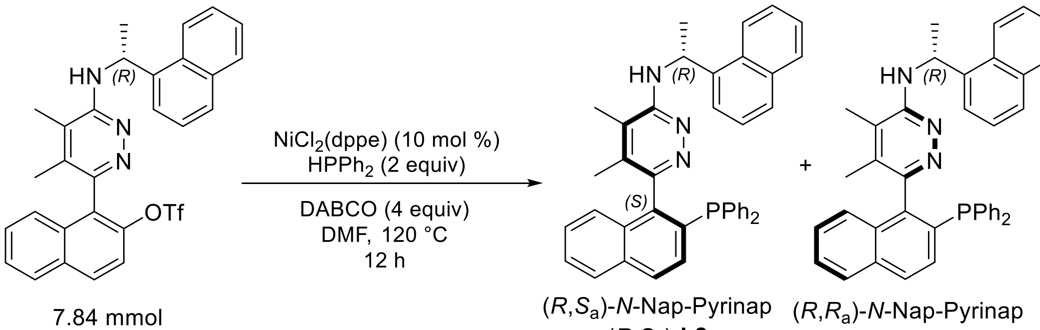

7.84 mmol (*R*)-**S8**
(*R*,*S*a)-*N*-Nap-Pyrinap (*R*,*S*a)-**L2** 35%
(*R*,*R*a)-*N*-Nap-Pyrinap (*R*,*R*a)-**L2** 27%

The flame-dried Schlenk tube was evacuated and back-filled with Ar for three times. Then Ni(dppe)Cl_2 (421.3 mg, 0.79 mmol) and diphenylphosphine (2.81 mL, d = 1.07 g/mL, 3.0067 g, 15.66 mmol)/DMF (18.8 mL) were added. After being stirred in

an oil bath preheated at 120 °C for 30 min, a solution of (*R*)-**S8** (4.3186 g, 7.84 mmol) and DABCO (3.5635 g, 31.45 mmol) in 18.8 mL of DMF was added via syringe. The residue in the flask was dissolved with 6.3 mL of DMF and added to Schlenk tube via syringe. The resulting solution was stirred at 120 °C for 12 h as monitored by TLC. After being cooled to room temperature, the resulting mixture was concentrated under reduced pressure (20 mbar, bath temperature: 60 °C). The residue was filtrated through a short column (5 cm) of silica gel eluted with toluene/acetone = 10: 1 (330 mL). After evaporation, the crude residue was purified by chromatography on silica gel (eluent: toluene/ethyl acetate = 40:1 (2050 mL); then toluene/acetone = 10:1 (330 mL)) to afford (R,S_a)-**L2** (1.6234 g, 35%, less polar) as a yellow solid and (R,R_a)-**L2** (1.2287 g, 27%, more polar) as a yellow solid:

(R,S_a)-**L2**: m.p. 211.4-212.0 °C (toluene/hexane); $[\alpha]_D^{26}$ = -65.0 (c = 1.00, $CHCl_3$); **^{1}H NMR** (400 MHz, $CDCl_3$) δ 8.32 (d, *J* = 7.2 Hz, 1 H, ArH), 7.90-7.77 (m, 4 H, ArH), 7.68 (d, *J* = 6.8 Hz, 1 H, ArH), 7.54-7.45 (m, 4 H, ArH), 7.40-7.19 (m, 13 H, ArH), 6.52 (quint, *J* = 7.2 Hz, 1 H, NCH), 4.44 (d, *J* = 7.2 Hz, 1 H, NH), 2.01 (s, 3 H, CH_3), 1.82 (d, *J* = 6.4 Hz, 3 H, CH_3), 1.74 (s, 3 H, CH_3); **^{13}C NMR** (100 MHz, $CDCl_3$, This compound contains many sp^2-carbons, it's difficult to recognize the C-P couplings. So we list all the signals here.) δ 155.7, 153.52, 153.46, 143.7, 143.3, 140.3, 137.8, 137.6, 137.1, 136.9, 135.29, 135.26, 134.8, 134.7, 133.91, 133.86, 133.7, 133.5, 133.4, 133.2, 132.7, 132.6, 131.4, 129.8, 128.5, 128.4, 128.3, 128.22, 128.18, 128.12, 128.05, 127.84, 127.78, 126.6, 126.4, 126.2, 126.1, 125.6, 125.2, 124.0, 122.4, 120.3, 46.4, 21.1, 15.4, 11.7; **^{31}P NMR** (162 MHz, $CDCl_3$) δ -13.1; **MS** (ESI) *m/z* 588 ($[M+H]^+$); **IR** (neat): ν = 3365, 3051, 3000, 2971, 2923, 1584, 1562, 1480, 1457, 1433, 1395, 1373, 1363, 1313, 1241, 1179, 1143, 1088, 1069, 1026 cm^{-1}; Anal. Calcd. for $C_{40}H_{34}N_3P$: C 81.75, H 5.83, N 7.15; Found: C 81.56, H 5.73, N 7.04;

(R,R_a)-**L2**: m.p. 128.2-128.8 °C (toluene/hexane); $[\alpha]_D^{27}$ = +129.5 (c = 1.00, $CHCl_3$); **^{1}H NMR** (400 MHz, $CDCl_3$) δ 8.31-8.23 (m, 1 H, ArH), 7.91-7.79 (m, 4 H, ArH), 7.68 (d, *J* = 7.2 Hz, 1 H, ArH), 7.54-7.45 (m, 4 H, ArH), 7.41-7.21 (m, 13 H, ArH), 6.47 (quint, *J* = 6.4 Hz, 1 H, NCH), 4.45 (d, *J* = 7.2 Hz, 1 H, NH), 1.98 (s, 3 H, CH_3), 1.86 (d, *J* = 6.8 Hz, 3 H, CH_3), 1.74 (s, 3 H, CH_3); **^{13}C NMR** (100 MHz, $CDCl_3$,

This compound contains many sp^2-carbons, it's difficult to recognize the C-P couplings. So we list all the signals here.) δ 155.7, 153.42, 153.36, 143.4, 143.1, 140.1, 137.8, 137.6, 136.7, 136.6, 135.24, 135.22, 135.19, 135.1, 133.8, 133.73, 133.5, 133.4, 133.3, 132.6, 132.5, 131.4, 129.6, 128.5, 128.2, 128.13, 128.06, 127.9, 127.8, 126.6, 126.4, 126.2, 126.0, 125.6, 125.2, 124.0, 122.4, 120.4, 46.4, 20.9, 15.4, 11.6; **^{31}P NMR** (162 MHz, $CDCl_3$) δ -12.6; **MS** (ESI) *m/z* 588 ([M+H]$^+$); **IR** (neat): ν = 3329, 3049, 2969, 2923, 1582, 1555, 1505, 1479, 1456, 1435, 1371, 1319, 1236, 1176, 1131, 1118, 1093, 1069, 1025 cm^{-1}; Anal. Calcd. for $C_{40}H_{34}N_3P$: C 81.75, H 5.83, N 7.15; Found: C 81.87, H 5.90, N 7.08.

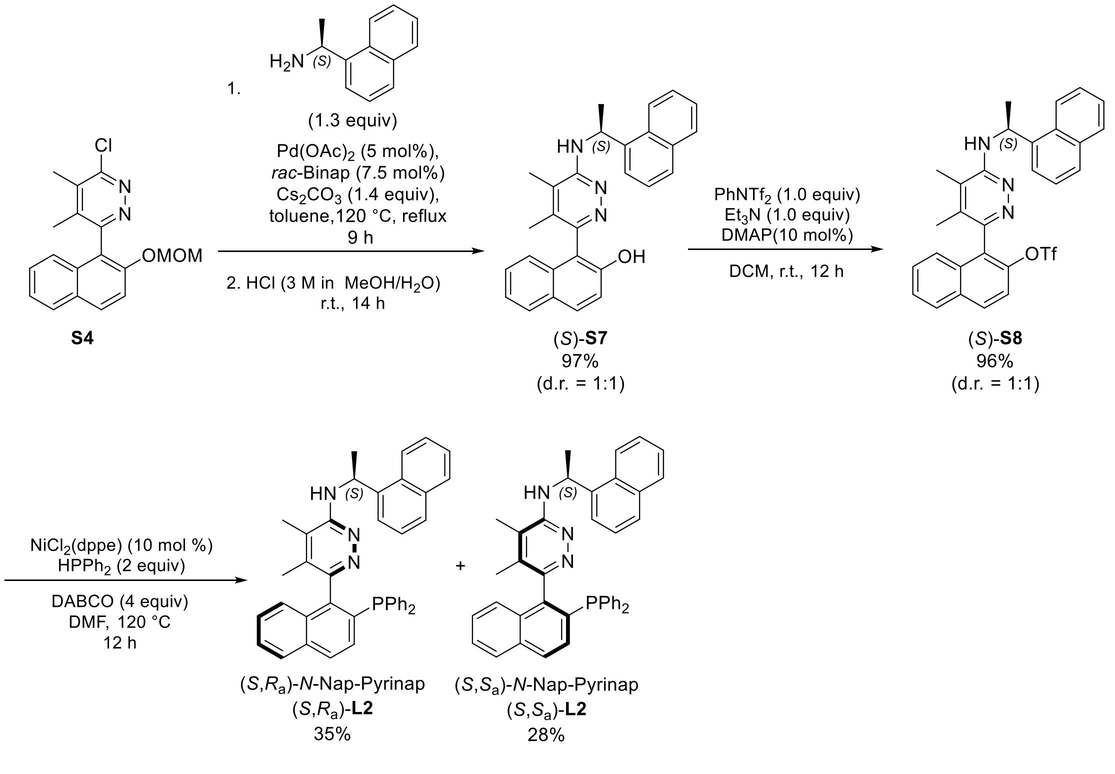

Supplementary Figure 3. **Synthetic route for (*S*,*R*a)-L2 and (*S*,*S*a)-L2.** DME = 1,2-dimethoxyethane, DMAP = 4-dimethylaminopyridine, DCM = dichloromethane, DABCO = triethylenediamine, DMF = *N*,*N*-dimethylformamide.

8. 1-(4,5-Dimethyl-6-(((*S*)-1-(naphthyl)ethyl)amino)pyridazin-3-yl)naphtha-2-ol (*S*)-**S7** (xhb-4-020, xhb-4-022)

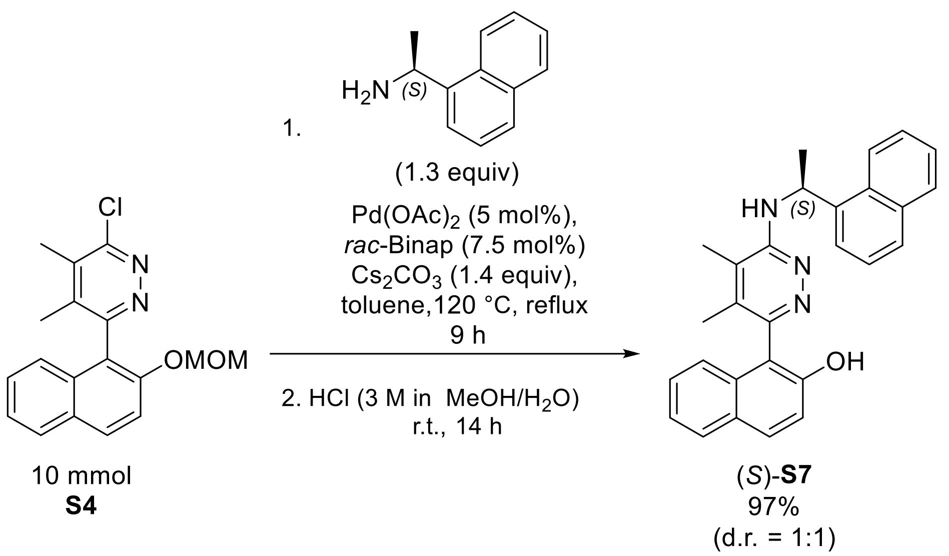

The flame-dried flask equipped with a reflux condenser was evacuated and refilled with Ar for three times. $Pd(OAc)_2$ (113.6 mg, 0.5 mmol), *rac*-BINAP (477.1 mg, 0.75 mmol) and toluene (10 mL) were added. After being stirred for 30 min at room temperature, **S4** (3.2881 g, 10 mmol), (*S*)-1-(naphthalen-1-yl)ethan-1-amine (2.2262 g, 13 mmol), Cs_2CO_3 (4.6078 g, 14 mmol) and toluene (10 mL) were added. The resulting suspension was stirred and refluxed with an oil bath preheated at 120 °C for 9 h as monitored by TLC. After cooling to room temperature, the crude reaction mixture was filtered through a short pad of basic aluminum oxide (200-300 mesh) eluted with ethyl acetate (100 mL). The filtrate was concentrated under reduced pressure and the residue was purified by chromatography on silica gel (eluent: petroleum ether/ethyl acetate = 5:1 (1200 mL) to 3:1 (800 mL) to 2:1 (300 mL)) to afford crude product as a yellow foam, which was used in the next step without further characterization.

To a flask were added the crude product obtained above and 180.4 mL of HCl (3 M in $MeOH/H_2O$ = 3:1). The resulting mixture was stirred for 14 h at room temperature as monitored by TLC. An aqueous solution of NH_3•H_2O (12 M) was added to adjust the pH of the resulting solution to neutral. Then H_2O (50 mL) was added, and the resulting solution was extracted with CH_2Cl_2 (3 × 150 mL). The combined organic layer was dried over anhydrous Na_2SO_4, filtrated, and concentrated under reduced pressure. The residue was purified by chromatography on silica gel (eluent: petroleum ether/ethyl

acetate = 4:1 (1000 mL) to 3:1 (800 mL), then dichloromethane/methanol = 20:1 (1470 mL) to 10:1 (1100 mL)) to afford (*S*)-**S7** (4.0597 g, 97%, d.r. = 1:1) (the d.r. of the product was determined via the NMR analysis) as a yellow foam: m.p. 205.4-206.5 °C (ethyl acetate/petroleum ether); **^{1}H NMR** (400 MHz, $CDCl_3$) δ 8.28-8.21 (m, 1 H, ArH), 7.94-7.09 (m, 13 H, ArH and OH), 6.49-6.37 (m, 1 H, NCH), [4.61 (d, J = 6.8 Hz, 0.5 H), 4.57 (d, J = 6.8 Hz, 0.51 H), 1 H, NH], 2.07-1.98 (m, 3 H, CH_3), 1.91-1.80 (m, 6 H, 2 × CH_3); **MS** (ESI) *m/z* 420 ($[M+H]^+$); **IR** (neat): ν = 3048, 2974, 2657, 2356, 1622, 1582, 1553, 1510, 1476, 1438, 1397, 1368, 1345, 1273, 1240, 1209, 1179, 1130, 1076 cm^{-1}; **HRMS** calcd for $C_{28}H_{26}N_3O$ ($[M+H]^+$): 420.2070. Found: 420.2060.

9. 1-(4,5-Dimethyl-6-(((*S*)-1-(naphthyl)ethyl)amino)pyridazin-3-yl)naphth-2-yl trifluoromethanesulfonate (*S*)-**S8** (xhb-4-025)

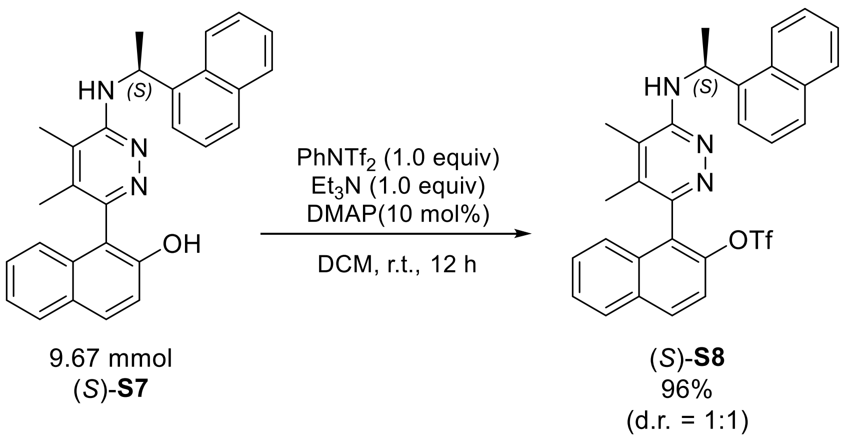

To a flask were added (*S*)-**S7** (4.0597 g, 9.67 mmol) and DMAP (121.2 mg, 0.98 mmol). After being evacuated and refilled with Ar for three times, DCM (96.7 mL), Et_3N (1.34 mL, d = 0.728 g/mL, 0.9785 g, 9.67 mmol), and *N*-phenyl-bis(trifluoromethanesulfonimide) (3.5255 g, 9.67 mmol) were added. The resulting solution was stirred at room temperature for 12 h as monitored by TLC, concentrated under reduced pressure directly. The residue was purified by chromatography on silica gel (eluent: petroleum ether/ethyl acetate = 10:1 (550 mL) to 5:1 (600 mL) to 3:1 (1200 mL) to 1:1 (800 mL)) to afford the crude product. The crude product was dissolved with 10 mL of dichloromethane and washed with a saturated aqueous solution of $NaHCO_3$ (3 × 10 mL), the organic layer was dried over anhydrous Na_2SO_4. After filtration and

evaporation, the pure (*S*)-**S8** (5.1132 g, 96%, d.r. = 1:1) (the d.r. of the product was determined via the NMR analysis) as a yellow foam: m.p. 101.9-103.1 °C (we were not able to obtain the crystal from the solvent tested, the m.p. was determined by using the solid after evaporation of the eluent); **^{1}H NMR** (400 MHz, $CDCl_3$) δ [8.31 (d, *J* = 8.4 Hz, 0.48 H), 8.17-8.12 (m, 0.50 H), 1 H, ArH], 7.97 (d, *J* = 9.2 Hz, 1 H, ArH), 7.94-7.83 (m, 2 H, ArH), 7.80 (d, *J* = 8.4 Hz, 1 H, ArH), [7.67 (d, *J* = 7.2 Hz, 0.49 H), 7.63 (d, *J* = 6.8 Hz, 0.53 H), 1 H, ArH], 7.57-7.43 (m, 7 H, ArH), 6.58-6.44 (m, 1 H, NCH), 4.64-4.54 (m, 1 H, NH), [2.03 (s, 1.49 H), 1.98 (s, 1.53 H), 3 H, CH_3], 1.90 (s, 3 H, CH_3), [1.85 (d, *J* = 6.4 Hz, 1.56 H), 1.81 (d, *J* = 6.4 Hz, 1.51 H), 3 H, CH_3]; **^{19}F NMR** (376 MHz, $CDCl_3$) δ -74.8, -74.9; **MS** (ESI) *m/z* 552 ([M+H]$^+$); **IR** (neat): ν = 3348, 3052, 2974, 1580, 1556, 1510, 1450, 1417, 1375, 1328, 1247, 1206, 1172, 1136, 1073 cm^{-1}; **HRMS** calcd for $C_{29}H_{25}F_3N_3O_3S$ ([M+H]$^+$): 552.1563. Found: 552.1548.

10. (*S*,*R*a)-6-(2-(Diphenylphosphanyl)naphthalen-1-yl)-4,5-dimethyl-*N*-(1-(naphth-1-yl)ethyl)pyridazin-3-amine (*S*,*R*a)-**L2** and (*S*,*S*a)-6-(2-(diphenylphosphanyl)naphthalene-1-yl)-4,5-dimethyl-*N*-(1-(naphthalen-1-yl)ethyl)pyridazin-3-amine (*S*,*S*a)-**L2** (xhb-4-026)

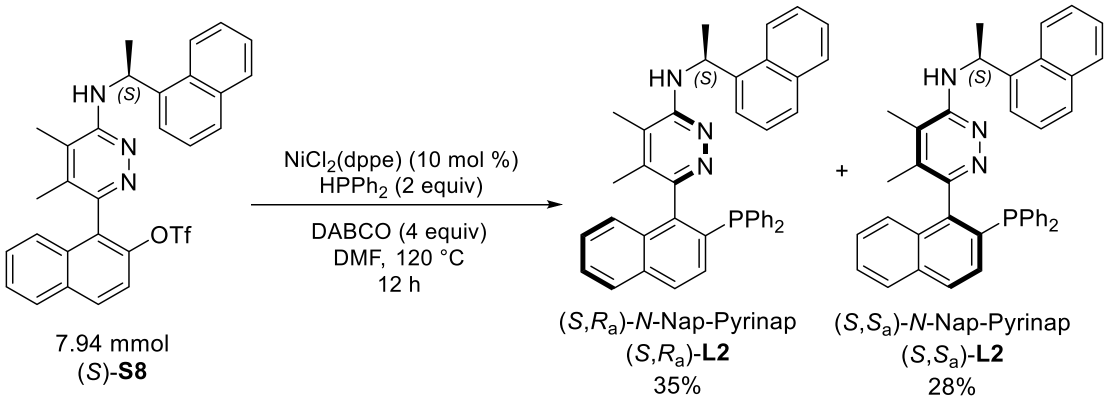

The flame-dried flask was evacuated and refilled with Ar for three times. Then Ni(dppe)Cl_2 (421.5 mg, 0.79 mmol) and diphenylphosphine (2.85 mL, d = 1.07 g/mL, 3.0481 g, 15.88 mmol)/DMF (18.8 mL) were added. After being stirred in an oil bath preheated at 120 °C for 30 min, a solution of (*S*)-**S8** (4.3805 g, 7.94 mmol) and DABCO (3.5992 g, 31.76 mmol) in 18.8 mL of DMF was added via syringe. The residue remaining in the flask was dissolved with 6.3 mL of DMF and the solution was also

added to flask via a syringe. The resulting solution was stirred at 120 °C for 12 h as monitored by TLC. After being cooled to room temperature, the resulting mixture was concentrated under reduced pressure (20 mbar, bath temperature: 60 °C). The residue was filtrated through a short column (5 cm) of silica gel eluted with toluene/acetone = 10: 1 (330 mL). After evaporation, the crude residue was purified by chromatography on silica gel (eluent: toluene/ethyl acetate = 40:1 (2460 mL) to 20:1 (1050 mL)) to afford (*S*,*R*a)-**L2** (1.6320 g, 35%, less polar) as a yellow foam and (*S*,*S*a)-**L2** (1.3056 g, 28%, more polar) as a yellow solid:

(*S*,*R*a)-**L2**: m.p. 208.6-209.8 °C (ethyl acetate/petroleum ether); $[\alpha]_D^{28}$ = +70.4 (c = 1.00, $CHCl_3$); **^{1}H NMR** (400 MHz, $CDCl_3$) δ 8.30 (d, *J* = 8.0 Hz, 1 H, ArH), 7.86-7.75 (m, 4 H, ArH), 7.65 (d, *J* = 7.2 Hz, 1 H, ArH), 7.51-7.42 (m, 4 H, ArH), 7.35-7.24 (m, 13 H, ArH), 6.54-6.45 (m, 1 H, NCH), 4.48 (d, *J* = 7.2 Hz, 1 H, NH), 1.95 (s, 3 H, CH_3), 1.78 (d, *J* = 6.4 Hz, 3 H, CH_3), 1.71 (s, 3 H, CH_3); **^{13}C NMR** (100 MHz, $CDCl_3$, This compound contains many *sp*^{2}-carbons, it's difficult to recognize the C-P couplings. So we list all the signals here.) δ 155.8, 153.53, 153.47, 143.6, 143.3, 140.3, 137.7, 137.6, 137.0, 136.9, 135.34, 135.32, 134.8, 134.7, 133.91, 133.86, 133.7, 133.5, 133.4, 133.2, 132.7, 132.6, 131.4, 129.8, 128.5, 128.4, 128.3, 128.23, 128.20, 128.14, 128.09, 127.9, 127.8, 126.7, 126.5, 126.1, 125.6, 125.2, 124.0, 122.4, 120.4, 46.4, 21.1, 15.44, 15.41, 11.7; **^{31}P NMR** (162 MHz, $CDCl_3$) δ -13.1; **MS** (ESI) *m/z* 588 ($[M+H]^+$); **IR** (neat): ν = 3364, 3054, 2971, 2922, 1738, 1583, 1560, 1480, 1433, 1394, 1372, 1311, 1239, 1179, 1141, 1088, 1069, 1048, 1025 cm^{-1}; **HRMS** calcd for $C_{40}H_{35}N_3P$ ($[M+H]^+$): 588.2563. Found: 588.2547;

(*S*,*S*a)-**L2**: m.p. 127.3-128.1 °C (ethyl acetate/petroleum ether); $[\alpha]_D^{29}$ = -136.2 (c = 1.00, $CHCl_3$); **^{1}H NMR** (400 MHz, $CDCl_3$) δ 8.29-8.21 (m, 1 H, ArH), 7.88-7.77 (m, 4 H, ArH), 7.65 (d, *J* = 7.2 Hz, 1 H, ArH), 7.50-7.44 (m, 4 H, ArH), 7.37-7.16 (m, 13 H, ArH), 6.50-6.40 (m, 1 H, NCH), 4.48 (d, *J* = 7.2 Hz, 1 H, NH), 1.92 (s, 3 H, CH_3), 1.82 (d, *J* = 6.4 Hz, 3 H, CH_3), 1.71 (s, 3 H, CH_3); **^{13}C NMR** (100 MHz, $CDCl_3$, This compound contains many *sp*^{2}-carbons, it's difficult to recognize the C-P couplings. So we list all the signals here.) δ 155.8, 153.44, 153.38, 143.4, 143.1, 140.2, 137.8, 137.7, 136.7, 136.6, 135.3, 135.25, 135.22, 135.1, 133.9, 133.7, 133.5, 133.41, 133.37, 132.6,

132.5, 131.5, 129.6, 128.5, 128.2, 128.15, 128.08, 127.9, 127.8, 126.6, 126.4, 126.2, 126.1, 126.0, 125.6, 125.2, 124.0, 122.4, 120.5, 46.4, 20.9, 15.39, 15.37, 11.6; **^{31}P NMR** (162 MHz, $CDCl_3$) δ -12.6; **MS** (ESI) *m/z* 588 ([M+H]$^+$); **IR** (neat): ν = 3341, 3048, 2974, 1731, 1581, 1552, 1478, 1433, 1370, 1320, 1237, 1176, 1132, 1090, 1068, 1024 cm^{-1}; **HRMS** calcd for $C_{40}H_{35}N_3P$ ([M+H]$^+$): 588.2563. Found: 588.2547.

3. Attempted synthesis of Pyriphen L3

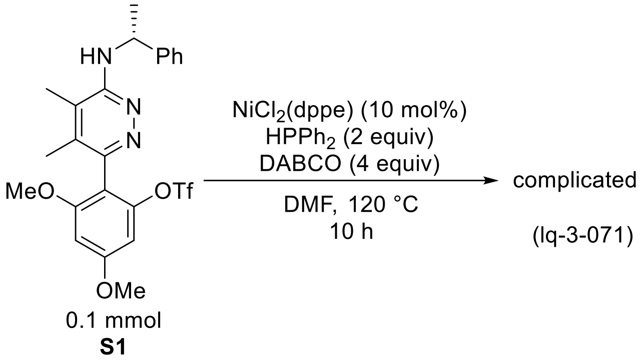

The flame-dried Schlenk tube was evacuated and back-filled with Ar for three times. Then Ni(dppe)Cl_2 (5.4 mg, 0.01 mmol) and diphenylphosphine (36 μL, d = 1.07 g/mL, 38.5 mg, 0.2 mmol)/DMF (0.2 mL) were added. After being stirred in an oil bath preheated at 120 °C for 30 min, a solution of **S1** (51.2 mg, 0.1 mmol) and DABCO (46.4 mg, 0.4 mmol) in 0.2 mL of DMF was added via syringe. The residue in the flask was dissolved with 0.1 mL of DMF and the solution was also added to Schlenk tube via a syringe. The resulting solution was stirred at 120 °C for 10 h as monitored by TLC. After being cooled to room temperature, the resulting mixture was concentrated under reduced pressure. The residue was filtrated through a short column (1 cm) of silica gel eluted with DCM/EE = 1:1 (60 mL). After evaporation, the crude residue was analyzed by ^{1}H NMR, and the reaction gave a complex mixture.

4. Synthesis of terminal alkynes 1p and 1u

1. Synthesis of terminal alkyne **1p** (lq-7-044)

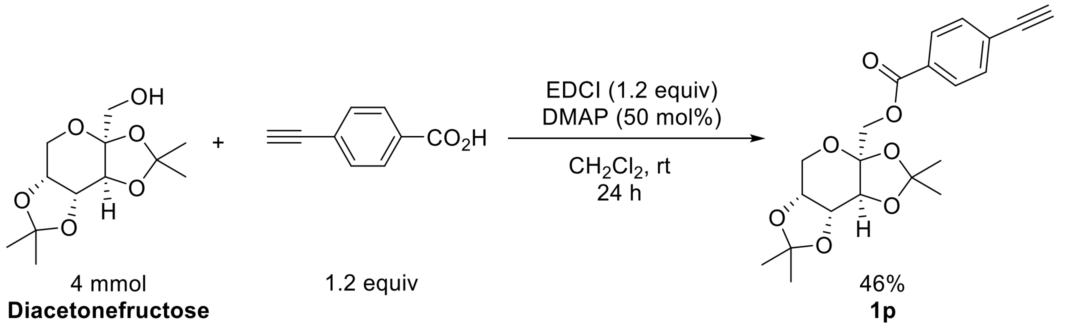

To an oven-dried round-bottomed flask were added 4-ethynylbenzoic acid (709.2 mg, 4.8 mmol), DCM (160 mL), and *N*-(3-Dimethylaminopropyl)-*N'*-ethylcarbodiimide hydrochloride (EDCI, 930.1 mg, 4.8 mmol) sequentially under Ar atmosphere. After being stirred at room temperature for 5 min, 4-dimethylaminopyridine (DMAP, 247.4 mg, 2 mmol) and diacetonefructose (1.0521 g, 4 mmol) were added. The resulting mixture was stirred at room temperature for 24 h as monitored by TLC, and then washed sequentially with a saturated aqueous solution of NH_4Cl (150 mL), H_2O (150 mL), and brine (150 mL). The organic layer was dried over anhydrous Na_2SO_4. After filtration and evaporation, the residue was purified by chromatography on silica gel to afford **1p** (717.7 mg, 46%) (eluent: petroleum ether/ethyl acetate = 10:1 (880 mL)) as a liquid: $[\alpha]_D^{30}$ = -25.8 (c = 1.025, $CHCl_3$); **^{1}H NMR** (400 MHz, $CDCl_3$) δ 8.03 (d, J = 8.4 Hz, 2 H, ArH), 7.55 (d, J = 8.4 Hz, 2 H, ArH), 4.70 (d, J = 12.0 Hz, 1 H, one proton of CH_2O(C=O)), 4.65 (dd, J_1 = 7.8 Hz, J_2 = 2.6 Hz, 1 H, OCH), 4.45 (d, J = 2.4 Hz, 1 H, OCH), 4.33 (d, J = 12.0 Hz, 1 H, one proton of CH_2O(C=O)), 4.27 (dd, J_1 = 8.0 Hz, J_2 = 0.8 Hz, 1 H, OCH), 3.96 (dd, J_1 = 13.0 Hz, J_2 = 1.8 Hz, 1 H, one proton of CH_2O), 3.81 (d, J = 12.8 Hz, 1 H, one proton of CH_2O), 3.26 (s, 1 H, C≡CH), 1.55 (s, 3 H, CH_3), 1.46 (s, 3 H, CH_3), 1.36 (s, 3 H, CH_3), 1.35 (s, 3 H, CH_3); **^{13}C NMR** (100 MHz, $CDCl_3$) δ 165.2, 132.0, 129.8, 129.6, 126.9, 109.1, 108.8, 101.5, 82.7, 80.3, 70.7, 70.5, 70.0, 65.5, 61.3, 26.4, 25.8, 25.4, 23.9; **MS** (ESI) *m/z* 406 ($[M+NH_4]^+$), 389 ($[M+H]^+$); **IR** (neat): ν = 3261, 2990, 2938, 2910, 1723, 1607, 1453, 1376, 1251, 1206, 1164, 1105, 1068, 1018, 753 cm^{-1}; **HRMS** calcd for $C_{21}H_{25}O_7$ ($[M+H]^+$): 389.1595, Found: 389.1595.

2. Synthesis of terminal alkyne **1u** (lq-7-055)

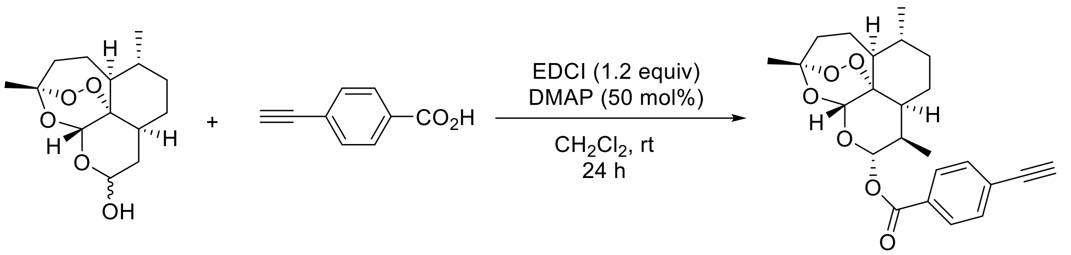

To an oven-dried round-bottomed flask were added 4-ethynylbenzoic acid (708.9 mg, 4.8 mmol), DCM (160 mL), *N*-(3-dimethylaminopropyl)-*N'*-ethylcarbodiimide hydrochloride (EDCI, 932.3 mg, 4.8 mmol), 4-dimethylaminopyridine (DMAP, 247.5 mg, 2 mmol), and dihydroartemisinin (1.1630 g, 4 mmol) sequentially under Ar atmosphere. The resulting mixture was stirred at room temperature for 24 h as monitored by TLC, and then washed sequentially with a saturated aqueous solution of NH_4Cl (150 mL), H_2O (150 mL), and brine (150 mL). The organic layer was dried over anhydrous Na_2SO_4. After filtration and evaporation, the residue was purified by chromatography on silica gel to afford **1u** (1.3826 g, 84%) (eluent: petroleum ether/ethyl acetate = 10:1 (1100 mL)) as a white solid: m.p. 152.5-153.2 °C (ethyl acetate/hexane); $[\alpha]_D^{26}$ = +26.0 (c = 1.015, $CHCl_3$); **^{1}H NMR** (400 MHz, $CDCl_3$) δ 8.08 (d, J = 8.0 Hz, 2 H, ArH), 7.55 (d, J = 8.0 Hz, 2 H, ArH), 6.00 (d, J = 9.6 Hz, 2 H, OCHO), 5.53 (s, 1 H, OCHO), 3.26 (s, 1 H, C≡CH), 2.82-2.69 (m, 1 H), 2.39 (td, J_1 = 13.9 Hz, J_2 = 3.73 Hz, 1 H), 2.05 (dt, J_1 = 14.0 Hz, J_2 = 5.2 Hz, 1 H), 1.97-1.63 (m, 4 H), 1.58-1.25 (m, 4 H), 1.43 (s, 3 H, CH_3), 1.10-0.96 (m, 1 H), 0.98 (d, J = 6.0 Hz, 3 H, CH_3), 0.92 (d, J = 7.2 Hz, 3 H, CH_3); **^{13}C NMR** (100 MHz, $CDCl_3$) δ 164.6, 132.0, 129.9, 129.6, 127.1, 104.4, 92.7, 91.5, 82.7, 80.3, 80.1, 51.6, 45.3, 37.2, 36.2, 34.0, 31.9, 25.9, 24.5, 22.0, 20.2, 12.2; **MS** (ESI) *m/z* 430 ($[M+NH_4]^+$); **IR** (neat): ν = 3270, 2999, 2963, 2924, 2875, 1719, 1608, 1453, 1409, 1392, 1376, 1357, 1313, 1267, 1209, 1179, 1129, 1086, 1052, 1031, 1005 cm^{-1}; Anal. Calcd. for $C_{24}H_{28}O_6$: C 69.89, H 6.84; Found: C 69.84, H 6.89.

5. Synthesis of chiral propargylic amines

1. (S)-4-Phenyl-4-(1-pyrrolidinyl)-2-butyn-1-ol (S)-**4aaa** (lq-5-006-b)

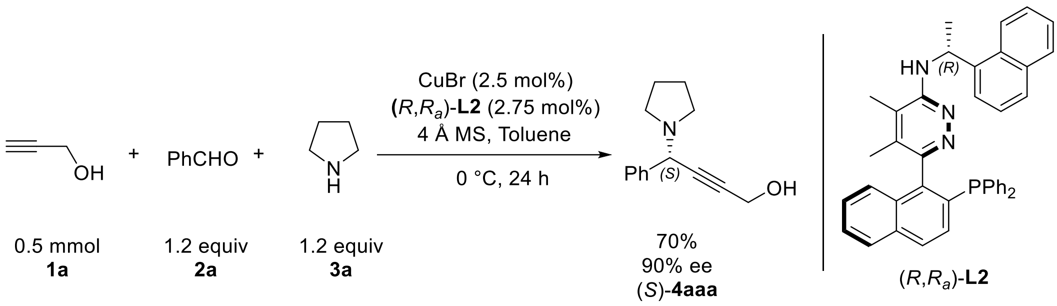

Typical Procedure I: To a flame-dried Schlenk tube were added CuBr (1.8 mg, 0.0125 mmol), (R,R_a)-**L2** (8.1 mg, 0.01375 mmol), 4 Å molecular sieves (150.5 mg), and toluene (0.75 mL) sequentially under Ar atmosphere. After being stirred at room temperature for 30 min, **1a** (28.0 mg, 0.5 mmol), and **2a** (63.7 mg, 0.6 mmol)/toluene (0.5 mL) were added sequentially under Ar atmosphere. The resulting mixture was stirred at 0 °C for another 10 min followed by the addition of pyrrolidine **3a** (42.7 mg, 0.6 mmol). After being stirred at 0 °C for 24 h, the reaction was complete as monitored by TLC. The resulting mixture was filtrated through a short pad of basic aluminum oxide (200-300 mesh) eluted with dichloromethane/MeOH (10:1, 44 mL). After evaporation, the residue was purified by chromatography on silica gel (eluent: petroleum ether/ethyl acetate = 2:1) to afford (S)-**4aaa** (74.9 mg, 70%) as a liquid: 90% ee (HPLC conditions: Chiralcel OD-H column, hexane/i-PrOH = 95/5, 1.2 mL/min, λ = 214 nm, t_R(major) = 10.1 min, t_R(minor) = 7.7 min); $[\alpha]_D^{31}$ = -28.1 (c = 1.05, $CHCl_3$) (reported value for (R)-isomer: 98% ee, $[\alpha]_D^{20}$ = +25.30 (c = 1, $CHCl_3$)[2]); **^{1}H NMR** (400 MHz, $CDCl_3$) δ 7.51 (d, J = 7.2 Hz, 2 H, ArH), 7.33 (t, J = 7.2 Hz, 2 H, ArH), 7.27 (t, J = 6.4 Hz, 1 H, ArH), 4.62 (s, 1 H, CH), 4.36 (d, J = 1.6 Hz, 2 H, OCH_2), 2.67-2.51 (m, 4 H, 2 × NCH_2), 2.04 (s, 1 H, OH), 1.83-1.71 (m, 4 H, 2 × CH_2); **^{13}C NMR** (100 MHz, $CDCl_3$) δ 138.9, 128.24, 128.21, 127.7, 85.0, 82.7, 58.9, 50.9, 50.5, 23.2; **MS** (EI) m/z (%) 215 (M^+, 20.49), 138 (100); **IR** (neat): ν = 3065, 2960, 2924, 2871, 2841, 2729, 1489, 1455, 1372, 1345, 1309, 1270, 1233, 1206, 1121, 1087, 1074, 1033, 1024 cm^{-1}; **HRMS** calcd for $C_{14}H_{18}NO$ ($[M+H]^+$): 216.1383, Found: 216.1381.

2. (*S*)-4-(4-Chlorophenyl)-4-(1-pyrrolidinyl)-2-butyn-1-ol (*S*)-**4aba** (Lq-5-020)

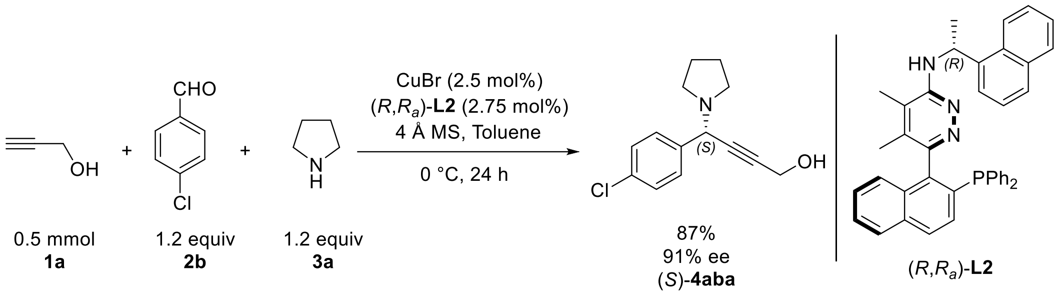

Following **Typical Procedure I**, the reaction of CuBr (1.8 mg, 0.0125 mmol), (*R*,*R*a)-**L2** (8.1 mg, 0.01375 mmol), 4 Å molecular sieves (150.2 mg)/toluene (0.75 mL), **1a** (28.4 mg, 0.5 mmol), **2b** (84.6 mg, 0.6 mmol)/toluene (0.5 mL), and pyrrolidine **3a** (43.2 mg, 0.6 mmol) afforded (*S*)-**4aba** (109.3 mg, 87%) (eluent: petroleum ether/ethyl acetate = 2:1) as a liquid: 91% ee (HPLC conditions: Chiralcel AD-H column, hexane/*i*-PrOH = 80/20, 1 mL/min, λ = 214 nm, t_R(major) = 4.8 min, t_R(minor) = 4.3 min); $[\alpha]_D^{30}$ = -31.1 (c = 1.05, $CHCl_3$); **^{1}H NMR** (400 MHz, $CDCl_3$) δ 7.45 (d, *J* = 8.4 Hz, 2 H, ArH), 7.30 (d, *J* = 8.4 Hz, 2 H, ArH), 4.62 (s, 1 H, NCH), 4.37 (d, *J* = 1.6 Hz, 2 H, OCH_2), 2.65-2.50 (m, 4 H, 2 × NCH_2), 1.91 (br, 1 H, OH), 1.82-1.71 (m, 4 H, 2 × CH_2); **^{13}C NMR** (100 MHz, $CDCl_3$) δ 137.5, 133.4, 129.5, 128.4, 85.4, 82.0, 58.2, 50.8, 50.4, 23.2; **MS** (ESI) *m/z* 252 ([M(^{37}Cl)+H]$^+$), 250 ([M(^{35}Cl)+H]$^+$); **IR** (neat): ν = 3280, 2965, 2932, 2876, 2811, 1595, 1578, 1489, 1460, 1406, 1346, 1290, 1261, 1234, 1199, 1121, 1089, 1014 cm^{-1}; **HRMS** calcd for $C_{14}H_{17}{}^{35}ClNO$ ([M+H]$^+$): 250.0993, Found: 250.0996.

3. (*S*)-4-(Pyrrolidin-1-yl)-4-(*p*-tolyl)-2-butyn-1-ol (*S*)-**4aca** (Lq-5-101)

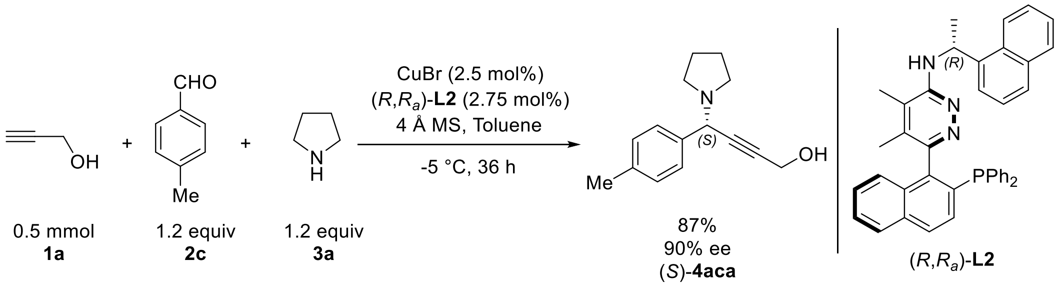

Following **Typical Procedure I**, the reaction of CuBr (1.8 mg, 0.0125 mmol), (*R*,*R*a)-**L2** (8.1 mg, 0.01375 mmol), 4 Å molecular sieves (151.2 mg)/toluene (0.75 mL),

1a (28.3 mg, 0.5 mmol), **2c** (73.2 mg, 0.6 mmol)/toluene (0.5 mL), and pyrrolidine **3a** (42.8 mg, 0.6 mmol) afforded (*S*)-**4aca** (100.3 mg, 87%) (eluent: petroleum ether/ethyl acetate = 2:1 (150 mL) to 1:1 (600 mL) to 2:3 (500 mL), it should be noted that the column packed with silica gel was eluted with a mixture of petroleum ether (50 mL) and Et_3N (0.25 mL) before loading the sample) as a liquid: 90% ee (HPLC conditions: Chiralcel AD-H column, hexane/*i*-PrOH = 95/5, 1.0 mL min^{-1}, λ = 214 nm, t_R(major) = 11.3 min, t_R(minor) = 8.4 min); $[\alpha]_D^{28}$ = -24.0 (c = 1.06, $CHCl_3$); **^{1}H NMR** (400 MHz, $CDCl_3$) δ 7.37 (d, *J* = 8.0 Hz, 2 H, ArH), 7.12 (d, *J* = 8.0 Hz, 2 H, ArH), 4.52 (s, 1 H, NCH), 4.31 (d, 2 H, OCH_2), 3.53-3.23 (br, 1 H, OH), 2.67-2.49 (m, 4 H, 2 × CH_2), 2.32 (s, 3 H, CH_3), 1.84-1.67 (m, 4 H, 2 × CH_2); **^{13}C NMR** (100 MHz, $CDCl_3$) δ 137.3, 135.9, 128.9, 128.1, 84.8, 82.8, 58.7, 50.8, 50.5, 23.2, 21.0; **MS** (ESI) *m/z* 230 ($[M+H]^+$); **IR** (neat): ν = 3289, 2965, 2922, 2875, 2810, 1614, 1512, 1458, 1345, 1300, 1264, 1215, 1200, 1180, 1093, 1020 cm^{-1}; **HRMS** calcd for $C_{15}H_{20}NO$ ($[M+H]^+$): 230.1539, Found: 230.1541.

4. (*S*)-4-(4-(Benzyloxy)phenyl)-4-(1-pyrrolidinyl)-2-butyn-1-ol (*S*)-**4ada** (Lq-5-094)

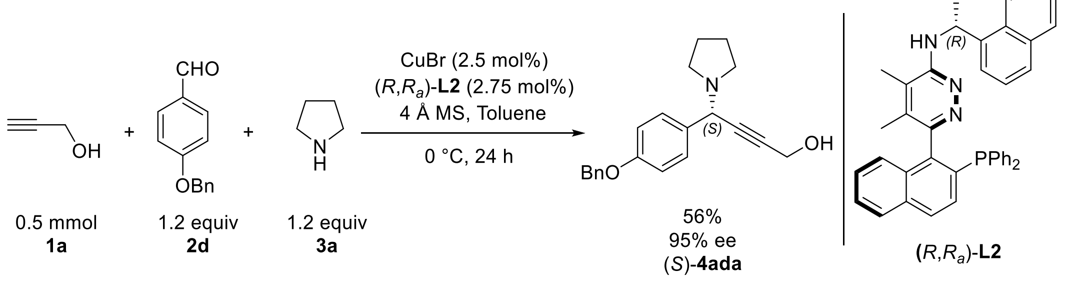

Following **Typical Procedure I**, the reaction of CuBr (1.8 mg, 0.0125 mmol), (*R*,*R*a)-**L2** (8.1 mg, 0.01375 mmol), 4 Å molecular sieves (151.0 mg)/toluene (0.75 mL), **1a** (28.1 mg, 0.5 mmol), **2d** (131.9 mg, 0.6 mmol)/toluene (0.5 mL), and pyrrolidine **3a** (42.9 mg, 0.6 mmol) afforded (*S*)-**4ada** (90.3 mg, 56%) (eluent: petroleum ether/ethyl acetate = 2:1 (150 mL) to 1:1 (400 mL) to 1:2 (600 mL)) as a liquid: 95% ee (HPLC conditions: Chiralcel AD-H column, hexane/*i*-PrOH = 95/5, 1.0 mL min^{-1}, λ = 214 nm, t_R(major) = 32.9 min, t_R(minor) = 24.5 min); $[\alpha]_D^{29}$ = -15.1 (c = 1.03, $CHCl_3$); **^{1}H NMR**

(400 MHz, $CDCl_3$) δ 7.46-7.27 (m, 7 H, ArH), 6.92 (d, *J* = 8.8 Hz, 2 H, ArH), 5.01 (s, 2 H, CH_2), 4.50 (s, 1 H, NCH), 4.30 (d, *J* = 1.2 Hz, 2 H, OCH_2), 3.44 (br, 1 H, OH), 2.68-2.45 (m, 4 H, 2 × CH_2), 1.84-1.65 (m, 4 H, 2 × CH_2); **^{13}C NMR** (100 MHz, $CDCl_3$) δ 158.2, 136.8, 131.4, 129.3, 128.5, 127.9, 127.4, 114.4, 84.9, 82.7, 69.9, 58.3, 50.7, 50.4, 23.1; **MS** (ESI) *m/z* 322 ($[M+H]^+$); **IR** (neat): ν = 3366, 3063, 3033, 2963, 2928, 2872, 2809, 1609, 1584, 1508, 1454, 1381, 1345, 1302, 1237, 1173, 1094, 1015 cm^{-1}; **HRMS** calcd for $C_{21}H_{24}NO_2$ ($[M+H]^+$): 322.1802, Found: 322.1806.

5. (*S*)-4-(3-Methoxyphenyl)-4-(1-pyrrolidinyl)-2-butyn-1-ol (*S*)-**4aea** (Lq-5-025)

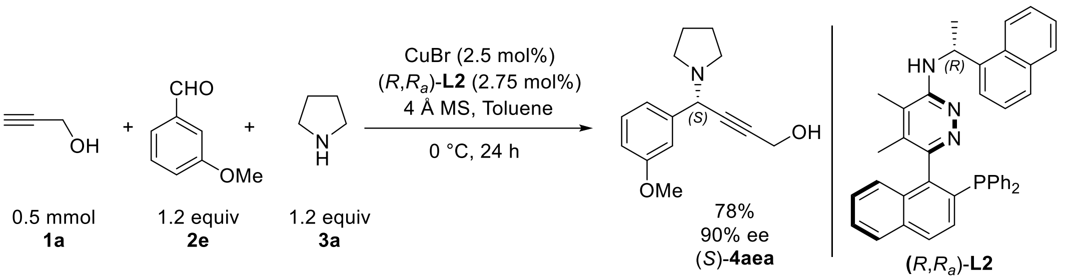

Following **Typical Procedure I**, the reaction of CuBr (1.8 mg, 0.0125 mmol), (*R*,*R*a)-**L2** (8.1 mg, 0.01375 mmol), 4 Å molecular sieves (150.7 mg)/toluene (0.75 mL), **1a** (28.4 mg, 0.5 mmol), **2e** (82.4 mg, 0.6 mmol)/toluene (0.5 mL), and pyrrolidine **3a** (43.2 mg, 0.6 mmol) afforded (*S*)-**4aea** (97.1 mg, 78%) (eluent: petroleum ether/ethyl acetate = 1:1) as a liquid: 90% ee (HPLC conditions: Chiralcel AD-H column, hexane/*i*-PrOH = 90/10, 0.8 mL min^{-1}, λ = 214 nm, t_R(major) = 9.8 min, t_R(minor) = 8.7 min); $[\alpha]_D^{28}$ = -30.4 (c = 0.95, $CHCl_3$); **^{1}H NMR** (400 MHz, $CDCl_3$) δ 7.24 (t, *J* = 8.2 Hz, 1 H, ArH), 7.12-7.06 (m, 2 H, ArH), 6.85-6.79 (m, 1 H, ArH), 4.56 (s, 1 H, NCH), 4.35 (d, *J* = 1.6 Hz, 2 H, OCH_2), 3.81 (s, 3 H, OCH_3), 2.67-2.52 (m, 4 H, 2 × NCH_2), 2.33-1.88 (br, 1 H, OH), 1.83-1.69 (m, 4 H, 2 × CH_2);**^{13}C NMR** (100 MHz, $CDCl_3$) δ 159.5, 140.5, 129.2, 120.6, 113.8, 113.1, 85.0, 82.6, 59.0, 55.2, 50.7, 50.6, 23.2; **MS** (ESI) *m/z* 246 ($[M+H]^+$); **IR** (neat): ν = 3348, 2962, 2875, 2834, 1600, 1586, 1488, 1453, 1434, 1357, 1345, 1313, 1265, 1248, 1155, 1120, 1094, 1081, 1021 cm^{-1}; **HRMS** calcd for $C_{15}H_{20}NO_2$ ($[M+H]^+$): 246.1489, Found: 246.1488.

6. (*S*)-4-([1,1'-Biphenyl])-4-(1-pyrrolidinyl)-2-butyn-1-ol (*S*)-**4afa** (Lq-5-037)

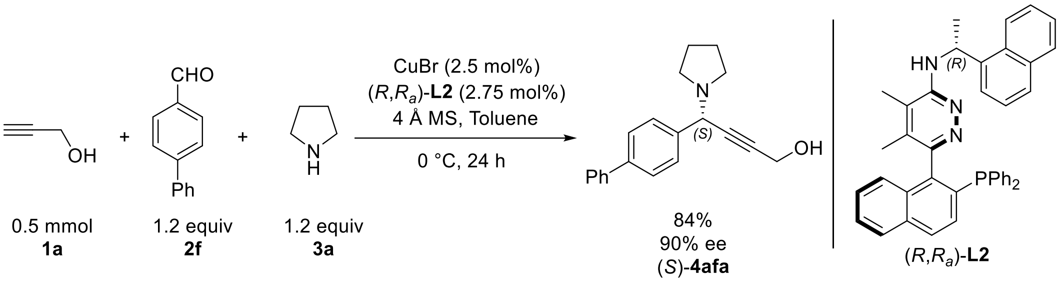

Following **Typical Procedure I**, the reaction of CuBr (1.8 mg, 0.0125 mmol), (R,R_a)-**L2** (8.1 mg, 0.01375 mmol), 4 Å molecular sieves (150.5 mg)/toluene (0.75 mL), **1a** (28.1 mg, 0.5 mmol), **2f** (111.6 mg, 0.6 mmol)/toluene (0.5 mL), and pyrrolidine **3a** (43.5 mg, 0.6 mmol) afforded (*S*)-**4afa** (123.1 mg, 84%) (eluent: petroleum ether/ethyl acetate = 3:1 (400 mL) to 2:1 (600 mL)) as a liquid: 90% ee (HPLC conditions: Chiralcel OD-H column, hexane/*i*-PrOH = 95/5, 1.0 mL min^{-1}, λ = 214 nm, t_R(major) = 12.6 min, t_R(minor) = 4.8 min); $[\alpha]_D^{28}$ = -27.3 (c = 1.035, $CHCl_3$); **^{1}H NMR** (400 MHz, $CDCl_3$) δ 7.61-7.51 (m, 6 H, ArH), 7.42 (t, J = 7.8 Hz, 2 H, ArH), 7.33 (t, J = 7.2 Hz, 1 H, ArH), 4.61 (s, 1 H, NCH), 4.35 (d, J = 1.6 Hz, 2 H, OCH_2), 2.98-2.70 (br, 1 H, OH), 2.69-2.52 (m, 4 H, 2 × NCH_2), 1.86-1.70 (m, 4 H, 2 ×CH_2); **^{13}C NMR** (100 MHz, $CDCl_3$) δ 140.7, 140.6, 138.0, 128.7, 128.6, 127.2, 127.03, 126.99, 85.1, 82.6, 58.7, 50.9, 50.6, 23.2; **MS** (ESI) *m/z* 292 ([M+H]$^+$); **IR** (neat): ν = 3342, 3057, 3028, 2963, 2874, 2810, 1599, 1486, 1460, 1448, 1407, 1345, 1296, 1260, 1199, 1092, 1020 cm^{-1}; **HRMS** calcd for $C_{20}H_{22}NO$ ([M+H]$^+$): 292.1696, Found: 292.1692.

7. (*S*)-4-(4-Cyanophenyl)-4-(1-pyrrolidinyl)-2-butyn-1-ol (*S*)-**4aga** (Lq-5-019)

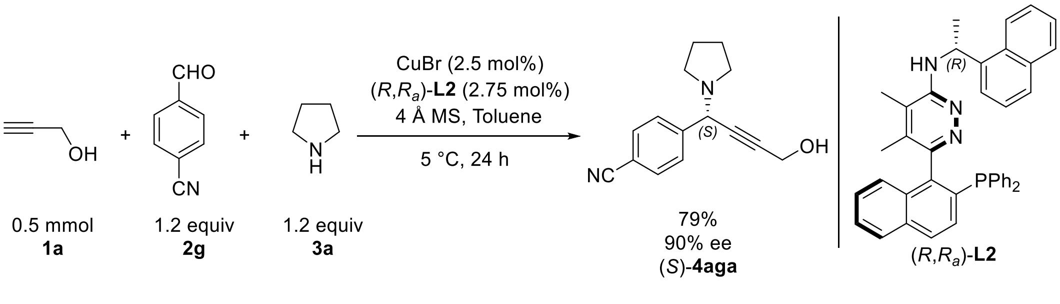

Following **Typical Procedure I**, the reaction of CuBr (1.8 mg, 0.0125 mmol), (R,R_a)-**L2** (8.1 mg, 0.01375 mmol), 4 Å molecular sieves (150.0 mg)/toluene (0.75 mL), **1a** (28.4 mg, 0.5 mmol), **2g** (80.5 mg, 0.6 mmol)/toluene (0.5 mL), and pyrrolidine **3a** (43.2 mg, 0.6 mmol) afforded (*S*)-**4aga** (95.6 mg, 79%) (eluent: petroleum ether/ethyl

acetate = 2:1) as a liquid: 90% ee (HPLC conditions: Chiralcel AD-H column, hexane/*i*-PrOH = 80/20, 1 mL/min, λ = 214 nm, t_R(major) = 7.4 min, t_R(minor) = 5.7 min); $[\alpha]_D^{30}$ = -34.8 (c = 1.04, $CHCl_3$); **^{1}H NMR** (400 MHz, $CDCl_3$) δ 7.67 (d, *J* = 8.4 Hz, 2 H, ArH), 7.63 (d, *J* = 8.4 Hz, 2 H, ArH), 4.75 (s, 1 H, CH), 4.39 (d, *J* = 1.6 Hz, 2 H, OCH_2), 2.64-2.51 (m, 4 H, 2 × NCH_2), 1.96-1.71 (m, 5 H, OH + 2 × CH_2); **^{13}C NMR** (100 MHz, $CDCl_3$) δ 144.5, 132.0, 128.8, 118.7, 111.3, 86.2, 80.9, 58.1, 50.8, 50.1, 23.3; **MS** (ESI) *m/z* 241 ($[M+H]^+$); **IR** (neat): ν = 3395, 2964, 2932, 2875, 2815, 2228, 1607, 1502, 1460, 1411, 1357, 1346, 1293, 1263, 1199, 1092, 1018 cm^{-1}; **HRMS** calcd for $C_{15}H_{17}N_2O$ ($[M+H]^+$): 241.1335, Found: 241.1335.

8. Methyl (*S*)-4-(4-hydroxy-1-(pyrrolidin-1-yl)but-2-yn-1-yl)benzoate (*S*)-**4aha** (Lq-5-076)

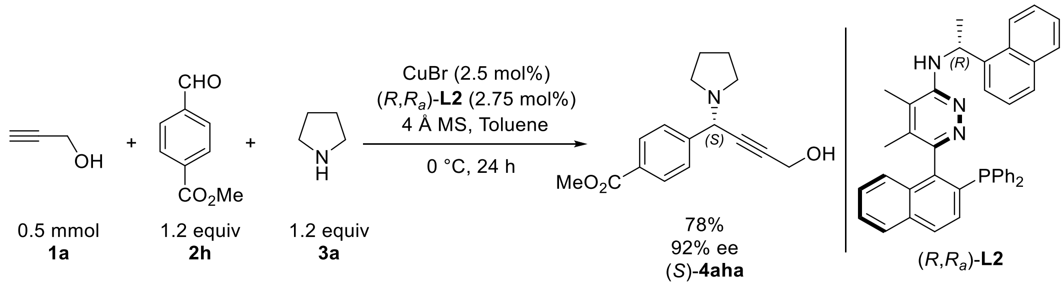

Following **Typical Procedure I**, the reaction of CuBr (1.8 mg, 0.0125 mmol), (*R*,*R*a)-**L2** (8.1 mg, 0.01375 mmol), 4 Å molecular sieves (150.0 mg)/toluene (0.75 mL), **1a** (28.2 mg, 0.5 mmol), **2h** (101.6 mg, 0.6 mmol)/toluene (0.5 mL), and pyrrolidine **3a** (43.0 mg, 0.6 mmol) afforded (*S*)-**4aha** (107.7 mg, 78%) (eluent: petroleum ether/ethyl acetate = 2:1, it should be noted that the column packed with silica gel was eluted with a mixture of petroleum ether (50 mL) and Et_3N (0.25 mL) before loading the sample) as a liquid: 92% ee (HPLC conditions: Chiralcel OD-H column, hexane/*i*-PrOH = 95/5, 1.0 mL min^{-1}, λ = 214 nm, t_R(major) = 17.0 min, t_R(minor) = 21.5 min); $[\alpha]_D^{28}$ = -30.3 (c = 1.01, $CHCl_3$); **^{1}H NMR** (400 MHz, $CDCl_3$) δ 8.00 (d, *J* = 8.4 Hz, 2 H, ArH), 7.59 (d, *J* = 8.4 Hz, 2 H, ArH), 4.66 (s, 1 H, NCH), 4.36 (d, *J* = 2.0 Hz, 2 H, OCH_2), 3.91 (s, 3 H, CH_3), 2.89 (br, 1 H, OH), 2.66-2.49 (m, 4 H, 2 × CH_2), 1.85-1.67 (m, 4 H, 2 × CH_2); **^{13}C NMR** (100 MHz, $CDCl_3$) δ 166.9, 144.1, 129.6, 129.4, 128.1, 85.7, 81.7, 58.4, 52.1, 50.8, 50.3, 23.2; **MS** (ESI) *m/z* 274 ($[M+H]^+$); **IR** (neat): ν = 3385, 2952,

2876, 2812, 1719, 1610, 1576, 1435, 1412, 1358, 1276, 1191, 1102, 1018 cm^{-1}; **HRMS** calcd for $C_{16}H_{20}NO_3$ ([M+H]$^+$): 274.1438, Found: 274.1436.

9. (*S*)-4-(Pyrrolidin-1-yl)-4-(4-(trifluoromethyl)phenyl)but-2-yn-1-ol (*S*)-**4aia** (Lq-5-090)

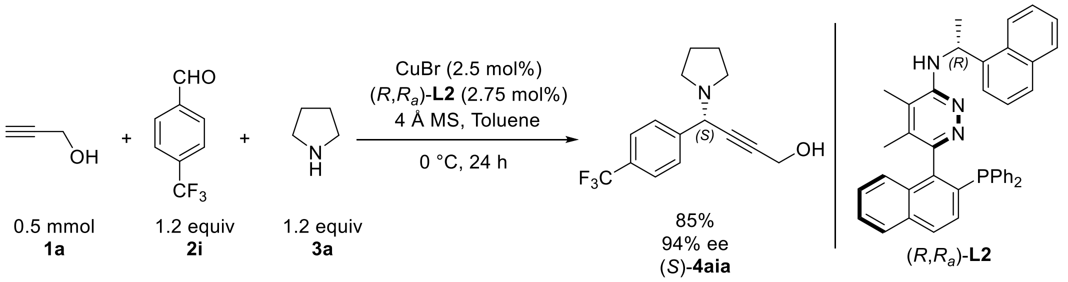

Following **Typical Procedure I**, the reaction of CuBr (1.8 mg, 0.0125 mmol), (*R*,*R*a)-**L2** (8.1 mg, 0.01375 mmol), 4 Å molecular sieves (150.4 mg)/toluene (0.75 mL), **1a** (28.2 mg, 0.5 mmol), **2i** (109.7 mg, 0.6 mmol)/toluene (0.5 mL), and pyrrolidine **3a** (43.0 mg, 0.6 mmol) afforded (*S*)-**4aia** (121.1 mg, 85%) (eluent: petroleum ether/ethyl acetate = 4:1(250 mL) to 2:1 (600 mL)) as a liquid: 94% ee (HPLC conditions: Chiralcel AD-H column, hexane/*i*-PrOH = 95/5, 1 mL/min, λ = 214 nm, t_R(major) = 11.1 min, t_R(minor) = 8.7 min); $[\alpha]_D^{29}$ = -26.5 (c = 1.025, $CHCl_3$); **^{1}H NMR** (400 MHz, $CDCl_3$) δ 7.64 (d, *J* = 8.1 Hz, 2 H, ArH), 7.57 (d, *J* = 8.2 Hz, 2 H, ArH), 4.65 (s, 1 H, NCH), 4.35 (d, *J* = 1.6 Hz, 2 H, OCH_2), 3.28 (br, 1 H, OH), 2.66-2.47 (m, 4 H, 2 × CH_2),1.85-1.66 (m, 4 H, 2 × CH_2); **^{13}C NMR** (100 MHz, $CDCl_3$) δ 143.0, 129.9 (q, *J* = 31.9 Hz), 128.5, 125.2 (q, *J* = 3.6 Hz), 124.0 (q, *J* = 270.4 Hz), 85.8, 81.6, 58.4, 50.7, 50.4, 23.2; **^{19}F NMR** (376 MHz, $CDCl_3$) δ -63.0; **MS** (ESI) *m/z* 284 ([M+H]$^+$); **IR** (neat): ν = 3295, 2967, 2814, 1619, 1416, 1322, 1269, 1162, 1120, 1065, 1017 cm^{-1}; **HRMS** calcd for $C_{15}H_{17}F_3NO$ ([M+H]$^+$): 284.1257, Found: 284.1264.

10. (*R*)-4-(2-Fluorophenyl)-4-(1-pyrrolidinyl)-2-butyn-1-ol (*R*)-**4aja** (Lq-5-124)

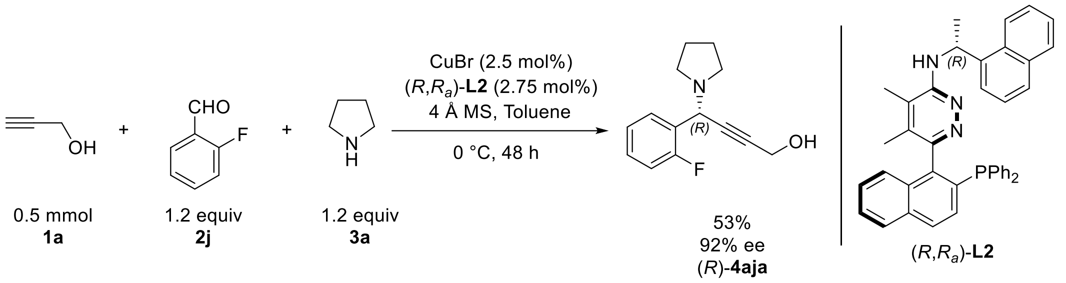

Following **Typical Procedure I**, the reaction of CuBr (1.8 mg, 0.0125 mmol), (R,R_a)-**L2** (8.1 mg, 0.01375 mmol), 4 Å molecular sieves (150.7 mg)/toluene (0.75 mL), **1a** (28.4 mg, 0.5 mmol), **2j** (76.3 mg, 0.6 mmol)/toluene (0.5 mL), and pyrrolidine **3a** (42.9 mg, 0.6 mmol) afforded (*R*)-**4aja** (63.1 mg, 53%) (eluent: petroleum ether/ethyl acetate = 2:1 (900 mL)) as a liquid: 92% ee (HPLC conditions: Chiralcel OD-H column, hexane/*i*-PrOH = 95/5, 1 mL/min, λ = 214 nm, t_R(major) = 12.0 min, t_R(minor) = 9.1 min); $[\alpha]_D^{26}$ = -3.9 (c = 1.105, $CHCl_3$); **^{1}H NMR** (400 MHz, $CDCl_3$) δ 7.62 (td, J_1 = 7.6 Hz, J_2 = 1.6 Hz, 1 H, ArH), 7.30-7.22 (m, 1 H, ArH), 7.47 (td, J_1 = 7.5 Hz, J_2 = 1.1 Hz, 1 H, ArH), 7.07-6.99 (m, 1 H, ArH), 4.92 (t, J = 1.6 Hz, 1 H, NCH), 4.32 (d, J = 2.0 Hz, 2 H, OCH_2), 2.97 (br, 1 H, OH), 2.70-2.53 (m, 4 H, 2 × NCH_2), 1.82-1.69 (m, 4 H, 2 × CH_2); **^{13}C NMR** (100 MHz, $CDCl_3$) δ 160.1 (d, J = 245.9 Hz), 130.2 (d, J = 3.8 Hz), 129.4 (d, J = 8.5 Hz), 125.9 (d, J = 13.0 Hz), 123.9 (d, J = 3.8 Hz), 115.4 (d, J = 22.2 Hz), 84.1, 82.3, 51.5 (d, J = 3.0 Hz), 50.9, 50.6, 23.1; **^{19}F NMR** (376 MHz, $CDCl_3$) δ -118.9; **MS** (ESI) *m/z* 234 ($[M+H]^+$); **IR** (neat): ν = 3203, 2964, 2876, 2812, 1615, 1588, 1488, 1456, 1359, 1347, 1306, 1267, 1230, 1174, 1082, 1019 cm^{-1}; **HRMS** calcd for $C_{14}H_{17}FNO$ ($[M+H]^+$): 234.1289, Found: 234.1294.

11. (*S*)-4-(3-Fluorophenyl)-4-(1-pyrrolidinyl)-2-butyn-1-ol (*S*)-**4aka** (Lq-5-112)

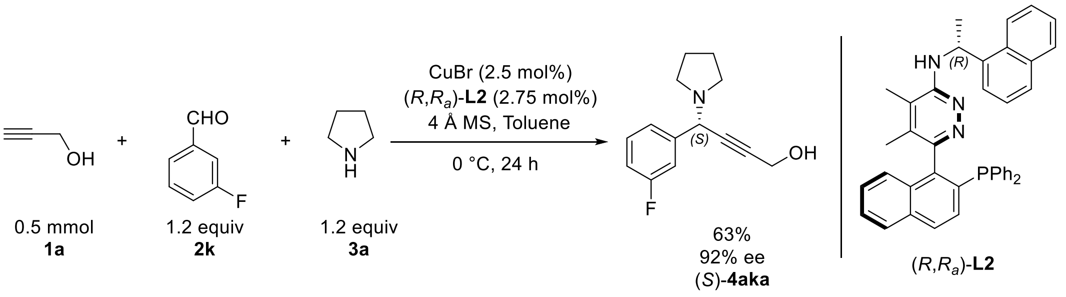

Following **Typical Procedure I**, the reaction of CuBr (1.8 mg, 0.0125 mmol), (R,R_a)-**L2** (8.1 mg, 0.01375 mmol), 4 Å molecular sieves (150.5 mg)/toluene (0.75 mL),

1a (28.2 mg, 0.5 mmol), **2k** (77.0 mg, 0.6 mmol)/toluene (0.5 mL), and pyrrolidine **3a** (42.9 mg, 0.6 mmol) afforded (*S*)-**4aka** (74.1 mg, 63%) (eluent: petroleum ether/ethyl acetate = 4:1 (250 mL) to 2:1 (900 mL)) as a liquid: 92% ee (HPLC conditions: Chiralcel OD-Hcolumn, hexane/*i*-PrOH = 95/5, 0.7 mL/min, λ = 214 nm, t_R(major) = 12.8 min, t_R(minor) = 11.9 min); $[\alpha]_D^{27}$ = -28.8 (c = 1.05, $CHCl_3$); **^{1}H NMR** (400 MHz, $CDCl_3$) δ 7.34-7.21 (m, 3 H, ArH), 7.02-6.92 (m, 1 H, ArH), 4.66 (s, 1 H, NCH), 4.38 (d, *J* = 1.6 Hz, 2 H, OCH_2), 2.67-2.51 (m, 4 H, 2 × CH_2), 2.16 (br, 1 H, OH), 1.85-1.71 (m, 4 H, 2 × CH_2); **^{13}C NMR** (100 MHz, $CDCl_3$) δ 162.7 (d, *J* = 244.3 Hz), 141.5 (d, *J* = 6.9 Hz), 129.6 (d, *J* = 8.5 Hz), 123.8 (d, *J* = 2.3 Hz), 115.1 (d, *J* = 22.2 Hz), 114.5 (d, *J* = 20.7 Hz), 85.5, 81.7, 58.2, 50.7, 50.2, 23.2; **^{19}F NMR** (376 MHz, $CDCl_3$) δ -113.7; **MS** (ESI) *m/z* 234 ($[M+H]^+$); **IR** (neat): ν = 3328, 3064, 2964, 2932, 2876, 2813, 1614, 1590, 1485, 1445, 1357, 1347, 1305, 1263, 1241, 1129, 1092, 1071, 1018 cm^{-1}; **HRMS** calcd for $C_{14}H_{17}FNO$ ($[M+H]^+$): 234.1289, Found: 234.1288.

12. (*S*)-4-(4-Fluorophenyl)-4-(1-pyrrolidinyl)-2-butyn-1-ol (*S*)-**4ala** (Lq-5-029)

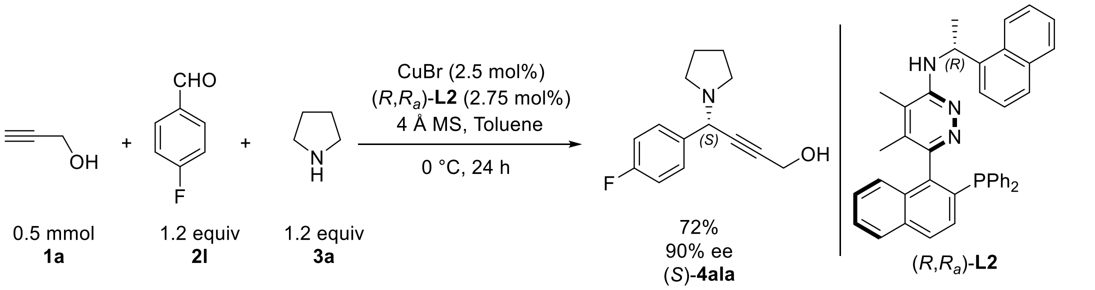

Following **Typical Procedure I**, the reaction of CuBr (1.8 mg, 0.0125 mmol), (*R*,*R*a)-**L2** (8.1 mg, 0.01375 mmol), 4 Å molecular sieves (150.8 mg)/toluene (0.75 mL), **1a** (28.2 mg, 0.5 mmol), **2l** (75.2 mg, 0.6 mmol)/toluene (0.5 mL), and pyrrolidine **3a** (43.4 mg, 0.6 mmol) afforded (*S*)-**4ala** (84.6 mg, 72%) (eluent: petroleum ether/ethyl acetate = 2:1 (900 mL)) as a liquid: 90% ee (HPLC conditions: Chiralcel AD-H column, hexane/*i*-PrOH = 90/10, 0.7 mL/min, λ = 214 nm, t_R(major) = 10.2 min, t_R(minor) = 8.4 min); $[\alpha]_D^{29}$ = -30.2 (c = 1.05, $CHCl_3$); **^{1}H NMR** (400 MHz, $CDCl_3$) δ 7.47 (dd, J_1 = 8.0 Hz, J_2 = 5.6 Hz, 2 H, ArH), 7.01 (t, *J* = 8.6, 2 H, ArH), 4.57 (s, 1 H, NCH), 4.34 (d, *J* = 1.6 Hz, 2 H, OCH_2), 2.82-2.46 (m, 5 H, OH + 2 × NCH_2), 1.86-1.68 (m, 4 H, 2 × CH_2); **^{13}C NMR** (100 MHz, $CDCl_3$) δ 162.2 (d, *J* = 244.8 Hz), 134.8 (d, *J* = 3.1 Hz),

129.8 (d, J = 7.9 Hz), 115.0 (d, J = 21.3 Hz), 85.2, 82.5, 58.1, 50.9, 50.4, 23.2; **^{19}F NMR** (376 MHz, $CDCl_3$) δ -115.5; **MS** (ESI) *m/z* 234 ($[M+H]^+$); **IR** (neat): ν = 3334, 2966, 2930, 2878, 2824, 1604, 1508, 1460, 1418, 1346, 1296, 1265, 1222, 1158, 1120, 1089, 1017 cm^{-1}; **HRMS** calcd for $C_{14}H_{17}FNO$ ($[M+H]^+$): 234.1289, Found: 234.1289.

13. (*S*)-4-(4-((*R*a)-2-phenylocta-2,3-dien-4-yl)phenyl)-4-(1-pyrrolidinyl)but-2-yn-1-ol (*S*,*R*a)-**4ama** (lq-7-076)

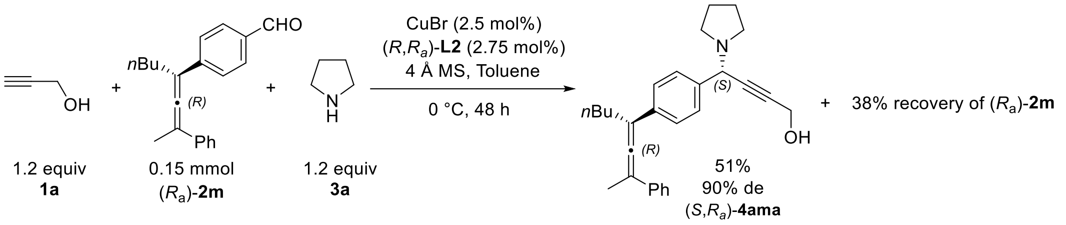

Following **Typical Procedure I**, the reaction of CuBr (0.5 mg, 3.42 μmol), (*R*,*R*a)-**L2** (2.4 mg, 4.08 μmol), 4 Å molecular sieves (45.3 mg)/toluene (0.75 mL), **1a** (10.3 mg, 0.18 mmol), (*R*a)-**2m** (43.7 mg, 0.15 mmol, 99% ee), and pyrrolidine **3a** (13.1 mg, 0.18 mmol) afforded (*S*,*R*a)-**4ama**. The recovery of (*R*a)-**2m** (38%) was determined by ^{1}H NMR analysis of the crude product using CH_2Br_2 as the internal standard. (*S*,*R*a)-**4ama** (30.7 mg, 51%) was obtained via column chromatography silica gel (eluent: petroleum ether/ethyl acetate = 4:1 (50 mL) to 1:1 (50 mL) 1:2 (45 mL)) as an oil: 90% de, (HPLC conditions: Chiralcel AD-H column, hexane/*i*-PrOH = 95/5, 1.0 mL/min, λ = 214 nm, t_R(major) = 14.3 min, t_R(minor) = 10.3 min; $[\alpha]_D^{26}$ = -317.3 (c = 1.03, $CHCl_3$); **^{1}H NMR** (400 MHz, $CDCl_3$) δ 7.47-7.41 (m, 4 H, ArH), 7.38 (d, J = 8.4 Hz, 2 H, ArH), 7.31 (t, J = 7.6 Hz, 2 H, ArH), 7.20 (t, J = 7.2 Hz, 1 H, ArH), 4.57 (s, 1 H, NCH), 4.33 (s, 2 H, OCH_2), 2.94-2.69 (br, 1 H, OH), 2.68-2.55 (m, 4 H, 2 × NCH_2), 2.53 (t, J = 7.4 Hz, 2 H, CH_2), 2.19 (s, 3 H, CH_3), 1.83-1.68 (m, 4 H, 2 × CH_2), 1.55 (quint, J = 7.5 Hz, 2 H, CH_2), 1.47-1.33 (m, 2 H, CH_2), 0.90 (t, J = 7.4 Hz, 3 H, CH_3); **^{13}C NMR** (100 MHz, $CDCl_3$) δ 205.6, 137.3, 137.1, 136.5, 128.4, 128.3, 126.6, 125.9, 125.6, 107.4, 103.6, 84.8, 82.9, 58.6, 51.1, 50.5, 30.0, 29.9, 23.2, 22.6, 16.8, 14.0; **MS** (ESI) *m/z* 400 ($[M+H]^+$); **IR** (neat): ν = 3344, 3083, 3058, 3026, 2958, 2925, 2858, 1931, 1597, 1508, 1492, 1460, 1443, 1415, 1357, 1295, 1261, 1093, 1065, 1019, 800, 731 cm^{-1}; **HRMS** calcd for $C_{28}H_{34}ON$ ($[M+H]^+$): 400.2635, Found: 400.2624.

14. (*R*)-4-(Pyrrolidin-1-yl)-4-(1-tosyl-indol-3-yl)but-2-yn-1-ol (*R*)-**4ana** (Lq-5-080)

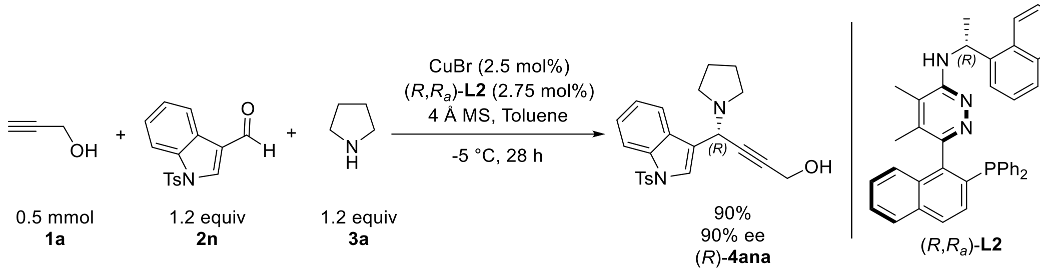

Following **Typical Procedure I**, the reaction of CuBr (1.8 mg, 0.0125 mmol), (*R*,*R*a)-**L2** (8.1 mg, 0.01375 mmol), 4 Å molecular sieves (151.4 mg)/toluene (0.75 mL), **1a** (28.4 mg, 0.5 mmol), **2n** (179.3 mg, 0.6 mmol)/toluene (0.5 mL), and pyrrolidine **3a** (43.0 mg, 0.6 mmol) afforded (*R*)-**4ana** (186.8 mg, 90%) (eluent: petroleum ether/ethyl acetate = 2:1 (900 mL) to 1:1 (400 mL), it should be noted that the column packed with silica gel was eluted with a mixture of petroleum ether (50 mL) and Et_3N (0.25 mL) before loading the sample) as a liquid: 90% ee (HPLC conditions: Chiralcel AD-H column, hexane/*i*-PrOH = 90/10, 1.0 mL/min, λ = 214 nm, t_R(major) = 19.1 min, t_R(minor) = 14.5 min); $[\alpha]_D^{29}$ = -25.4 (c = 1.015, $CHCl_3$); **^{1}H NMR** (400 MHz, $CDCl_3$) δ 7.94 (d, *J* = 8.0 Hz, 1 H, ArH), 7.79 (d, *J* = 7.6 Hz, 1 H, ArH), 7.74 (d, *J* = 8.4 Hz, 2 H, ArH), 7.66 (s, 1 H, ArH), 7.28 (t, *J* = 7.6 Hz, 1 H, ArH), 7.20 (t, *J* = 7.8 Hz, 1 H, ArH), 7.15 (d, *J* = 8.4 Hz, 2 H, ArH), 4.92 (s, 1 H, NCH),4.36 (d, *J* = 1.6 Hz, 2 H, OCH_2),2.91 (br, 1 H, OH), 2.68-2.49 (m, 4 H, 2 × CH_2), 2.27 (s, 3 H, CH_3), 1.81-1.62 (m, 4 H, 2 × CH_2); **^{13}C NMR** (100 MHz, $CDCl_3$) δ 144.8, 135.3, 135.0, 129.7, 129.4, 126.7, 124.74, 124.68, 123.1, 121.3, 120.9, 113.4, 84.3, 81.2, 50.8, 50.5, 49.7, 23.4, 21.4; **MS** (ESI) *m/z* 409 ($[M+H]^+$); **IR** (neat): ν = 3348, 2962, 1596, 1445, 1366, 1273, 1172, 1120, 1089, 1018 cm^{-1}; **HRMS** calcd for $C_{23}H_{25}N_2O_3S$ ($[M+H]^+$): 409.1580. Found: 409.1579.

15. (*R*)-4-(Benzo[b]thiophen-3-yl)-4-(pyrrolidin-1-yl)but-2-yn-1-ol (*R*)-**4aoa** (Lq-5-091)

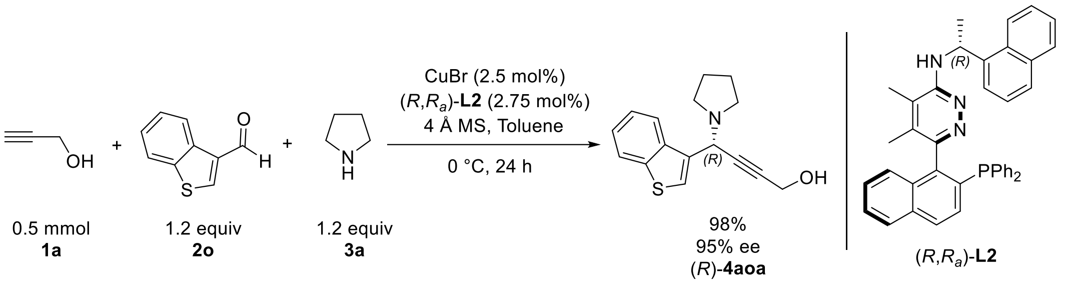

Following **Typical Procedure I**, the reaction of CuBr (1.8 mg, 0.0125 mmol), (R,R_a)-**L2** (8.1 mg, 0.01375 mmol), 4 Å molecular sieves (150.7 mg)/toluene (0.75 mL), **1a** (28.2 mg, 0.5 mmol), **2o** (106.7 mg, 0.6 mmol)/toluene (0.5 mL), and pyrrolidine **3a** (43.0 mg, 0.6 mmol) afforded (R)-**4aoa** (133.3 mg, 98%) (eluent: petroleum ether/ethyl acetate = 4:1 (250 mL) to 2:1 (600 mL)) as a liquid: 95% ee (HPLC conditions: Chiralcel AD-H column, hexane/i-PrOH = 95/5, 1.0 mL/min, λ = 214 nm, t_R(major) = 14.9 min, t_R(minor) = 12.3 min); $[\alpha]_D^{29}$ = -51.0 (c = 0.98, $CHCl_3$); **^{1}H NMR** (400 MHz, $CDCl_3$) δ 8.07 (dd, J_1 = 6.8 Hz, J_2 = 2.0 Hz, 1 H, ArH), 7.83 (dd, J_1 = 6.8 Hz, J_2 = 2.0 Hz, 1 H, ArH), 7.54 (s, 1 H, ArH), 7.40-7.30 (m, 2 H, ArH), 5.03 (s, 1 H, NCH), 4.36 (d, J = 2.0 Hz, 2 H, OCH_2), 2.74-2.54 (m, 4 H, 2 × CH_2), 2.14 (br, 1 H, OH), 1.83-1.67 (m, 4 H, 2 × CH_2); **^{13}C NMR** (100 MHz, $CDCl_3$) δ 140.6, 137.7, 134.2, 124.7, 124.3, 123.9, 122.9, 122.6, 84.4, 81.9, 53.2, 50.9, 50.2, 23.5; **MS** (ESI) m/z 272 ($[M+H]^+$); **IR** (neat): ν = 3289, 3058, 2962, 2928, 2872, 2808, 1458, 1426, 1344, 1292, 1245, 1113, 1070, 1018, 1008 cm^{-1}; **HRMS** calcd for $C_{16}H_{18}NOS$ ($[M+H]^+$): 272.1104, Found: 272.1105.

16. Gram-scale synthesis of (R)-4-(Benzo[b]thiophen-3-yl)-4-(pyrrolidin-1-yl)but-2-yn-1-ol (R)-**4aoa** (Lq-6-184)

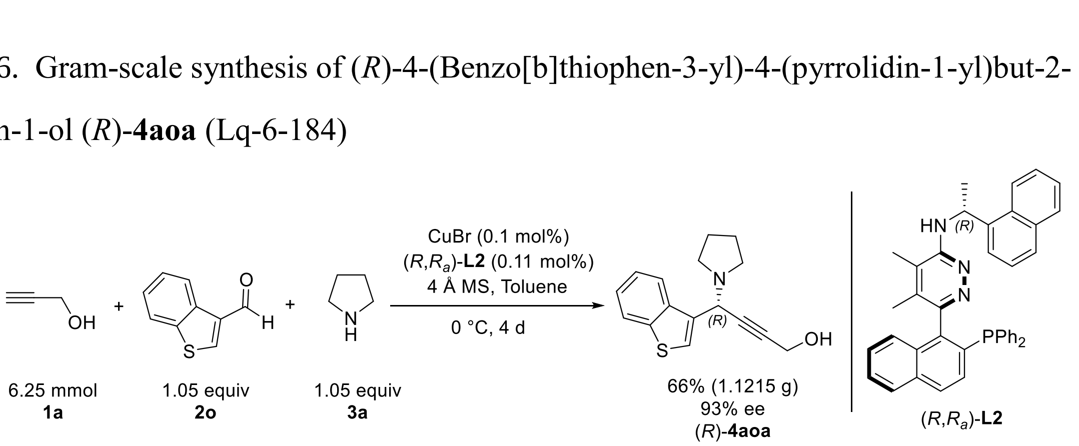

To a flame-dried Schlenk flask were added CuBr (0.9 mg, 6.25 μmol), (R,R_a)-**L2** (4.0 mg, 6.88 μmol), and toluene (1 mL) sequentially under Ar atmosphere. After being

stirred at room temperature for 30 min, 4 Å molecular sieves (1.8772 g), **1a** (351.2 mg, 6.25 mmol)/toluene (5 mL) and **2o** (1.1864 g, 6.56 mmol)/toluene (5 mL) were added sequentially under Ar atmosphere. The resulting mixture was stirred at 0 °C for another 10 min followed by the addition of pyrrolidine **3a** (467.3 mg, 6.56 mmol)/toluene (5 mL). After being stirred at 0 °C for 4 d, the reaction was complete as monitored by TLC. Then the resulting mixture was filtrated through a short pad of silica gel eluted with dichloromethane/MeOH (10:1, 110 mL). After evaporation, the residue was purified by chromatography on silica gel (eluent: petroleum ether/ethyl acetate = 5:1 (480 mL) to 1:1 (500 mL)) to afford (*R*)-**4aoa** (1.1215 g, 66%) as a liquid: 93% ee (HPLC conditions: Chiralcel AD-H column, hexane/*i*-PrOH = 95/5, 1.0 mL/min, λ = 214 nm, t_R(major) = 13.5 min, t_R(minor) = 11.5 min); **^{1}H NMR** (400 MHz, $CDCl_3$) δ 8.05 (dd, J_1 = 7.0 Hz, J_2 = 1.8 Hz, 1 H, ArH), 7.81 (dd, J_1 = 6.8 Hz, J_2 = 1.2 Hz, 1 H, ArH), 7.54 (s, 1 H, ArH), 7.40-7.28 (m, 2 H, ArH), 4.99 (s, 1 H, NCH), 4.32 (d, J = 2.0 Hz, 2 H, OCH_2), 2.78 (br, 1 H, OH), 2.70-2.52 (m, 4 H, 2 × CH_2), 1.84-1.63 (m, 4 H, 2 × CH_2); **^{13}C NMR** (100 MHz, $CDCl_3$) δ 140.6, 137.6, 134.1, 124.7, 124.3, 123.9, 122.8, 122.6, 84.4, 81.9, 53.2, 50.8, 50.2, 23.5.

17. (*R*)-4-(Pyrrolidin-1-yl)-4-(thien-2-yl)-2-butyn-1-ol (*R*)-**4apa** (Lq-5-109)

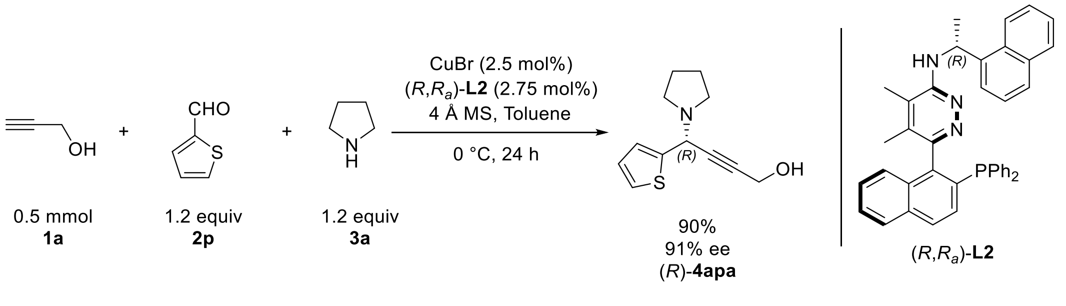

Following **Typical Procedure I**, the reaction of CuBr (1.8 mg, 0.0125 mmol), (*R*,*R*a)-**L2** (8.1 mg, 0.01375 mmol), 4 Å molecular sieves (151.2 mg)/toluene (0.75 mL), **1a** (28.2 mg, 0.5 mmol), **2p** (69.1 mg, 0.6 mmol)/toluene (0.5 mL), and pyrrolidine **3a** (42.9 mg, 0.6 mmol) afforded (*R*)-**4apa** (100.5 mg, 90%) (eluent: petroleum ether/ethyl acetate = 2:1 (900 mL)) as a liquid: 91% ee (HPLC conditions: Chiralcel AD-Hcolumn, hexane/*i*-PrOH = 95/5, 1.0 mL/min, λ = 214 nm, t_R(major) = 11.3 min, t_R(minor) = 9.7 min); $[\alpha]_D^{24}$ = -55.5 (c = 1.045, $CHCl_3$); **^{1}H NMR** (400 MHz, $CDCl_3$) δ 7.24 (d, J =

4.8 Hz, 1 H, ArH), 7.12 (d, J = 3.2 Hz, 1 H, ArH), 6.93 (dd, J_1 = 4.8 Hz, J_2 = 3.6 Hz, 1 H, ArH), 4.94 (s, 1 H, NCH), 4.36 (s, 2 H, OCH_2), 3.17-2.79 (br, 1 H, OH), 2.76-2.57 (m, 4 H, 2 × CH_2), 1.86-1.72 (m, 4 H, 2 × CH_2); **^{13}C NMR** (100 MHz, $CDCl_3$) δ 143.3, 126.2, 125.6, 125.3, 84.6, 81.8, 53.8, 50.8, 49.9, 23.4; **MS** (ESI) *m/z* 222 ($[M+H]^+$); **IR** (neat): ν = 3066, 2962, 2914, 2878, 2842, 2816, 2725, 2628, 1479, 1456, 1446, 1433, 1377, 1347, 1320, 1310, 1295, 1237, 1206, 1185, 1120, 1106, 1088, 1075, 1035, 1020 cm^{-1}; **HRMS** calcd for $C_{12}H_{16}NOS$ ($[M+H]^+$): 222.0947, Found: 222.0951.

18. (*S*)-4-Cyclohexyl-4-(1-pyrrolidinyl)-2-butyn-1-ol (*S*)-**4aqa** (Lq-5-028)

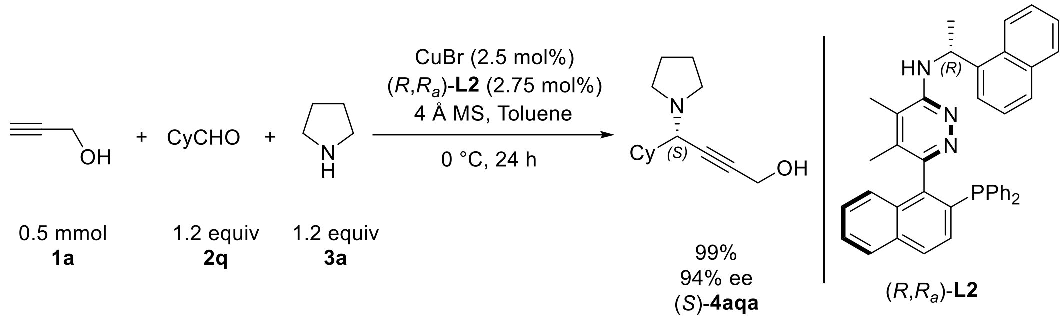

Following **Typical Procedure I**, the reaction of CuBr (1.8 mg, 0.0125 mmol), (*R*,*R*a)-**L2** (8.1 mg, 0.01375 mmol), 4 Å molecular sieves (150.3 mg)/toluene (0.75 mL), **1a** (28.3 mg, 0.5 mmol), **2q** (68.1 mg, 0.6 mmol)/toluene (0.5 mL), and pyrrolidine **3a** (43.6 mg, 0.6 mmol) afforded (*S*)-**4aqa**[3] (110.6 mg, 99%) (eluent: petroleum ether/ethyl acetate = 2:1 (210 mL) to 1:1 (600 mL)) as a liquid: 94% ee (HPLC conditions: Chiralcel AD-H column, hexane/*i*-PrOH = 90/10, 0.7 mL/min, λ = 214 nm, t_R(major) = 6.6 min, t_R(minor) = 5.9 min); $[\alpha]_D^{28}$ = -18.4 (c = 0.955, $CHCl_3$)(reported value: 98% ee; $[\alpha]_D^{22}$ = -19.7 (c = 1.03, $CHCl_3$)[3]); **^{1}H NMR** (400 MHz, $CDCl_3$) δ 4.30 (d, J = 1.6 Hz, 2 H, OCH_2), 3.15 (d, J = 8.0 Hz, 1 H, NCH), 2.93 (br, 1 H, OH), 2.73-2.61 (m, 2 H, two protons from 2 × NCH_2), 2.61-2.50 (m, 2 H, two protons from 2 × NCH_2), 1.98 (d, J = 12.4 Hz, 1 H, one proton from CH_2), 1.84 (d, J = 12.8 Hz, 1 H, one proton from CH_2), 1.81-1.61 (m, 7 H, protons from Cy and pyrrolidine), 1.56-1.43 (m, 1 H, one proton from Cy and pyrrolidine), 1.31-0.98 (m, 5 H, protons from Cy and pyrrolidine); **^{13}C NMR** (100 MHz, $CDCl_3$) δ 84.0, 83.2, 60.9, 50.9, 50.1, 40.9, 30.5, 29.7, 26.5, 26.1, 26.0, 23.2; **MS** (ESI) *m/z* 222 ($[M+H]^+$); **IR** (neat): ν = 3372, 2921,

2850, 2709, 2669, 1448, 1348, 1337, 1310, 1277, 1260, 1219, 1146, 1113, 1069, 1014 cm^{-1}.

19. Gram-scale synthesis of (*S*)-4-cyclohexyl-4-(1-pyrrolidinyl)-2-butyn-1-ol (*S*)-**4aqa** (Lq-5-119)

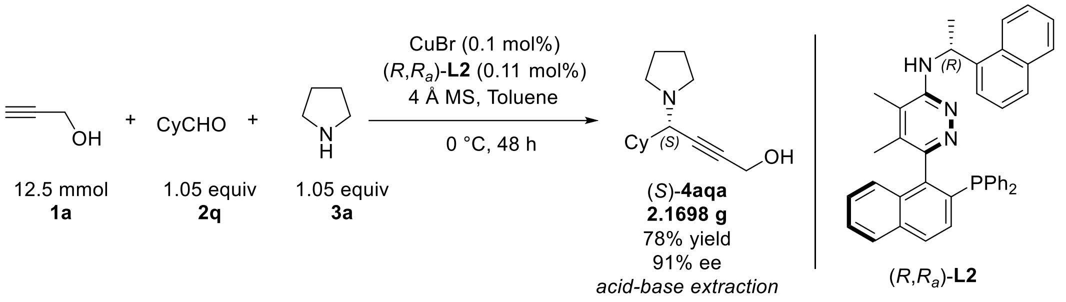

To a flame-dried Schlenk flask were added CuBr (1.8 mg, 0.0125 mmol), (*R*,*R*a)-**L2** (8.1 mg, 0.01375 mmol), 4 Å molecular sieves (1.8915 g), and toluene (10 mL) sequentially under Ar atmosphere. After being stirred at room temperature for 1 h, **1a** (0.7018 g, 12.5 mmol)/toluene (7 mL) and **2q** (1.4829 g, 13.1 mmol)/toluene (7 mL) were added sequentially under Ar atmosphere. The resulting mixture was stirred at 0 °C for another 10 min followed by the addition of pyrrolidine **3a** (0.9371 g, 13.1 mmol)/toluene (7 mL). After being stirred at 0 °C for 48 h, the reaction was complete as monitored by TLC. The resulting mixture was filtrated through a short pad of basic aluminum oxide (200-300 mesh) eluted with dichloromethane/MeOH (10:1, 110 mL). After evaporation, the residue was dissolved in dichloromethane (30 mL) and washed with brine (30 mL). The organic layer was extracted with an aqueous solution of hydrochloric acid (1 M, 3 × 25 mL). The combined aqueous layer was basified using aqueous $NH_3•H_2O$ (12 M) and extracted with ether (3× 50 mL). The combined organic layer was dried over anhydrous Na_2SO_4. After filtration and evaporation, the residue was pure (*S*)-**4aqa**[3] (2.1698 g, 78%) as a liquid: 91% ee (HPLC conditions: Chiralcel AD-H column, hexane/*i*-PrOH = 95/5, 0.8 mL/min, λ = 214 nm, t_R(major) = 8.8 min, t_R(minor) = 7.5 min); **^{1}H NMR** (400 MHz, $CDCl_3$) δ 4.31 (d, *J* = 1.6 Hz, 2 H, OCH_2), 3.15 (d, *J* = 8.0 Hz, 1 H, NCH), 2.74-2.60 (m, 2 H, two protons from 2 × NCH_2), 2.59-2.47 (m, 2 H, two protons from 2 × NCH_2), 2.06-1.60 (m, 10 H), 1.56-1.40 (m, 1 H,

one proton from Cy and pyrrolidine), 1.31-0.96 (m, 5 H, protons from Cy and pyrrolidine); **^{13}C NMR** (100 MHz, $CDCl_3$) δ 84.1, 83.1, 60.9, 50.8, 50.1, 41.0, 30.5, 29.7, 26.5, 26.1, 26.0, 23.2.

20. (*R*)-2-(5-Hydroxy-2-(1-pyrrolidinyl)-3-pentyn-1-yl)isoindoline-1,3-dione (*R*)-**4ara** (Lq-5-108)

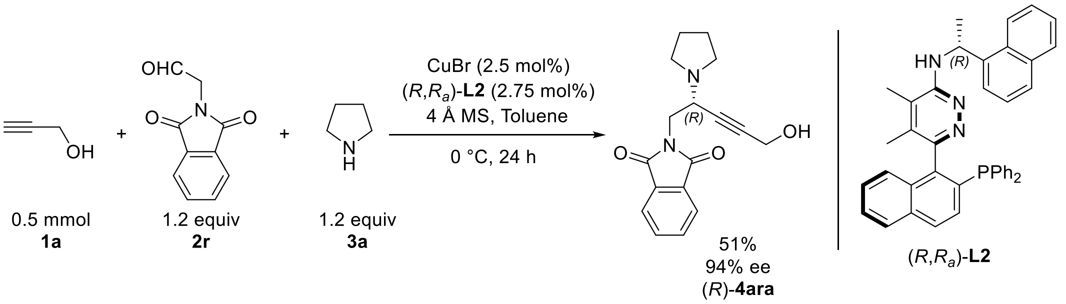

Following **Typical Procedure I**, the reaction of CuBr (1.8 mg, 0.0125 mmol), (*R*,*R*a)-**L2** (8.1 mg, 0.01375 mmol), 4 Å molecular sieves (150.5 mg)/toluene (0.75 mL), **1a** (28.1 mg, 0.5 mmol), **2r** (115.6 mg, 0.6 mmol)/toluene (0.5 mL), and pyrrolidine **3a** (42.8 mg, 0.6 mmol) afforded (*R*)-**4ara** (75.9 mg, 51%) (eluent: petroleum ether/ethyl acetate = 2:1 (150 mL) to 1:1 (800 mL) as a liquid. It should be noted that the column packed with silica gel was eluted with a mixture of petroleum ether (50 mL) and Et_3N (0.25 mL) before loading the sample): 94% ee (HPLC conditions: PC-2 column, hexane/*i*-PrOH = 70/30, 1.0 mL/min, λ = 214 nm, t_R(major) = 10.6 min, t_R(minor) = 17.2 min); $[\alpha]_D^{24}$ = -40.1 (c = 1.01, $CHCl_3$); **^{1}H NMR** (400 MHz, $CDCl_3$) δ 7.85 (dd, J_1 = 5.2 Hz, J_2 = 3.2 Hz, 2 H, ArH), 7.72 (dd, J_1 = 5.2 Hz, J_2 = 3.2 Hz, 2 H, ArH), 4.22 (s, 2 H, OCH_2), 4.14 (t, J = 7.8 Hz, 1 H, NCH), 3.96-3.82 (m, 2 H, CH_2), 2.83 (br, 1 H, OH), 2.81-2.71 (m, 2 H, CH_2), 2.70-2.56 (m, 2 H, CH_2), 1.81-1.66 (m, 4 H, 2 × CH_2); **^{13}C NMR** (100 MHz, $CDCl_3$) δ 168.2, 133.9, 131.9, 123.2, 85.2, 80.5, 52.2, 50.7, 49.2, 40.9, 23.4; **MS** (ESI) *m/z* 299 ($[M+H]^+$); **IR** (neat): ν = 3129, 2973, 2944, 2877, 2829, 2696, 1774, 1710, 1611, 1483, 1465, 1453, 1434, 1392, 1359, 1335, 1244, 1189, 1133, 1115, 1083, 1034, 1007 cm^{-1}; **HRMS** calcd for $C_{17}H_{19}N_2O_3$ ($[M+H]^+$): 299.1390, Found: 299.1391.

21. (*S*)-5-(4-Bromophenyl)-5-(1-pyrrolidinyl)-3-pentyn-1-ol (*S*)-**4bsa** (Lq-5-049)

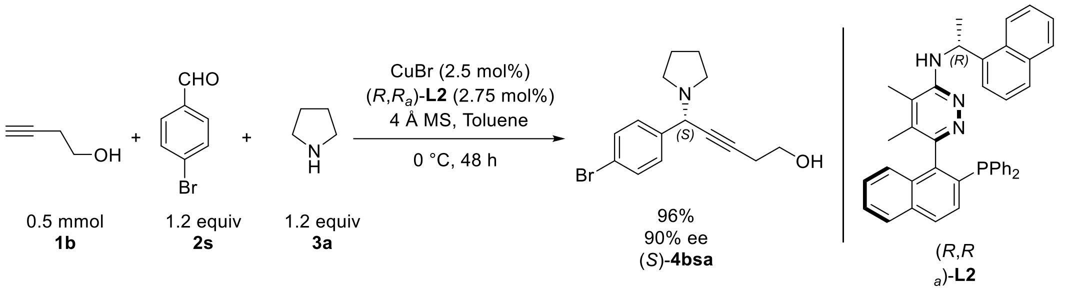

Following **Typical Procedure I**, the reaction of CuBr (1.8 mg, 0.0125 mmol), (*R*,*R*a)-**L2** (8.1 mg, 0.01375 mmol), 4 Å molecular sieves (150.7 mg)/toluene (0.75 mL), **1b** (36.5 mg, 0.5 mmol), **2s** (113.3 mg, 0.6 mmol)/toluene (0.5 mL), and pyrrolidine **3a** (43.0 mg, 0.6 mmol) afforded (*S*)-**4bsa** (148.8 mg, 96%) (eluent: petroleum ether/ethyl acetate = 2:1 (900 mL), it should be noted that the column packed with silica gel was eluted with a mixture of petroleum ether (50 mL) and Et_3N (0.25 mL) before loading the sample) as a liquid: 90% ee (HPLC conditions: Chiralcel AD-H column, hexane/*i*-PrOH = 98/2, 1.0 mL/min, λ = 214 nm, t_R(major) = 28.8 min, t_R(minor) = 33.6 min); $[\alpha]_D^{27}$ = -23.1 (c = 1.01, $CHCl_3$); **^{1}H NMR** (400 MHz, $CDCl_3$) δ 7.45 (d, *J* = 8.4 Hz, 2 H, ArH), 7.39 (d, *J* = 8.4 Hz, 2 H, ArH), 4.49 (s, 1 H, NCH), 3.73 (t, *J* = 6.4 Hz, 2 H, CH_2), 2.87 (br, 1 H, OH), 2.64-2.47 (m, 6 H, 3 × CH_2), 1.84-1.68 (m, 4 H, 2 × CH_2); **^{13}C NMR** (100 MHz, $CDCl_3$) δ 138.5, 131.3, 129.9, 121.4, 83.9, 78.6, 61.0, 58.4, 50.4, 23.2, 23.1; **MS** (ESI) *m/z* 310 ([M(^{81}Br)+H]$^+$), 308 ([M(^{79}Br)+H]$^+$); **IR** (neat): ν = 3322, 2960, 2875, 2810, 2242, 1590, 1574, 1484, 1460, 1402, 1373, 1344, 1303, 1287, 1262, 1197, 1132, 1120, 1096, 1069, 1045, 1010 cm^{-1}; **HRMS** calcd for $C_{15}H_{19}{}^{79}BrNO$ ([M+H]$^+$): 308.0645, Found: 308.0648.

22. (*S*)-1-(1-Phenyl-2-undecyn-1-yl)pyrrolidine (*S*)-**4caa** (Lq-5-097)

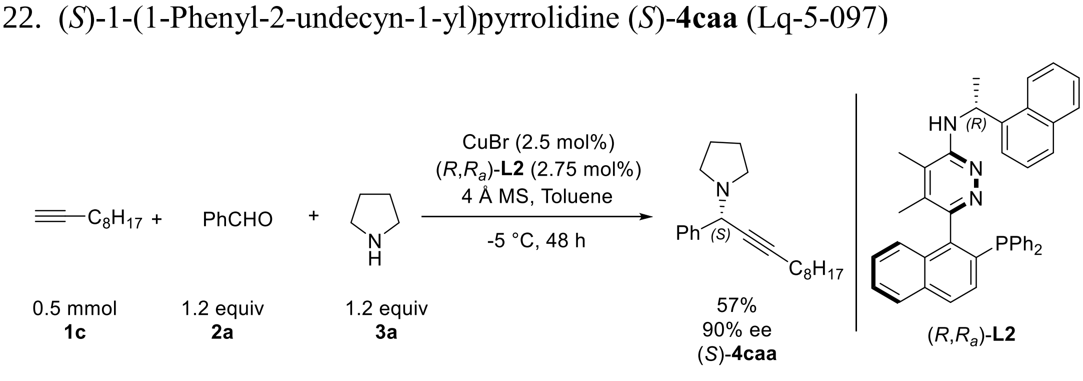

Following **Typical Procedure I**, the reaction of CuBr (1.8 mg, 0.0125 mmol), (R,R_a)-**L2** (8.1 mg, 0.01375 mmol), 4 Å molecular sieves (150.5 mg)/toluene (0.75 mL), **1c** (73.1 mg, 0.5 mmol), **2a** (64.2 mg, 0.6 mmol)/toluene (0.5 mL), and pyrrolidine **3a** (42.9 mg, 0.6 mmol) afforded (*S*)-**4caa**[4] (85.6 mg, 57%) (eluent: petroleum ether/ethyl acetate = 20:1 (420 mL) to 10:1 (330 mL)) as a liquid: 90% ee (HPLC conditions: Chiralcel OJ-H column, hexane/*i*-PrOH = 100/0, 0.5 mL/min, λ = 214 nm, t_R(major) = 10.0 min, t_R(minor) = 8.1 min); $[\alpha]_D^{28}$ = -20.6 (c = 1.02, $CHCl_3$) (reported value: 92% ee, $[\alpha]_D^{20}$ = -25.9 (c = 0.5, $CHCl_3$)[4]); **^{1}H NMR** (400 MHz, $CDCl_3$) δ 7.53 (d, J = 7.2 Hz, 2 H, ArH), 7.32 (t, J = 7.4 Hz, 2 H, ArH), 7.25 (t, J = 7.2 Hz, 1 H, ArH), 4.62 (s, 1 H, NCH), 2.68-2.50 (m, 4 H, 2 × CH_2), 2.27 (td, J_1 = 7.0 Hz, J_2 = 1.6 Hz, 2 H, CH_2), 1.85-1.69 (m, 4 H, 2 × CH_2), 1.55 (quint, J = 7.2 Hz, 2 H, CH_2), 1.49-1.37 (m, 2 H, CH_2), 1.36-1.18 (m, 8 H, 4× CH_2), 0.88 (t, J = 6.4 Hz, 3 H, CH_3); **^{13}C NMR** (100 MHz, $CDCl_3$) δ 140.0, 128.2, 128.0, 127.3, 87.1, 76.8, 58.7, 50.1, 31.8, 29.2, 29.0, 28.9, 28.8, 23.4, 22.6, 18.7, 14.1; **MS** (ESI) *m/z* 298 ($[M+H]^+$); **IR** (neat): ν = 3029, 2956, 2925, 2854, 2808, 1602, 1492, 1450, 1378, 1345, 1326, 1301, 1267, 1198, 1134, 1122, 1098, 1071, 1029 cm^{-1}.

23. (*S*)-1-(1,3-Diphenyl-2-propyn-1-yl)pyrrolidine (*S*)-**4daa** (Lq-5-062)

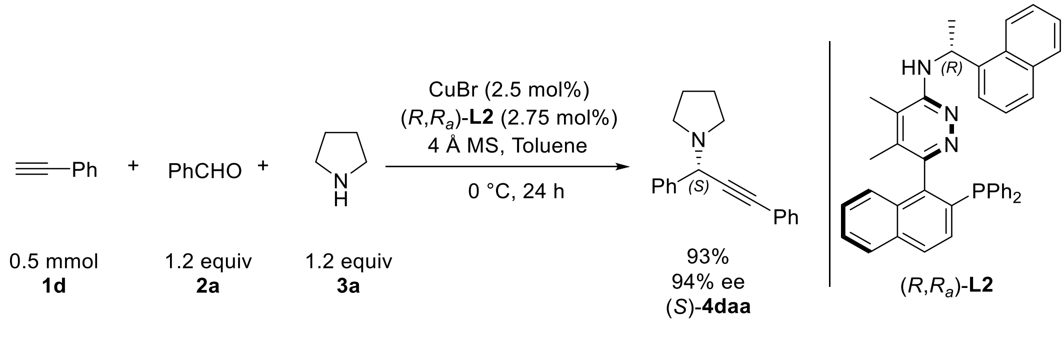

Following **Typical Procedure I**, the reaction of CuBr (1.8 mg, 0.0125 mmol), (R,R_a)-**L2** (8.1 mg, 0.01375 mmol), 4 Å molecular sieves (150.0 mg)/toluene (0.75 mL), **1d** (51.4 mg, 0.5 mmol), **2a** (63.8 mg, 0.6 mmol)/toluene (0.5 mL), and pyrrolidine **3a** (43.2 mg, 0.6 mmol) afforded (*S*)-**4daa**[4] (118.8 mg, 93%) (eluent: petroleum ether/ethyl acetate = 20:1 (210 mL) to 10:1 (440 mL)) as a liquid: 94% ee (HPLC conditions: Chiralcel OJ-H column, hexane/*i*-PrOH = 99/1, 1.0 mL/min, λ = 214 nm,

t_R(major) = 8.0 min, t_R(minor) = 6.5 min); $[\alpha]_D^{32}$ = -14.9 (c = 1.07, $CHCl_3$)(reported value: 92% ee, $[\alpha]_D^{20}$ = -8.2 (c = 0.5, $CHCl_3$)[4]); **^{1}H NMR** (400 MHz, $CDCl_3$) δ 7.61 (d, *J* = 8.0 Hz, 2 H, ArH), 7.53-7.45 (m, 2 H, ArH), 7.40-7.25 (m, 6 H, ArH), 4.88 (s, 1 H, NCH), 2.75-2.60 (m, 4 H, 2 × NCH_2), 1.88-1.71 (m, 4 H, 2 × CH_2); **^{13}C NMR** (100 MHz, $CDCl_3$) δ 139.5, 131.7, 128.2, 128.0, 127.5, 123.2, 86.8, 86.7, 59.1, 50.2, 23.4; **MS** (ESI) *m/z* 262 ($[M+H]^+$); **IR** (neat): ν = 3059, 3030, 2963, 2874, 2805, 1598, 1489, 1449, 1344, 1321, 1302, 1269, 1195, 1113, 1070, 1027, 1003 cm^{-1}.

24. (*S*)-2-methyl-5-phenyl-5-(1-pyrrolidinyl)pent-3-yn-2-ol (*S*)-**4eaa** (Lq-7-028)

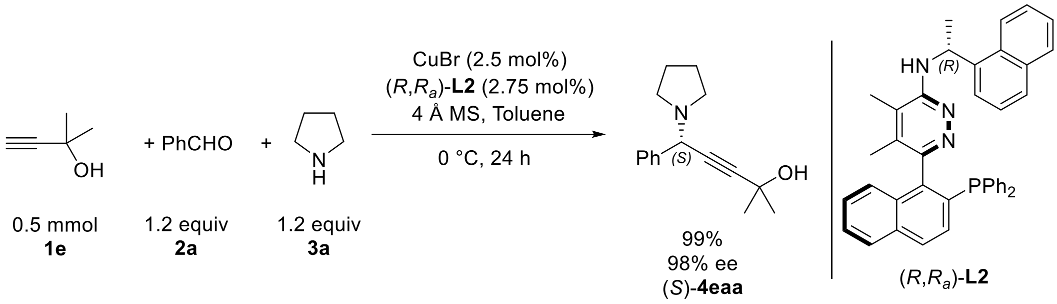

Following **Typical Procedure I**, the reaction of CuBr (1.8 mg, 0.0125 mmol), (*R*,*R*$_a$)-**L2** (8.1 mg, 0.01375 mmol), 4 Å molecular sieves (150.7 mg)/toluene (0.75 mL), **1e** (43.1 mg, 0.5 mmol), **2a** (63.9 mg, 0.6 mmol)/toluene (0.5 mL), and pyrrolidine **3a** (43.0 mg, 0.6 mmol) afforded (*S*)-**4eaa**[3] (121.0 mg, 99%) (eluent: petroleum ether/ethyl acetate = 5:1 (24 mL) to 1:1 (60 mL)) as a liquid: 98% ee (HPLC conditions: Chiralcel OD-H column, hexane/*i*-PrOH = 99/1, 1.0 mL/min, λ = 214 nm, t_R(major) = 14.6 min, t_R(minor) = 17.8 min; $[\alpha]_D^{26}$ = -38.8 (c = 1.02, $CHCl_3$) (reported value: 98% ee, $[\alpha]_D^{20}$ = -39.0 (c = 1.00, $CHCl_3$)[3]); **^{1}H NMR** (400 MHz, $CDCl_3$) δ 7.50 (d, *J* = 7.2 Hz, 2 H, ArH), 7.32 (t, *J* = 7.2 Hz, 2 H, ArH), 7.26 (t, *J* = 6.8 Hz, 1 H, ArH), 4.62 (s, 1 H, NCH), 2.67-2.49 (m, 5 H, 2 × NCH_2 + OH), 1.84-1.68 (m, 4 H, 2 × CH_2), 1.56 (s, 6 H, 2 × CH_3); **^{13}C NMR** (100 MHz, $CDCl_3$) δ 139.2, 128.2, 128.1, 127.5, 91.6, 79.0, 65.1, 58.4, 50.1, 31.7, 23.3; **MS** (ESI) *m/z* 244 ($[M+H]^+$); **IR** (neat): ν = 3364, 3061, 3030, 2972, 2932, 2875, 2807, 1602, 1491, 1451, 1360, 1345, 1301, 1265, 1228, 1166, 1131, 1076, 1024 cm^{-1}.

25. (*S*)-5-Cyclohexyl-2-methyl-5-(1-pyrrolidinyl)-3-pentyn-2-ol (*S*)-**4eqa** (Lq-5-059)

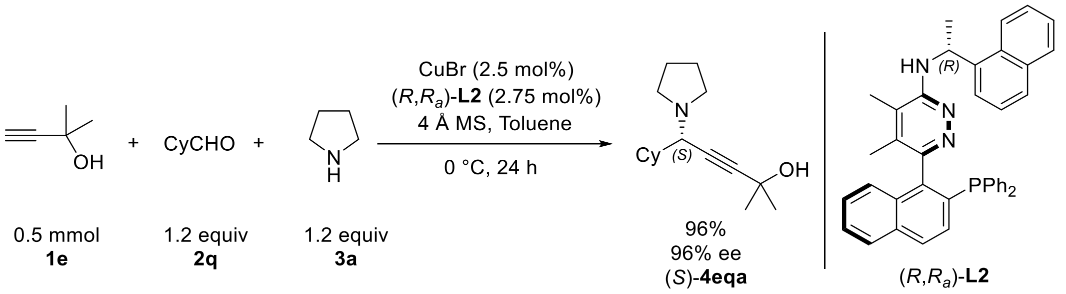

Following **Typical Procedure I**, the reaction of CuBr (1.8 mg, 0.0125 mmol), (*R*,*R*a)-**L2** (8.1 mg, 0.01375 mmol), 4 Å molecular sieves (150.5 mg)/toluene (0.75 mL), **1e** (43.2 mg, 0.5 mmol), **2q** (67.5 mg, 0.6 mmol)/toluene (0.5 mL), and pyrrolidine **3a** (43.2 mg, 0.6 mmol) afforded (*S*)-**4eqa**[2] (120.6 mg, 96%) (eluent: petroleum ether/ethyl acetate = 5:1 (240 mL) to 2:1 (600 mL), it should be noted that the column packed with silica gel was eluted with a mixture of petroleum ether (50 mL) and Et_3N (0.25 mL) before loading the sample) as a liquid: 96% ee (HPLC conditions: Chiralcel OD-H column, hexane/*i*-PrOH = 200/1, 1.0 mL/min, λ = 214 nm, t_R(major) = 15.1 min, t_R(minor) = 13.2 min); $[\alpha]_D^{28}$ = -16.9 (c = 1.0, $CHCl_3$)(reported value: 98% ee; $[\alpha]_D^{20}$ = -17.6 (c = 1.01, $CHCl_3$)[2]); **^{1}H NMR** (400 MHz, $CDCl_3$) δ 3.10 (d, *J* = 8.0 Hz, 1 H, NCH), 2.69-2.60 (m, 2 H, two protons from 2 × NCH_2), 2.60-2.49 (m, 2 H, two protons from 2 × NCH_2), 2.29 (br, 1 H, OH), 2.02-1.92 (m, 1 H), 1.91-1.82 (m, 1 H), 1.81-1.62 (m, 7 H), 1.52 (s, 6 H, 2 × CH_3), 1.51-1.41 (m, 1 H), 1.29-0.94(m, 5 H); **^{13}C NMR** (100 MHz, $CDCl_3$) δ 90.5, 79.8, 65.2, 60.6, 50.0, 41.0, 31.8, 30.6, 29.8, 26.6, 26.13, 26.08, 23.4; **MS** (ESI) *m/z* 250[M+H]$^+$); **IR** (neat): ν = 3390, 2977, 2950, 2925, 2868, 2852, 2819, 1449, 1360, 1314, 1260, 1220, 1173, 1164, 1115, 1021 cm^{-1}.

26. (*S*)-1-(3-(4-Methoxyphenyl)-1-phenylprop-2-yn-1-yl)piperidine (*S*)-**4fab** (Lq-5-070)

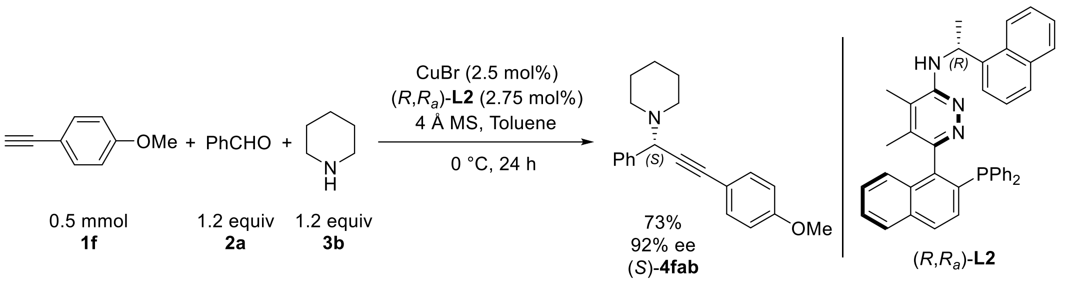

Following **Typical Procedure I**, the reaction of CuBr (1.8 mg, 0.0125 mmol), (*R*,*R*a)-**L2** (8.1 mg, 0.01375 mmol), 4 Å molecular sieves (150.0 mg)/toluene (0.75 mL), **1f** (67.6 mg, 0.5 mmol), **2a** (64.0 mg, 0.6 mmol)/toluene (0.5 mL), and piperidine **3b** (51.7 mg, 0.6 mmol) afforded (*S*)-**4fab** (111.5 mg, 73%) (eluent: petroleum ether/ethyl ether = 50:1 (510 mL) to 20:1 (420 mL)) as a liquid: 92% ee, (HPLC conditions: Chiralcel OJ-H column, hexane/*i*-PrOH = 99/1, 0.5 mL/min, λ = 214 nm, t_R(major) = 23.8 min, t_R(minor) = 19.5 min; $[\alpha]_D^{29}$ = -4.1 (c = 1.02, $CHCl_3$); **^{1}H NMR** (400 MHz, $CDCl_3$) δ 7.62 (d, *J* = 7.6 Hz, 2 H, ArH), 7.45 (d, *J* = 8.8 Hz, 2 H, ArH), 7.35 (t, *J* = 7.4 Hz, 2 H, ArH), 7.27 (t, *J* = 7.2 Hz, 1 H, ArH), 6.85 (d, *J* = 8.8 Hz, 2 H, ArH), 4.77 (s, 1 H, NCH), 3.80 (s, 3 H, CH_3), 2.64-2.44 (m, 4 H, 2 × NCH_2), 1.67-1.50 (m, 4 H, 2 × CH_2), 1.49-1.36 (m, 2 H, CH_2); **^{13}C NMR** (100 MHz, $CDCl_3$) δ 159.4, 138.7, 133.1, 128.5, 128.0, 127.3, 115.4, 113.8, 87.5, 84.5, 62.4, 55.2, 50.6, 26.1, 24.4; **MS** (ESI) *m/z* 306 ($[M+H]^+$); **IR** (neat): ν = 2931, 2851, 2835, 2803, 2748, 1605, 1569, 1508, 1465, 1450, 1318, 1289, 1272, 1244, 1203, 1171, 1153, 1105, 1093, 1071, 1031 cm^{-1}; **HRMS** calcd for $C_{21}H_{24}NO$ ($[M+H]^+$): 306.1852, Found: 306.1850.

27. (*S*)-1-(1,3-diphenylprop-2-yn-1-yl)piperidine (*S*)-**4dab** (Lq-7-006)

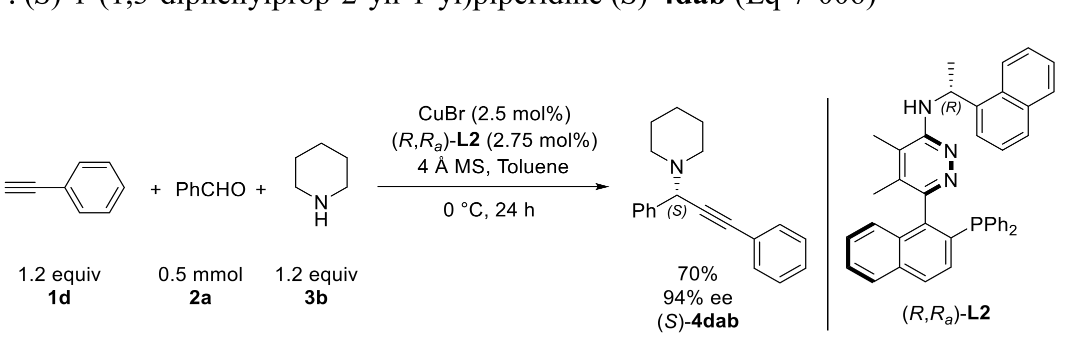

Following **Typical Procedure I**, the reaction of CuBr (1.8 mg, 0.0125 mmol), (*R*,*R*a)-**L2** (8.1 mg, 0.01375 mmol), 4 Å molecular sieves (151.2 mg)/toluene (0.75 mL), **1d** (61.5 mg, 0.6 mmol), **2a** (53.1 mg, 0.5 mmol)/toluene (0.5 mL), and **3b** (51.9 mg,

0.6 mmol) afforded (*S*)-**4dab** (96.3 mg, 70%) (eluent: petroleum ether/ethyl ether = 100:1 (50 mL) to 50:1 (50 mL)) as a white solid: 94% ee, (HPLC conditions: Chiralcel OJ-H column, hexane/*i*-PrOH = 99/1, 0.5 mL/min, λ = 214 nm, t_R(major) = 10.7 min, t_R(minor) = 9.7 min; $[\alpha]_D^{27}$ = -10.4 (c = 1.015, $CHCl_3$) (reported value: 68% ee, $[\alpha]_D^{20}$ = -7.8 (c = 0.5, $CHCl_3$)[4]); m.p. 69.9-72.9 °C (hexane, 94% ee) (reported value: 68% ee, m.p. 63-65 °C[4]); **^{1}H NMR** (400 MHz, $CDCl_3$) δ 7.64 (d, *J* = 7.2 Hz, 2 H, ArH), 7.56-7.46 (m, 2 H, ArH), 7.40-7.25 (m, 6 H, ArH), 4.79 (s, 1 H, NCH), 2.67-2.41 (m, 4 H, 2 × NCH_2), 1.68-1.51 (m, 4 H), 1.50-1.35 (m, 2 H); **^{13}C NMR** (100 MHz, $CDCl_3$) δ 138.6, 131.8, 128.5, 128.2, 128.0, 127.4, 123.3, 87.8, 86.1, 62.4, 50.7, 26.2, 24.4; **MS** (ESI) *m/z* 276 ($[M+H]^+$); **IR** (neat): ν = 2935, 2856, 2800, 2741, 1598, 1489, 1466, 1448, 1324, 1314, 1296, 1269, 1200, 1183, 1154, 1112, 1090, 1074, 1065, 1037, 1026 cm^{-1}.

28. (*S*)-4-Cyclohexyl-4-(1-piperidinyl)-2-butyn-1-ol (*S*)-**4aqb** (Lq-5-095)

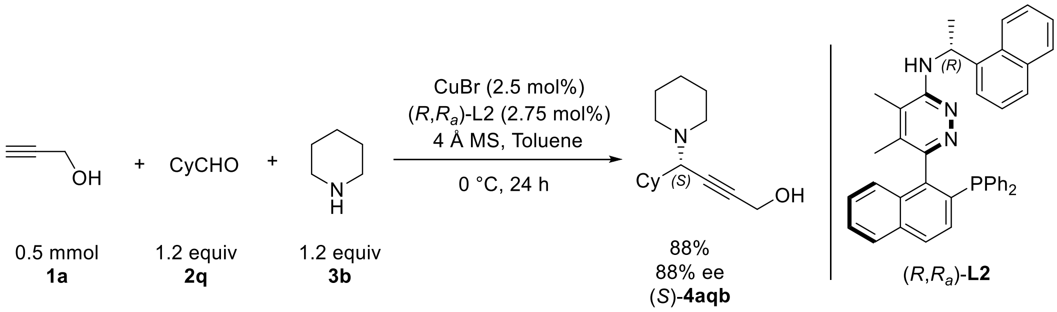

Following **Typical Procedure I**, the reaction of CuBr (1.8 mg, 0.0125 mmol), (*R*,*R*a)-**L2** (8.1 mg, 0.01375 mmol), 4 Å molecular sieves (150.4 mg)/toluene (0.75 mL), **1a** (28.2 mg, 0.5 mmol), **2q** (68.1 mg, 0.6 mmol)/toluene (0.5 mL), and **3b** (51.7 mg, 0.6 mmol) afforded (*S*)-**4aqb** (103.8 mg, 88%) (eluent: petroleum ether/ethyl acetate = 4:1 (250 mL) to 2:1 (450 mL)) as a liquid: 88% ee (HPLC conditions: Chiralcel AD-H column, hexane/*i*-PrOH = 99/1, 1.0 mL/min, λ = 214 nm, t_R(major) = 16.0 min, t_R(minor) = 13.5 min); $[\alpha]_D^{28}$ = -17.4 (c = 1.07, $CHCl_3$); **^{1}H NMR** (400 MHz, $CDCl_3$) δ 4.33 (d, *J* = 2.0 Hz, 2 H, OCH_2), 2.92 (d, *J* = 9.6 Hz, 1 H, NCH), 2.62-2.48 (m, 2 H), 2.39-2.23 (m, 3 H), 2.07-1.89 (m, 2 H), 1.80-1.36 (m, 10 H), 1.31-1.09 (m, 3 H), 1.04-0.82 (m, 2 H); **^{13}C NMR** (100 MHz, $CDCl_3$) δ 84.1, 83.3, 63.8, 51.1, 50.5, 39.2, 31.0 30.3, 26.6,

26.12, 26.05, 26.01, 24.5; **MS** (ESI) *m/z* 236 ([M+H]$^+$); **IR** (neat): ν = 3314, 2921, 2850, 2803, 2751, 1448, 1385, 1359, 1317, 1301, 1261, 1230, 1182, 1157, 1101, 1078, 1053, 1013 cm^{-1}; **HRMS** calcd for $C_{15}H_{26}NO$ ([M+H]$^+$): 236.2009, Found: 236.2008.

29. (*S*)-1-(3-(3-chlorophenyl)-1-phenylprop-2-yn-1-yl)azepane (*S*)-**4gac** (Lq-6-194)

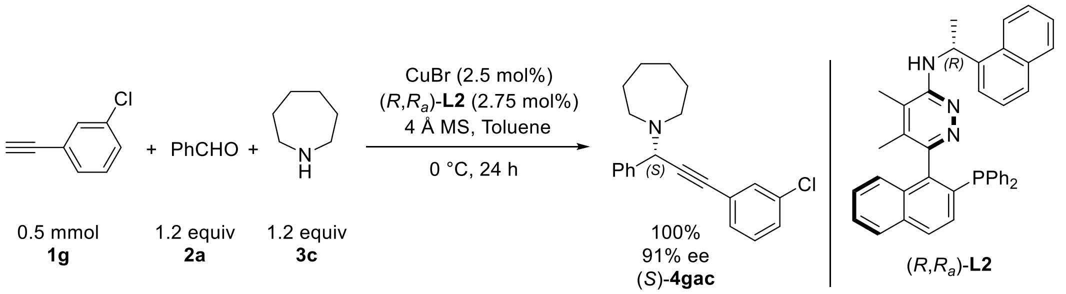

Following **Typical Procedure I**, the reaction of CuBr (1.8 mg, 0.0125 mmol), (*R*,*R*a)-**L2** (8.1 mg, 0.01375 mmol), 4 Å molecular sieves (150.3 mg)/toluene (0.75 mL), **1g** (70.5 mg, 0.5 mmol), **2a** (63.9 mg, 0.6 mmol)/toluene (0.5 mL), and **3c** (59.7 mg, 0.6 mmol) afforded (*S*)-**4gac** (161.9 mg, 100%) (eluent: petroleum ether/ethyl ether = 100:1 (50 mL) to 50:1 (50 mL)) as a liquid: 91% ee (HPLC conditions: Chiralcel AD-H column, hexane/*i*-PrOH = 100/0, 0.2 mL/min, λ = 214 nm, t_R(major) = 26.6 min, t_R(minor) = 31.3 min; $[\alpha]_D^{25}$ = +1.4 (c = 1.01, $CHCl_3$); **^{1}H NMR** (400 MHz, $CDCl_3$) δ 7.66 (d, *J* = 7.6 Hz, 2 H, ArH), 7.49 (s, 1 H, ArH), 7.41-7.32 (m, 3 H, ArH), 7.31-7.20 (m, 3 H, ArH), 4.90 (s, 1 H, NCH), 2.80-2.60 (m, 4 H, 2 × NCH_2), 1.73-1.50 (m, 8 H, 4 × CH_2); **^{13}C NMR** (100 MHz, $CDCl_3$) δ 139.4, 134.1, 131.7, 129.9, 129.5, 128.2, 128.1, 128.0, 127.3, 125.1, 88.3, 85.7, 62.6, 52.7, 29.0, 26.9; **MS** (ESI) *m/z* 326 ([M(^{37}Cl)+H]$^+$), 324 ([M(^{35}Cl)+H]$^+$); **IR** (neat): ν = 3061, 3028, 2924, 2850, 2764, 1591, 1561, 1491, 1473, 1449, 1406, 1391, 1356, 1322, 1299, 1274, 1237, 1184, 1146, 1117, 1091, 1078, 1066, 1029 cm^{-1}; **HRMS** calcd for $C_{21}H_{23}NCl$ ([M(^{35}Cl)+H]$^+$): 324.1514, Found: 324.1505.

30. (*S*)-1-(3-(4-fluorophenyl)-1-phenylprop-2-yn-1-yl)azocane (*S*)-**4had** (Lq-6-196)

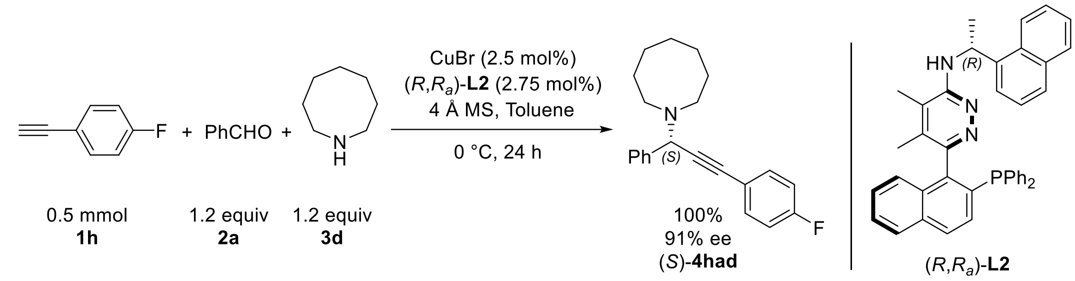

Following **Typical Procedure I**, the reaction of CuBr (1.8 mg, 0.0125 mmol), (R,R_a)-**L2** (8.1 mg, 0.01375 mmol), 4 Å molecular sieves (150.7 mg)/toluene (0.75 mL), **1h** (61.5 mg, 0.5 mmol), **2a** (64.0 mg, 0.6 mmol)/toluene (0.5 mL), and **3d** (68.0 mg, 0.59 mmol) afforded (S)-**4had** (160.2 mg, 100%) (eluent: petroleum ether/ethyl ether = 50:1 (50 mL)) as a liquid: 91% ee, (HPLC conditions: Chiralcel AD-H column, hexane/i-PrOH = 100/0, 0.2 mL/min, λ = 214 nm, t_R(major) = 26.5 min, t_R(minor) = 32.4 min; $[\alpha]_D^{27}$ = -6.3 (c = 1.025, $CHCl_3$); **^{1}H NMR** (400 MHz, $CDCl_3$) δ 7.67 (d, J = 7.6 Hz, 2 H, ArH), 7.47 (dd, J_1 = 7.2 Hz, J_2 = 6.4 Hz, 2 H, ArH), 7.35 (t, J = 7.4 Hz, 2 H, ArH), 7.27 (t, J = 7.2 Hz, 1 H, ArH), 7.01 (t, J = 8.6 Hz, 2 H, ArH), 4.88 (s, 1 H, NCH), 2.77-2.56 (m, 4 H, 2 × NCH_2), 1.80-1.33 (m, 10 H, 5 × CH_2); **^{13}C NMR** (100 MHz, $CDCl_3$) δ 162.3 (d, J = 247.3 Hz), 139.8, 133.6 (d, J = 8.1 Hz), 128.5, 127.9, 127.3, 119.5 (d, J = 3.2 Hz), 115.5 (d, J = 21.7 Hz), 86.5 (d, J = 1.2 Hz), 86.1, 62.5, 51.3, 28.0, 27.8, 25.8; **^{19}F NMR** (376 MHz, $CDCl_3$) δ -112.0; **MS** (ESI) m/z 322 ($[M+H]^+$); **IR** (neat): ν = 3060, 3029, 2918, 2849, 2809, 1601, 1505, 1471, 1450, 1358, 1323, 1272, 1229, 1154, 1126, 1091, 1045, 1014 cm^{-1}; **HRMS** calcd for $C_{22}H_{25}NF$ ($[M+H]^+$): 322.1966, Found: 322.1965.

31. (S)-1-(4-methyl-1-phenylpent-1-yn-3-yl)piperidin-4-one (S)-**4dte** (xhb-3-039)

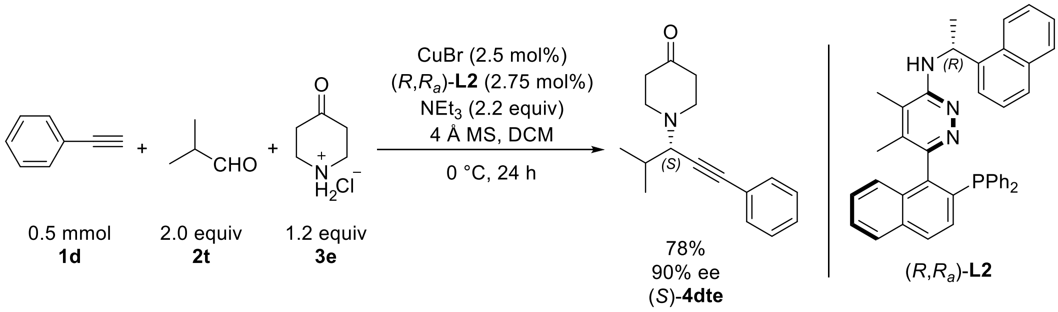

To a flame-dried Schlenk tube were added CuBr (1.8 mg, 0.0125 mmol), (R,R_a)-**L2** (8.1 mg, 0.01375 mmol), 4 Å molecular sieves (150.3 mg), and DCM (0.75 mL)

sequentially under Ar atmosphere. After being stirred at room temperature for 30 min, **3e** (85.6 mg, 0.6 mmol), NEt_3 (113.7 mg, 1.1 mmol), and **1d** (51.2 mg, 0.5 mmol)/DCM (0.5 mL) were added sequentially under Ar atmosphere. The resulting mixture was stirred at 0 ºC for another 10 min followed by the addition of **2t** (72.2 mg, 1.0 mmol). After being stirred at 0 ºC for 24 h, the reaction was complete as monitored by TLC. The resulting mixture was filtrated through a short pad of basic aluminum oxide (200-300 mesh) eluted with dichloromethane/MeOH (10:1, 22 mL). After evaporation, the residue was purified by chromatography on silica gel (eluent: petroleum ether/ethyl acetate = 20:1 (120 mnL) to 10:1 (330 mL)) to afford (*S*)-**4dte**[5] (99.6 mg, 78%) as a white solid: 90% ee (HPLC conditions: Chiralcel OJ-H column, hexane/*i*-PrOH = 99/1, 0.5 mL/min, λ = 214 nm, t_R(major) = 24.1 min, t_R(minor) = 29.8 min); $[\alpha]_D^{31}$ = -22.6 (c = 1.00, $CHCl_3$) (reported value: 85% ee, $[\alpha]_D^{25}$ = -14.8 (c = 0.9, $CHCl_3$)[5]); m.p. 69.3-70.2 ºC (petroleum ether/ethyl acetate, 90% ee) (reported value: 85% ee, 70 ºC[5]); **^{1}H NMR** (400 MHz, $CDCl_3$) δ 7.45-7.37 (m, 2 H, ArH), 7.33-7.24 (m, 3 H, ArH), 3.19 (d, *J* = 10.0 Hz, 1 H, NCH), 3.03-2.95 (m, 2 H, NCH_2), 2.81-2.73 (m, 2 H, NCH_2), 2.56-2.42 (m, 4 H, 2 × OCH_2), 1.93 (dq, J_1 = 16.4 Hz, J_2 = 6.5 Hz, 1 H, CH), 1.15 (d, *J* = 6.8 Hz, 3 H, CH_3), 1.08 (d, *J* = 6.8 Hz, 3 H, CH_3); **^{13}C NMR** (100 MHz, $CDCl_3$) δ 209.5, 131.6, 128.2, 128.0, 123.1, 86.5, 86.2, 64.4, 49.5, 41.6, 31.1, 20.6, 19.8; **MS** (ESI) *m/z* 256 ($[M+H]^+$); **IR** (neat): ν = 2970, 2954, 2915, 2867, 2813, 1710, 1493, 1479, 1469, 1443, 1417, 1380, 1338, 1263, 1211, 1132, 1113, 1102, 1077 cm^{-1}.

32. (*S*)-4-(3-morpholino-3-phenylprop-1-yn-1-yl)benzonitrile (*S*)-**4iaf** (xhb-3-060)

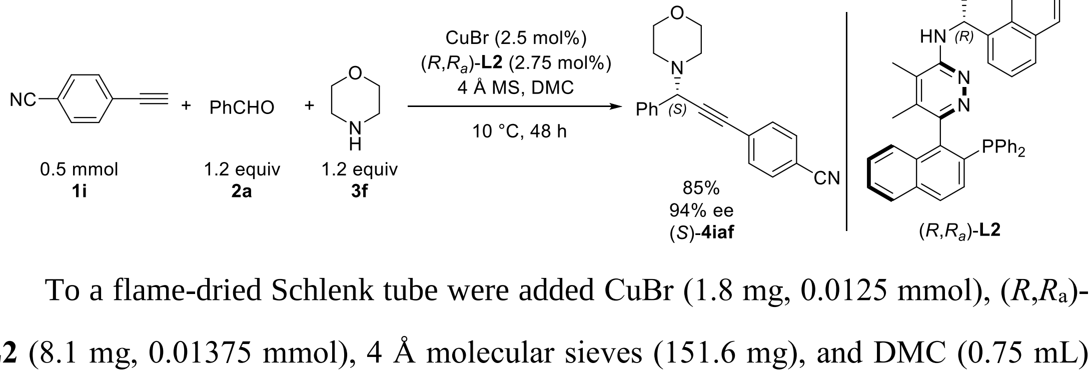

To a flame-dried Schlenk tube were added CuBr (1.8 mg, 0.0125 mmol), (*R*,*R*a)-**L2** (8.1 mg, 0.01375 mmol), 4 Å molecular sieves (151.6 mg), and DMC (0.75 mL) sequentially under Ar atmosphere. After being stirred at room temperature for 30 min,

1i (65.7 mg, 0.5 mmol) and **2a** (63.9 mg, 0.6 mmol)/DMC (0.5 mL) were added sequentially under Ar atmosphere. The resulting mixture was stirred at 10 °C for another 10 min followed by the addition of **3f** (54.1 mg, 0.6 mmol). After being stirred at 10 °C for 48 h, the reaction was complete as monitored by TLC. The resulting mixture was filtrated through a short pad of basic aluminum oxide (200-300 mesh) eluted with dichloromethane/MeOH (10:1, 22 mL). After evaporation, the residue was purified by chromatography on silica gel (eluent: petroleum ether/ethyl acetate = 20:1 (210 mnL) to 10:1 (220 mL)) to afford (*S*)-**4iaf** (128.4 mg, 85%) as an oil: 94% ee (HPLC conditions: Chiralcel OD-H column, hexane/*i*-PrOH = 100/1, 1.0 mL/min, λ = 214 nm, t_R(minor) = 26.3 min, t_R(major) = 23.3 min); $[\alpha]_D^{29}$ = -4.9 (c = 1.00, $CHCl_3$); **^{1}H NMR** (400 MHz, $CDCl_3$) δ 7.67-7.55 (m, 6 H, ArH), 7.42-7.30 (m, 3 H, ArH), 4.81 (s, 1 H, NCH), 3.80-3.66 (m, 4 H, 2 × CH_2), 2.67-2.56 (m, 4 H, 2 × CH_2); **^{13}C NMR** (100 MHz, $CDCl_3$) δ 137.0, 132.3, 132.0, 128.4, 128.3, 128.0, 127.8, 118.4, 111.6, 90.0, 86.9, 67.0, 62.0, 49.9; **MS** (ESI) *m/z* 303 ([M+H]$^+$); **IR** (neat): ν = 2973, 2931, 2852, 2813, 2223, 1600, 1495, 1449, 1403, 1391, 1321, 1270, 1247, 1207, 1177, 1132, 1112, 1073, 1031, 1000 cm^{-1}; **HRMS** calcd for $C_{20}H_{19}N_2O$ ([M+H]$^+$): 303.1492. Found: 303.1488.

33. (*S*)-3-(4-chlorophenyl)-1-phenylprop-2-yn-1-yl *N*,*N*-diallyl amine (*S*)-**4jag** (xhb-3-059)

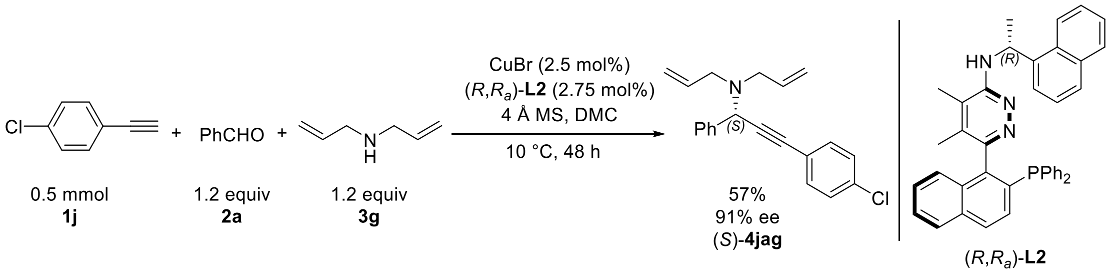

To a flame-dried Schlenk tube were added CuBr (1.8 mg, 0.0125 mmol), (*R*,*R*a)-**L2** (8.1 mg, 0.01375 mmol), 4 Å molecular sieves (150.6 mg), and DMC (0.75 mL) sequentially under Ar atmosphere. After being stirred at room temperature for 30 min, **1j** (70.5 mg, 0.5 mmol) and **2a** (63.7 mg, 0.6 mmol)/DMC (0.5 mL) were added sequentially under Ar atmosphere. The resulting mixture was stirred at 10 °C for another 10 min followed by the addition of **3g** (59.7 mg, 0.6 mmol). After being stirred at 10 °C

for 48 h, the reaction was complete as monitored by TLC. The resulting mixture was filtrated through a short pad of basic aluminum oxide (200-300 mesh) eluted with dichloromethane/MeOH (10:1, 22 mL). After evaporation, the residue was purified by chromatography on silica gel (eluent: petroleum ether (100 mL) to petroleum ether/ethyl acetate = 100:1 (100 mL) to 50:1 (100 mL)) to afford (*S*)-**4jag** (91.7 mg, 57%) as an oil: 91% ee (HPLC conditions: Chiralcel OD-H column, hexane/*i*-PrOH = 100/0, 0.7 mL/min, λ = 214 nm, t_R(minor) = 14.5 min, t_R(major) = 17.1 min); $[\alpha]_D^{27}$ = -120.0 (c = 1.00, $CHCl_3$); **^{1}H NMR** (400 MHz, $CDCl_3$) δ 7.66 (d, *J* = 7.2 Hz, 2 H, ArH), 7.46 (d, *J* = 8.8 Hz, 2 H, ArH), 7.38-7.25 (m, 5 H, ArH), 5.92-5.77 (m, 2 H, 2 × =CH), 5.27 (d, *J* = 17.2 Hz, 2 H, = CH_2), 5.14 (d, *J* = 10.4 Hz, 2 H, = CH_2), 5.09 (s, 1 H, NCH), 3.31-3.23 (m, 2 H, NCH_2), 3.02 (dd, J_1 = 14.2 Hz, J_2 = 8.4 Hz, 2 H, NCH_2); **^{13}C NMR** (100 MHz, $CDCl_3$) δ 139.0, 136.4, 134.1, 133.0, 128.6, 128.2, 128.1, 127.5, 121.7, 117.4, 86.7, 86.5, 56.5, 53.5; **MS** (ESI) *m/z* 324 ([M(^{37}Cl)+H]$^+$), 322 ([M(^{35}Cl)+H]$^+$); **IR** (neat): ν = 3064, 3030, 3007, 2978, 2815, 1642, 1601, 1488, 1448, 1417, 1397, 1351, 1328, 1276, 1111, 1090, 1030, 1014 cm^{-1}; **HRMS** calcd for $C_{21}H_{21}{}^{35}ClN$ ([M+H]$^+$): 322.1357. Found: 322.1353.

34. (*S*)-*N*-allyl-*N*-methyl-1-phenyl-3-(*p*-tolyl)prop-2-yn-1-yl amine (*S*)-**4kah** (xhb-3-063)

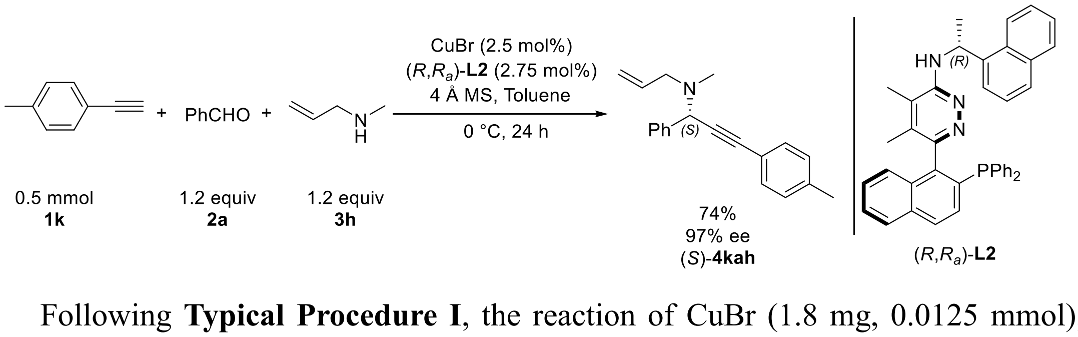

Following **Typical Procedure I**, the reaction of CuBr (1.8 mg, 0.0125 mmol), (*R*,*R*a)-**L2** (8.1 mg, 0.01375 mmol), 4 Å molecular sieves (151.3 mg)/toluene (0.75 mL), **1k** (59.3 mg, 0.5 mmol), **2a** (63.8 mg, 0.6 mmol)/toluene (0.5 mL), and **3h** (44.1 mg, 0.6 mmol) afforded (*S*)-**4kah** (101.9 mg, 74%) (eluent: petroleum ether (100 mL) to petroleum ether/ethyl acetate = 100:1 (100 mL) to 50:1 (100 mL)) as an oil: 97% ee (HPLC conditions: Chiralcel OD-H column, hexane/*i*-PrOH = 100/0, 0.7 mL/min, λ =

214 nm, t_R(minor) = 15.9 min, t_R(major) = 17.5 min); $[\alpha]_D^{28}$ = -60.7 (c = 1.00, $CHCl_3$); **^{1}H NMR** (400 MHz, $CDCl_3$) δ 7.64 (d, J = 7.6 Hz, 2 H, ArH), 7.42 (d, J = 7.6 Hz, 2 H, ArH), 7.36 (t, J = 7.4 Hz, 2 H, ArH), 7.28 (t, J = 7.2 Hz, 1 H, ArH), 7.14 (d, J = 7.6 Hz, 2 H, ArH), 5.98-5.85 (m, 1 H, =CH), 5.28 (dd, J_1 = 17.2 Hz, J_2 = 1.6 Hz, 1 H, one proton of =CH_2), 5.21-5.12 (m, 1 H, one proton of =CH_2), 4.96 (s, 1 H, NCH), 3.18 (d, J = 6.4 Hz, 2 H, NCH_2), 2.36 (s, 3 H, CH_3), 2.22 (s, 3 H, CH_3); **^{13}C NMR** (100 MHz, $CDCl_3$) δ 139.0, 138.2, 136.2, 131.7, 129.0, 128.4, 128.1, 127.5, 120.1, 117.6, 88.4, 84.1, 59.7, 57.8, 37.7, 21.4; **MS** (ESI) *m/z* 276 ($[M+H]^+$); **IR** (neat): ν = 3060, 3028, 2977, 2943, 2920, 2845, 2812, 2787, 1643, 1602, 1509, 1492, 1448, 1421, 1411, 1323, 1274, 1195, 1176, 1126, 1106, 1020 cm^{-1}; **HRMS** calcd for $C_{20}H_{22}N$ ($[M+H]^+$): 276.1747. Found: 276.1747.

35. (*S*)-5-(Dibenzylamino)-2-methyl-5-phenylpent-3-yn-2-ol (*S*)-**4eai** (Lq-5-066)

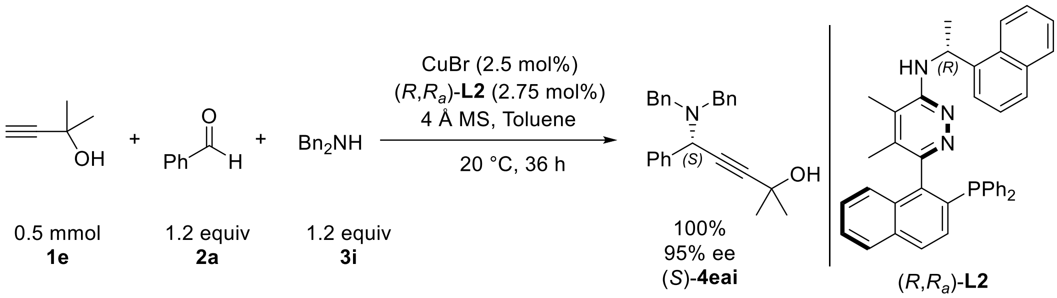

Following **Typical Procedure I**, the reaction of CuBr (1.8 mg, 0.0125 mmol), (*R*,*R*a)-**L2** (8.1 mg, 0.01375 mmol), 4 Å molecular sieves (150.5 mg)/toluene (0.75 mL), **1e** (43.2 mg, 0.5 mmol), **2a** (63.6 mg, 0.6 mmol)/toluene (0.5 mL), and dibenzylamine **3i** (120.5 mg, 0.6 mmol) afforded (*S*)-**4eai** (186.1 mg, 100%) (eluent: petroleum ether/ethyl acetate = 20:1 (420 mL) to 10:1 (330 mL)) as a liquid: 95% ee (HPLC conditions: Chiralcel OD-H column, hexane/*i*-PrOH = 100/1, 1.2 mL/min, λ = 214 nm, t_R(major) = 15.8 min, t_R(minor) = 17.8 min); $[\alpha]_D^{31}$ = -65.2 (c = 1.025, $CHCl_3$) (reported value for (*R*)-isomer: 32% ee, $[\alpha]_D^{20}$ = +38.34 (c = 1, $CHCl_3$)[2]); **^{1}H NMR** (400 MHz, $CDCl_3$) δ 7.63 (d J = 8.0 Hz, 2 H, ArH), 7.39 (d, J = 7.2 Hz, 4 H, ArH), 7.36-7.28 (m, 6 H, ArH), 7.27-7.19 (m, 3 H, ArH), 4.72 (s, 1 H, NCH), 3.71 (d, J = 13.2 Hz, 2 H, 2 × one proton from NCH_2), 3.40 (d, J = 13.6 Hz, 2 H, 2 × one proton from

NCH_2), 2.09 (br, 1 H, OH), 1.71 (s, 6 H, 2 × CH_3); **^{13}C NMR** (100 MHz, $CDCl_3$) δ 139.4, 139.0, 128.8, 128.2, 128.1, 128.0, 127.4, 127.0, 93.6, 76.9, 65.5, 55.3, 54.5, 32.0; **MS** (ESI) *m/z* 370 ($[M+H]^+$); **IR** (neat): ν = 3358, 3085, 3061, 3028, 2979, 2930, 2832, 2807, 1602, 1493, 1451, 1362, 1327, 1274, 1232, 1165, 1116, 1070, 1028 cm^{-1}; **HRMS** calcd for $C_{26}H_{28}NO$ ($[M+H]^+$): 370.2165, Found: 370.2170.

36. (*S*)-5-(Dibenzylamino)-2,7-dimethyloct-3-yn-2-ol (*S*)-**4eui** (Lq-5-065)

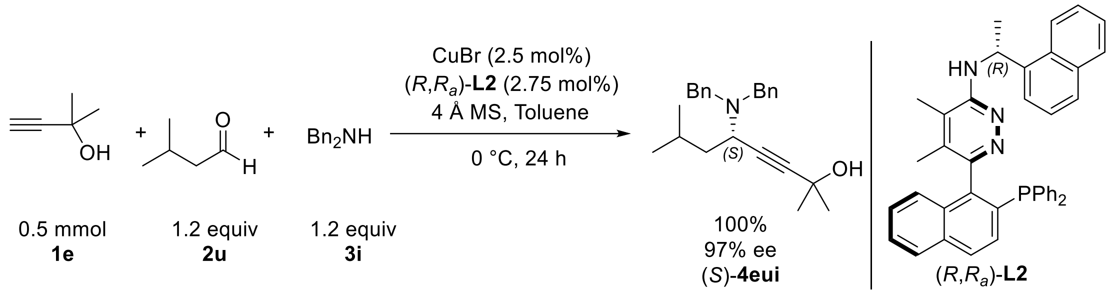

Following **Typical Procedure I**, the reaction of CuBr (1.8 mg, 0.0125 mmol), (*R*,*R*$_a$)-**L2** (8.1 mg, 0.01375 mmol), 4 Å molecular sieves (150.7 mg)/toluene (0.75 mL), **1e** (43.1 mg, 0.5 mmol), **2u** (53.5 mg, 0.6 mmol)/toluene (0.5 mL), and dibenzylamine **3i** (120.2 mg, 0.6 mmol) afforded (*S*)-**4eui** (175.8 mg, 100%) (eluent: petroleum ether/ethyl acetate = 20:1 (315 mL) to 10:1 (330 mL)) as a liquid: 97% ee (HPLC conditions: Chiralcel AD-H column, hexane/*i*-PrOH = 98/2, 1.0 mL/min, λ = 214 nm, t_R(major) = 8.5 min, t_R(minor) = 11.9 min); $[\alpha]_D^{31}$ = -169.4 (c = 1.02, $CHCl_3$); **^{1}H NMR** (400 MHz, $CDCl_3$) δ 7.37 (d, *J* = 7.6 Hz, 4 H, ArH), 7.30 (t, *J* = 7.4 Hz, 4 H, ArH), 7.22 (t, *J* = 7.2 Hz, 2 H, ArH), 3.79 (d, *J* = 13.6 Hz, 2 H, 2 × one proton from NCH_2), 3.47 (t, *J* = 7.6 Hz, 1 H, NCH), 3.34 (d, *J* = 13.6 Hz, 2 H, 2 × one proton from NCH_2), 2.04 (br, 1 H, OH),1.91-1.78 (m, 1 H, CH), 1.69-1.61 (m, 1 H, one proton from CH_2),1.59 (s, 6 H, 2 × CH_3), 1.45-1.35 (m, 1 H, one proton from CH_2),0.78 (d, *J* = 6.8 Hz, 3 H, CH_3, 0.65 (d, *J* = 6.4 Hz, 3 H, CH_3); **^{13}C NMR** (100 MHz, $CDCl_3$) δ 139.7, 128.9, 128.1, 126.8, 89.8, 80.2, 65.3, 54.8, 49.3, 42.8, 32.0, 24.5, 22.8, 21.8; **MS** (ESI) *m/z* 350 ($[M+H]^+$); **IR** (neat): ν = 3339, 3062, 3028, 2954, 2931, 2868, 2807, 1603, 1494, 1453, 1363, 1317, 1232, 1162, 1134, 1111, 1072, 1028 cm^{-1}; **HRMS** calcd for $C_{24}H_{32}NO$ ($[M+H]^+$): 350.2478, Found: 350.2481.

37. (*S*,*E*)-*N*,*N*-Dibenzyl-1-phenyl-5-(trimethylsilyl)pent-1-en-4-yn-3-yl amine (*S*,*E*)-**4lvi** (Lq-5-139)

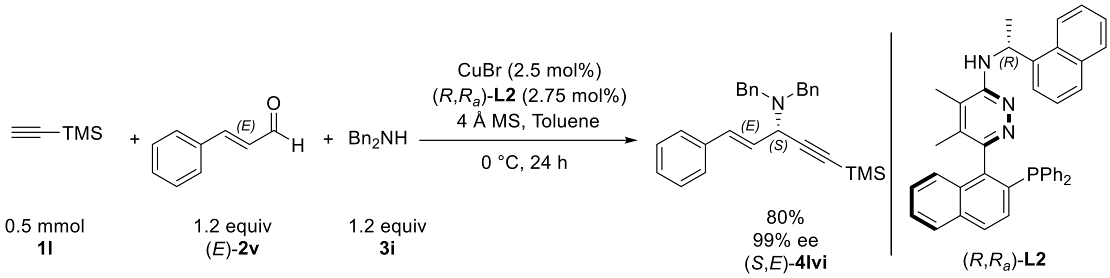

Following **Typical Procedure I**, the reaction of CuBr (1.8 mg, 0.0125 mmol), (*R*,*R*a)-**L2** (8.1 mg, 0.01375 mmol), 4 Å molecular sieves (150.4 mg)/toluene (0.75 mL), **1l** (50.3 mg, 0.5 mmol), (*E*)-**2v** (80.8 mg, 0.6 mmol)/toluene (0.5 mL), and dibenzylamine **3i** (120.1 mg, 0.6 mmol) afforded (*S*,*E*)-**4lvi**[6] (164.1 mg, 80%) (eluent: petroleum ether/ethyl ether = 100:1 (700 mL)) as a liquid: 99% ee (HPLC conditions: Chiralcel OD-H column, hexane/*i*-PrOH = 100/0, 0.2 mL/min, λ = 214 nm, t_R(major) = 43.1 min, t_R(minor) = 40.1 min); $[\alpha]_D^{31}$ = -16.8 (c = 1.095, $CHCl_3$) (reported value: 82% ee, $[\alpha]_D^{20}$ = -13 (c = 0.7, $CHCl_3$)[6]); **^{1}H NMR** (400 MHz, $CDCl_3$) δ 7.47-7.15 (m, 15 H, ArH), 6.89 (d, *J* = 16.0 Hz, 1 H, HC=), 6.19 (dd, J_1 = 16.0 Hz, J_2 = 4.4 Hz, 1 H, HC=), 4.29 (dd, J_1 = 4.2 Hz, J_2 = 1.4 Hz, 1 H, NCH), 3.83 (d, *J* = 13.6 Hz, 2 H, NCH_2), 3.45 (d, *J* = 13.6 Hz, 2 H, NCH_2), 0.30 (s, 9 H, 3 × CH_3); **^{13}C NMR** (100 MHz, $CDCl_3$) δ 139.6, 136.8, 132.6, 128.8, 128.5, 128.3, 128.2, 127.6, 126.9, 126.6, 101.2, 92.6, 54.8, 54.5, 0.4; **MS** (ESI) *m/z* 410 ($[M+H]^+$); **IR** (neat): ν = 3062, 3031, 2957, 2894, 2826, 2152, 1600, 1494, 1449, 1373, 1358, 1307, 1249, 1202, 1102, 1071, 1028 cm^{-1}.

38. (*S*)-*N*,*N*-dibenzyl-1-phenyl-5-(trimethylsilyl)penta-1,4-diyn-3-yl amine (*S*)-**4lwi** (Lq-5-156)

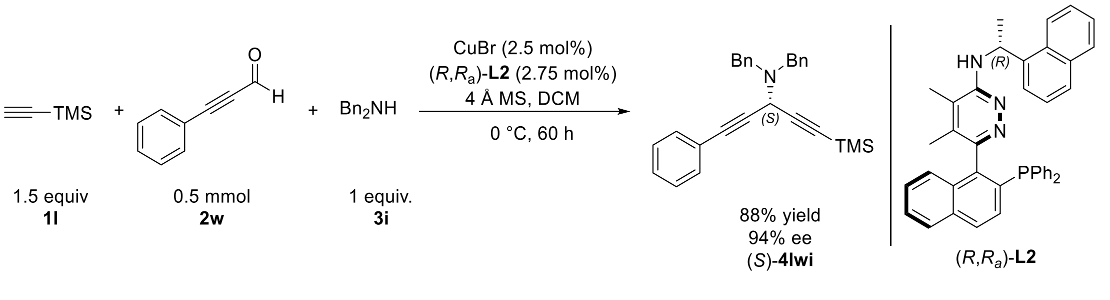

To a flame-dried Schlenk tube were added CuBr (1.8 mg, 0.0125 mmol), (*R*,R_a)-**L2** (8.1 mg, 0.01375 mmol), 4 Å molecular sieves (150.6 mg), and dichloromethane (0.75 mL) sequentially under Ar atmosphere. After being stirred at room temperature for 30 min, **1l** (75.9 mg, 0.75 mmol) and **2w** (66.3 mg, 0.5 mmol)/dichloromethane (0.5 mL) were added sequentially under Ar atmosphere. The resulting mixture was stirred at 0 ºC for another 10 min followed by the addition of **3i** (100.2 mg, 0.5 mmol). After being stirred at 0 ºC for 60 h, the reaction was complete as monitored by TLC. The resulting mixture was filtrated through a short pad of silica gel eluted with ether (40 mL). After evaporation, the residue was purified by chromatography on silica gel (eluent: petroleum ether/ether = 20:1) to afford (*S*)-**4lwi** (178.5 mg, 88%) as a white solid: (m.p. 77.8-78.4 ºC, we were not able to obtain the crystal from all the solvent tested, the m.p. value was determined by using the solid after evaporation of the eluent. (reported value for (*R*)-isomer: 96% ee, m.p. 76 ºC[7])): 94% ee (HPLC conditions: Chiralcel OD-H column, hexane/*i*-PrOH = 100/0, 0.5 mL/min, λ = 214 nm, t_R(major) = 15.1 min, t_R(minor) = 18.0 min); $[\alpha]_D^{27}$= +79.9 (c = 1.06, $CHCl_3$)(reported value for (*R*)-isomer: 96% ee, $[\alpha]_D^{24}$ = -69.65 (c = 1.0, $CHCl_3$)[7]); **^{1}H NMR** (400 MHz, $CDCl_3$) δ 7.51-7.45 (m, 2 H, ArH), 7.45-7.40 (m, 4 H, ArH), 7.35-7.28 (m, 7 H, ArH), 7.28-7.20 (m, 2 H, ArH), 4.57 (s, 1 H, CH), 3.80 (d, *J* = 13.2 Hz, 2 H, NCH_2), 3.75 (d, *J* = 13.2 Hz, 2 H, NCH_2), 0.23 (s, 9 H, 3 × CH_3); **^{13}C NMR** (100 MHz, $CDCl_3$) δ 139.0, 131.9, 129.0, 128.33, 128.29, 128.2, 127.1, 122.7, 100.2, 88.9, 84.4, 84.1, 54.8, 45.3, 0.04; **MS** (ESI) *m/z* 408 ($[M+H]^+$); **IR** (neat): ν = 3060, 3028, 2958, 2840, 2811, 2190, 1661, 1598, 1490, 1453, 1369, 1304, 1286, 1249, 1209, 1106, 1069, 1028 cm^{-1}; **HRMS** calcd for $C_{28}H_{30}NSi$ ($[M+H]^+$): 408.2142, Found: 408.2152.

39. (1*S*,4*S*)-4-(Dibenzylamino)-5-methyl-1-phenyl-2-hexyn-1-ol (*S*,*S*)-**4oti** (Lq-5-073)

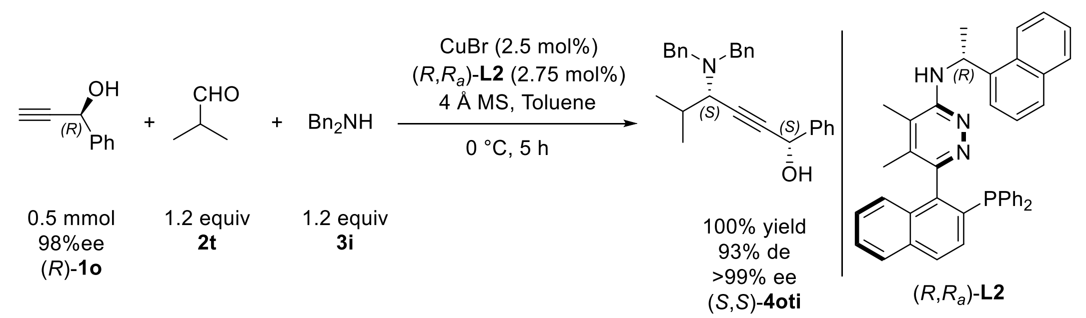

Following **Typical Procedure I**, the reaction of CuBr (1.8 mg, 0.0125 mmol), (R,R_a)-**L2** (8.1 mg, 0.01375 mmol), 4 Å molecular sieves (150.4 mg)/toluene (0.75 mL), (R)-**1o**[8] (67.5 mg, 0.5 mmol), **2t** (43.5 mg, 0.6 mmol)/toluene (0.5 mL), and dibenzylamine **3i** (118.9 mg, 0.6 mmol) afforded (S,S)-**4oti** (200.2 mg, 100%) (eluent: petroleum ether/ethyl acetate = 20:1 (210 mL) to 10:1 (220 mL)) as a liquid: >99% ee, 93% de; (HPLC conditions: Chiralcel OD-H column, hexane/i-PrOH = 95/5, 0.5 mL/min, λ = 214 nm, t_R(major) = 16.4 min, t_R(minor) = 17.8 min, t_R(minor) = 25.9 min, t_R(minor) = 28.3 min); $[\alpha]_D^{28}$ = -205.6 (c = 1.02, $CHCl_3$); **^{1}H NMR** (400 MHz, $CDCl_3$) δ 7.63 (d, J = 7.6 Hz, 2 H, ArH), 7.46-7.33 (m, 7 H, ArH), 7.30 (t, J = 7.6 Hz, 4 H, ArH), 7.22 (t, J = 7.2 Hz, 2 H, ArH), 5.60 (s, 1 H, OCH), 3.83 (d, J = 14.0 Hz, 2 H, 2 × one proton from NCH_2), 3.38 (d, J = 14.0 Hz, 2 H, 2 × one proton from NCH_2), 3.00 (dd, J_1 = 10.4 Hz, J_2 = 1.2 Hz, 1 H, NCH), 2.25 (br, 1 H, OH), 2.02-1.89 (m, 1 H, CH), 1.01 (d, J = 6.4 Hz, 3 H, CH_3), 0.98 (d, J = 6.4 Hz, 3 H, CH_3); **^{13}C NMR** (100 MHz, $CDCl_3$) δ 141.2, 139.5, 128.8, 128.6, 128.3, 128.2, 126.9, 126.7, 85.6, 84.6, 64.8, 59.1, 55.0, 30.6, 20.9, 19.9; **MS** (ESI) m/z 384 ([M+H]$^+$); **IR** (neat): ν = 3344, 3062, 3028, 2958, 2931, 2869, 2808, 1602, 1493, 1453, 1372, 1260, 1155, 1101, 1069, 1027, 1003 cm^{-1}; **HRMS** calcd for $C_{27}H_{30}NO$ ([M+H]$^+$): 384.2322, Found: 384.2316.

40. (1S,4R)-4-(Dibenzylamino)-5-methyl-1-phenyl-2-hexyn-1-ol (S,R)-**4oti** (Lq-5-079)

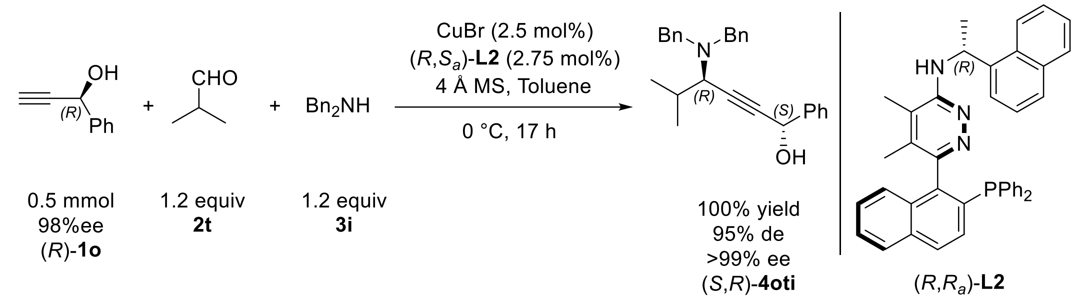

Following **Typical Procedure I**, the reaction of CuBr (1.8 mg, 0.0125 mmol), (R,S_a)-**L2** (8.1 mg, 0.01375 mmol), 4 Å molecular sieves (153.0 mg)/toluene (0.75 mL), (R)-**1o**[8] (67.6 mg, 0.5 mmol), **2t** (43.7 mg, 0.6 mmol)/toluene (0.5 mL), and dibenzylamine **3i** (119.8 mg, 0.6 mmol) afforded (S,R)-**4oti** (196.0 mg, 100%) (eluent: petroleum ether/ethyl acetate = 20:1 (210 mL) to 10:1 (330 mL)) as a liquid: >99% ee, 95% de; (HPLC conditions: Chiralcel OD-H column, hexane/i-PrOH = 95/5, 0.5 mL/min, λ = 214 nm, t_R(major) = 18.0 min, t_R(minor) = 16.4 min, t_R(minor) = 25.6 min, t_R(minor) = 28.0 min); $[\alpha]_D^{29}$ = +181.6 (c = 1.05, $CHCl_3$); **^{1}H NMR** (400 MHz, $CDCl_3$) δ 7.63 (d, J = 7.2 Hz, 2 H, ArH), 7.45-7.33 (m, 7 H, ArH), 7.29 (t, J = 7.4 Hz, 4 H, ArH), 7.21 (t, J = 7.4 Hz, 2 H, ArH), 5.59 (s, 1 H, OCH), 3.83 (d, J = 13.6 Hz, 2 H, 2 × one proton from NCH_2), 3.37 (d, J = 14.0 Hz, 2 H, 2 × one proton from NCH_2), 3.01 (dd, J_1 = 10.2 Hz, J_2 = 1.4 Hz, 1 H, NCH), 2.26 (br, 1 H, OH), 2.02-1.87 (m, 1 H, CH), 1.01 (d, J = 6.8 Hz, 3 H, CH_3), 0.98 (d, J = 6.8 Hz, 3 H, CH_3); **^{13}C NMR** (100 MHz, $CDCl_3$) δ 141.2, 139.5, 128.8, 128.6, 128.3, 128.2, 126.9, 126.7, 85.6, 84.6, 64.8, 59.1, 55.0, 30.6, 20.9, 19.9; **MS** (ESI) m/z 384 ($[M+H]^+$); **IR** (neat): ν = 3343, 3085, 3062, 3028, 2957, 2929, 2869, 2832, 2808, 1602, 1493, 1453, 1365, 1329, 1266, 1155, 1129, 1102, 1069, 1028 cm^{-1}; **HRMS** calcd for $C_{27}H_{30}NO$ ($[M+H]^+$): 384.2322, Found: 384.2321.

41. (1R,4S)-4-(Dibenzylamino)-5-methyl-1-phenyl-2-hexyn-1-ol (R,S)-**4oti** (Lq-5-131)

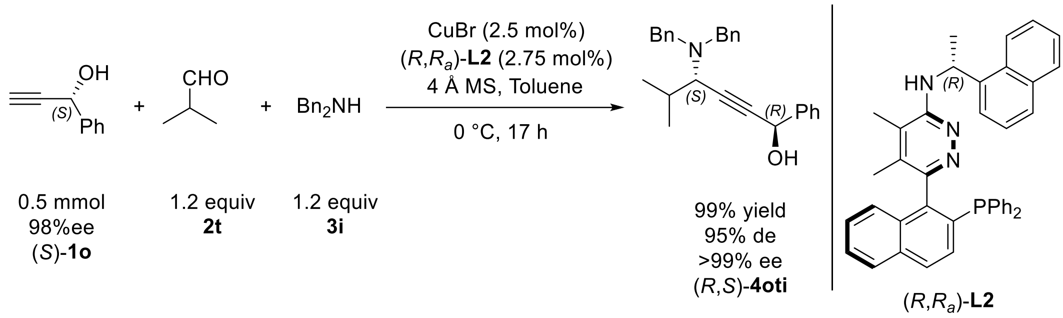

Following **Typical Procedure I**, the reaction of CuBr (1.8 mg, 0.0125 mmol), (*R*,*R*a)-**L2** (8.1 mg, 0.01375 mmol), 4 Å molecular sieves (150.5 mg)/toluene (0.75 mL), (*S*)-**1o**[8] (67.4 mg, 0.5 mmol), **2t** (44.0 mg, 0.6 mmol)/toluene (0.5 mL), and dibenzylamine **3i** (119.6 mg, 0.6 mmol) afforded (*R*,*S*)-**4oti** (194.6 mg, 99%) (eluent: petroleum ether/ethyl acetate = 20:1 (210 mL) to 10:1 (330 mL)) as a liquid: >99% ee, 95% de; (HPLC conditions: Chiralcel OD-H column, hexane/*i*-PrOH = 95/5, 0.5 mL/min, λ = 214 nm, t_R(major) = 26.5 min, t_R(minor) = 16.8 min, t_R(minor) = 18.5 min, t_R(minor) = 29.3 min); $[\alpha]_D^{26}$ = -188.3 (c = 1.015, $CHCl_3$); **^{1}H NMR** (400 MHz, $CDCl_3$) δ 7.63 (d, *J* = 7.2 Hz, 2 H, ArH), 7.46-7.33 (m, 7 H, ArH), 7.29 (t, *J* = 7.4 Hz, 4 H, ArH), 7.21 (t, *J* = 7.0 Hz, 2 H, ArH), 5.59 (s, 1 H, OCH), 3.82 (d, *J* = 13.6 Hz, 2 H, 2 × one proton from NCH_2), 3.38 (d, *J* = 13.6 Hz, 2 H, 2 × one proton from NCH_2), 3.01 (d, *J* = 10.4 Hz, 1 H, NCH), 2.26 (br, 1 H, OH), 2.04-1.85 (m, 1 H, CH), 1.01 (d, *J* = 6.4 Hz, 3 H, CH_3), 0.98 (d, *J* = 6.4 Hz, 3 H, CH_3); **^{13}C NMR** (100 MHz, $CDCl_3$) δ 141.2, 139.5, 128.8, 128.6, 128.3, 128.2, 126.9, 126.7, 85.6, 84.6, 64.9, 59.2, 55.0, 30.6, 20.9, 19.9; **MS** (ESI) *m/z* 384 ($[M+H]^+$); **IR** (neat): ν = 3322, 3085, 3062, 3028, 2958, 2931, 2869, 2832, 2808, 1602, 1493, 1453, 1365, 1264, 1155, 1102, 1069, 1027 cm^{-1}; **HRMS** calcd for $C_{27}H_{30}NO$ ($[M+H]^+$): 384.2322, Found: 384.2330.

42. (1*R*,4*R*)-4-(Dibenzylamino)-5-methyl-1-phenyl-2-hexyn-1-ol (*R*,*R*)-**4oti** (Lq-5-135)

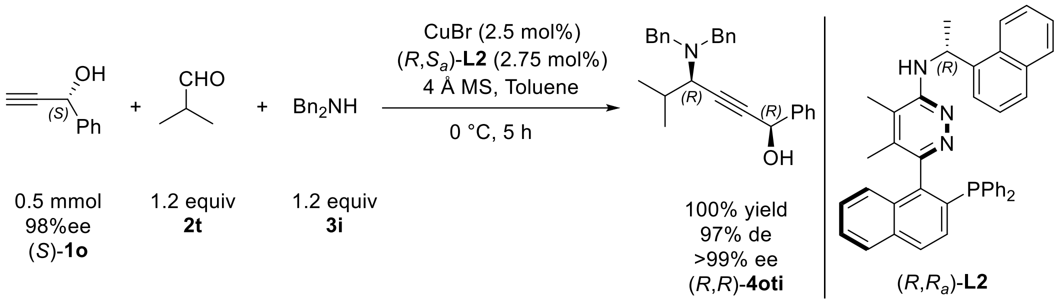

Following **Typical Procedure I**, the reaction of CuBr (1.8 mg, 0.0125 mmol), (*R*,*R*a)-**L2** (8.1 mg, 0.01375 mmol), 4 Å molecular sieves (151.2 mg)/toluene (0.75 mL), (*S*)-**1o**[8] (67.7 mg, 0.5 mmol), **2t** (43.4 mg, 0.6 mmol)/toluene (0.5 mL), and dibenzylamine **3i** (119.7 mg, 0.6 mmol) afforded (*R*,*R*)-**4oti** (195.6 mg, 100%) (eluent: petroleum ether/ethyl acetate = 20:1 (210 mL) to 10:1 (440 mL)) as a liquid: >99% ee, 97% de; (HPLC conditions: Chiralcel OD-H column, hexane/*i*-PrOH = 95/5, 0.5 mL/min, λ = 214 nm, t_R(major) = 28.2 min, t_R(minor) = 18.0 min, t_R(minor) = 25.7 min); $[\alpha]_D^{26}$ = +214.5 (c = 1.03, $CHCl_3$); **^{1}H NMR** (400 MHz, $CDCl_3$) δ 7.62 (d, *J* = 7.2 Hz, 2 H, ArH), 7.45-7.32 (m, 7 H, ArH), 7.29 (t, *J* = 7.4 Hz, 4 H, ArH), 7.21 (t, *J* = 7.2 Hz, 2 H, ArH), 5.59 (s, 1 H, OCH), 3.82 (d, *J* = 14.0 Hz, 2 H, 2 × one proton from NCH_2), 3.38 (d, *J* = 14.0 Hz, 2 H, 2 × one proton from NCH_2), 3.00 (dd, J_1 = 10.4 Hz, J_2 = 1.2 Hz, 1 H, NCH), 2.30 (br, 1 H, OH), 2.02-1.87 (m, 1 H, CH), 1.01 (d, *J* = 6.4 Hz, 3 H, CH_3), 0.97 (d, *J* = 6.4 Hz, 3 H, CH_3); **^{13}C NMR** (100 MHz, $CDCl_3$) δ 141.2, 139.5, 128.8, 128.6, 128.3, 128.2, 126.8, 126.7, 85.6, 84.6, 64.8, 59.1, 55.0, 30.6, 20.9, 19.9; **MS** (ESI) *m/z* 384 ($[M+H]^+$); **IR** (neat): ν = 3339, 3085, 3062, 3028, 2957, 2926, 2869, 2833, 2808, 1602, 1493, 1453, 1372, 1329, 1265, 1155, 1129, 1102, 1069, 1028 cm^{-1}; **HRMS** calcd for $C_{27}H_{30}NO$ ($[M+H]^+$): 384.2322, Found: 384.2328.

43. (*R*)-**4poa** (Lq-7-050)

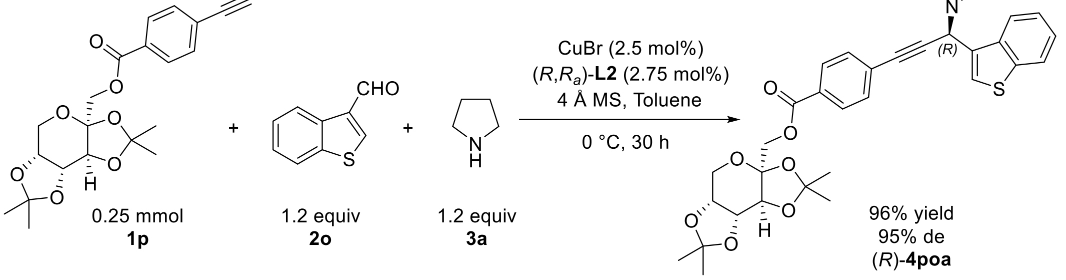

Following **Typical Procedure I**, the reaction of CuBr (0.9 mg, 6.25 μmol), (R,R_a)-**L2** (4.0 mg, 6.88 μmol), 4 Å molecular sieves (75.2 mg)/toluene (0.75 mL), **1p** (97.5 mg, 0.25 mmol), **2o** (53.3 mg, 0.3 mmol)/toluene (0.5 mL), and pyrrolidine **3a** (21.5 mg, 0.3 mmol) afforded (*R*)-**4poa** (146.1 mg, 96%) (eluent: petroleum ether/ethyl acetate = 10:1 (440 mL) to 5:1 (480 mL)) as an oil: 95% de, (HPLC conditions: Chiralcel OZ-H column, hexane/*i*-PrOH = 95/5, 1.0 mL/min, λ = 214 nm, t_R(major) = 11.8 min, t_R(minor) = 15.3 min; $[\alpha]_D^{30}$ = -25.6 (c = 1.0, $CHCl_3$); **^{1}H NMR** (400 MHz, $CDCl_3$) δ 8.15 (d, J = 7.2 Hz, 1 H, ArH), 8.03 (d, J = 8.4 Hz, 2 H, ArH), 7.85 (d, J = 7.2 Hz, 1 H, ArH), 7.62 (s, 1 H, ArH), 7.55 (d, J = 8.4 Hz, 2 H, ArH), 7.42-7.32 (m, 2 H, ArH), 5.28 (s, 1 H, NCH), 4.70 (d, J = 11.6 Hz, 1 H, one proton of CH_2O(C=O)), 4.65 (dd, J_1 = 8.0 Hz, J_2 = 2.4 Hz, 1 H, OCH), 4.46 (d, J = 2.8 Hz, 1 H, OCH), 4.33 (d, J = 11.6 Hz, 1 H, one proton of CH_2O(C=O)), 4.26 (d, J = 7.6 Hz, 1 H, OCH), 3.96 (dd, J_1 = 12.8 Hz, J_2 = 1.6 Hz, 1 H, one proton of CH_2O), 3.81 (d, J = 12.8 Hz, 1 H, one proton of CH_2O), 2.84-2.74 (m, 2 H, two proton from 2 × NCH_2), 2.73-2.64 (m, 2 H, two proton from 2 × NCH_2), 1.88-1.70 (m, 4 H, 2 × CH_2), 1.55 (s, 3 H, CH_3), 1.47 (s, 3 H, CH_3), 1.36 (s, 3 H, CH_3), 1.35 (s, 3 H, CH_3); **^{13}C NMR** (100 MHz, $CDCl_3$) δ 165.4, 140.7, 137.7, 134.3, 131.7, 129.6, 129.2, 128.0, 124.6, 124.4, 123.9, 123.1, 122.7, 109.1, 108.8, 101.6, 89.5, 85.7, 70.7, 70.5, 70.0, 65.3, 61.3, 53.9, 50.0, 26.5, 25.9, 25.5, 24.0, 23.7; **MS** (ESI) *m/z* 604 ([M+H]$^+$); **IR** (neat): ν = 2972, 2935, 2909, 2805, 1724, 1606, 1456, 1428, 1405, 1374, 1344, 1306, 1270, 1251, 1206, 1165, 1106, 1070, 1018, 766 cm^{-1}; **HRMS** calcd for $C_{34}H_{38}O_7NS$ ([M+H]$^+$): 604.2363, Found: 604.2360.

44. Methyl (*S*)-2-((tert-butoxycarbonyl)amino)-3-(4-((*S*)-3-phenyl-3-(1-pyrrolidinyl) prop-1-yn-1-yl)phenyl)propanoate (*S,S*)-**4qaa** (Lq-7-054)

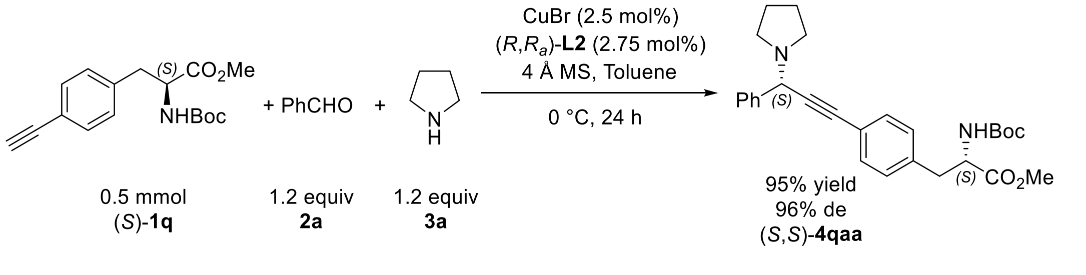

Following **Typical Procedure I**, the reaction of CuBr (1.8 mg, 0.0125 mmol),

(R,R_a)-**L2** (8.1 mg, 0.01375 mmol), 4 Å molecular sieves (150.7 mg)/toluene (0.75 mL), (S)-**1q**[9] (151.4 mg, 0.5 mmol), **2a** (64.0 mg, 0.6 mmol)/toluene (0.5 mL), and pyrrolidine **3a** (42.9 mg, 0.6 mmol) afforded (S,S)-**4qaa** (218.3 mg, 95%) (eluent: petroleum ether/ethyl acetate = 10:1 (110 mL) to 5:1 (120 mL) to 3:1 (200 mL) to 2:1 (300 mL)) as an oil: 96% de, (HPLC conditions: Chiralcel OJ-H column, hexane/*i*-PrOH = 95/5, 1.0 mL/min, λ = 214 nm, t_R(major) = 18.7 min, t_R(minor) = 12.5 min; $[\alpha]_D^{26}$ = +10.8 (c = 1.015, $CHCl_3$); **^{1}H NMR** (400 MHz, $CDCl_3$) δ 7.59 (d, J = 7.2 Hz, 2 H, ArH), 7.41 (d, J = 8.0 Hz, 2 H, ArH), 7.36 (t, J = 7.2 Hz, 2 H, ArH), 7.32-7.27 (m, 1 H, ArH), 7.08 (d, J = 8.0 Hz, 2 H, ArH), 4.98 (d, J = 8.8 Hz, 1 H, NH), 4.88 (s, 1 H, NCH), 4.58 (dd, J_1 = 13.8 Hz, J_2 = 6.2 Hz, 1 H, $NCHCO_2$), 3.71 (s, 3 H, OCH_3), 3.12 (dd, J_1 = 13.6 Hz, J_2 = 5.6 Hz, 1 H, one proton of CH_2), 3.04 (dd, J_1 = 14.0 Hz, J_2 = 6.0 Hz, 1 H, one proton of CH_2), 2.77-2.61 (m, 4 H, 2 × NCH_2), 1.87-1.72 (m, 4 H, 2 × CH_2), 1.42 (s, 9 H, $C(CH_3)_3$); **^{13}C NMR** (100 MHz, $CDCl_3$) δ 172.1, 155.0, 139.4, 136.1, 131.9, 129.2, 128.2, 127.5, 121.9, 86.8, 86.5, 79.9, 59.0, 54.2, 52.2, 50.2, 38.1, 28.2, 23.4; **MS** (ESI) *m/z* 463 ([M+H]$^+$); **IR** (neat): ν = 3368, 3029, 2968, 2875, 2808, 1744, 1711, 1602, 1494, 1449, 1391, 1365, 1249, 1214, 1161, 1107, 1057, 1020, 731, 699 cm^{-1}; **HRMS** calcd for $C_{28}H_{35}O_4N_2$ ([M+H]$^+$): 463.2591, Found: 463.2589.

45. (S)-**4raa** (Lq-7-022)

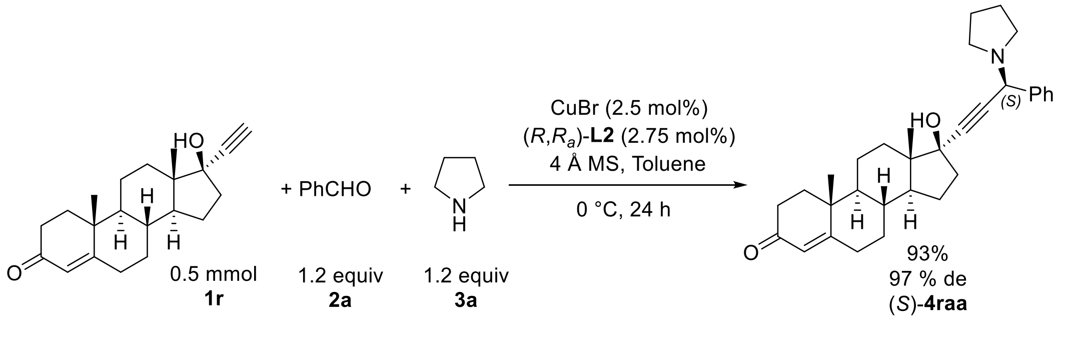

Following **Typical Procedure I**, the reaction of CuBr (1.8 mg, 0.0125 mmol), (R,R_a)-**L2** (8.1 mg, 0.01375 mmol), 4 Å molecular sieves (150.3 mg)/toluene (0.75 mL), **1r** (159.1 mg, 0.5 mmol), **2a** (64.0 mg, 0.6 mmol)/toluene (0.5 mL), and pyrrolidine **3a** (42.9 mg, 0.6 mmol) afforded (S)-**4raa** (217.9 mg, 93%) (eluent: petroleum ether/ethyl acetate/DCM = 5:1:1 (420 mL) to 1:1:1 (180 mL)) as an oil: 97% de, (HPLC conditions:

Chiralcel AD-H column, hexane/*i*-PrOH = 95/5, 1.0 mL/min, λ = 214 nm, t_R(major) = 49.1 min, t_R(minor) = 44.7 min; $[\alpha]_D^{27}$ = -13.6 (c = 1.05, $CHCl_3$); **^{1}H NMR** (400 MHz, $CDCl_3$) δ 7.50 (d, *J* = 7.2 Hz, 2 H, ArH), 7.31 (t, *J* = 7.4 Hz, 2 H, ArH), 7.28-7.22 (m, 1 H, ArH), 5.75 (s, 1 H, =CH), 4.66 (s, 1 H, NCH), 2.69-2.57 (m, 4 H, 2 × NCH_2), 2.56-2.21 (m, 6 H), 2.12-1.97 (m, 2 H), 1.92-1.82 (m, 1 H), 1.81-1.50 (m, 11 H), 1.49-1.29 (m, 2 H), 1.19 (s, 3 H, CH_3), 1.08-0.79 (m, 2 H), 0.91 (s, 3 H, CH_3); **^{13}C NMR** (100 MHz, $CDCl_3$) δ 199.5, 171.2, 139.4, 128.11, 128.10, 127.5, 123.8, 90.1, 83.2, 79.7, 58.7, 53.7, 50.3, 50.0, 46.8, 39.2, 38.5, 36.1, 35.6, 33.8, 32.7, 32.5, 31.6, 23.4, 22.9, 20.7, 17.3, 12.8; **MS** (ESI) *m/z* 472 ($[M+H]^+$); **IR** (neat): ν = 3375, 2941, 2872, 1737, 1662, 1614, 1491, 1449, 1372, 1358, 1330, 1291, 1237, 1187, 1127, 1068, 1044, 699 cm^{-1}; **HRMS** calcd for $C_{32}H_{42}O_2N$ ($[M+H]^+$): 472.3210, Found: 472.3212.

46. (*S*)-**4raa** (Lq-8-038)

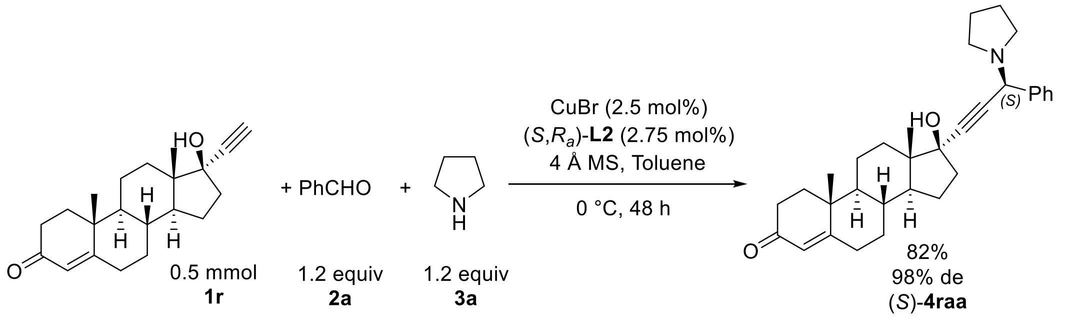

Following **Typical Procedure I**, the reaction of CuBr (1.8 mg, 0.0125 mmol), (*S*,*R*a)-**L2** (8.1 mg, 0.01375 mmol), 4 Å molecular sieves (150.5 mg)/toluene (0.75 mL), **1r** (159.8 mg, 0.5 mmol), **2a** (63.9 mg, 0.6 mmol)/toluene (0.5 mL), and pyrrolidine **3a** (42.8 mg, 0.6 mmol) afforded (*S*)-**4raa** (192.9 mg, 82%) (eluent: petroleum ether/ethyl acetate/DCM = 5:1:1 (420 mL) to 1:1:1 (540 mL)) to DCM/MeOH = 40:1 (400 mL) as an oil: 98% de, (HPLC conditions: Chiralcel OZ-H column, hexane/*i*-PrOH = 80/20, 0.7 mL/min, λ = 214 nm, t_R(major) = 14.4 min, t_R(minor) = 19.0 min; **^{1}H NMR** (400 MHz, $CDCl_3$) δ 7.50 (d, *J* = 7.2 Hz, 2 H, ArH), 7.35-7.23 (m, 3 H, ArH), 5.74 (s, 1 H, =CH), 4.67 (s, 1 H, NCH), 2.69-2.54 (m, 4 H, 2 × NCH_2), 2.49-2.24 (m, 6 H), 2.11-1.97 (m, 2 H), 1.91-1.83 (m, 1 H), 1.82-1.49 (m, 11 H), 1.49-1.30 (m, 2 H), 1.19 (s, 3 H, CH_3), 1.08-0.81 (m, 2 H), 0.91 (s, 3 H, CH_3); **^{13}C NMR** (100 MHz, $CDCl_3$) δ 199.6,

171.2, 139.3, 128.18, 128.15, 127.6, 123.8, 90.2, 83.3, 79.8, 58.8, 53.8, 50.3, 50.1, 46.9, 39.2, 38.6, 36.2, 35.7, 33.9, 32.7, 32.6, 31.6, 23.4, 23.0, 20.7, 17.4, 12.8.

47. (*S*)-**4raa** (Lq-8-040)

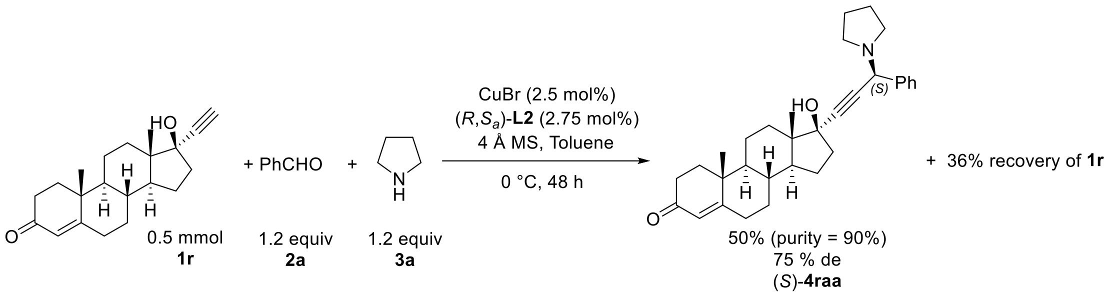

Following **Typical Procedure I**, the reaction of CuBr (1.8 mg, 0.0125 mmol), (*R*,*S*a)-**L2** (8.1 mg, 0.01375 mmol), 4 Å molecular sieves (151.1 mg)/toluene (0.75 mL), **1r** (159.5 mg, 0.5 mmol), **2a** (63.9 mg, 0.6 mmol)/toluene (0.5 mL), and pyrrolidine **3a** (43.0 mg, 0.6 mmol) afforded (*S*)-**4raa**. The recovery of **1r** (36%) was determined by ^{1}H NMR analysis of the crude product using CH_2Br_2 as the internal standard. (*S*)-**4raa** (129.9 mg, 50%, purity 90%) was obtained via column chromatography silica gel (eluent: petroleum ether/ethyl acetate/DCM = 5:1:1 (420 mL) to 1:1:1 (540 mL)) to DCM/MeOH = 40:1 (400 mL) as an oil: 75% de, (HPLC conditions: Chiralcel OZ-H column, hexane/*i*-PrOH = 80/20, 0.7 mL/min, λ = 214 nm, t_R(major) = 14.7 min, t_R(minor) = 18.9 min; **^{1}H NMR** (400 MHz, $CDCl_3$) δ 7.50 (d, *J* = 6.8 Hz, 2 H, ArH), 7.36-7.25 (m, 3 H, ArH), 5.74 (s, 1 H, =CH), [4.70 (s, 0.17 H), 4.67 (s, 0.86 H), 1 H, NCH], 2.70-2.53 (m, 4 H, 2 × NCH_2), 2.49-2.23 (m, 6 H), 2.11-1.97 (m, 2 H), 1.91-1.82 (m, 1 H), 1.81-1.50 (m, 11 H), 1.48-1.31 (m, 2 H), 1.19 (s, 3 H, CH_3), 1.07-0.80 (m, 2 H), 0.91 (s, 3 H, CH_3).

48. (*S*)-**4raa** (Lq-8-039)

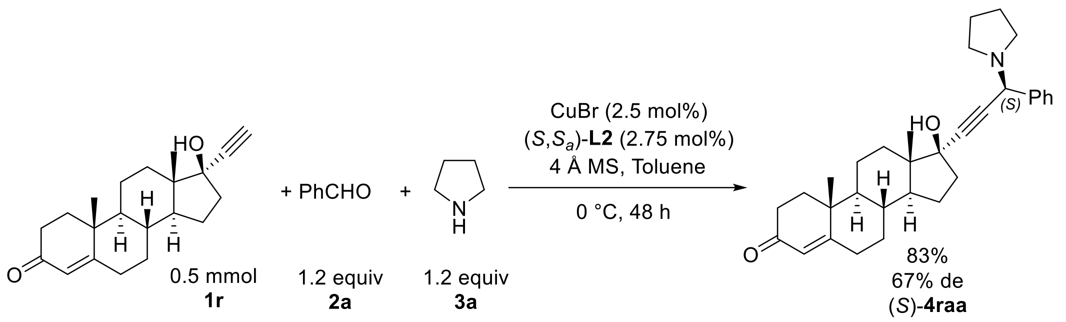

Following **Typical Procedure I**, the reaction of CuBr (1.8 mg, 0.0125 mmol), (*S*,*S*a)-**L2** (8.1 mg, 0.01375 mmol), 4 Å molecular sieves (150.4 mg)/toluene (0.75 mL), **1r** (159.7 mg, 0.5 mmol), **2a** (63.8 mg, 0.6 mmol)/toluene (0.5 mL), and pyrrolidine **3a** (42.9 mg, 0.6 mmol) afforded (*S*)-**4raa** (196.5 mg, 83%) (eluent: petroleum ether/ethyl acetate/DCM = 5:1:1 (420 mL) to 1:1:1 (540 mL)) to DCM/MeOH = 40:1 (400 mL) as an oil: 67% de, (HPLC conditions: Chiralcel OZ-H column, hexane/*i*-PrOH = 80/20, 0.7 mL/min, λ = 214 nm, t_R(major) = 14.4 min, t_R(minor) = 18.5 min; ^{1}**H NMR** (400 MHz, $CDCl_3$) δ 7.50 (d, *J* = 6.8 Hz, 2 H, ArH), 7.36-7.23 (m, 3 H, ArH), 5.74 (s, 1 H, =CH), [4.70 (s, 0.18 H), 4.67 (s, 0.83 H), 1 H, NCH], 2.70-2.57 (m, 4 H, 2 × NCH_2), 2.49-2.22 (m, 6 H), 2.12-1.97 (m, 2 H), 1.92-1.82 (m, 1 H), 1.81-1.50 (m, 11 H), 1.49-1.29 (m, 2 H), 1.19 (s, 3 H, CH_3), 1.09-0.78 (m, 2 H), 0.91 (s, 3 H, CH_3).

49. (*S*)-**4saa** (Lq-7-023)

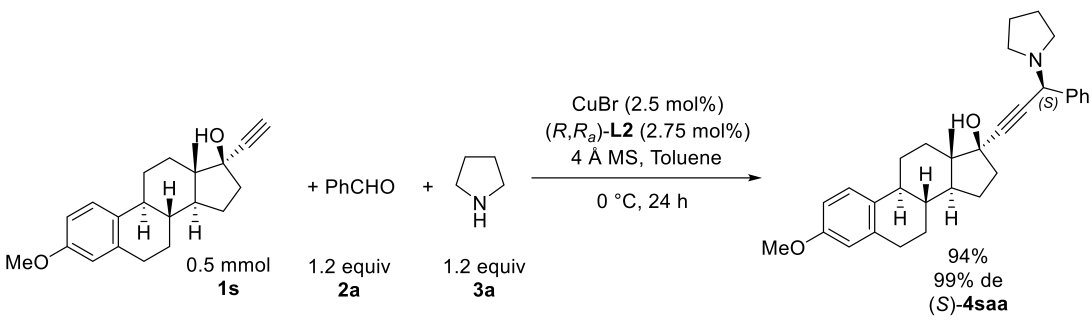

Following **Typical Procedure I**, the reaction of CuBr (1.8 mg, 0.0125 mmol), (*R*,*R*a)-**L2** (8.1 mg, 0.01375 mmol), 4 Å molecular sieves (150.5 mg)/toluene (0.75 mL), **1s** (155.1 mg, 0.5 mmol), **2a** (64.0 mg, 0.6 mmol)/toluene (0.5 mL), and pyrrolidine **3a** (42.9 mg, 0.6 mmol) afforded (*S*)-**4saa** (221.6 mg, 94%) (eluent: petroleum ether/ethyl acetate = 5:1 (360 mL) to petroleum ether/ethyl acetate/DCM = 1:1:1 (180 mL) to DCM/MeOH = 20:1 (420 mL)) as a white foam (m.p. 53.6-61.9 ºC, we were not able

to obtain the crystal from all the solvent tested, the m.p. value was determined by using the solid after evaporation of the eluent.): 99% de, (HPLC conditions: Chiralcel AD-H column, hexane/*i*-PrOH = 95/5, 1.0 mL/min, λ = 214 nm, t_R(major) = 11.7 min, t_R(minor) = 10.6 min; $[\alpha]_D^{28}$ = -25.1 (c = 1.03, $CHCl_3$); **^{1}H NMR** (400 MHz, $CDCl_3$) δ 7.53 (d, *J* = 7.2 Hz, 2 H, ArH), 7.32 (t, *J* = 7.4 Hz, 2 H, ArH), 7.28-7.24 (m, 1 H, ArH), 7.21 (d, *J* = 8.8 Hz, 1 H, ArH), 6.71 (dd, J_1 = 8.6 Hz, J_2 = 2.6 Hz, 1 H, ArH), 6.63 (d, *J* = 2.4 Hz, 1 H, ArH), 4.72 (s, 1 H, NCH), 3.77 (s, 3 H, CH_3), 2.94-2.77 (m, 2 H), 2.71-2.54 (m, 4 H, 2 × NCH_2), 2.42-2.01 (m, 6 H), 1.98-1.70 (m, 8 H), 1.56-1.22 (m, 4 H), 0.90 (s, 3 H, CH_3); **^{13}C NMR** (100 MHz, $CDCl_3$) δ 157.4, 139.4, 137.9, 132.5, 128.18, 128.17, 127.5, 126.3, 113.8, 111.5, 90.4, 83.1, 80.0, 58.7, 55.1, 50.3, 49.7, 47.3, 43.7, 39.4, 32.9, 29.8, 27.4, 26.4, 23.5, 22.8, 12.8; **MS** (ESI) *m/z* 470 ([M+H]$^+$); **IR** (neat): ν = 3434, 2928, 2869, 2831, 1737, 1608, 1576, 1498, 1451, 1378, 1344, 1279, 1253, 1235, 1184, 1146, 1126, 1100, 1071, 1029, 1003, 698 cm^{-1}; **HRMS** calcd for $C_{32}H_{40}O_2N$ ([M+H]$^+$): 470.3054, Found: 470.3056.

50. (*S*)-**4sxa** (Lq-7-082)

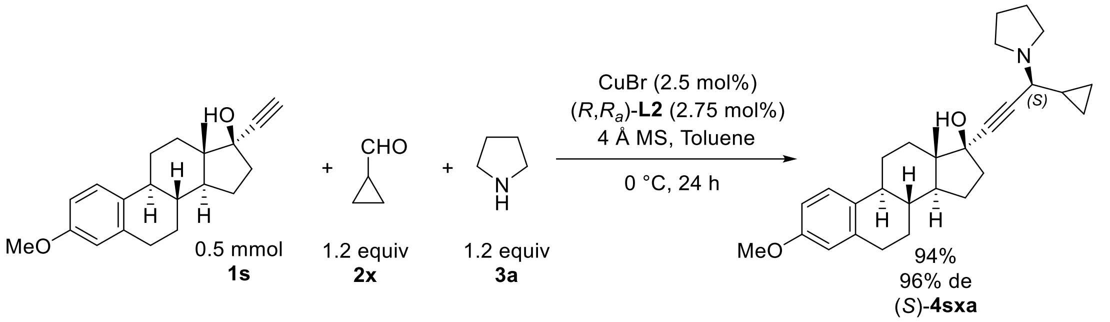

Following **Typical Procedure I**, the reaction of CuBr (1.8 mg, 0.0125 mmol), (*R*,*R*a)-**L2** (8.1 mg, 0.01375 mmol), 4 Å molecular sieves (150.7 mg)/toluene (0.75 mL), **1s** (154.7 mg, 0.5 mmol), **2x** (43.0 mg, 0.6 mmol)/toluene (0.5 mL), and pyrrolidine **3a** (42.9 mg, 0.6 mmol) afforded (*S*)-**4sxa** (202.1 mg, 94%) (eluent: petroleum ether/ethyl acetate/DCM = 1:1:1 (180 mL) to DCM/MeOH = 20:1 (525 mL) to 10:1 (220 mL)) as a white solid: 96% de, (HPLC conditions: Chiralcel AD-H column, hexane/*i*-PrOH = 90/10, 0.5 mL/min, λ = 214 nm, t_R(major) = 11.7 min, t_R(minor) = 13.3 min; m.p. 184.9-186.6 °C (ethyl acetate/hexane, 98% de); 98% de, $[\alpha]_D^{20}$ = +5.7 (c = 1.05, $CHCl_3$); **^{1}H**

NMR (400 MHz, $CDCl_3$) δ 7.24 (d, J = 8.6 Hz, 1 H, ArH), 6.72 (dd, J_1 = 8.2 Hz, J_2 = 2.2 Hz, 1 H, ArH), 6.64 (s, 1 H, ArH), 3.78 (s, 3 H, CH_3), 3.58 (d, J = 5.6 Hz, 1 H, NCH), 2.95-2.60 (m, 7 H), 2.42-2.33 (m, 1 H), 2.32-2.13 (m, 2 H), 2.10-1.97 (m, 1 H), 1.94-1.64 (m, 9 H), 1.56-1.26 (m, 4 H), 1.19-1.06 (m, 1 H, one proton of cyclopropane), 0.87 (s, 3 H, CH_3), 0.57-0.34 (m, 4 H, protons of cyclopropane); **^{13}C NMR** (100 MHz, $CDCl_3$) δ 157.4, 137.9, 132.4, 126.3, 113.8, 111.5, 89.3, 81.4, 79.9, 57.9, 55.2, 50.3, 49.6, 47.1, 43.8, 39.5, 39.3, 32.9, 29.7, 27.3, 26.3, 23.4, 22.6, 14.0, 12.7, 3.1, 2.4; **MS** (ESI) *m/z* 434 ($[M+H]^+$); **IR** (neat): ν = 3082, 3000, 2982, 2943, 2913, 2868, 2818, 2685, 1609, 1573, 1495, 1469, 1453, 1445, 1429, 1379, 1367, 1350, 1315, 1306, 1276, 1251, 1221, 1190, 1148, 1139, 1122, 1096, 1073, 1051, 1035, 1020, 1004 cm^{-1}; Anal. Calcd. for $C_{29}H_{39}NO_2$: C 80.33, H 9.07, N 3.23; Found: C 80.62, H 9.21, N 3.12.

51. (*S*,*R*)-**4tba** (xhb-3-139)

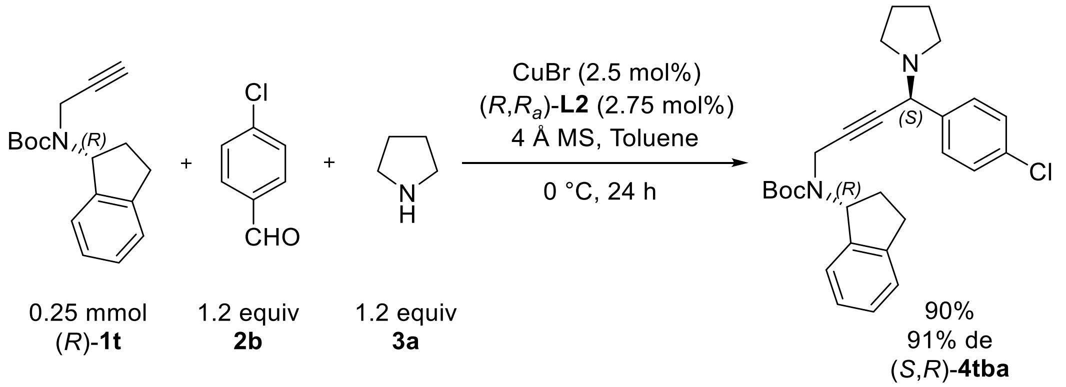

Following **Typical Procedure I**, the reaction of CuBr (0.9 mg, 6.25 μmol), (*R*,R_a)-**L2** (4.0 mg, 6.88 μmol), 4 Å molecular sieves (75.6 mg)/toluene (0.75 mL), (*R*)-**1t** (67.8 mg, 0.25 mmol), **2b** (43.2 mg, 0.3 mmol)/toluene (0.5 mL), and pyrrolidine **3a** (21.4 mg, 0.3 mmol) afforded (*S*,*R*)-**4tba** (104.6 mg, 90%) (eluent: petroleum ether/ethyl acetate = 10:1 (220 mL) to 5:1 (360 mL)) as an oil: 91% de (HPLC conditions: Chiralcel AD-H column, hexane/*i*-PrOH = 100/1, 1.0 mL/min, λ = 214 nm, t_R(minor) = 16.4 min, t_R(major) = 22.6 min); $[\alpha]_D^{20}$ = -35.7 (c = 1.00, $CHCl_3$); **^{1}H NMR** (400 MHz, d_6-DMSO, 373 K) δ 7.44 (dd, J_1 = 5.8 Hz, J_2 = 1.6 Hz, 2 H, ArH), 7.35-7.30 (m, 2 H, ArH), 7.23-7.11 (m, 4 H, ArH), 5.50 (s, 1 H, NCH), 4.68 (s, 1 H, NCH), 4.03 (d, J = 12.0 Hz, 1 H, one proton of NCH_2), 3.74 (d, J = 11.6 Hz, 1 H, one proton of NCH_2), 3.01-2.96

(m, 1 H, one proton of CH_2), 2.83-2.77 (m, 1 H, one proton of CH_2), 2.57-2.51 (m, 2 H, NCH_2), 2.49-2.47 (m, 2 H, NCH_2), 2.39-2.33 (m, 1 H, one proton of CH_2), 2.19-2.12 (m, 1 H, one proton of CH_2), 1.71-1.64 (m, 4 H, 2×CH_2), 1.38 (s, 9 H, 3×CH_3); **^{13}C NMR** (100 MHz, d_6-DMSO, 373 K) δ 153.9, 142.5, 141.1, 138.2, 131.5, 128.9, 127.4, 126.9, 125.7, 124.1, 123.4, 84.2, 84.2, 78.9, 61.2, 56.5, 48.8, 33.4, 29.3, 29.2, 27.5, 22.7; **MS** (ESI) *m/z* 467 ([M(^{37}Cl)+H]$^+$) 465 ([M(^{35}Cl)+H]$^+$); **IR** (neat): ν = 2968, 2931, 2874, 2812, 1692, 1488, 1456, 1437, 1401, 1364, 1327, 1252, 1223, 1158, 1113, 1089, 1015 cm^{-1}; **HRMS** calcd for $C_{28}H_{34}{}^{35}ClN_2O_2$ ([M+H]$^+$): 465.2303. Found: 465.2300.

52. (*R*)-**4unb** (Lq-7-061)

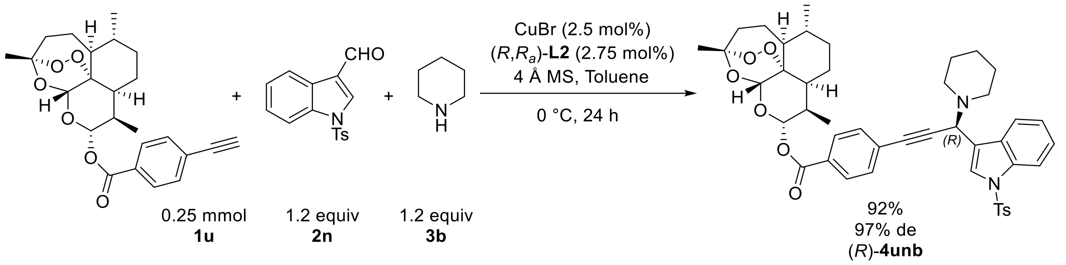

Following **Typical Procedure I**, the reaction of CuBr (0.9 mg, 6.25 μmol), (*R*,*R*a)-**L2** (4.0 mg, 6.88 μmol), 4 Å molecular sieves (75.9 mg)/toluene (0.75 mL), **1u** (103.2 mg, 0.25 mmol), **2n** (90.1 mg, 0.3 mmol)/toluene (0.5 mL), and **3b** (26.2 mg, 0.3 mmol) afforded (*R*)-**4unb** (179.2 mg, 92%) (eluent: petroleum ether/ethyl acetate/DCM = 20:1:1 (220 mL) to 10:1:1 (720 mL)) as a white foam (we were not able to obtain the crystal from the solvent tested, the m.p. was determined by using the solid after evaporation of the eluent. When the white solid was heated up to 73.5 °C, it expanded. At 122.7 °C, the solid started melting. At 132.3 °C, the sample was completely melted and the color of the liquid was yellow); 97% de, (HPLC conditions: Chiralcel OD-H column, hexane/*i*-PrOH = 90/10, 1.0 mL/min, λ = 214 nm, t_R(major) = 28.5 min, t_R(minor) = 47.1 min; $[\alpha]_D^{22}$ = +16.7 (c = 1.02, $CHCl_3$); **^{1}H NMR** (400 MHz, $CDCl_3$) δ 8.11 (d, *J* = 8.4 Hz, 2 H, ArH), 7.96 (d, *J* = 8.4 Hz, 1 H, ArH), 7.93 (d, *J* = 8.0 Hz, 1 H, ArH), 7.77 (d, *J* = 8.0 Hz, 2 H, ArH), 7.69 (s, 1 H, ArH), 7.59 (d, *J* = 8.4 Hz, 2 H, ArH), 7.31 (t, *J* = 7.6 Hz, 1 H, ArH), 7.26-7.18 (m, 3 H, ArH), 6.03 (d, *J* = 9.6 Hz, 1 H, OCHO), 5.54 (s, 1 H, OCHO), 5.01 (s, 1 H, NCH), 2.83-2.72 (m, 1 H), 2.67-2.50 (m, 4

H, 2 × NCH_2), 2.40 (td, J_1 = 14.5 Hz, J_2 = 2.67 Hz, 1 H), 2.33 (s, 3 H, CH_3), 2.11-1.99 (m, 1 H), 1.98-1.21 (m, 14 H), 1.44 (s, 3 H, CH_3), 1.12-1.02 (m, 1 H), 0.99 (d, J = 5.6 Hz, 3 H, CH_3), 0.94 (d, J = 7.2 Hz, 3 H, CH_3); **^{13}C NMR** (100 MHz, $CDCl_3$) δ 164.7, 144.9, 135.6, 135.1, 131.7, 130.0, 129.8, 129.6, 129.0, 128.0, 126.8, 125.3, 124.7, 123.0, 121.5, 120.6, 113.5, 104.4, 92.6, 91.6, 88.3, 86.1, 80.2, 55.5, 51.6, 50.4, 45.3, 37.2, 36.2, 34.0, 31.9, 26.1, 25.9, 24.5, 24.3, 22.0, 21.5, 20.2, 12.2; **MS** (ESI) *m/z* 801 ($[M+Na]^+$), 817 ($[M+K]^+$); **IR** (neat): ν = 2926, 2853, 2801, 1730, 1605, 1445, 1404, 1373, 1263, 1209, 1174, 1129, 1119, 1089, 1035, 1012 cm^{-1}; Anal. Calcd. for $C_{45}H_{50}N_2O_8S$: C 69.39, H 6.47, N 3.60; Found: C 68.85, H 6.83, N 3.31; **HRMS** calcd for $C_{45}H_{51}O_8N_2S$ ($[M+H]^+$): 779.3361, Found: 779.3367.

53. (*R*)-**4unb** (Lq-8-013)

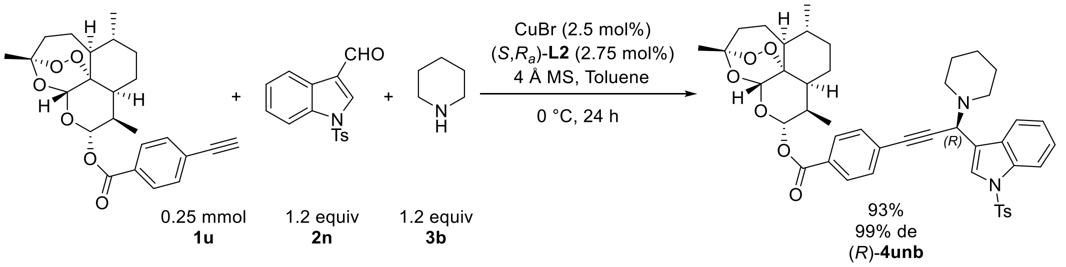

Following **Typical Procedure I**, the reaction of CuBr (0.9 mg, 6.25 μmol), (*S*,*R*a)-**L2** (4.0 mg, 6.88 μmol), 4 Å molecular sieves (75.6 mg)/toluene (0.75 mL), **1u** (103.4 mg, 0.25 mmol), **2n** (89.9 mg, 0.3 mmol)/toluene (0.5 mL), and **3b** (26.1 mg, 0.3 mmol) afforded (*R*)-**4unb** (181.2 mg, 93%) (eluent: petroleum ether/ethyl acetate/DCM = 20:1:1 (440 mL) to 10:1:1 (480 mL)) as a white foam; 99% de, (HPLC conditions: Chiralcel OD-H column, hexane/*i*-PrOH = 90/10, 1.0 mL/min, λ = 214 nm, t_R(major) = 21.6 min, t_R(minor) = 34.0 min); **^{1}H NMR** (400 MHz, $CDCl_3$) δ 8.11 (d, J = 8.4 Hz, 2 H, ArH), 7.96 (d, J = 8.4 Hz, 1 H, ArH), 7.92 (d, J = 7.6 Hz, 1 H, ArH), 7.77 (d, J = 8.4 Hz, 2 H, ArH), 7.68 (s, 1 H, ArH), 7.59 (d, J = 8.0 Hz, 2 H, ArH), 7.31 (t, J = 7.8 Hz, 1 H, ArH), 7.25-7.18 (m, 3 H, ArH), 6.02 (d, J = 10.0 Hz, 1 H, OCHO), 5.54 (s, 1 H, OCHO), 5.00 (s, 1 H, NCH), 2.84-2.71 (m, 1 H), 2.65-2.49 (m, 4 H, 2 × NCH_2), 2.40 (td, J_1 = 13.9 Hz, J_2 = 3.73 Hz, 1 H), 2.33 (s, 3 H, CH_3), 2.10-2.02 (m, 1 H), 1.97-1.23 (m, 14 H), 1.44 (s, 3 H, CH_3), 1.12-1.02 (m, 1 H), 0.99 (d, J = 6.0 Hz, 3 H, CH_3), 0.94

(d, J = 7.2 Hz, 3 H, CH_3); **^{13}C NMR** (100 MHz, $CDCl_3$) δ 164.7, 144.9, 135.6, 135.2, 131.8, 130.0, 129.8, 129.7, 129.0, 128.1, 126.8, 125.3, 124.7, 123.0, 121.5, 120.6, 113.5, 104.4, 92.7, 91.6, 88.3, 86.1, 80.2, 55.5, 51.6, 50.4, 45.3, 37.2, 36.2, 34.1, 32.0, 26.1, 25.9, 24.5, 24.4, 22.0, 21.5, 20.2, 12.2.

54. (*S*)-**4unb** (Lq-7-066)

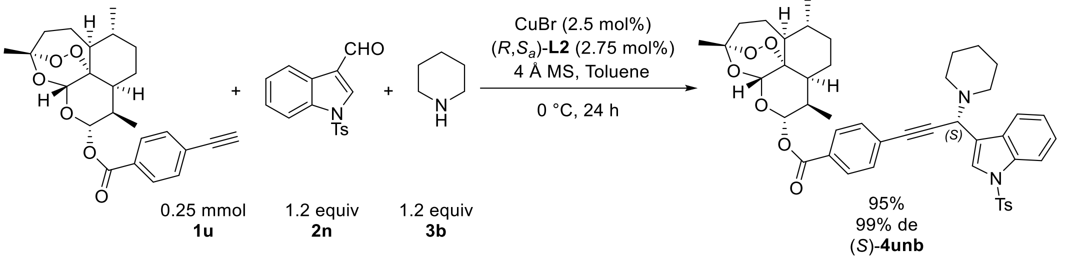

Following **Typical Procedure I**, the reaction of CuBr (0.9 mg, 6.25 μmol), (R,S_a)-**L2** (4.0 mg, 6.88 μmol), 4 Å molecular sieves (75.4 mg)/toluene (0.75 mL), **1u** (103.3 mg, 0.25 mmol), **2n** (89.9 mg, 0.3 mmol)/toluene (0.5 mL), and **3b** (26.0 mg, 0.3 mmol) afforded (*S*)-**4unb** (185.7 mg, 95%) (eluent: petroleum ether/ethyl acetate/DCM = 20:1:1 (220 mL) to 10:1:1 (720 mL)) as an oil: 99% de, (HPLC conditions: Chiralcel OD-H column, hexane/*i*-PrOH = 90/10, 1.0 mL/min, λ = 214 nm, t_R(major) = 42.1 min, t_R(minor) = 29.1 min; $[\alpha]_D^{23}$ = +11.4 (c = 1.1, $CHCl_3$); **^{1}H NMR** (400 MHz, $CDCl_3$) δ 8.11 (d, J = 8.4 Hz, 2 H, ArH), 7.96 (d, J = 8.4 Hz, 1 H, ArH), 7.93 (d, J = 8.0 Hz, 1 H, ArH), 7.77 (d, J = 8.4 Hz, 2 H, ArH), 7.68 (s, 1 H, ArH), 7.59 (d, J = 8.4 Hz, 2 H, ArH), 7.31 (t, J = 7.4 Hz, 1 H, ArH), 7.25-7.18 (m, 3 H, ArH), 6.03 (d, J = 10.0 Hz, 1 H, OCHO), 5.54 (s, 1 H, OCHO), 5.00 (s, 1 H, NCH), 2.84-2.71 (m, 1 H), 2.66-2.49 (m, 4 H, 2 × NCH_2), 2.40 (td, J_1 = 14.1 Hz, J_2 = 3.7 Hz, 1 H), 2.34 (s, 3 H, CH_3), 2.10-1.99 (m, 1 H), 1.97-1.23 (m, 14 H), 1.44 (s, 3 H, CH_3), 1.12-1.02 (m, 1 H), 0.99 (d, J = 6.0 Hz, 3 H, CH_3), 0.94 (d, J = 7.6 Hz, 3 H, CH_3); **^{13}C NMR** (100 MHz, $CDCl_3$) δ 164.7, 144.8, 135.7, 135.2, 131.7, 130.0, 129.8, 129.7, 129.0, 128.1, 126.8, 125.3, 124.7, 123.0, 121.5, 120.7, 113.5, 104.4, 92.7, 91.6, 88.3, 86.1, 80.1, 55.5, 51.6, 50.5, 45.3, 37.2, 36.2, 34.1, 32.0, 26.1, 25.9, 24.5, 24.4, 22.0, 21.5, 20.2, 12.2; **MS** (ESI) *m/z* 801 ([M+Na]$^+$), 817 ([M+K]$^+$); **IR** (neat): ν = 2930, 2874, 2803, 1731, 1605, 1445, 1373, 1264, 1209, 1174, 1129, 1119, 1090, 1035, 1012 cm^{-1}; **HRMS** calcd for $C_{45}H_{51}O_8N_2S$ ([M+H]$^+$):

779.3361, Found: 779.3369.

55. (*S*)-**4unb** (Lq-8-017)

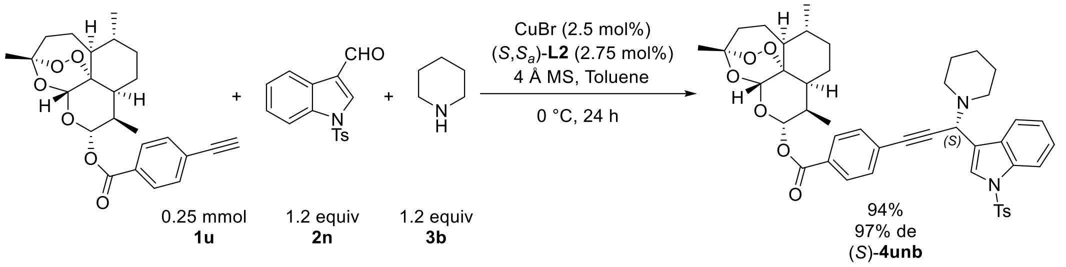

Following **Typical Procedure I**, the reaction of CuBr (0.9 mg, 6.25 μmol), (*R*,*S*a)-**L2** (4.0 mg, 6.88 μmol), 4 Å molecular sieves (75.4 mg)/toluene (0.75 mL), **1u** (103.2 mg, 0.25 mmol), **2n** (90.2 mg, 0.3 mmol)/toluene (0.5 mL), and **3b** (25.9 mg, 0.3 mmol) afforded (*S*)-**4unb** (181.2 mg, 94%) (eluent: petroleum ether/ethyl acetate/DCM = 20:1:1 (220 mL) to 10:1:1 (480 mL) to petroleum ether/ethyl acetate = 5:1 (360 mL)) as an oil: 97% de, (HPLC conditions: Chiralcel OD-H column, hexane/*i*-PrOH = 90/10, 1.0 mL/min, λ = 214 nm, t_R(major) = 31.3 min, t_R(minor) = 21.8 min; **^{1}H NMR** (400 MHz, $CDCl_3$) δ 8.11 (d, *J* = 8.4 Hz, 2 H, ArH), 7.96 (d, *J* = 8.4 Hz, 1 H, ArH), 7.92 (d, *J* = 8.0 Hz, 1 H, ArH), 7.77 (d, *J* = 8.4 Hz, 2 H, ArH), 7.68 (d, *J* = 1.2 Hz, 1 H, ArH), 7.59 (d, *J* = 8.0 Hz, 2 H, ArH), 7.31 (t, *J* = 7.8 Hz, 1 H, ArH), 7.26-7.19 (m, 3 H, ArH), 6.02 (d, *J* = 10.0 Hz, 1 H, OCHO), 5.54 (s, 1 H, OCHO), 5.00 (d, *J* = 1.2 Hz, 1 H, NCH), 2.85-2.70 (m, 1 H), 2.64-2.51 (m, 4 H, 2 × NCH_2), 2.40 (td, J_1 = 13.9 Hz, J_2 = 3.9 Hz, 1 H), 2.34 (s, 3 H, CH_3), 2.11-2.01 (m, 1 H), 1.97-1.23 (m, 14 H), 1.44 (s, 3 H, CH_3), 1.12-1.02 (m, 1 H), 0.99 (d, *J* = 6.0 Hz, 3 H, CH_3), 0.94 (d, *J* = 7.2 Hz, 3 H, CH_3); **^{13}C NMR** (100 MHz, $CDCl_3$) δ 164.7, 144.9, 135.7, 135.2, 131.8, 130.0, 129.8, 129.7, 129.0, 128.1, 126.8, 125.3, 124.7, 123.0, 121.5, 120.7, 113.5, 104.5, 92.7, 91.6, 88.3, 86.1, 80.2, 55.5, 51.6, 50.5, 45.3, 37.2, 36.2, 34.1, 32.0, 26.1, 25.9, 24.5, 24.4, 22.0, 21.5, 20.2, 12.2.

56. (*S*)-1-(1,3-diphenylprop-2-yn-1-yl)-4-(2,3,4-trimethoxybenzyl)piperazine (*S*)-**4daj** (xhb-3-103)

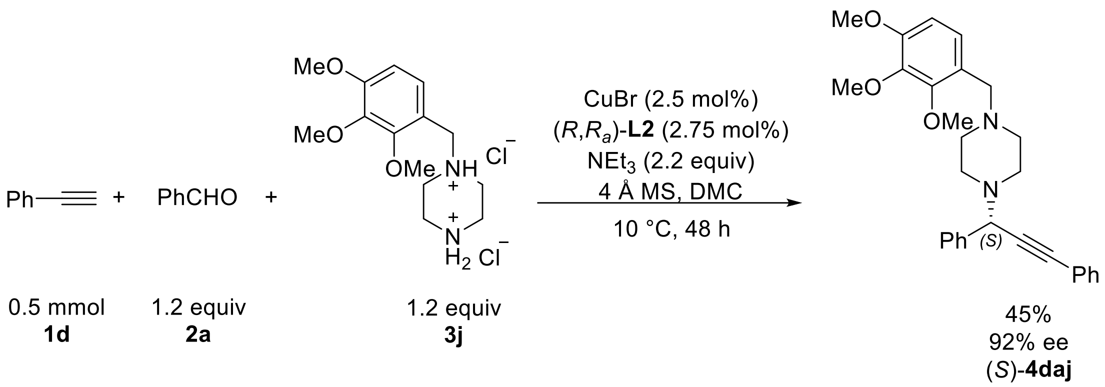

To a flame-dried Schlenk tube were added CuBr (1.8 mg, 0.0125 mmol), (*R*,*R*a)-**L2** (8.1 mg, 0.01375 mmol), 4 Å molecular sieves (151.3 mg), and DMC (0.75 mL) sequentially under Ar atmosphere. After being stirred at room temperature for 30 min, trimetazidine dihydrochloride **3j** (207.9 mg, 0.6 mmol), NEt_3 (113.8 mg, 1.1 mmol), and **1d** (51.1 mg, 0.5 mmol)/DMC (0.5 mL) were added sequentially under Ar atmosphere. The resulting mixture was stirred at 10 °C for another 10 min followed by the addition of **2a** (63.9 mg, 0.6 mmol). After being stirred at 10 °C for 48 h, the reaction was complete as monitored by TLC. The resulting mixture was filtrated through a short pad of basic aluminum oxide (200-300 mesh) eluted with dichloromethane/MeOH (10:1, 22 mL). After evaporation, the residue was purified by chromatography on silica gel (eluent: petroleum ether/ethyl acetate = 2:1 (300 mL) to 1:1 (400 mL)) to afford (*S*)-**4daj** (102.7 mg, 45%) as an oil: 92% ee (HPLC conditions: Chiralcel OJ-H column, hexane/*i*-PrOH = 80/20, 0.7 mL/min, λ = 214 nm, t_R(minor) = 12.8 min, t_R(major) = 23.4 min); $[\alpha]_D^{26}$ = -1.8 (c = 1.00, $CHCl_3$); **^{1}H NMR** (400 MHz, $CDCl_3$) δ 7.61 (d, *J* = 7.2 Hz, 2 H, ArH), 7.52-7.45 (m, 2 H, ArH), 7.37-7.26 (m, 6 H, ArH), 7.00 (d, *J* = 8.4 Hz, 1 H, ArH), 6.62 (d, *J* = 8.8 Hz, 1 H, ArH), 4.79 (s, 1 H, NCH), 3.87 (s, 3 H, OCH_3), 3.86 (s, 3 H, OCH_3), 3.85 (s, 3 H, OCH_3), 3.53 (s, 2 H, NCH_2), 2.67 (br, 4 H, 2×NCH_2), 2.57 (br, 4 H, 2×NCH_2); **^{13}C NMR** (100 MHz, $CDCl_3$) δ 152.7, 152.5, 142.1, 138.1, 131.7, 128.4, 128.1, 128.04, 128.0, 127.5, 125.2, 123.5, 123.0, 106.8, 87.9, 85.6, 61.5, 61., 60.7, 56.1, 55.8, 52.8; **MS** (ESI) *m/z* 457 ($[M+H]^+$); **IR** (neat): ν = 2947, 2904, 1619, 1592, 1541, 1527, 1506, 1468, 1451, 1435, 1410, 1380, 1338, 1310, 1263, 1245, 1229, 1215, 1195, 1172, 1151, 1104, 1088, 1078, 1030, 1010 cm^{-1}; **HRMS** calcd for $C_{29}H_{33}N_2O_3$ ($[M+H]^+$): 457.2486. Found: 457.2484.

57. (*S*)-*N*-methyl-*N*-((S)-3-(naphthyloxy)-3-(thien-2-yl)propyl)-1,3-diphenylprop-2-ynyl amine (*S*,*S*)-**4dak** (xhb-3-083)

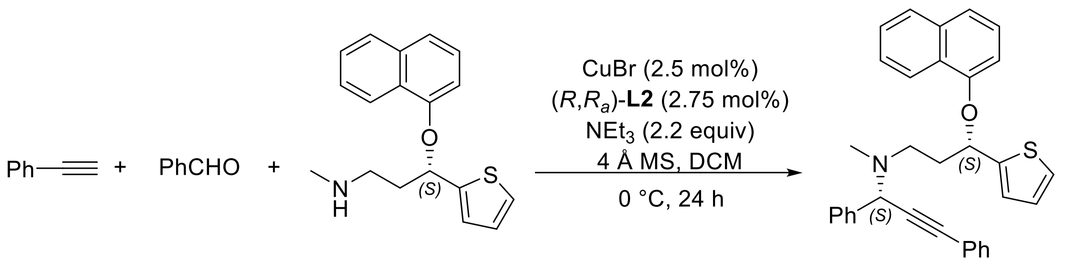

0.5 mmol **1d** 1.2 equiv **2a** 1.2 equiv (*S*)-**3k** 65% 90% de (*S*,*S*)-**4dak**

To a flame-dried Schlenk tube were added CuBr (1.8 mg, 0.0125 mmol), (*R*,*R*a)-**L2** (8.1 mg, 0.01375 mmol), 4 Å molecular sieves (151.3 mg), and DCM (0.75 mL) sequentially under Ar atmosphere. After being stirred at room temperature for 30 min, duloxetine hydrochloride (*S*)-**3k** (204.7 mg, 0.6 mmol), NEt_3 (113.8 mg, 1.1 mmol), and **1d** (51.3 mg, 0.5 mmol)/DCM (0.5 mL) were added sequentially under Ar atmosphere. The resulting mixture was stirred at 0 ºC for another 10 min followed by the addition of **2a** (63.9 mg, 0.6 mmol). After being stirred at 0 ºC for 24 h, the reaction was complete as monitored by TLC. The resulting mixture was filtrated through a short pad of basic aluminum oxide (200-300 mesh) eluted with dichloromethane/MeOH (10:1, 22 mL). After evaporation, the residue was purified by chromatography on silica gel (eluent: petroleum ether/ethyl acetate = 10:1 (440 mL)) to afford (*S*,*S*)-**4dak** (158.5 mg, 65%) as an oil: 90% de (the de value of the product was determined via the NMR analysis); $[\alpha]_D^{27}$ = +31.4 (c = 1.00, $CHCl_3$); **^{1}H NMR** (400 MHz, $CDCl_3$) δ 8.23 (d, *J* = 8.4 Hz, 1 H, ArH), 7.76 (d, *J* = 7.6 Hz, 1 H, ArH), 7.57 (d, *J* = 7.6 Hz, 2 H, ArH), 7.50-7.36 (m, 5 H, ArH), 7.32-7.04 (m, 9 H, ArH), 6.91 (dd, J_1 = 4.8 Hz, J_2 = 3.6 Hz, 1 H, ArH), 6.82 (d, *J* = 7.6 Hz, 1 H, ArH), 5.80 (dd, J_1 = 7.8 Hz, J_2 = 4.8 Hz, 1 H, OCH), 4.94 (s, 1 H, NCH), 2.87-2.74 (m, 2 H, NCH_2), 2.54-2.44 (m, 1 H, one proton of CH_2), 2.29 (s, 3 H, CH_3), 2.26-2.19 (m, 1 H, one proton of CH_2); **^{13}C NMR** (100 MHz, $CDCl_3$) δ 153.5, 145.6, 138.6, 134.6, 131.8, 128.3, 128.2, 128.1, 128.0, 127.3, 126.5, 126.2, 126.1, 125.7, 125.1, 124.6, 124.4, 123.1, 122.2, 120.4, 106.8, 88.4, 84.8, 74.1, 61.0, 50.2, 38.1, 37.3; **MS** (ESI) *m/z* 488 ($[M+H]^+$); **IR** (neat): ν = 3054, 2951, 2845, 2795,

1596, 1577, 1507, 1489, 1461, 1449, 1396, 1322, 1263, 1234, 1193, 1177, 1155, 1123, 1093, 1065, 1017 cm^{-1}; **HRMS** calcd for $C_{33}H_{30}NOS$ ($[M+H]^+$): 488.2043. Found: 488.2044.

58. (*S*,*S*,*R*)-**4dal** (xhb-3-079)

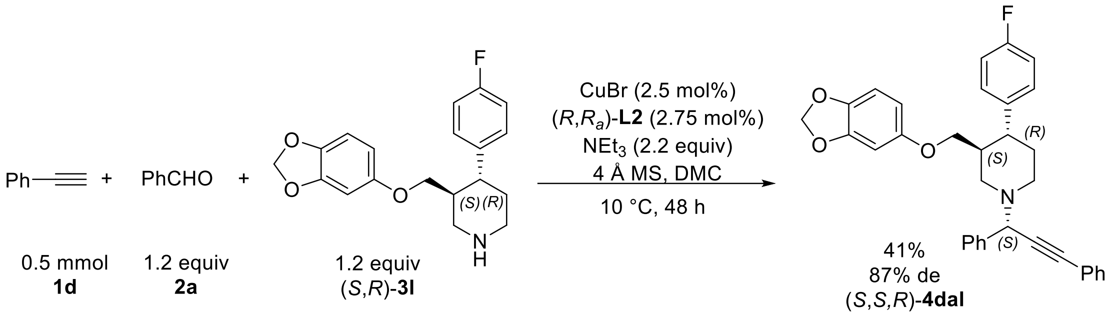

To a flame-dried Schlenk tube were added CuBr (1.8 mg, 0.0125 mmol), (*R*,*R*$_a$)-**L2** (8.1 mg, 0.01375 mmol), 4 Å molecular sieves (152.7 mg), and DMC (0.75 mL) sequentially under Ar atmosphere. After being stirred at room temperature for 30 min, paroxetine hydrochloride (*S*,*R*)-**3l** (224.7 mg, 0.6 mmol), NEt_3 (113.9 mg, 1.1 mmol), and **1d** (51.3 mg, 0.5 mmol)/DMC (0.5 mL) were added sequentially under Ar atmosphere. The resulting mixture was stirred at 10 °C for another 10 min followed by the addition of **2a** (63.7 mg, 0.6 mmol). After being stirred at 10 °C for 48 h, the reaction was complete as monitored by TLC. The resulting mixture was filtrated through a short pad of basic aluminum oxide (200-300 mesh) eluted with dichloromethane/MeOH (10:1, 22 mL). After evaporation, the residue was purified by chromatography on silica gel (eluent: petroleum ether/ethyl acetate = 20:1 (210 mL) to 10:1 (220 mL)) to afford (*S*,*S*,*R*)-**4dal** (107.6 mg, 41%) as an oil: 87% de (the de value of the product was determined via the NMR analysis); $[\alpha]_D^{23}$ = -93.5 (c = 1.00, $CHCl_3$); **^{1}H NMR** (400 MHz, $CDCl_3$) δ 7.69 (d, J = 7.2 Hz, 2 H, ArH), 7.56-7.52 (m, 2 H, ArH), 7.40-7.29 (m, 6 H, ArH), 7.18-7.13 (m, 2 H, ArH), 6.96 (t, J = 8.6 Hz, 2 H, ArH), 6.62 (d, J = 8.4 Hz, 1 H, ArH), 6.35 (d, J = 2.4 Hz, 1 H, ArH), 6.14 (dd, J_1 = 8.6 Hz, J_2 = 2.0 Hz, 1 H, ArH), 5.85 (s, 2 H, ArH), 4.96 (s, 1 H, NCH), 3.60 (dd, J_1 = 9.4 Hz, J_2 = 2.8 Hz, 1 H, one proton of OCH_2), 3.50 (dd, J_1 = 8.6 Hz, J_2 = 8.0 Hz, 1 H, one proton of OCH_2), 3.40 (d, J = 9.6 Hz, 1 H, CH), 2.72 (d, J = 10.8 Hz, 1 H, CH), 2.62 (dd, J_1 = 10.6 Hz, J_2 = 10.4

Hz, 1 H, one proton of CH_2), 2.47-2.40 (m, 1 H, one proton of CH_2), 2.39-2.28 (m, 2 H, NCH_2), 1.79-1.67 (m, 2 H, CH_2); **^{13}C NMR** (100 MHz, $CDCl_3$) δ 161.4 (d, J = 242.4 Hz), 154.3, 148.1, 141.4, 139.8 (d, J = 2.9 Hz), 138.3, 131.8, 128.7 (d, J = 7.6 Hz), 128.4, 128.3, 128.15, 128.12, 127.6, 123.1, 115.3 (d, J = 20.6 Hz), 107.8, 105.4, 101.0, 97.9, 88.3, 85.3, 69.6, 61.9, 56.7, 47.2, 44.3, 42.4, 34.4; **^{19}F NMR** (376 MHz, $CDCl_3$) δ -117.1; **MS** (ESI) *m/z* 520 ($[M+H]^+$); **IR** (neat): ν = 2949, 2921, 2833, 1632, 1601, 1503, 1486, 1466, 1388, 1365, 1269, 1226, 1188, 1159, 1137, 1124, 1103, 1041, 1018 cm^{-1}; **HRMS** calcd for $C_{34}H_{31}FNO_3$ ($[M+H]^+$): 520.2282. Found: 520.2282.

6. Synthesis of (*S*)-(-)-*N*-acetylcolchinol (*S*)-6

1. (*R*)-*N*,*N*-dibenzyl-1-(3-((*tert*-butyldimethylsilyl)oxy)phenyl)-3(3,4,5-trimethoxy-phenyl)prop-2-ynyl amine (*R*)-**4vyi** (Lq-5-176)

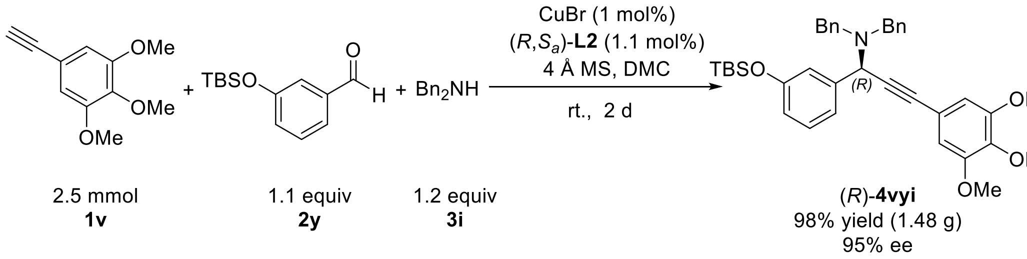

To a flame-dried Schlenk tube were added CuBr (3.7 mg, 0.025 mmol), (*R*,*S*a)-**L2** (16.2 mg, 0.0275 mmol), 4 Å molecular sieves (751.0 mg), and dimethyl carbonate (DMC) (3.75 mL) sequentially under Ar atmosphere. After being stirred at room temperature for 30 min, **1v** (481.0 mg, 2.5 mmol), **2y** (650.4 mg, 2.75 mmol)/DMC (1.25 mL), and **3i** (598.1 mg, 3 mmol)/DMC (1.25 mL) were added sequentially under Ar atmosphere. The resulting mixture was stirred at room temperature for 48 as monitored by TLC. The resulting mixture was filtrated through a short pad of silica gel eluted with dichloromethane/MeOH (10:1, 55 mL). After evaporation, the residue was purified by chromatography on silica gel (eluent: petroleum ether/ether/dichloromethane = 40:1:1 (~200mL) to 20:1:1 (1100 mL)) to afford (*R*)-**4vyi** (1.4845 g, 98%) as an oil: 95% ee (HPLC conditions: Chiralcel AD-H column, hexane/*i*-PrOH = 99/1, 0.5 mL/min, λ = 214 nm, t_R(major) = 53.1 min, t_R(minor) = 43.1

min); $[\alpha]_D^{30}$= +117.5 (c = 1.065, $CHCl_3$)); **^{1}H NMR** (400 MHz, $CDCl_3$) δ 7.50-7.39 (m, 4 H, ArH), 7.37-7.28 (m, 5 H, ArH), 7.28-7.18 (m, 4 H, ArH), 6.82 (s, 2 H, ArH), 6.76 (d, *J* = 8.4 Hz, 1 H, ArH), 4.87 (s, 1 H, NCH), 3.93 (s, 6 H, 2 × OCH_3), 3.90 (s, 3 H, OCH_3), 3.81 (d, *J* = 13.2 Hz, 2 H, NCH_2), 3.54 (d, *J* = 13.6 Hz, 2 H, NCH_2), 0.99 (s, 9 H, 3 × CH_3), 0.21 (s, 6 H, 2 × CH_3); **^{13}C NMR** (100 MHz, $CDCl_3$) δ 155.5, 153.1, 140.7, 139.5, 138.8, 129.0, 128.9, 128.3, 127.0, 121.2, 119.9, 119.3, 118.3, 109.1, 88.3, 83.8, 61.0, 56.2, 55.9, 54.6, 25.7, 18.2, -4.4; **MS** (ESI) *m/z* 608 ($[M+H]^+$); **IR** (neat): ν = 3028, 3001, 2930, 2885, 2856, 2834, 1599, 1576, 1503, 1484, 1463, 1452, 1433, 1409, 1362, 1346, 1278, 1234, 1184, 1168, 1128, 1071, 1053, 1027, 1004 cm^{-1}; **HRMS** calcd for $C_{38}H_{46}NO_4Si$ ($[M+H]^+$): 608.3191, Found: 608.3201.

2. (*S*)-*N*-(1-(3-((*tert*-butyldimethylsilyl)oxy)phenyl)-3-(3,4,5-trimethoxyphenyl)-propyl) acetamide (*S*)-**5** (Lq-5-185, Lq-5-187)

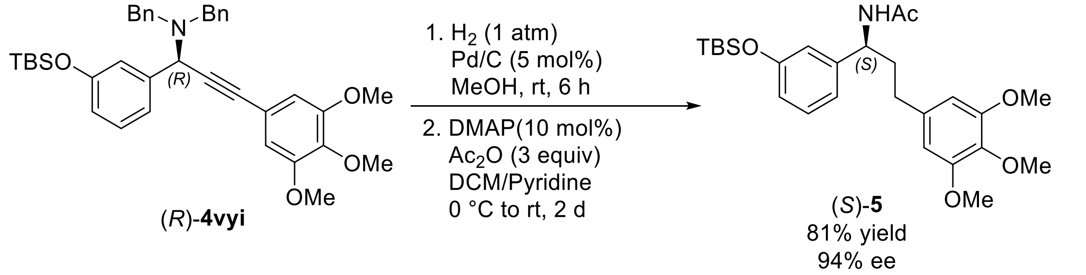

To a Schlenk flask were added (*R*)-**4vyi** (608.1 mg, 1 mmol), Pd/C (53.3 mg, 0.05 mmol, 10% in mass), and MeOH (10 mL) sequentially. The flask was purged three times with hydrogen. The suspension was stirred for 6 h at room temperature under a hydrogen pressure of 1 atm as monitored by TLC. The resulting mixture was filtered through a short column of celite eluted with methanol (50 mL). After evaporation, the crude product was subjected to the next step without further purification and characterization.

To another Schlenk flask were added the above crude product/dichloromethane (2 mL), pyridine (2 mL), and DMAP (12.3 mg, 0.1 mmol) sequentially under Ar atmosphere. After cooling with an ice-water bath for 10 min, Ac_2O (0.28 mL, d = 1.087 g/mL, 304.4 mg, 3 mmol) was added dropwise. The resulting mixture was allowed to warm up to room temperature naturally and stirred at room temperature for 48 h as

monitored by TLC. Ethyl acetate (5 mL) was added. The resulting mixture was washed with a saturated aqueous solution of $CuSO_4$ (3 × 2.5 mL) and a saturated aqueous solution of $NaHCO_3$ (3 × 2.5 mL). The organic layer was dried over anhydrous Na_2SO_4. After filtration and evaporation, the residue was purified by chromatography on silica gel to afford (*S*)-**5**[10,11] (385.8 mg, 81%) (eluent: petroleum ether/ethyl acetate/dichloromethane = 2:1:1 (200 mL) to 1:1:1 (600 mL)) as an oil: 94% ee (HPLC conditions: Chiralcel OD-H column, hexane/*i*-PrOH = 80/20, 0.5 mL/min, λ = 214 nm, t_R(major) = 15.8 min, t_R(minor) = 12.1 min); $[\alpha]_D^{19}$ = -41.8 (c = 1.06, $CHCl_3$) (reported value: 99.6% ee, $[\alpha]_D^{25}$ = -42 (c = 1, $CHCl_3$)[11]; $[\alpha]_D^{25}$ = -35 (c = 1.1, $CHCl_3$)[10]); **^{1}H NMR** (400 MHz, $CDCl_3$) δ 7.21 (t, *J* = 7.8 Hz, 1 H, ArH), 6.89 (d, *J* = 7.6 Hz, 1 H, ArH), 6.81-6.72 (m, 2 H, ArH), 6.36 (s, 2 H, ArH), 5.82 (br, 1 H, NH), 4.97 (dd, J_1 = 15.2 Hz, J_2 = 7.6 Hz, 1 H, NCH), 3.83 (s, 6 H, 2 × OCH_3), 3.82 (s, 3 H, OCH_3), 2.64-2.43 (m, 2 H, CH_2), 2.24-2.11 (m, 1 H, one proton of CH_2), 2.10-2.00 (m, 1 H, one proton of CH_2), 1.97 (s, 3 H, CH_3), 0.98 (s, 9 H, 3 × CH_3), 0.20 (s, 6 H, 2 × CH_3); **^{13}C NMR** (100 MHz, $CDCl_3$) δ 169.2, 155.9, 153.0, 143.3, 137.1, 136.0, 129.7, 119.5, 119.1, 118.5, 105.1, 60.8, 55.9, 53.1, 37.4, 32.9, 25.6, 23.4, 18.1, -4.4; **MS** (ESI) *m/z* 474 ($[M+H]^+$); **IR** (neat): ν = 3312, 2952, 2933, 2857, 2838, 1648, 1591, 1545, 1504, 1484, 1462, 1422, 1372, 1332, 1276, 1245, 1236, 1182, 1156, 1124, 1096, 1063, 1036, 1004 cm^{-1}.

3. (*S*)-(-)-*N*-acetylcolchinol (*S*)-**6** (Lq-5-189)[12]

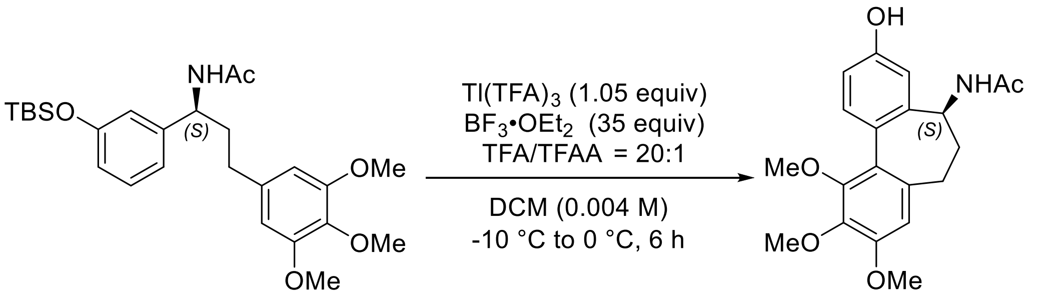

(*S*)-**5**
81% yield
94% ee

(*S*)-**6**
50% yield, 95% ee
after MeOH/H_2O recrystallization
47% yield, 97% ee

To a Schlenk flask were added $Tl(TFA)_3$ (414.7 mg, 0.7245 mmol), TFA(120 mL),

and TFAA (6 mL) sequentially under Ar atmosphere. After being stirred at -10 ºC for 10 min, a solution of (*S*)-**5** (326.0 mg, 0.69 mmol, 94% ee) in CH_2Cl_2 (10 mL) and $BF_3{\cdot}OEt_2$ (3 mL, d = 1.15 g/mL, 3.45 g, 24.15 mmol) were added. The resulting mixture was allowed to warm up to 0 ºC naturally and stirred for 6 h as monitored by TLC. Most of the solvent was removed under reduced pressure followed by the addition of H_2O (20 mL). The resulting mixture was extracted with dichoromethane (3 × 20 mL). The combined organic layer was dried over anhydrous Na_2SO_4. After filtration and evaporation, the residue was purified by chromatography on silica gel (eluent: ethyl acetate) to afford (*S*)-**6**[10,11,13-16] (122.2 mg, 50%) (eluent: ethyl acetate 1000 mL) as an off-white solid: 95% ee (HPLC conditions: Chiralcel OD-H column, hexane/*i*-PrOH = 80/20, 0.4 mL/min, λ = 214 nm, t_R(major) = 16.8 min, t_R(minor) = 12.8 min); Recrystallization from MeOH and H_2O afforded (*S*)-**6** (114.9 mg, 47%, 97% ee) as a white solid (HPLC conditions: Chiralcel OD-H column, hexane/*i*-PrOH = 80/20, 1.0 mL/min, λ = 214 nm, t_R(major) = 6.5 min, t_R(minor) = 5.0 min); m.p. 150-152 ºC (MeOH/H_2O) (reported value: m.p. 152-154 ºC (EtOH/H_2O);[15] m.p. 150 ºC (EtOH)[16]); $[\alpha]_D^{27}$ = -37.5 (c = 0.99, $CHCl_3$) (reported value: 94% ee, $[\alpha]_D^{27}$ = -34.0 (c = 1, $CHCl_3$)[16]; $[\alpha]_D^{25}$ = -45.2 (c = 0.6, $CHCl_3$)[10]; $[\alpha]_D^{20}$ = -51.6 (c = 1.123, $CHCl_3$)[13]; $[\alpha]_D^{20}$ = -55.8 (c = 0.135, $CHCl_3$)[14]); **^{1}H NMR** (400 MHz, CD_3OD) δ 7.23 (d, *J* = 8.4 Hz, 1 H, ArH), 6.79 (d, *J* = 2.4 Hz, 1 H, ArH), 6.72 (dd, J_1 = 8.4 Hz, J_2 = 2.4 Hz, 1 H, ArH), 6.70 (s, 1 H, ArH), 4.61 (dd, J_1 = 12.2 Hz, J_2 = 5.8 Hz, 1 H, NCH), 3.86 (s, 3 H, OCH_3), 3.85 (s, 3 H, OCH_3), 3.49 (s, 3 H, OCH_3), 2.56-2.42 (m, 1 H, one proton of CH_2), 2.33-2.19 (m, 2 H, two protons of 2 × CH_2), 2.00 (s, 3 H, CH_3), 1.97-1.86 (m, 1 H, one proton of CH_2); **^{13}C NMR** (100 MHz, CD_3OD) δ 173.3, 158.8, 154.6, 153.0, 143.3, 143.2, 137.5, 133.0, 127.6, 127.4, 115.1, 111.7, 109.9, 62.5, 62.2, 57.5, 51.4, 40.7, 32.4, 23.5; **MS** (ESI) *m/z* 380 ($[M+Na]^+$), 375 ($[M+NH_4]^+$), 358 ($[M+H]^+$); **IR** (neat): ν = 3302, 2928, 1647, 1581, 1550, 1485, 1453, 1403, 1376, 1351, 1326, 1277, 1238, 1197, 1143, 1101, 1049, 1010, 821, 730 cm^{-1}.

7. Synthetic transformations

1. (*R*,*E*)-4-Phenyl-4-(pyrrolidin-1-yl)but-2-en-1-ol (*R*,*E*)-**7** (xhb-1-145)[17]

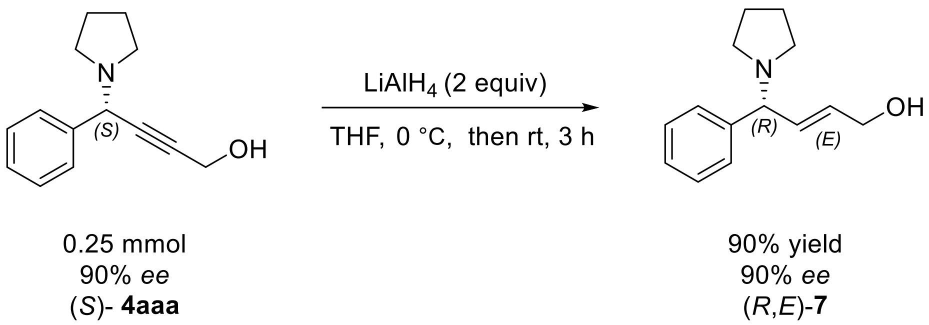

To a flask was added (*S*)-**4aaa** (58.0 mg, 0.27 mmol). Then the flask was evacuated and back-filled with Ar for three times. THF(1.4 mL) was added and the resulting solution was cooled with an ice-water bath. Lithium aluminum hydride (21.2 mg, 0.54 mmol) was added slowly in portions. Then the mixture was allowed to warm up to room temperature naturally and stirred at room temperature for 3 h as monitored by TLC. After completion of reaction, the mixture was cooled by ice-water bath. Then 0.1 mL of H_2O, 0.1 mL of 10% NaOH solution in H_2O, and 0.3 mL of H_2O were slowly added to the solution sequentially (with generation of H_2). The resulting mixture was dried over anhydrous Na_2SO_4, filtered through a short pad of celite eluted with ethyl acetate (25 mL), and concentrated under reduced pressure. The crude residue was purified by chromatography on silica gel (eluent: dichloromethane/methanol= 40:1 (~80 mL) to 20:1 (~100 mL) to 10:1 (550 mL)) to afford (*R*,*E*)-**7** (52.8 mg, 90%) as a yellow liquid: 90% ee (HPLC conditions: OD-H column, hexane/iPrOH= 98/2, 1.0 mL/min, λ = 214 nm, t_R (minor) = 17.7 min, t_R (major) = 19.6 min); $[\alpha]_D^{25}$= -48.7 (c = 1.00, $CHCl_3$); **^{1}H NMR** (400 MHz, $CDCl_3$) δ 7.36 (d, *J* = 8.0 Hz, 2 H, ArH), 7.30 (t, *J* = 7.4 Hz, 2 H, ArH), 7.23 (t, *J* = 7.2 Hz, 1 H, ArH), 5.96 (dd, J_1 = 15.4 Hz, J_2 = 8.8 Hz, 1 H, =CH), 5.81 (dt, J_1 = 15.2 Hz, J_2 = 5.4 Hz, 1 H, =CH), 4.07 (d, *J* = 5.6 Hz, 2 H, OCH_2), 3.65 (d, *J* = 8.4 Hz, 1 H, NCH), 3.12 (br, 1 H, OH), 2.62– 2.48 (m, 2 H, NCH_2), 2.45– 2.34 (m, 2 H, NCH_2), 1.82– 1.67 (m, 4 H, 2 × CH_2); **^{13}C NMR** (100 MHz, $CDCl_3$) δ 142.3, 133.5, 130.6, 128.5, 127.6, 127.2, 73.7, 62.7, 53.0, 23.2; **MS** (ESI) *m/z* 218 ($[M+H]^+$); **IR** (neat): ν =3329, 2965, 2799, 1494, 1453, 1362, 1275, 1196, 1097, 1012

cm^{-1}; **HRMS** calcd for$C_{14}H_{20}NO$ ([M+H]$^+$): 218.1539. Found: 218.1546.

2. (*R*,*Z*)-4-phenyl-4-(pyrrolidin-1-yl)but-2-en-1-ol (*R*,*Z*)-**7** (xhb-1-151)[18]

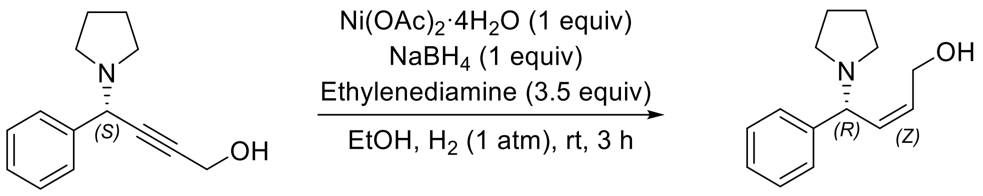

0.25 mmol
90% ee
(*S*)-**4aaa**

92% yield
95% ee
(*R*,*Z*)-**7**

The flame-dried flask was evacuated and back-filled with Ar for three times. $Ni(OAc)_2·4H_2O$ (62.2 mg, 0.25 mmol), EtOH (0.3 mL), and $NaBH_4$ (10.0 mg, 0.25 mmol) were added. Then the flask was purged three times with H_2. After being stirred for 1 h at room temperature under H_2 atmosphere (1 atm), a solution of (*S*)-**4aaa** (53.8 mg, 0.25 mmol) and ethylenediamine (0.06 mL, d = 0.899 g/mL, 0.0539 g, 0.875 mmol) in EtOH (0.7 mL) was added. After being stirred at room temperature under H_2 atmosphere (1 atm) for additional 3 h as monitored by TLC, the reaction was complete and the resulting mixture was filtered through a short pad of celite eluted with ethyl acetate (25 mL) and concentrated under reduced pressure. The crude residue was purified by chromatography on silica gel (eluent: dichloromethane/methanol= 40:1 (~120 mL) to 20:1 (~ 100 mL) to 10:1 (440 mL)) to afford (*R*,*Z*)-**7** (50.0 mg, 92%) as a yellow liquid: 95% ee (HPLC conditions: OJ-H column, hexane/iPrOH= 95/5, 0.5 mL/min, λ = 214 nm, t_R (major) = 17.3 min, t_R (minor) = 21.1 min); $[\alpha]_D^{26}$ = -15.9 (c = 1.00, $CHCl_3$); **^{1}H NMR** (400 MHz, $CDCl_3$) δ 7.38 (d, *J* = 7.6 Hz, 2 H, ArH), 7.32 (t, *J* = 7.4 Hz, 2 H, ArH), 7.25 (t, *J* = 7.2 Hz, 1 H, ArH), 5.83 (dd, J_1 = 11.6 Hz, J_2 = 8.0 Hz, 1 H, =CH), 5.67 (dt, J_1 = 11.6 Hz, J_2 = 5.8 Hz, 1 H, =CH), 4.38 (dd, J_1 = 14.6 Hz, J_2 = 5.6 Hz, 1 H, one proton from OCH_2), 4.26 (dd, J_1 = 14.4 Hz, J_2 = 5.6 Hz, 1 H, one proton from OCH_2), 4.18-3.59 (br, 1 H, OH), 4.05 (d, *J* = 8.0 Hz, 1 H, NCH), 2.63-2.52 (m, 2 H, NCH_2), 2.44-2.35 (m, 2 H, NCH_2), 1.84-1.70 (m, 4 H, 2 × CH_2); **^{13}C NMR** (100 MHz, $CDCl_3$) δ 141.4, 133.0, 129.8, 128.4, 128.1, 127.2, 67.4, 59.3, 52.1, 23.0; **MS** (ESI) *m/z* 218 ([M+H]$^+$); **IR** (neat): ν =3342, 2965, 2929, 2875, 2801, 1490, 1453,

1294, 1266, 1196, 1129, 1030 cm^{-1}; **HRMS** calcd for$C_{14}H_{20}NO$ ($[M+H]^+$): 218.1539. Found: 218.1544.

3. (*S*)-2-(4-phenyl-4-(pyrrolidin-1-yl)but-2-yn-1-yl)isoindoline-1,3-dione (*S*)-**8** (xhb-1-174)[19,20]

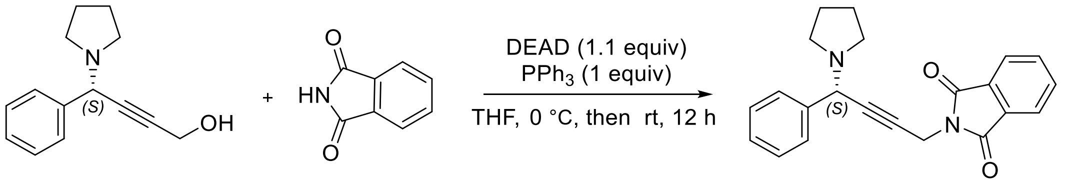

0.5 mmol
91% ee
(*S*)-**4aaa**

1.0 equiv

75% yield
91% ee
(*S*)-**8**

The flame-dried Schlenk tube was evacuated and refilled with Ar for three times. (*S*)-**4aaa** (108.1 mg, 0.5 mmol)/THF (1.0 mL), phenylimide (75.5 mg, 0.5 mmol), and PPh_3 (132.0 mg, 0.5 mmol) were added. After being cooled with an ice-water bath, DEAD (98.7 mg, 0.55 mmol) was added dropwise. Then the mixture was allowed to warm up to room temperature naturally and stirred at room temperature for 12 h as monitored by TLC. The resulting solution was concentrated under reduced pressure, then 1 mL of cold Et_2O was added to the residue. The resulting mixture was filtered and washed with cold Et_2O (3 × 0.5 mL). The filtrate was concentrated under reduced pressure and purified by chromatography on silica gel (eluent: petroleum ether/ethyl acetate = 4:1 (250 mL) to 2:1 (600 mL)) to afford 216.7 mg of impure (*S*)-**8**, which was further purified via recrystallization (petroleum ether/ethyl acetate) to remove the solid impurity. The filtrate was concentrated under reduced pressure, the residue was recrystallized from petroleum ether/ethyl acetate and placed in a refrigerator (approximate -26 ºC) for further precipitation to afford (*S*)-**8** (129.2 mg, 75%) as a white solid: 91% ee (HPLC conditions: OD-H column, hexane/iPrOH= 98/2, 0.7 mL/min, λ = 214 nm, t_R (minor) = 23.3 min, t_R (major) = 25.5 min); $[\alpha]_D^{28}$= -28.4 (c = 1.00, $CHCl_3$); m.p. 96.0-97.0 ºC (petroleum ether/ethyl acetate); **^{1}H NMR** (400 MHz, $CDCl_3$) δ 7.88 (dd, J_1 = 5.2 Hz, J_2 = 3.2 Hz, 2 H, ArH), 7.73 (dd, J_1 = 5.0 Hz, J_2 = 3.2 Hz, 2 H, ArH), 7.48 (d, *J* = 7.6 Hz, 2 H, ArH), 7.31 (t, *J* = 7.4 Hz, 2 H, ArH), 7.25 (d, *J* = 7.2 Hz, 1 H,

ArH), 4.63 (s, 1 H, NCH), 4.57 (s, 2 H, NCH_2), 2.63– 2.47 (m, 4 H, 2×NCH_2), 1.78–1.65 (m, 4 H, 2 × CH_2); **^{13}C NMR** (100 MHz, $CDCl_3$) δ 167.0, 138.9, 134.1, 132.0, 128.13, 128.11, 127.4, 123.4, 80.4, 79.9, 58.4, 50.1, 27.4, 23.3; **MS** (ESI) *m/z* 345 ($[M+H]^+$); **IR** (neat): ν =2965, 2811, 1772, 1706, 1465, 1453, 1423, 1394, 1342, 1320, 1265, 1190, 1115, 1089, 1072 cm^{-1}; Anal.Calcd.for $C_{22}H_{20}N_2O_2$: C 76.72, H 5.85, N8.13; Found: C 76.60, H 5.94, N 8.05.

4. (*R*)-4-cyclohexyl-2,3-butadien-1-ol (*R*)-**9** (Lq-5-134, Lq-5-137-2)[21,22]

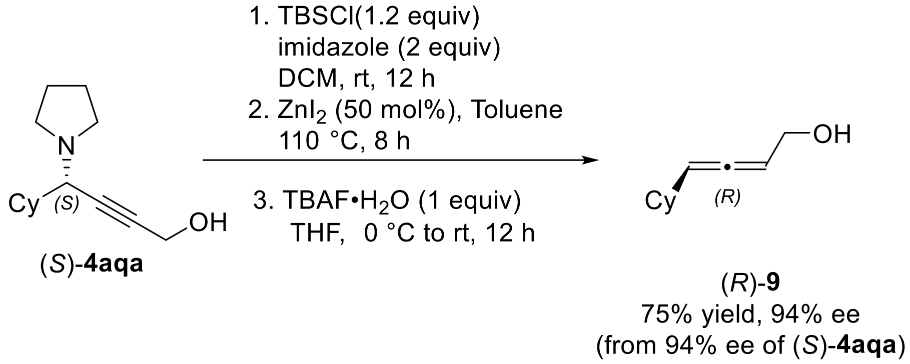

To a flask were added (*S*)-**4aqa** (225.9 mg, 1 mmol, 94% ee)/dichloromethane (3.5 mL), imidazole (139.5 mg, 2 mmol), and TBSCl (184.6 mg, 1.2 mmol). The resulting solution was stirred for 12 h at room temperature as monitored by TLC. The resulting mixture was diluted with H_2O (4 mL). The organic layer was separated and the aqueous layer was extracted with dichloromethane (3 × 4 mL).The combined organic layer was dried over anhydrous Na_2SO_4. After filtration and evaporation, the residue was used in the next step without further purification.

To a dried Schlenk tube was added ZnI_2(162.3 mg, 0.5 mmol) under Ar atmosphere. The above crude product was then dissolved in toluene (1.5 mL) and transferred to the Schlenk tube via a syringe. The residue in the flask was dissolved with 1.5 mL of toluene and the solution was also added to Schlenk tube via a syringe. The Schlenk tube was then placed in an oil bath pre-heated at 110 °C for 8 h as monitored by TLC. After cooling to room temperature, the crude reaction mixture was filtered through a short pad of celite eluted with ether (30 mL). After evaporation, the residue was directly dissolved in THF (3 mL) and treated with TBAF·$3H_2O$ (315.7 mg, 1mmol) at 0 °C. The resulting mixture was allowed to warm up to room temperature naturally with stirring.

After 12 h, the reaction was complete as monitored by TLC, ether (10 mL) and H_2O (10 mL) were then added. The organic layer was separated, and the aqueous layer was extracted with ether (2 × 10 mL). The combined organic layer was dried over anhydrous Na_2SO_4. After filtration and evaporation, the residue was purified by chromatography on silica gel to afford (*R*)-**9**[21](114.1 mg, 75%) (eluent: petroleum ether/ethyl acetate = 20:1 (840 mL) to 10:1 (440 mL)) as a liquid: 94% ee (HPLC conditions: Chiralcel AS-H column, hexane/*i*-PrOH = 98/2, 1.0 mL/min, λ = 214 nm, t_R(major) = 10.4 min, t_R(minor) = 12.7 min); $[\alpha]_D^{31}$ = -93.4 (c = 1.055, $CHCl_3$) (reported value: 99% ee, $[\alpha]_D^{22}$= -99.8 (c = 1.01, $CHCl_3$)[21]); **^{1}H NMR** (400 MHz, $CDCl_3$) δ 5.41-5.32 (m, 1 H, one proton of HC=C=CH), 5.32-5.25 (m, 1 H, one proton of HC=C=CH), 4.10 (dd, J_1 = 6.0 Hz, J_2 = 2.8 Hz, 2 H, CH_2O), 2.08-1.94 (m, 1 H, CH), 1.84-1.57 (m, 6 H), 1.37-0.99 (m, 5 H); **^{13}C NMR** (100 MHz, $CDCl_3$) δ 201.8, 100.0, 92.6, 60.8, 37.0, 33.02, 32.96, 26.0, 25.9; **MS** (EI) *m/z* 152 (M^+, 1.78); **IR** (neat): ν = 3311, 2921, 2849, 1960, 1447, 1416, 1348, 1301, 1258, 1212, 1181, 1123, 1062, 1047, 1007 cm^{-1}.

8. Determination of the rotation barrier for L2

(*R*,*S*a)-**L2** (10.8 mg) was dissolved in anhydrous toluene (5 mL) under Ar atmosphere and stirred at 100 °C for 3 d. Each time, 0.1 mL of the reaction mixture was taken out and evaporated to dryness at room temperature under vacuum. The ratios of diastereoisomers (*R*,*S*a)-**L2** and (*R*,*R*a)-**L2** were determined by HPLC (Chiralpak IC column, hexane/*i*-PrOH = 70/30, 0.7 mL/min, λ = 214 nm, t_R[(*R*,*S*a)-**L2**] = 13.2 min, t_R[(*R*,*R*a)-**L2**] = 22.7 min).[23]

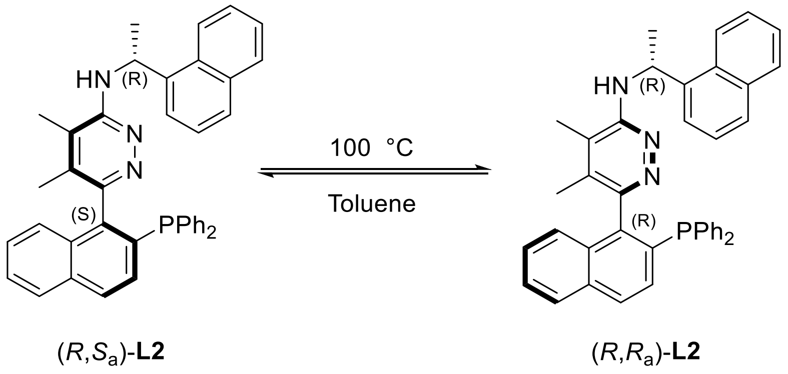

Supplementary Table 1. Monitoring the de (%) for (R,S_a)-**L2** over time

entry	time (min)	(R,S_a)-**L2** (%)	de (%)
1	0	100	100
2	30	99.27	98.54
3	60	98.32	96.64
4	120	96.13	92.26
5	180	94.35	88.7
6	301	90.83	81.66
7	371	88.75	77.5
8	454	86.26	72.52
9	710	79.2	58.4
10	945	74.11	48.22
11	1106	71.31	42.62
12	1267	68.74	37.48
13	1428	66.27	32.54
14	1555	64.88	29.76
15	1700	63.24	26.48
16	1904	61.21	22.42
17	2495	56.67	13.34
18	2677	55.82	11.64
19	2903	54.88	9.76
20	4459	50.56	1.12

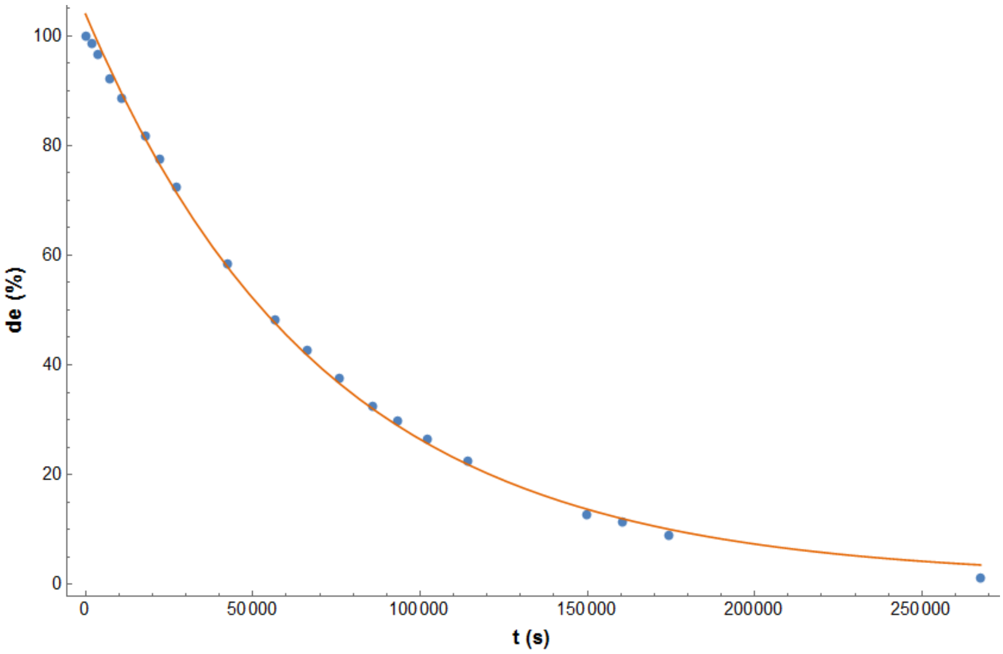

Supplementary Figure 4. Plot of de (%) vs Time (s) at 100 °C for (*R*,*S*a)-L2. The rotational barrier between (*R*,*S*a)-**L2** and (*S*,*S*a)-**L2** was studied.

According to the corresponding kinetics equation for equilibrium:

$$\ln\left(\frac{A_0 - A_{\mathrm{eq}}}{A_t - A_{\mathrm{eq}}}\right) = (k_f + k_r)t$$

The k_f and k_r are the rate constants for the forward and reverse reactions, respectively. The above experimental data (Supplementary Table 1, Entry 20; t = 4459 min, de [(*R*,*S*a)-**L2**)] = 1.12) suggested:

$$A_{eq} = 0.5\ A_0$$

$$k_f = k_r$$

Then we plotted this equation:

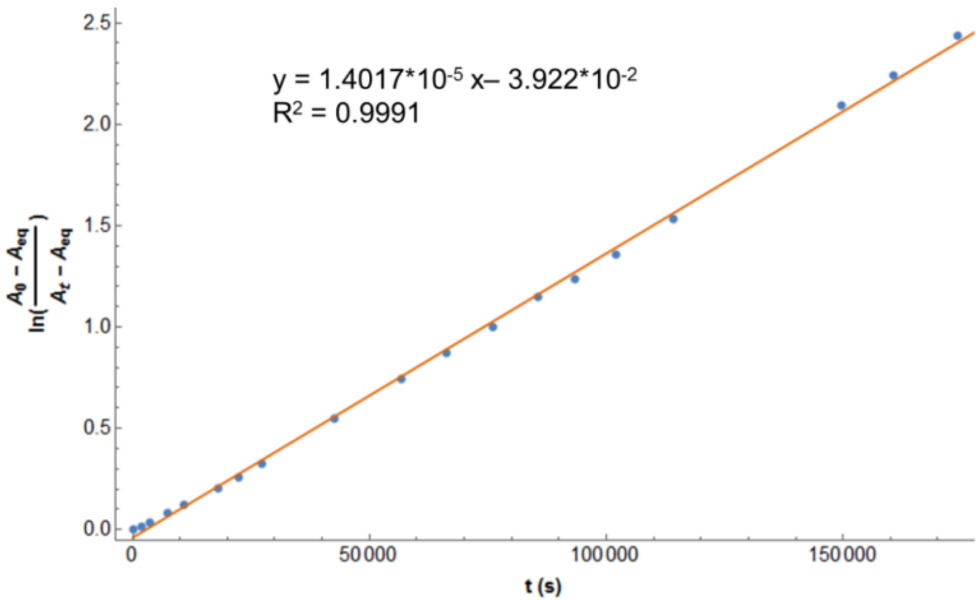

$$\text{Slope} = k_f + k_r = 2k = 1.4017 \times 10^{-5}\ \text{s}^{-1}$$

The rotation barrier was obtained from the Eyring equation:

$$\Delta G_T^{\ddagger} = -RT\ln\left(\frac{k \times h}{k_B \times T}\right)$$

$$(R = 8.31451\ \text{J·K}^{-1}\text{·mol}^{-1},\ h = 6.62697 \times 10^{-34}\ \text{J·s},\ k_B = 1.3807 \times 10^{-23}\ \text{J·K}^{-1})$$

$$\Delta G_{100\ °C}^{\ddagger} = 129.0\ \text{kJ·mol}^{-1} = 30.8\ \text{kcal·mol}^{-1}$$

9. The effect of the ee of the ligand on the ee of (*S*)-4aqa

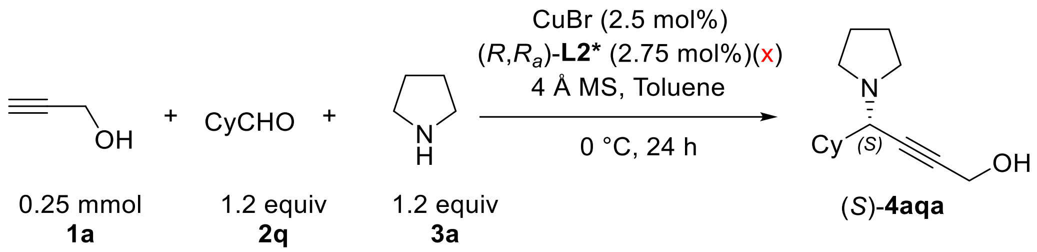

entry	x (ee of (*R*,*R*a)-L2*)[a]	yield[b]	ee[c]
1	25.7	94	29.4
2	51.7	98	51.4
3	75.6	99	73.7
4[d]	100	99	94

[a] ee values determined by chiral HPLC analysis (Chiralpak IC column, hexane/*i*-PrOH

= 80/20, 1.0 mL/min, λ = 214 nm, t_R[(*S*,*S*a)-**L2**] = 23.9 min, t_R[(*R*,*R*a)-**L2**] = 29.8 min). [b]Isolated yield. [c]ee values determined by chiral HPLC analysis (Chiralcel AD-H column, hexane/*i*-PrOH = 95/5, 0.8 mL/min, λ = 214 nm, t_R(major) = 8.0 min, t_R(minor) = 6.7 min). [d]The reaction was conducted on 0.5 mmol scale.

10. ^{31}P-NMR experiments

To a flame-dried flask were added CuBr (1.9 mg,0.013 mmol), (*R*,*R*a)-**L2** (7.6 mg, 0.013 mmol), and d_6-toluene (0.5 mL) sequentially inside a glovebox. After being stirred at room temperature for 30 min, the resulting mixture was transferred to a dried NMR tube.

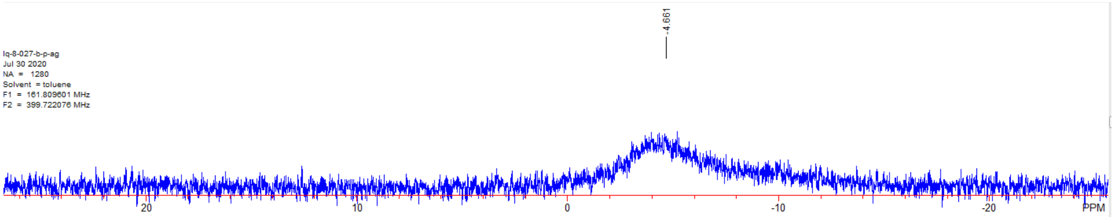

Supplementary Figure 5. ^{31}P-NMR spectrum (162 MHz, 298 K, d_6-toluene) of the resulting mixture showing the signal at -4.7 ppm.

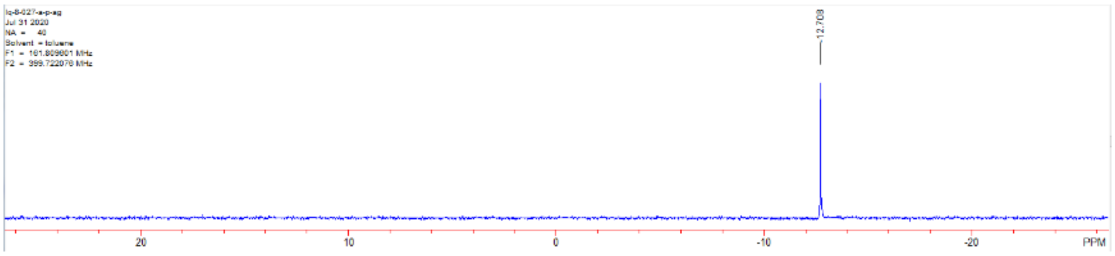

Supplementary Figure 6. ^{31}P-NMR spectrum (162 MHz, 298 K, d_6-toluene) of (*R*,*R*a)-**L2** showing the signal at -12.7 ppm.

11. SAESI-MS experiments

SAESI-MS conditions

SAESI-MS spectra were recorded on a Thermo TSQ Quantum Access triple-quadrupole mass spectrometer (Thermo Fisher Scientific, Waltham, MA) equipped with a home-made SAESI ion source in positive mode. The basic SAESI conditions were: vacuum, 2.8×10^{-6} torr; spray voltage, 4000 V; capillary temperature, 275 °C; sheath gas pressure of two sprayers, 3 arb. units; the collision energy of CID, 20 eV. Data acquisition and analysis were done with the Xcalibur (version 2.0, Thermo Fisher Scientific) software package.

In solvent-assisted electrospray ionization mass spectrometric experiment, the angle (α) between the two sprayers is 45° and the distance (b) between the tip of sprayers and the inlet to the mass is 6 mm. The chemical solutions were injected by a 500-μL air-tight syringe with a speed at 5 μL/min to SAESI-MS. The assisted solvent of methanol was injected by another 500-μL air-tight syringe with a speed at 5 μL/min.[24]

SAESI-MS device

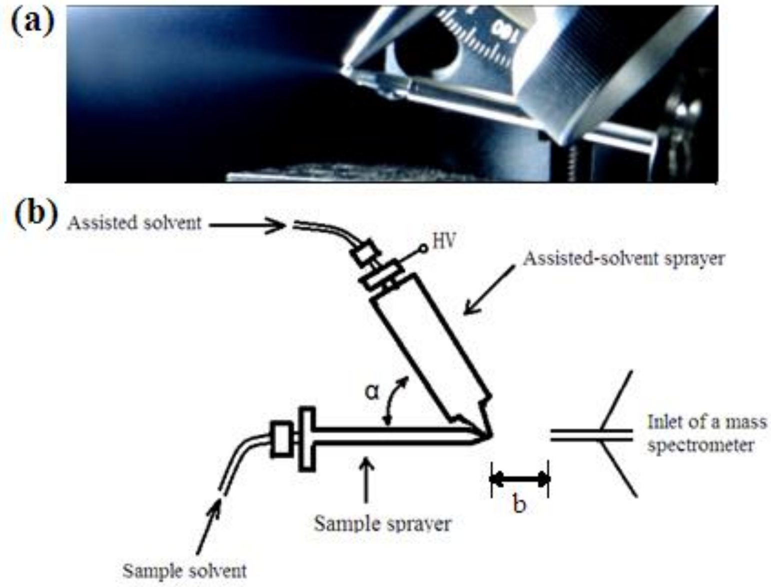

Supplementary Figure 7. (a) Photographic image of the SAESI apparatus. (b)

Schematic representation of the SAESI. The angle (α) between the two sprayers is 45°and the distance (b) between the tip of sprayers and the inlet to the mass is 6 mm.

Mass spectrometric experimental results

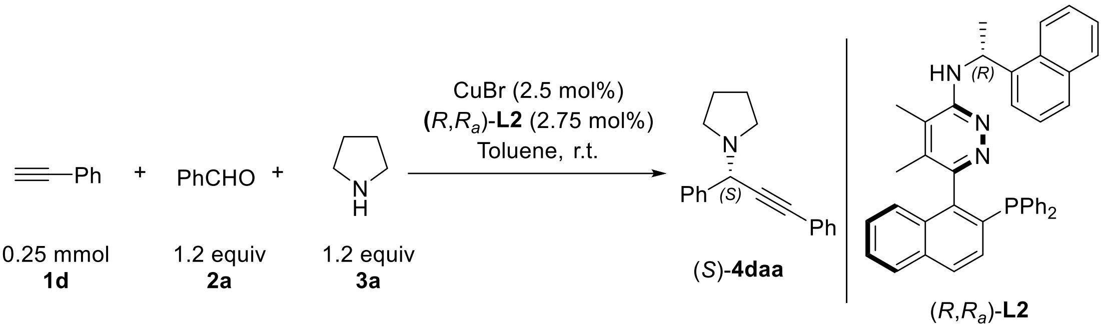

1. To a flame-dried Schlenk tube were added CuBr (0.9 mg, 0.00625 mmol), (*R*,*R*a)-**L2** (4.0 mg, 0.006875 mmol), and toluene (1 mL) sequentially under Ar atmosphere. The resulting mixture was stirred at room temperature for 30 min.

2. Then alkyne **1d** (27.5 μL, d = 0.93 g/mL, 25.5 mg, 0.25 mmol) was added and the resulting mixture was stirred at room temperature for 10 min. This resulting mixture (50 μL) was dissolved in 2 mL of CH_3CN for SAESI-MS study.

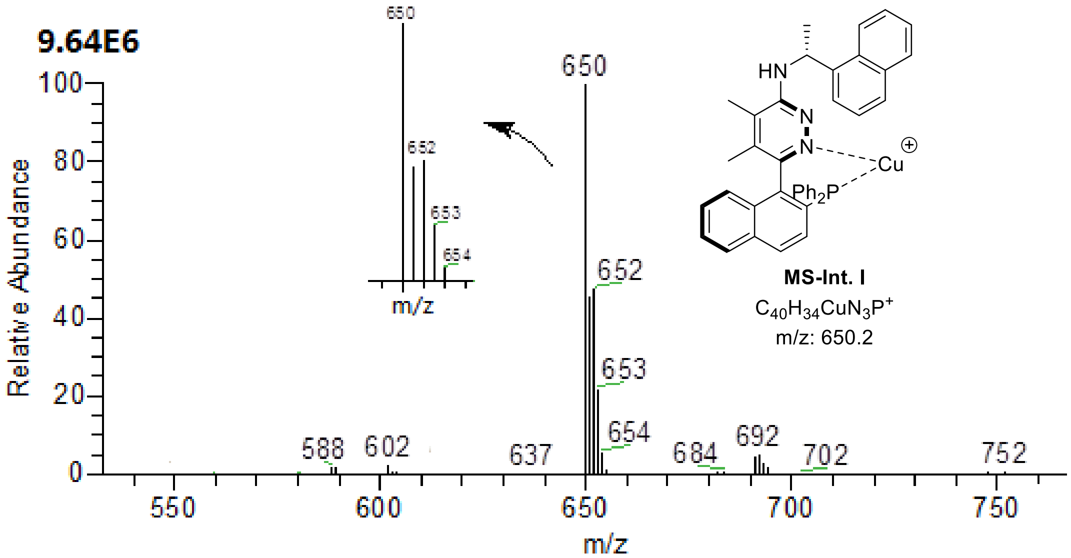

Supplementary Figure 8. Expanded SAESI-MS spectrum, showing the signal from *m/z* 540 to 760.

SAESI-MS/MS experiment was conducted to identify the signal at *m*/*z* 752:

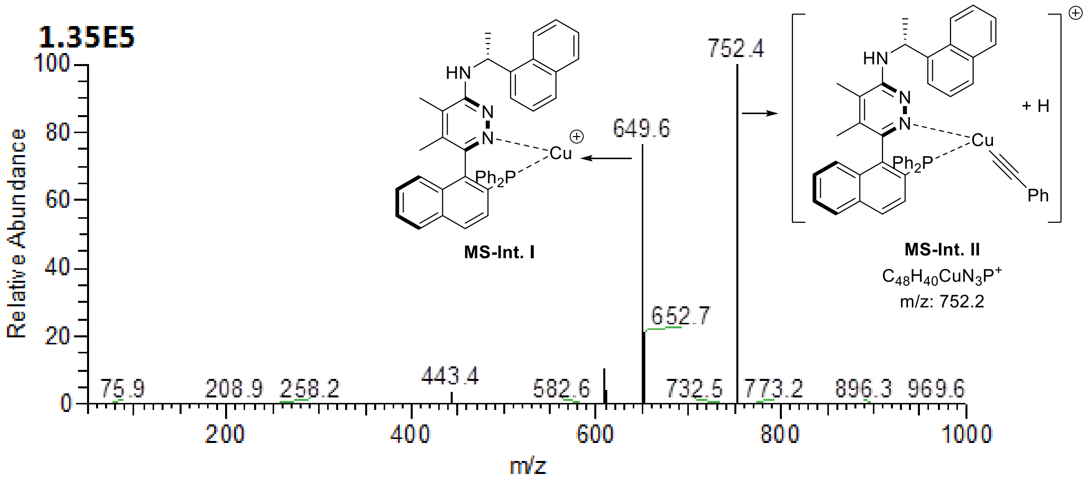

Supplementary Figure 9. SAESI-MS/MS spectrum of the solution showing the signal of **MS-Int. II** at *m*/*z* 752.

3. Later, aldehyde **2a** (30.5 µL, d = 1.044 g/mL, 31.8 mg, 0.3 mmol) and pyrrolidine **3a** (24.5 µL, d = 0.866 g/mL, 21.3 mg, 0.3 mmol) were added to the remaining mixture. This resulting mixture (50 µL) was dissolved in 2 mL of CH_3CN for SAESI-MS study.

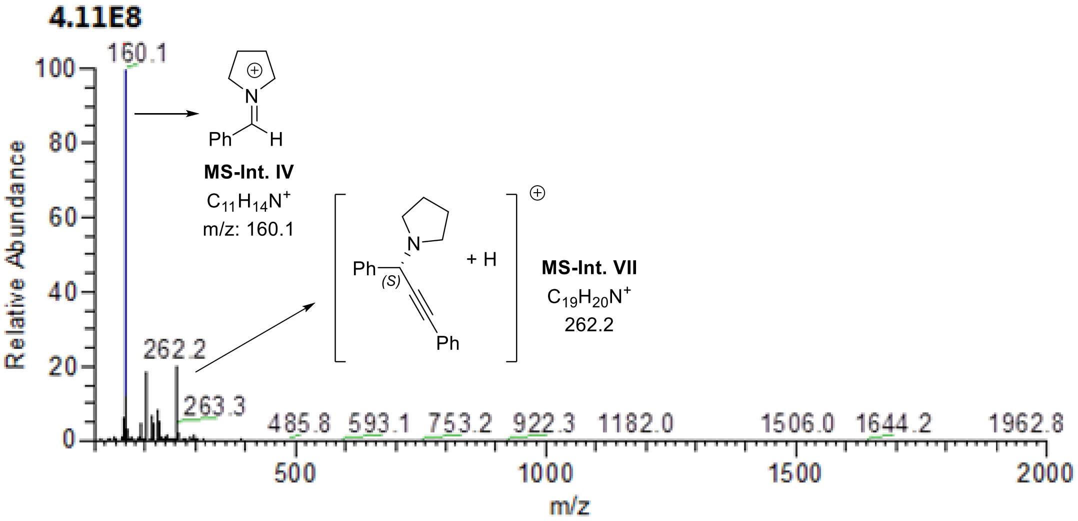

Supplementary Figure 10. SAESI-MS spectrum of the solution showing the signals of **MS-Int. VII** at *m*/*z* 262 and **MS-Int. IV** at *m*/*z* 160.

SAESI-MS/MS experiment was conducted to identify the signals at *m*/*z* 178, 929, 991, 262:

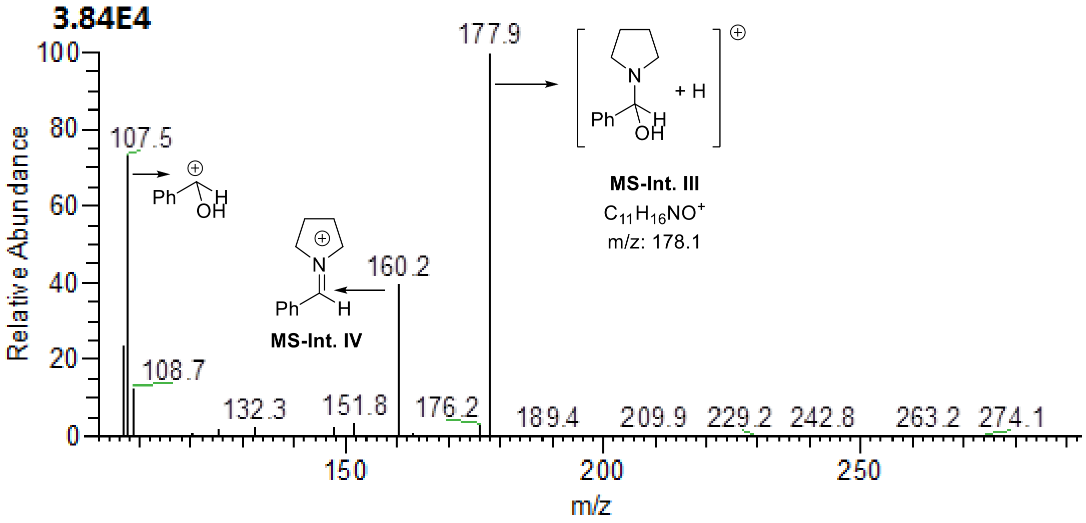

Supplementary Figure 11. SAESI-MS/MS spectrum of the solution showing the signal of **MS-Int. III** at *m*/*z* 178.

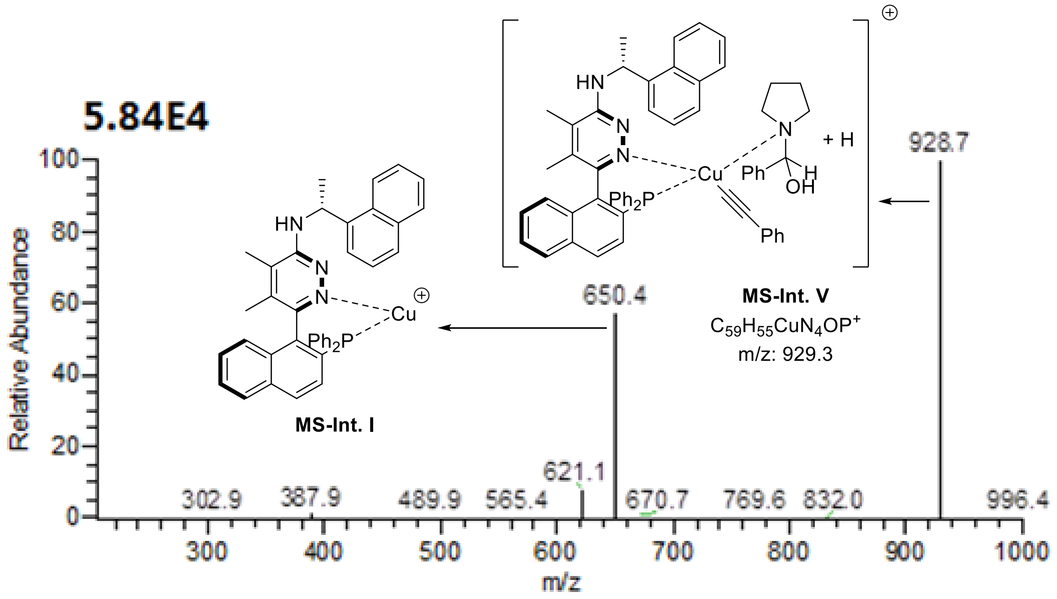

Supplementary Figure 12. SAESI-MS/MS spectrum of the solution showing the signal of **MS-Int. V** at *m*/*z* 929.

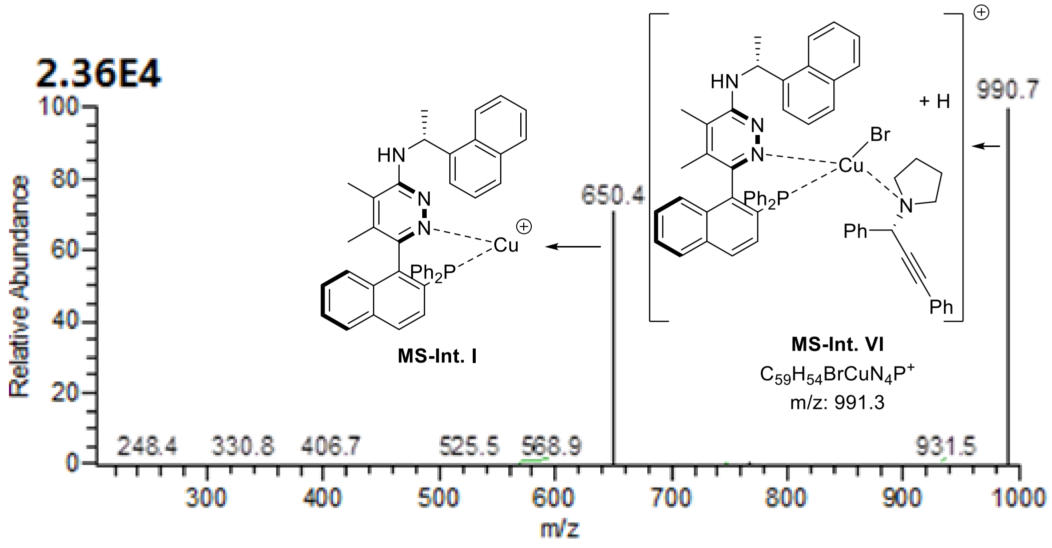

Supplementary Figure 13. SAESI-MS/MS spectrum of the solution showing the signal of **MS-Int. VI** at *m*/*z* 991.

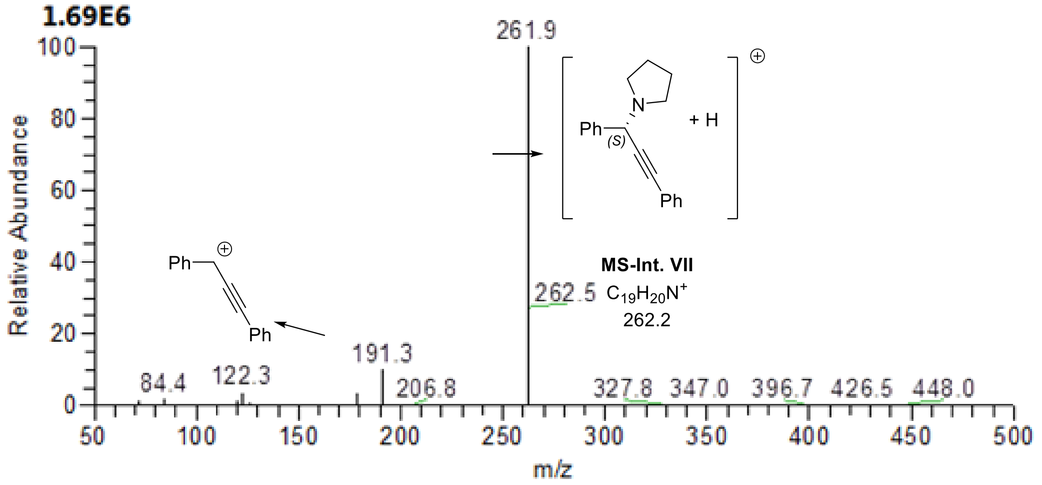

Supplementary Figure 14. SAESI-MS/MS spectrum of the solution showing the signal of **MS-Int. VII** at *m*/*z* 262.

12. Computational studies

Computational method

All calculations were performed with the Gaussian 09 program.[25] Geometries have been fully optimized with the density functional theory of M06 method.[26] The LANL2DZ[27] basis set in conjunction with the LANL2DZ pseudopotential[28] was used for Cu and Br atoms, while the 6-31G(d,p)[29] basis set was used for other atoms.

Harmonic vibration frequency calculations were conducted at the same level of theory at 273.15 K. The solvent effects were determined by single-point calculations of the gas-phase stationary points at M06/SDD[30]-6-311++G(2d,p) level by using SMD[31] solvation model. The reported energies are the solution-phase Gibbs free energies (ΔG_{sol}) in toluene.

Energies of two coordination modes

Supplementary Table 2. Electronic energies (E_{elec}), enthalpies ($H_{273.15}$), Gibbs free energies ($G_{273.15}$), thermal correction to Gibbs free energy ($cor\ G_{gas}$), solvation energies (E_{sol}), solvation free energies (G_{sol}) in toluene ($\varepsilon = 2.37$) for two coordination modes.

species	E_{elec} (a.u.)	$H_{273.15}$ (a.u.)	$G_{273.15}$ (a.u.)	$cor\ G_{gas}$ (a.u.)	E_{sol} (a.u.)	G_{sol} (a.u.)
**Int_N,P**	-2258.4798230	-2257.808072	-2257.908485	0.571338	-2260.3755434	-2259.8042054
**Int_N**	-2258.4792553	-2257.807817	-2257.906431	0.572824	-2260.3746990	-2259.8018750

Cartesian coordinates of the optimized structures for two coordination modes

Int_N,P

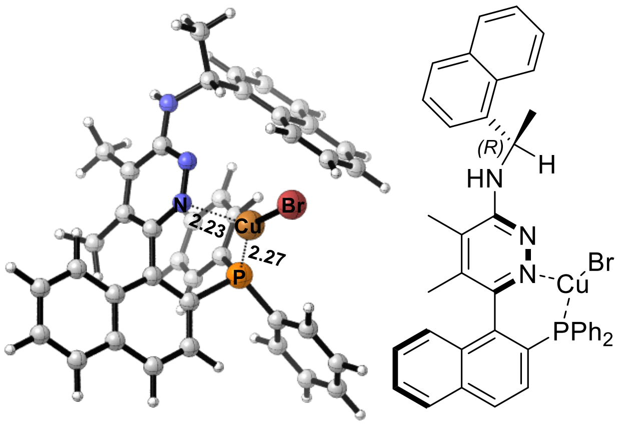

Cu	0.31602000	-1.02319100	-1.51118200
P	1.08350400	-1.17170200	0.62285800
C	2.59598600	-0.13687700	0.40814900
C	2.46267400	1.16086900	-0.07806000
C	1.10511200	1.76131200	-0.22179700
N	0.30379100	1.16489500	-1.10253600
Br	0.08023400	-2.09920900	-3.58293000
C	3.62240700	1.91229000	-0.42689400
C	3.88304400	-0.69418800	0.59452400
H	3.97550800	-1.70824300	0.97815200
C	5.00866500	0.03097100	0.30349800
H	5.99693700	-0.39869600	0.46108600

C	4.91241100	1.33899600	-0.22023800
C	3.54698000	3.21131000	-0.99407100
H	2.56886500	3.64449900	-1.19402000
C	4.68387500	3.90785200	-1.31525100
H	4.60370100	4.89752000	-1.75881900
C	5.95924100	3.34616000	-1.08630700
C	6.06680400	2.08811400	-0.55401800
H	7.04325200	1.63569900	-0.38697400
H	6.85189500	3.91062600	-1.34464400
C	0.64574000	2.82341500	0.60000800
C	-0.66619000	3.22755000	0.44005300
C	-1.44465600	2.53367800	-0.52363200
N	-0.95727500	1.53497500	-1.26178800
N	-2.74980200	2.88320000	-0.74730800
H	-3.16720900	3.47850600	-0.04424300
C	1.52741700	3.44424000	1.64233600
H	2.46725000	2.89839500	1.75127000
H	1.77612500	4.48372300	1.39280400
H	1.02612500	3.44333500	2.61720100
C	-1.30444800	4.30843500	1.25105200
H	-0.59765200	4.80113200	1.92078500
H	-1.74159000	5.08113900	0.60442300
H	-2.11702900	3.90036800	1.87117900
C	-3.70126700	1.96451000	-1.38855300
H	-3.12209200	1.41249100	-2.13069200
C	-4.76152600	2.77746600	-2.11397700
H	-4.29845200	3.39886000	-2.88669600
H	-5.49416600	2.11468900	-2.58520000
H	-5.30774800	3.44041200	-1.43075400
C	1.69073100	-2.74403400	1.31200600
C	1.94114200	-3.76587200	0.38943500
C	1.92719400	-2.96506900	2.67155900
C	2.44351200	-4.98813900	0.82274500
H	1.73133700	-3.59701600	-0.66857400
C	2.41851000	-4.19249100	3.10086900
H	1.71754300	-2.17998200	3.39655000
C	2.68105800	-5.20126100	2.17718100
H	2.63734900	-5.77775100	0.10095200
H	2.59631600	-4.36382200	4.16017100
H	3.06513800	-6.16034000	2.51744000
C	0.17315100	-0.30351700	1.95060400
C	0.80601200	0.32528400	3.02878300
C	-1.21167100	-0.19032400	1.80809500
C	0.05906300	1.05358600	3.94875400

H	1.89059700	0.26754500	3.13024800
C	-1.95805700	0.55020400	2.72019900
H	-1.70647500	-0.66948700	0.96340200
C	-1.32044100	1.17453000	3.78842800
H	0.55606200	1.53896400	4.78659600
H	-3.03601700	0.63865400	2.58543700
H	-1.89947400	1.75560900	4.50343700
C	-4.28843400	1.00449100	-0.36472700
C	-3.97208000	-0.39001300	-0.34893600
C	-5.12606600	1.51657300	0.60481600
C	-3.11371900	-1.01697500	-1.29385700
C	-4.53364100	-1.20628900	0.68623600
C	-5.68265300	0.70964300	1.61599000
H	-5.37704400	2.57794500	0.59258300
C	-2.82191200	-2.35789700	-1.21285700
H	-2.64340700	-0.43897600	-2.08674800
C	-4.21144300	-2.58513300	0.74143700
C	-5.38849900	-0.62889300	1.65435800
H	-6.34603400	1.15132200	2.35614900
C	-3.37415900	-3.15131300	-0.18386200
H	-2.14631400	-2.79225900	-1.94926600
H	-4.64624300	-3.18344900	1.54125300
H	-5.80737100	-1.27175700	2.42739200
H	-3.13123000	-4.20991400	-0.13095000

Int_N

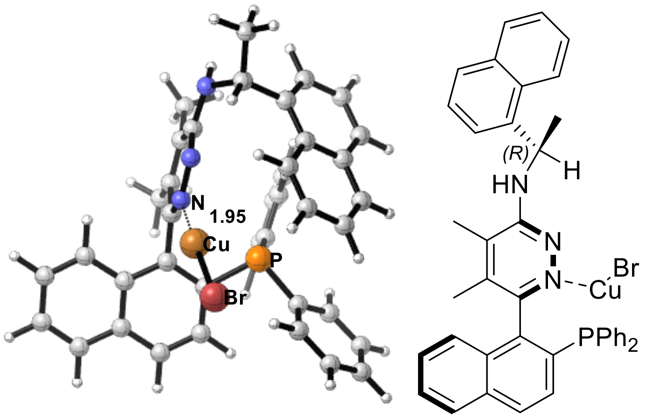

Cu	1.04801400	-0.06385300	-2.17703300
P	0.31783400	-0.92189000	0.92703200
C	1.93457700	-0.00544200	0.86243400
C	1.92108200	1.28475700	0.34238400
C	0.62429100	1.86767000	-0.09428600
N	0.16849600	1.41530700	-1.26399000
Br	2.06351100	-1.84847400	-3.18667700

C	3.12407400	2.03134300	0.19539200
C	3.17133900	-0.57643900	1.25148600
H	3.18400200	-1.59021700	1.64710800
C	4.34213400	0.12421200	1.12310900
H	5.28730900	-0.32523100	1.42490400
C	4.35572000	1.43538300	0.59349400
C	3.14660300	3.33977900	-0.35042100
H	2.21159200	3.79514300	-0.67340100
C	4.32887200	4.02251200	-0.48826400
H	4.32990300	5.02309600	-0.91415500
C	5.54759600	3.43294600	-0.08727200
C	5.55739300	2.16725200	0.44067300
H	6.49213800	1.69916600	0.74568500
H	6.47753500	3.98398600	-0.20453400
C	-0.15096100	2.74161000	0.70065300
C	-1.42385500	3.05055000	0.25083300
C	-1.83544500	2.47471200	-0.97756100
N	-1.03205700	1.71843600	-1.72395800
N	-3.10703500	2.66364600	-1.47126400
H	-3.72650500	3.13296700	-0.82182200
C	0.36922600	3.24679600	2.01073400
H	1.35961700	2.84048700	2.22795800
H	0.44478800	4.34122400	2.01155600
H	-0.29519700	2.95433500	2.83444600
C	-2.39132700	3.87184800	1.03939500
H	-1.94508900	4.28245000	1.94706600
H	-2.78208600	4.71199700	0.45035100
H	-3.24931700	3.25375100	1.34585300
C	-3.77564200	1.53059600	-2.14451000
H	-3.08155700	1.18384400	-2.91223000
C	-5.02912800	2.04145900	-2.83132000
H	-4.77154600	2.80730900	-3.56954200
H	-5.54646500	1.22094400	-3.33794300
H	-5.73659800	2.48463500	-2.11850400
C	0.83250500	-2.60521200	1.43603800
C	1.48517800	-3.37873000	0.46404800
C	0.48776500	-3.19572100	2.65382300
C	1.81369600	-4.70291900	0.72663800
H	1.72799900	-2.94226400	-0.50909800
C	0.80391300	-4.52876800	2.90533000
H	-0.03473700	-2.61557800	3.41247300
C	1.47176500	-5.28234300	1.94689200
H	2.32909700	-5.28681000	-0.03254500
H	0.52849500	-4.97684900	3.85787800

H	1.72084000	-6.32229900	2.14641600
C	-0.38187800	-0.18413500	2.46883400
C	0.35853100	0.03918300	3.63597500
C	-1.72061700	0.21449200	2.43182500
C	-0.23320900	0.63626700	4.74332100
H	1.40822900	-0.25421000	3.67069800
C	-2.31728300	0.80954600	3.54062100
H	-2.29484700	0.06195500	1.51775300
C	-1.57280600	1.02186700	4.69714400
H	0.34998700	0.80468700	5.64614900
H	-3.36624400	1.09877900	3.49572100
H	-2.03414800	1.48636700	5.56614700
C	-4.03978100	0.41680700	-1.13936300
C	-3.30794300	-0.81316000	-1.14568800
C	-4.95069900	0.65329000	-0.12927800
C	-2.34139400	-1.15503000	-2.13011100
C	-3.53229400	-1.74689600	-0.08237100
C	-5.18759900	-0.27908100	0.89939400
H	-5.51041600	1.58984300	-0.11716800
C	-1.61912100	-2.31984100	-2.04880000
H	-2.12990400	-0.47124900	-2.94790400
C	-2.77086900	-2.94155500	-0.02809500
C	-4.48532800	-1.45637400	0.92189300
H	-5.92310300	-0.05902100	1.67014900
C	-1.83226200	-3.22371000	-0.98574400
H	-0.85239000	-2.53068900	-2.79283400
H	-2.94172200	-3.62641900	0.80202000
H	-4.63952800	-2.18453800	1.71713400
H	-1.23967100	-4.13375800	-0.92481600

NMR and HPLC spectra

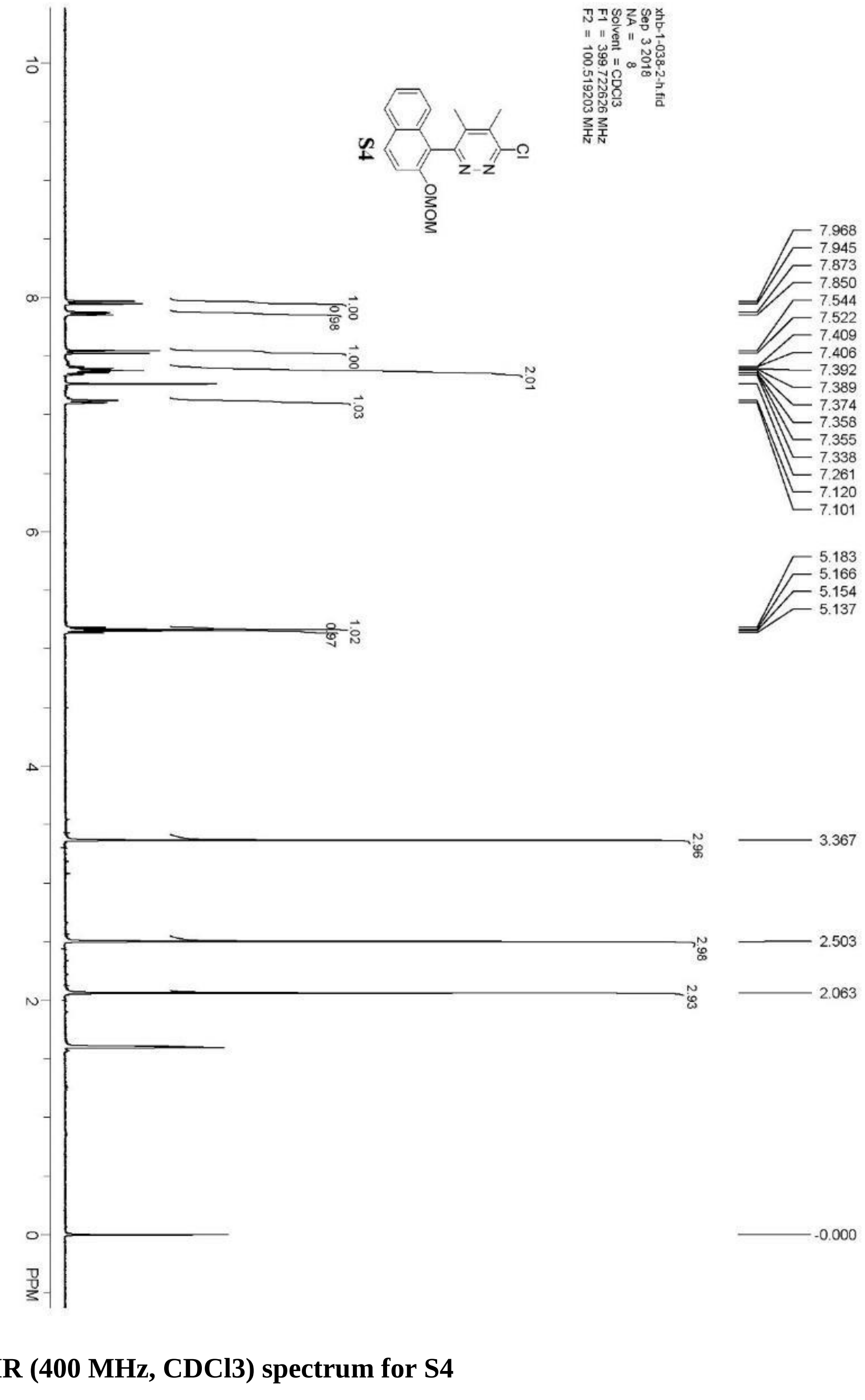

^{1}H NMR (400 MHz, CDCl3) spectrum for S4

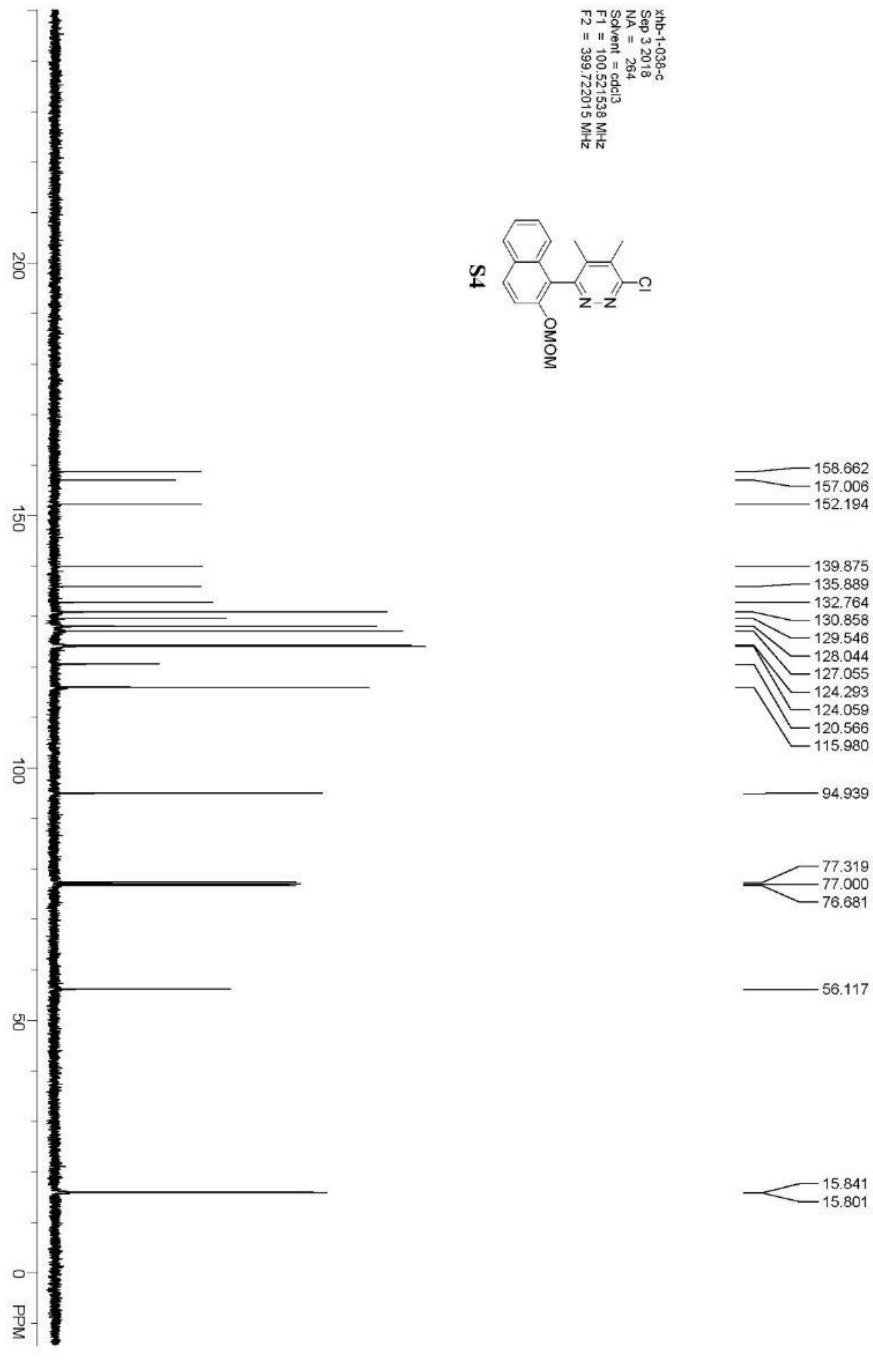

^{13}C NMR (400 MHz, $CDCl_3$) spectrum for S4

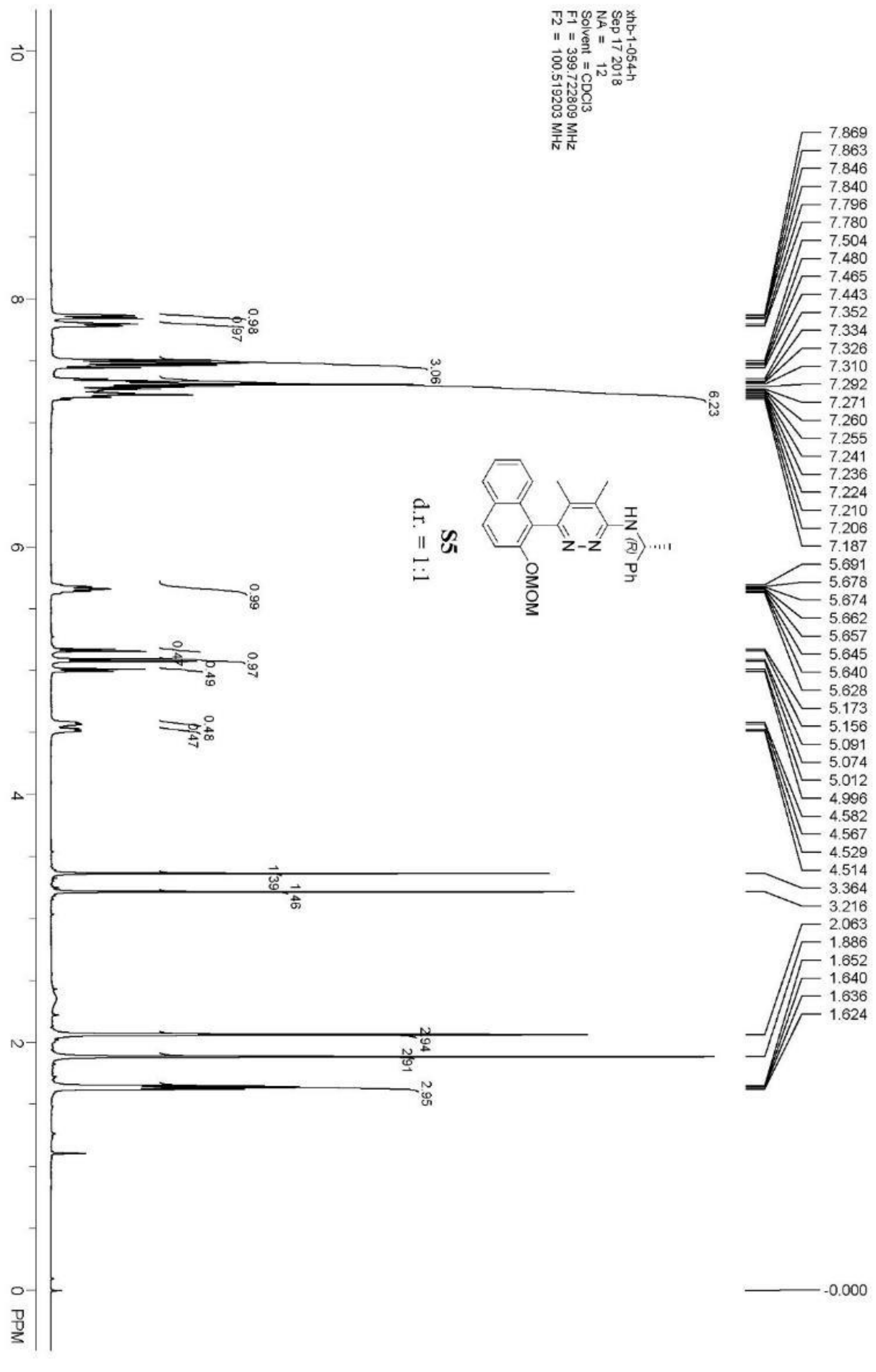

^{1}H NMR (400 MHz, CDCl3) spectrum for S5

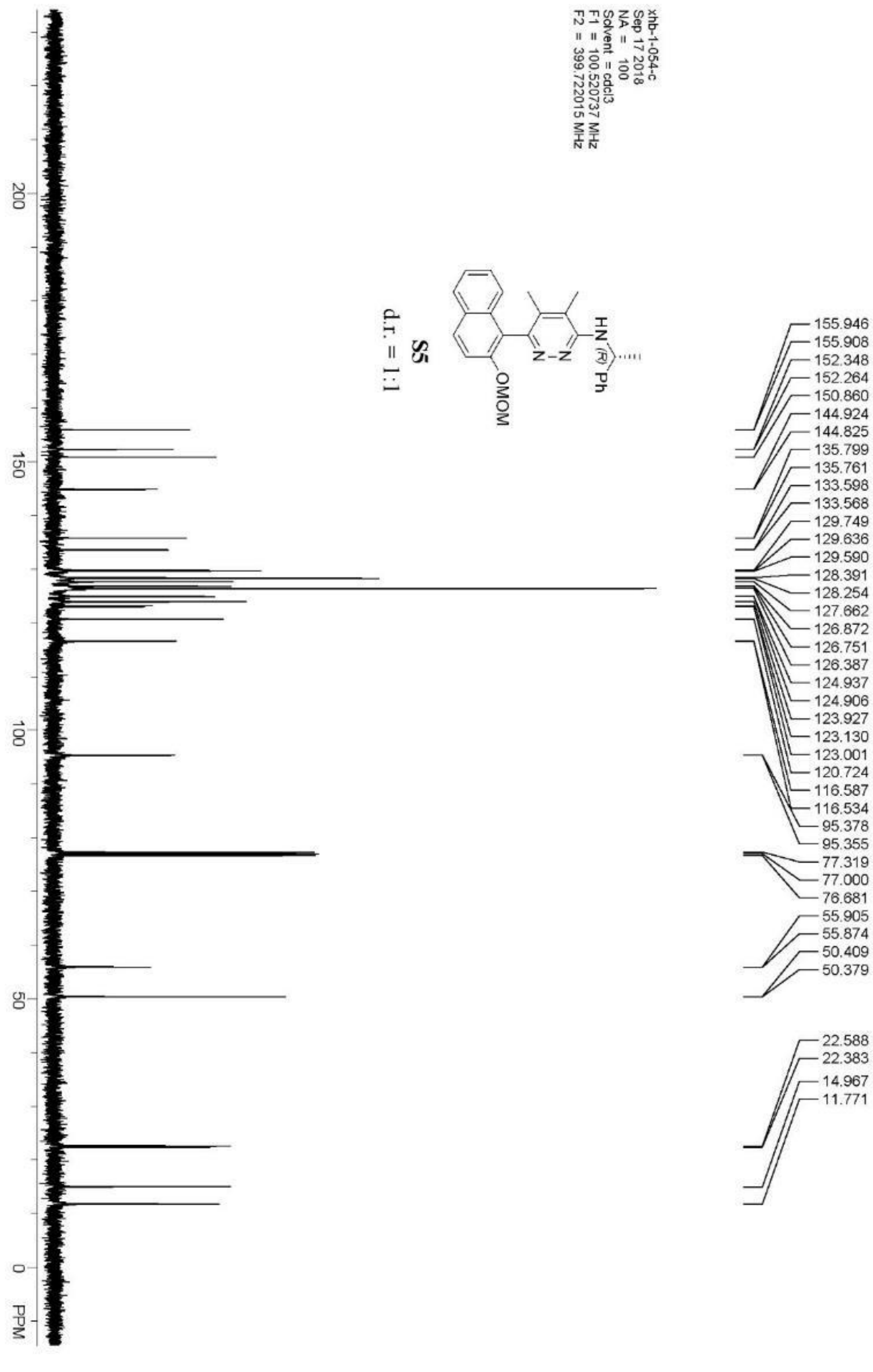

^{13}C NMR (400 MHz, CDCl$_3$) spectrum for S5

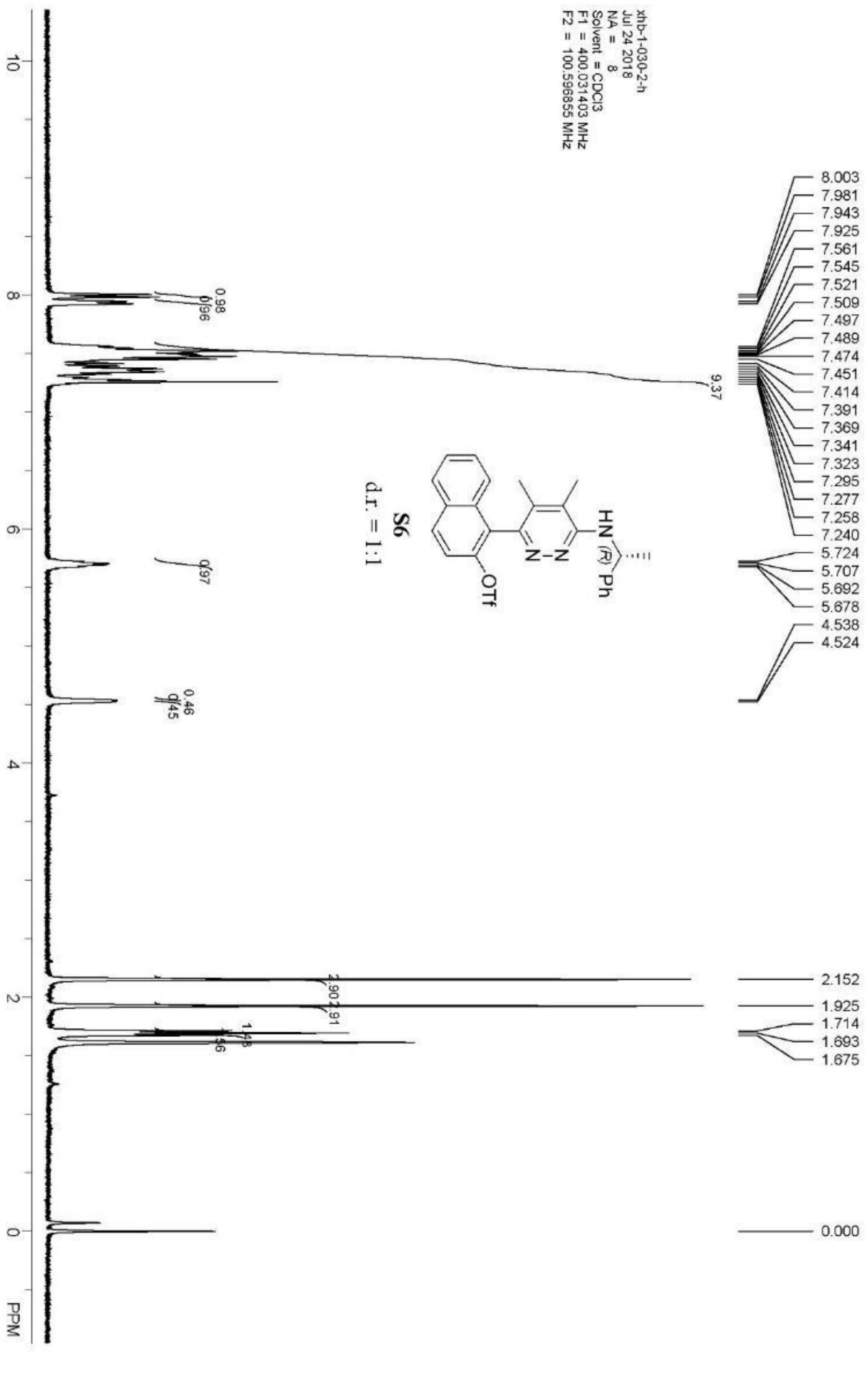

^{1}H NMR (400 MHz, CDCl3) spectrum for S6

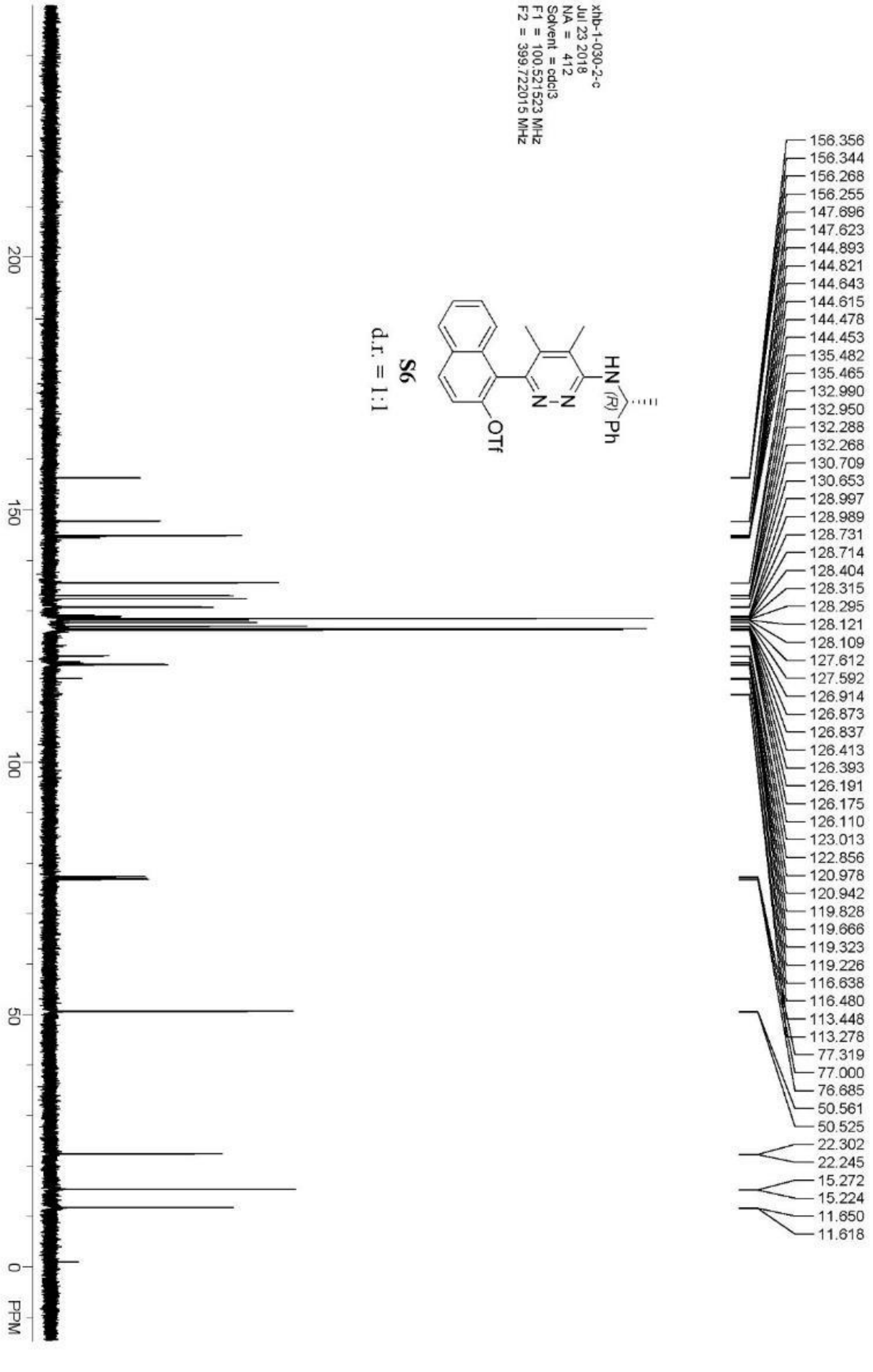

^{13}C NMR (400 MHz, $CDCl_3$) spectrum for S6

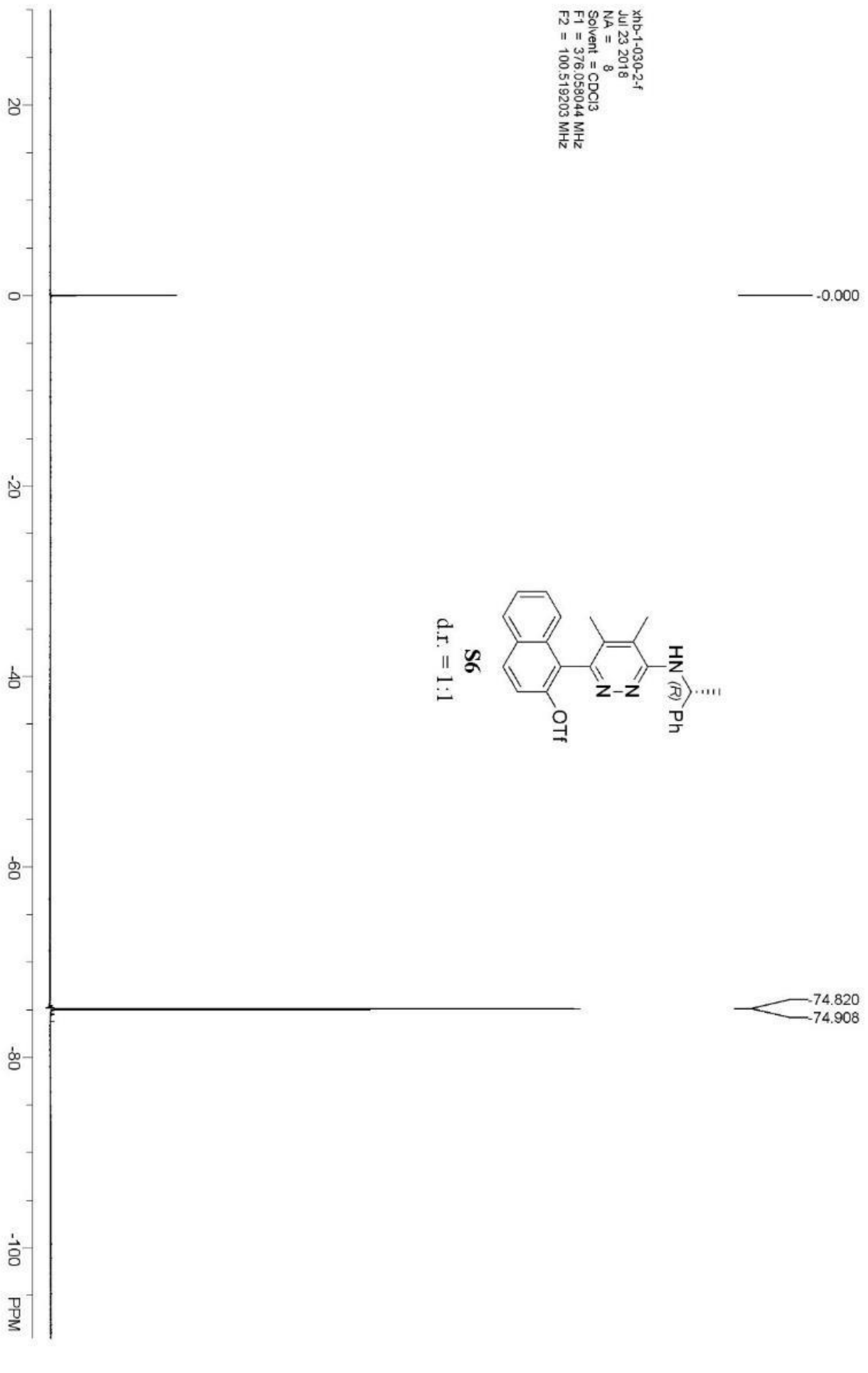

^{19}F NMR (376 MHz, CDCl3) spectrum for S6 ($CFCl_3$ was used as the internal standard)

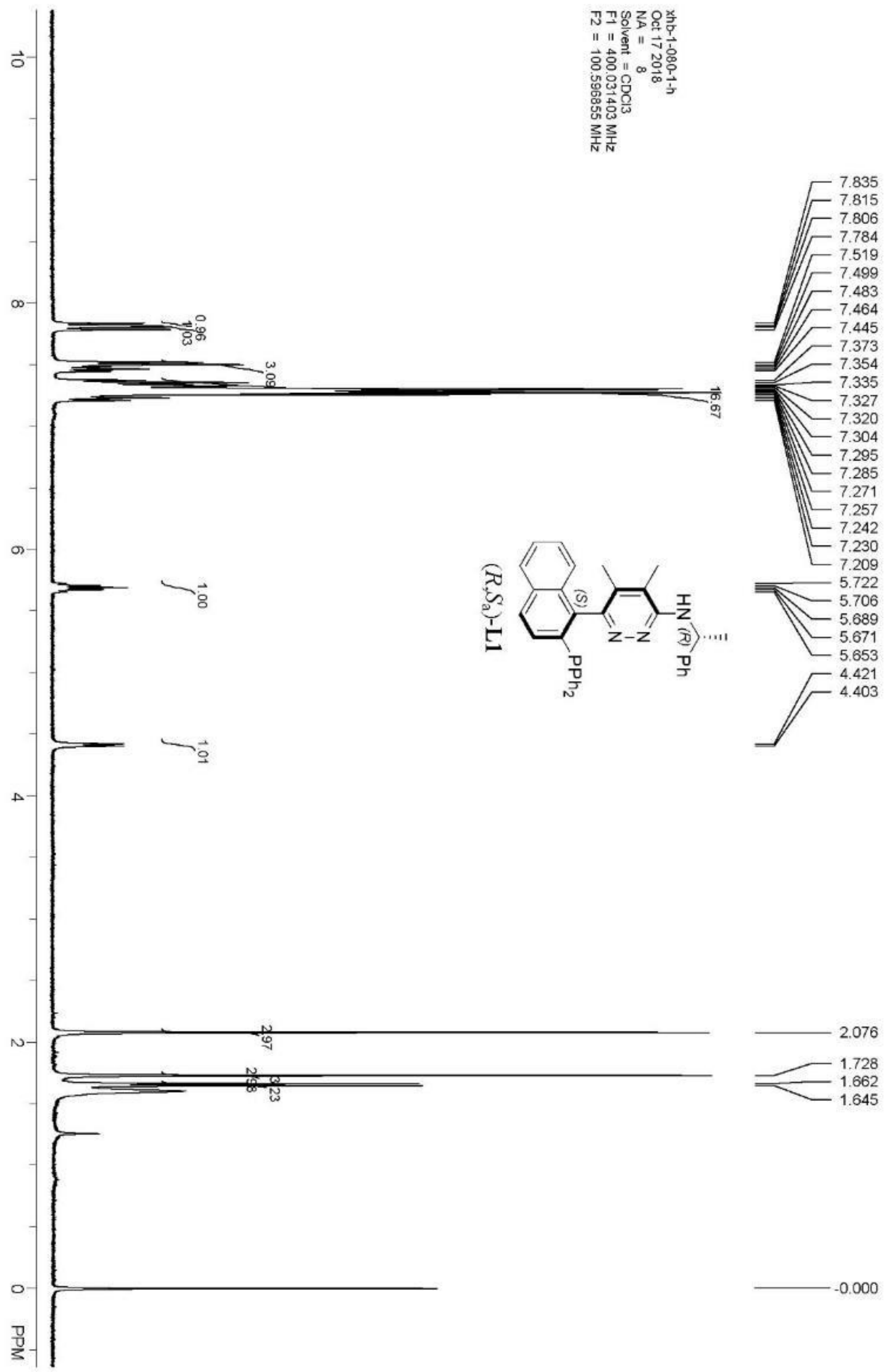

^{1}H NMR (400 MHz, CDCl3) spectrum for (*R*,*S*a)-**L1**

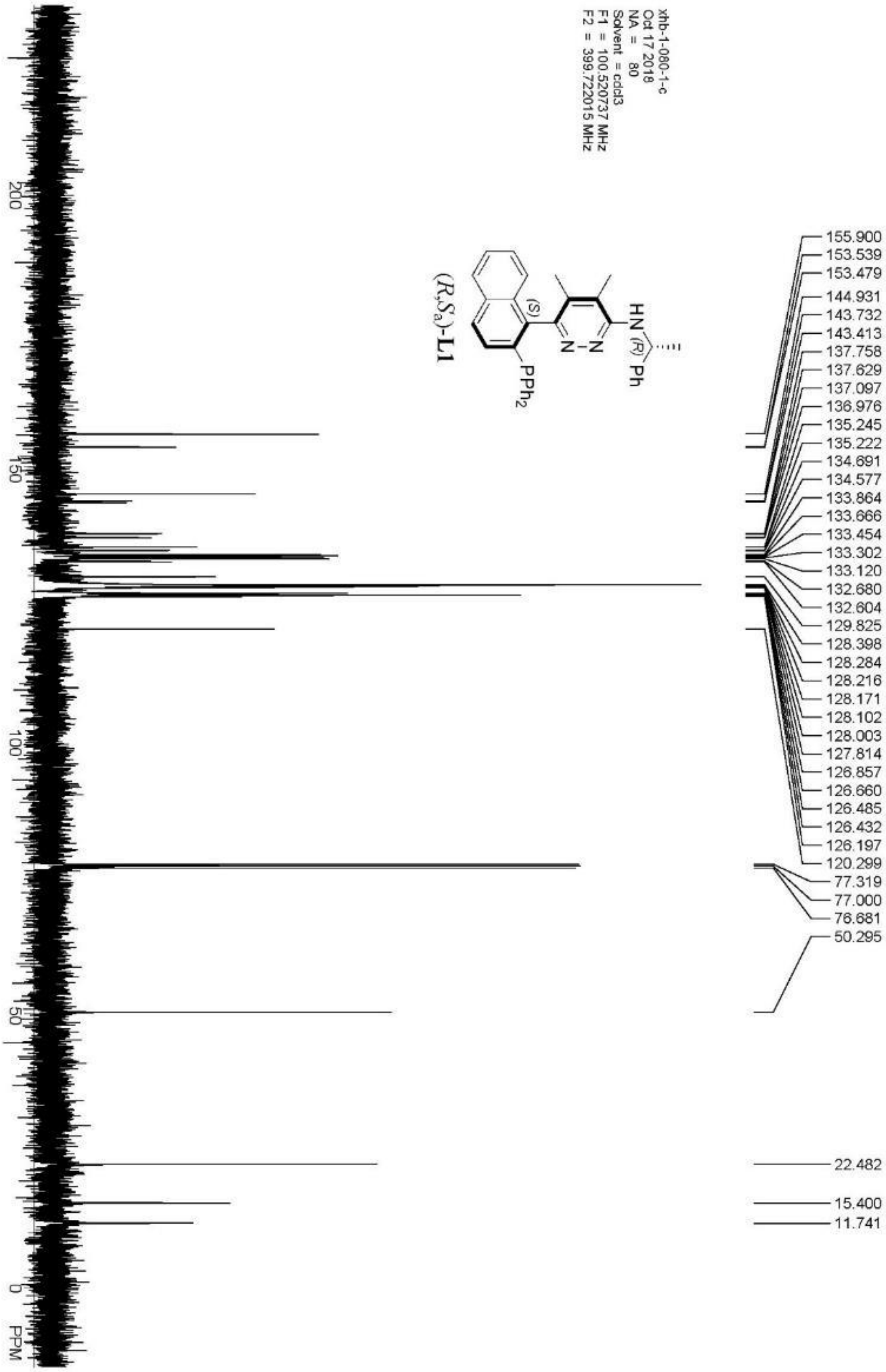

^{13}C NMR (400 MHz, CDCl$_3$) spectrum for (R,S_a)**-L1**

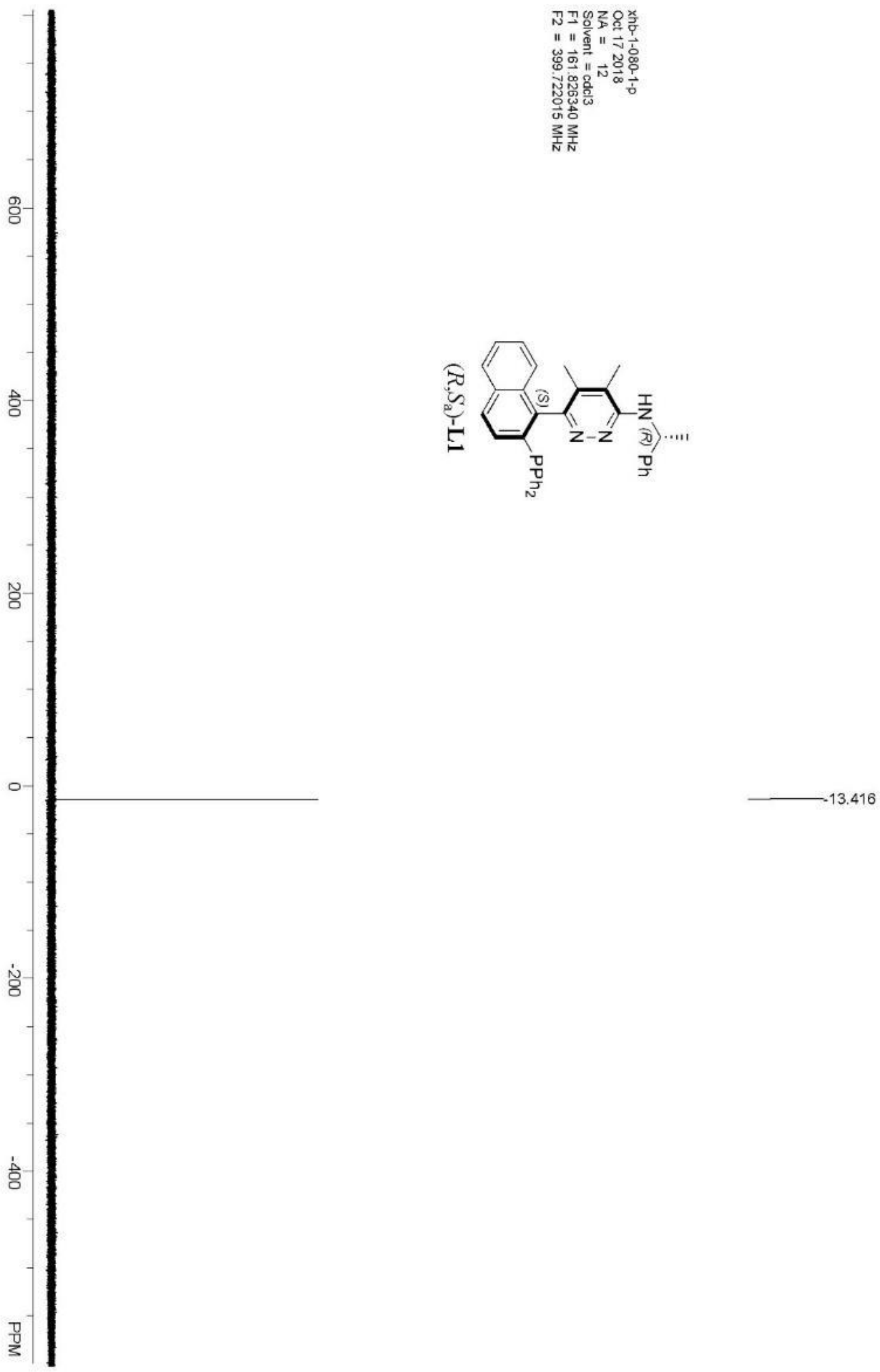

^{31}P NMR (162 MHz, $CDCl_3$) spectrum for (*R*,*S*$_a$)-L1 (85% H_3PO_4 in D_2O was used as the external standard)

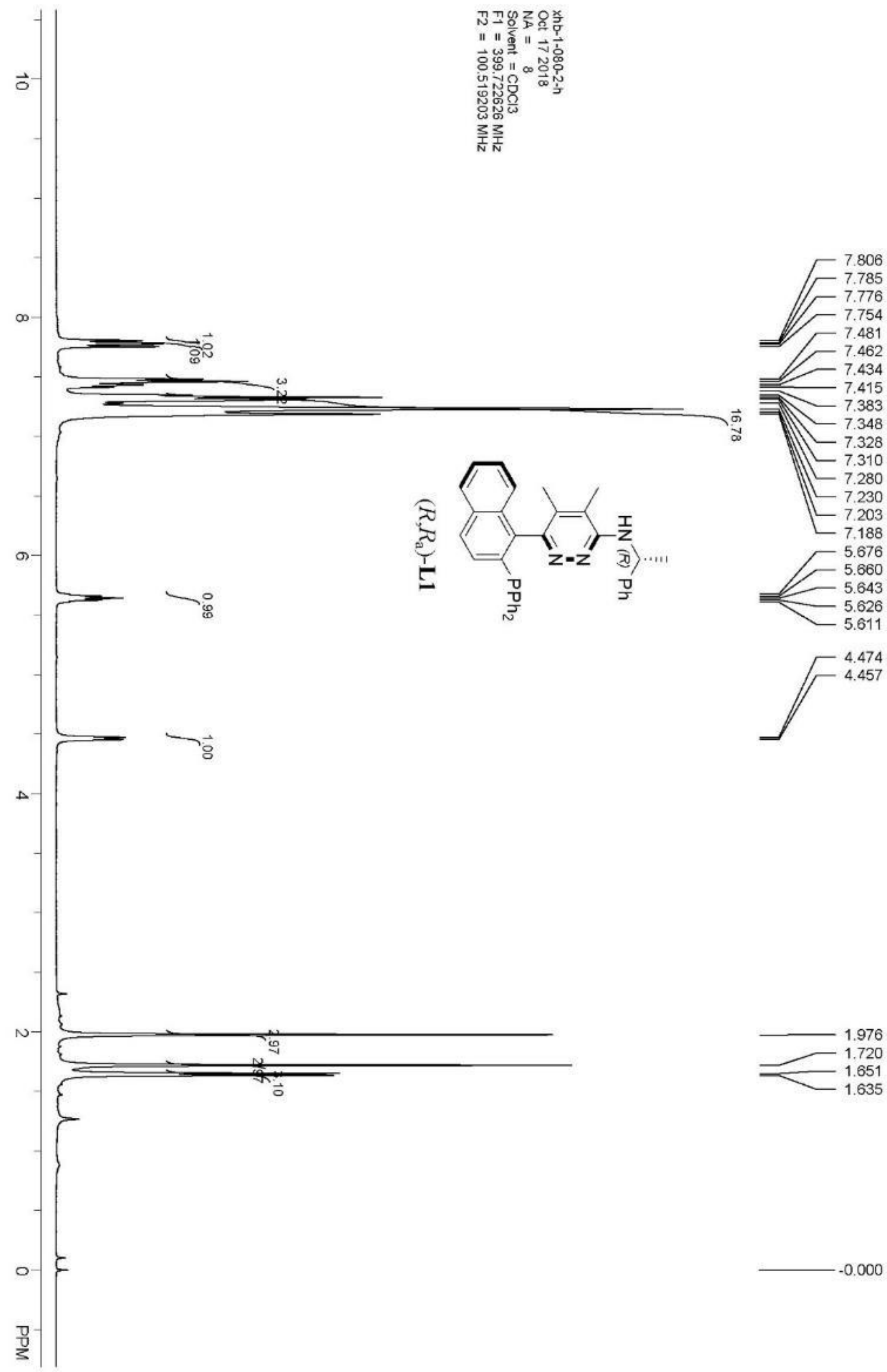

^{1}H NMR (400 MHz, CDCl3) spectrum for (R,R_a)-**L1**

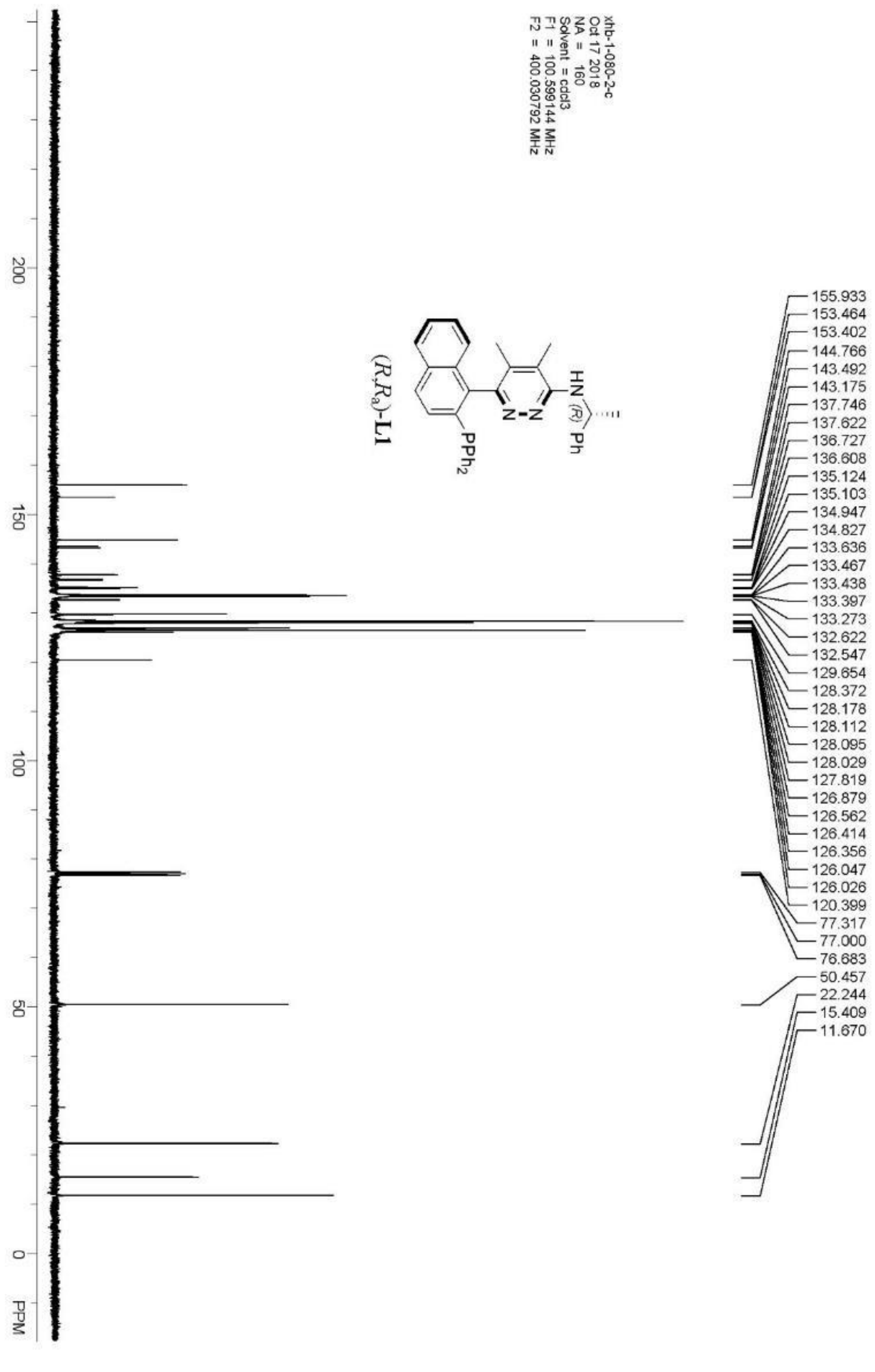

^{13}C NMR (400 MHz, CDCl$_3$) spectrum for (R,R_a)-**L1**

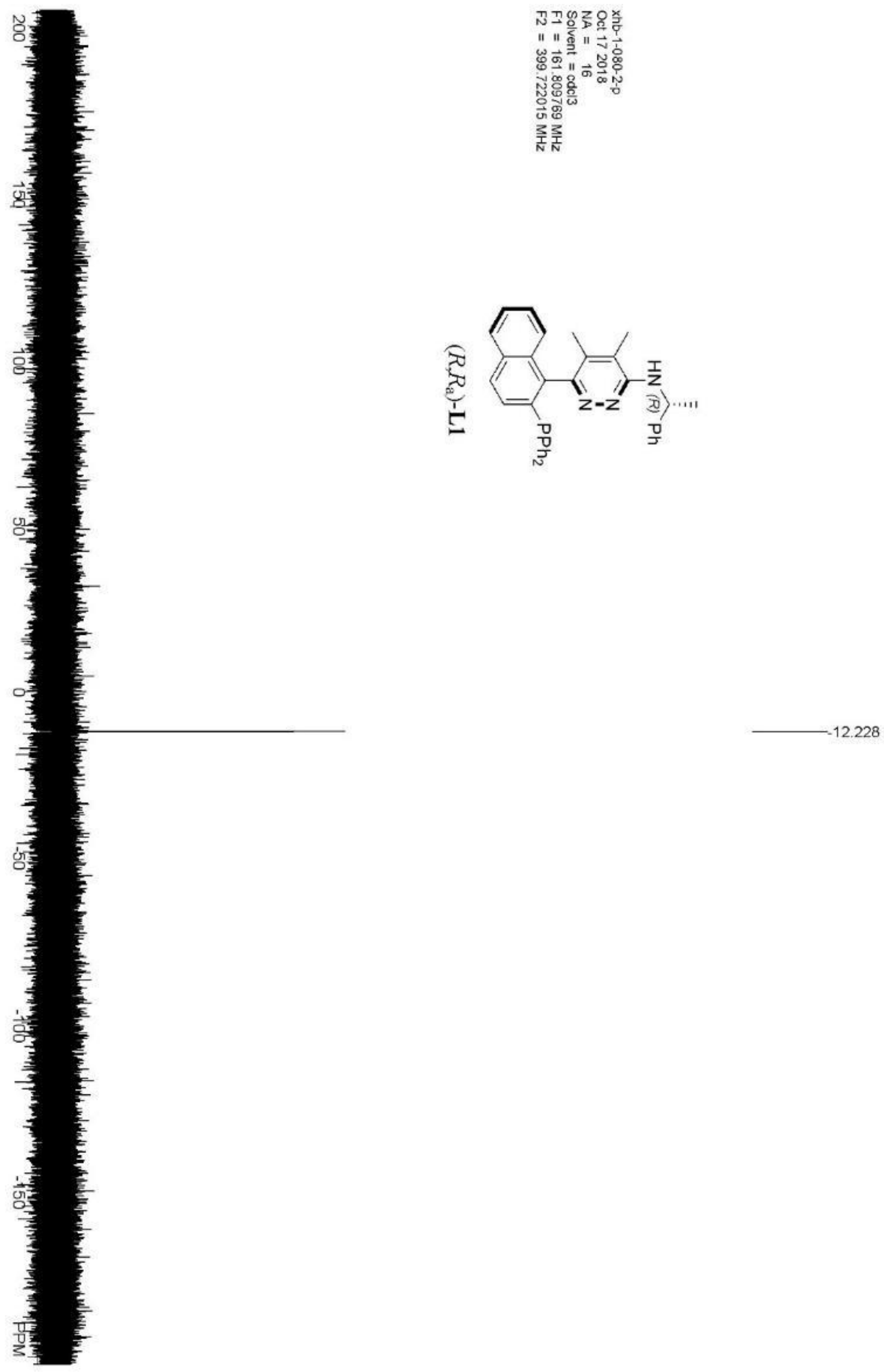

^{31}P NMR (162 MHz, $CDCl_3$) spectrum for (*R*,*R*a)-L1 (85% H_3PO_4 in D_2O was used as the external standard)

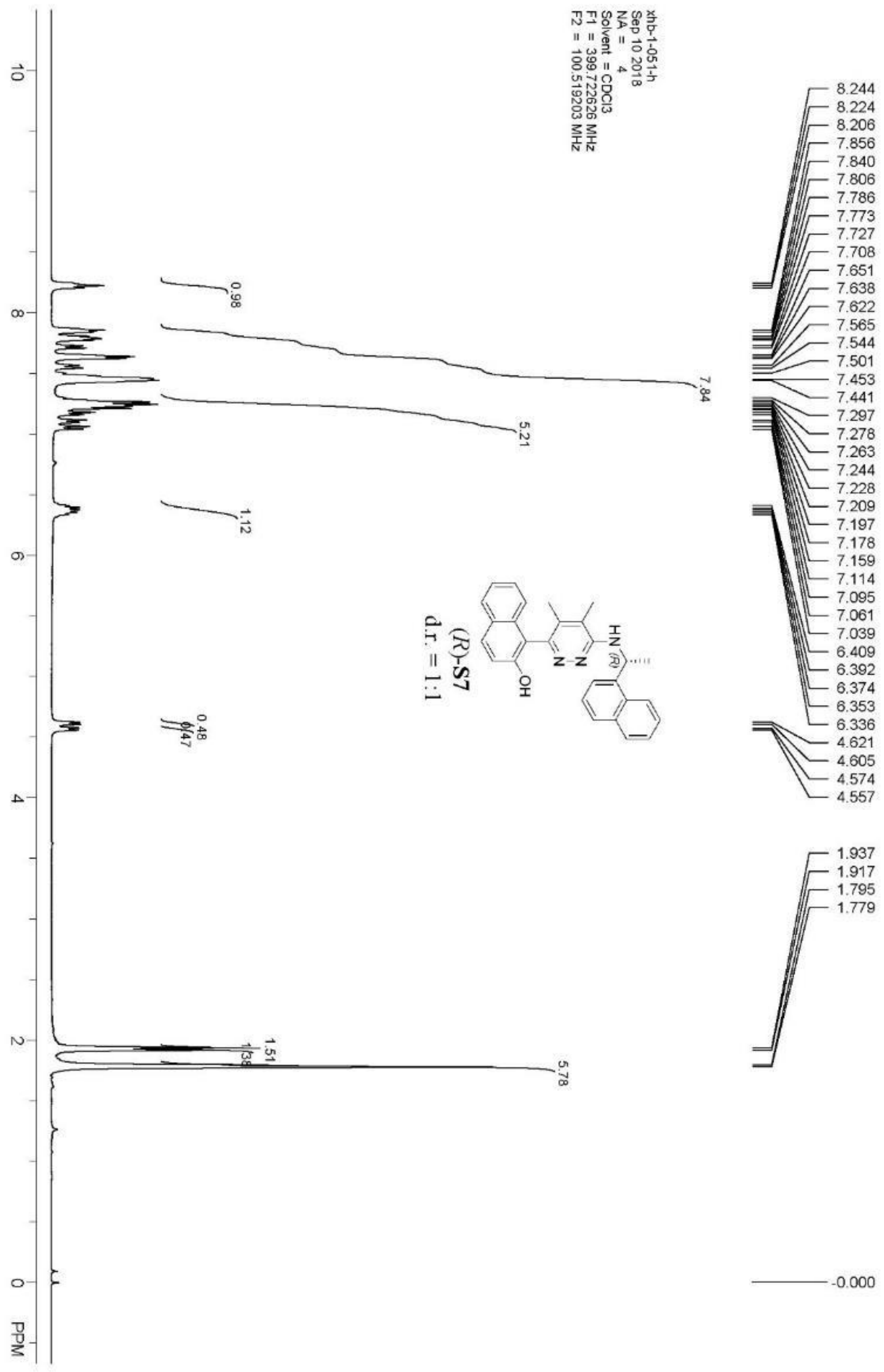

^{1}H NMR (400 MHz, CDCl3) spectrum for (*R*)**-S7**

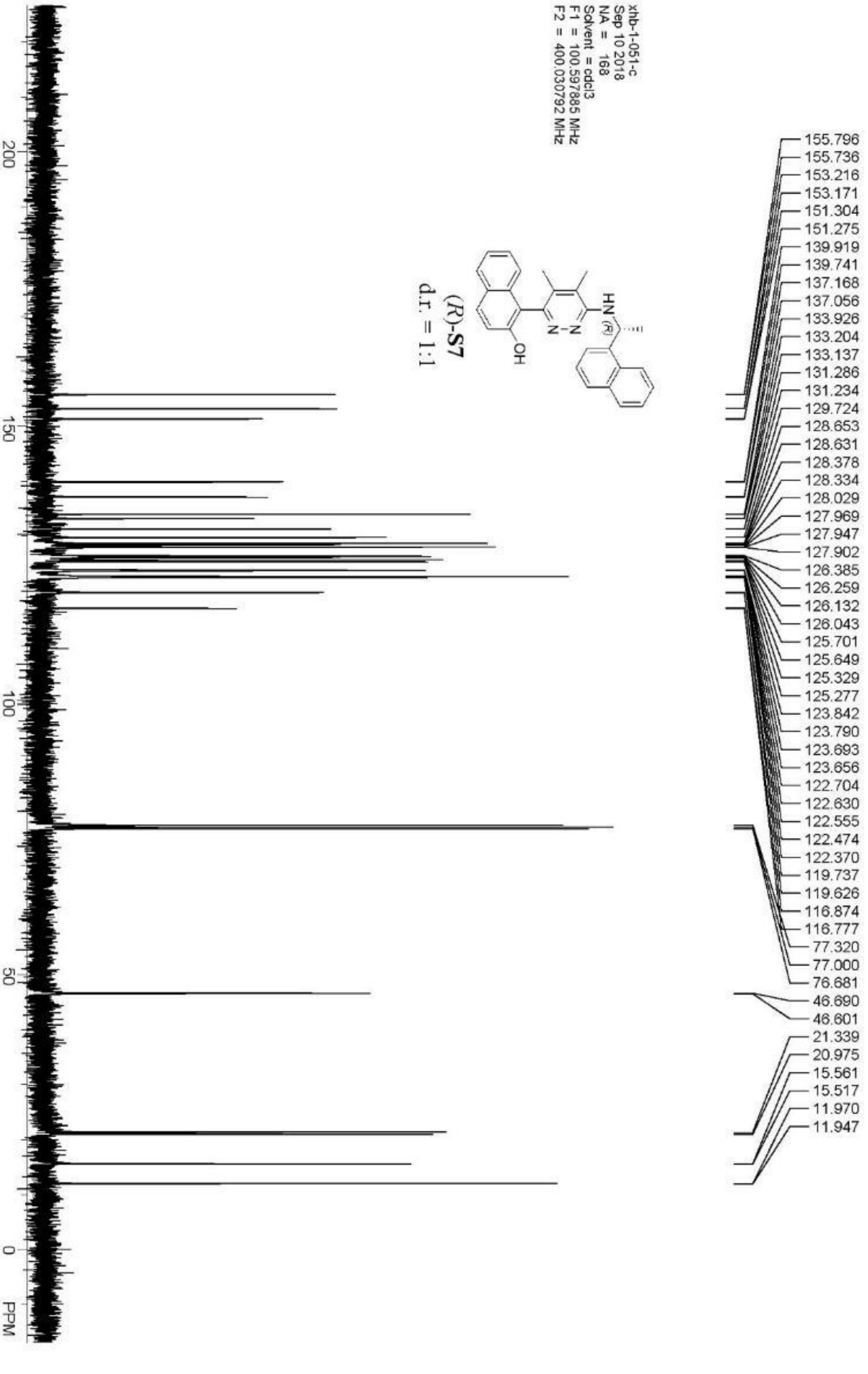

^{13}C NMR (400 MHz, CDCl$_3$) spectrum for (*R*)-S7

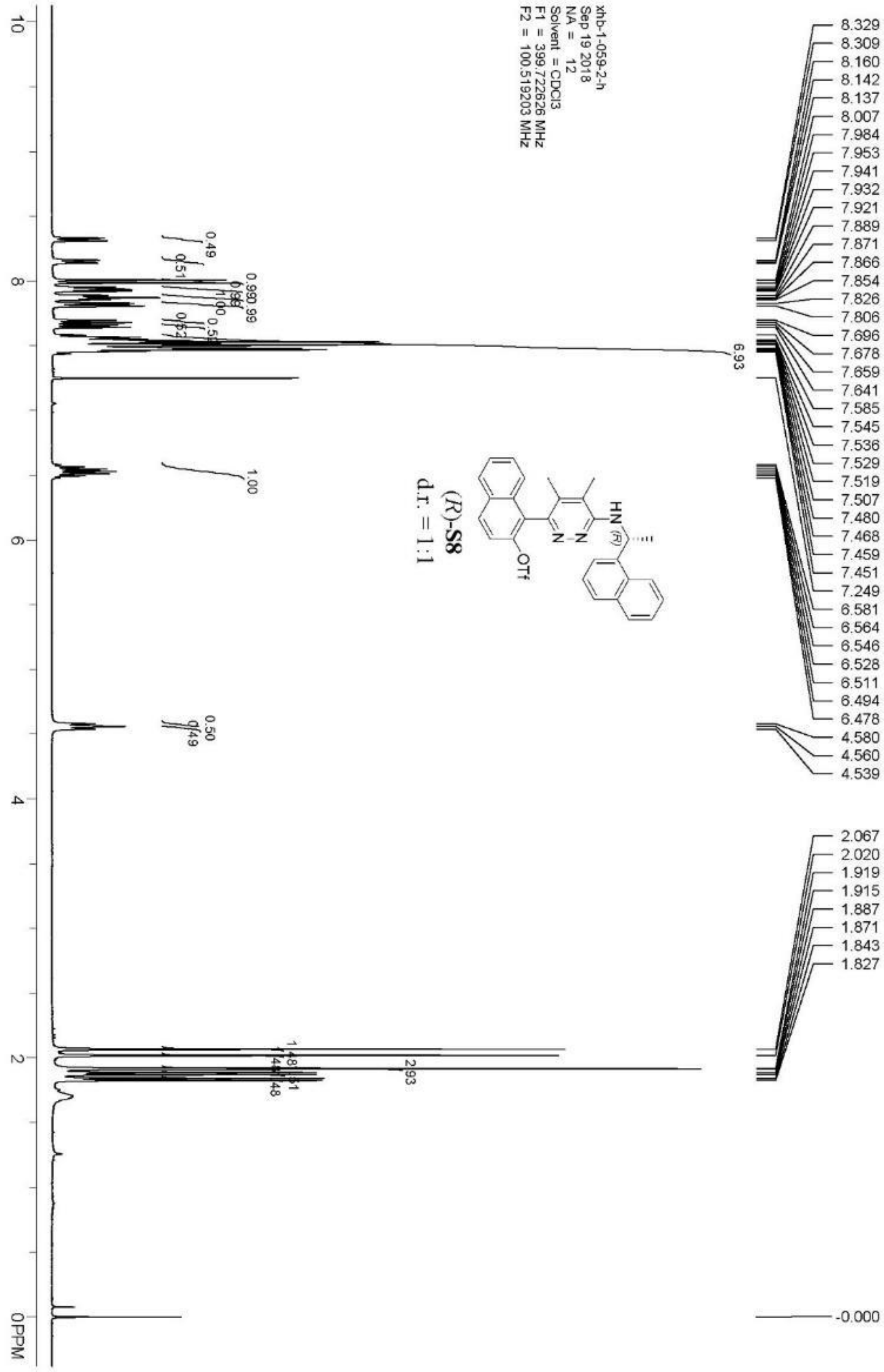

^{1}H NMR (400 MHz, CDCl3) spectrum for (*R*)**-S8**

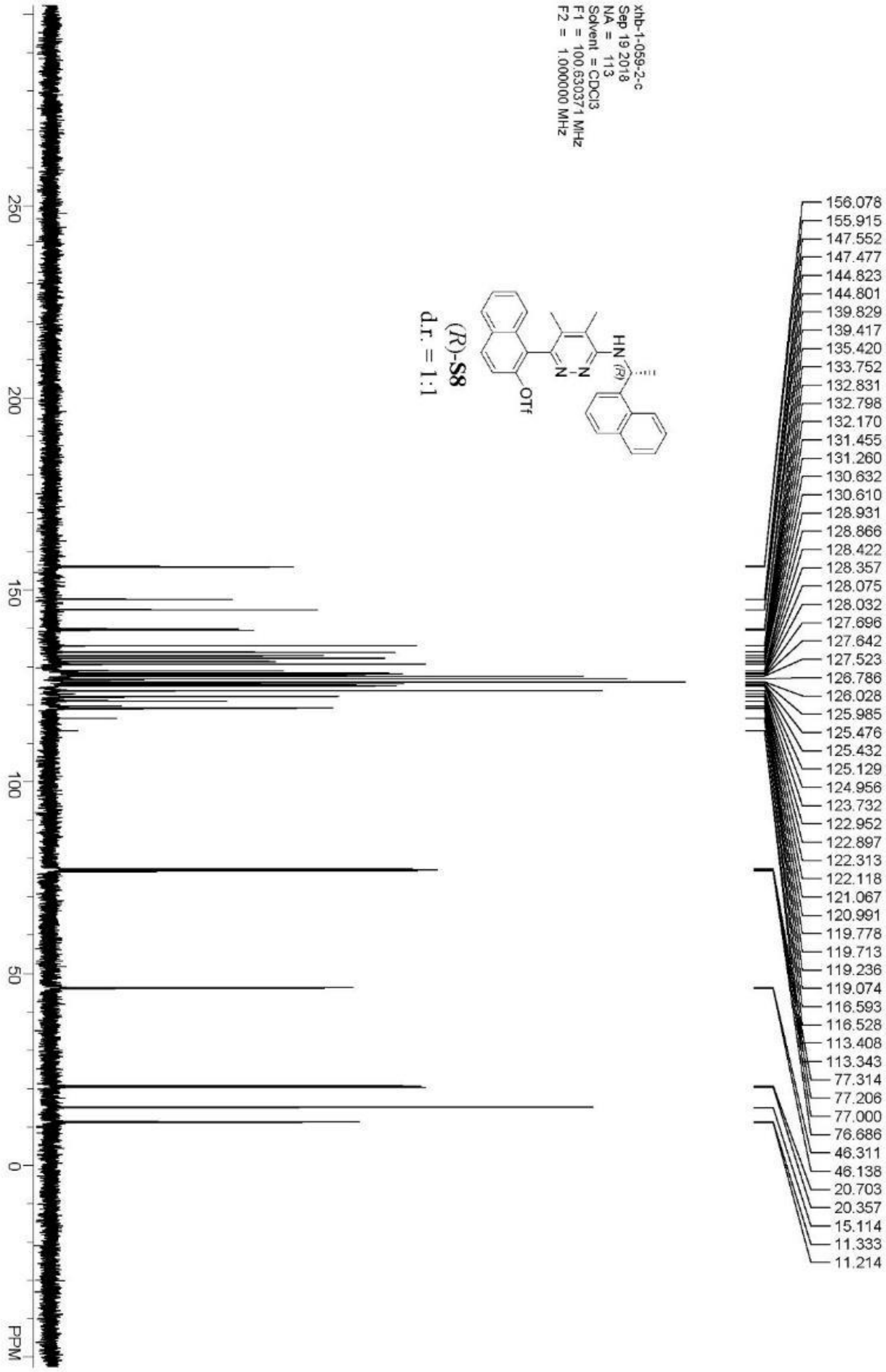

^{13}C NMR (400 MHz, $CDCl_3$) spectrum for (*R*)-S8

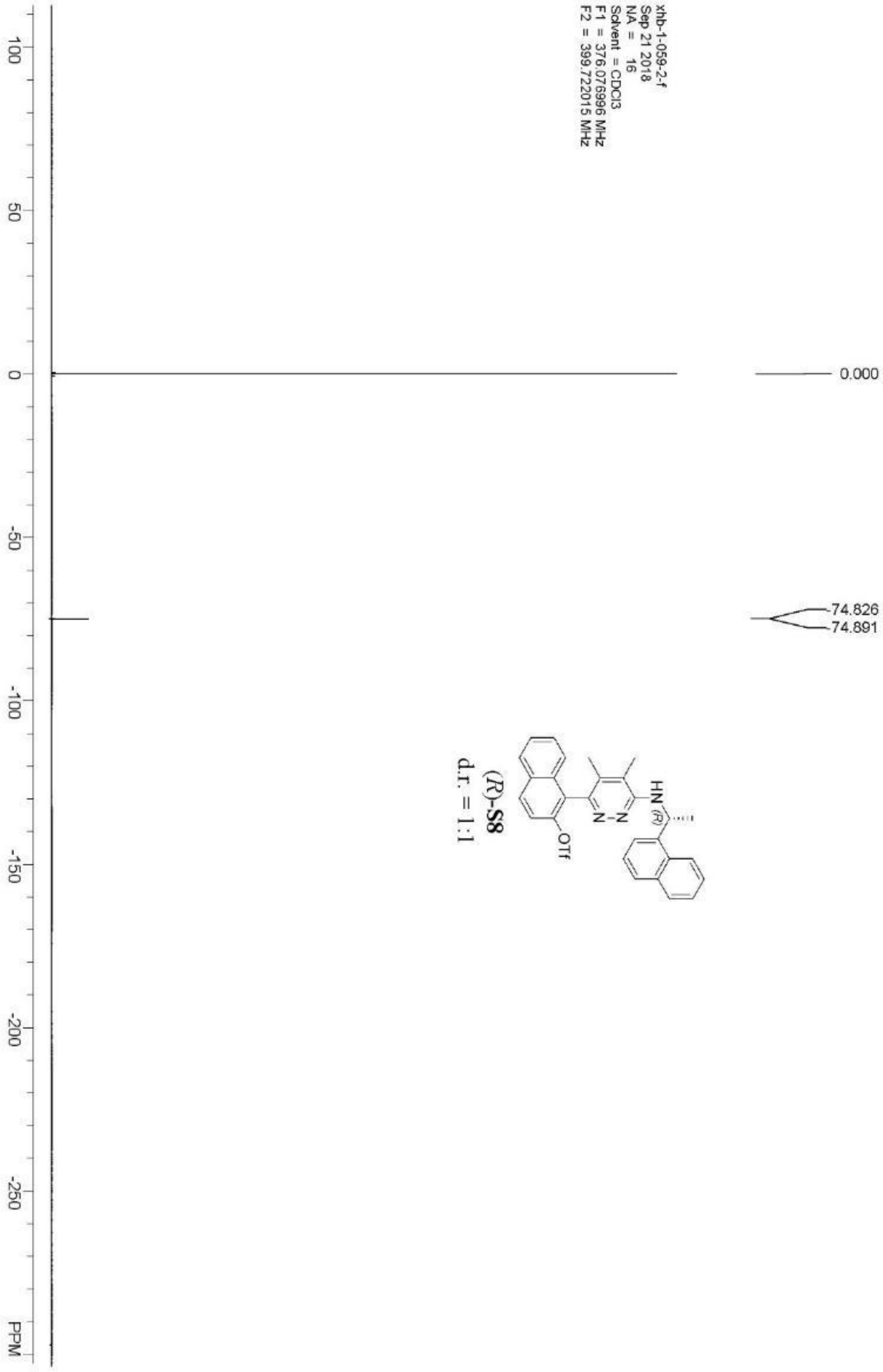

^{19}F NMR (376 MHz, CDCl$_3$) spectrum for (*R*)-S8 (CFCl$_3$ was used as the internal standard)

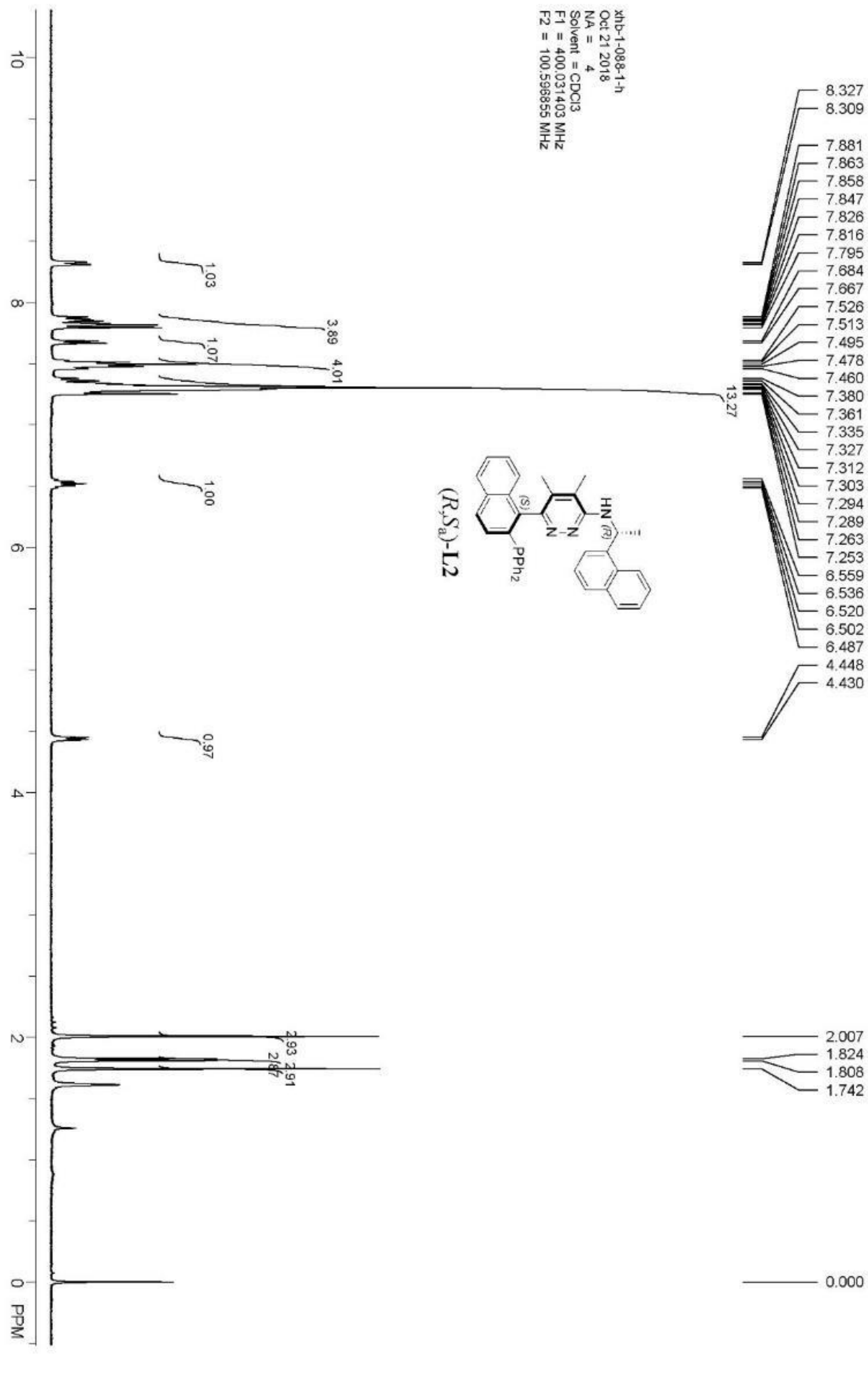

^{1}H NMR (400 MHz, CDCl3) spectrum for (R,S_a)-L2

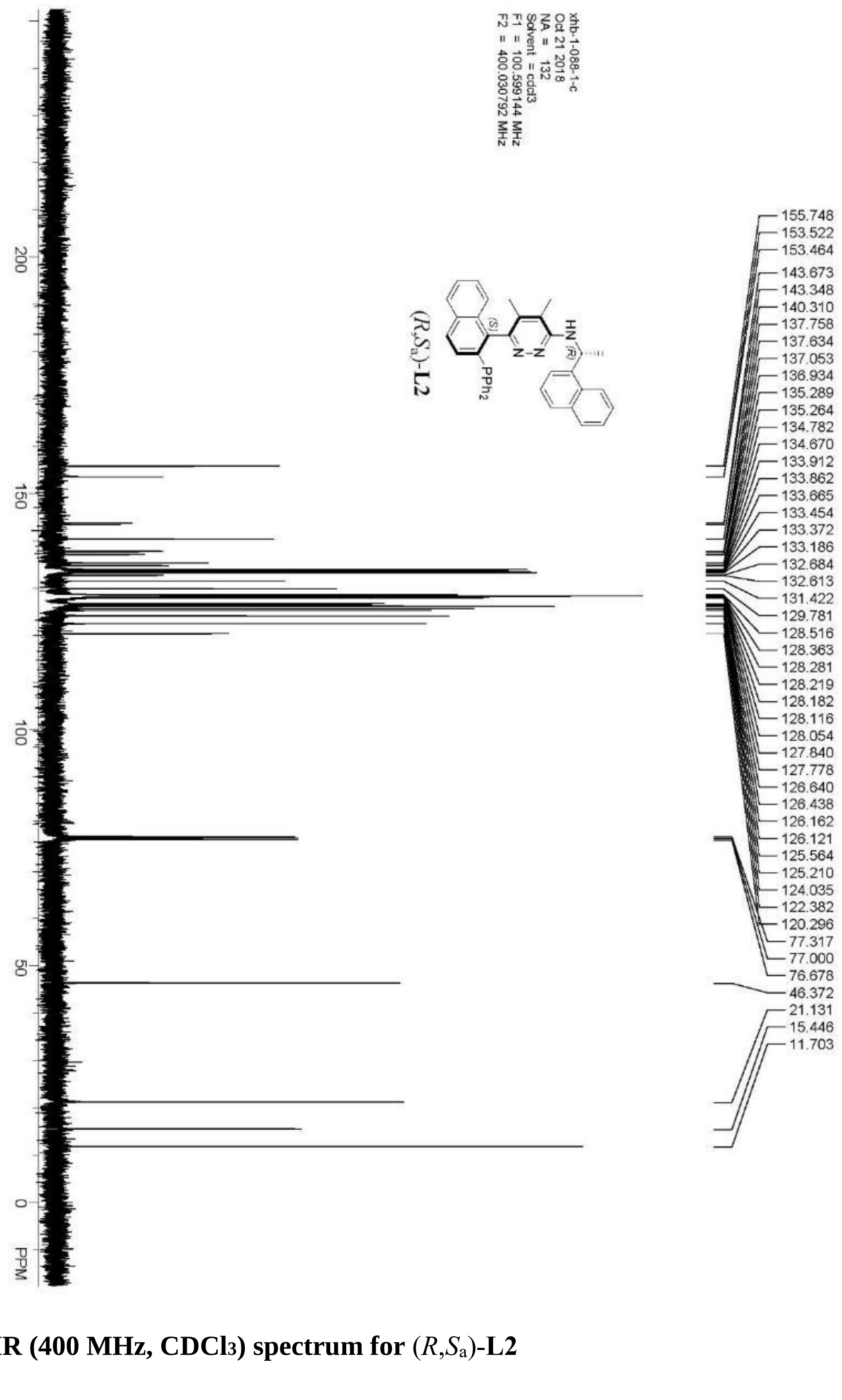

^{13}C NMR (400 MHz, CDCl$_3$) spectrum for (R,S_a)-**L2**

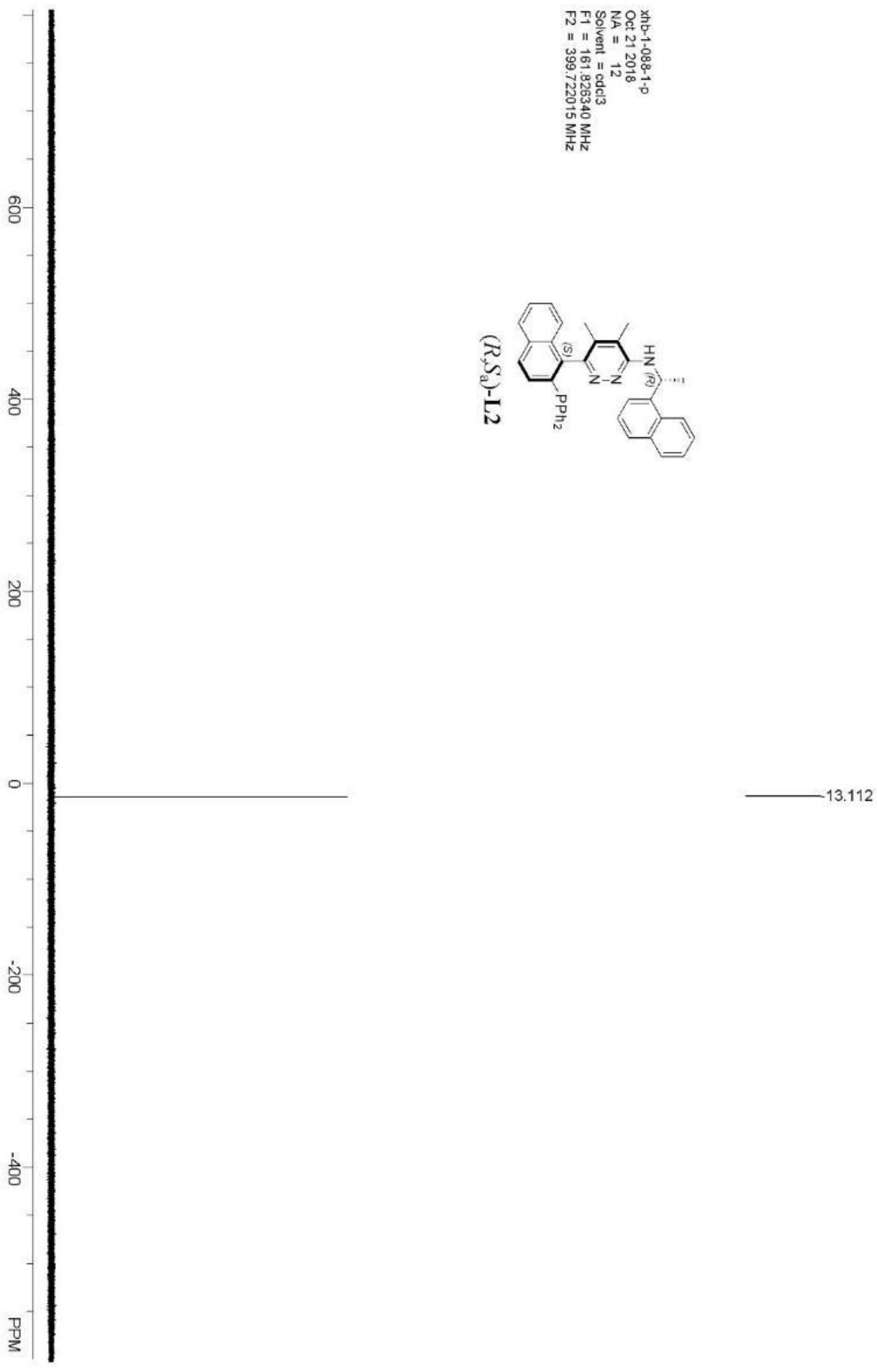

^{31}P NMR (162 MHz, $CDCl_3$) spectrum for (*R*,*S*a)-L2 (85% H_3PO_4 in D_2O was used as the external standard)

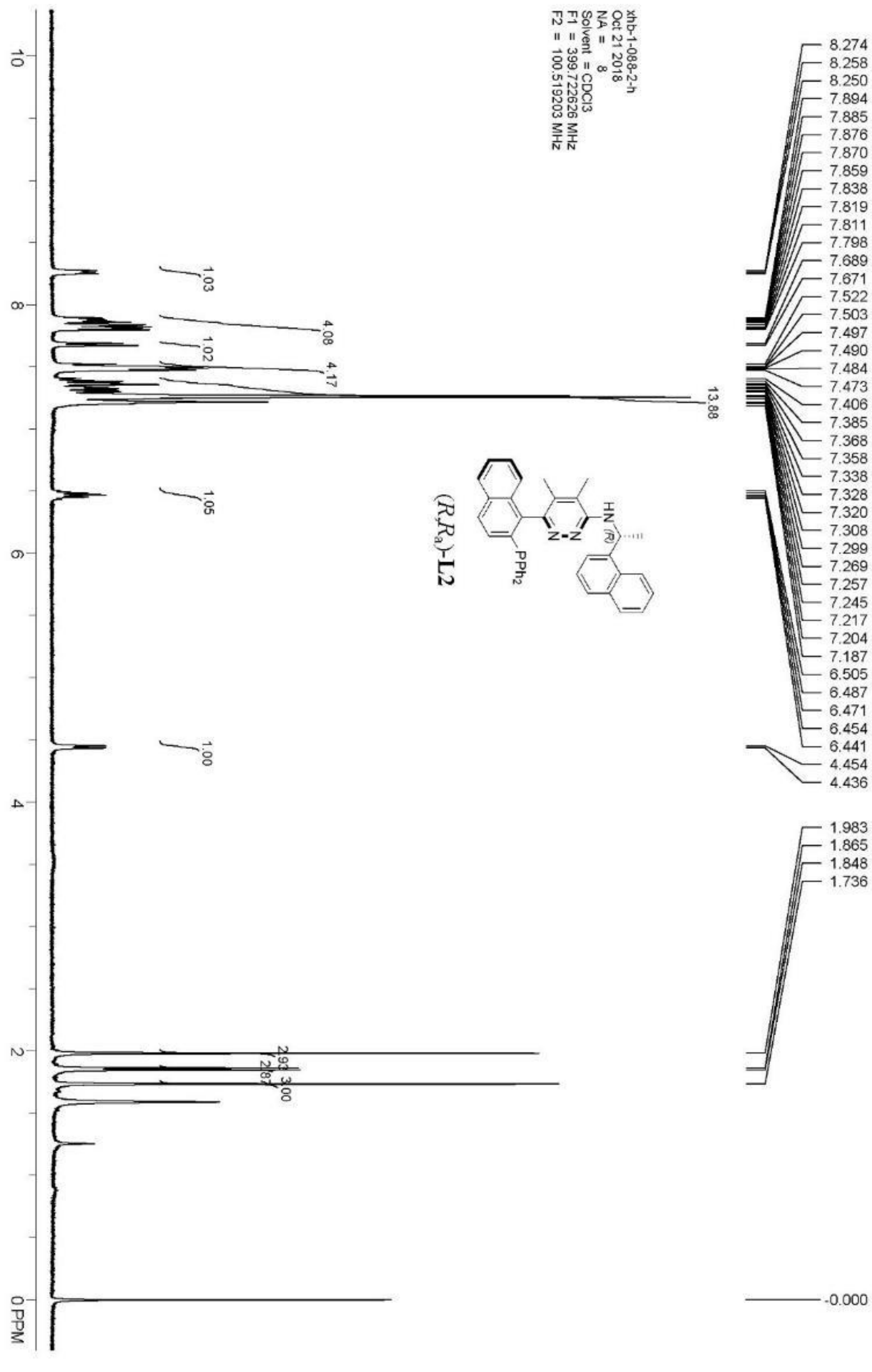

^{1}H NMR (400 MHz, CDCl3) spectrum for (R,R_a)-**L2**

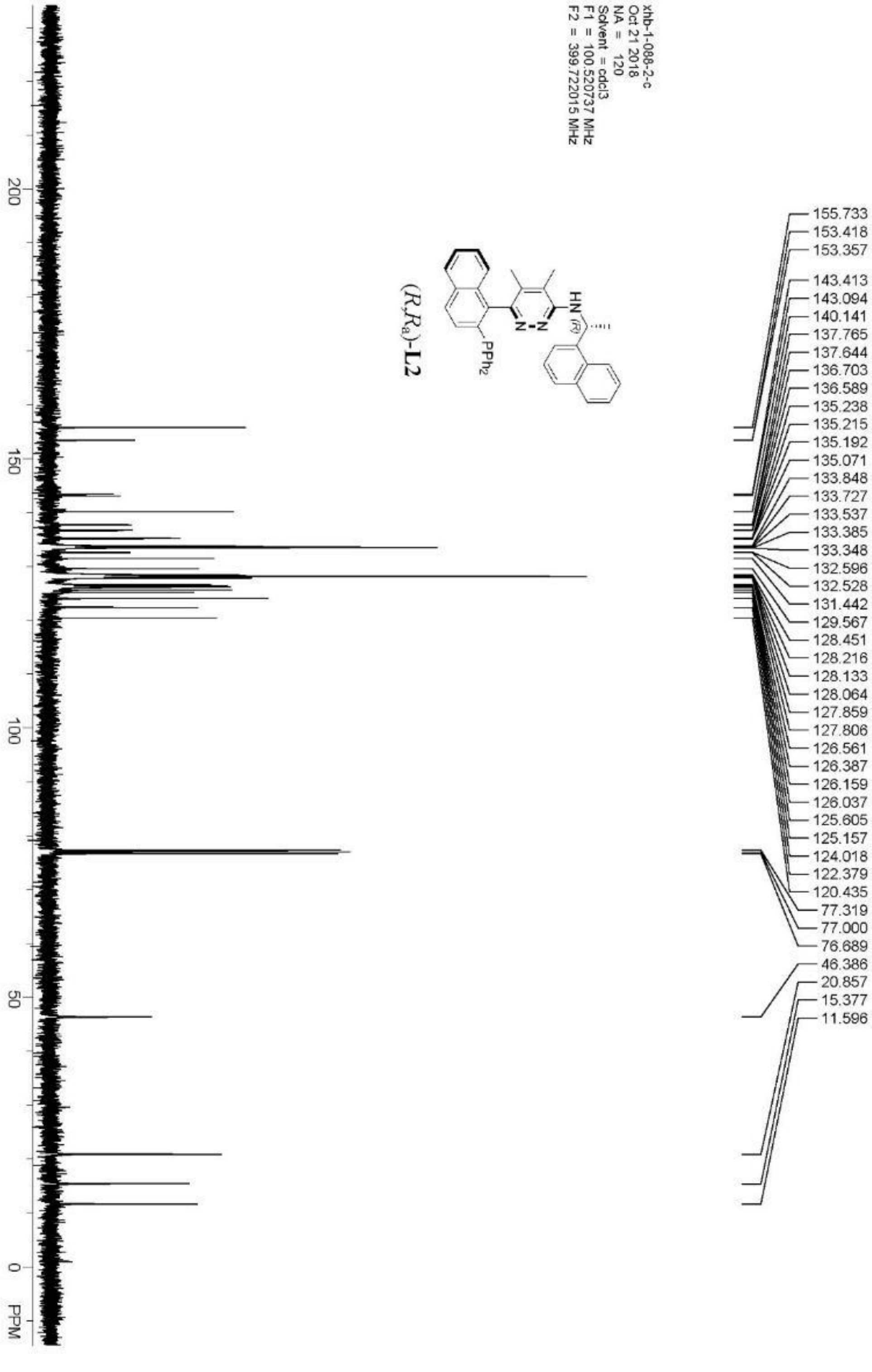

^{13}C NMR (400 MHz, CDCl$_3$) spectrum for (R,R_a)**-L2**

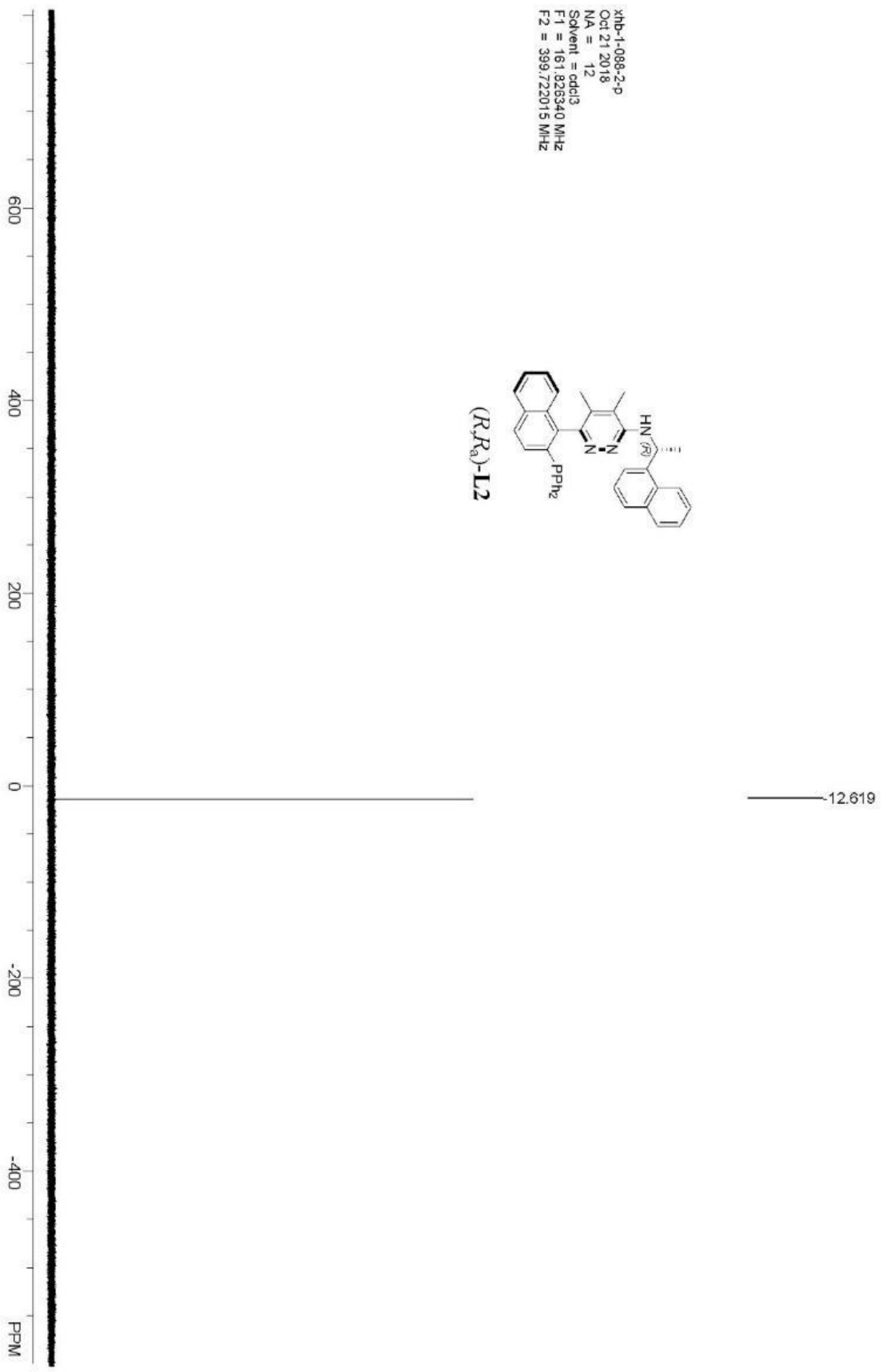

^{31}P NMR (162 MHz, $CDCl_3$) spectrum for (*R*,*R*a)-L2 (85% H_3PO_4 in D_2O was used as the external standard)

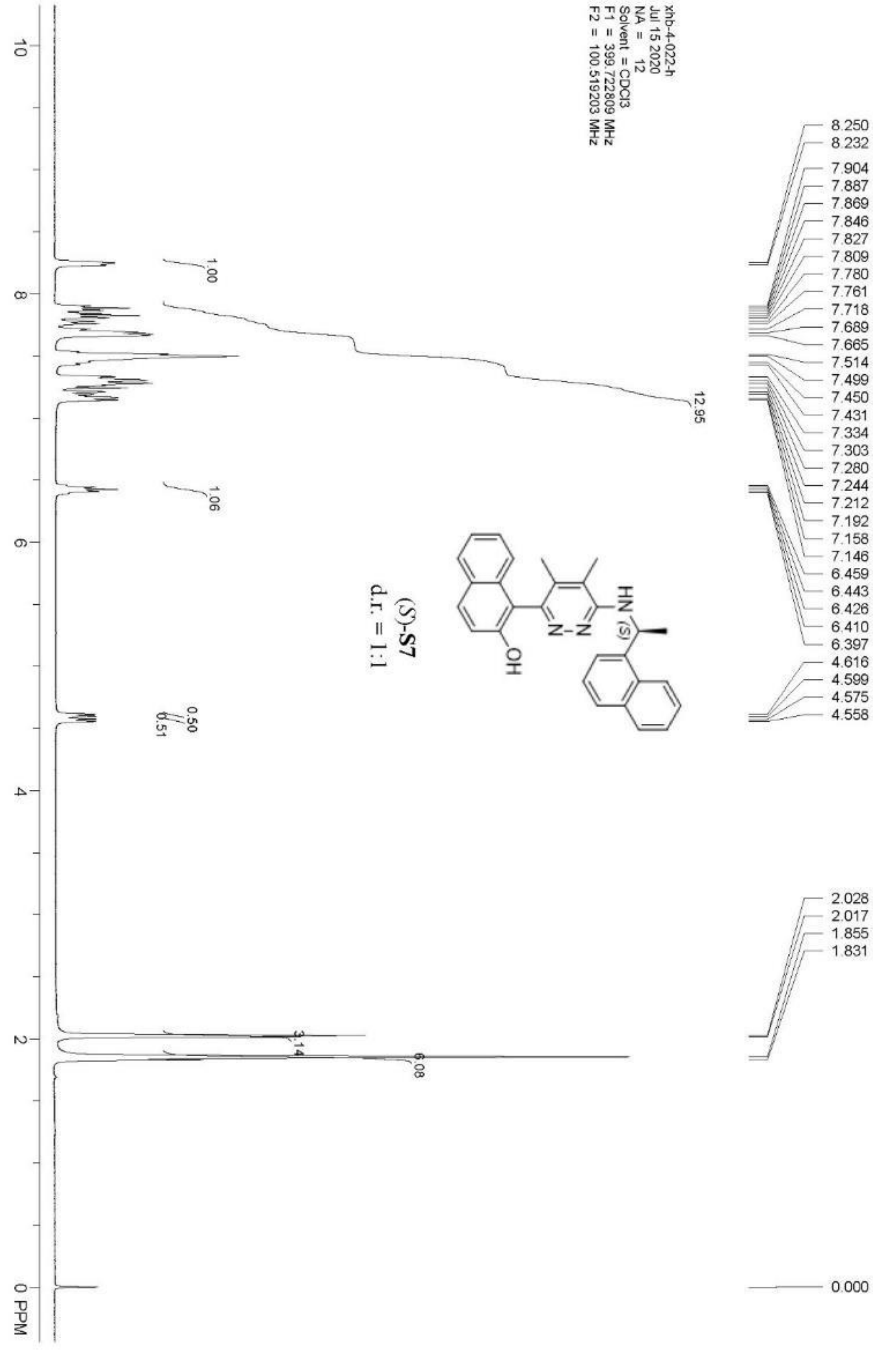

^{1}H NMR (400 MHz, CDCl3) spectrum for (*S*)-**S7**

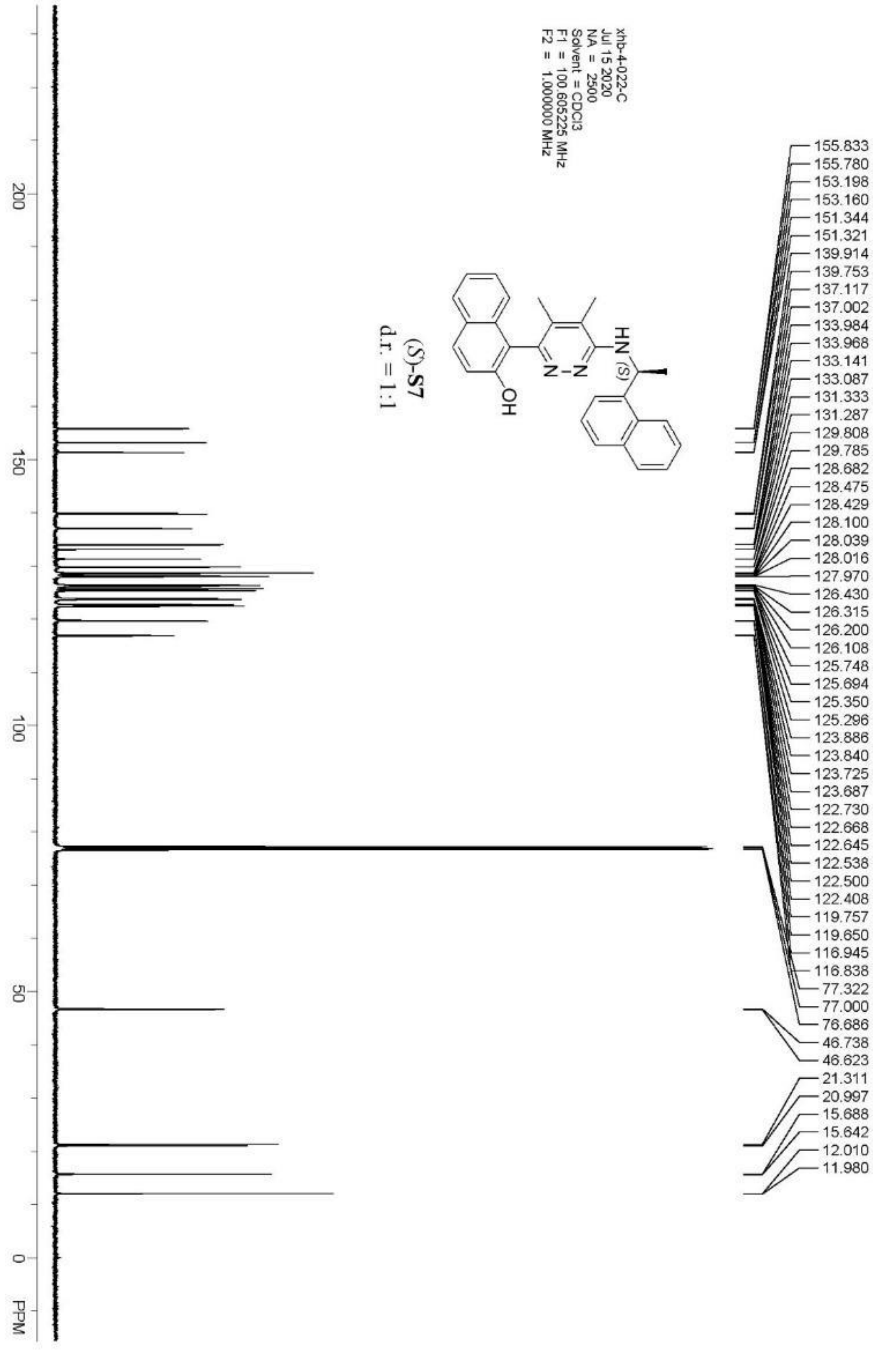

^{13}C NMR (400 MHz, CDCl$_3$) spectrum for (*S*)-S7

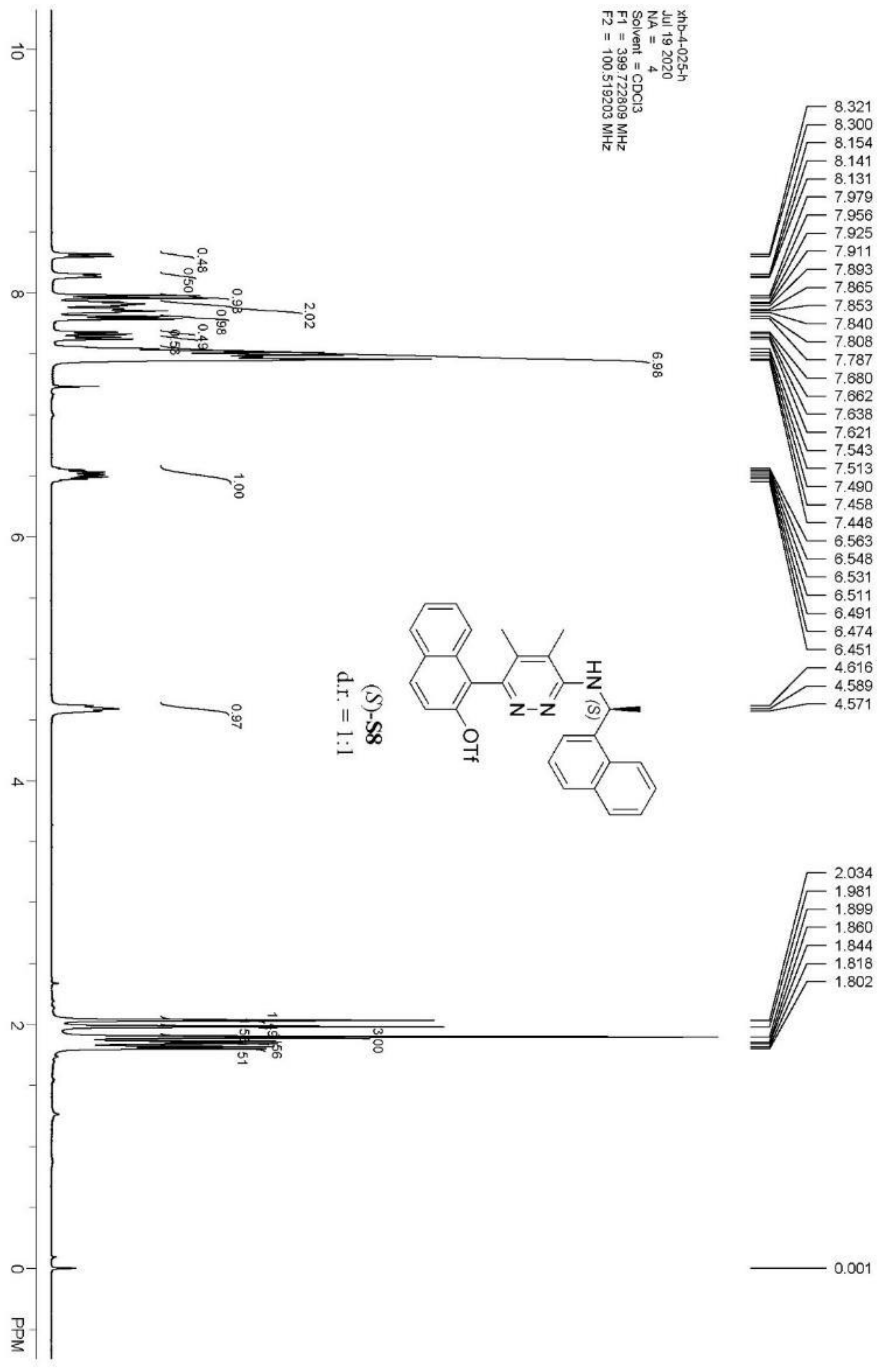

^{1}H NMR (400 MHz, CDCl3) spectrum for (*S*)**-S8**

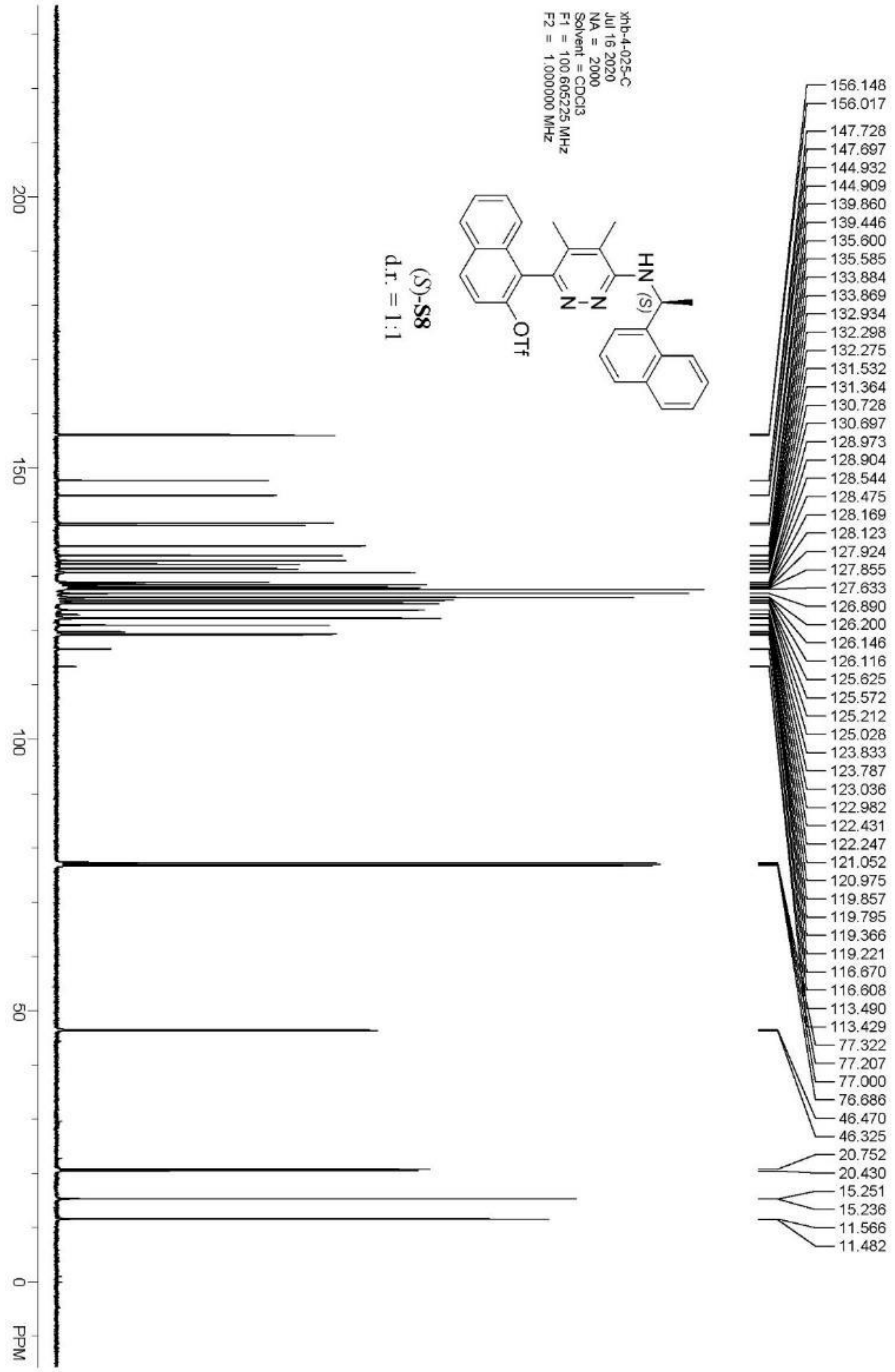

^{13}C NMR (400 MHz, $CDCl_3$) spectrum for (*S*)-S8

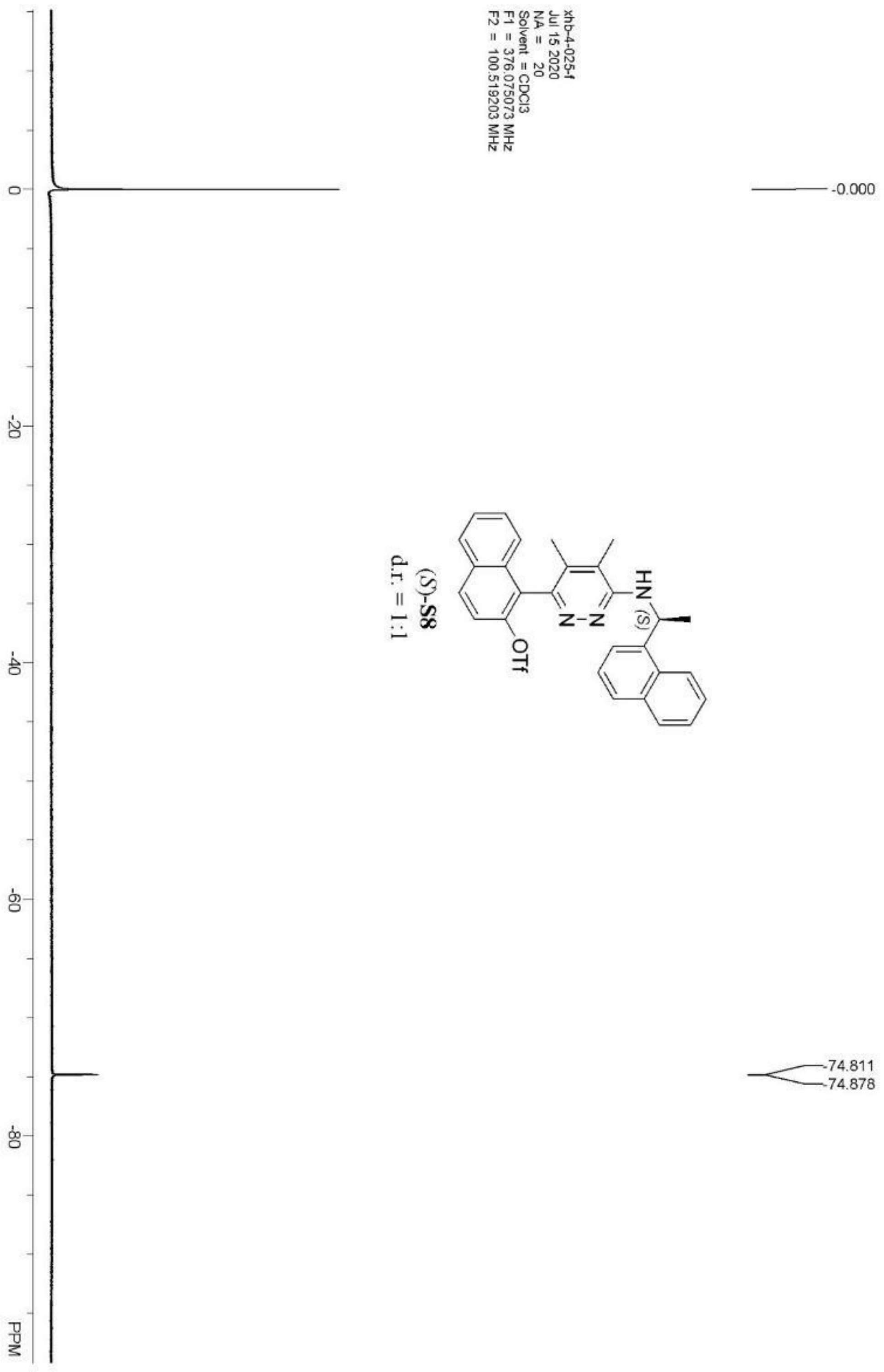

^{31}P NMR (162 MHz, $CDCl_3$) spectrum for (*S*)-S8 (85% H_3PO_4 in D_2O was used as the external standard)

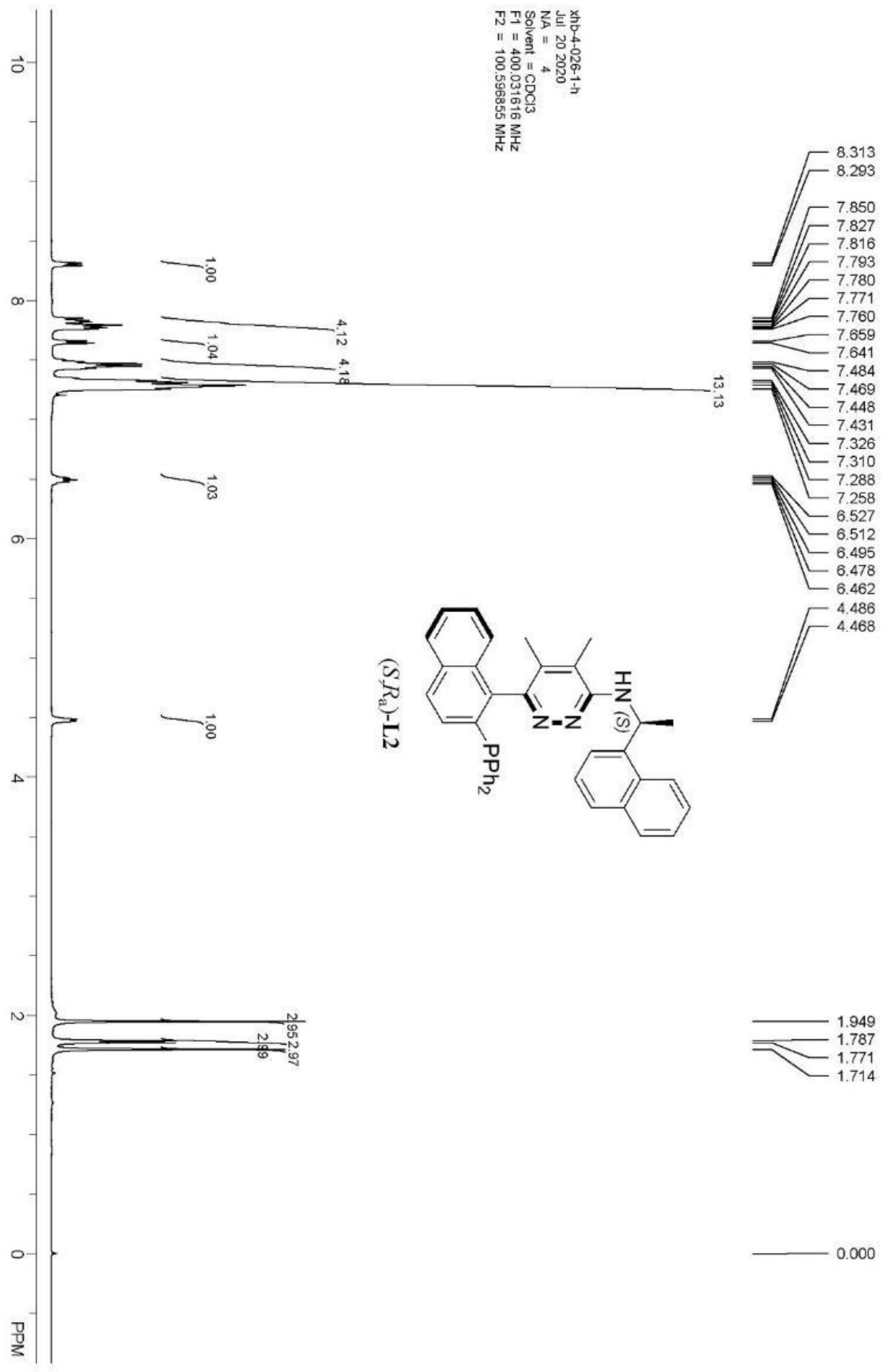

^{1}H NMR (400 MHz, CDCl3) spectrum for (*S*,*R*a)**-L2**

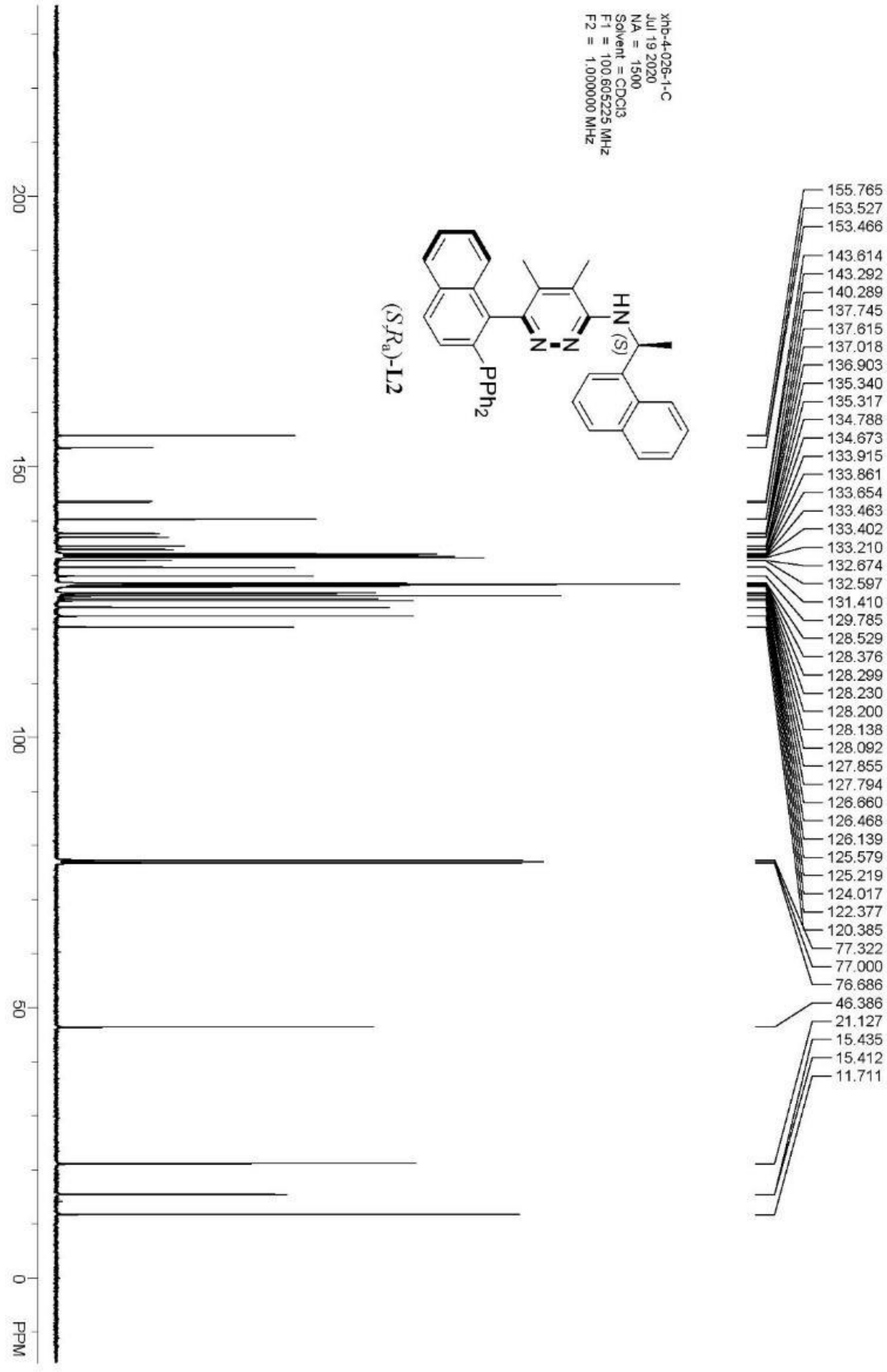

^{13}C NMR (400 MHz, $CDCl_3$) spectrum for (*S*,*R*a)-**L2**

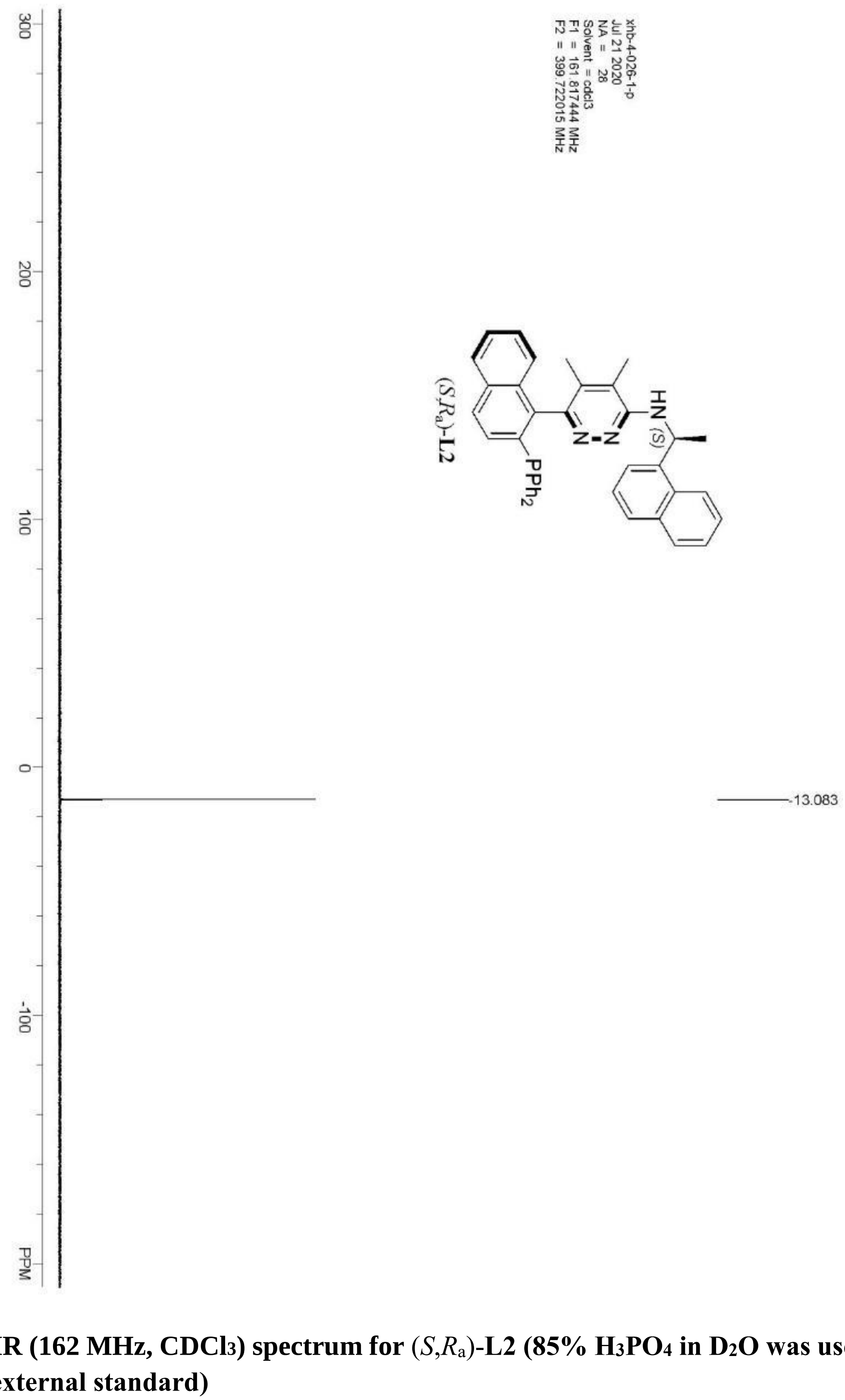

^{31}P NMR (162 MHz, $CDCl_3$) spectrum for (*S*,*R*a)-L2 (85% H_3PO_4 in D_2O was used as the external standard)

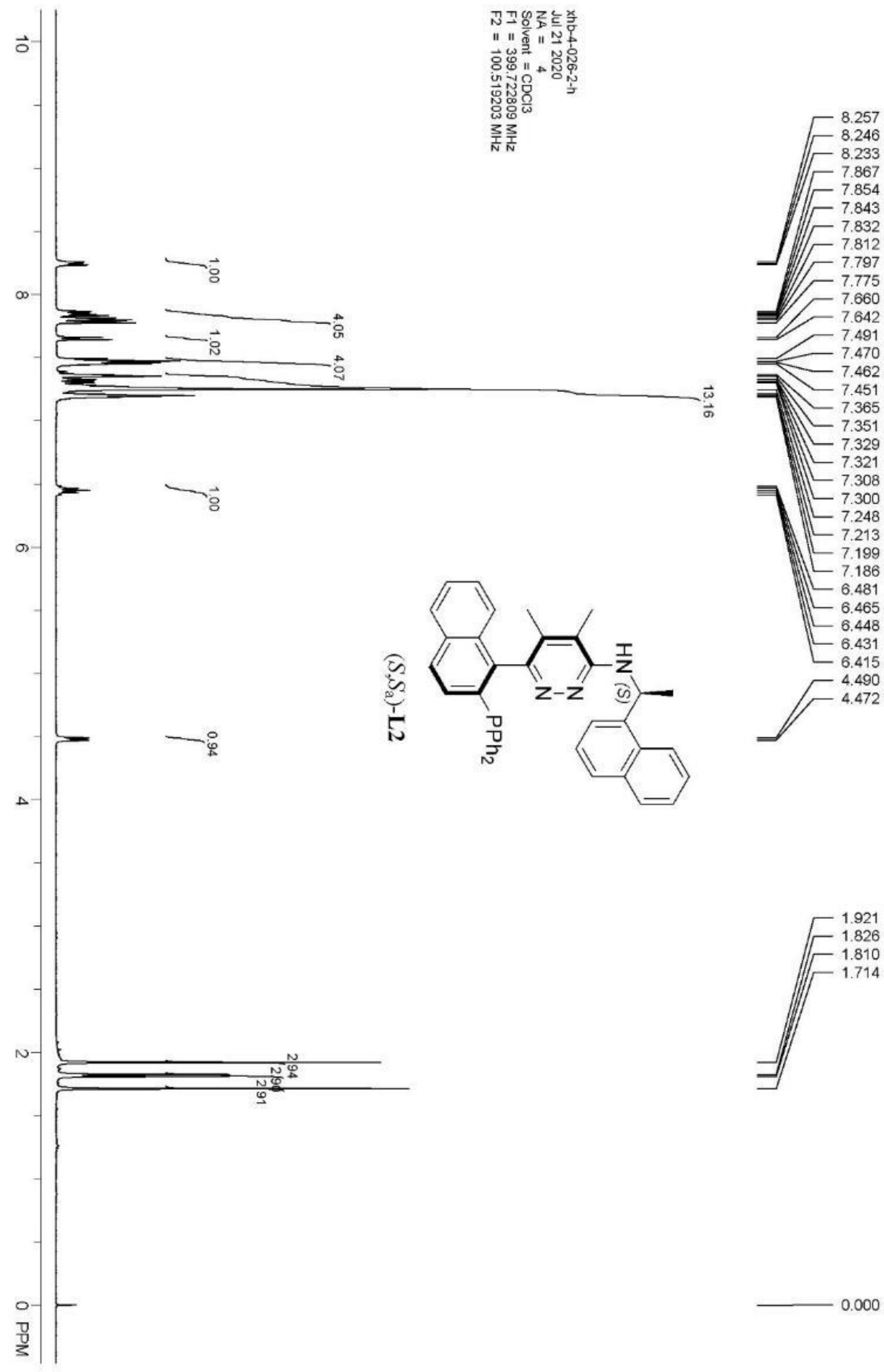

^{1}H NMR (400 MHz, CDCl3) spectrum for (*S*,*S*$_a$)-**L2**

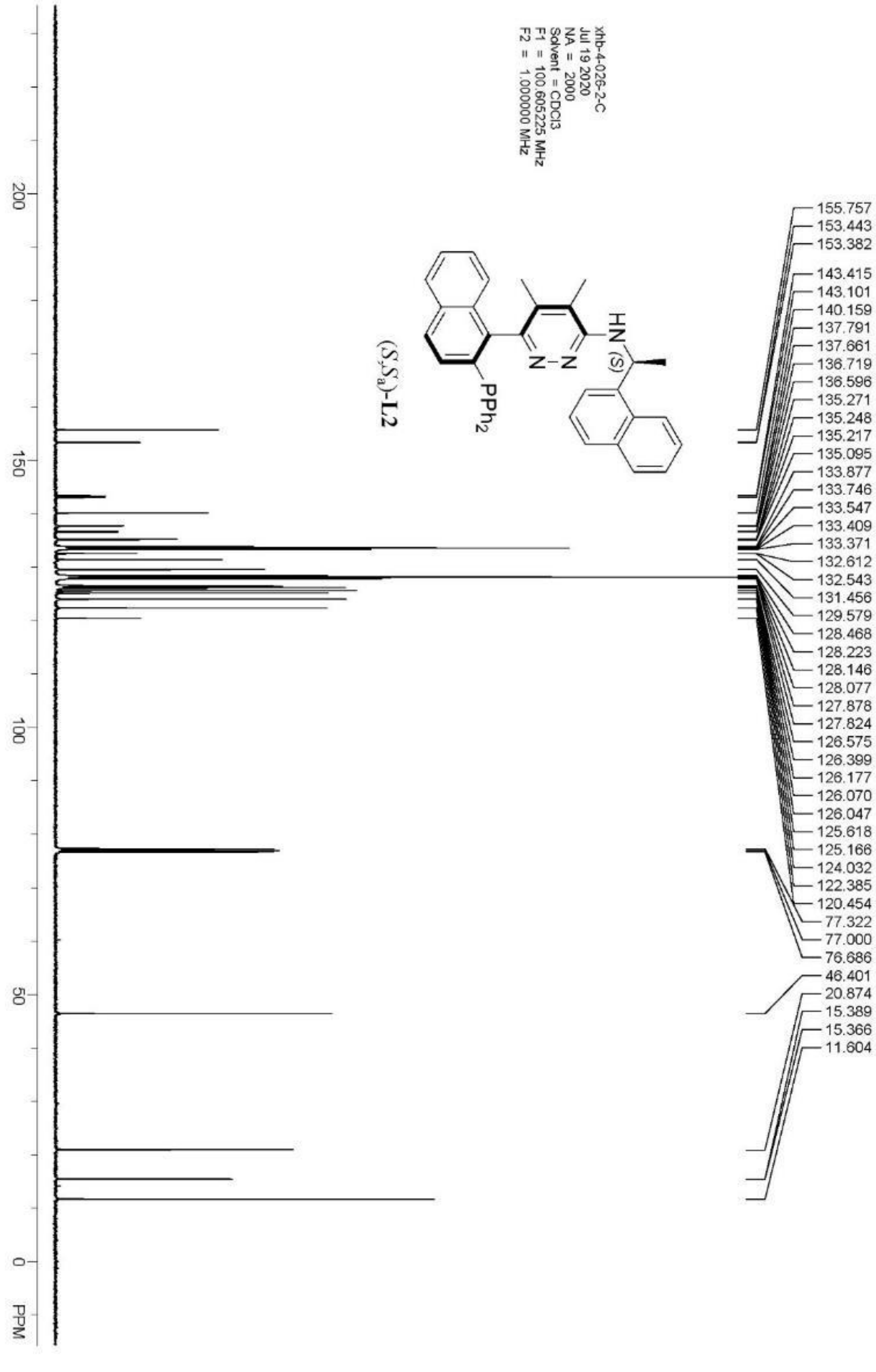

^{13}C NMR (400 MHz, CDCl$_3$) spectrum for (S,S_a)-**L2**

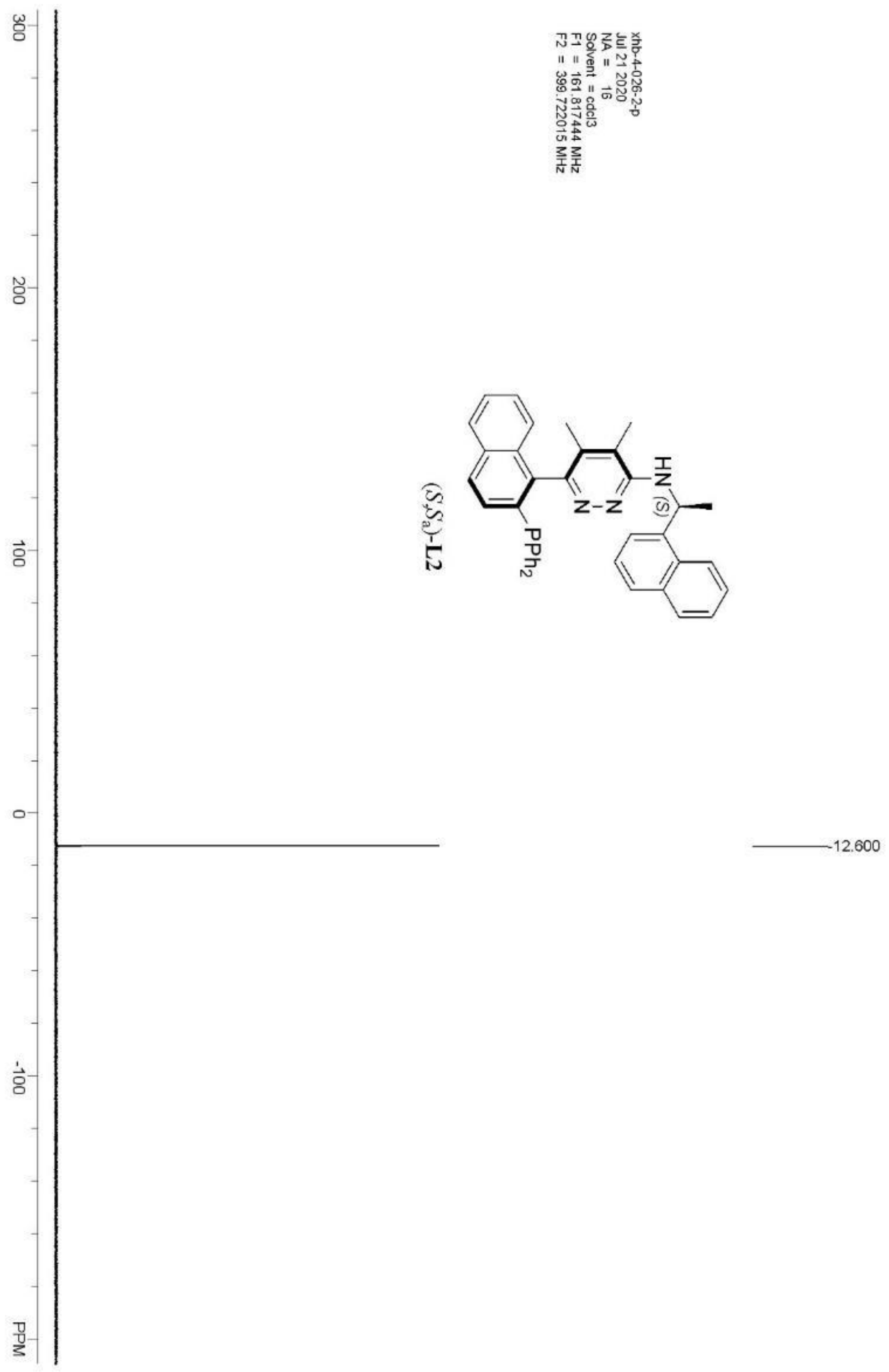

^{31}P NMR (162 MHz, $CDCl_3$) spectrum for (*S*,*S*a)-L2 (85% H_3PO_4 in D_2O was used as the external standard)

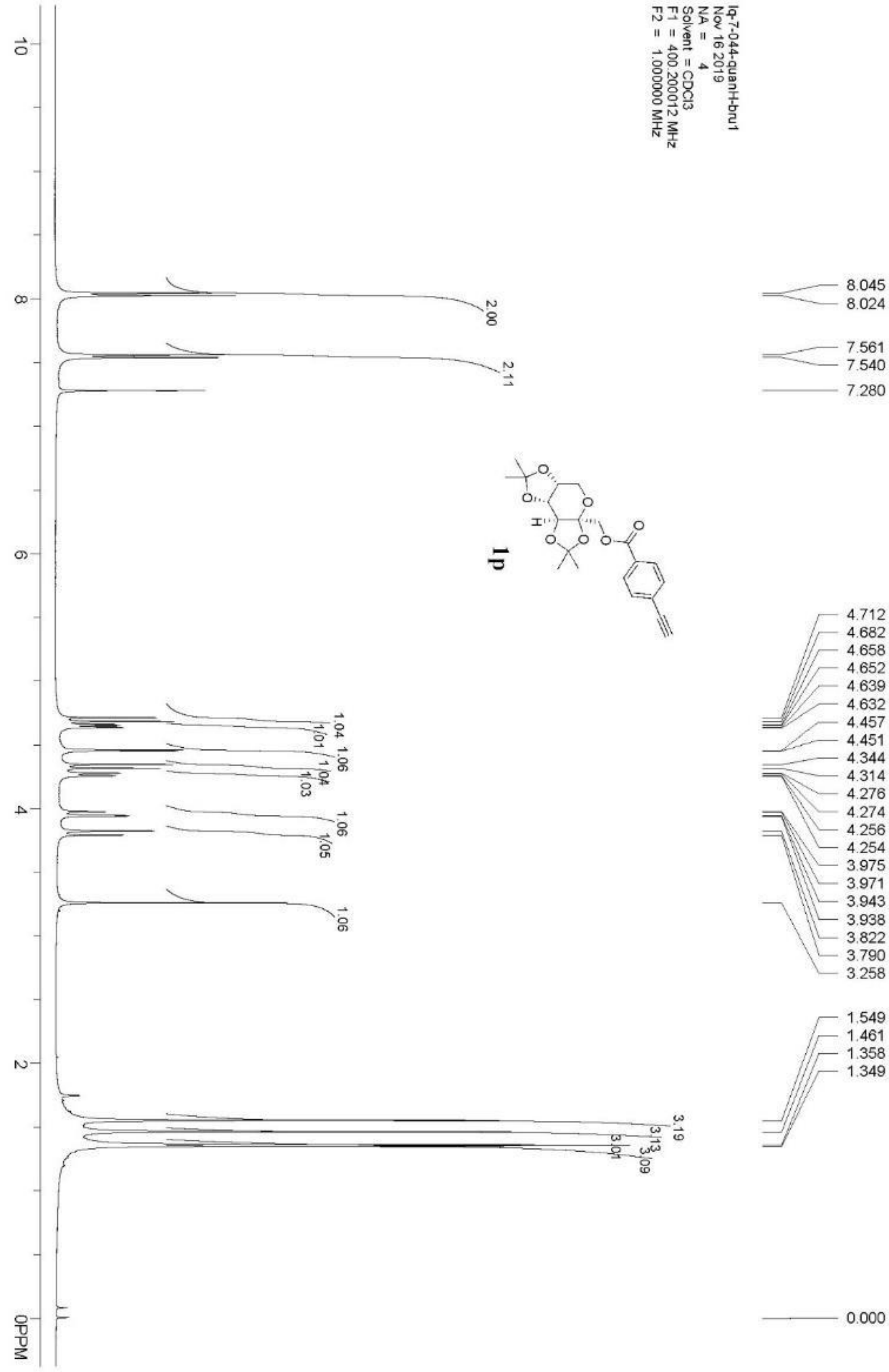

^{1}H NMR (400 MHz, CDCl3) spectrum for 1p

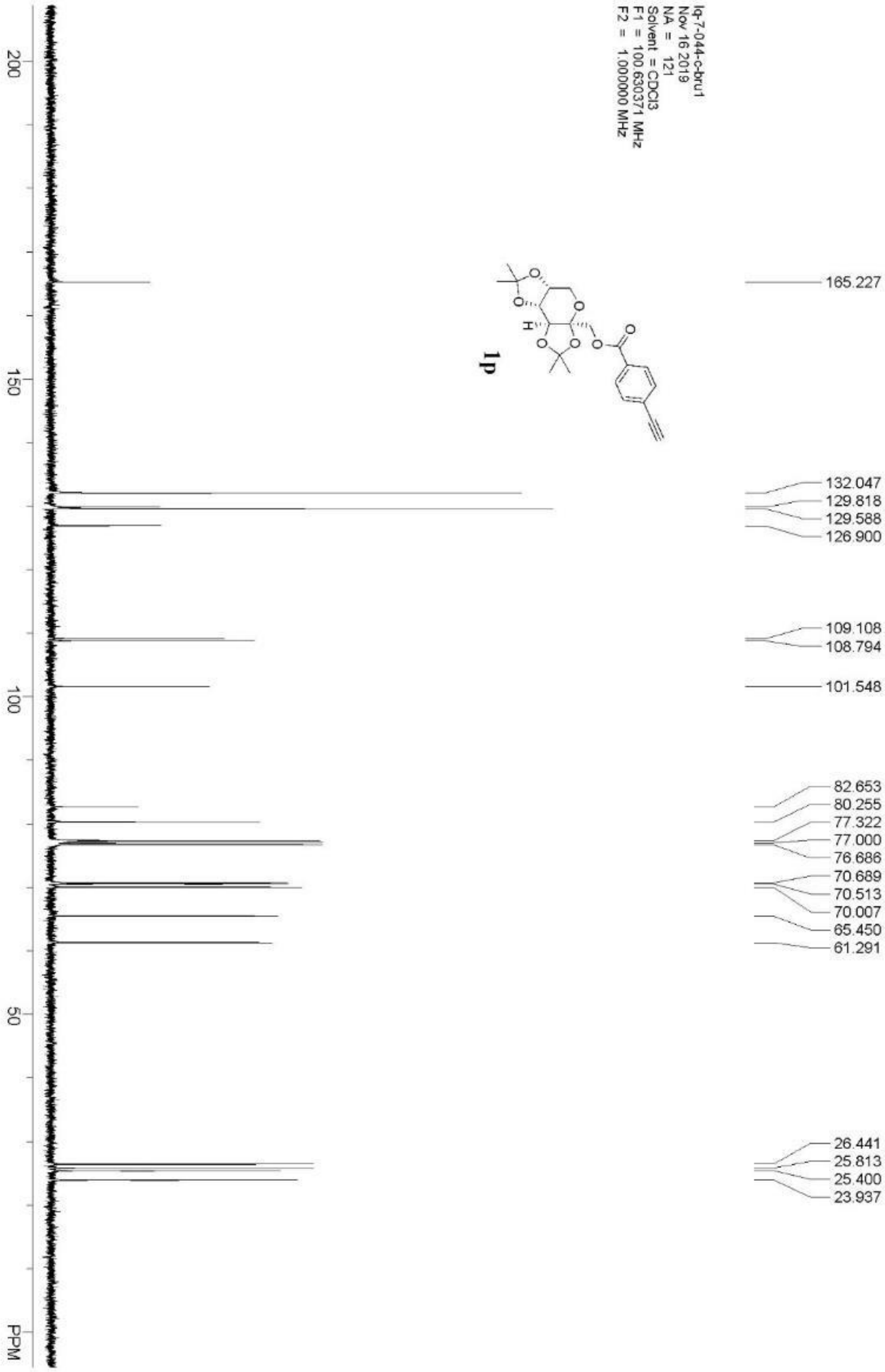

^{13}C NMR (400 MHz, $CDCl_3$) spectrum for 1p

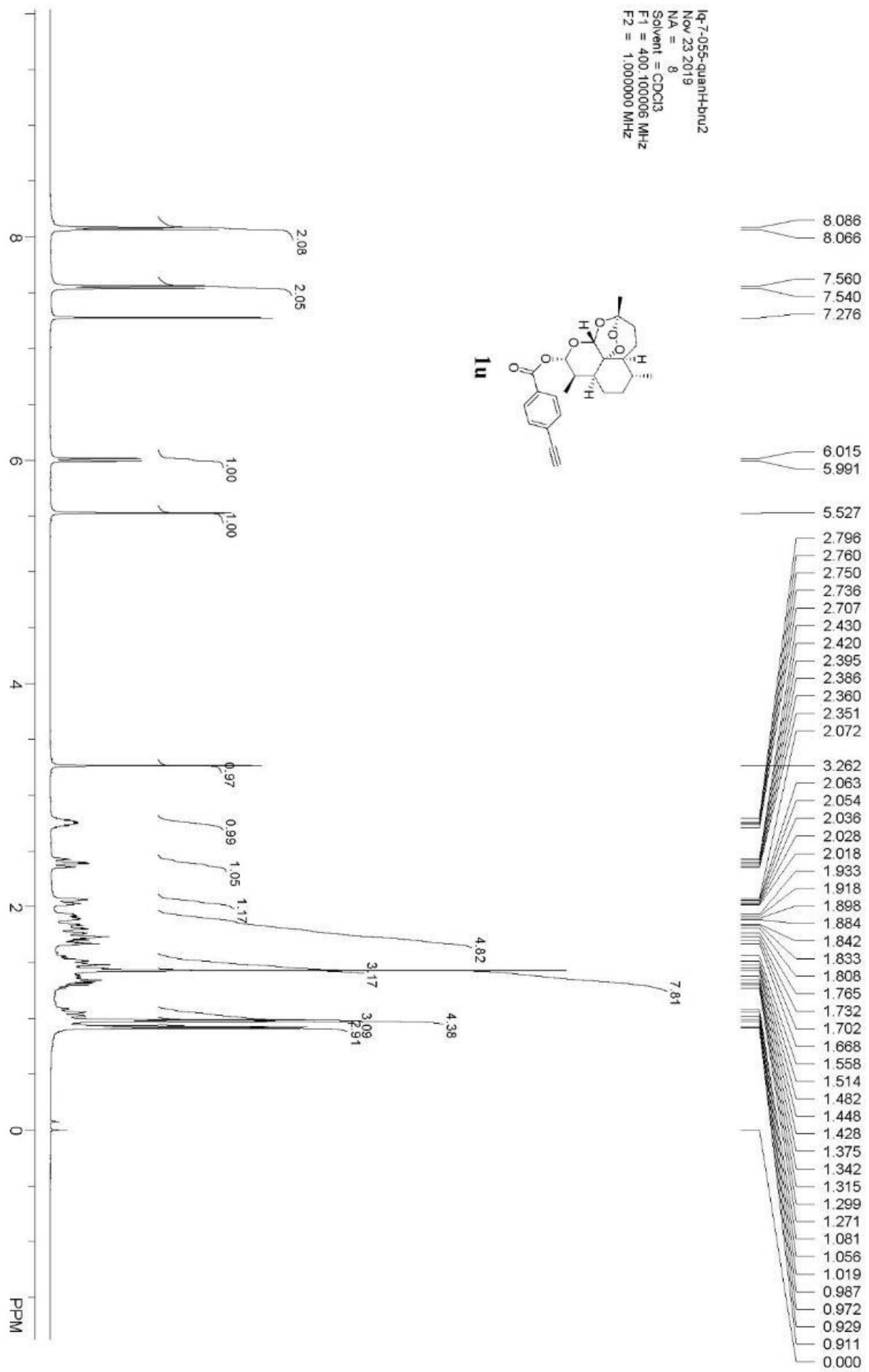

^{1}H NMR (400 MHz, CDCl3) spectrum for 1u

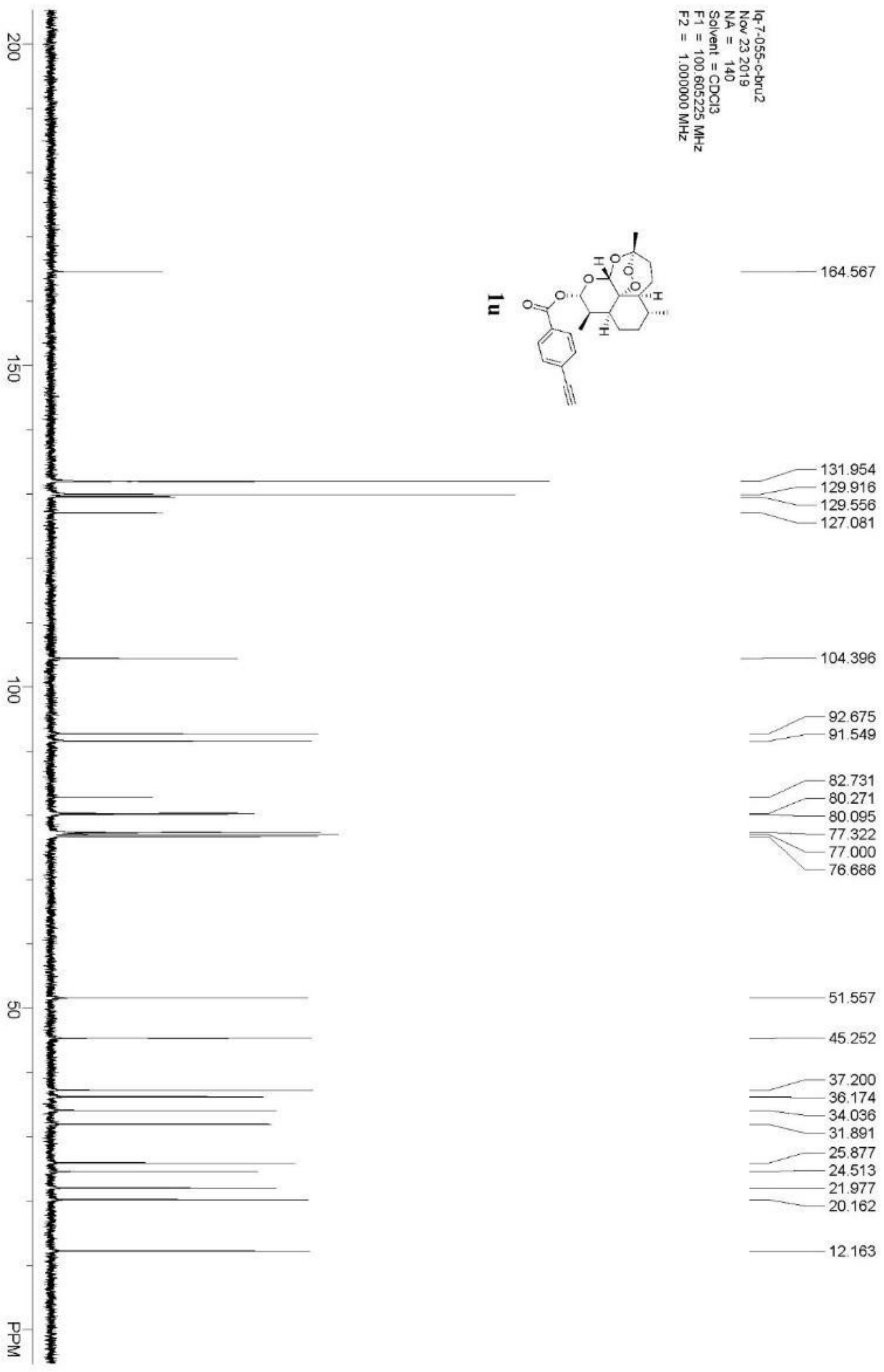

^{13}C NMR (400 MHz, $CDCl_3$) spectrum for 1u

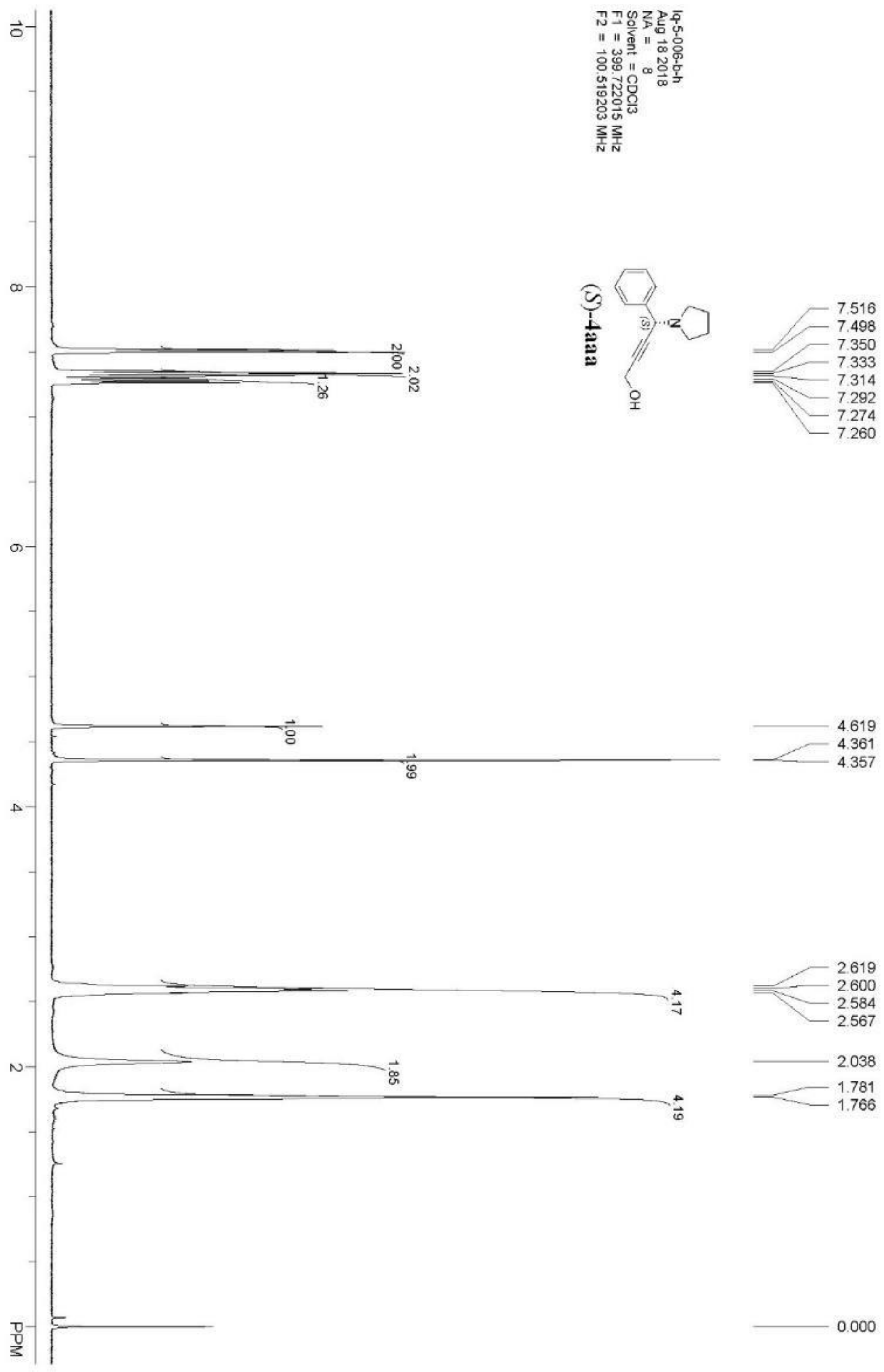

^{1}H NMR (400 MHz, CDCl3) spectrum for (*S*)**-4aaa**

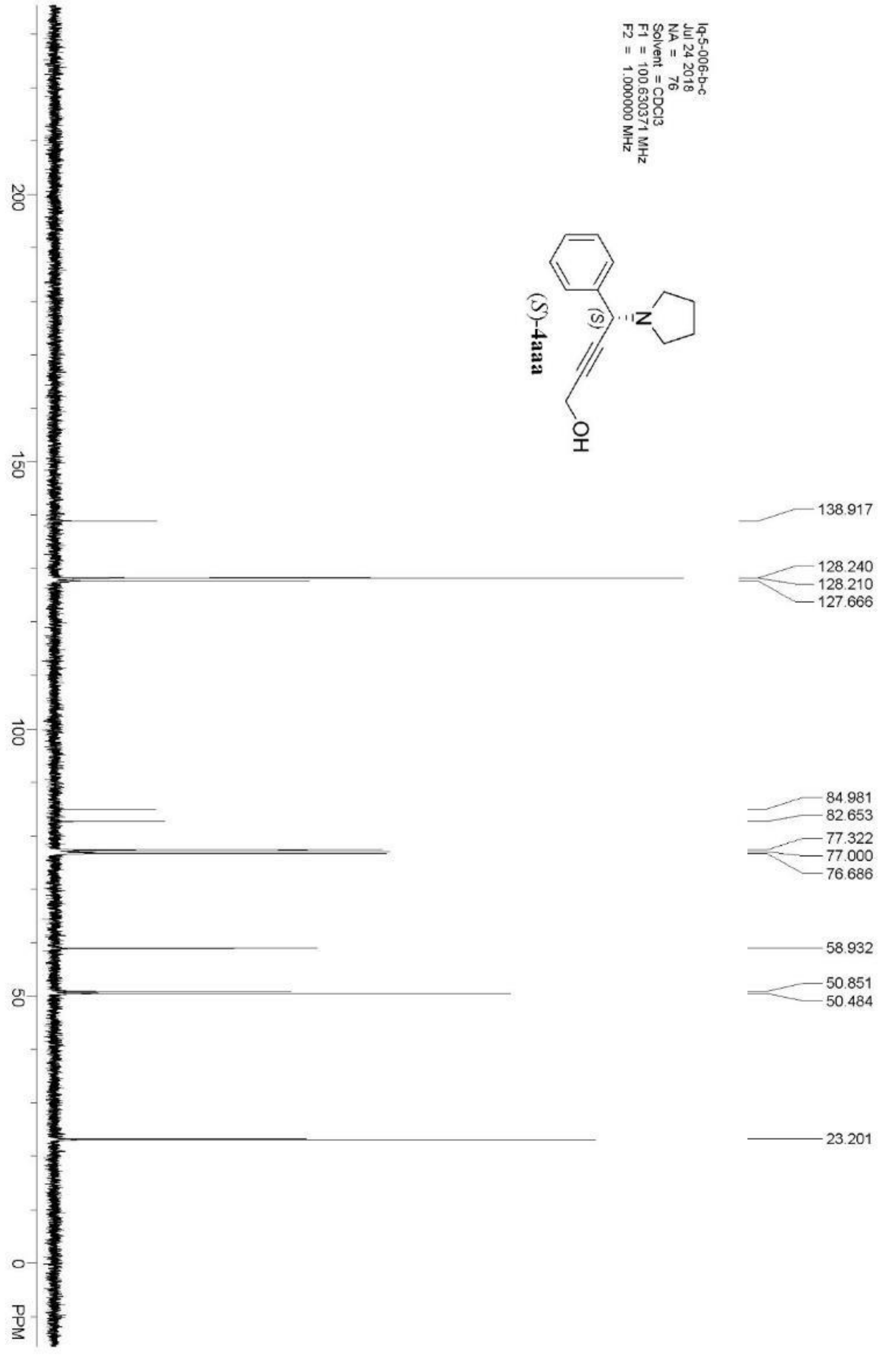

^{13}C NMR (400 MHz, CDCl$_3$) spectrum for (*S*)-4aaa

1q-5-006-B

实验时间：2018-07-24, 19:17:31　　　　　　　　　　报告时间：2018-07-25, 8:50:35
谱图文件:H:\2018-07-24\1q-5-006-b-od-h-95+5-1.2-214.org

实验内容简介：
OD-H　　95:5
214nm　　1.2ml/min

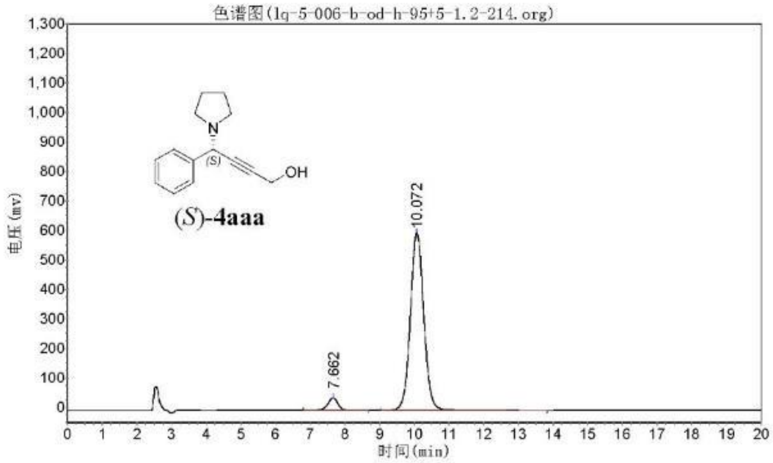

分析结果表

峰号	峰名	保留时间	峰高	峰面积	含量
1		7.662	42433.332	852280.438	4.9752
2		10.072	600913.500	16278439.000	95.0248
总计			643346.832	17130719.438	100.0000

HPLC spectrum for (*S*)-4aaa

LQ-3-115

实验时间：2018-7-24,20:01:27　　　　　　　　　报告时间：2018-7-25,8:54:57
谱图文件:H:\2018-07-24\lq-3-115-od-h-95+5-1.2-214.org

实验内容简介：
OD-H　　95:5
214nm　　1.2ml/min

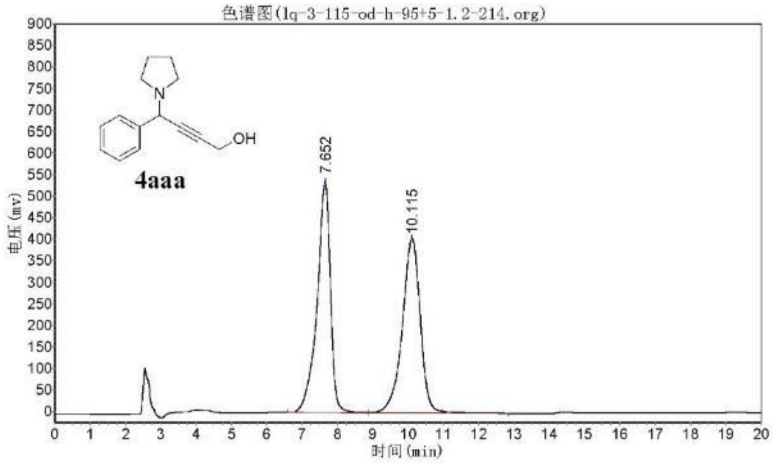

分析结果表

峰号	峰名	保留时间	峰高	峰面积	含量
1		7.652	535102.625	14159724.000	49.7128
2		10.115	404355.750	14323304.000	50.2872
**总计**			939458.375	28483028.000	100.0000

HPLC spectrum for (±)-4aaa

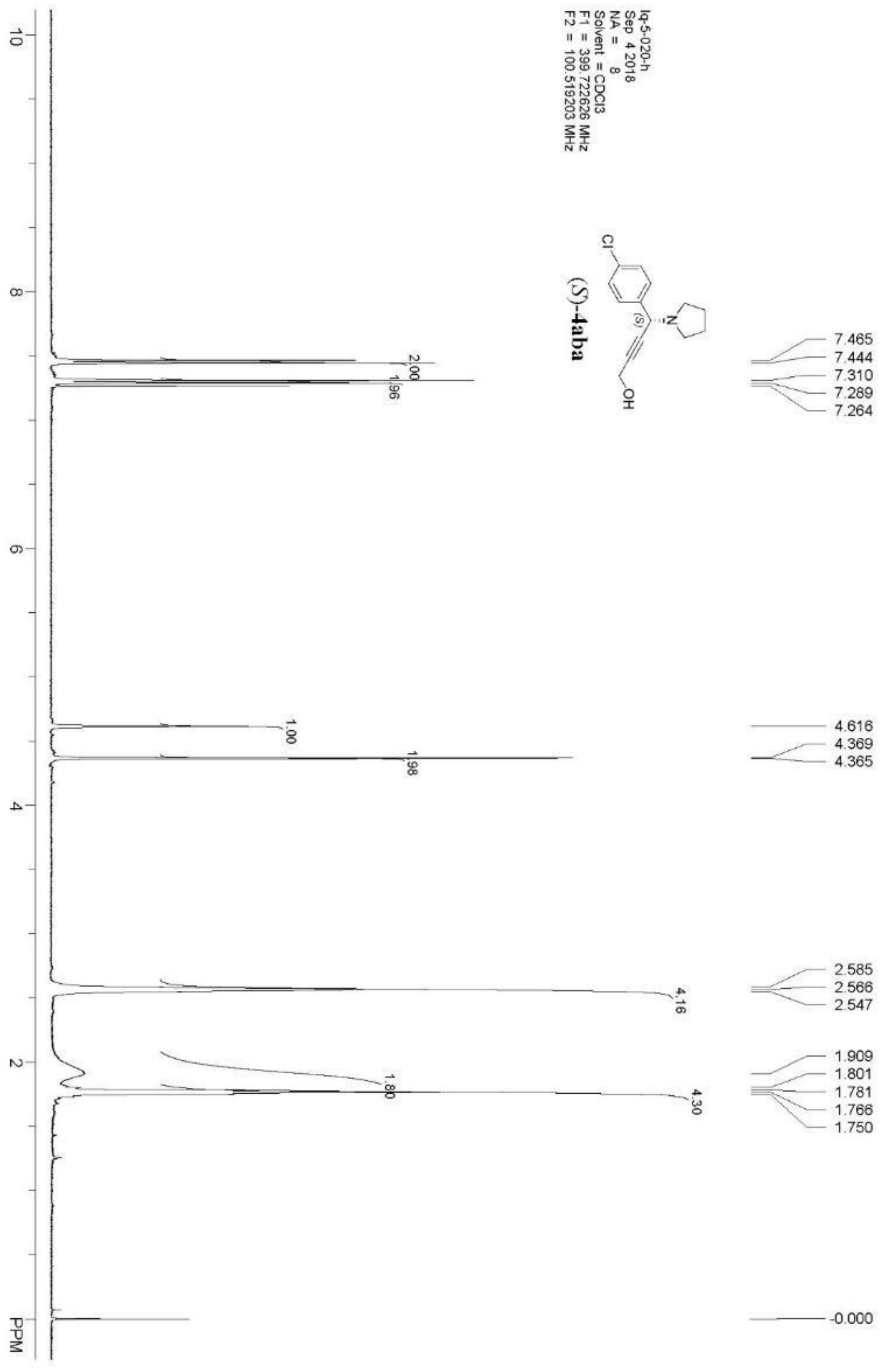

¹H NMR (400 MHz, CDCl3) spectrum for (*S*)-4aba

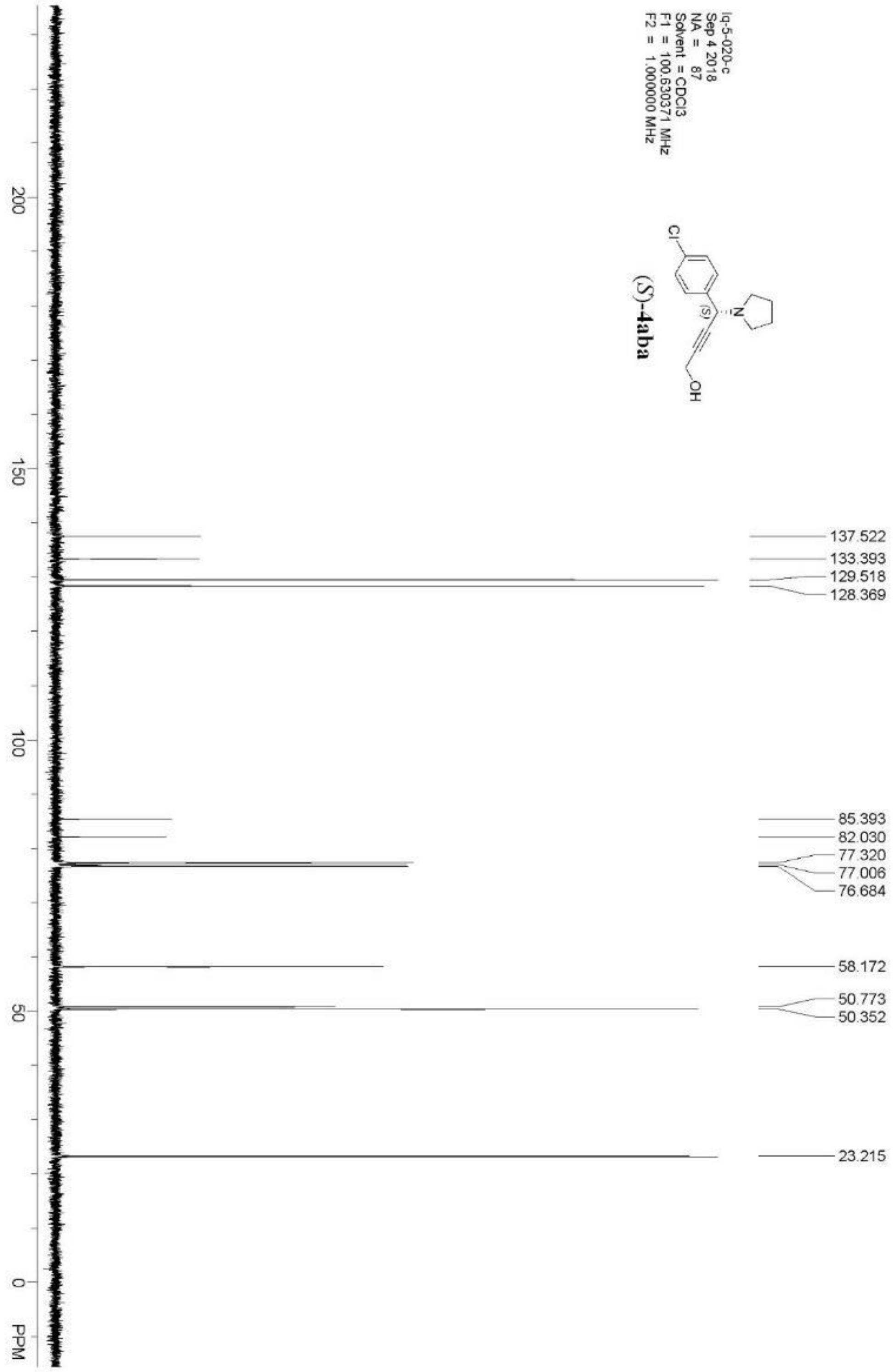

^{13}C NMR (400 MHz, $CDCl_3$) spectrum for (*S*)-4aba

lq-5-020

实验时间：2018/9/4, 13:07:03　　　　　　　　　　　　报告时间：2018/9/4, 13:21:17
谱图文件:D:\data\slf\lq\2018-9-4\lq-5-020-ad-h-80+20-1-214.org

实验内容简介：
AD-H　800:20
214nm　1.0ml/min

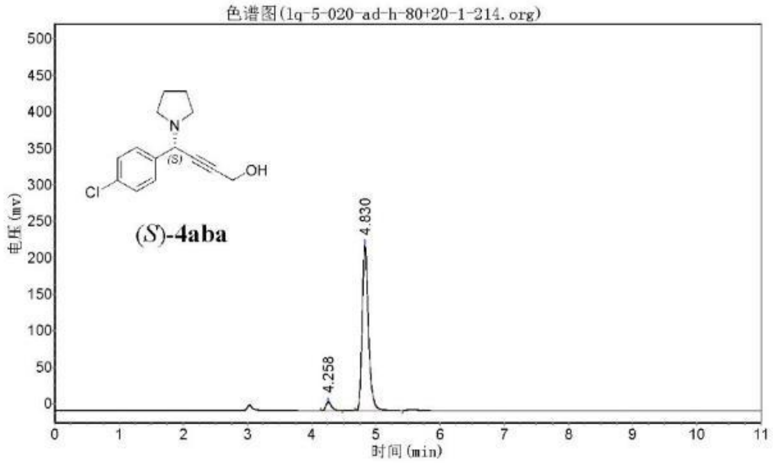

分析结果表

峰号	峰名	保留时间	峰高	峰面积	含量
1		4.258	12089.413	72704.969	4.4809
2		4.830	228143.281	1549861.625	95.5191
**总计**			240232.694	1622566.594	100.0000

HPLC spectrum for (*S*)-4aba

lq-5-018

实验时间：2018/9/4, 13:19:10　　　　报告时间：2018/9/4, 13:30:57
谱图文件:D:\data\slf\lq\2018-9-4\lq-5-018-ad-h-80+20-1-214.org

实验内容简介：
AD-H　80:20
214nm　1.0ml/min

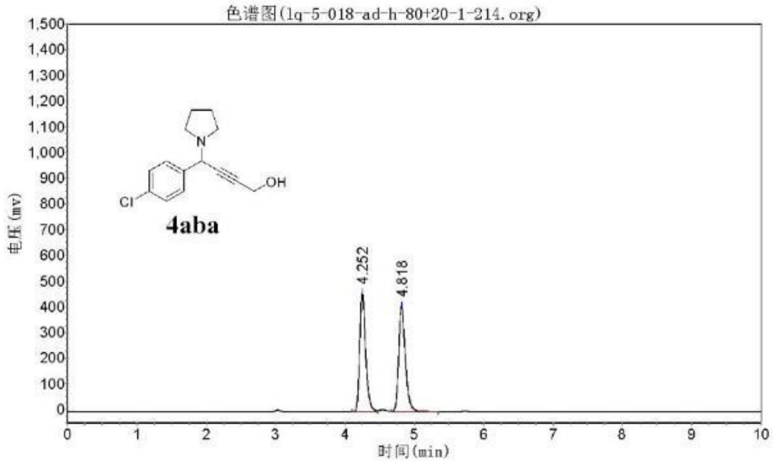

分析结果表

峰号	峰名	保留时间	峰高	峰面积	含量
1		4.252	462757.688	2750472.250	49.6492
2		4.818	412197.938	2789334.750	50.3508
**总计**			874955.625	5539807.000	100.0000

HPLC spectrum for (±)-4aba

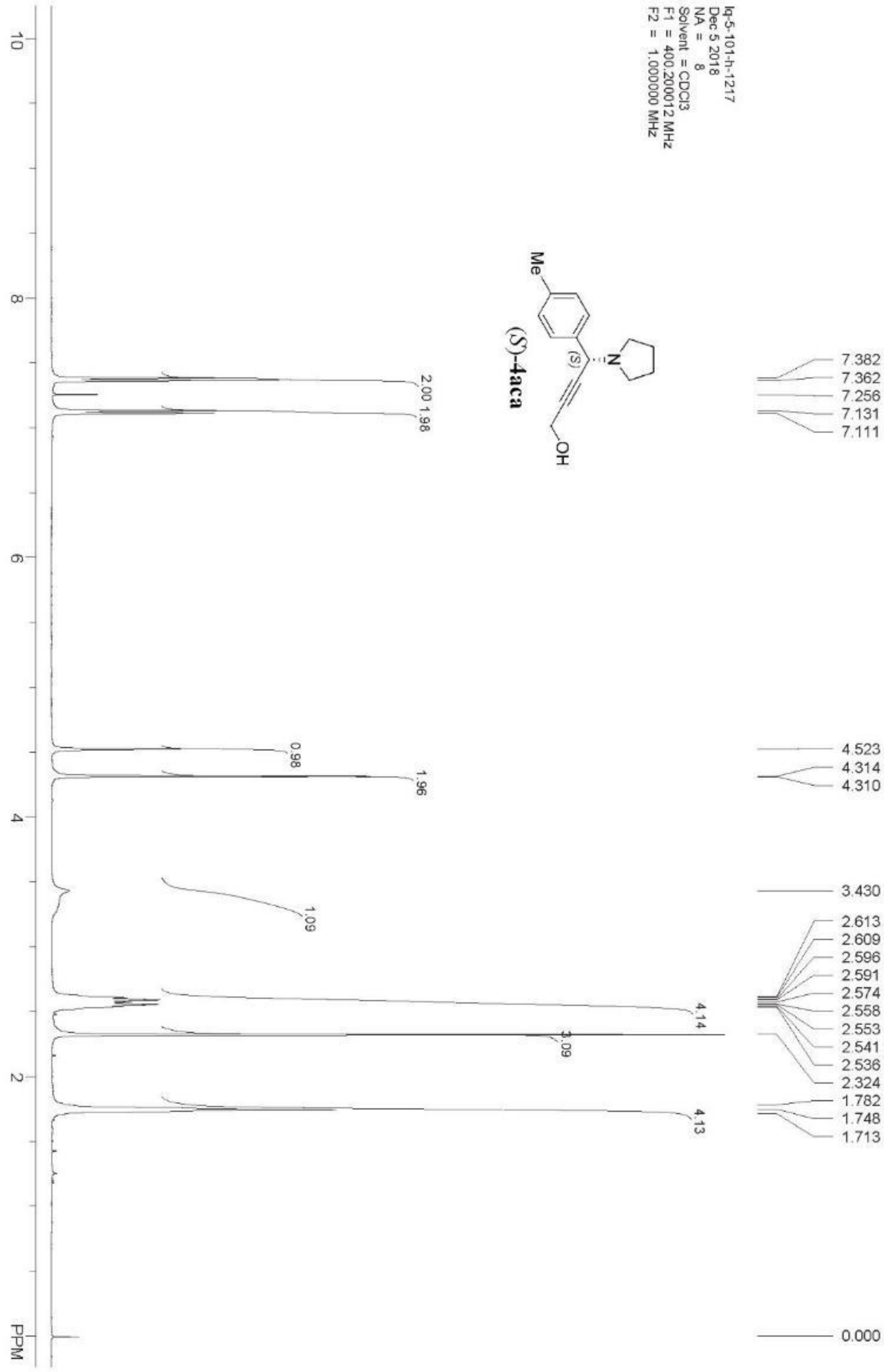

^{1}H NMR (400 MHz, CDCl3) spectrum for (*S*)-4aca

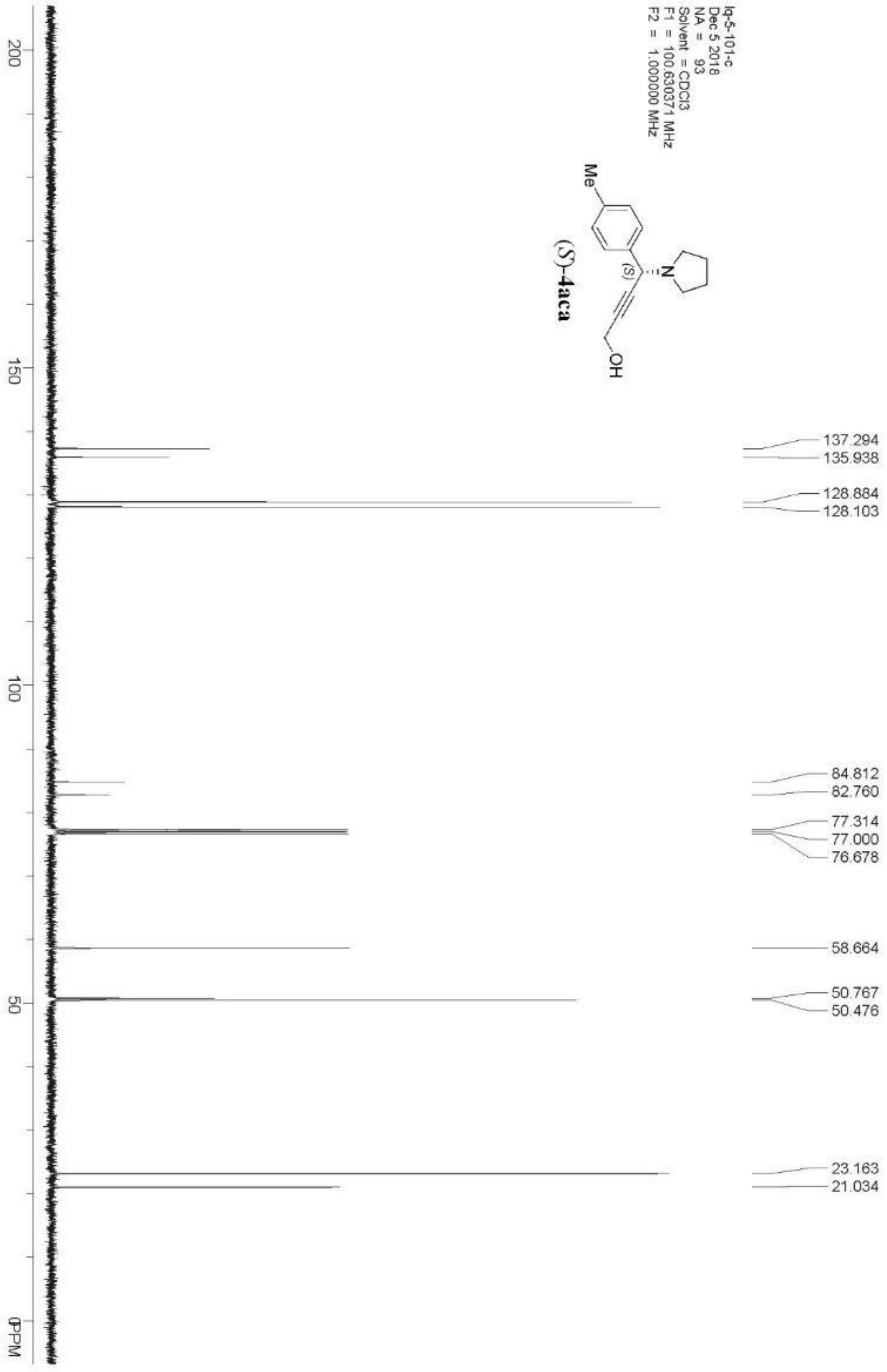

^{13}C NMR (400 MHz, CDCl$_3$) spectrum for (*S*)-4aca

Empower 3 SOFTWARE

Injection Summary Report

SAMPLE INFORMATION

Sample Name:	lq-5-101-ad-h-95-5-1.0-214	Acquired By:	System
Sample Type:	Unknown	Sample Set Name:	
Vial:	1	Acq. Method Set:	HPLC
Injection #:	2	Processing Method:	20181124
Injection Volume:	4.00 ul	Channel Name:	W2489 ChA
Run Time:	30.0 Minutes	Proc. Chnl. Descr.:	W2489 ChA 214nm
Date Acquired:	12/5/2018 3:33:34 PM CST		
Date Processed:	12/5/2018 3:56:20 PM CST		

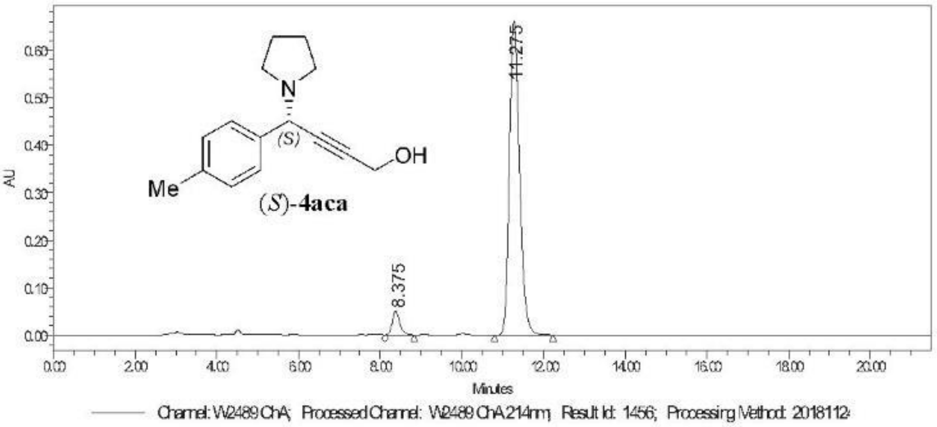

Processed Channel Descr.: W2489 ChA 214nm

	Processed Channel Descr.	RT	Area	%Area	Height
1	W2489 ChA 214nm	8.375	619813	5.19	49907
2	W2489 ChA 214nm	11.275	11323377	94.81	662622

Reported by User: System
Report Method: Injection Summary Report
Report Method ID: 1374 1374
Page: 1 of 1

Project Name: HPLC
Date Printed:
12/5/2018
11:26:47 PM PRC

HPLC spectrum for (*S*)-4aca

Empower 3 SOFTWARE

Injection Summary Report

SAMPLE INFORMATION

Sample Name:	xhb-1-110-ad-h-95-5-1.0-214	Acquired By:	System
Sample Type:	Unknown	Sample Set Name:	
Vial:	1	Acq. Method Set:	HPLC
Injection #:	4	Processing Method:	20181124
Injection Volume:	5.00 ul	Channel Name:	W2489 ChA
Run Time:	30.0 Minutes	Proc. Chnl. Descr.:	W2489 ChA 214nm
Date Acquired:	12/5/2018 4:13:48 PM CST		
Date Processed:	12/5/2018 4:36:20 PM CST		

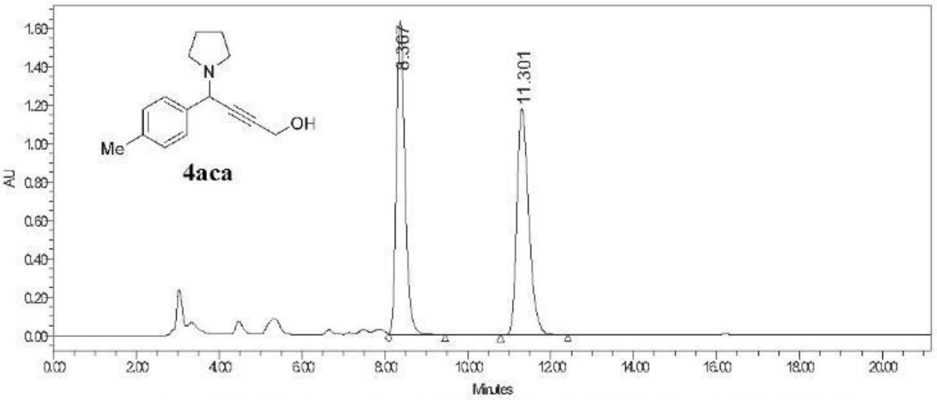

Channel: W2489 ChA; Processed Channel: W2489 ChA 214nm; Result Id: 1463; Processing Method: 2018112

Processed Channel Descr.: W2489 ChA 214nm

	Processed Channel Descr.	RT	Area	%Area	Height
1	W2489 ChA 214nm	8.367	23453756	50.01	1633055
2	W2489 ChA 214nm	11.301	23446649	49.99	1179797

Reported by User: System
Report Method: Injection Summary Report
Report Method ID: 1374 1374
Page: 1 of 1

Project Name: HPLC
Date Printed:
12/5/2018
11:27:25 PM PRC

HPLC spectrum for (±)-4aca

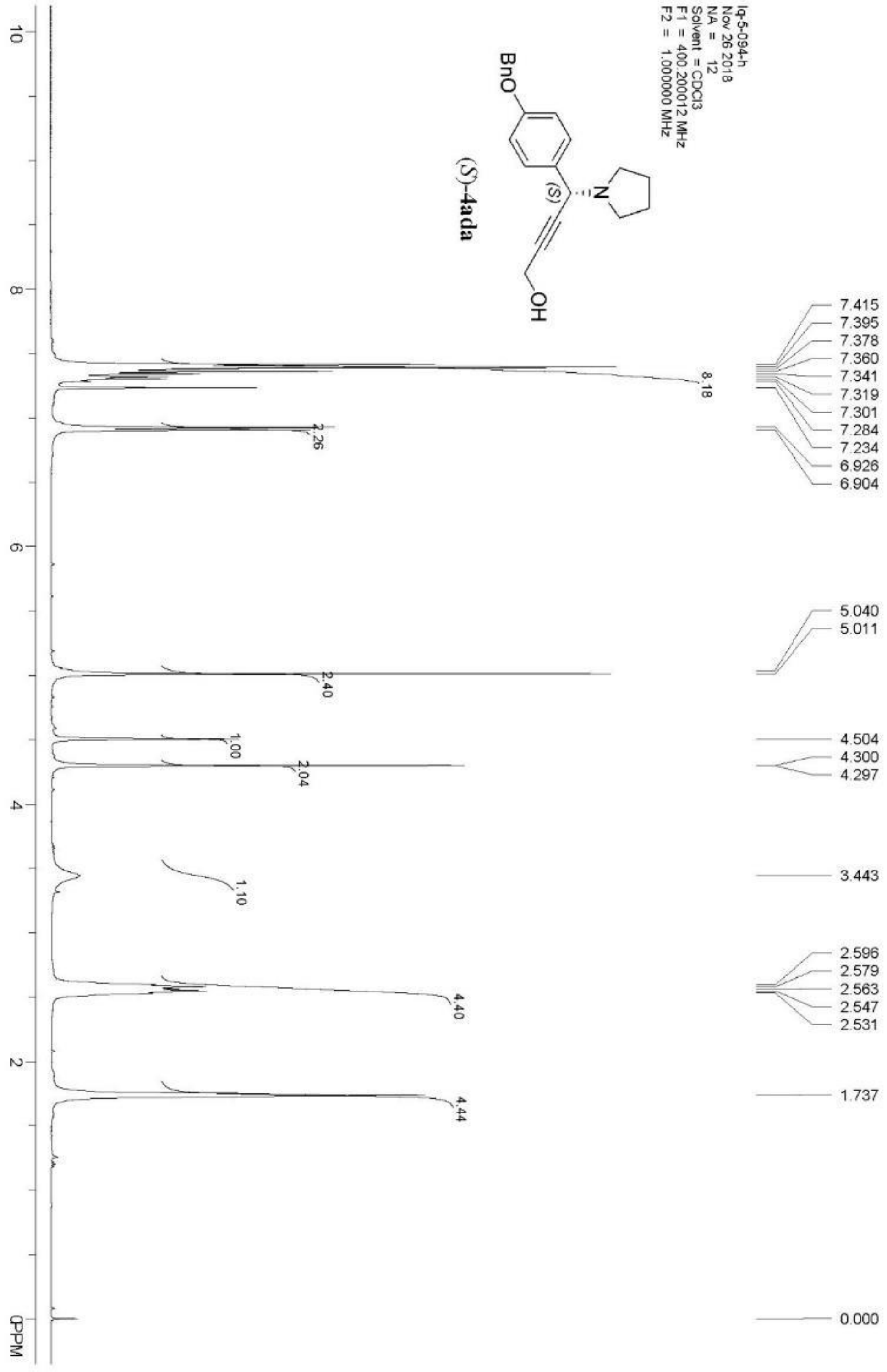

^{1}H NMR (400 MHz, CDCl3) spectrum for (*S*)-4ada

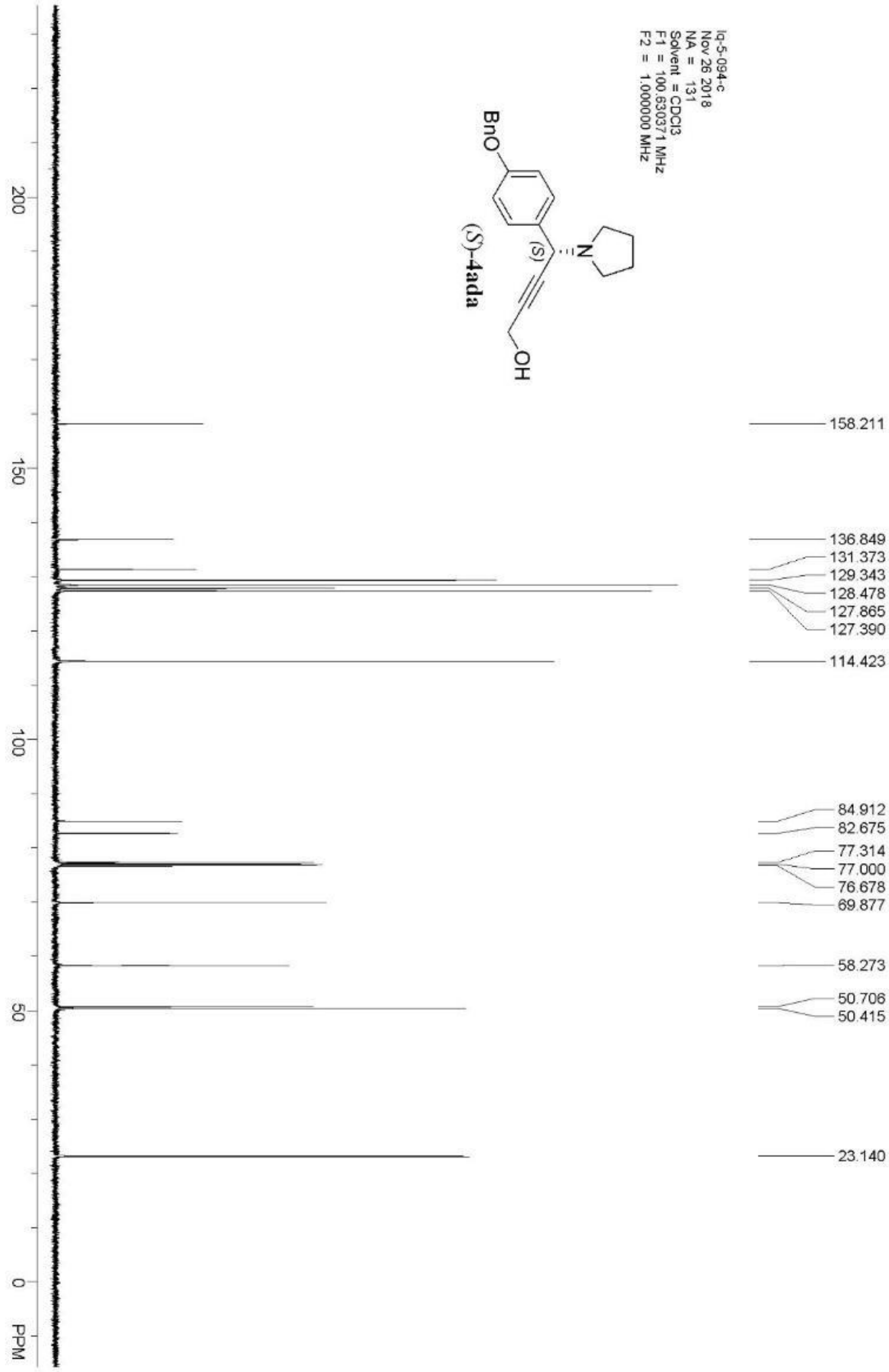

^{13}C NMR (400 MHz, CDCl$_3$) spectrum for (*S*)-4ada

Empower 3 SOFTWARE

Injection Summary Report

SAMPLE INFORMATION

Sample Name:	lq-5-094-ad-h-95-5-1.0-214	Acquired By:	System
Sample Type:	Unknown	Sample Set Name:	
Vial:	1	Acq. Method Set:	HPLC
Injection #:	2	Processing Method:	20181124
Injection Volume:	4.00 ul	Channel Name:	W2489 ChA
Run Time:	60.0 Minutes	Proc. Chnl. Descr.:	W2489 ChA 214nm
Date Acquired:	11/25/2018 1:55:33 PM CST		
Date Processed:	11/25/2018 2:44:37 PM CST		

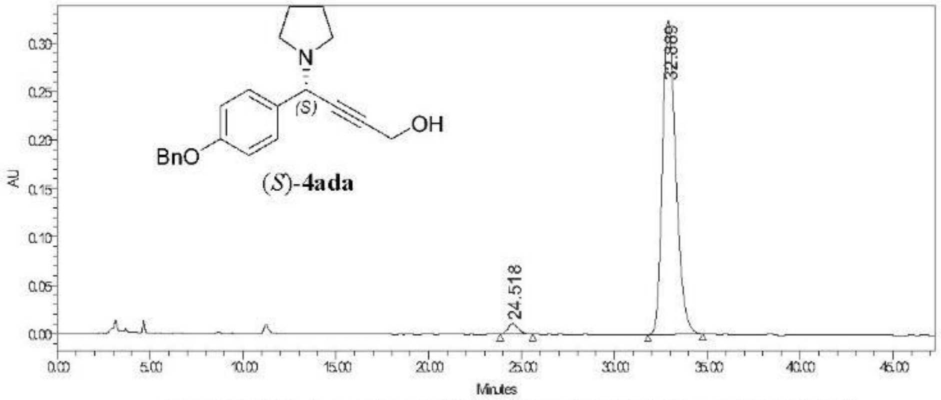

Channel: W2489 ChA; Processed Channel: W2489 ChA 214nm; Result Id: 1080; Processing Method: 2018112·

Processed Channel Descr.: W2489 ChA 214nm

	Processed Channel Descr.	RT	Area	%Area	Height
1	W2489 ChA 214nm	24.518	421873	2.53	10989
2	W2489 ChA 214nm	32.889	16236927	97.47	323631

Reported by User: System
Report Method: Injection Summary Report
Report Method ID: 1007 1007
Page: 1 of 1

Project Name: HPLC
Date Printed:
11/25/2018
2:45:09 PM PRC

HPLC spectrum for (*S*)-4ada

Empower 3 SOFTWARE

Injection Summary Report

SAMPLE INFORMATION

Sample Name:	lq-5-092-ad-h-95-5-1.0-214	Acquired By:	System
Sample Type:	Unknown	Sample Set Name:	
Vial:	1	Acq. Method Set:	HPLC
Injection #:	1	Processing Method:	20181124
Injection Volume:	2.50 ul	Channel Name:	W2489 ChA
Run Time:	60.0 Minutes	Proc. Chnl. Descr.:	W2489 ChA 214nm
Date Acquired:	11/25/2018 1:08:15 PM CST		
Date Processed:	11/25/2018 1:57:32 PM CST		

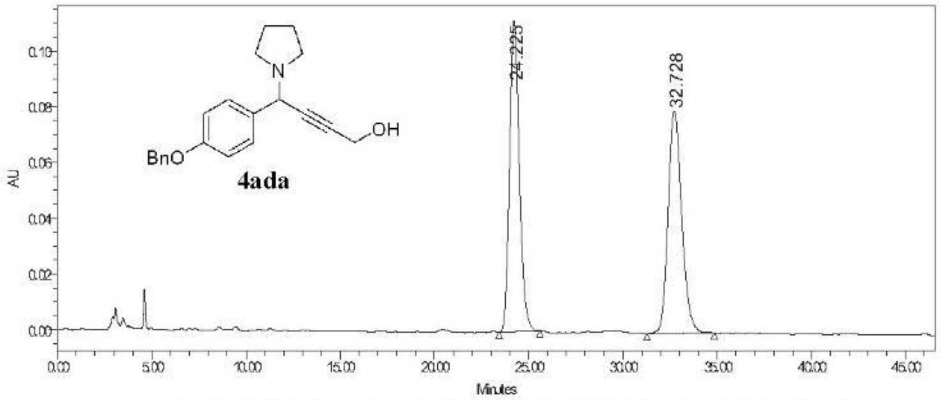

Channel: W2489 ChA; Processed Channel: W2489 ChA 214nm; Result Id: 1079; Processing Method: 2018112[illegible]

Processed Channel Descr.: W2489 ChA 214nm

	Processed Channel Descr.	RT	Area	%Area	Height
1	W2489 ChA 214nm	24.225	4011177	49.95	11148[illegible]
2	W2489 ChA 214nm	32.728	4019419	50.05	79735

Reported by User: System
Report Method: Injection Summary Report
Report Method ID: 1007 1007
Page: 1 of 1

Project Name: HPLC
Date Printed:
11/25/2018
1:57:54 PM PRC

HPLC spectrum for (±)-4ada

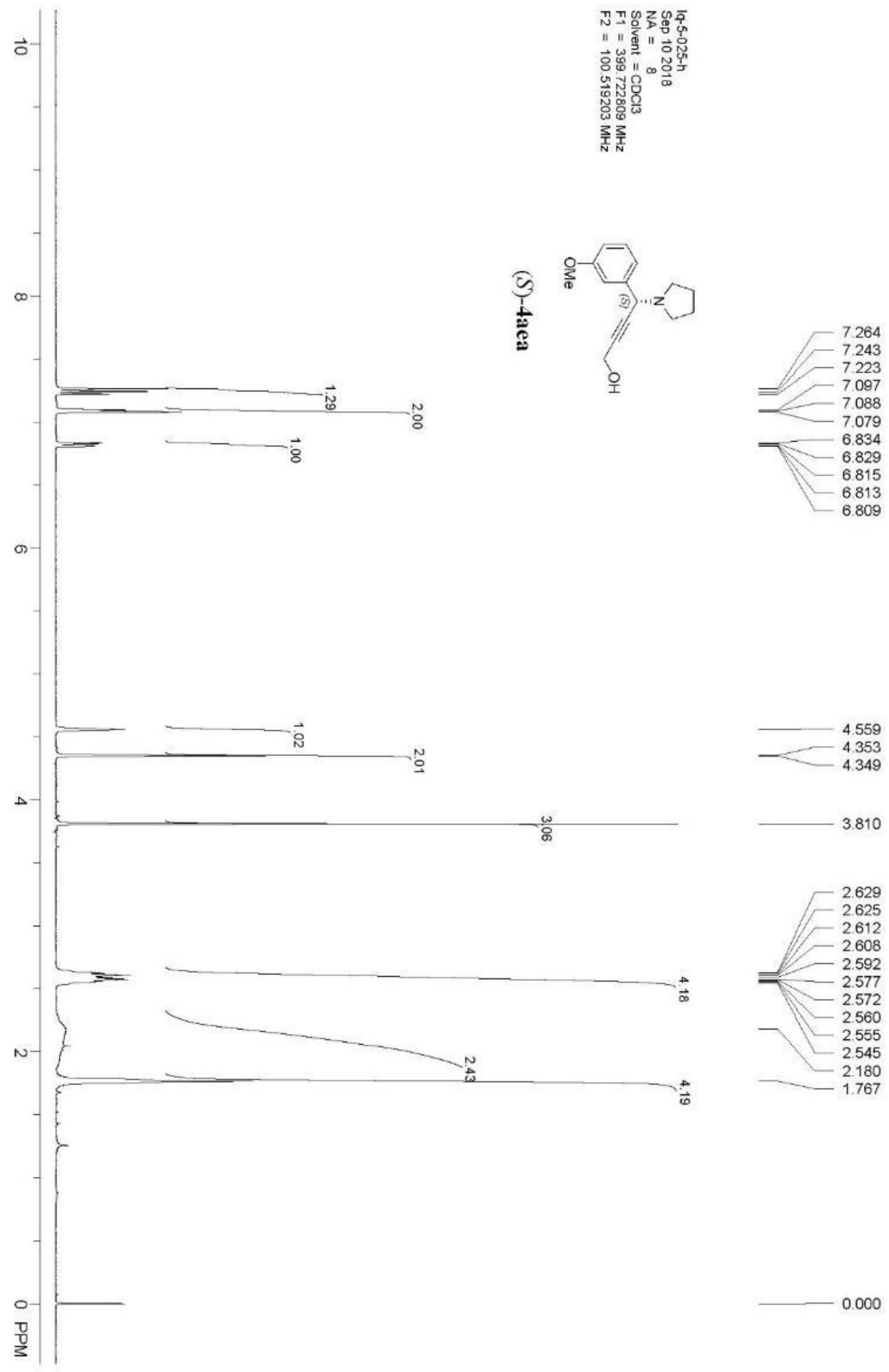

^{1}H NMR (400 MHz, CDCl3) spectrum for (*S*)-4aea

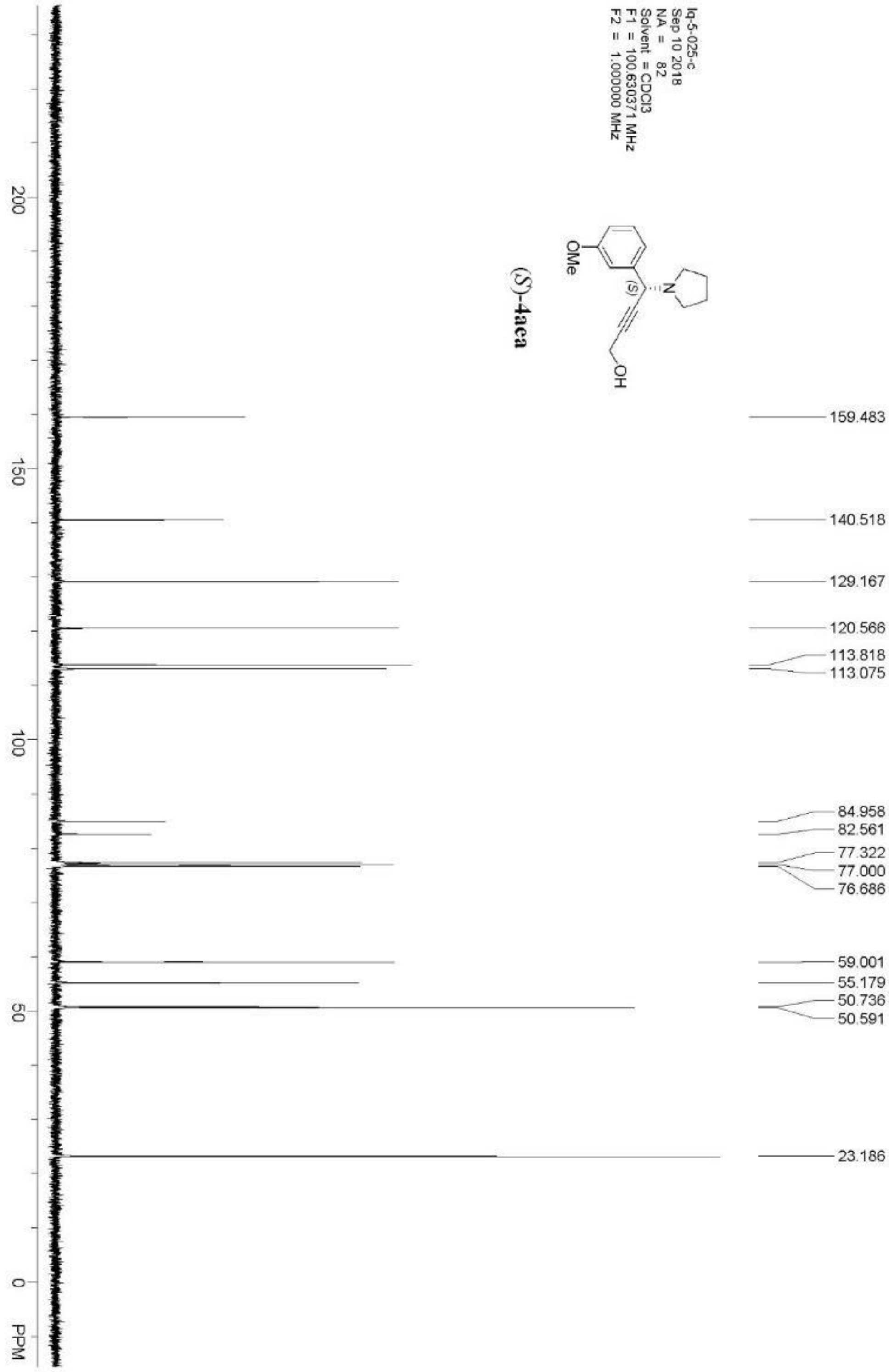

^{13}C NMR (400 MHz, CDCl$_3$) spectrum for (*S*)-4aea

1q-5-025

实验时间：2018-09-10, 11:35:39　　　　报告时间：2018-09-10, 11:56:00
谱图文件:D:\data\slf\lq\2018-09-10\lq-5-25-AD-H-90+10-0.8-214.org

实验内容简介：
AD-H　90:10
214nm　0.8ml/min

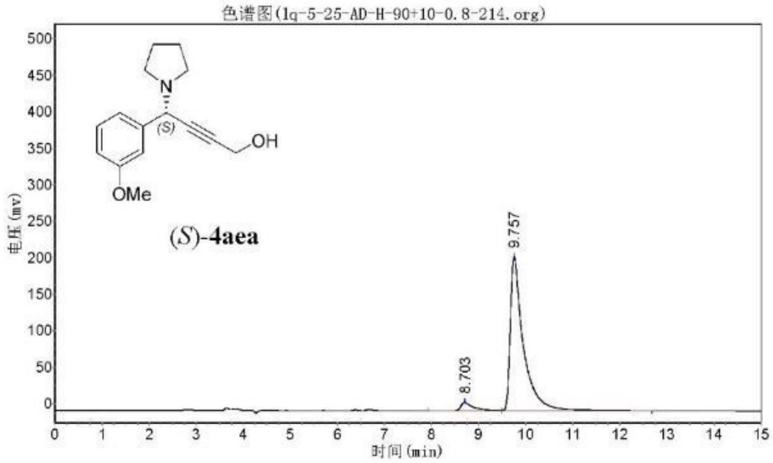

分析结果表

峰号	峰名	保留时间	峰高	峰面积	含量
1		8.703	11418.294	212894.609	4.9548
2		9.757	210695.859	4083814.000	95.0452
**总计**			222114.153	4296708.609	100.0000

HPLC spectrum for (*S*)-4aea

xhb-1-045

实验时间：2018-09-10, 11:18:41　　　　报告时间：2018-09-10, 11:37:36
谱图文件:D:\data\slf\lq\2018-09-10\xhb-1-045-AD-H-90+10-0.8-214.org

实验内容简介：
AD-H　90:10
214nm　0.8ml/min

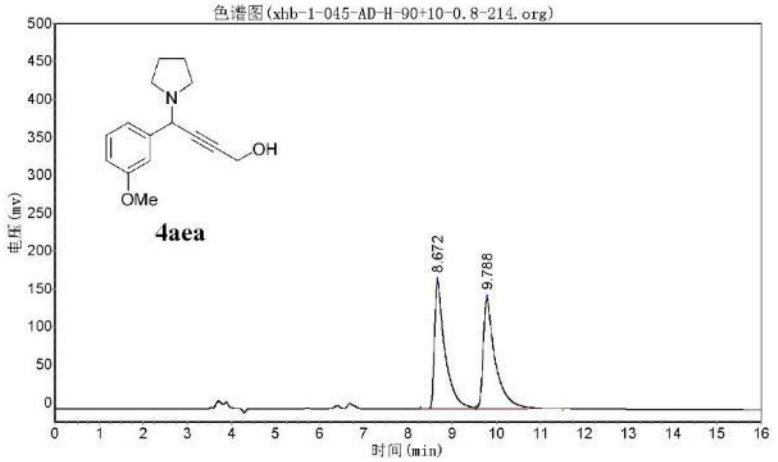

分析结果表

峰号	峰名	保留时间	峰高	峰面积	含量
1		8.672	168671.188	2799241.750	49.8850
2		9.788	145638.016	2812142.500	50.1150
总计			314309.203	5611384.250	100.0000

HPLC spectrum for (±)-4aea

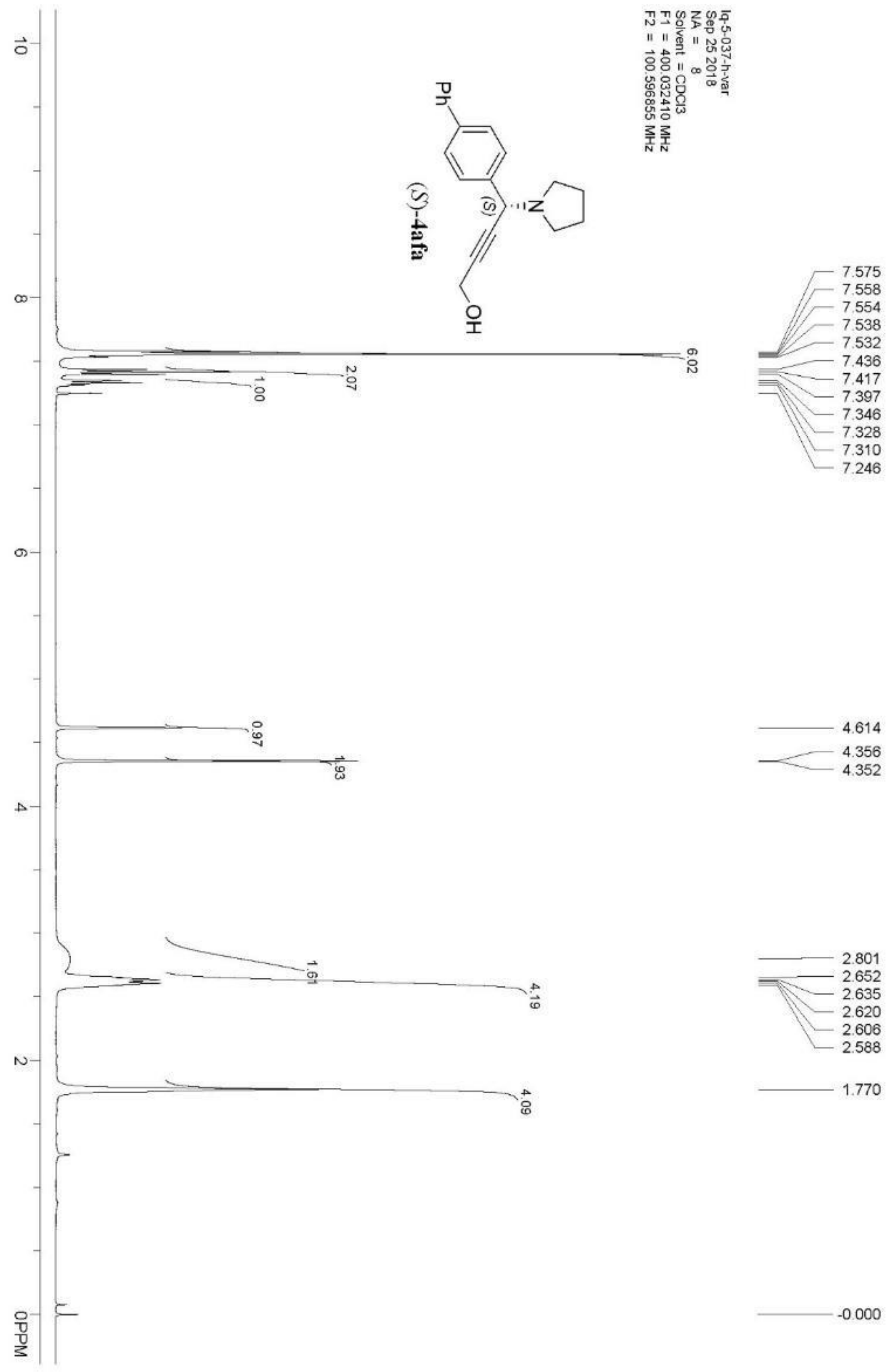

^{1}H NMR (400 MHz, CDCl3) spectrum for (*S*)-4afa

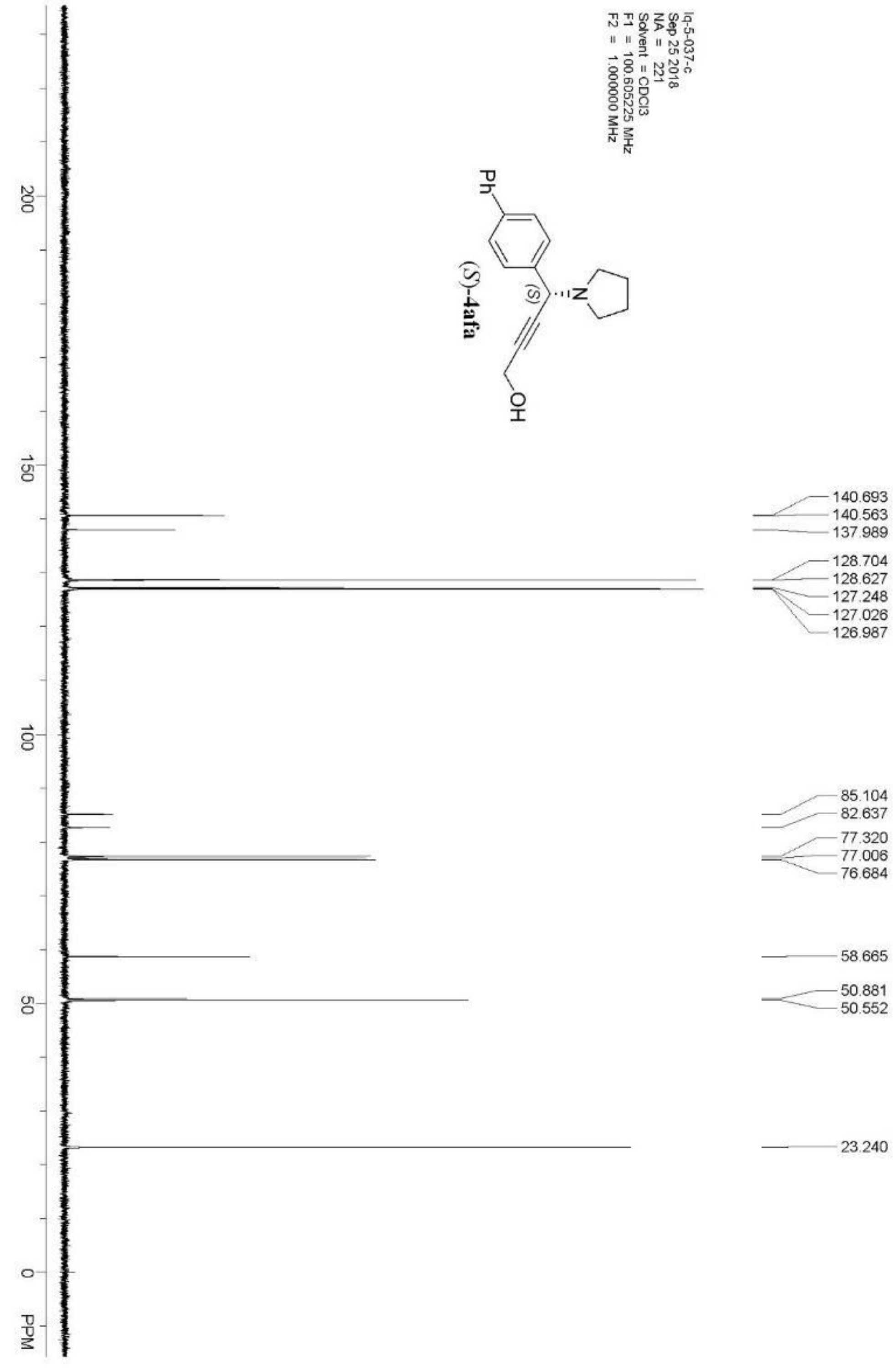

^{13}C NMR (400 MHz, $CDCl_3$) spectrum for (*S*)-4afa

1q-5-037

实验时间：2018-09-21,20:44:12　　　　报告时间：2018-09-25,16:09:37
谱图文件:D:\data\slf\lq\2018-09-21\lq-5-037-OD-H-95-5-1.0-214-2.org

实验内容简介：
OD-H　95:5
214nm　1.0ml/min

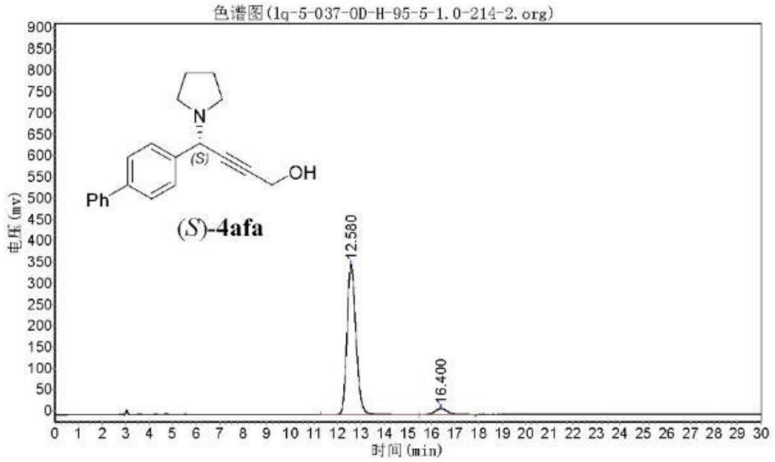

分析结果表

峰号	峰名	保留时间	峰高	峰面积	含量
1		12.580	352128.219	9326926.000	95.1920
2		16.400	13536.856	471086.000	4.8080
总计			365665.075	9798012.000	100.0000

HPLC spectrum for (*S*)-4afa

xhb-1-057

实验时间：2018-09-21, 19:33:33　　　　　　　　　　报告时间：2018-09-25, 16:07:43
谱图文件:D:\data\slf\lq\2018-09-21\xhb-1-057-OD-H-95-5-1.0-214.org

实验内容简介：
OD-H　95:5
214nm　1.0ml/min

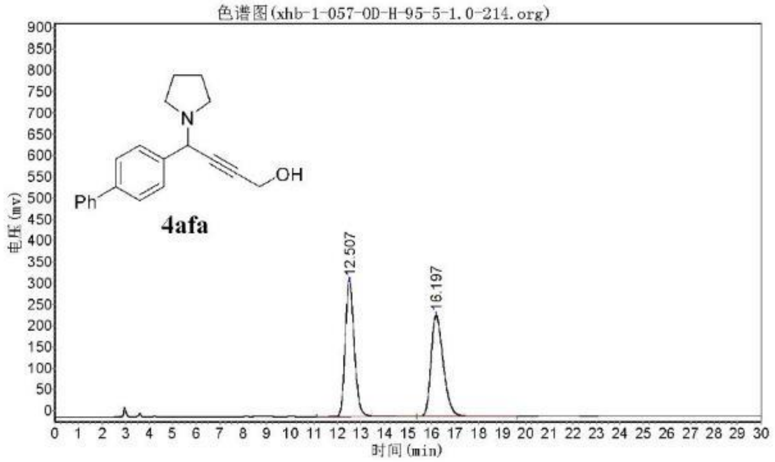

分析结果表

峰号	峰名	保留时间	峰高	峰面积	含量
1		12.507	319421.063	8660248.000	49.8565
2		16.197	237269.594	8710097.000	50.1435
总计			556690.656	17370345.000	100.0000

HPLC spectrum for (±)-4afa

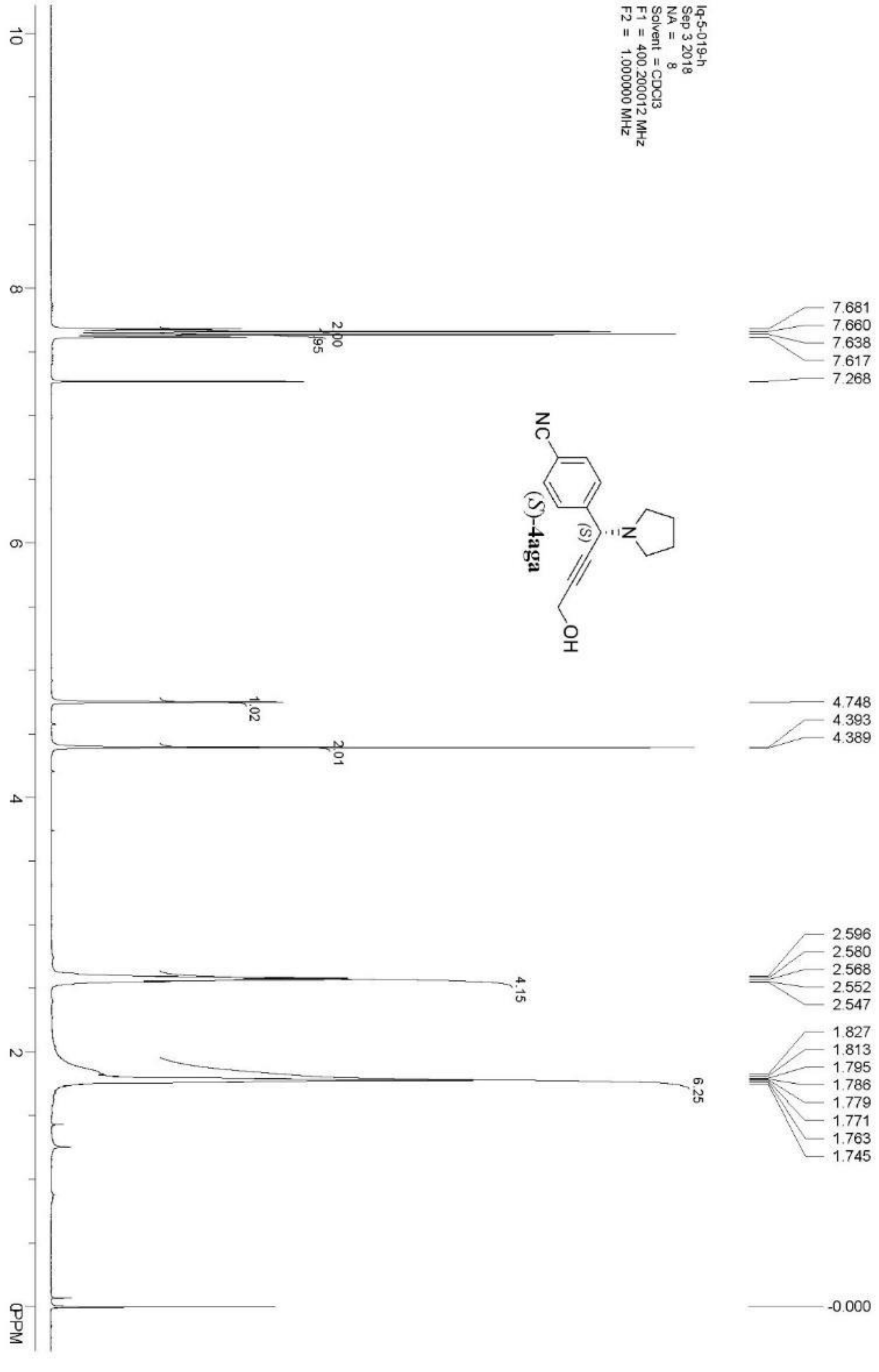

^{1}H NMR (400 MHz, CDCl3) spectrum for (*S*)-4aga

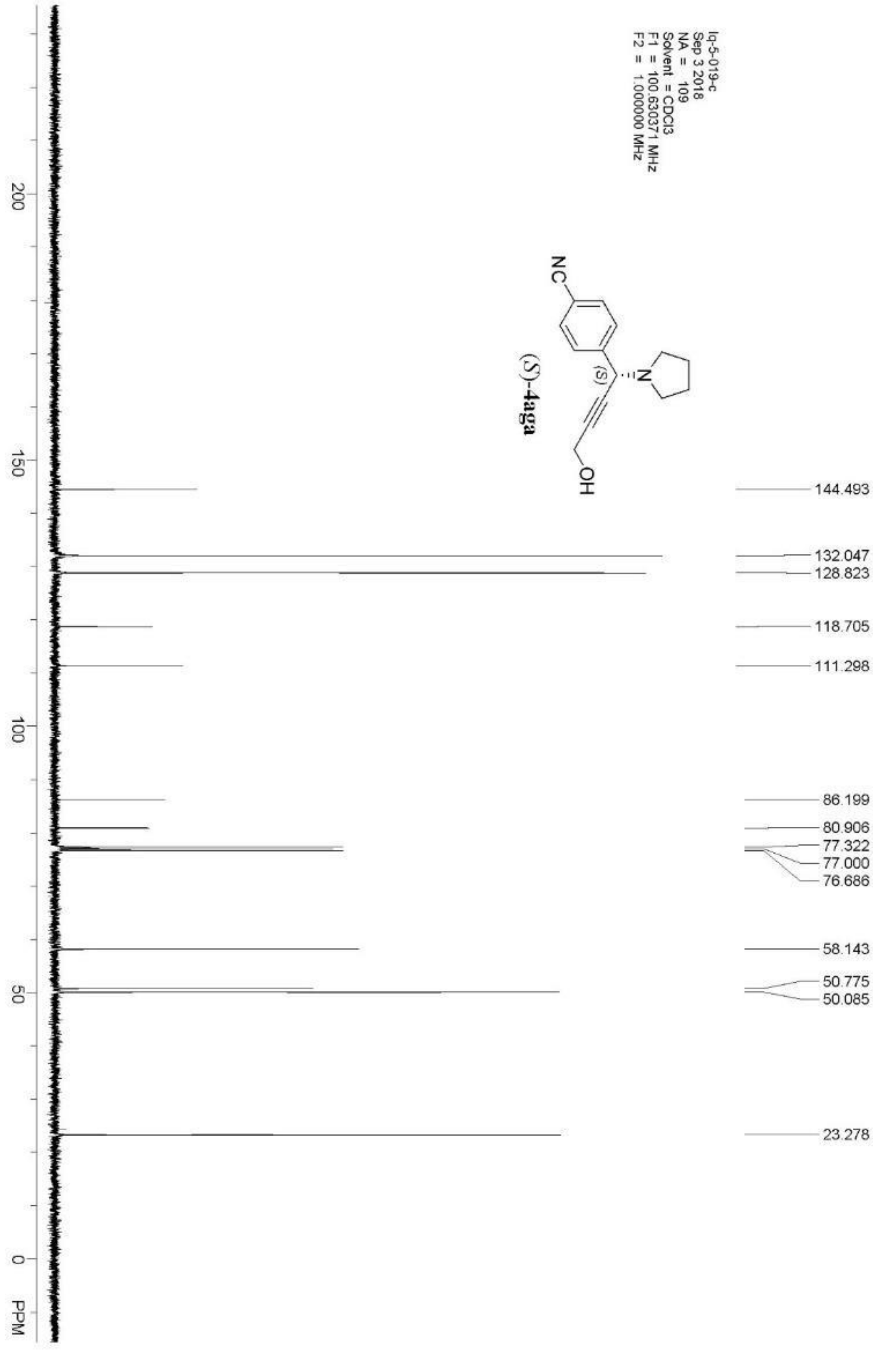

^{13}C NMR (400 MHz, CDCl3) spectrum for (*S*)-4aga

1q-5-019

实验时间：2018/9/3, 9:26:45　　　　报告时间：2018/9/3, 9:44:02
谱图文件:D:\data\slf\lq\2018-9-3\lq-5-019-ad-h-80+20-1-214.org

实验内容简介：
AD-H　80:20
214nm　1.0ml/min

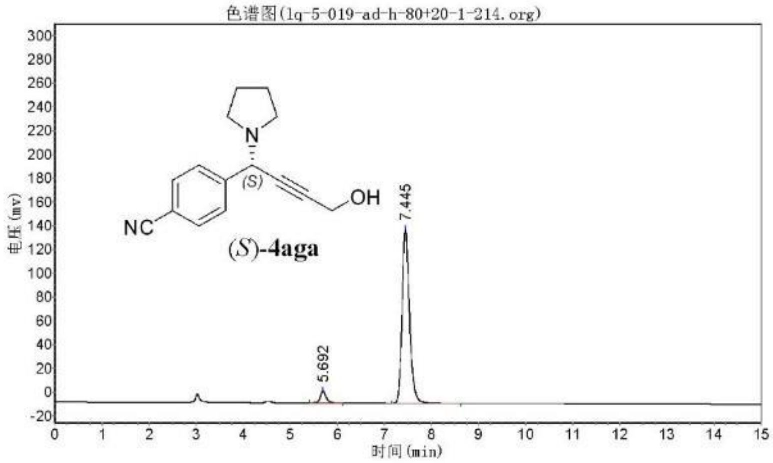

分析结果表

峰号	峰名	保留时间	峰高	峰面积	含量
1		5.692	9685.859	81720.727	4.8793
2		7.445	146404.297	1593131.000	95.1207
**总计**			156090.156	1674851.727	100.0000

HPLC spectrum for (*S*)-4aga

1q-5-009

实验时间：2018/9/3,9:42:44　　　　　　　　　　　报告时间：2018/9/3,9:59:16
谱图文件:D:\data\slf\lq\2018-9-3\lq-5-009-ad-h-80+20-1-214.org

实验内容简介：
AD-H　80:20
214nm　1.0ml/min

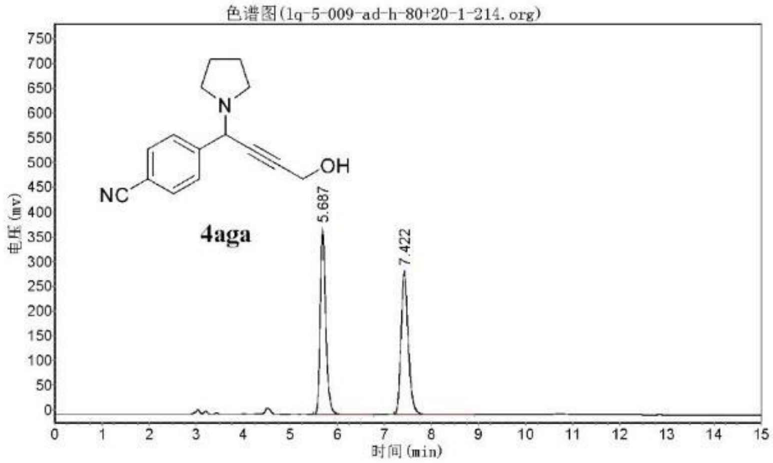

分析结果表

峰号	峰名	保留时间	峰高	峰面积	含量
1		5.687	370123.594	3061461.750	49.9539
2		7.422	282973.813	3067116.500	50.0461
**总计**			653097.406	6128578.250	100.0000

HPLC spectrum for (±)-4aga

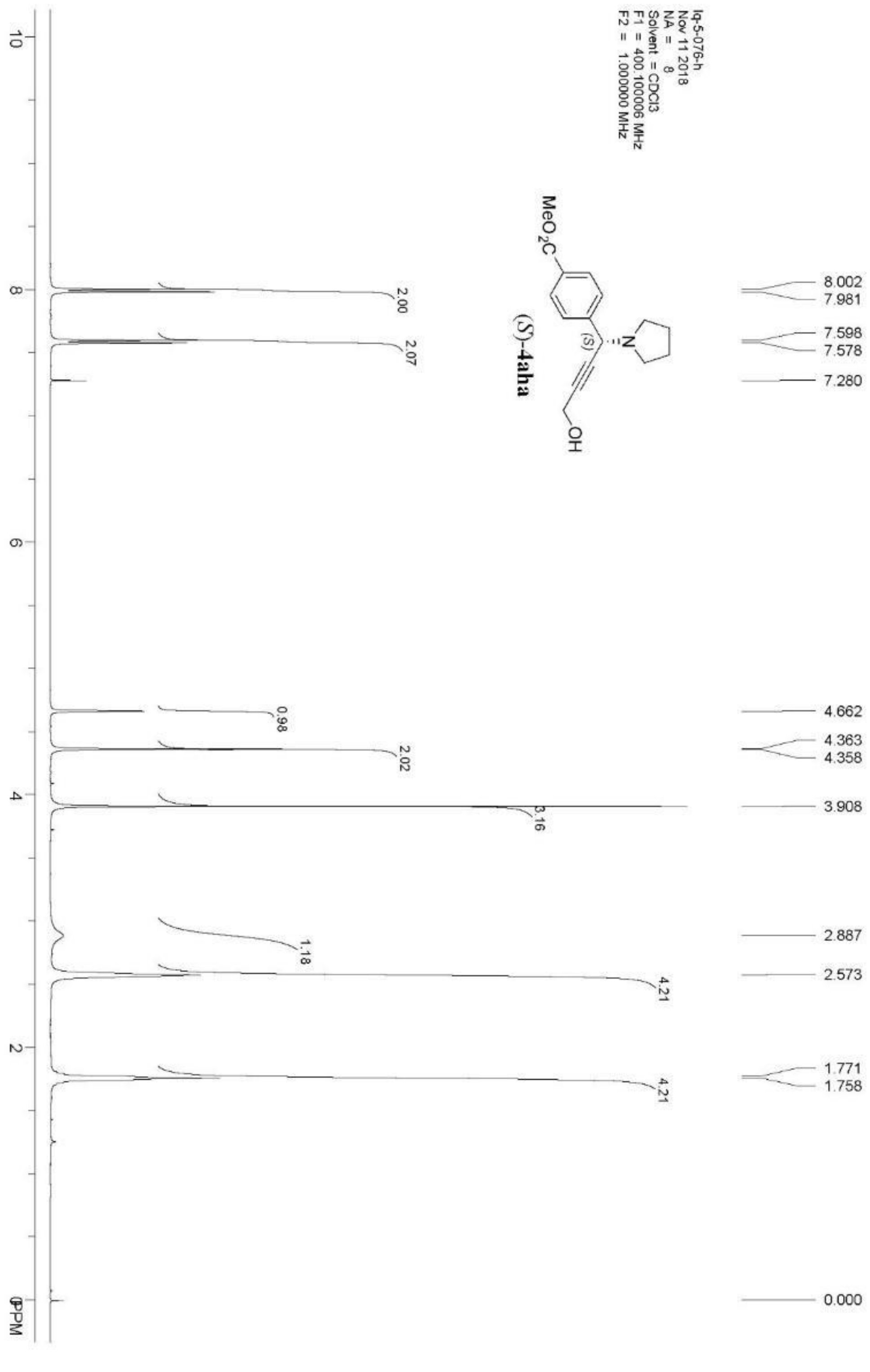

^{1}H NMR (400 MHz, CDCl3) spectrum for (*S*)-4aha

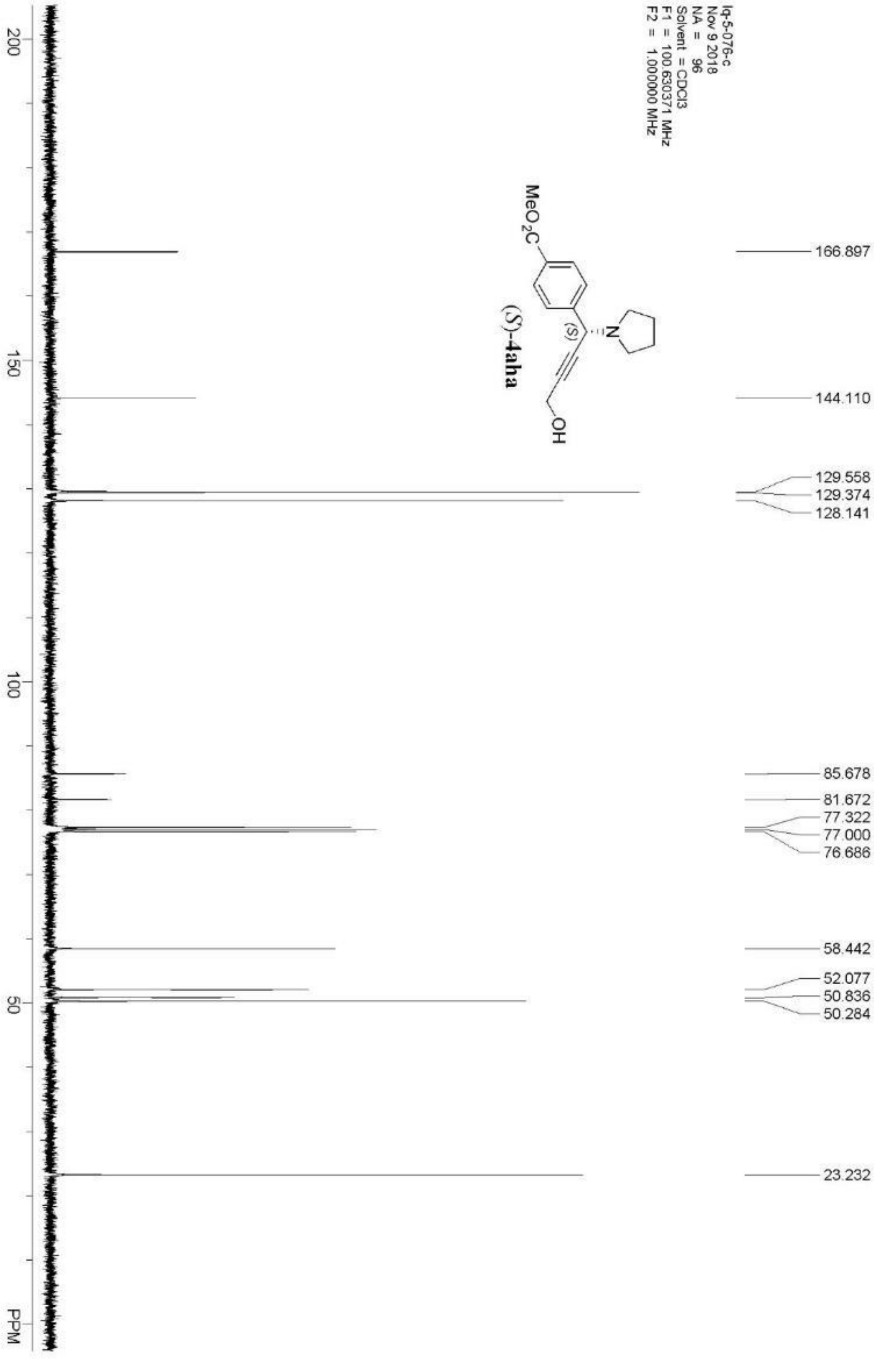

^{13}C NMR (400 MHz, CDCl3) spectrum for (*S*)**-4aha**

lq-5-076

实验时间：2018-11-09, 0:02:49 报告时间：2018-11-09, 0:02:52
谱图文件:D:\data\slf\lq\2018-11-08-4\LQ-5-076-OD-H-95-5-1-214-2.mdy

实验内容简介：
OD-H 95:5
1ml/min 214nm

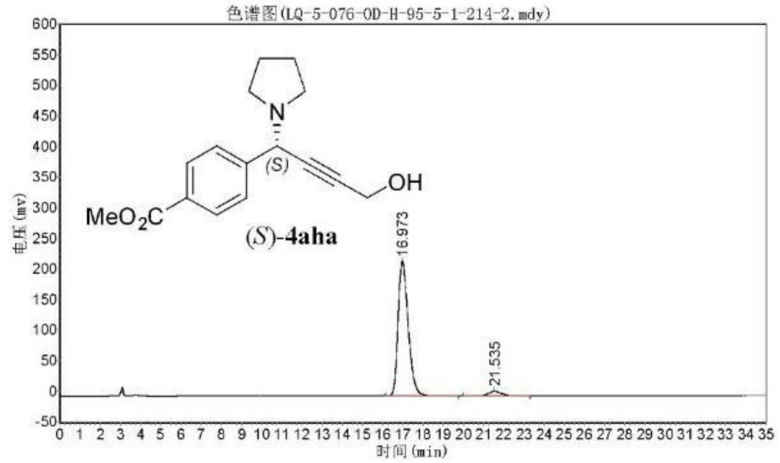

分析结果表

峰号	峰名	保留时间	峰高	峰面积	含量
1		16.973	220238.844	7673197.500	95.8023
2		21.535	7384.401	336213.281	4.1977
**总计**			227623.245	8009410.781	100.0000

HPLC spectrum for (*S*)-4aha

LQ-5-072

实验时间：2018-11-08,21:42:52　　　　报告时间：2018-11-08,22:29:35
谱图文件:D:\data\slf\lq\2018-11-08-4\LQ-5-072-OD-H-95-5-1-214-2.org

实验内容简介：
OD-H　95:5
1ml/min　214nm

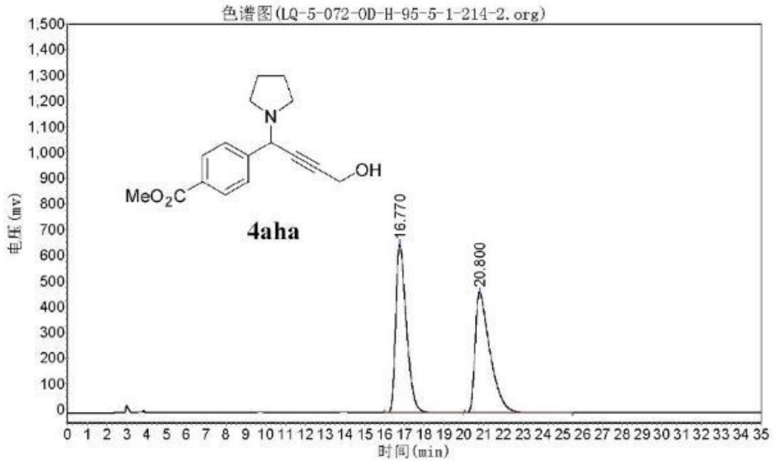

分析结果表

峰号	峰名	保留时间	峰高	峰面积	含量
1		16.770	655659.000	24390814.000	50.0134
2		20.800	470387.031	24377784.000	49.9866
**总计**			1126046.031	48768598.000	100.0000

HPLC spectrum for (±)-4aha

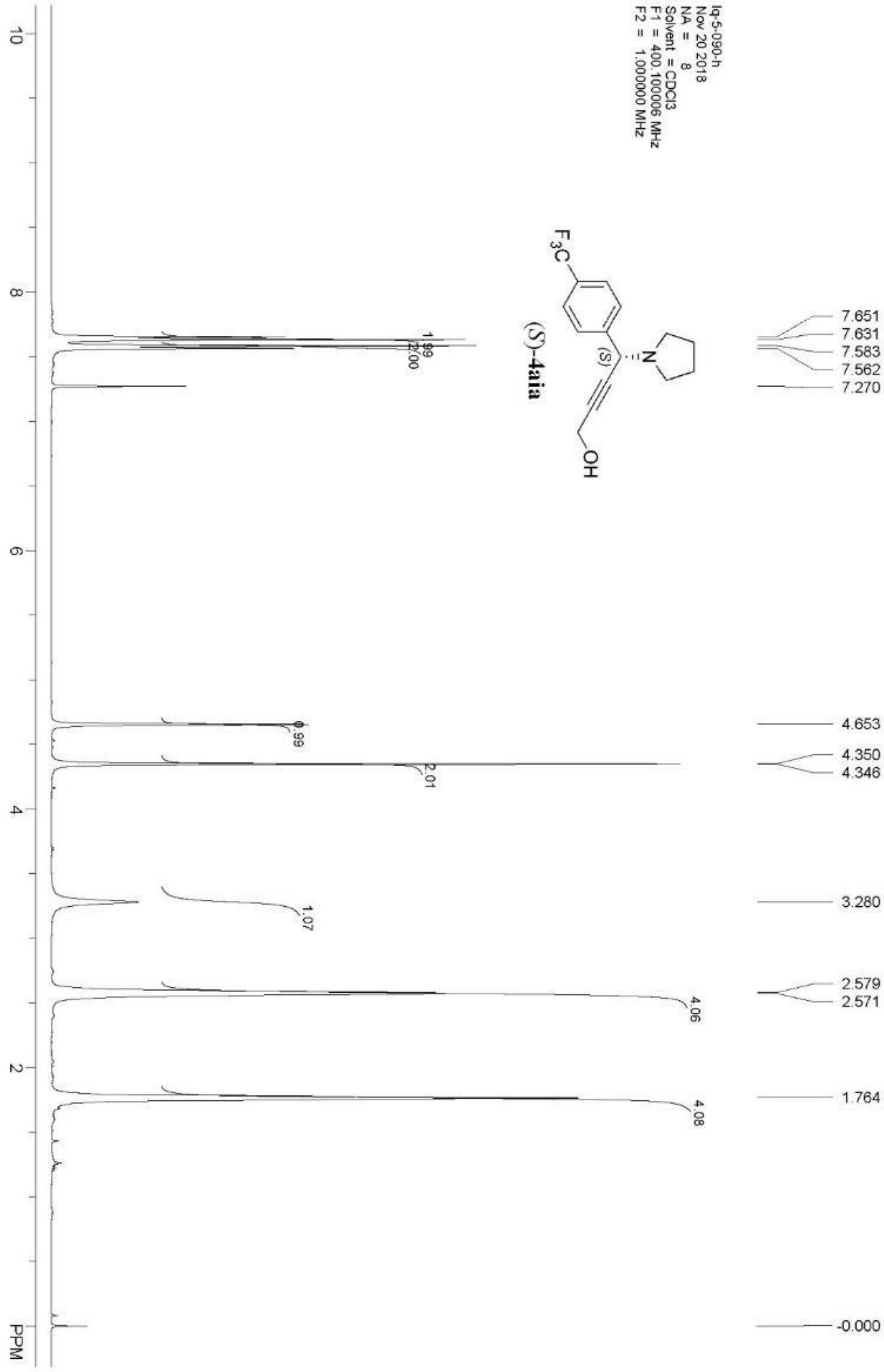

^{1}H NMR (400 MHz, CDCl3) spectrum for (*S*)-4aia

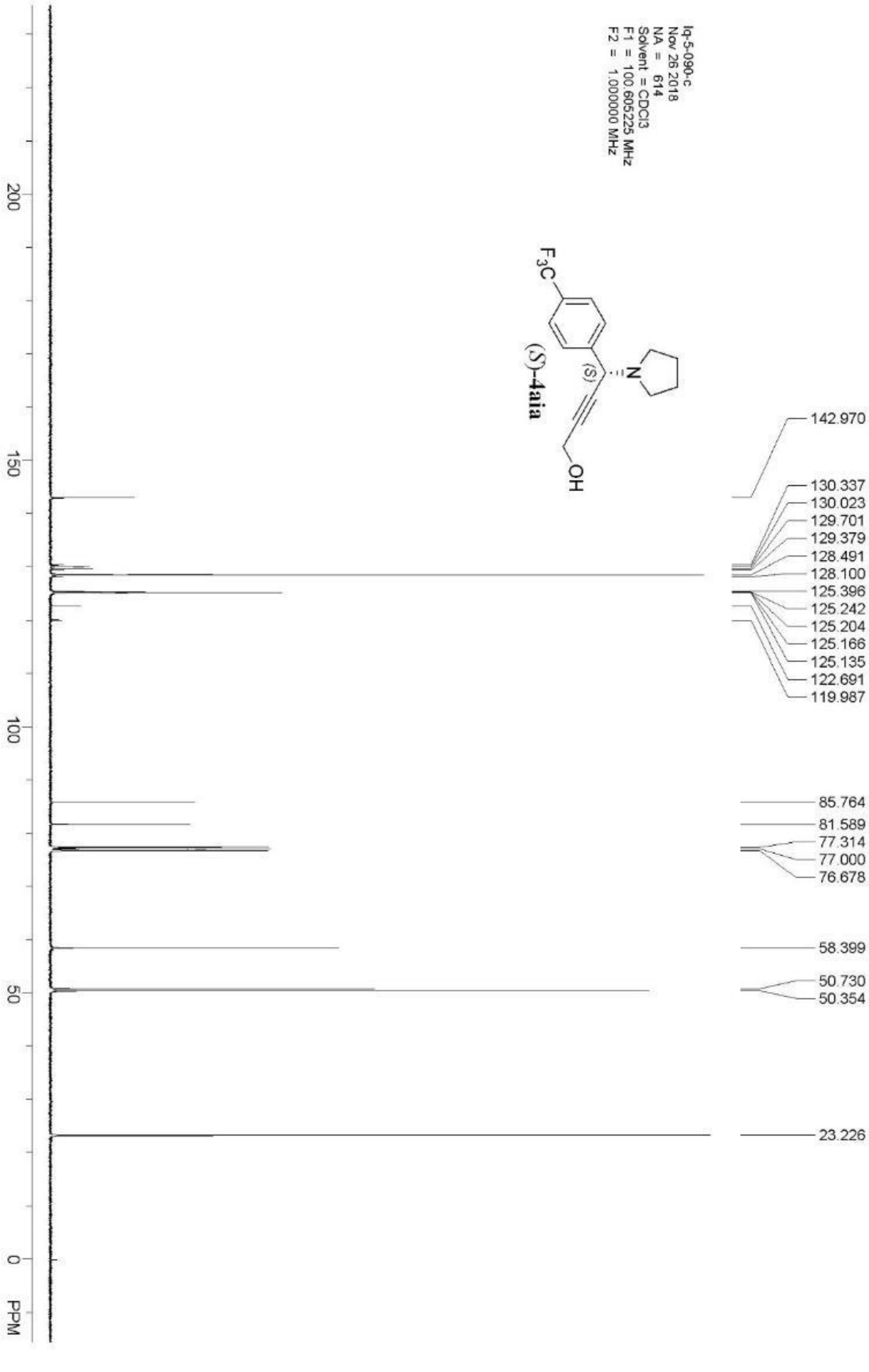

^{13}C NMR (400 MHz, CDCl$_3$) spectrum for (*S*)-4aia

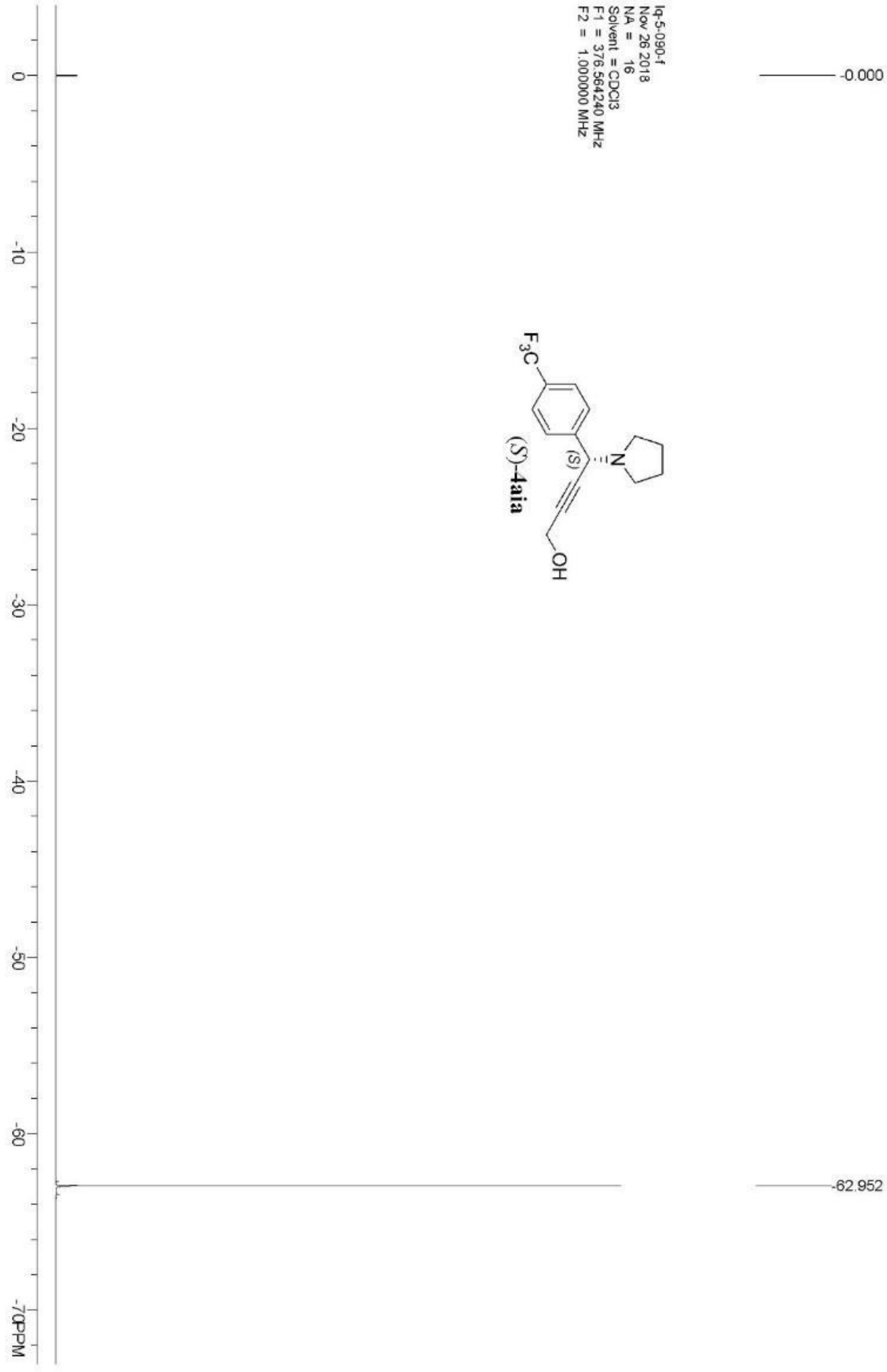

^{19}F NMR (376 MHz, $CDCl_3$) spectrum for (*S*)-4aia ($CFCl_3$ was used as the internal standard)

1q-5-090

实验时间：2018-11-20, 16:44:16 报告时间：2018-11-20, 17:05:04
谱图文件:D:\data\slf\lq\2018-11-20-3\lq-5-090-ad-h-95+5-1-214.org

实验内容简介：
AD-H 95:5
214nm 1.0ml/min

色谱图(lq-5-090-ad-h-95+5-1-214.org)

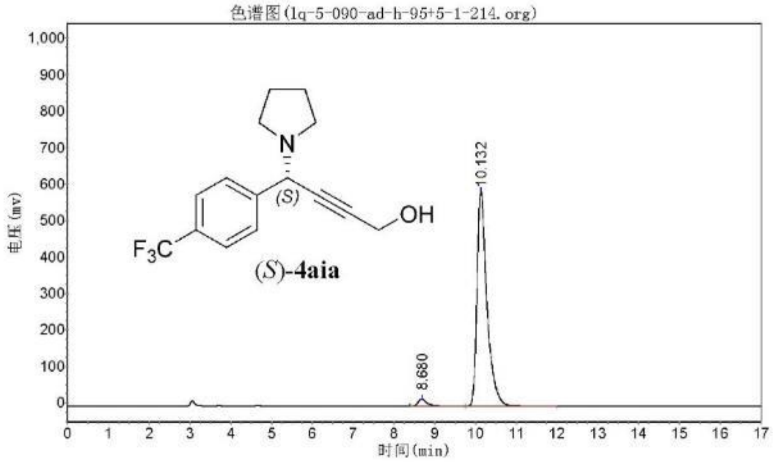

分析结果表

峰号	峰名	保留时间	峰高	峰面积	含量
1		8.680	19229.711	287770.656	2.8057
2		10.132	587921.250	9968700.000	97.1943
**总计**			607150.961	10256470.656	100.0000

HPLC spectrum for (±)-4aia

xhb-1-105

实验时间：2018-11-20, 16:26:07　　　　　　　　　　　　　报告时间：2018-11-20, 16:45:54
谱图文件:D:\data\slf\lq\2018-11-20-3\xhb-1-105-ad-h-95+5-1-214.org

实验内容简介：
AD-H　95:5
214nm　1.0ml/min

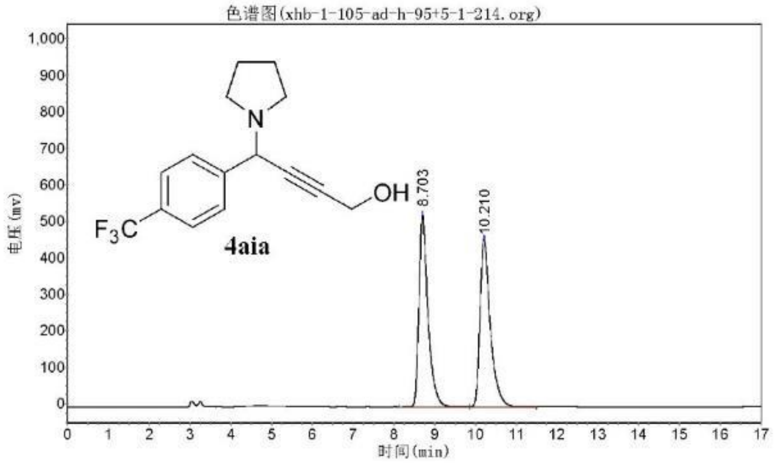

分析结果表

峰号	峰名	保留时间	峰高	峰面积	含量
1		8.703	524751.250	8111887.500	49.9908
2		10.210	459010.656	8114864.500	50.0092
**总计**			983761.906	16226752.000	100.0000

HPLC spectrum for (*S*)-4aia

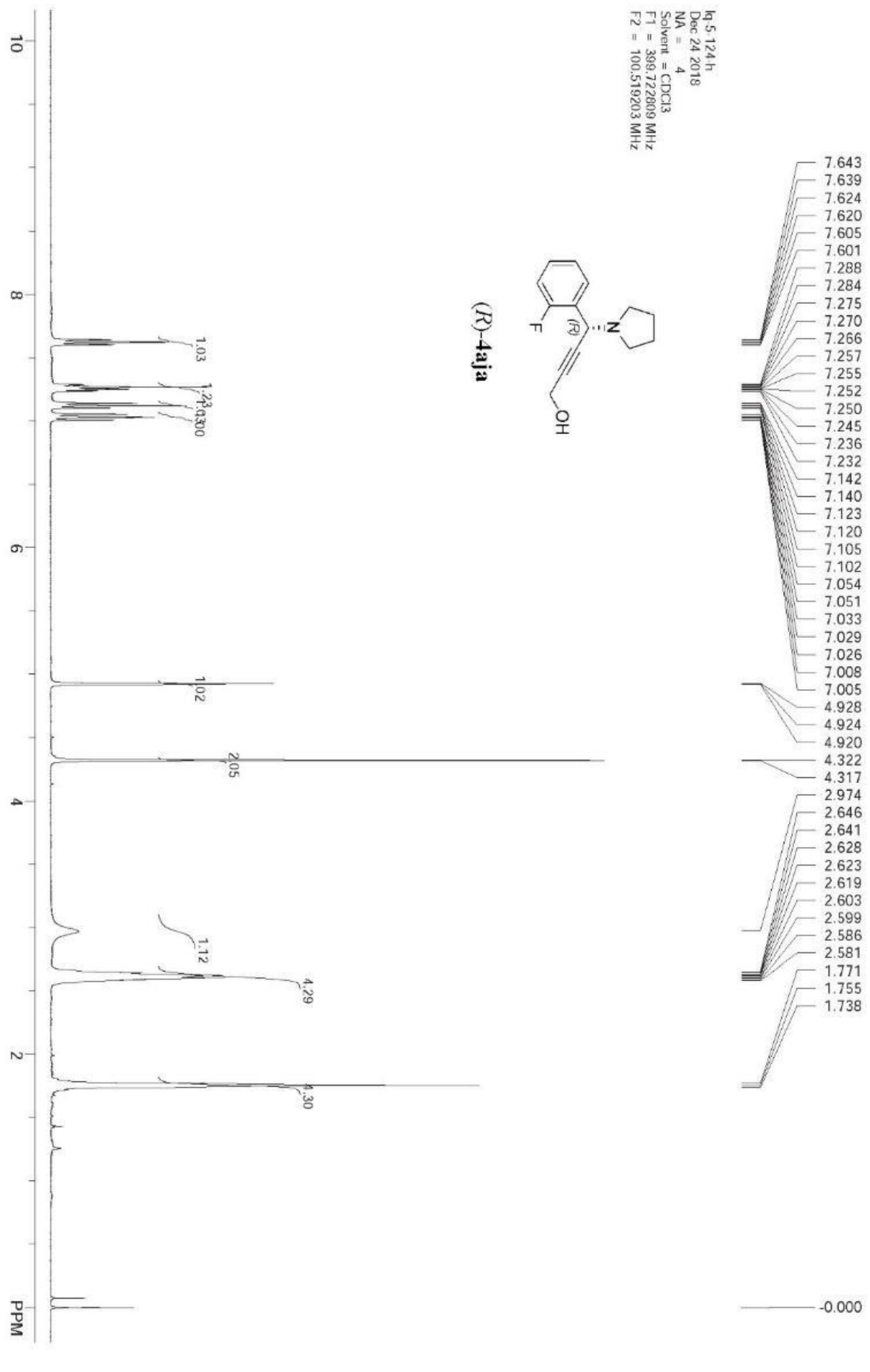

^{1}H NMR (400 MHz, CDCl3) spectrum for (*R*)**-4aja**

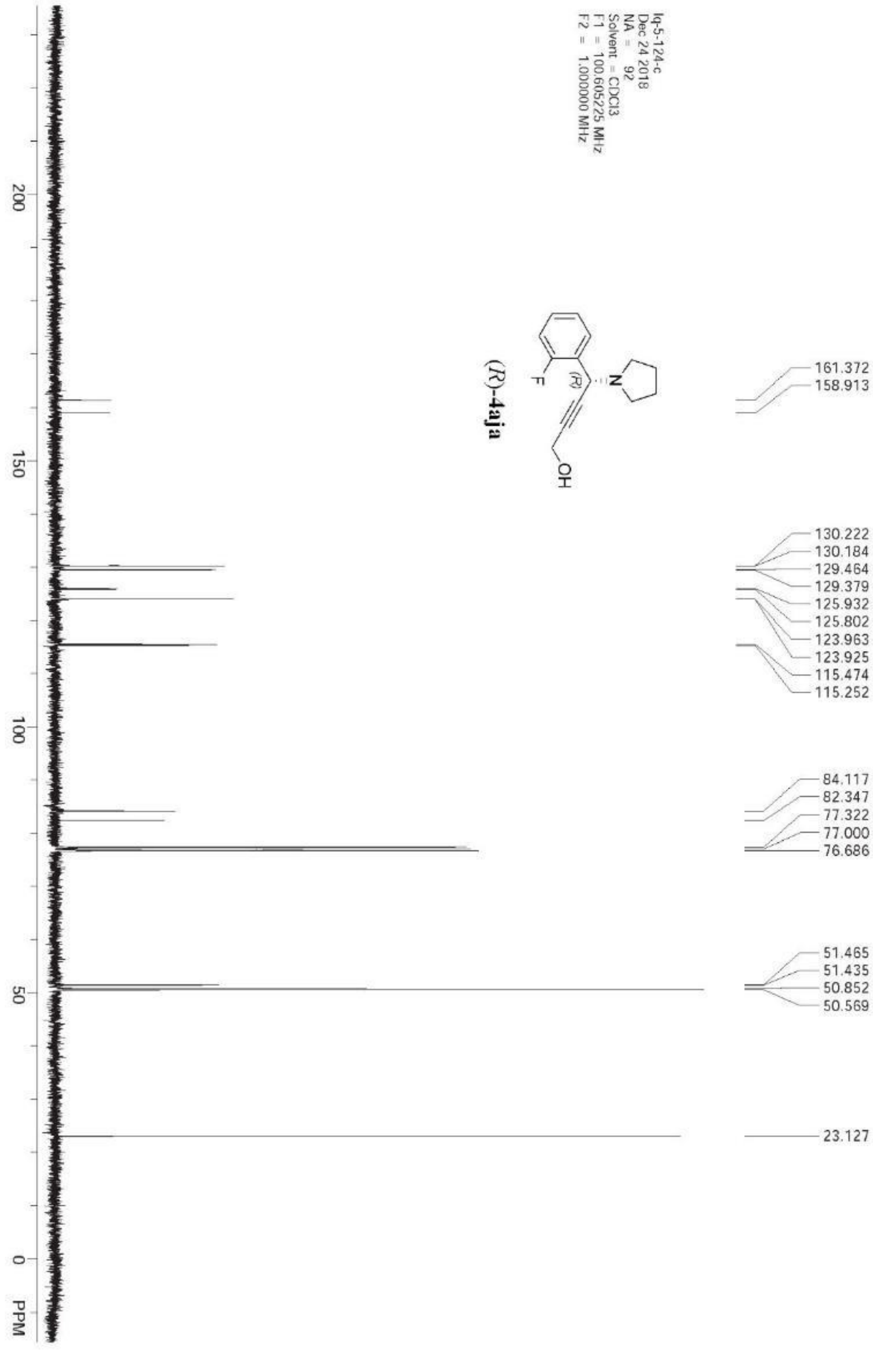

^{13}C NMR (400 MHz, CDCl$_3$) spectrum for (*R*)**-4aja**

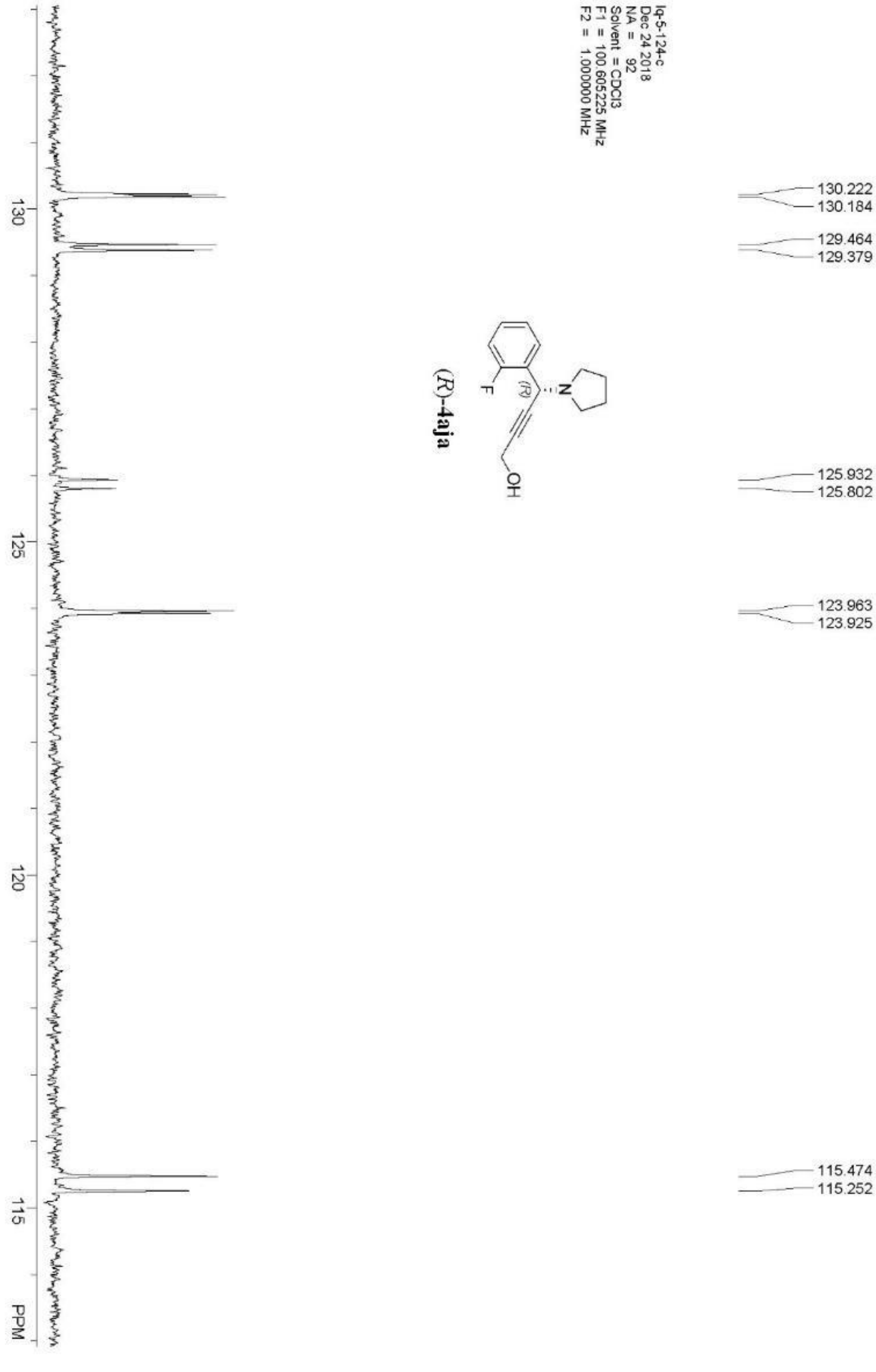

^{13}C NMR (400 MHz, $CDCl_3$) spectrum for (*R*)-4aja

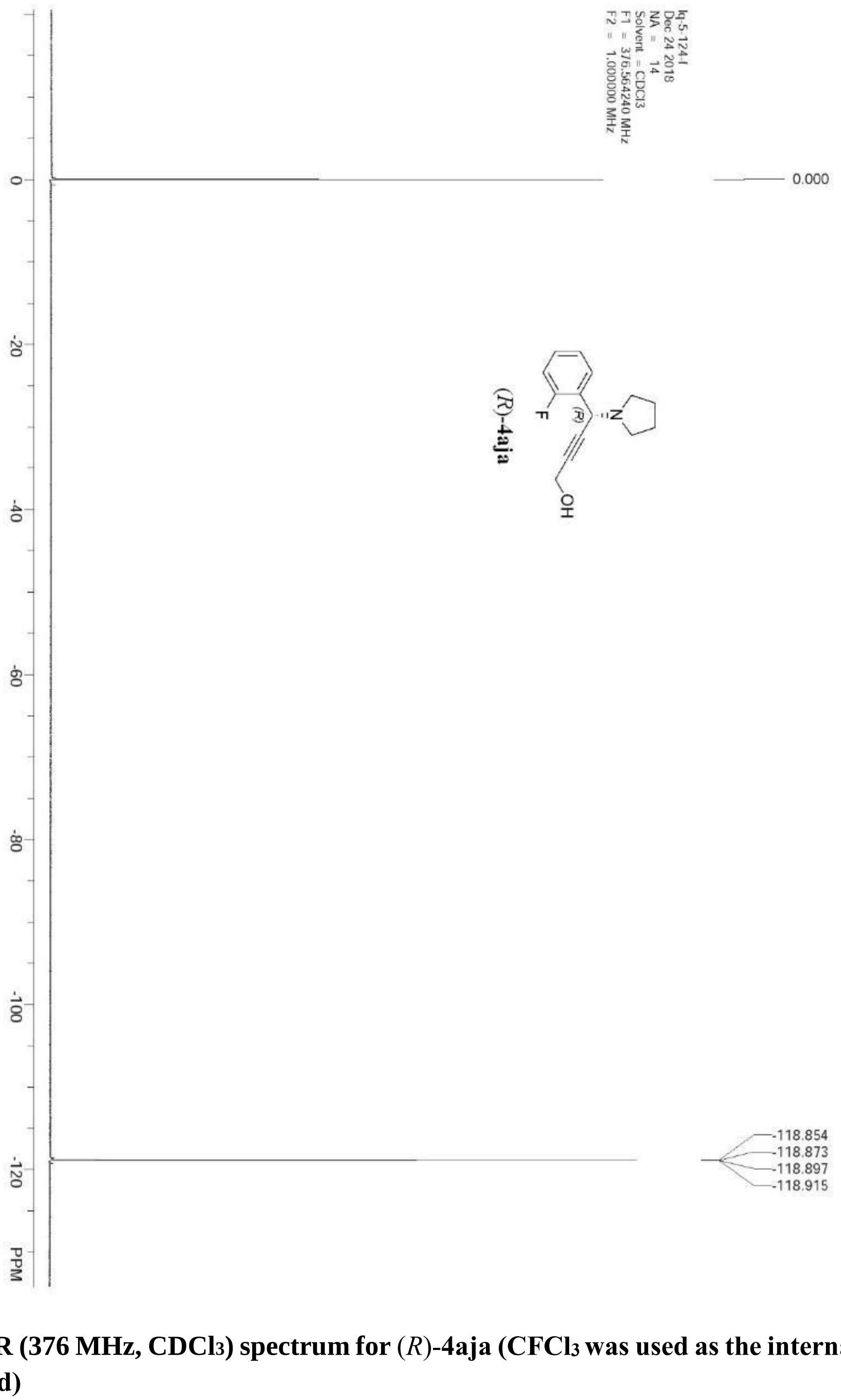

^{19}F NMR (376 MHz, $CDCl_3$) spectrum for (*R*)-4aja ($CFCl_3$ was used as the internal standard)

Empower 3 SOFTWARE

Injection Summary Report

SAMPLE INFORMATION

Sample Name:	lq-5-124-od-h-95-5-1.0-214	Acquired By:	System
Sample Type:	Unknown	Sample Set Name:	
Vial:	1	Acq. Method Set:	HPLC
Injection #:	3	Processing Method:	Default
Injection Volume:	2.50 ul	Channel Name:	W2489 ChA
Run Time:	30.0 Minutes	Proc. Chnl. Descr.:	W2489 ChA 214nm
Date Acquired:	12/25/2018 2:06:20 AM CST		
Date Processed:	12/25/2018 2:31:44 AM CST		

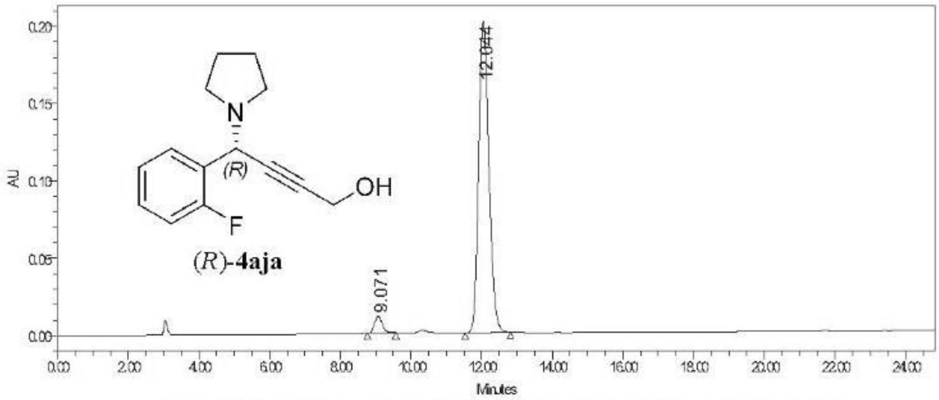

Processed Channel Descr.: W2489 ChA 214nm

	Processed Channel Descr.	RT	Area	%Area	Height
1	W2489 ChA 214nm	9.071	162858	3.88	10986
2	W2489 ChA 214nm	12.044	4039284	96.12	201493

Reported by User: System
Report Method: Injection Summary Report
Report Method ID: 1639 1639
Page: 1 of 1

Project Name: HPLC
Date Printed:
12/25/2018
2:32:53 AM PRC

HPLC spectrum for (*R*)-4aja

Empower 3 SOFTWARE

Injection Summary Report

SAMPLE INFORMATION

Sample Name:	xhb-1-134-od-h-95-5-1.0-214	Acquired By:	System
Sample Type:	Unknown	Sample Set Name:	
Vial:	1	Acq. Method Set:	HPLC
Injection #:	1	Processing Method:	20181124
Injection Volume:	5.00 ul	Channel Name:	W2489 ChA
Run Time:	60.0 Minutes	Proc. Chnl. Descr.:	W2489 ChA 214nm
Date Acquired:	12/25/2018 1:08:16 AM CST		
Date Processed:	12/25/2018 1:31:04 AM CST		

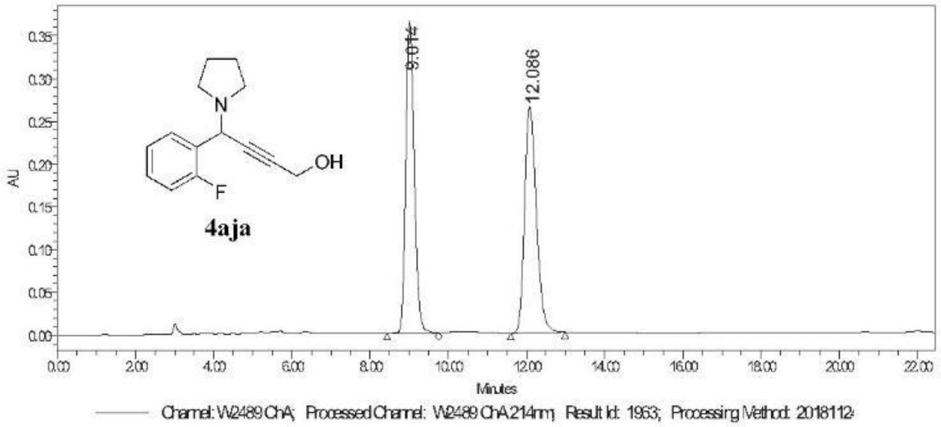

Processed Channel Descr.: W2489 ChA 214nm

	Processed Channel Descr.	RT	Area	%Area	Height
1	W2489 ChA 214nm	9.014	5470880	49.91	365156
2	W2489 ChA 214nm	12.086	5490910	50.09	264065

Reported by User: System
Report Method: Injection Summary Report
Report Method ID: 1639 1639
Page: 1 of 1

Project Name: HPLC
Date Printed:
12/25/2018
1:46:12 AM PRC

HPLC spectrum for (±)-4aja

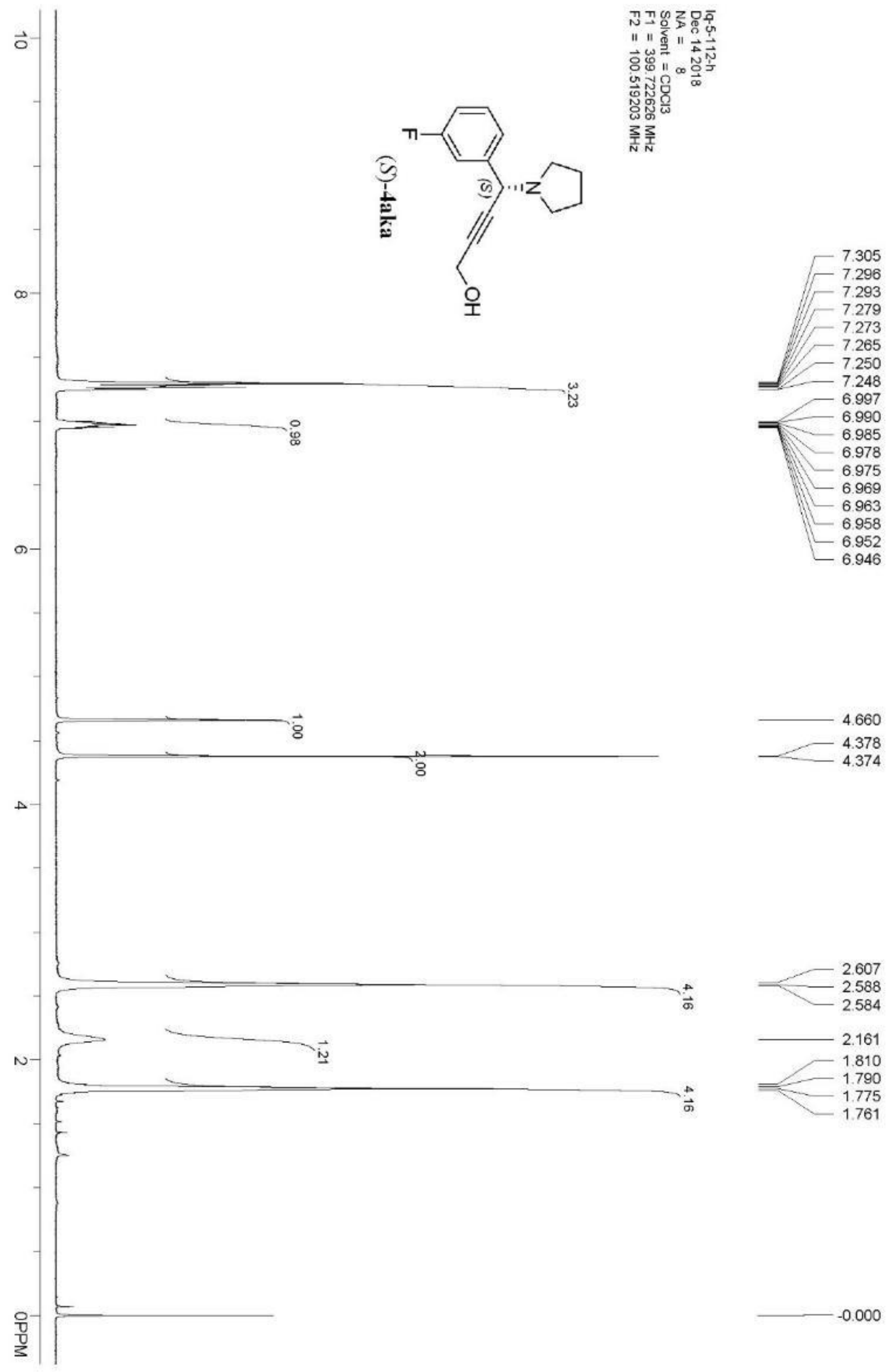

^{1}H NMR (400 MHz, CDCl3) spectrum for (*S*)-4aka

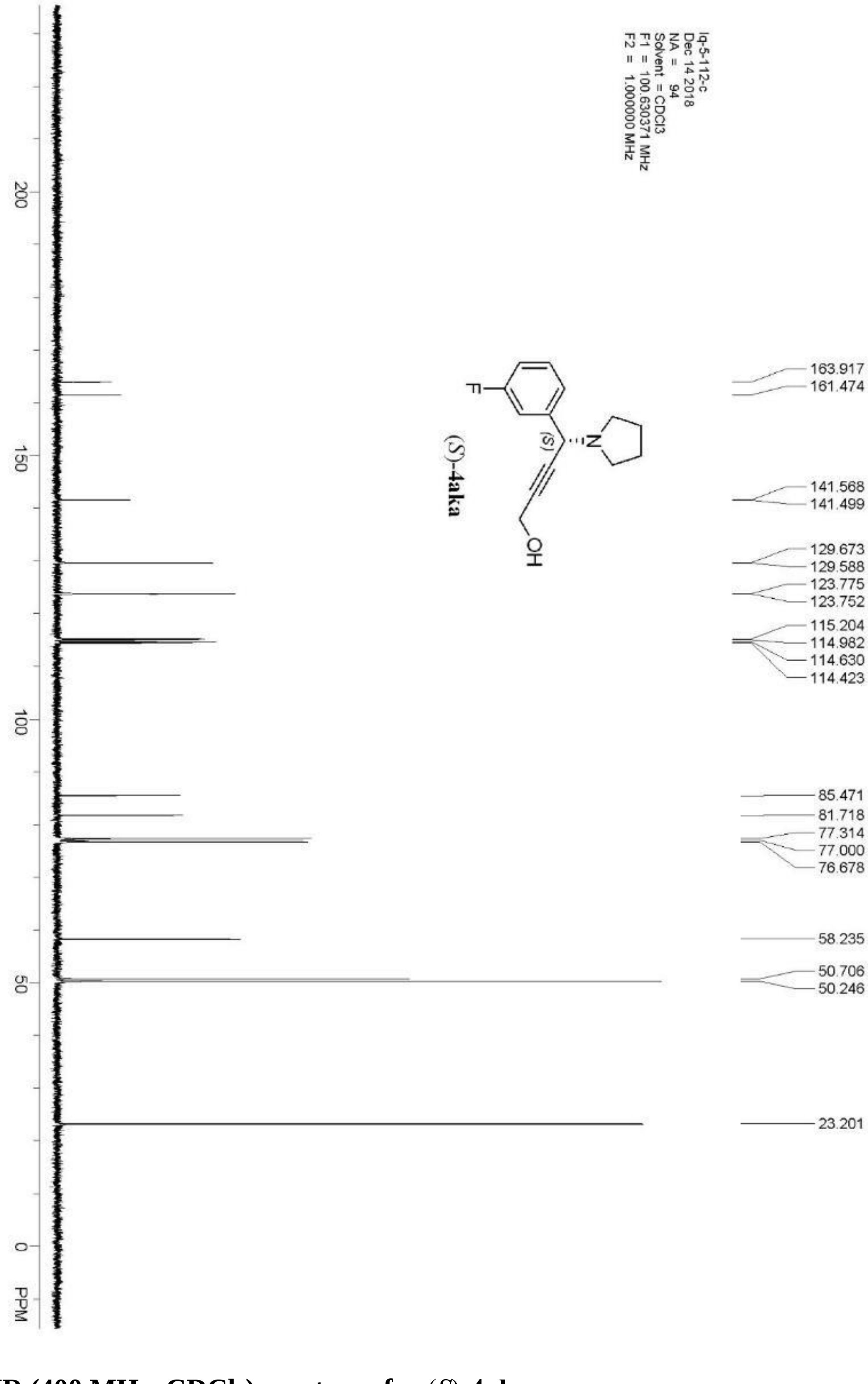

^{13}C NMR (400 MHz, CDCl3) spectrum for (*S*)-4aka

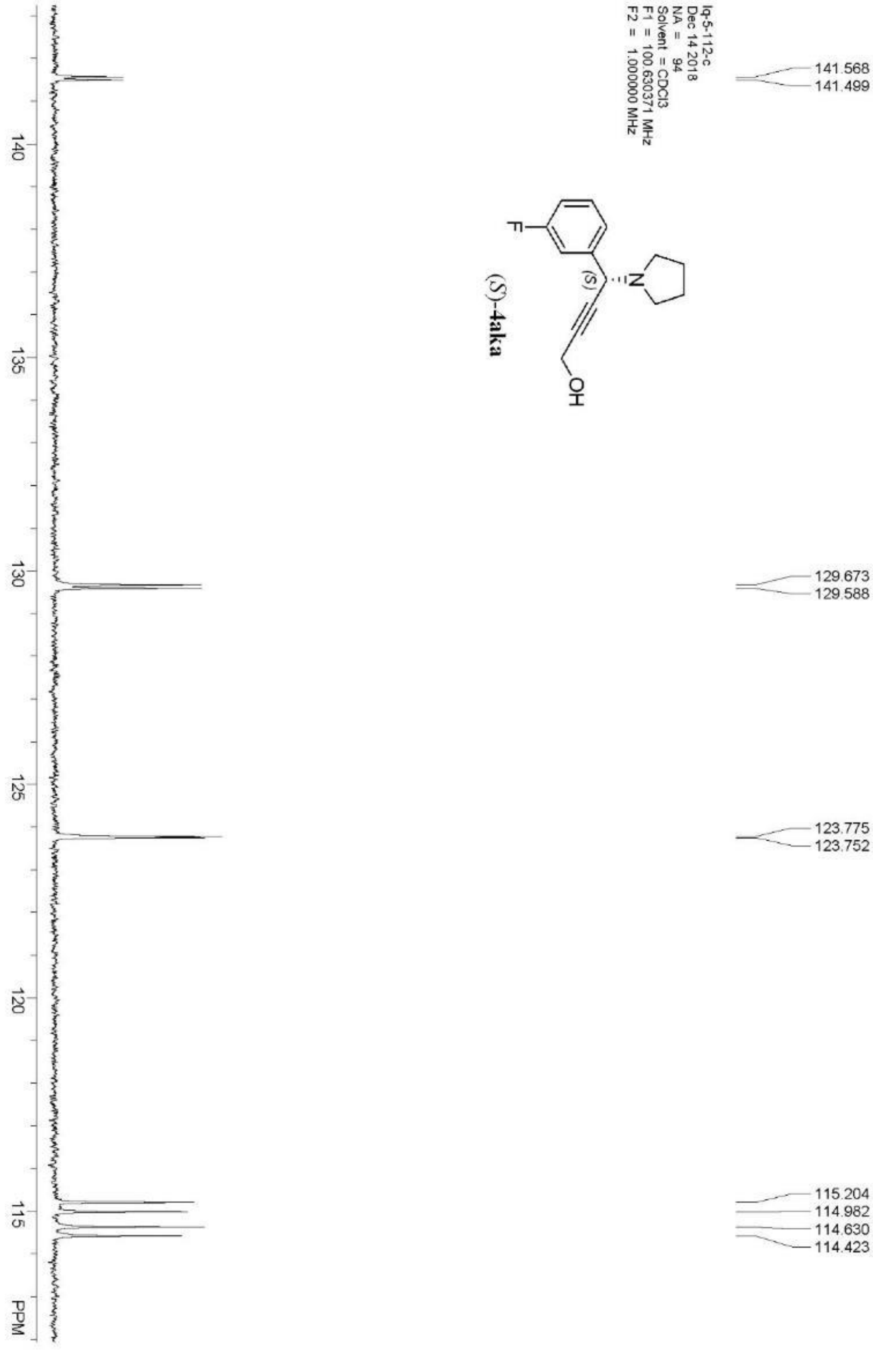

^{13}C NMR (400 MHz, $CDCl_3$) spectrum for (*S*)-4aka

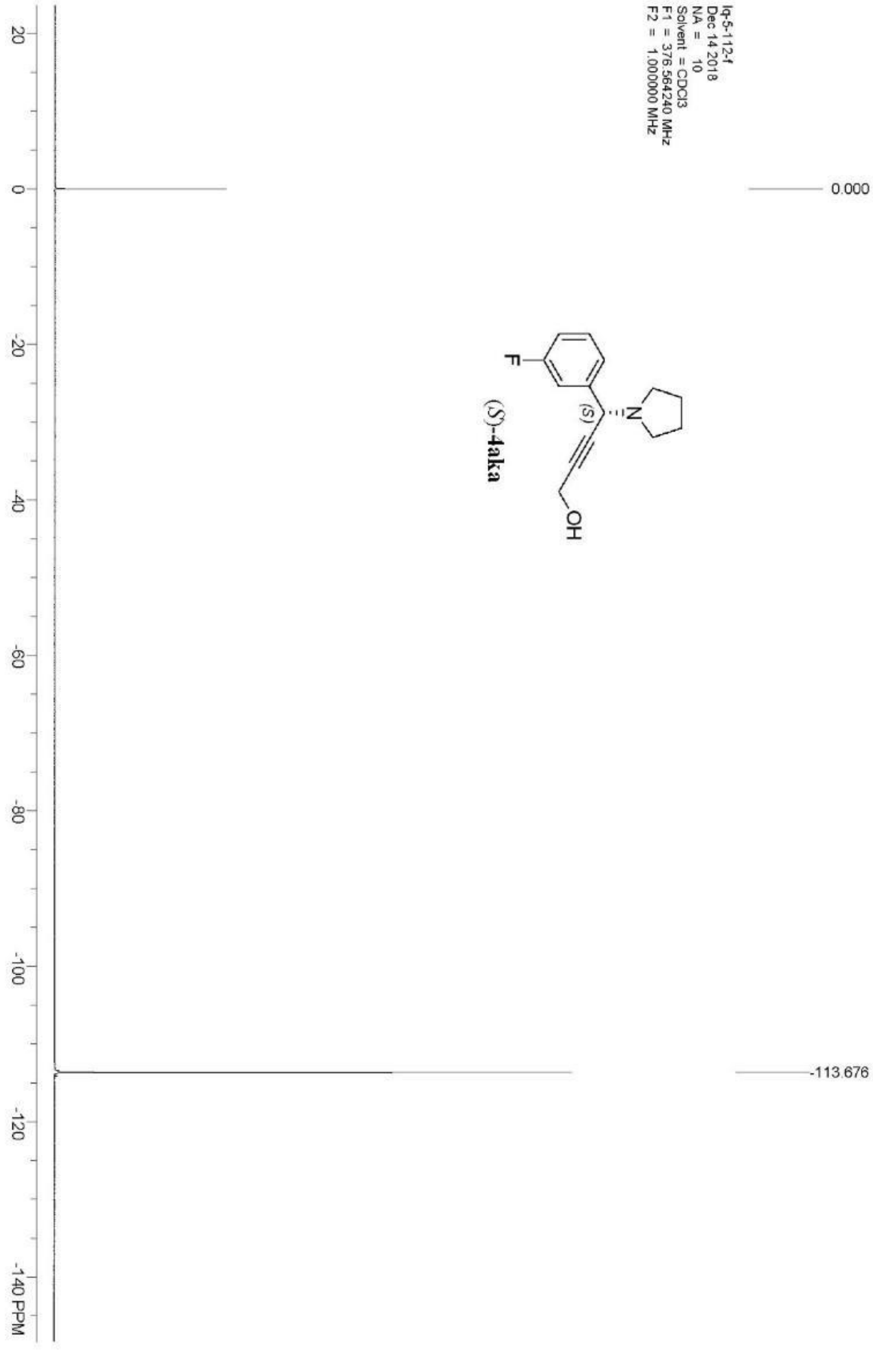

^{19}F NMR (376 MHz, $CDCl_3$) spectrum for (*S*)-4aka ($CFCl_3$ was used as the internal standard)

Empower 3 SOFTWARE

Injection Summary Report

SAMPLE INFORMATION

Sample Name:	lq-5-112-od-h-95-5-0.7-214	Acquired By:	System
Sample Type:	Unknown	Sample Set Name:	
Vial:	1	Acq. Method Set:	HPLC
Injection #:	1	Processing Method:	20181124
Injection Volume:	5.00 ul	Channel Name:	W2489 ChA
Run Time:	60.0 Minutes	Proc. Chnl. Descr.:	W2489 ChA 214nm
Date Acquired:	12/15/2018 3:28:33 AM CST		
Date Processed:	12/15/2018 3:53:22 AM CST		

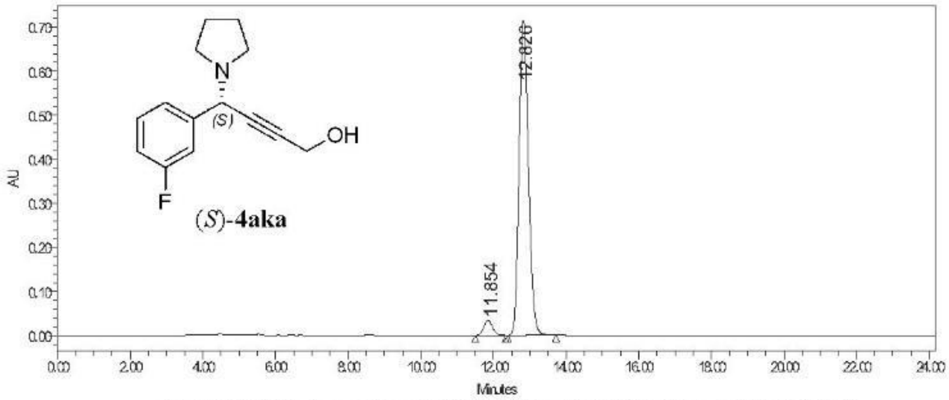

Channel: W2489 ChA; Processed Channel: W2489 ChA 214nm; Result Id: 1750; Processing Method: 2018112

Processed Channel Descr.: W2489 ChA 214nm

	Processed Channel Descr.	RT	Area	%Area	Height
1	W2489 ChA 214nm	11.854	549578	4.13	34485
2	W2489 ChA 214nm	12.826	12756887	95.87	715076

Reported by User: System
Report Method: Injection Summary Report
Report Method ID: 1639 1639
Page: 1 of 1

Project Name: HPLC
Date Printed:
12/15/2018
3:53:39 AM PRC

HPLC spectrum for (*S*)-4aka

Empower 3 SOFTWARE

Injection Summary Report

SAMPLE INFORMATION

Sample Name:	xhb-1-124-od-h-95-5-0.7-214	Acquired By:	System
Sample Type:	Unknown	Sample Set Name:	
Vial:	1	Acq. Method Set:	HPLC
Injection #:	1	Processing Method:	20181124
Injection Volume:	5.00 ul	Channel Name:	W2489 ChA
Run Time:	60.0 Minutes	Proc. Chnl. Descr.:	W2489 ChA 214nm
Date Acquired:	12/15/2018 12:59:10 AM CST		
Date Processed:	12/15/2018 1:24:33 AM CST		

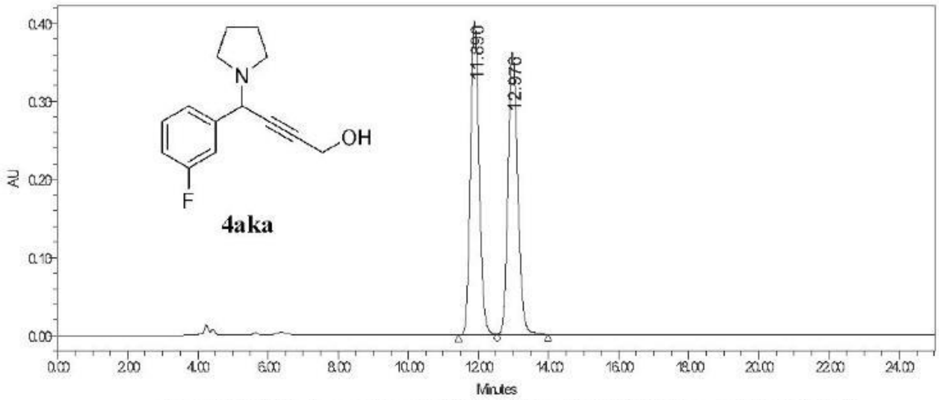

Channel: W2489 ChA; Processed Channel: W2489 ChA 214nm; Result Id: 1746; Processing Method: 2018112

Processed Channel Descr.: W2489 ChA 214nm

	Processed Channel Descr.	RT	Area	%Area	Height
1	W2489 ChA 214nm	11.890	6884441	49.81	402649
2	W2489 ChA 214nm	12.976	6937877	50.19	362782

Reported by User: System
Report Method: Injection Summary Report
Report Method ID: 1639 1639
Page: 1 of 1

Project Name: HPLC
Date Printed:
12/15/2018
1:24:51 AM PRC

HPLC spectrum for (±)-4aka

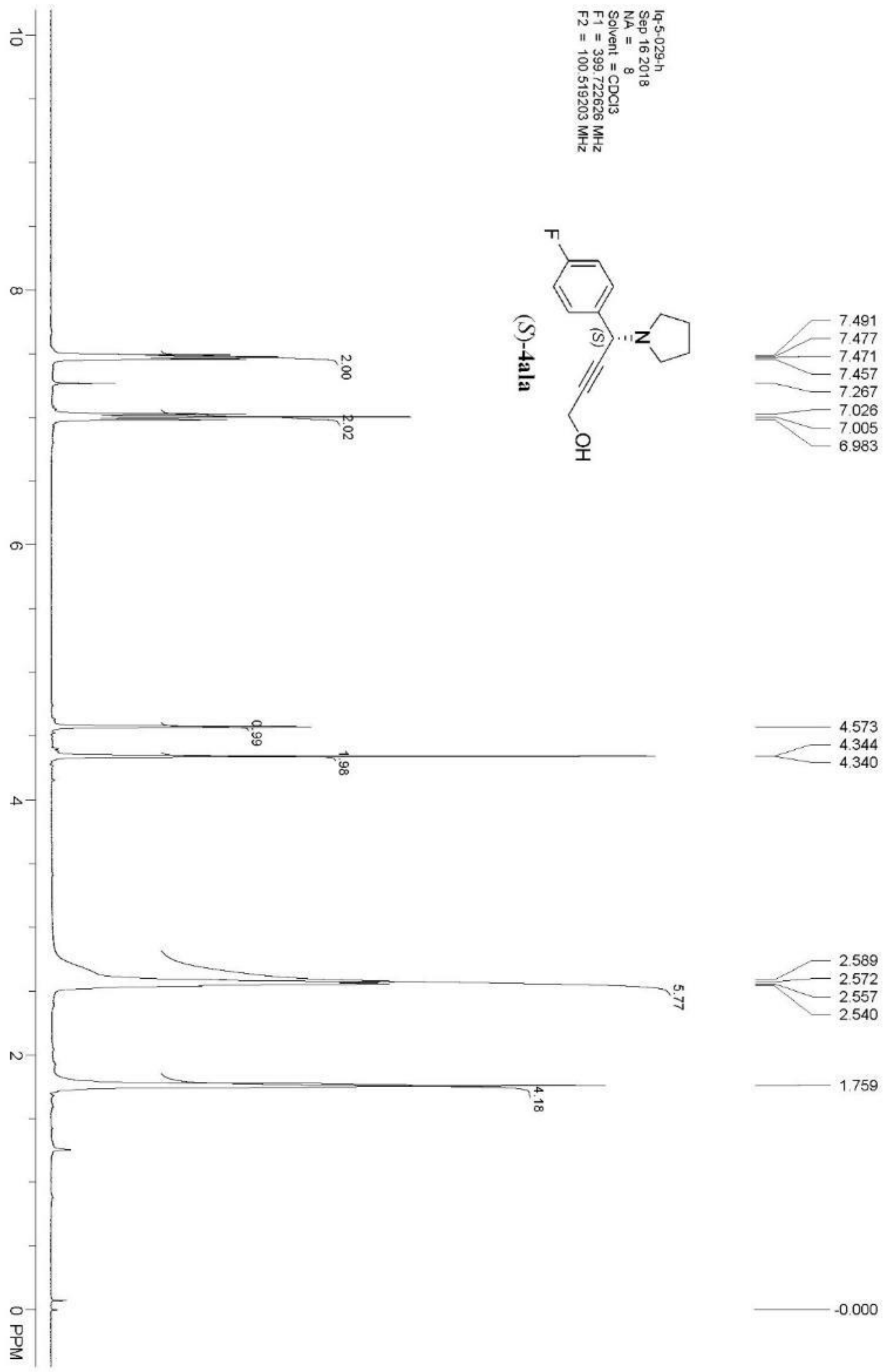

¹H NMR (400 MHz, CDCl3) spectrum for (*S*)-4ala

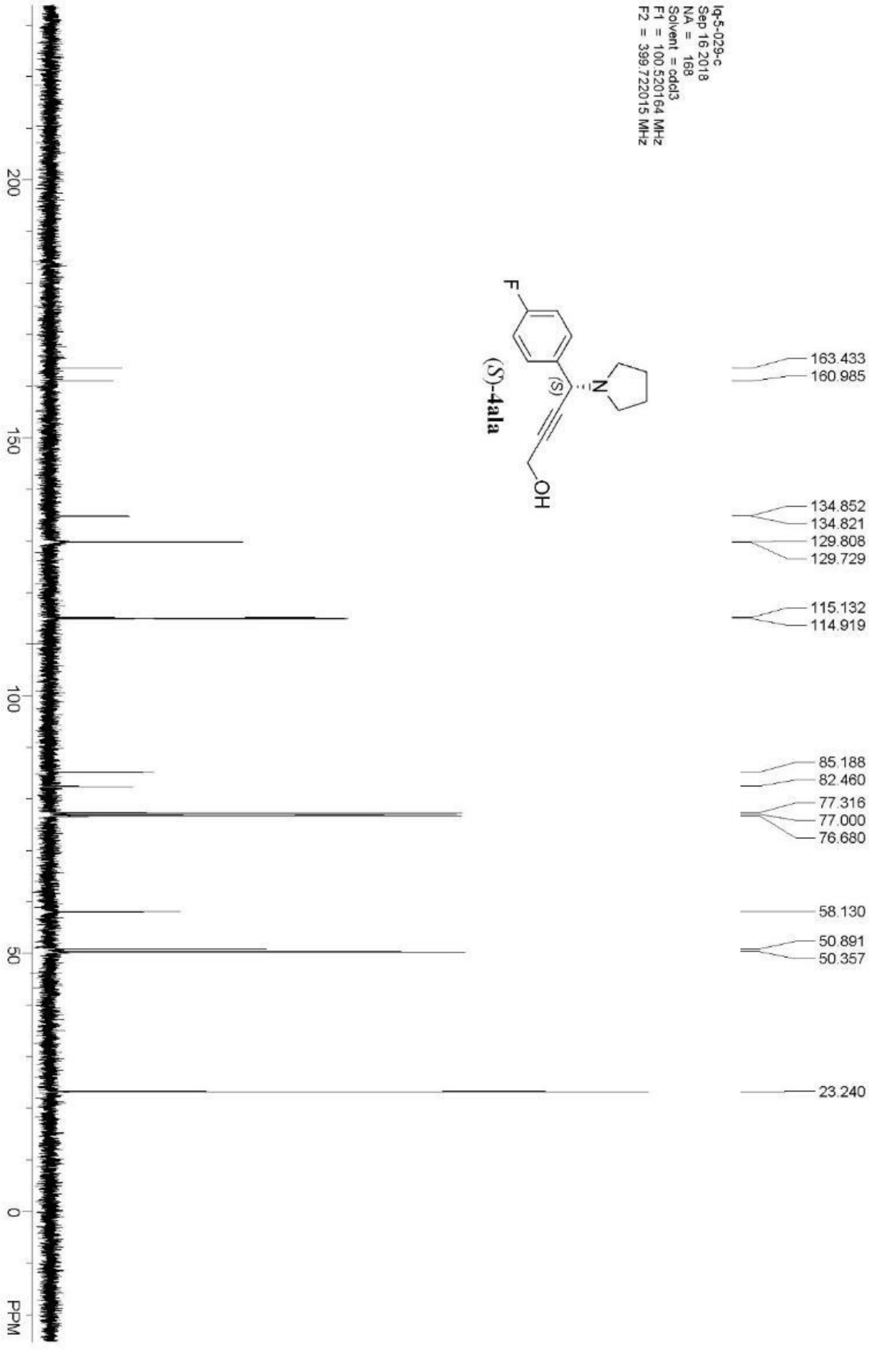

^{13}C NMR (400 MHz, $CDCl_3$) spectrum for (*S*)-4ala

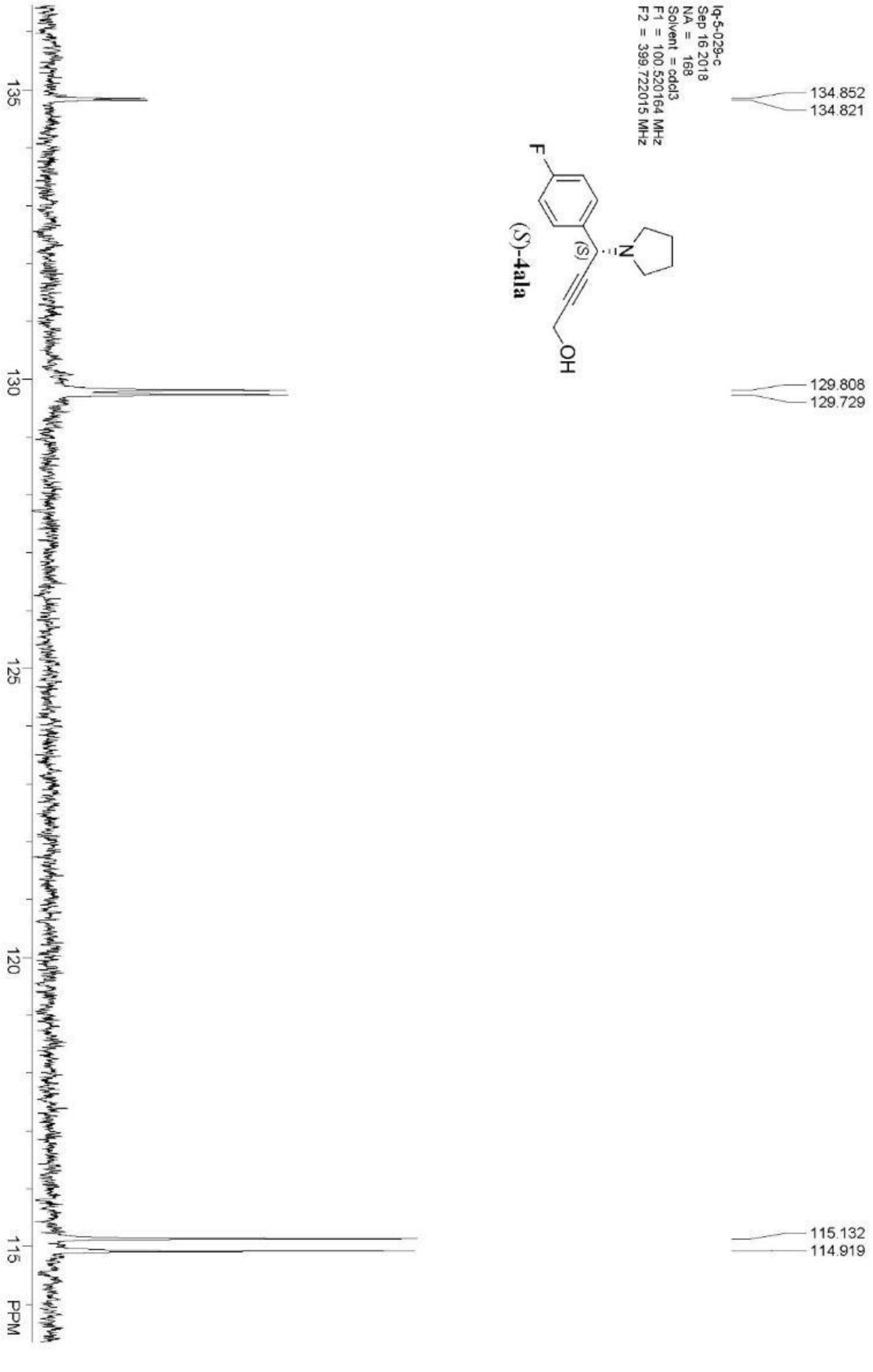

^{13}C NMR (400 MHz, $CDCl_3$) spectrum for (*S*)-4ala

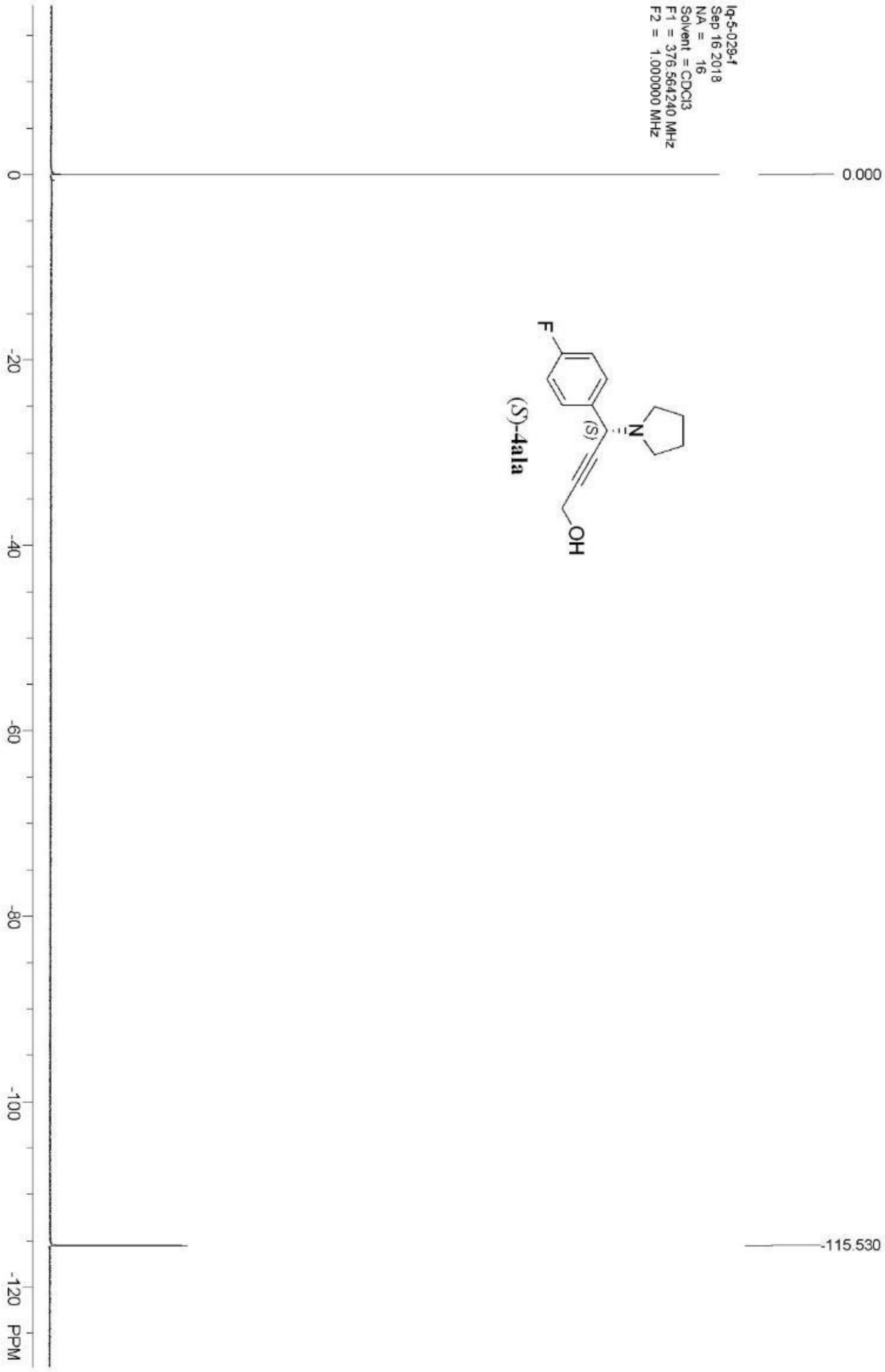

^{19}F NMR (376 MHz, $CDCl_3$) spectrum for (*S*)-4ala ($CFCl_3$ was used as the internal standard)

lq-5-029

实验时间：2018-09-15, 19:11:20　　　　　　　报告时间：2018-09-17, 12:28:10
谱图文件:D:\data\slf\lq\2018-09-15\lq-5-029-ad-h-90+10-0.7-214.org

实验内容简介：
AD-H　90+10
214nm　0.7ml/min

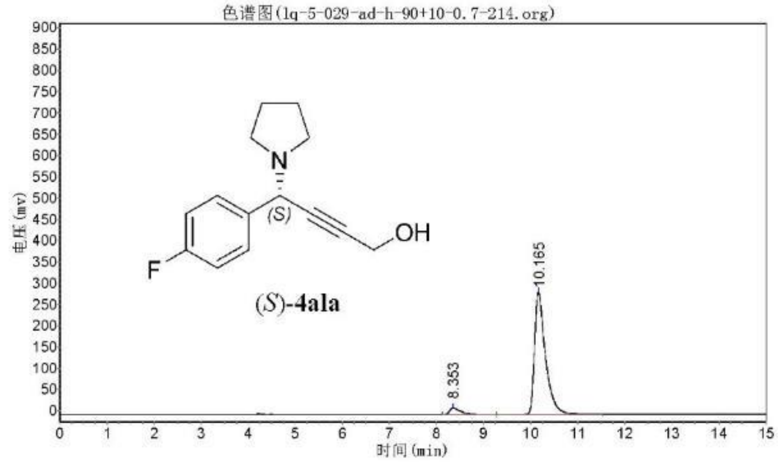

分析结果表

峰号	峰名	保留时间	峰高	峰面积	含量
1		8.353	15877.740	250364.766	5.1403
2		10.165	287059.063	4620270.000	94.8597
**总计**			302936.803	4870634.766	100.0000

HPLC spectrum for (*S*)-4ala

xhb-1-044

实验时间：2018-09-15, 19:49:13　　　　　　　　　　　　　报告时间：2018-09-17, 12:30:38
谱图文件:D:\data\slf\lq\2018-09-15\xhb-1-044-ad-h-90+10-0.7-214-02.org

实验内容简介：
AD-H　90+10
214nm　0.7ml/min

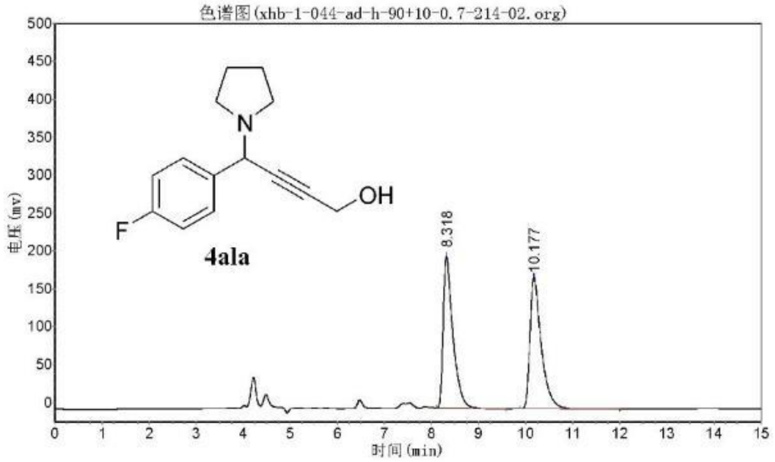

分析结果表

峰号	峰名	保留时间	峰高	峰面积	含量
1		8.318	200698.875	2865983.000	49.8575
2		10.177	173273.094	2882363.000	50.1425
**总计**			373971.969	5748346.000	100.0000

HPLC spectrum for (±)-4ala

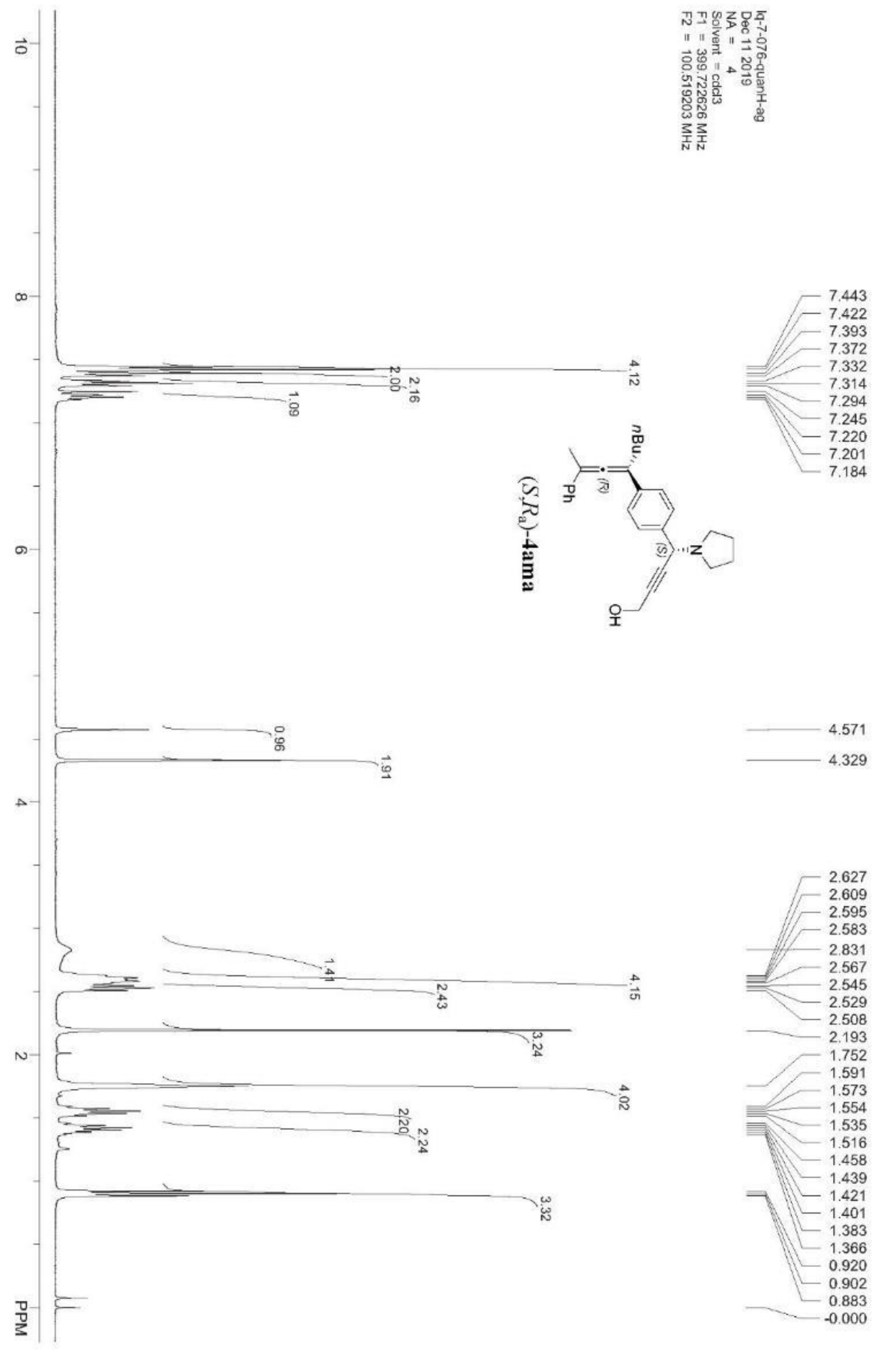

^{1}H NMR (400 MHz, CDCl3) spectrum for (S,R_a)**-4ama**

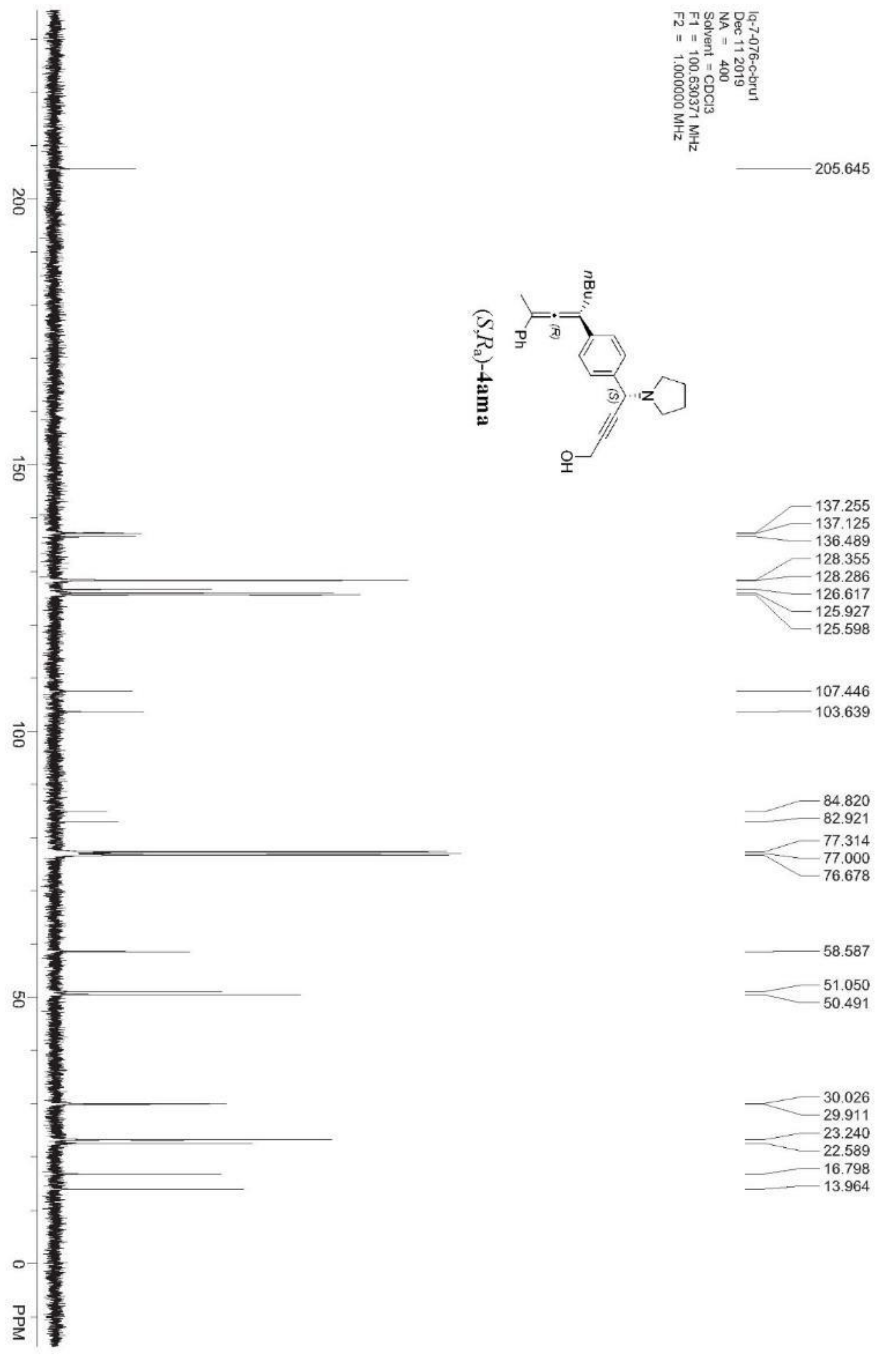

^{13}C NMR (400 MHz, CDCl$_3$) spectrum for (*S*,*R*$_a$)**-4ama**

Empower 3

Injection Summary Report

SAMPLE INFORMATION

Sample Name:	lq-7-076-ad-h-95-5-1-214	Acquired By:	System
Sample Type:	Unknown	Sample Set Name:	
Vial:	1	Acq. Method Set:	HPLC
Injection #:	1	Processing Method:	Default
Injection Volume:	5.00 ul	Channel Name:	W2489 ChA
Run Time:	20.0 Minutes	Proc. Chnl. Descr.:	W2489 ChA 214nm
Date Acquired:	12/11/2019 1:47:05 PM CST		
Date Processed:	12/12/2019 1:57:41 AM CST		

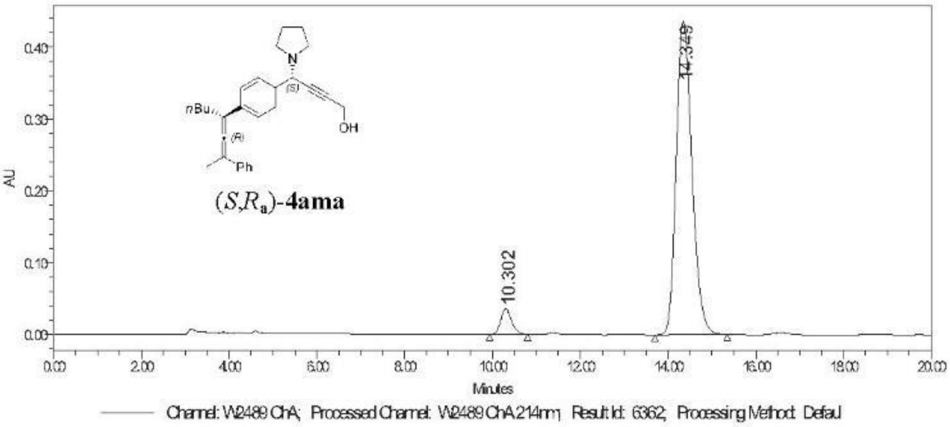

Processed Channel Descr.: W2489 ChA 214nm

	Processed Channel Descr.	RT	Area	%Area	Height
1	W2489 ChA 214nm	10.302	602437	5.27	35794
2	W2489 ChA 214nm	14.349	10838215	94.73	436724

Reported by User: System
Report Method: Injection Summary Report
Report Method ID: 1639 1639
Page: 1 of 1

Project Name: HPLC
Date Printed:
12/12/2019
1:58:11 AM PRC

HPLC spectrum for (S,R_a)**-4ama**

Empower 3 SOFTWARE

Injection Summary Report

SAMPLE INFORMATION

Sample Name:	lq-7-072-ad-h-95-5-1-214	Acquired By:	System
Sample Type:	Unknown	Sample Set Name:	
Vial:	1	Acq. Method Set:	HPLC
Injection #:	3	Processing Method:	Default
Injection Volume:	5.00 ul	Channel Name:	W2489 ChA
Run Time:	20.0 Minutes	Proc. Chnl. Descr.:	W2489 ChA 214nm
Date Acquired:	12/11/2019 2:32:59 PM CST		
Date Processed:	12/12/2019 1:56:42 AM CST		

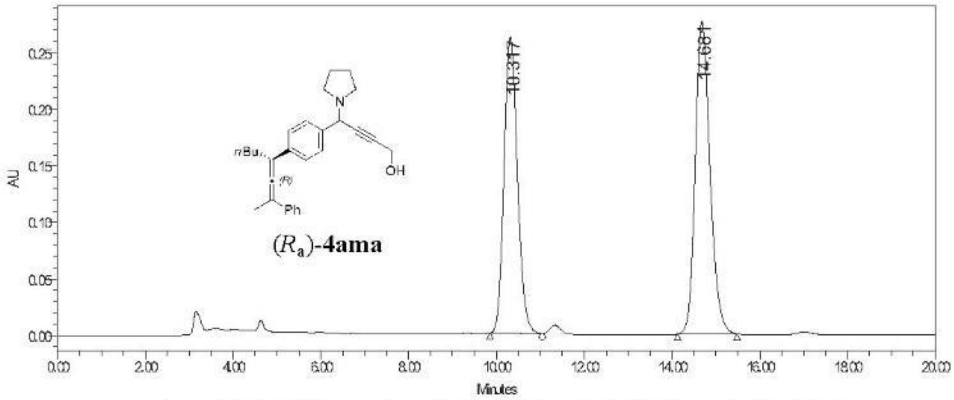

Processed Channel Descr.: W2489 ChA 214nm

	Processed Channel Descr.	RT	Area	%Area	Height
1	W2489 ChA 214nm	10.317	5539292	46.28	262242
2	W2489 ChA 214nm	14.681	6429939	53.72	276596

Reported by User: System
Report Method: Injection Summary Report
Report Method ID: 1639 1639
Page: 1 of 1

Project Name: HPLC
Date Printed:
12/12/2019
1:59:10 AM PRC

HPLC spectrum for (R_a)-4ama

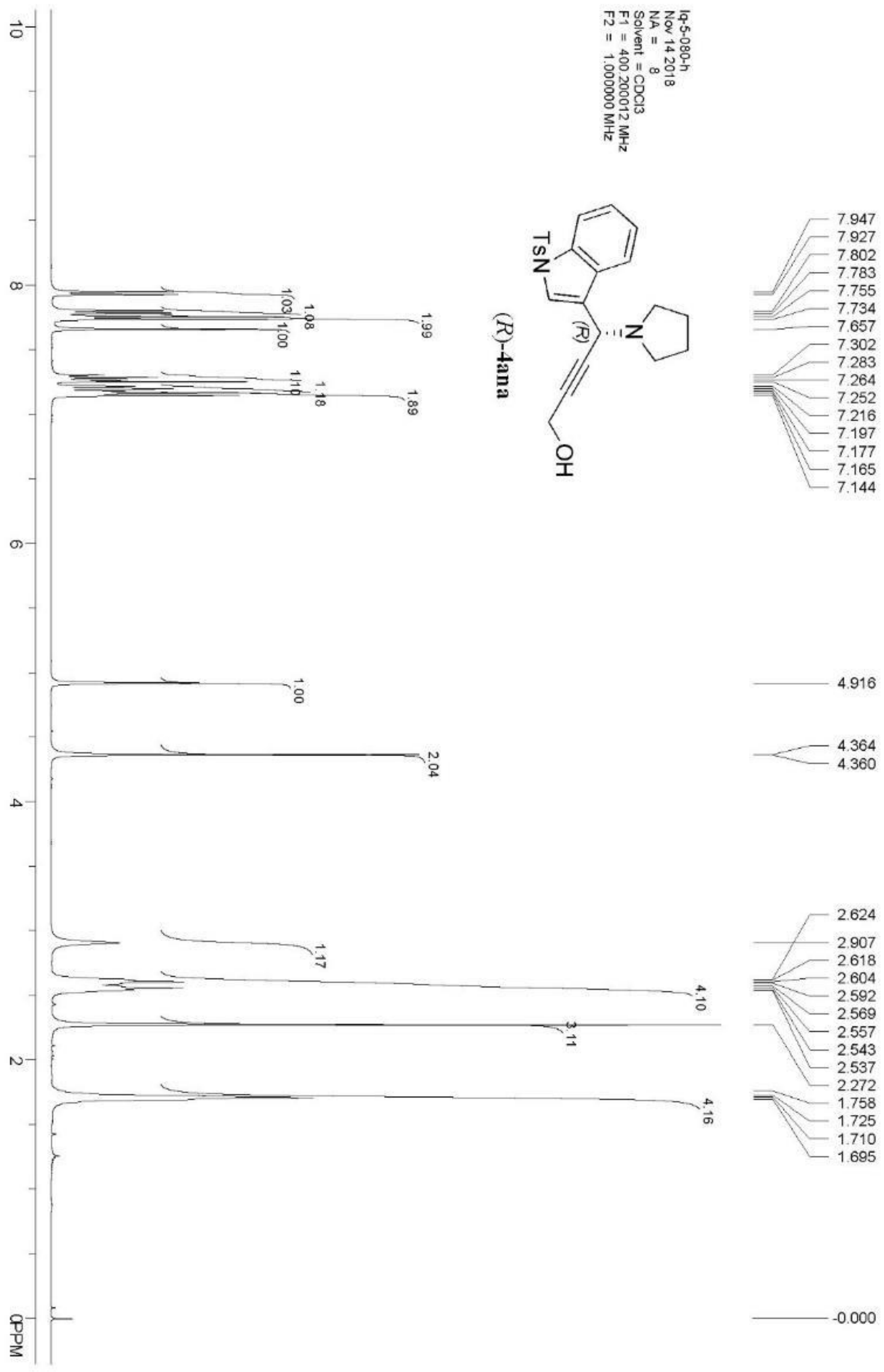

^{1}H NMR (400 MHz, CDCl3) spectrum for (*R*)**-4ana**

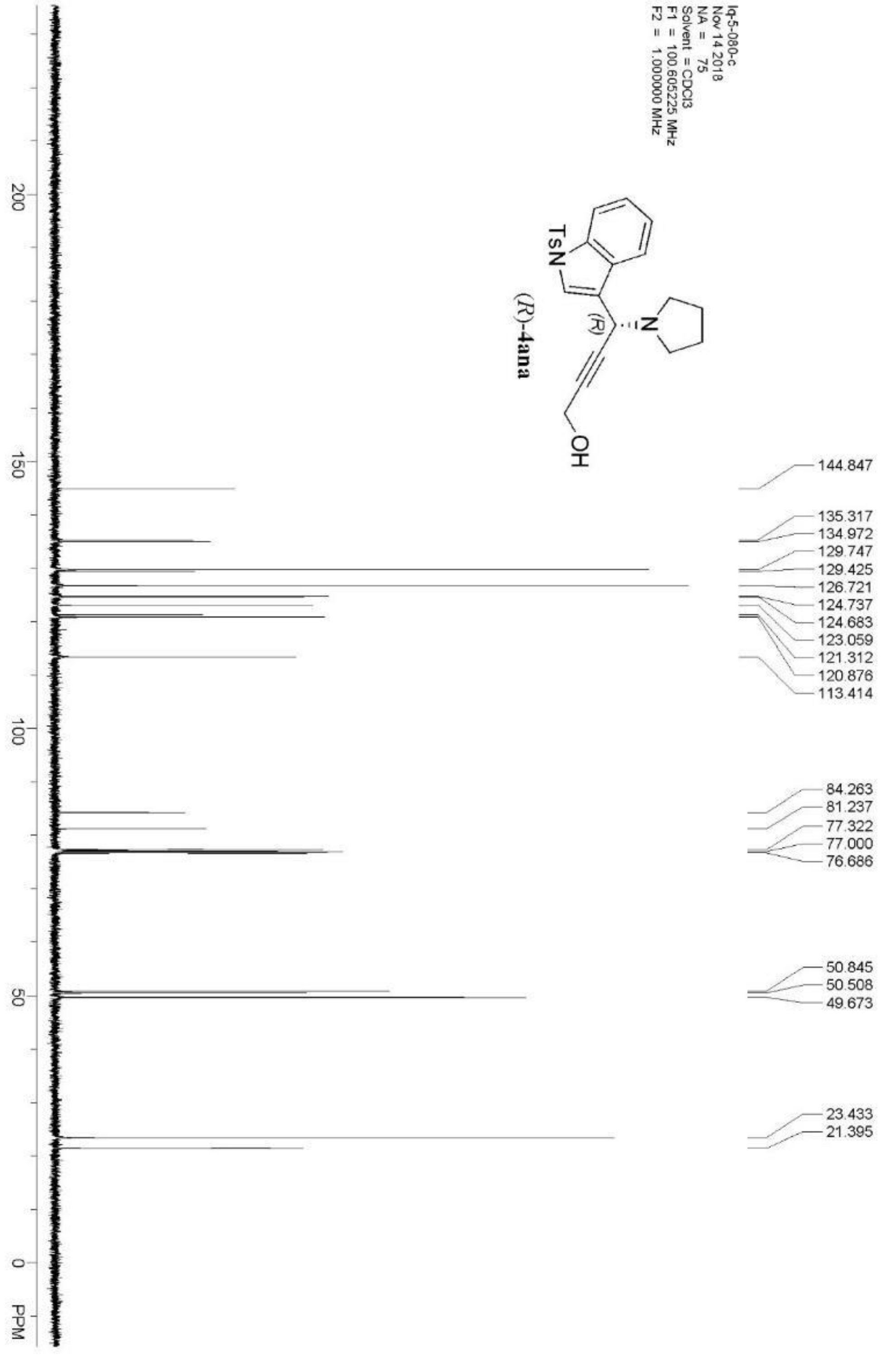

^{13}C NMR (400 MHz, CDCl$_3$) spectrum for (*R*)-4ana

LQ-5-080

实验时间：2018/11/14, 9:26:24　　　　报告时间：2018/11/14, 10:30:48
谱图文件:D:\data\slf\lq\2018-11-14\lq-5-080-AD-H-90-10-1.0-214-2.org

实验内容简介：
AD-H　90:10
214nm　1.0ml/min

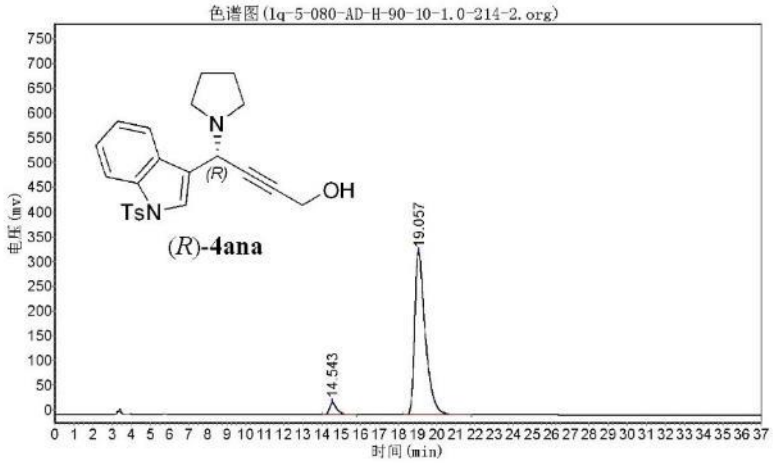

分析结果表

峰号	峰名	保留时间	峰高	峰面积	含量
1		14.543	24356.762	643406.375	4.8202
2		19.057	329010.844	12704839.000	95.1798
**总计**			353367.605	13348245.375	100.0000

HPLC spectrum for (*R*)-4ana

XHB-1-095

实验时间：2018/11/14, 10:09:12　　　　报告时间：2018/11/14, 10:42:13
谱图文件:D:\data\slf\lq\2018-11-14\xhb-1-095-AD-H-90+10-1-214.org

实验内容简介：
AD-H　90:10
214nm　1.0ml/min

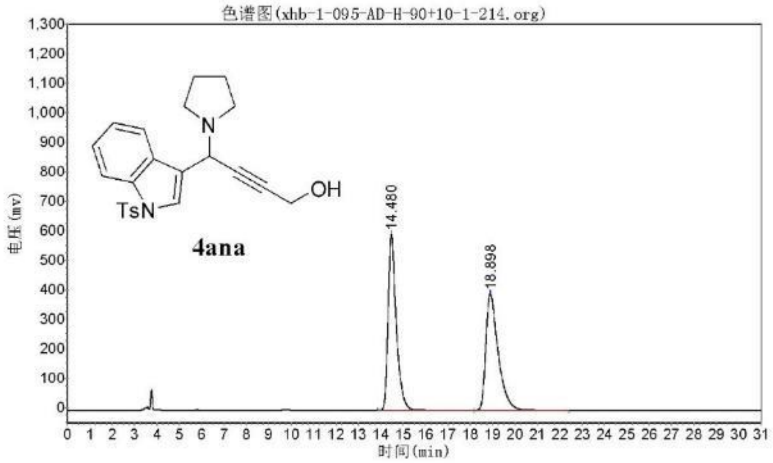

分析结果表

峰号	峰名	保留时间	峰高	峰面积	含量
1		14.480	595919.938	15132521.000	50.0863
2		18.898	393341.875	15080369.000	49.9137
总计			989261.813	30212890.000	100.0000

HPLC spectrum for (±)-4ana

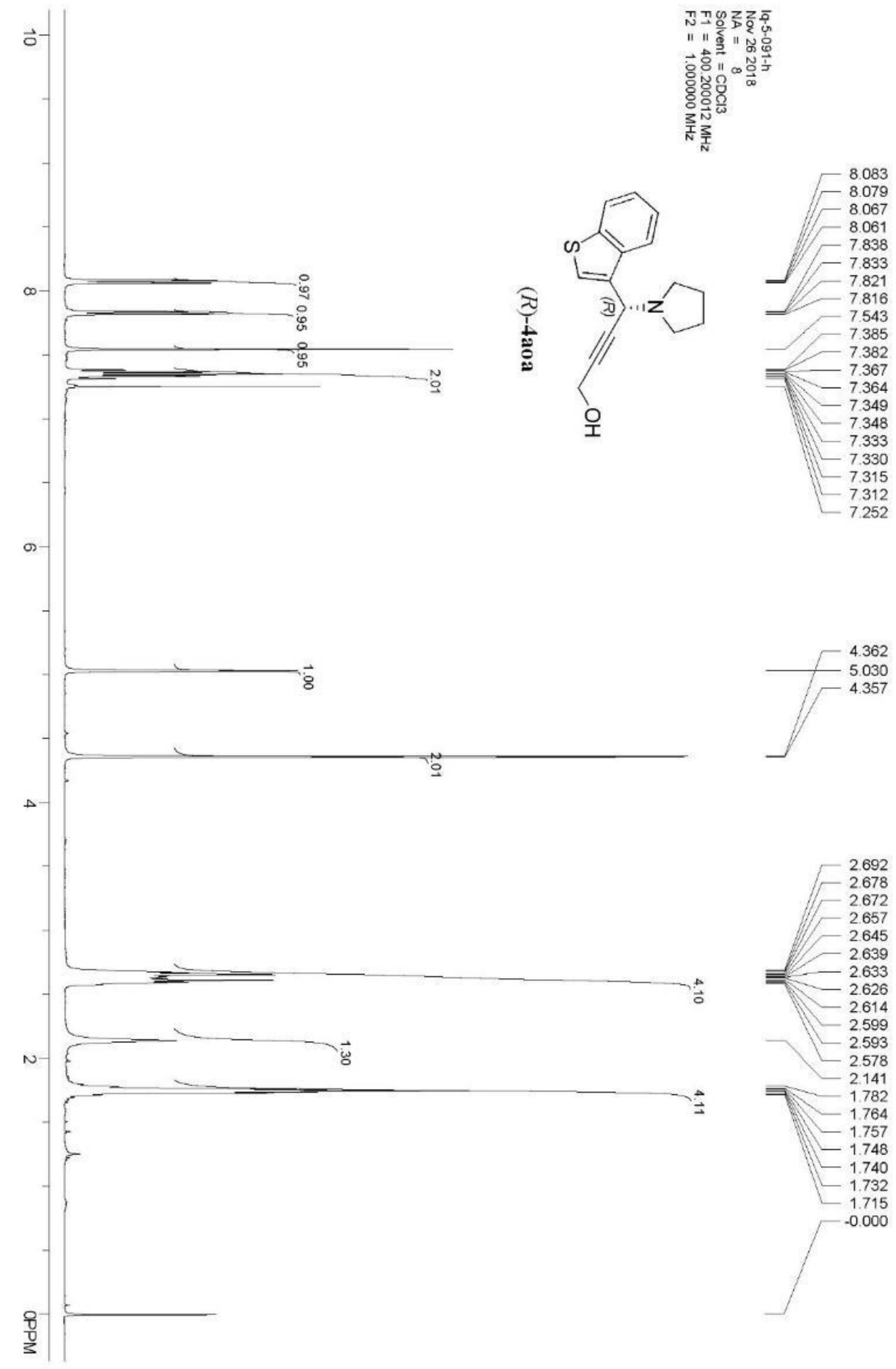

^{1}H NMR (400 MHz, CDCl3) spectrum for (*R*)**-4aoa**

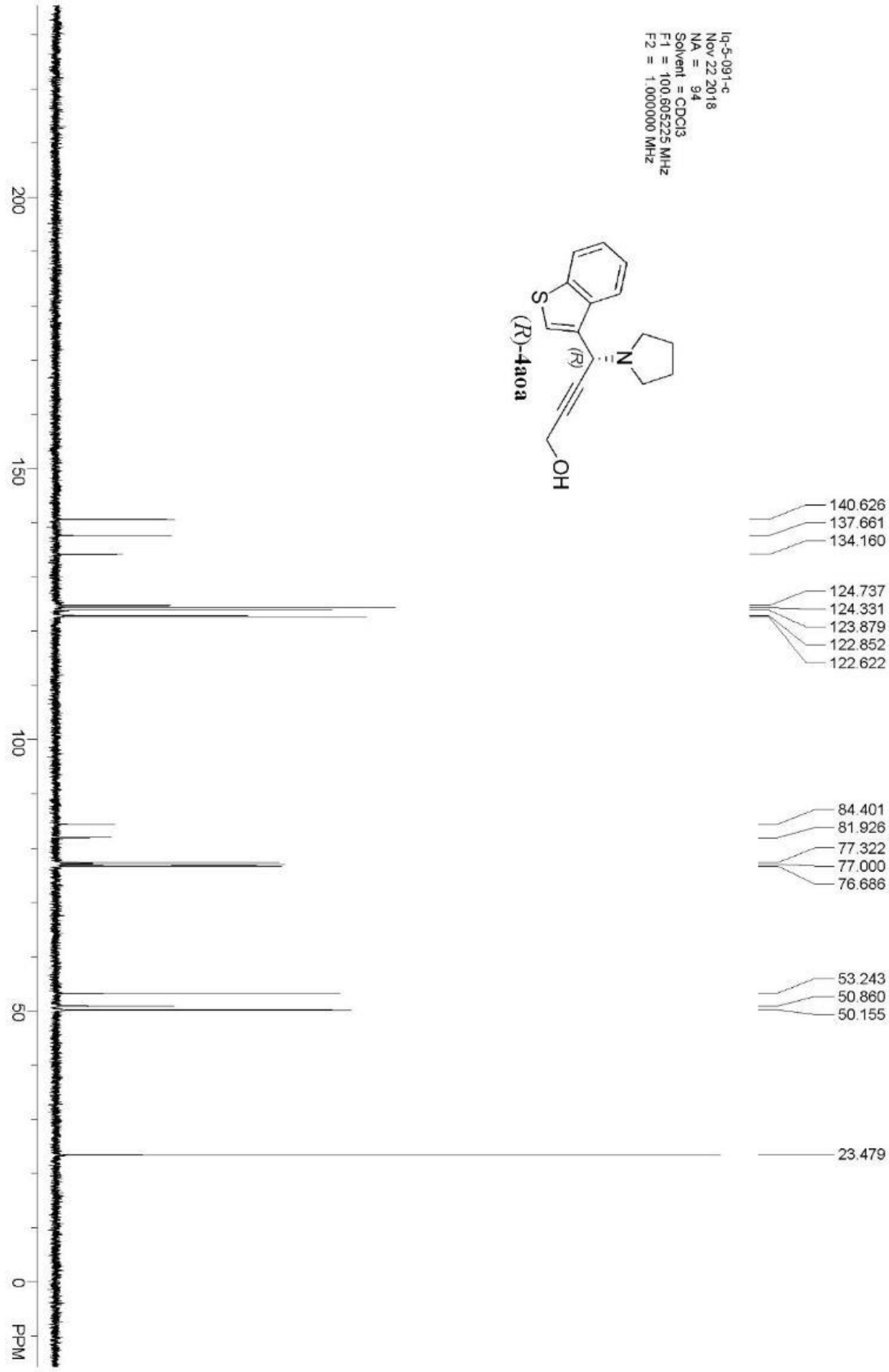

^{13}C NMR (400 MHz, $CDCl_3$) spectrum for (*R*)-4aoa

1q-5-091

实验时间：2018-11-22, 13:02:22　　　　　　　　　　报告时间：2018-11-22, 13:34:13
谱图文件:D:\data\slf\lq\2018-11-22\lq-5-091-ad-h-95-5-1.0-214-3.org

实验内容简介：
ad-h　　95:5
1.0ml/min　214nm

色谱图(lq-5-091-ad-h-95-5-1.0-214-3.org)

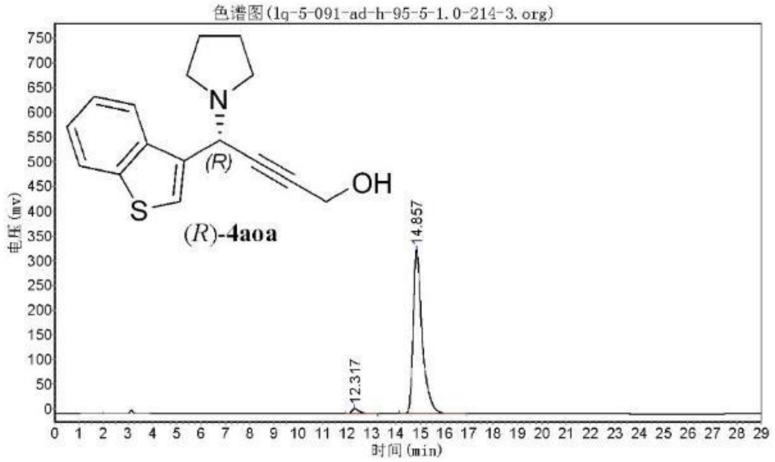

分析结果表

峰号	峰名	保留时间	峰高	峰面积	含量
1		12.317	10308.856	214621.266	2.4513
2		14.857	331567.406	8540845.000	97.5487
**总计**			341876.263	8755466.266	100.0000

HPLC spectrum for (*R*)-4aoa

xhb-1-106

实验时间：2018-11-22, 12:12:01　　　　　　　　　　　　　报告时间：2018-11-22, 12:12:04
谱图文件:D:\data\slf\lq\2018-11-22\xhb-1-106.mdy

实验内容简介：
ad-h　　95:5
1.0ml/min 214nm

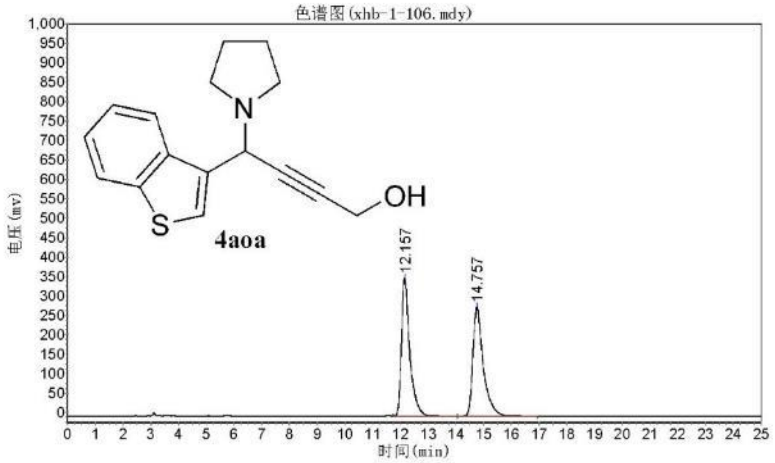

分析结果表

峰号	峰名	保留时间	峰高	峰面积	含量
1		12.157	355354.250	7282050.500	50.0209
2		14.757	280811.219	7275975.500	49.9791
**总计**			636165.469	14558026.000	100.0000

HPLC spectrum for (±)-4aoa

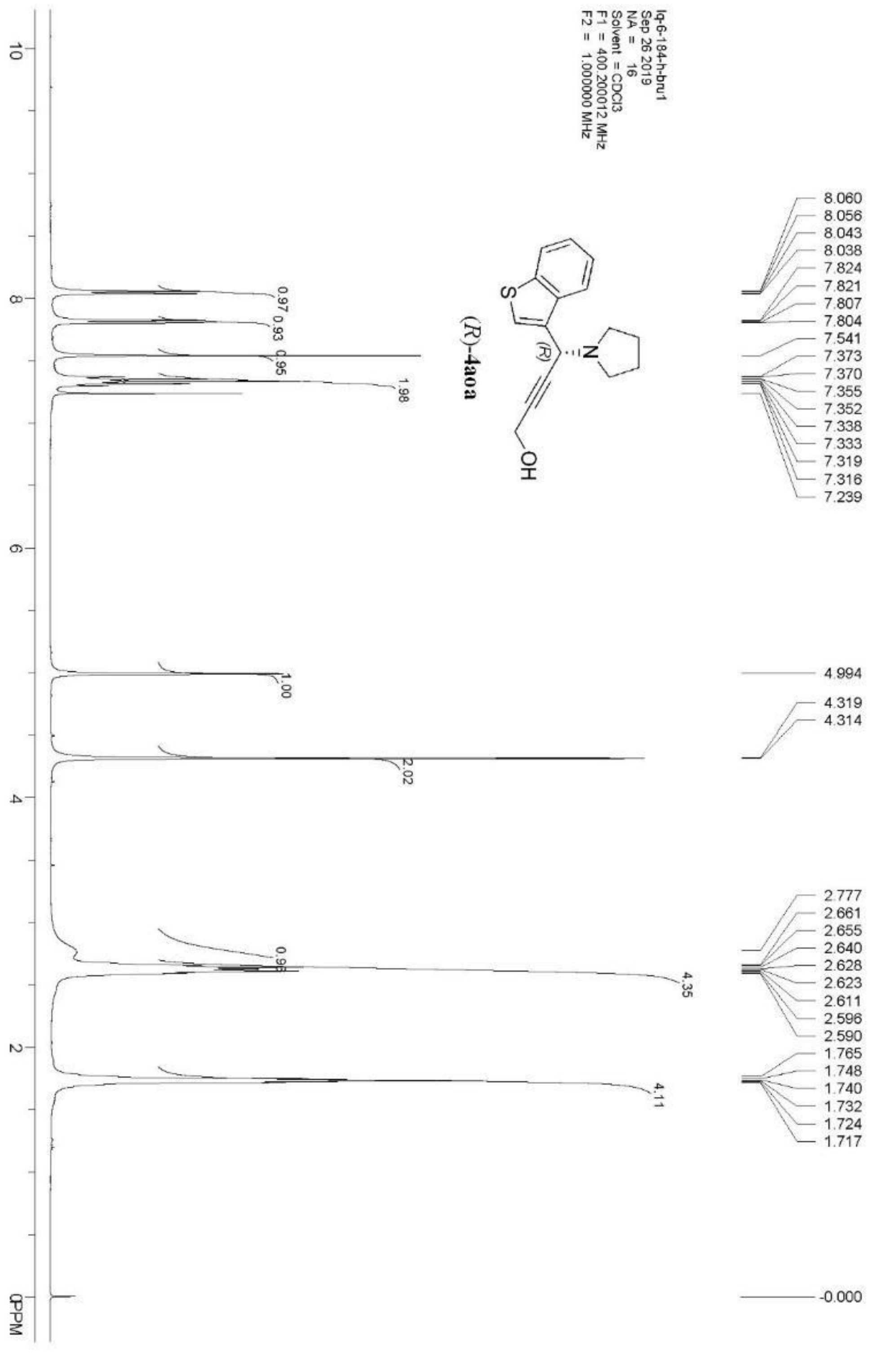

^{1}H NMR (400 MHz, CDCl3) spectrum for (*R*)**-4aoa**

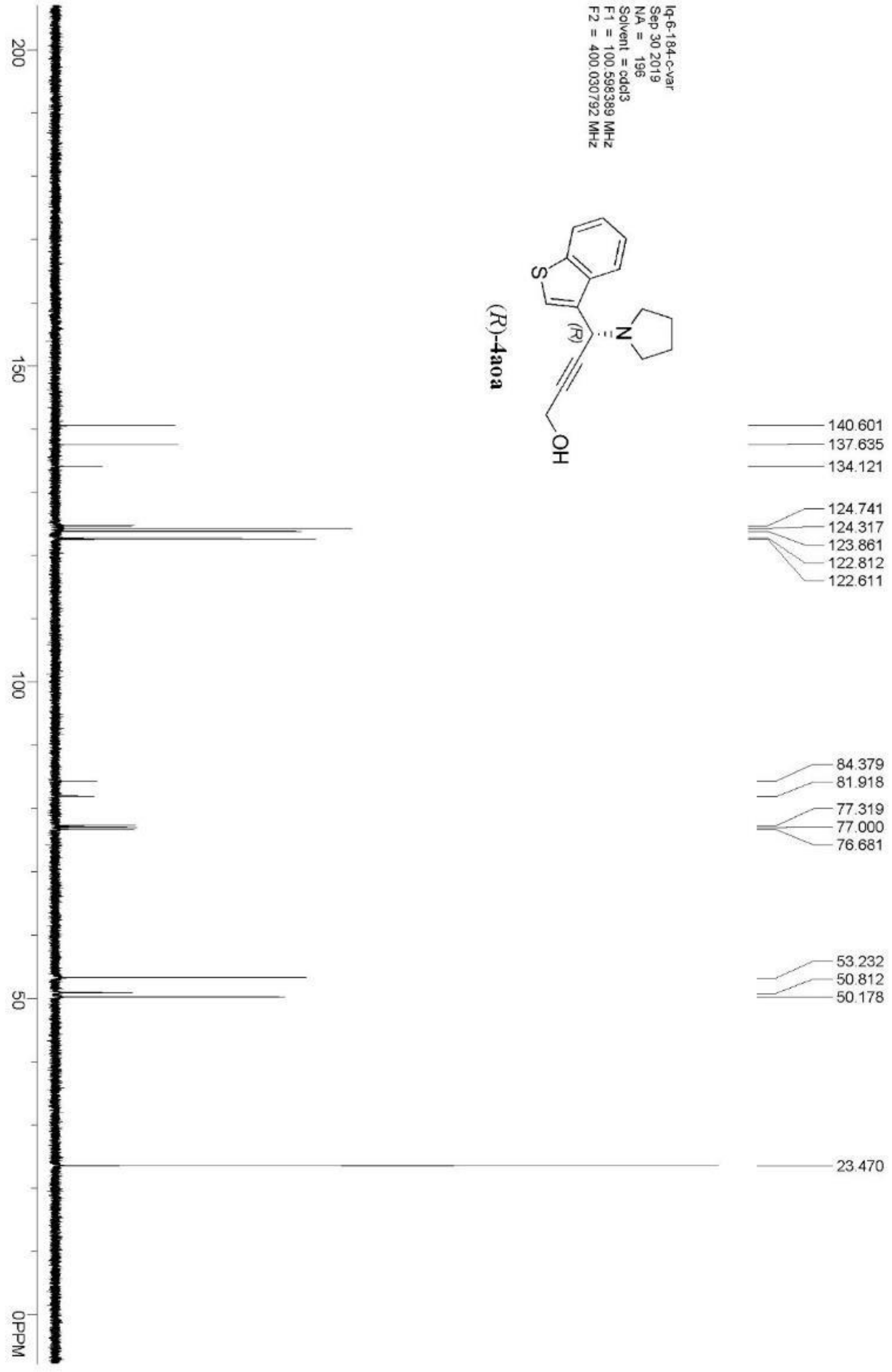

^{13}C NMR (400 MHz, CDCl$_3$) spectrum for (*R*)**-4aoa**

Empower 3 SOFTWARE

Injection Summary Report

SAMPLE INFORMATION

Sample Name:	lq-6-184-ad-h-95-5-1-214	Acquired By:	System
Sample Type:	Unknown	Sample Set Name:	
Vial:	1	Acq. Method Set:	HPLC
Injection #:	1	Processing Method:	Default
Injection Volume:	2.50 ul	Channel Name:	W2489 ChA
Run Time:	20.0 Minutes	Proc. Chnl. Descr.:	W2489 ChA 214nm
Date Acquired:	9/25/2019 11:29:19 PM CST		
Date Processed:	9/25/2019 11:50:38 PM CST		

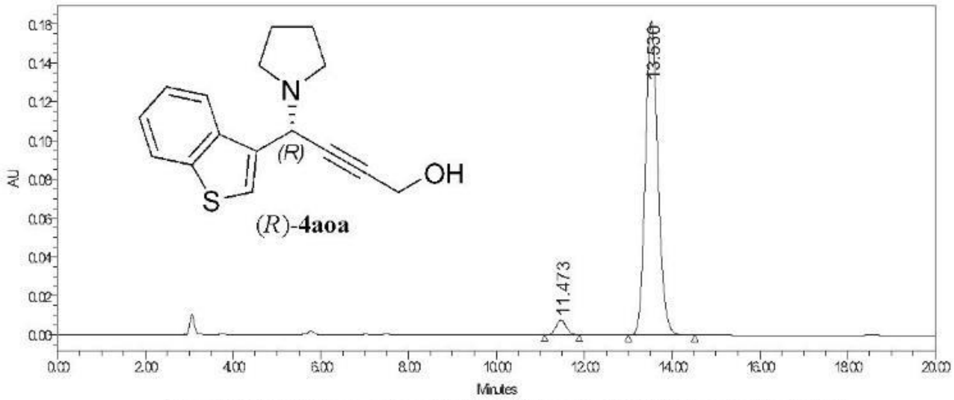

Channel: W2489 ChA; Processed Channel: W2489 ChA 214nm; Result Id: 5163; Processing Method: Defaul

Processed Channel Descr.: W2489 ChA 214nm

	Processed Channel Descr.	RT	Area	%Area	Height
1	W2489 ChA 214nm	11.473	121451	3.65	7703
2	W2489 ChA 214nm	13.530	3202666	96.35	161804

Reported by User: System
Report Method: Injection Summary Report
Report Method ID: 1639 1639
Page: 1 of 1

Project Name: HPLC
Date Printed:
9/26/2019
12:47:49 AM PRC

HPLC spectrum for (*R*)-4aoa

Empower 3 SOFTWARE

Injection Summary Report

SAMPLE INFORMATION

Sample Name:	xhb-1-106-adh-95-5-1-214	Acquired By:	System
Sample Type:	Unknown	Sample Set Name:	
Vial:	1	Acq. Method Set:	HPLC
Injection #:	3	Processing Method:	Default
Injection Volume:	4.00 ul	Channel Name:	W2489 ChA
Run Time:	20.0 Minutes	Proc. Chnl. Descr.:	W2489 ChA 214nm
Date Acquired:	9/26/2019 12:25:52 AM CST		
Date Processed:	9/26/2019 12:46:22 AM CST		

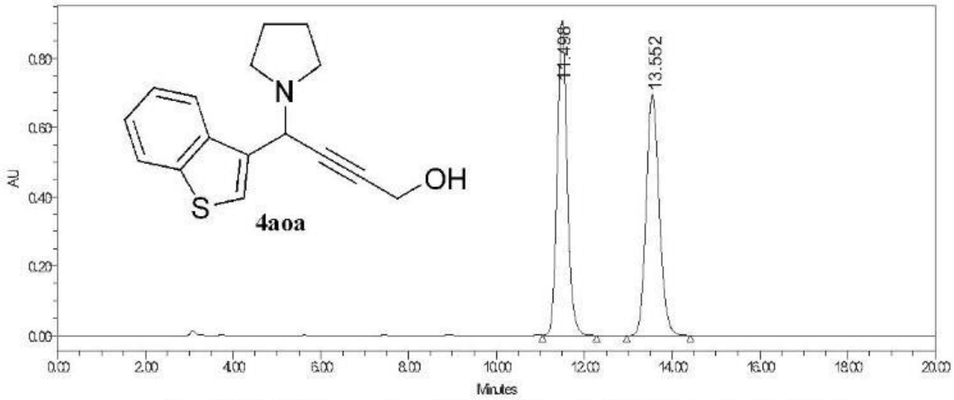

Channel: W2489 ChA; Processed Channel: W2489 ChA 214nm; Result Id: 5171; Processing Method: Default

Processed Channel Descr.: W2489 ChA 214nm

	Processed Channel Descr.	RT	Area	%Area	Height
1	W2489 ChA 214nm	11.498	14191865	49.84	906737
2	W2489 ChA 214nm	13.552	14280501	50.16	695500

Reported by User: System
Report Method: Injection Summary Report
Report Method ID: 1639 1639
Page: 1 of 1

Project Name: HPLC
Date Printed:
9/26/2019
12:46:37 AM PRC

HPLC spectrum for (±)-4aoa

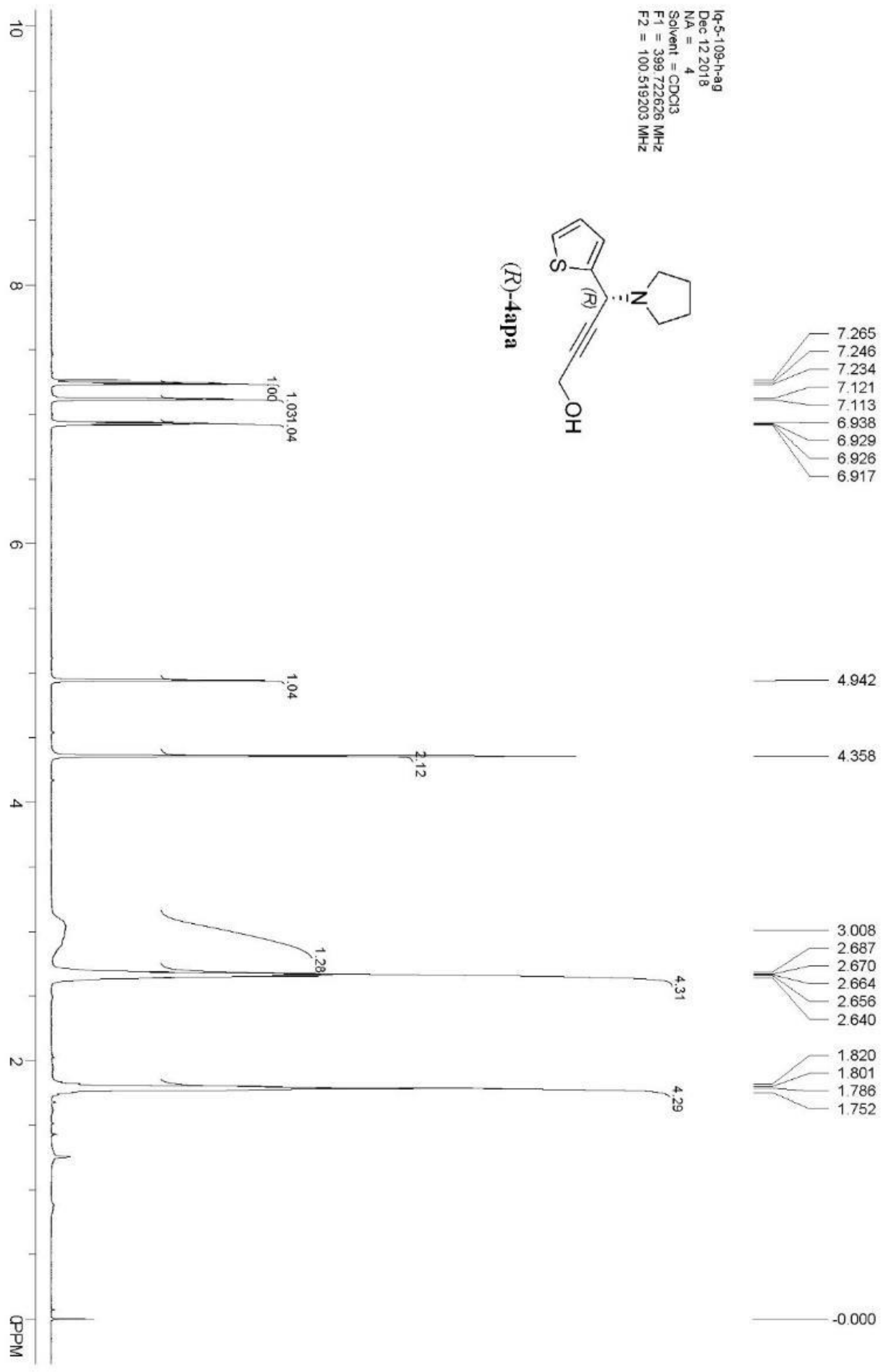

^{1}H NMR (400 MHz, CDCl3) spectrum for (*R*)**-4apa**

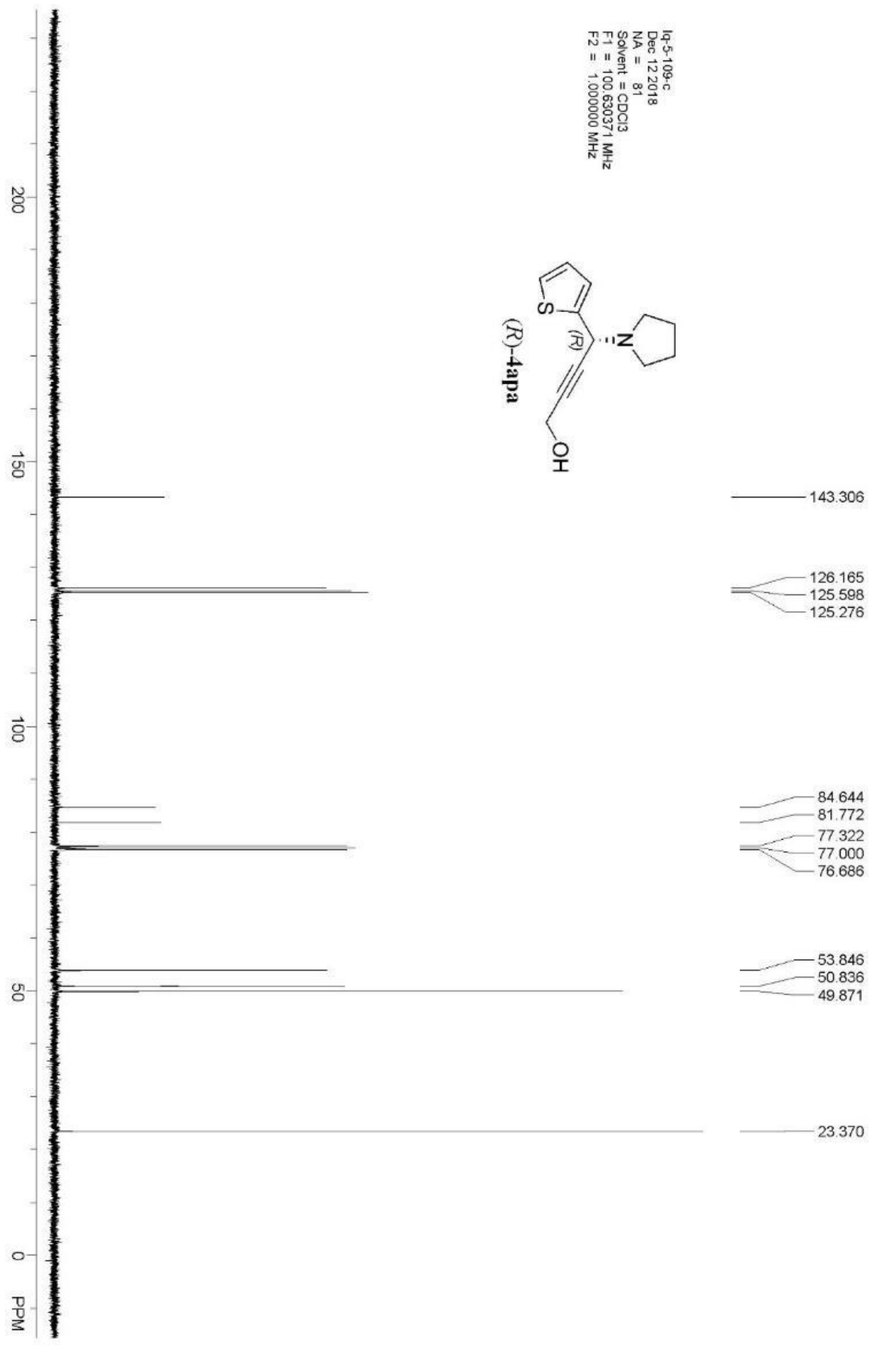

^{13}C NMR (400 MHz, $CDCl_3$) spectrum for (*R*)-4apa

Empower 3 SOFTWARE

Injection Summary Report

SAMPLE INFORMATION

Sample Name:	lq-5-109-ad-h-95-5-1.0-214	Acquired By:	System
Sample Type:	Unknown	Sample Set Name:	
Vial:	1	Acq. Method Set:	HPLC
Injection #:	1	Processing Method:	20181124
Injection Volume:	5.00 ul	Channel Name:	W2489 ChA
Run Time:	25.0 Minutes	Proc. Chnl. Descr.:	W2489 ChA 214nm
Date Acquired:	12/11/2018 2:30:58 PM CST		
Date Processed:	12/11/2018 2:55:27 PM CST		

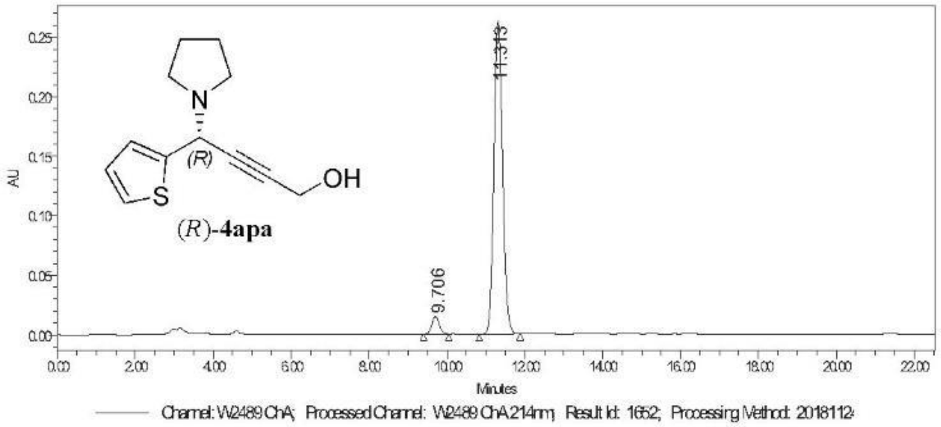

Processed Channel Descr.: W2489 ChA 214nm

	Processed Channel Descr.	RT	Area	%Area	Height
1	W2489 ChA 214nm	9.706	185888	4.40	14730
2	W2489 ChA 214nm	11.313	4038139	95.60	263018

Reported by User: System
Report Method: Injection Summary Report
Report Method ID: 1639 1639
Page: 1 of 1

Project Name: HPLC
Date Printed:
12/11/2018
2:55:46 PM PRC

HPLC spectrum for (*R*)-4apa

Injection Summary Report

SAMPLE INFORMATION

Sample Name:	xhb-1-122-ad-h-95-5-1.0-214	Acquired By:	System
Sample Type:	Unknown	Sample Set Name:	
Vial:	1	Acq. Method Set:	HPLC
Injection #:	2	Processing Method:	20181124
Injection Volume:	5.00 ul	Channel Name:	W2489 ChA
Run Time:	25.0 Minutes	Proc. Chnl. Descr.:	W2489 ChA 214nm
Date Acquired:	12/11/2018 2:57:26 PM CST		
Date Processed:	12/11/2018 3:23:02 PM CST		

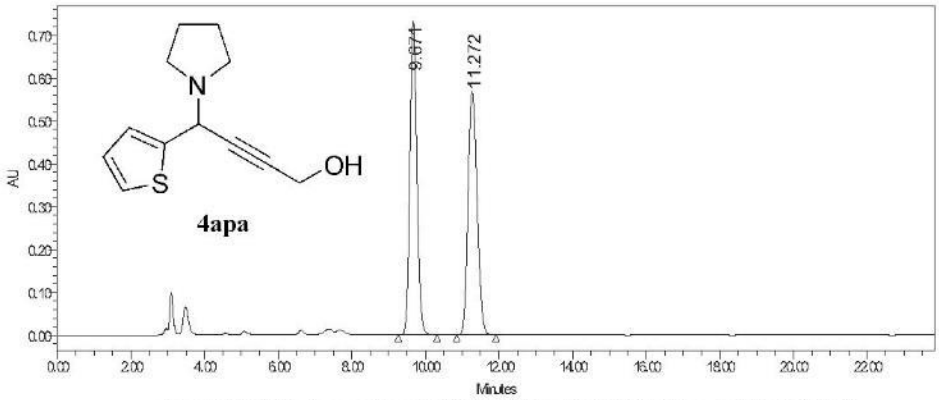

Channel: W2489 ChA; Processed Channel: W2489 ChA 214nm; Result Id: 1659; Processing Method: 2018112[illegible]

Processed Channel Descr.: W2489 ChA 214nm

	Processed Channel Descr.	RT	Area	%Area	Height
1	W2489 ChA 214nm	9.671	9644241	49.83	732102
2	W2489 ChA 214nm	11.272	9708940	50.17	567267

Reported by User: System
Report Method: Injection Summary Report
Report Method ID: 1639 1639
Page: 1 of 1

Project Name: HPLC
Date Printed:
12/11/2018
3:23:19 PM PRC

HPLC spectrum for (±)-4apa

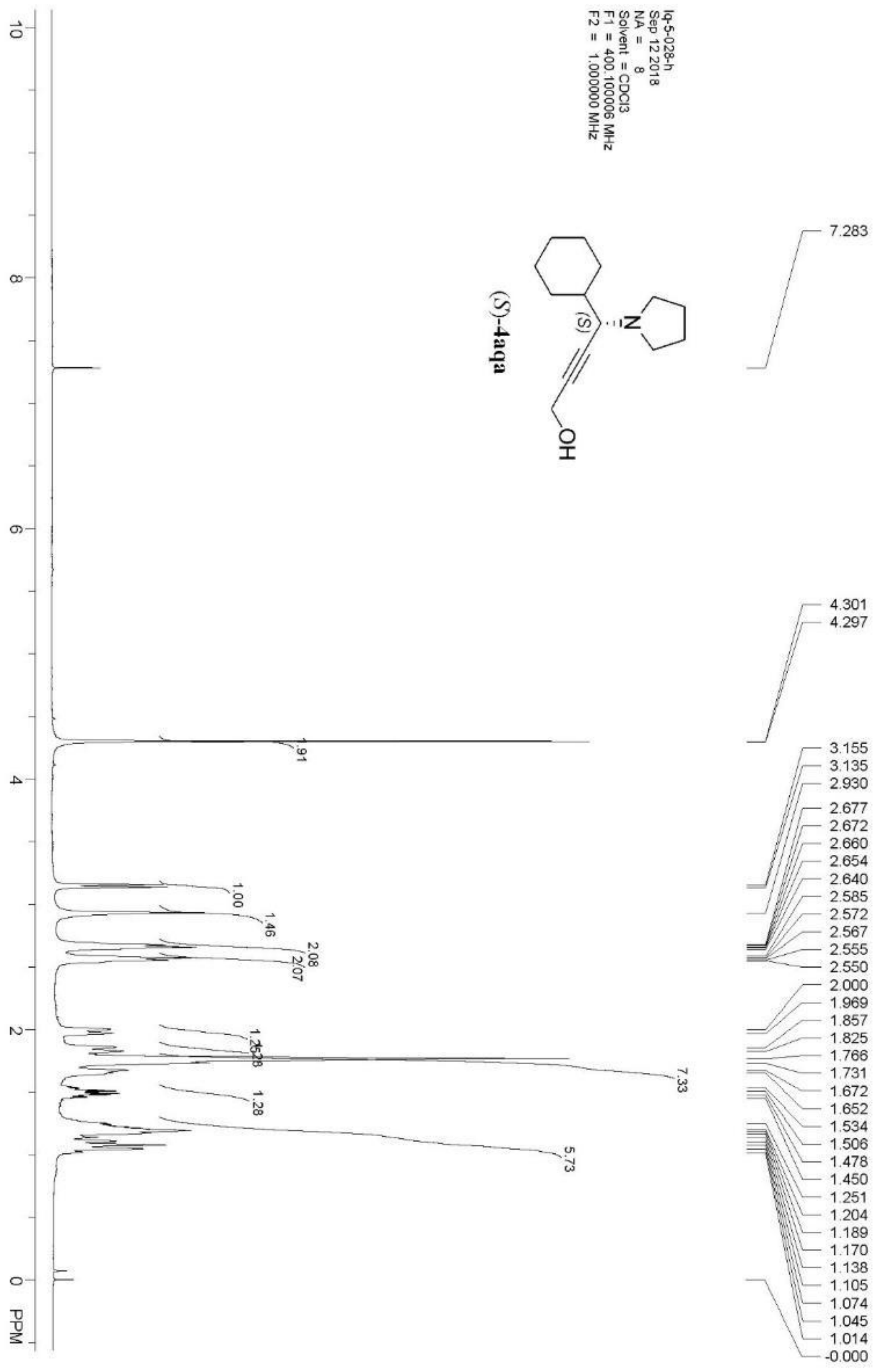

^{1}H NMR (400 MHz, CDCl3) spectrum for (*S*)**-4aqa**

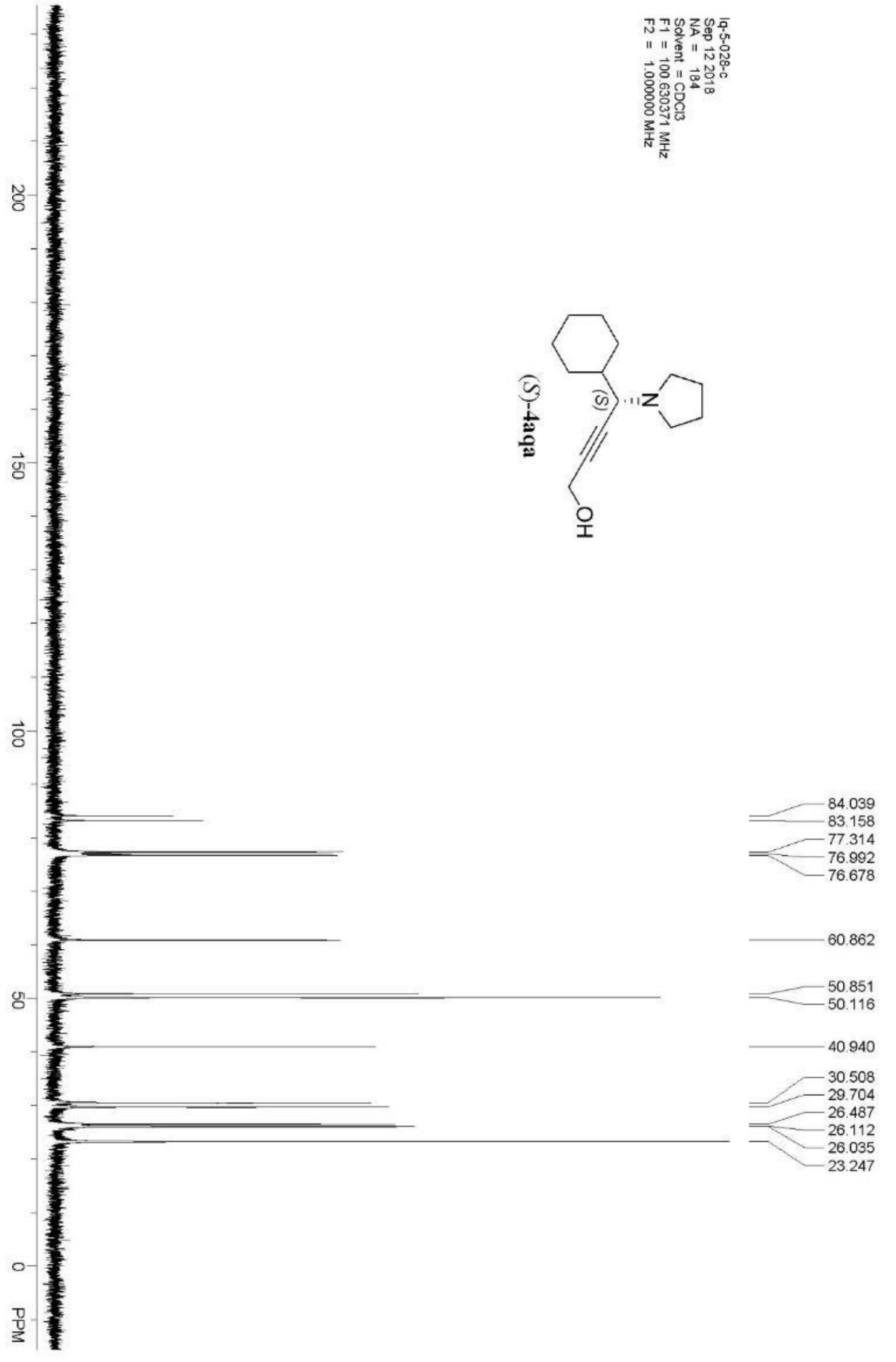

^{13}C NMR (400 MHz, CDCl$_3$) spectrum for (*S*)-4aqa

LQ-5-028

实验时间：2018-09-11,22:09:16　　　　　　　　　　　报告时间：2018-09-12,9:00:41
谱图文件:D:\data\slf\lq\2018-09-11-3\LQ-5-028-AD-H-90+10-0.7-214.org

实验内容简介：
AD-H　90:10
214nm　0.7ml/min

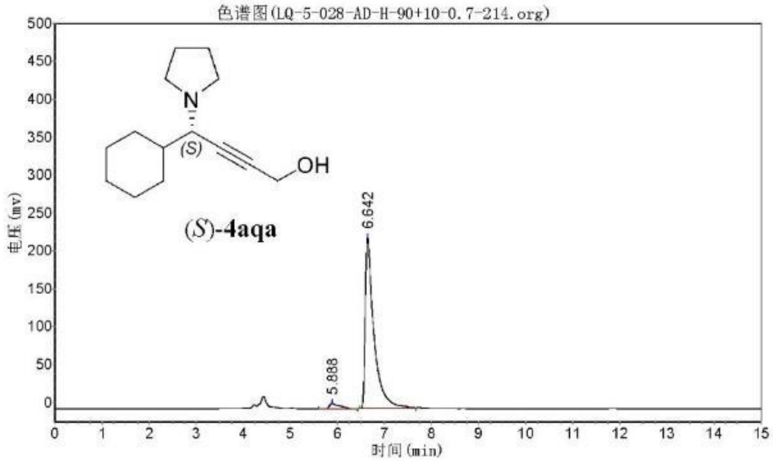

分析结果表

峰号	峰名	保留时间	峰高	峰面积	含量
1		5.888	7072.935	112194.016	3.7350
2		6.642	225317.313	2891694.750	96.2650
总计			232390.248	3003888.766	100.0000

HPLC spectrum for (*S*)-4aqa

lq-5-027

实验时间：2018-09-11,22:37:22　　　　　　　　　　报告时间：2018-09-12,9:06:07
谱图文件:D:\data\slf\lq\2018-09-11-3\LQ-5-027-AD-H-90+10-0.7-214-02.org

实验内容简介：
AD-H　90:10
214nm　0.7ml/min

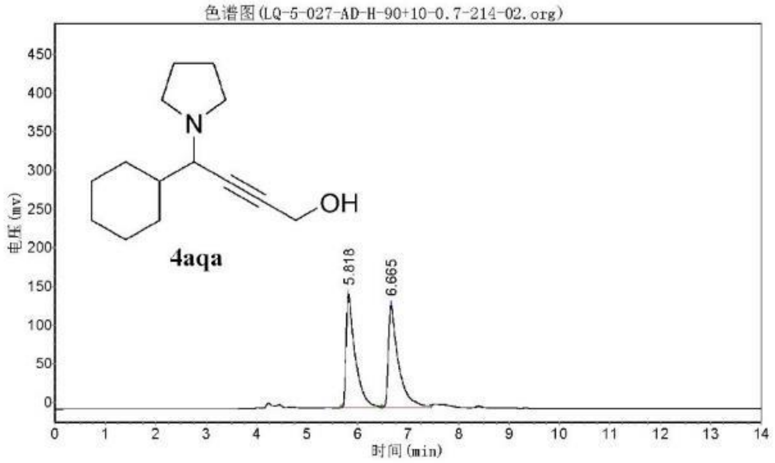

分析结果表

峰号	峰名	保留时间	峰高	峰面积	含量
1		5.818	147348.219	1835248.500	49.7079
2		6.665	132933.859	1856816.500	50.2921
总计			280282.078	3692065.000	100.0000

HPLC spectrum for (±)-4aqa

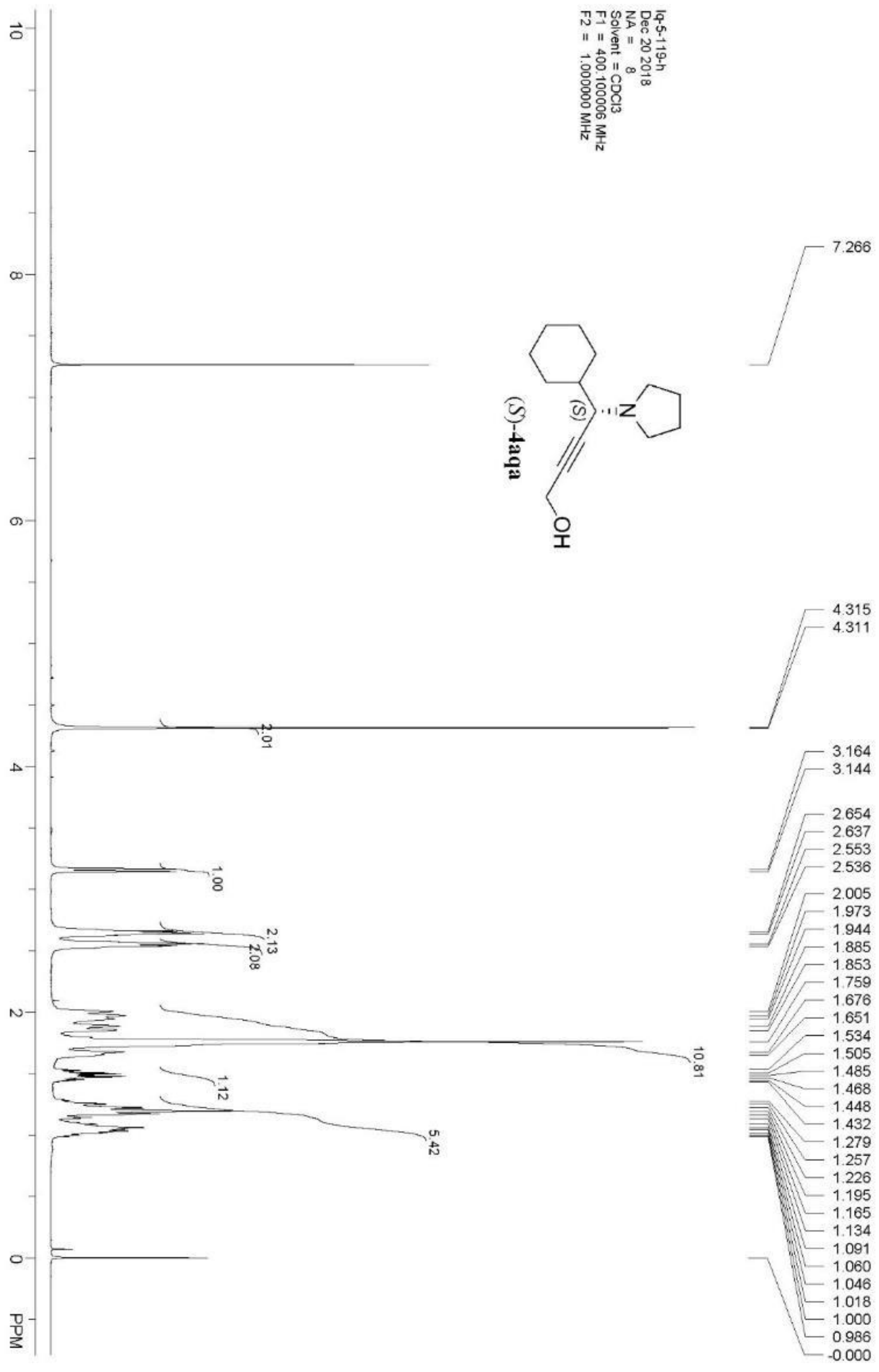

^{1}H NMR (400 MHz, CDCl3) spectrum for (*S*)**-4aqa**

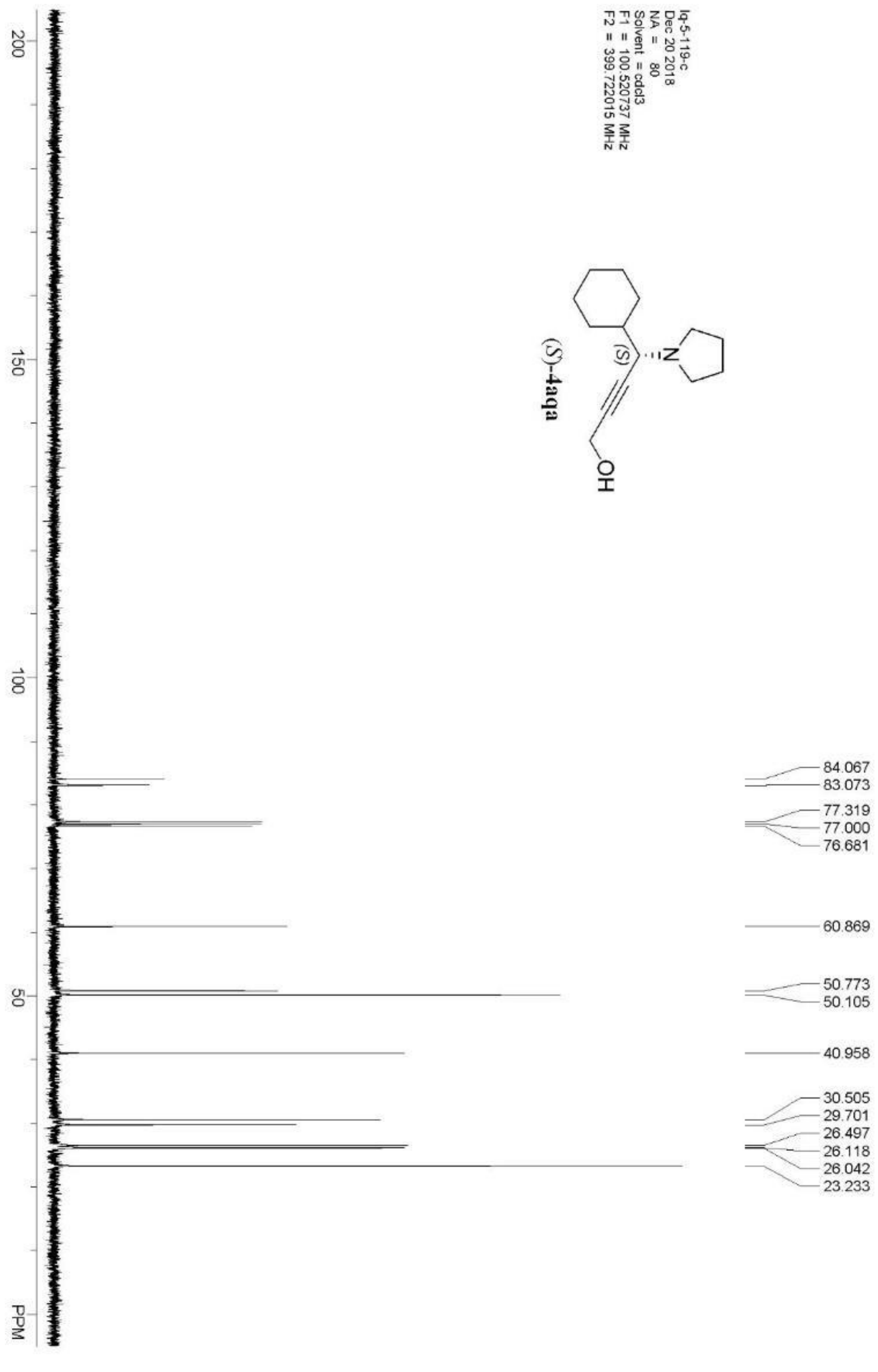

^{13}C NMR (400 MHz, $CDCl_3$) spectrum for (*S*)-4aqa

lq-5-119

实验时间：2018-12-21, 9:35:03　　　　　　　　　　　报告时间：2018-12-21, 10:26:16
谱图文件:D:\data\slf\lq\2018-12-21\lq-5-119-AD-H-95+5-0.8-214-2.org

实验内容简介：
AD-H　95:5
214nm　0.8ml/min

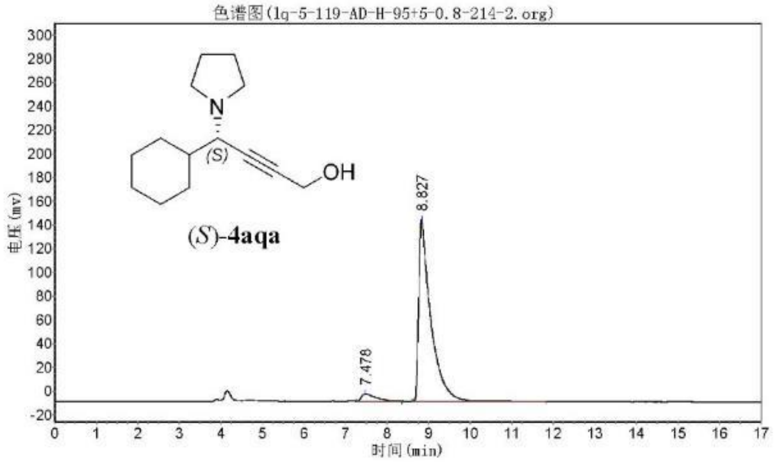

分析结果表

峰号	峰名	保留时间	峰高	峰面积	含量
1		7.478	6355.607	153683.906	4.7434
2		8.827	152875.000	3086284.500	95.2566
**总计**			159230.607	3239968.406	100.0000

HPLC spectrum for (*S*)-4aqa

xhb-1-125

实验时间：2018-12-21, 10:06:17　　　　报告时间：2018-12-21, 10:28:05
谱图文件:D:\data\slf\lq\2018-12-21\XHB-1-125-AD-H-95+5-0.8-214-2.org

实验内容简介：
AD-H　95:5
214nm　0.8ml/min

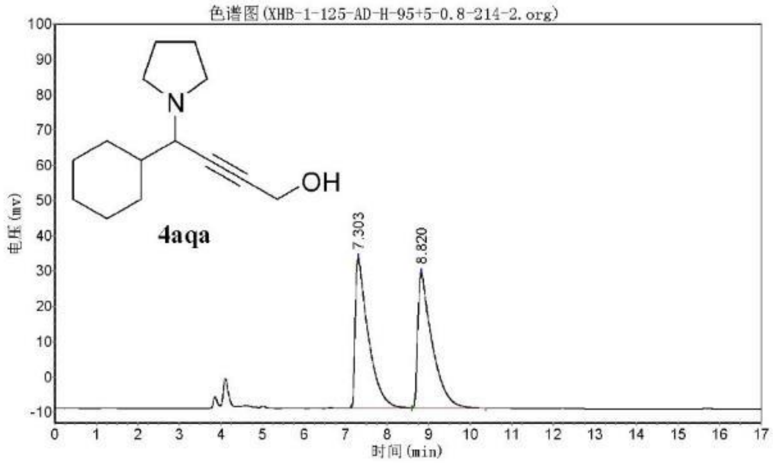

分析结果表

峰号	峰名	保留时间	峰高	峰面积	含量
1		7.303	42533.223	920737.250	50.1956
2		8.820	38326.930	913560.563	49.8044
总计			80860.152	1834297.813	100.0000

HPLC spectrum for (±)-4aqa

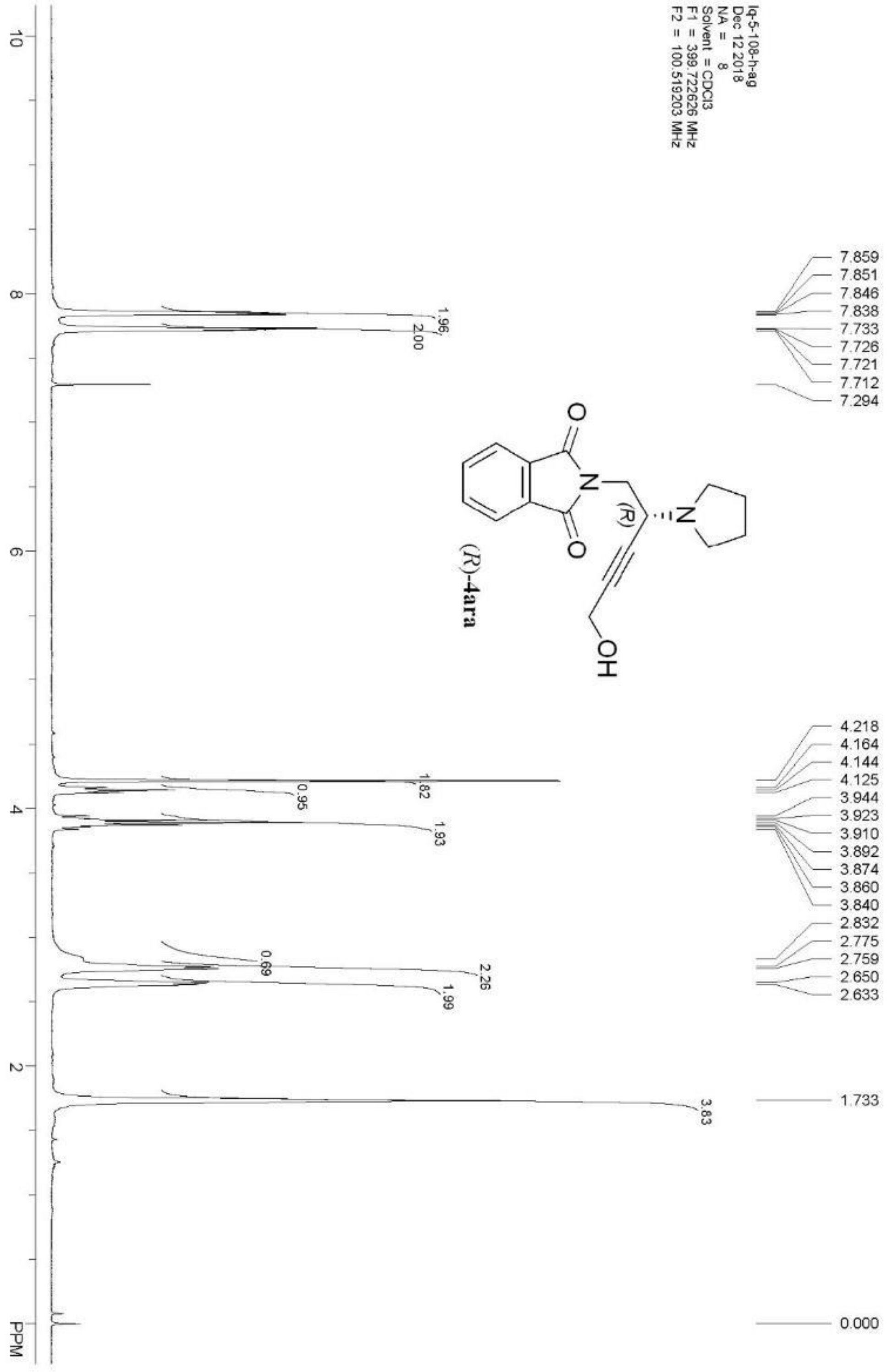

^{1}H NMR (400 MHz, CDCl3) spectrum for (*R*)**-4ara**

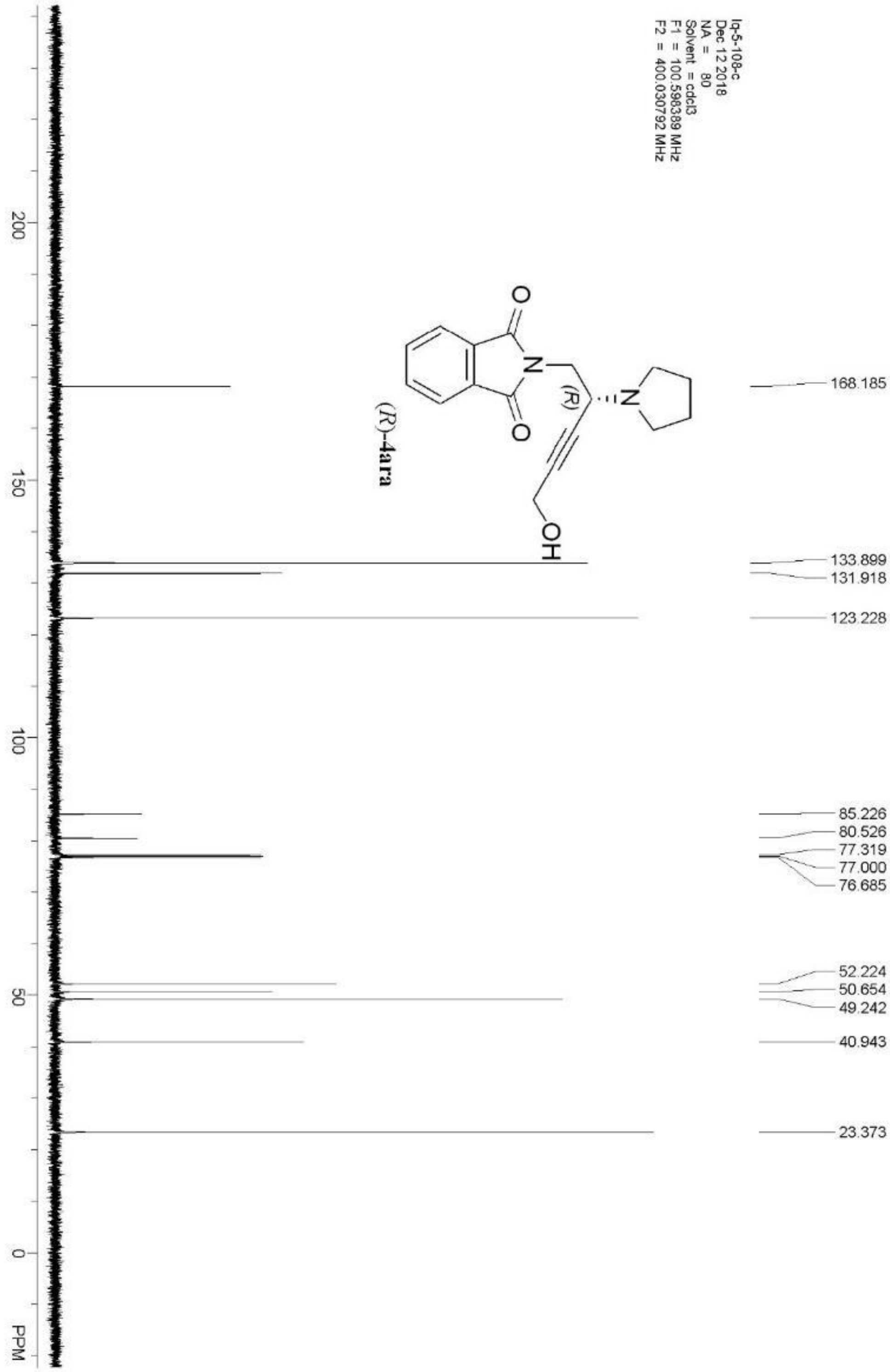

^{13}C NMR (400 MHz, CDCl$_3$) spectrum for (*R*)-4ara

Injection Summary Report

SAMPLE INFORMATION

Sample Name:	lq-5-108-pc-2-70-30-1.0-214	Acquired By:	System
Sample Type:	Unknown	Sample Set Name:	
Vial:	1	Acq. Method Set:	HPLC
Injection #:	2	Processing Method:	20181124
Injection Volume:	2.50 ul	Channel Name:	W2489 ChA
Run Time:	45.0 Minutes	Proc. Chnl. Descr.:	W2489 ChA 214nm
Date Acquired:	12/11/2018 4:15:03 AM CST		
Date Processed:	12/11/2018 5:07:43 AM CST		

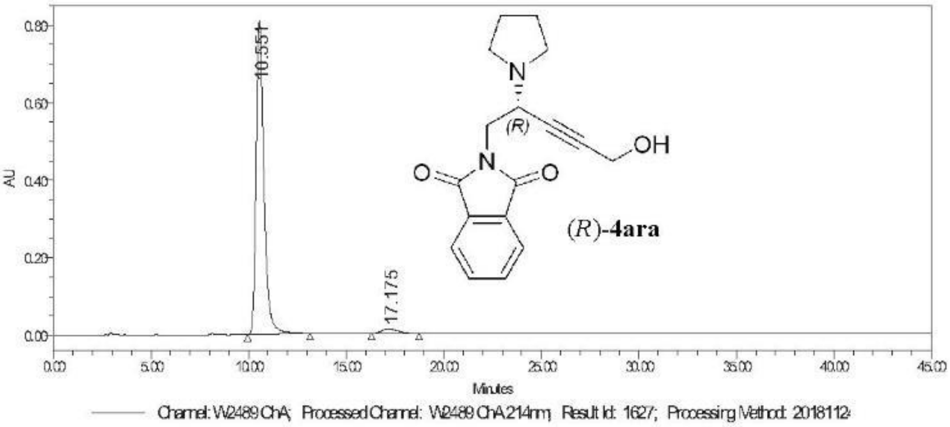

Processed Channel Descr.: W2489 ChA 214nm

	Processed Channel Descr.	RT	Area	%Area	Height
1	W2489 ChA 214nm	10.551	22659772	97.05	808914
2	W2489 ChA 214nm	17.175	688413	2.95	12819

Reported by User: System
Report Method: Injection Summary Report
Report Method ID: 1374 1374
Page: 1 of 1

Project Name: HPLC
Date Printed:
12/11/2018
5:08:03 AM PRC

HPLC spectrum for (*R*)-4ara

Empower 3 SOFTWARE

Injection Summary Report

SAMPLE INFORMATION

Sample Name:	xhb-1-115-po-2-70-30-1.0-214	Acquired By:	System
Sample Type:	Unknown	Sample Set Name:	
Vial:	1	Acq. Method Set:	HPLC
Injection #:	3	Processing Method:	20181124
Injection Volume:	3.00 ul	Channel Name:	W2489 ChA
Run Time:	45.0 Minutes	Proc. Chnl. Descr.:	W2489 ChA 214nm
Date Acquired:	12/11/2018 5:12:06 AM CST		
Date Processed:	12/11/2018 5:46:02 AM CST		

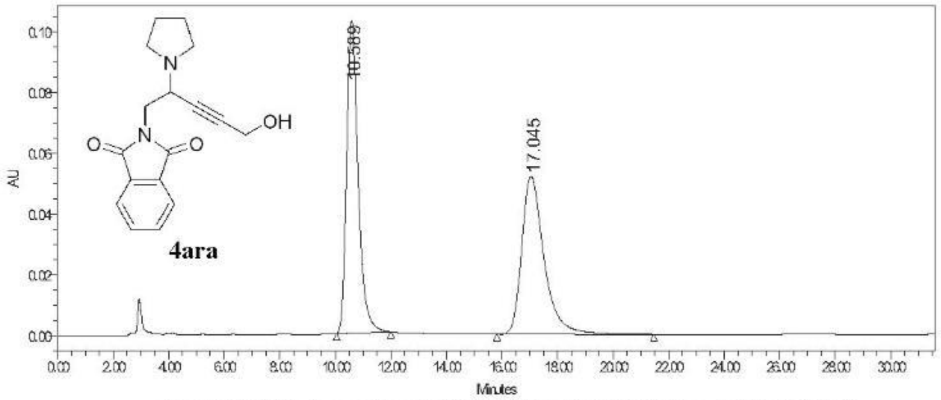

Channel: W2489 ChA; Processed Channel: W2489 ChA 214nm; Result Id: 1631; Processing Method: 2018112

Processed Channel Descr.: W2489 ChA 214nm

	Processed Channel Descr.	RT	Area	%Area	Height
1	W2489 ChA 214nm	10.589	2943283	50.49	102677
2	W2489 ChA 214nm	17.045	2886539	49.51	51991

Reported by User: System
Report Method: Injection Summary Report
Report Method ID: 1374 1374
Page: 1 of 1

Project Name: HPLC
Date Printed:
12/11/2018
5:46:27 AM PRC

HPLC spectrum for (±)-4ara

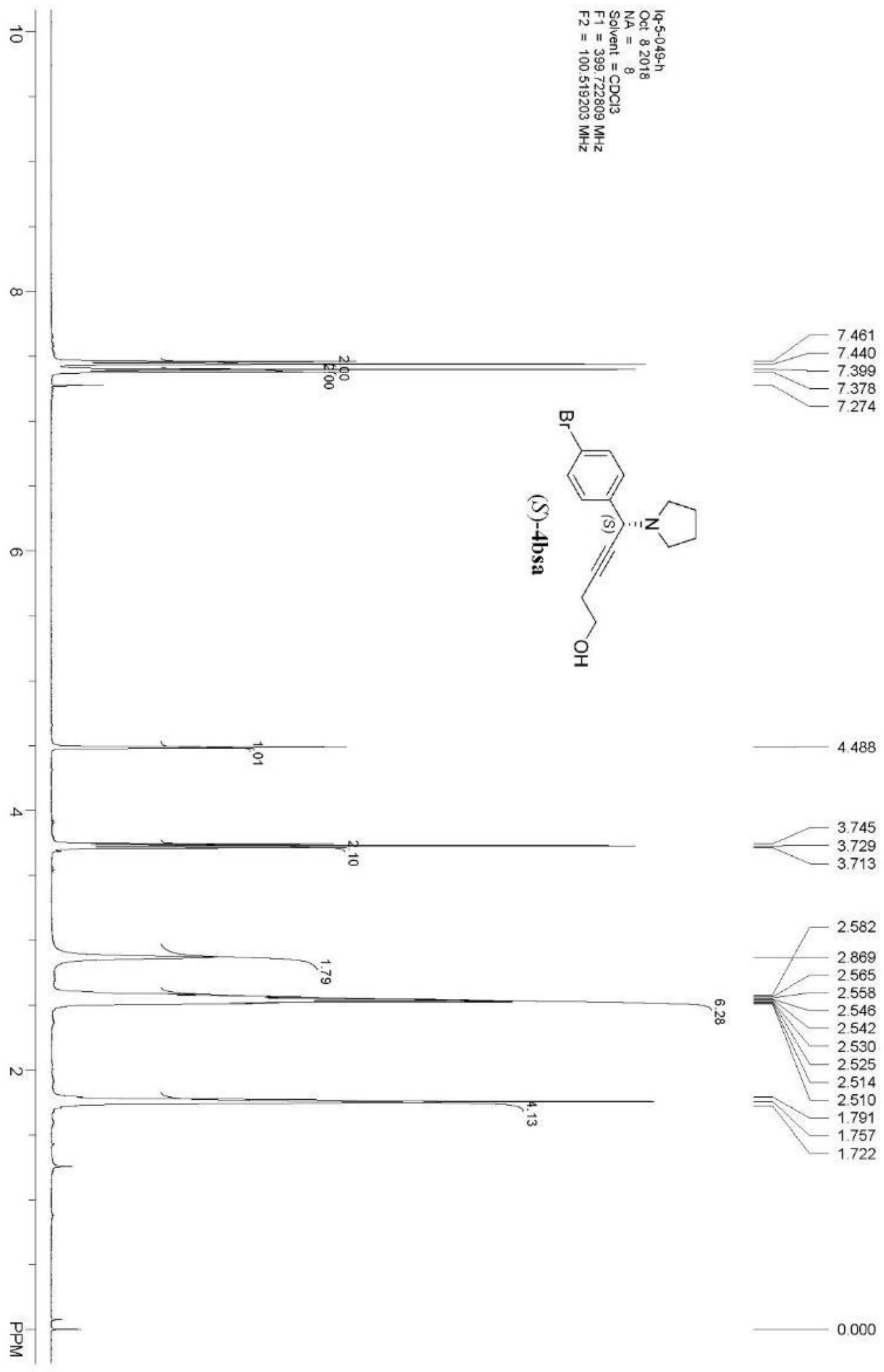

^{1}H NMR (400 MHz, CDCl3) spectrum for (*S*)-4bsa

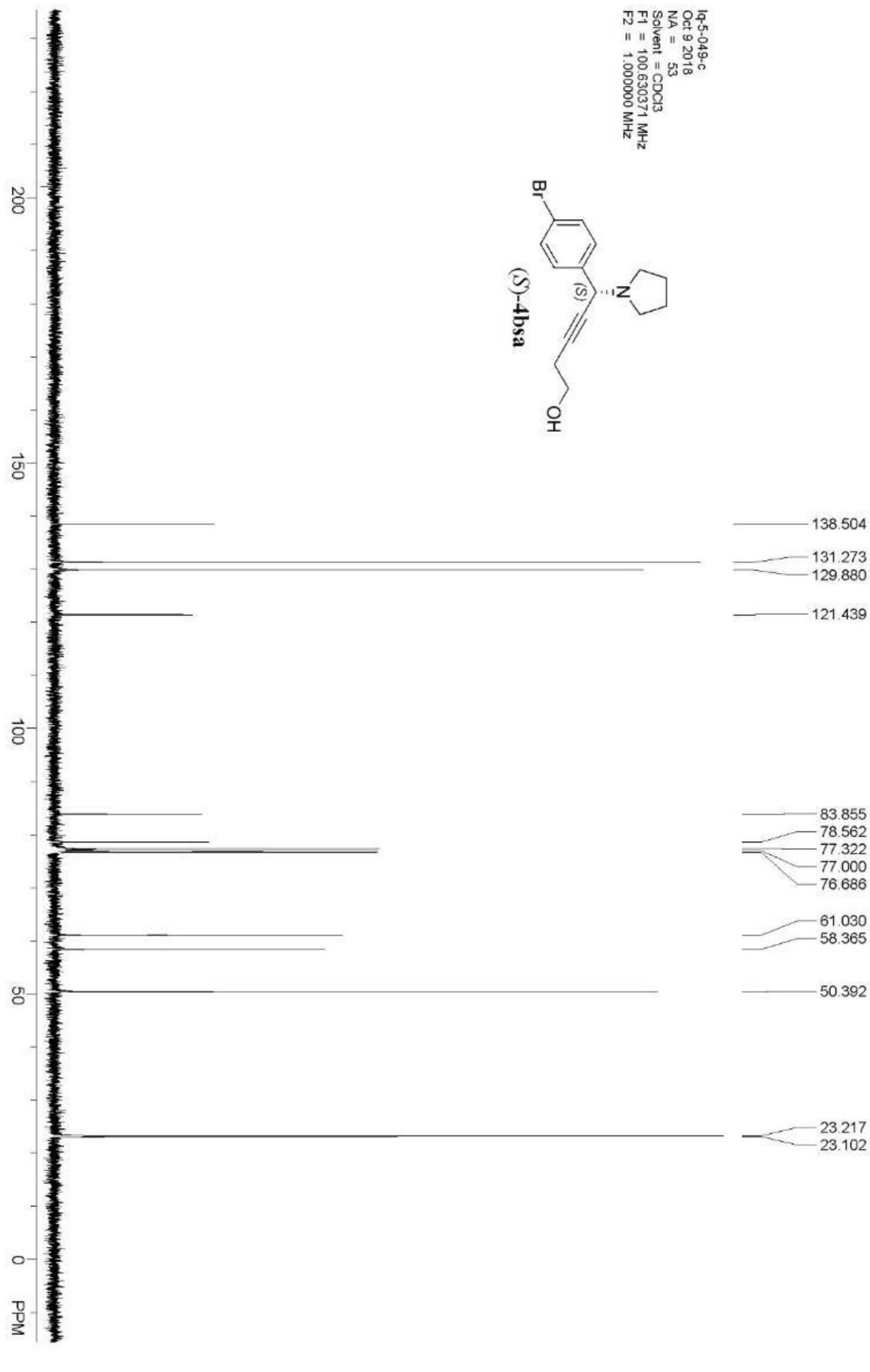

^{13}C NMR (400 MHz, $CDCl_3$) spectrum for (*S*)-4bsa

1q-5-049

实验时间：2018-10-08, 12:11:58　　　　　　　　报告时间：2018-10-09, 9:18:16
谱图文件:D:\data\slf\lq\2018-10-08\lq-5-049-ad-h-98-2-1-214.org

实验内容简介：
AD-H　98:2
214nm　1.0ml/min

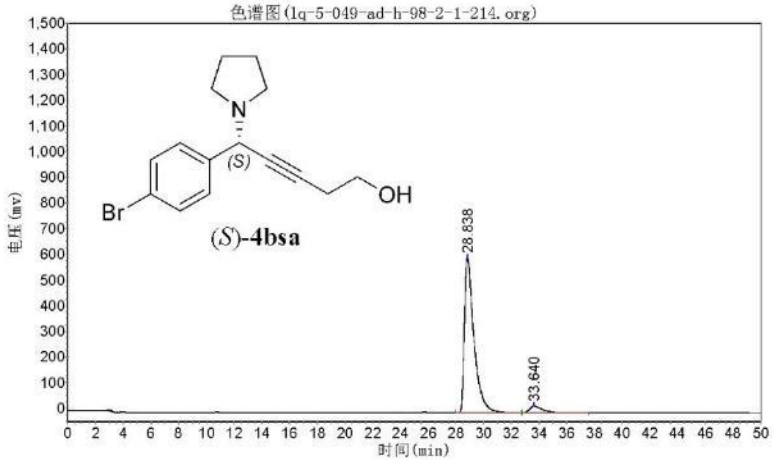

分析结果表

峰号	峰名	保留时间	峰高	峰面积	含量
1		28.838	603195.188	28861330.000	94.9081
2		33.640	25615.701	1548442.250	5.0919
总计			628810.889	30409772.250	100.0000

HPLC spectrum for (*S*)-4bsa

xhb-1-050

实验时间：2018-10-08, 11:05:41　　　　报告时间：2018-10-09, 9:20:16
谱图文件:D:\data\slf\lq\2018-10-08\xhb-1-050-ad-h-98-2-1-214.org

实验内容简介：
AD-H　98:2
214nm　1.0ml/min

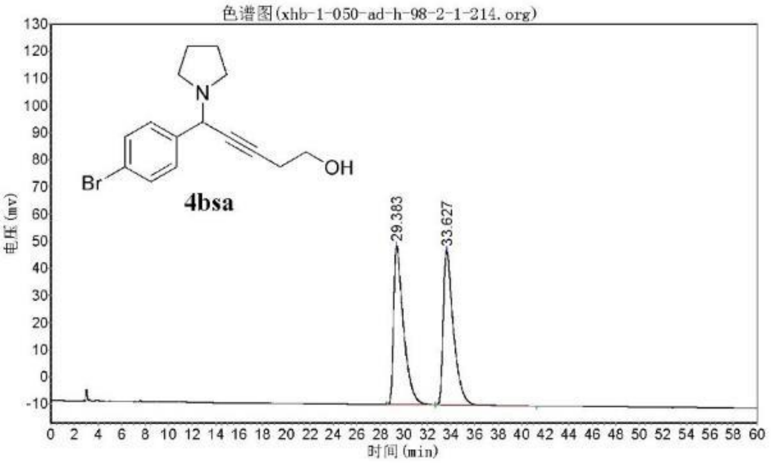

分析结果表

峰号	峰名	保留时间	峰高	峰面积	含量
1		29.383	58455.824	3410440.750	49.8591
2		33.627	57031.363	3429718.000	50.1409
总计			115487.188	6840158.750	100.0000

HPLC spectrum for (±)-4bsa

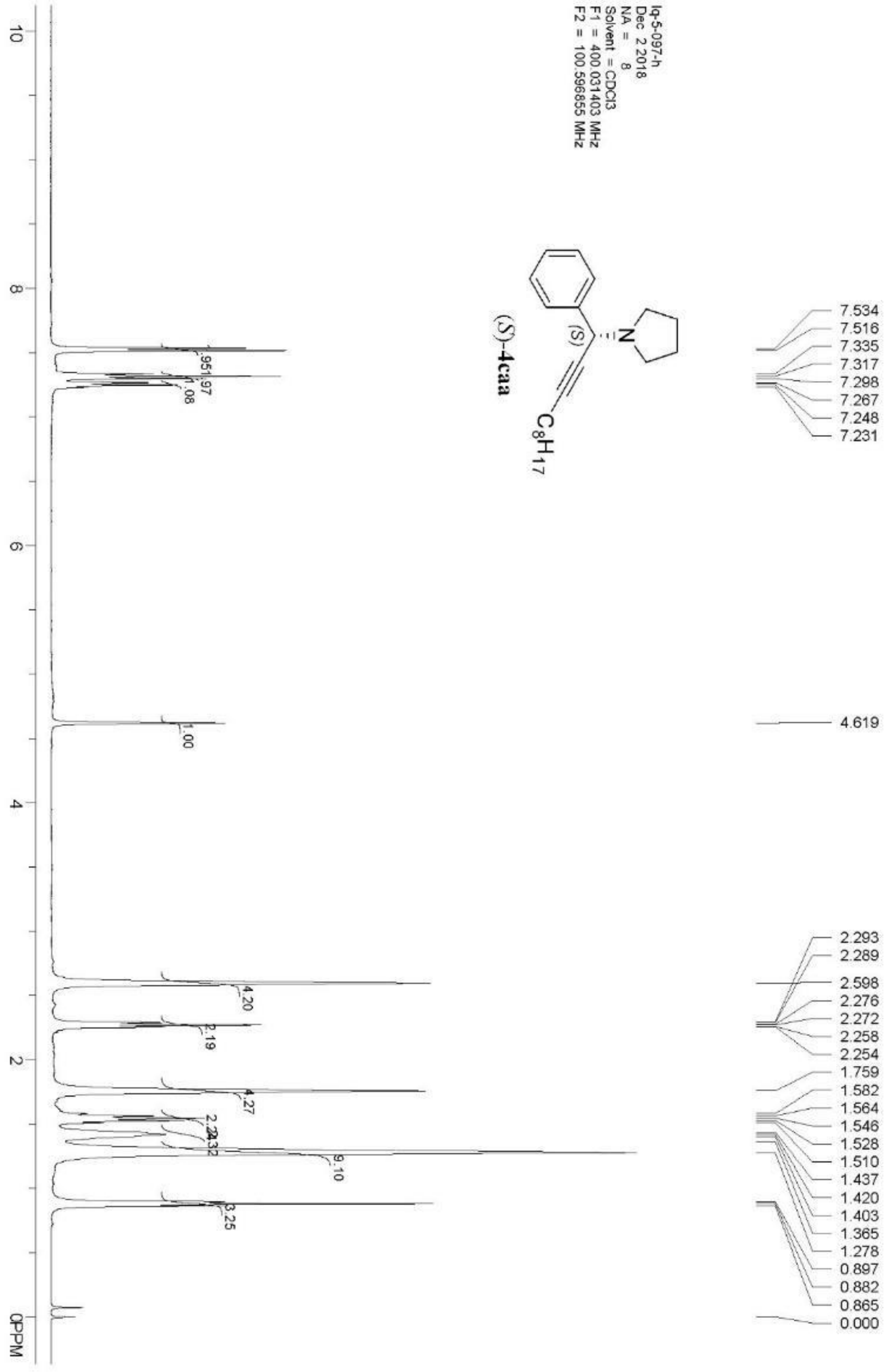

^{1}H NMR (400 MHz, CDCl3) spectrum for (*S*)-4caa

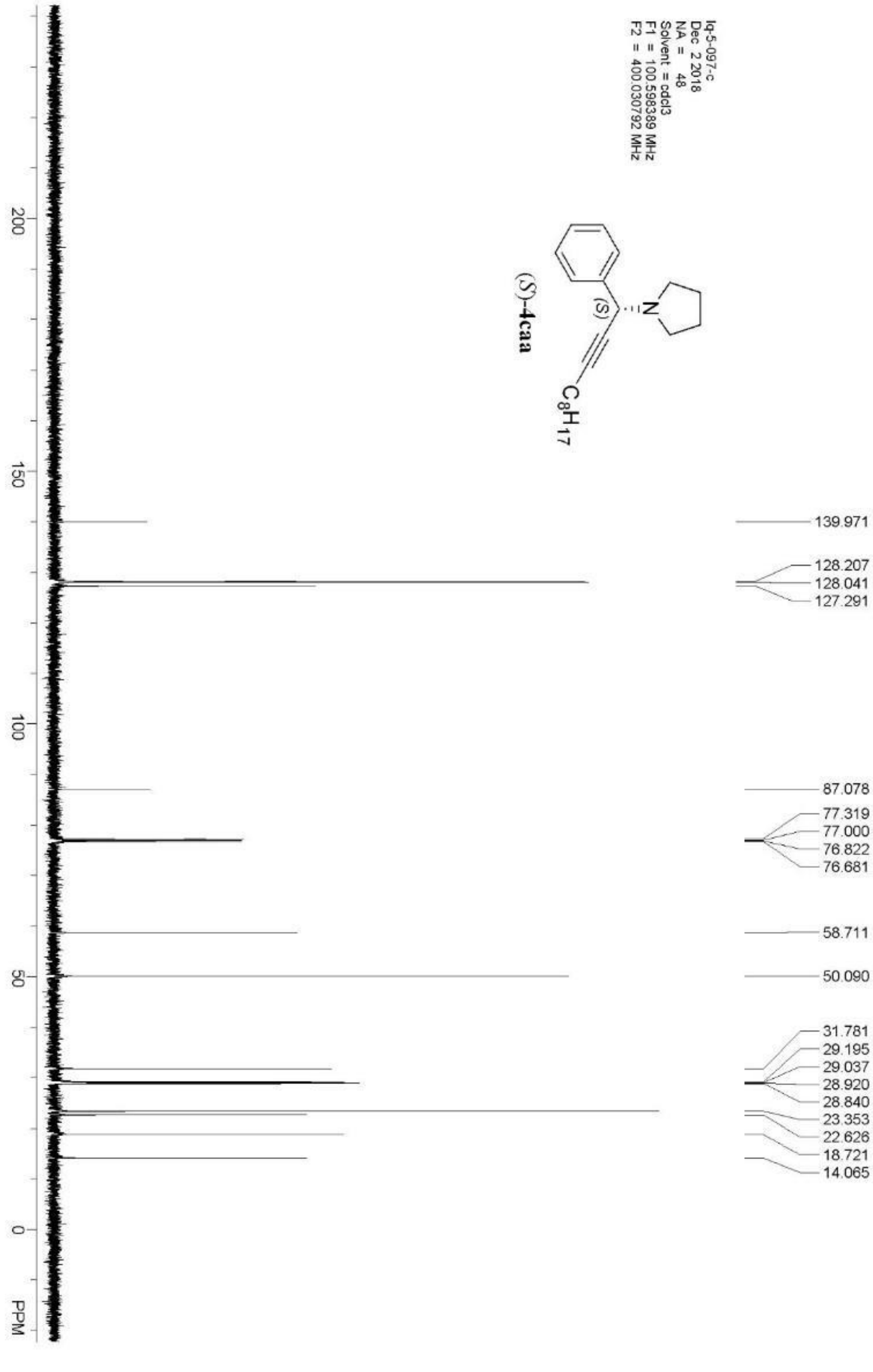

^{13}C NMR (400 MHz, CDCl$_3$) spectrum for (*S*)-4caa

Empower 3 SOFTWARE

Injection Summary Report

SAMPLE INFORMATION

Sample Name:	lq-5-097-cj-h-100-0-0.5-214	Acquired By:	System
Sample Type:	Unknown	Sample Set Name:	
Vial:	1	Acq. Method Set:	HPLC
Injection #:	1	Processing Method:	20181124
Injection Volume:	5.00 ul	Channel Name:	W2489 ChA
Run Time:	30.0 Minutes	Proc. Chnl. Descr.:	W2489 ChA 214nm
Date Acquired:	12/2/2018 8:31:33 AM CST		
Date Processed:	12/4/2018 12:23:55 AM CST		

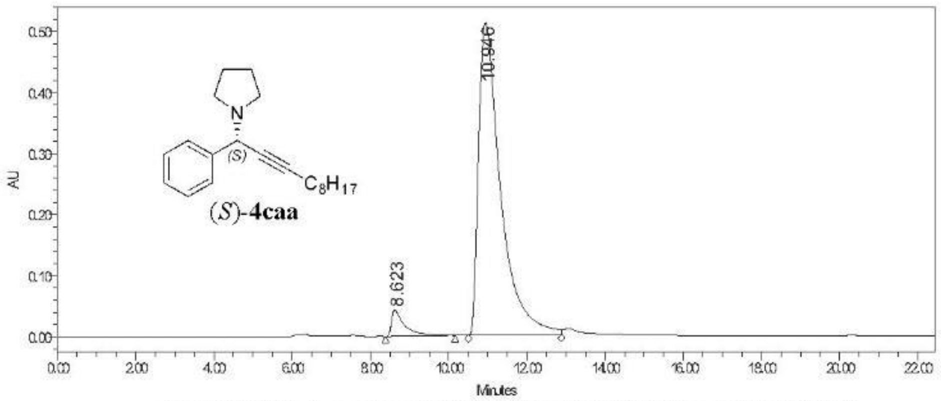

Processed Channel Descr.: W2489 ChA 214nm

	Processed Channel Descr.	RT	Area	%Area	Height
1	W2489 ChA 214nm	8.623	994615	4.73	43665
2	W2489 ChA 214nm	10.946	20014740	95.27	512385

Reported by User: System
Report Method: Injection Summary Report
Report Method ID: 1374 1374
Page: 1 of 1

Project Name: HPLC
Date Printed:
12/4/2018
12:28:50 AM PRC

HPLC spectrum for (*S*)-4caa

Empower 3 SOFTWARE

Injection Summary Report

SAMPLE INFORMATION

Sample Name:	xhb-1-111-oj-h-100-0-0.5-214	Acquired By:	System
Sample Type:	Unknown	Sample Set Name:	
Vial:	1	Acq. Method Set:	HPLC
Injection #:	1	Processing Method:	20181124
Injection Volume:	5.00 ul	Channel Name:	W2489 ChA
Run Time:	30.0 Minutes	Proc. Chnl. Descr.:	W2489 ChA 214nm
Date Acquired:	12/2/2018 7:34:17 AM CST		
Date Processed:	12/2/2018 7:58:54 AM CST		

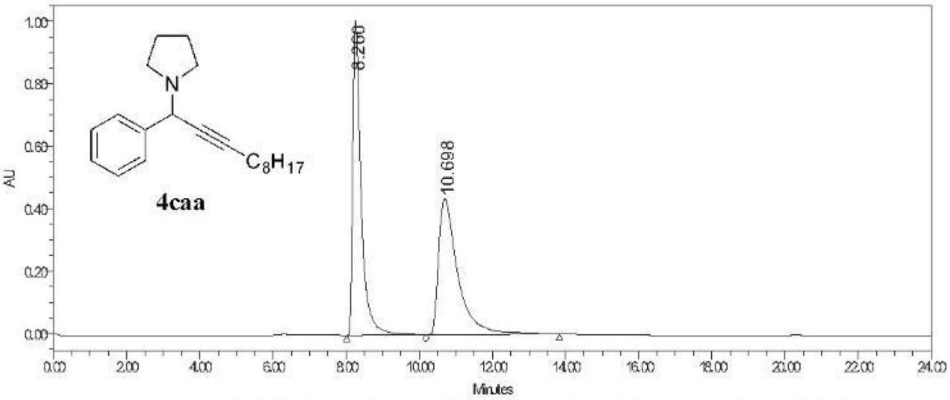

Processed Channel Descr.: W2489 ChA 214nm

	Processed Channel Descr.	RT	Area	%Area	Height
1	W2489 ChA 214nm	8.260	15949270	50.20	1007930
2	W2489 ChA 214nm	10.698	15824812	49.80	434443

Reported by User: System
Report Method: Injection Summary Report
Report Method ID: 1007 1007
Page: 1 of 1

Project Name: HPLC
Date Printed:
12/2/2018
8:01:01 AM PRC

HPLC spectrum for (±)-4caa

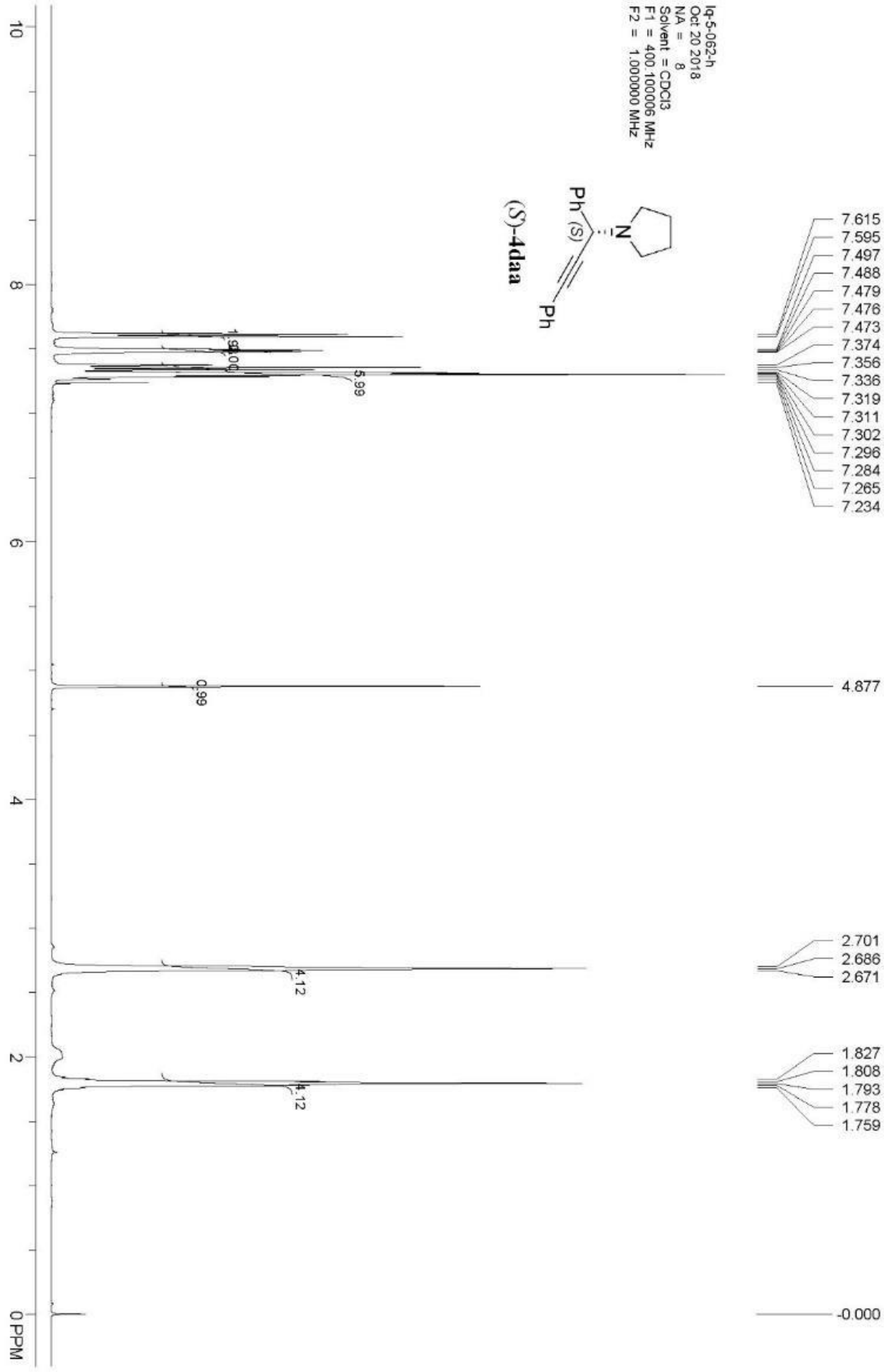

^{1}H NMR (400 MHz, CDCl3) spectrum for (*S*)-4daa

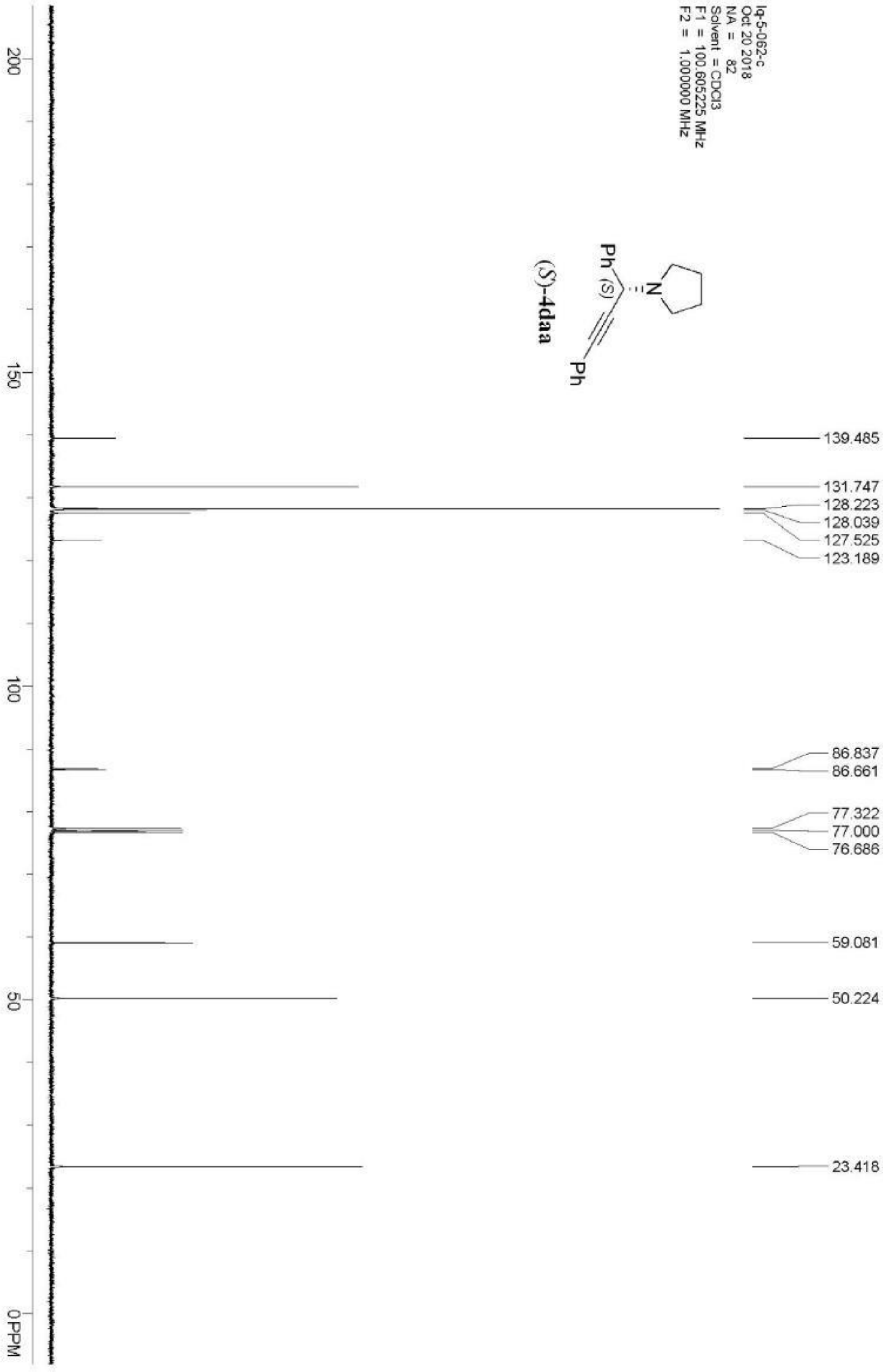

^{13}C NMR (400 MHz, CDCl$_3$) spectrum for (*S*)-4daa

1q-5-062

实验时间：2018-10-19, 13:05:48　　　　报告时间：2018-10-19, 13:25:03
谱图文件:D:\data\slf\lq\2018-10-19\lq-5-062-oj-h-99+1-1-214-2.org

实验内容简介：
OJ-H　99:1
214nm　1.0ml/min

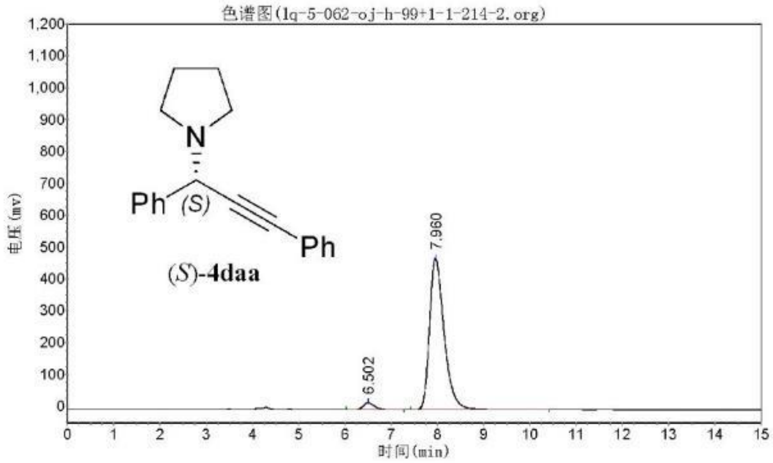

分析结果表

峰号	峰名	保留时间	峰高	峰面积	含量
1		6.502	21398.996	350065.781	3.2610
2		7.960	474499.563	10384928.000	96.7390
**总计**			495898.559	10734993.781	100.0000

HPLC spectrum for (*S*)-4daa

xhb-1-072

实验时间：2018-10-19, 12:06:38　　　　报告时间：2018-10-19, 12:31:03
谱图文件:D:\data\slf\lq\2018-10-19\xhb-1-072-oj-h-99+1-1-214-2.org

实验内容简介：
OJ-H　99:1
214nm　1.0ml/min

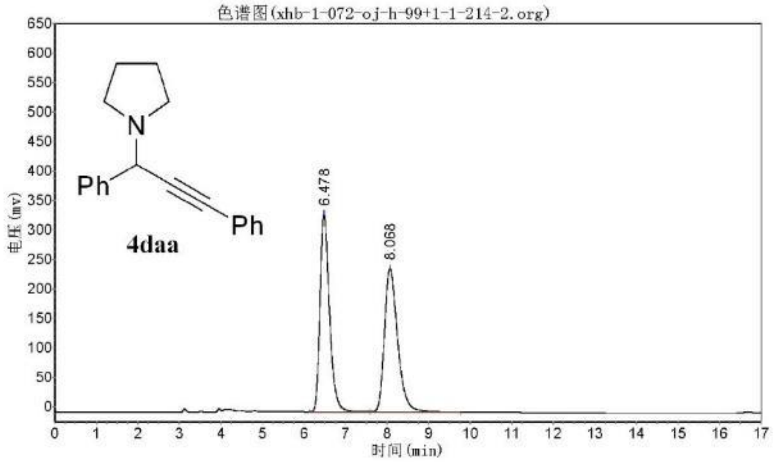

分析结果表

峰号	峰名	保留时间	峰高	峰面积	含量
1		6.478	335306.500	5337256.000	49.6938
2		8.068	243592.359	5403019.000	50.3062
**总计**			578898.859	10740275.000	100.0000

HPLC spectrum for (±)-4daa

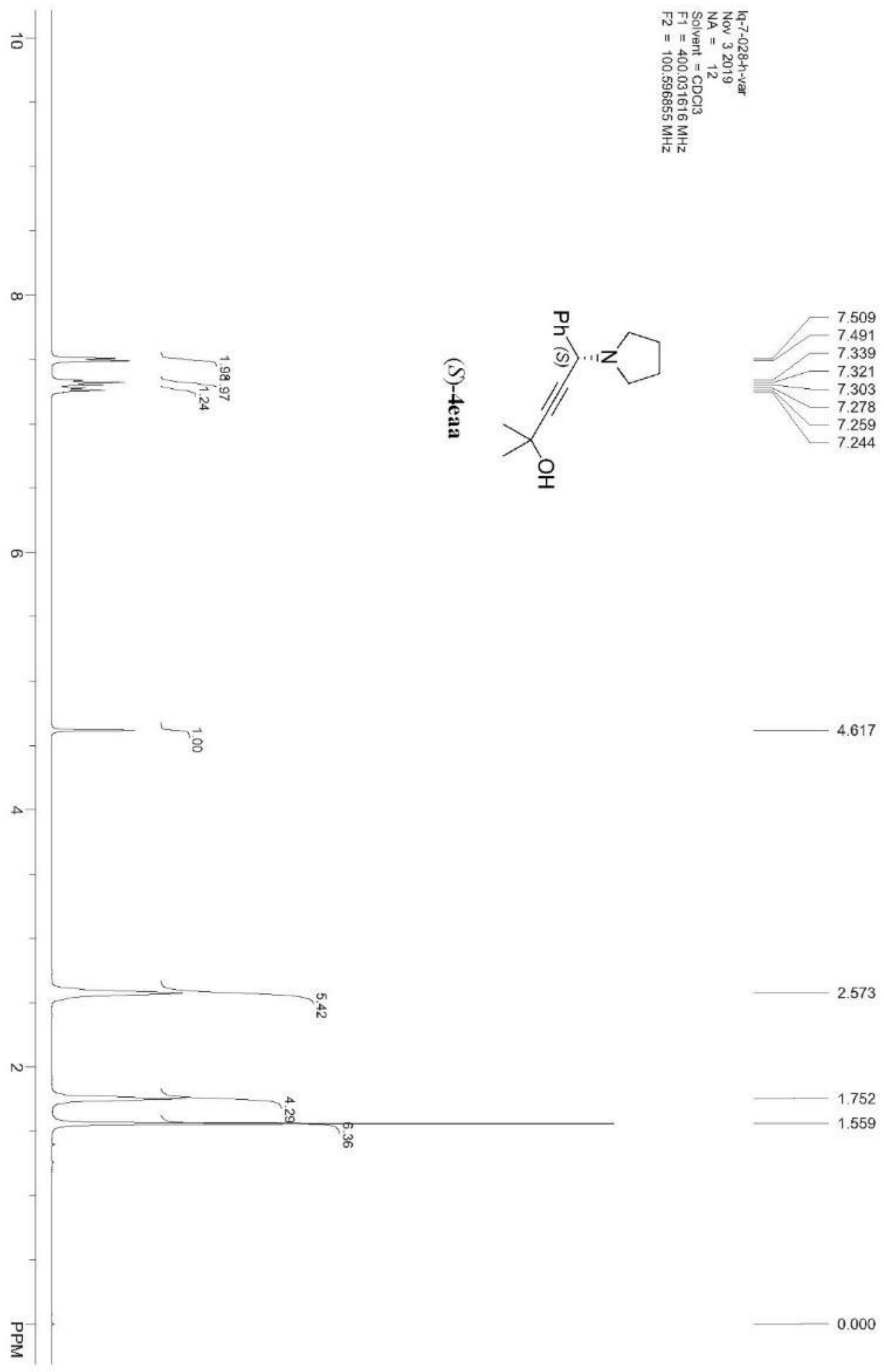

^{1}H NMR (400 MHz, CDCl3) spectrum for (*S*)**-4eaa**

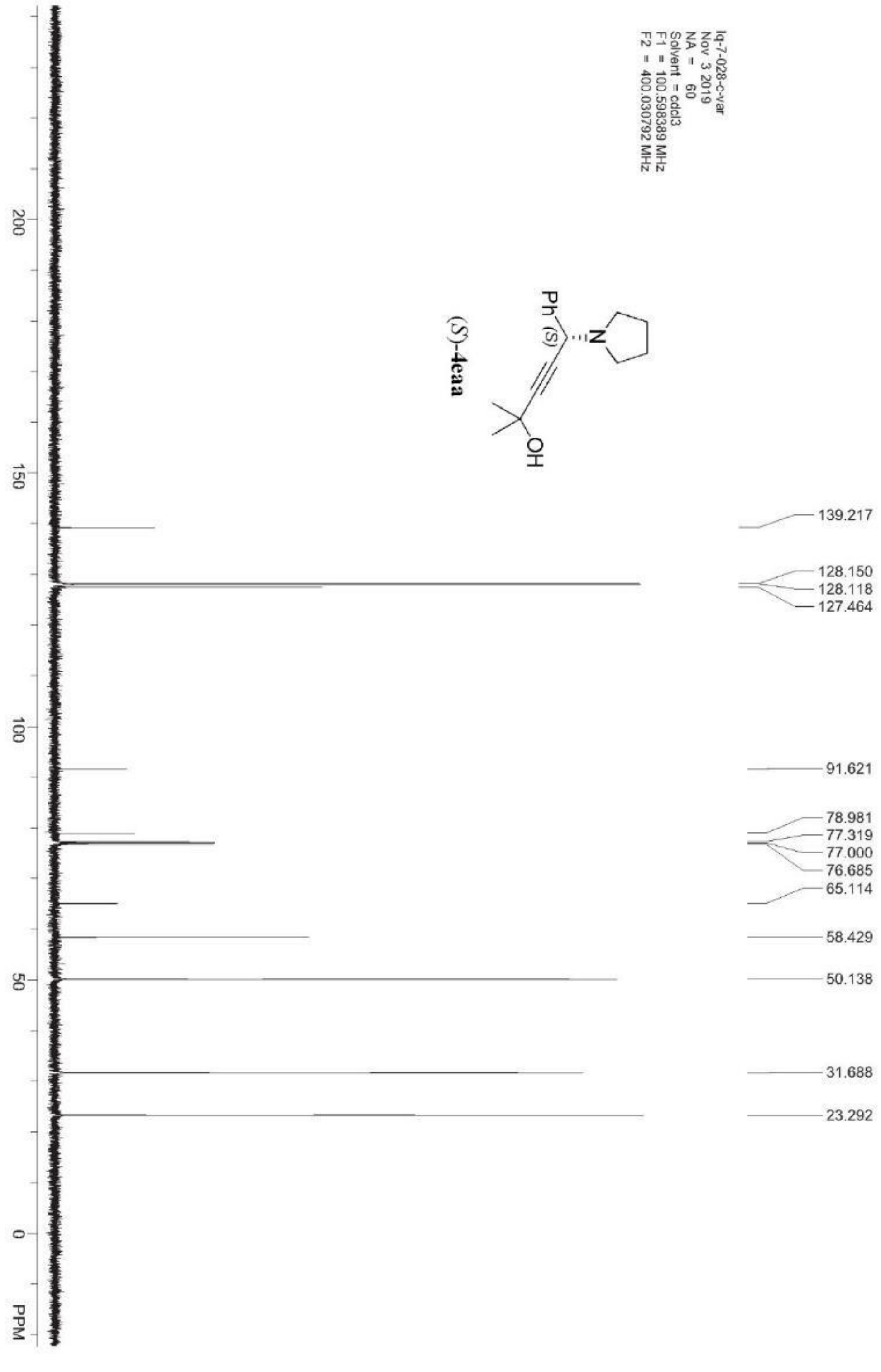

^{13}C NMR (400 MHz, CDCl$_3$) spectrum for (*S*)-4eaa

Empower™ 3 SOFTWARE

Injection Summary Report

SAMPLE INFORMATION

Sample Name:	lq-7-028-od-h-99-1-1-214	Acquired By:	System
Sample Type:	Unknown	Sample Set Name:	
Vial:	1	Acq. Method Set:	HPLC
Injection #:	1	Processing Method:	Default
Injection Volume:	5.00 ul	Channel Name:	W2489 ChA
Run Time:	30.0 Minutes	Proc. Chnl. Descr.:	W2489 ChA 214nm
Date Acquired:	11/7/2019 12:29:23 AM CST		
Date Processed:	11/7/2019 6:43:16 AM CST		

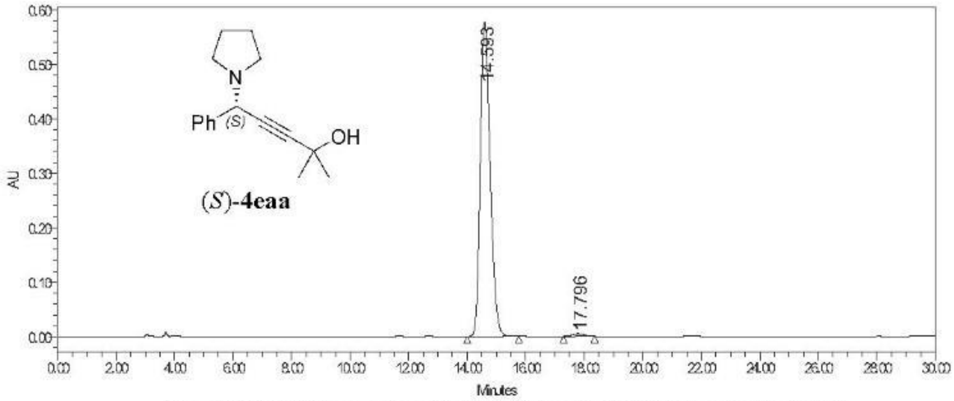

Processed Channel Descr.: W2489 ChA 214nm

	Processed Channel Descr.	RT	Area	%Area	Height
1	W2489 ChA 214nm	14.593	13530629	99.05	576095
2	W2489 ChA 214nm	17.796	129600	0.95	4864

Reported by User: System
Report Method: Injection Summary Report
Report Method ID: 1639 1639
Page: 1 of 1

Project Name: HPLC
Date Printed:
11/7/2019
6:43:31 AM PRC

HPLC spectrum for (*S*)-4eaa

Empower 3 SOFTWARE

Injection Summary Report

SAMPLE INFORMATION

Sample Name:	lq-6-165-od-h-99-1-1-214	Acquired By:	System
Sample Type:	Unknown	Sample Set Name:	
Vial:	1	Acq. Method Set:	HPLC
Injection #:	5	Processing Method:	Default
Injection Volume:	5.00 ul	Channel Name:	W2489 ChA
Run Time:	30.0 Minutes	Proc. Chnl. Descr.:	W2489 ChA 214nm
Date Acquired:	11/7/2019 2:48:44 AM CST		
Date Processed:	11/7/2019 6:42:12 AM CST		

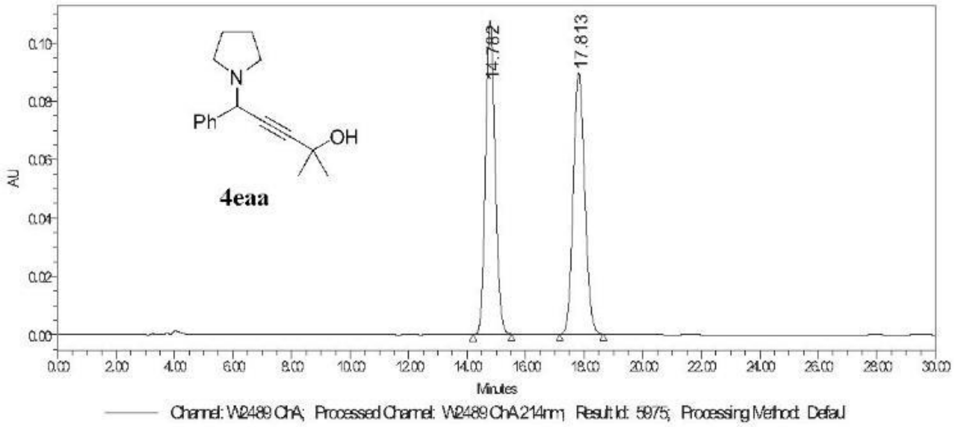

Processed Channel Descr.: W2489 ChA 214nm

	Processed Channel Descr.	RT	Area	%Area	Height
1	W2489 ChA 214nm	14.782	2439294	49.97	107373
2	W2489 ChA 214nm	17.813	2442077	50.03	89725

Reported by User: System
Report Method: Injection Summary Report
Report Method ID: 1639 1639
Page: 1 of 1

Project Name: HPLC
Date Printed:
11/7/2019
6:44:11 AM PRC

HPLC spectrum for (±)-4eaa

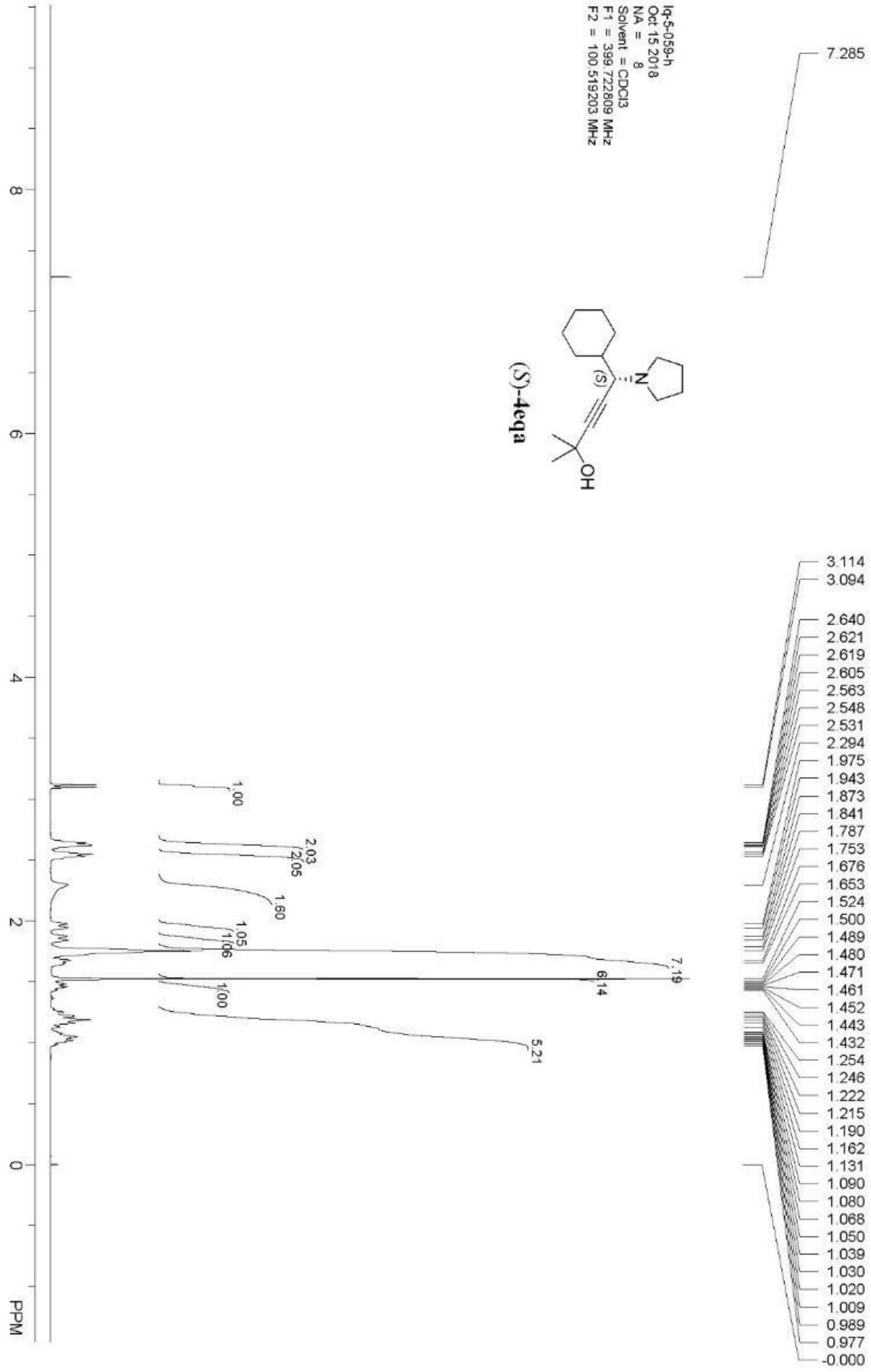

^{1}H NMR (400 MHz, CDCl3) spectrum for (*S*)-4eqa

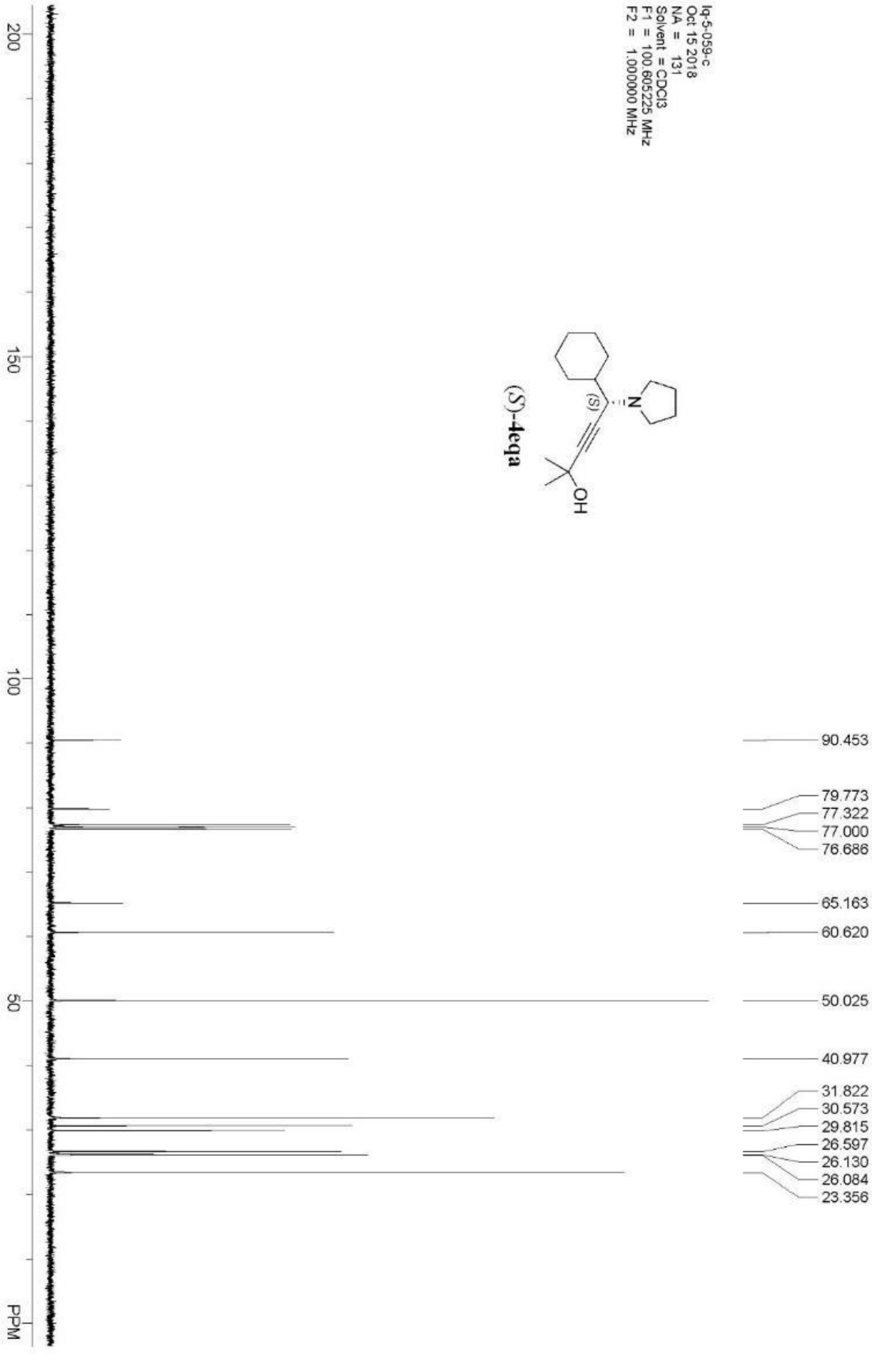

^{13}C NMR (400 MHz, $CDCl_3$) spectrum for (*S*)-4eqa

1q-5-059-od-h-200-1-1-214

实验时间：2018-10-17, 15:27:54　　报告时间：2018-10-17, 17:35:27
谱图文件:D:\zhuguangjiong\liuq\20181016\新建文件夹\1q-5-059-od-h-200-1-1-214.org

实验内容简介：

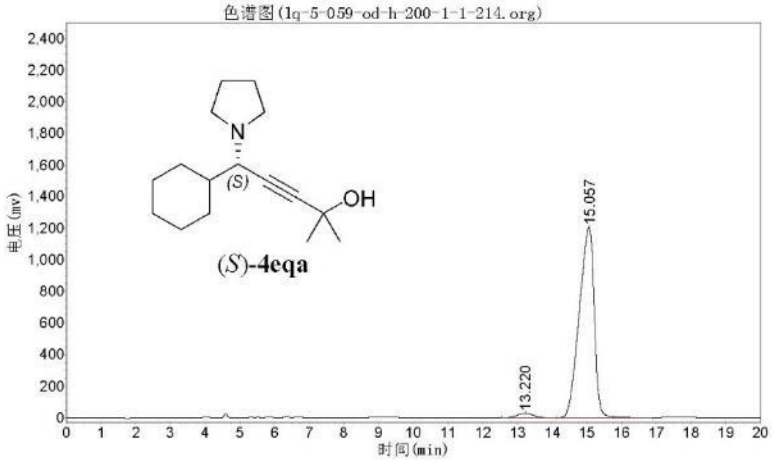

分析结果表

峰号	峰名	保留时间	峰高	峰面积	含量
1		13.220	24363.967	823396.813	2.2424
2		15.057	1201058.375	35895428.000	97.7576
总计			1225422.342	36718824.813	100.0000

HPLC spectrum for (*S*)-4eqa

zjs-1-40-od-h-200-1-1-214

实验时间：2018-10-17, 16:12:28　　　　报告时间：2018-10-17, 17:36:27
谱图文件:D:\zhuguangjiong\liuq\20181016\新建文件夹\zjs-1-40-od-h-200-1-1-214.....org

实验内容简介：

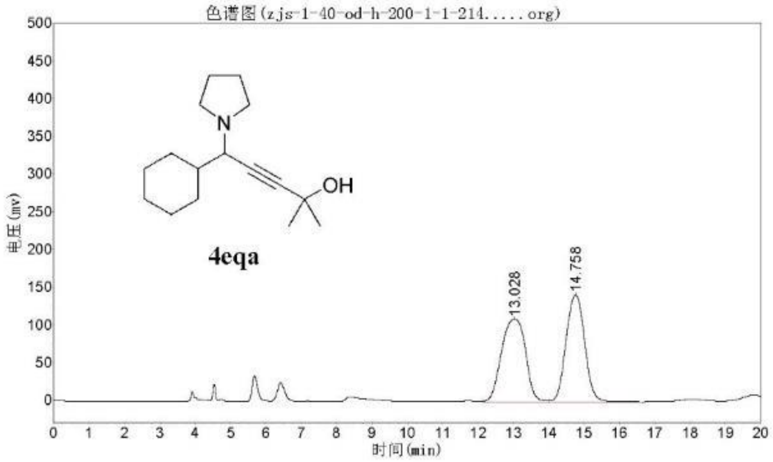

分析结果表

峰号	峰名	保留时间	峰高	峰面积	含量
1		13.028	109351.008	5173350.000	49.9272
2		14.758	141607.719	5188434.000	50.0728
总计			250958.727	10361784.000	100.0000

HPLC spectrum for (±)-4eqa

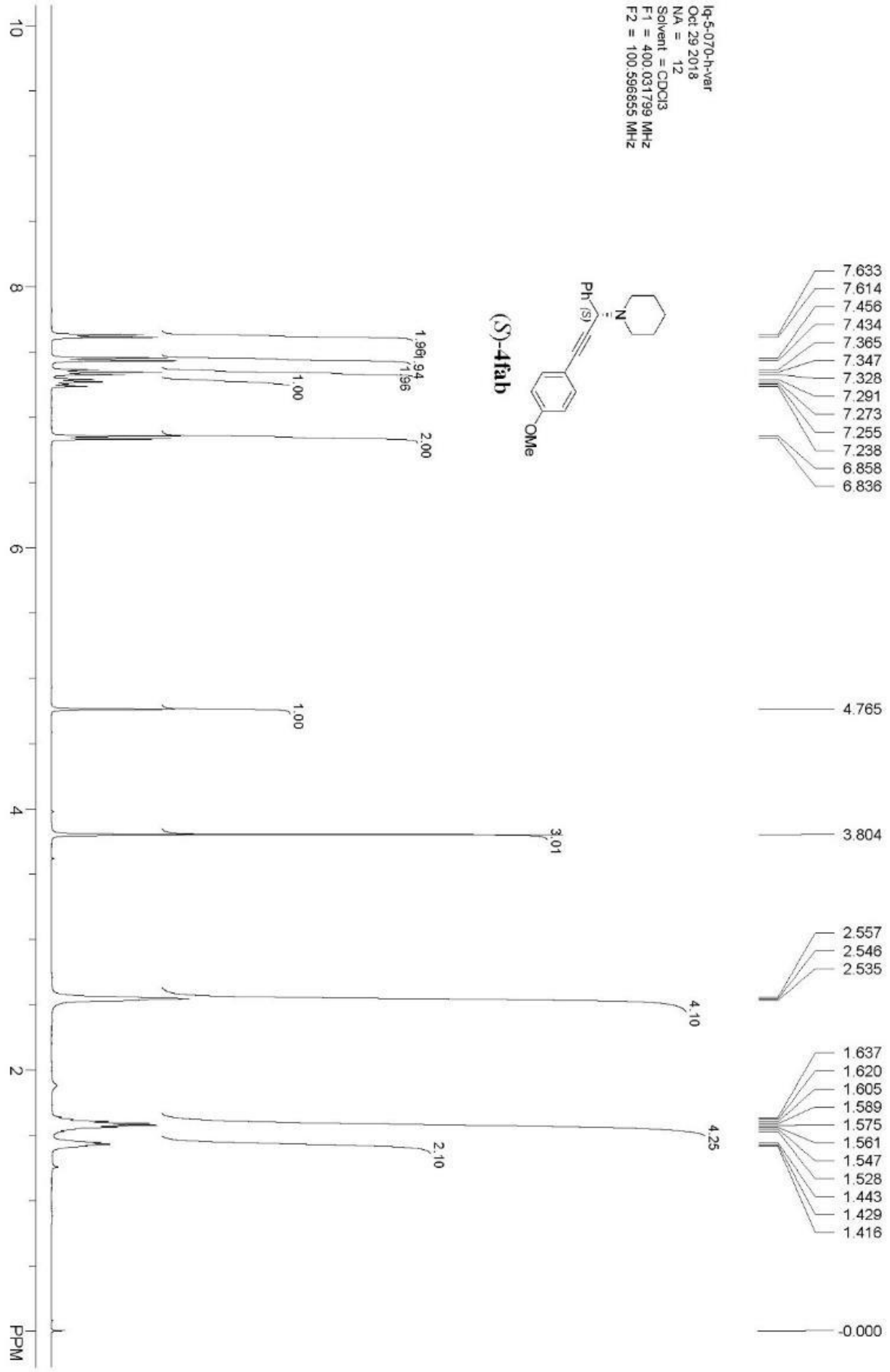

^{1}H NMR (400 MHz, CDCl3) spectrum for (*S*)-4fab

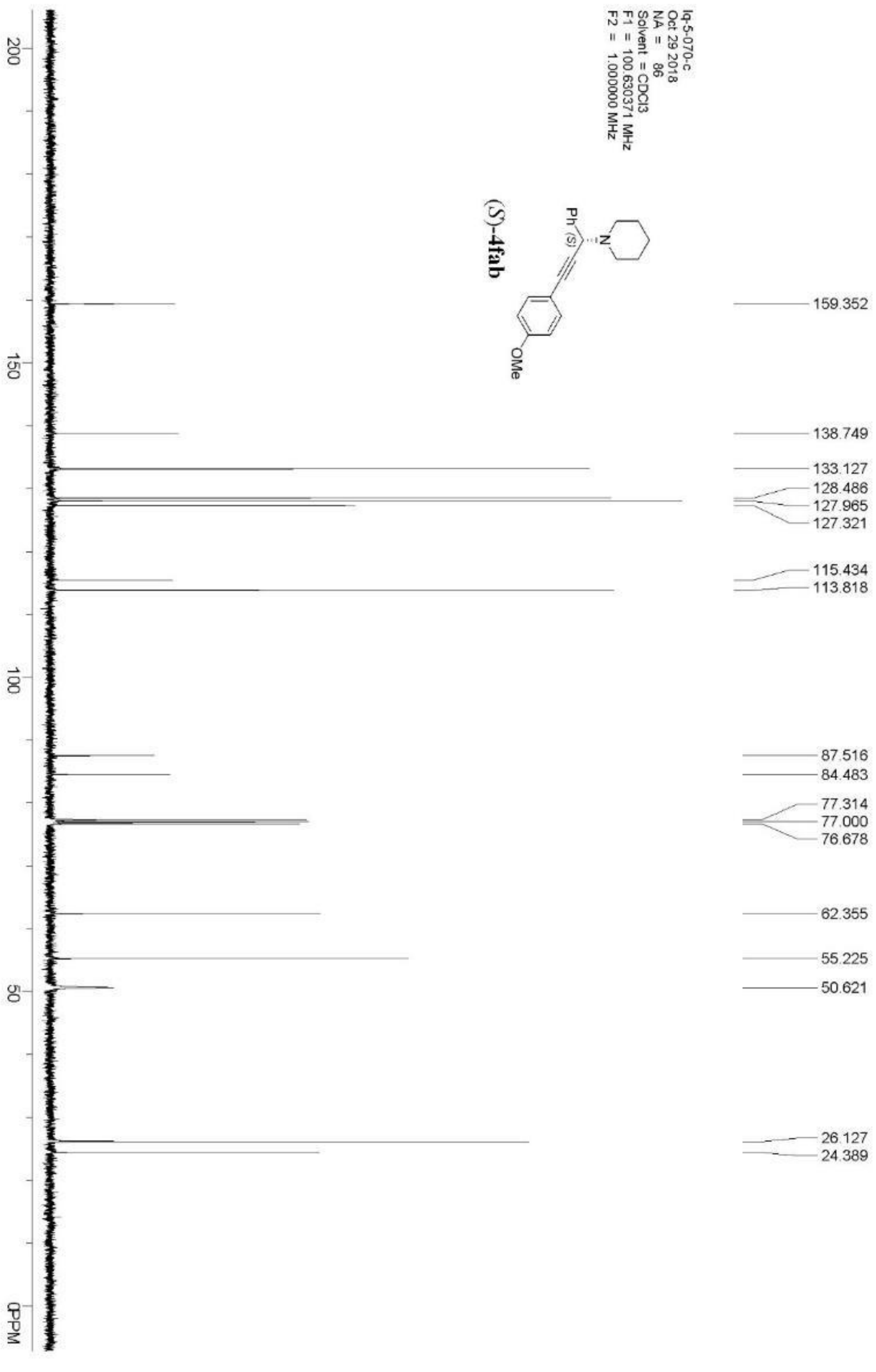

^{13}C NMR (400 MHz, CDCl$_3$) spectrum for (*S*)-4fab

lq-5-070

实验时间：2018/10/29, 13:58:24　　　　报告时间：2018/10/29, 13:58:27
谱图文件:D:\data\slf\lq\2018-10-29\lq-5-070-oj-h-99-1-0.5-214-2.mdy

实验内容简介：
OJ-H　99:1
214nm　0.5ml/min

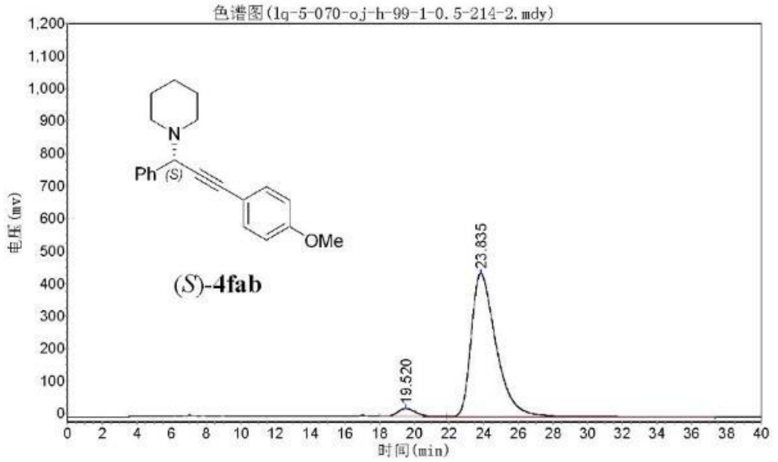

分析结果表

峰号	峰名	保留时间	峰高	峰面积	含量
1		19.520	24401.082	1802279.125	3.9322
2		23.835	440481.594	44031748.000	96.0678
**总计**			464882.676	45834027.125	100.0000

HPLC spectrum for (*S*)-4fab

xhb-1-093

实验时间：2018/10/29, 13:10:33　　　　报告时间：2018/10/29, 13:11:00
谱图文件:D:\data\slf\lq\2018-10-29\xhb-1-093-oj-h-99-1-0.5-214.mdy

实验内容简介：
OJ-H　99:1
214nm　0.5ml/min

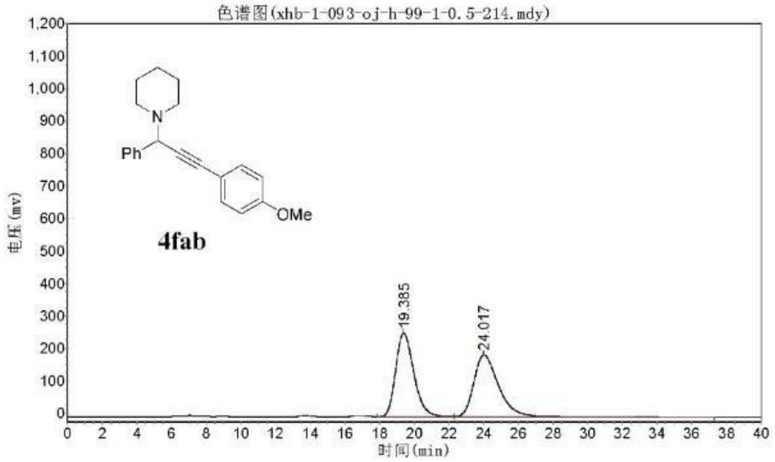

分析结果表

峰号	峰名	保留时间	峰高	峰面积	含量
1		19.385	257734.563	18888638.000	49.5937
2		24.017	190689.156	19198170.000	50.4063
**总计**			448423.719	38086808.000	100.0000

HPLC spectrum for (±)-4fab

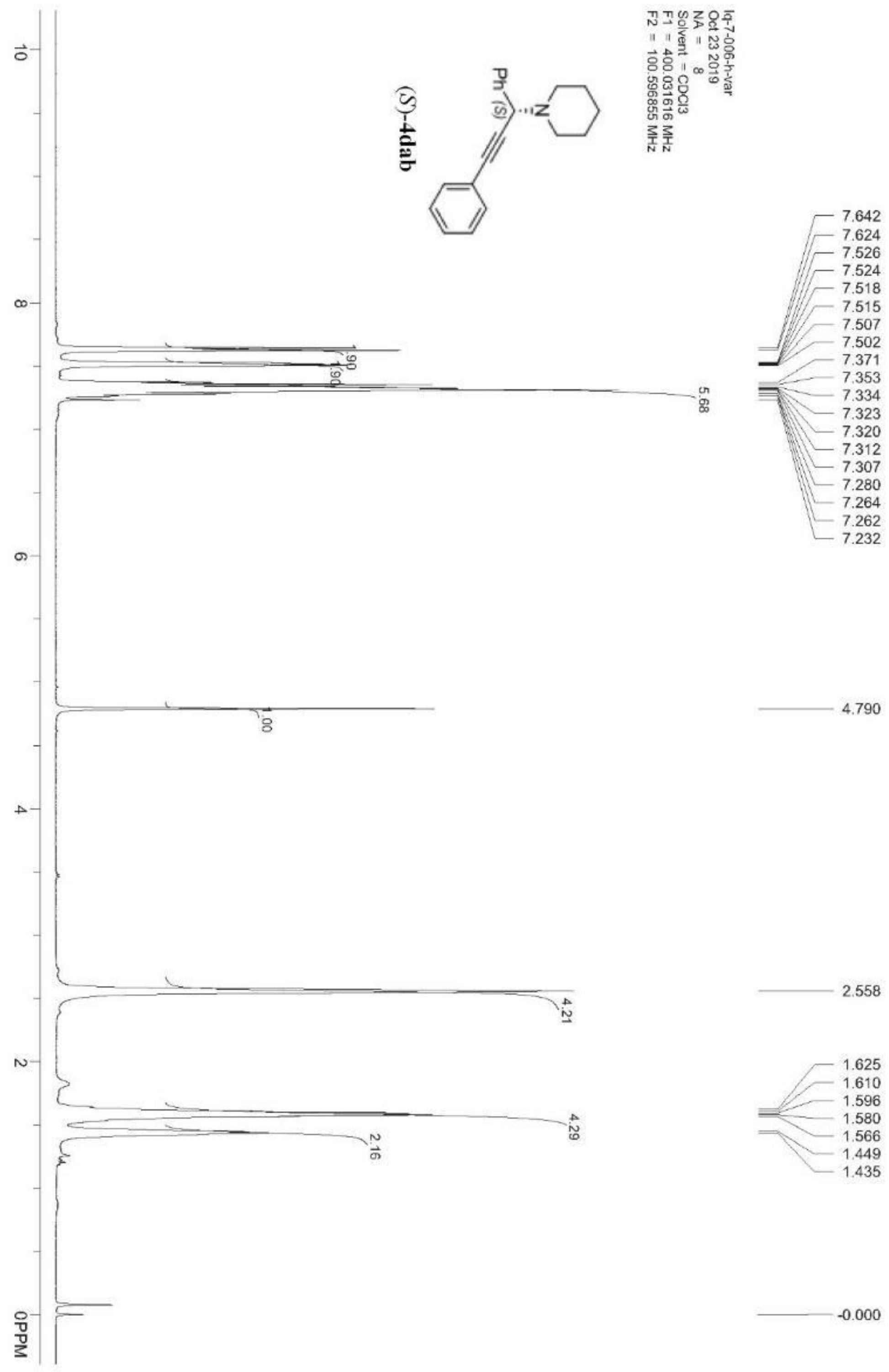

^{1}H NMR (400 MHz, CDCl3) spectrum for (*S*)-4dab

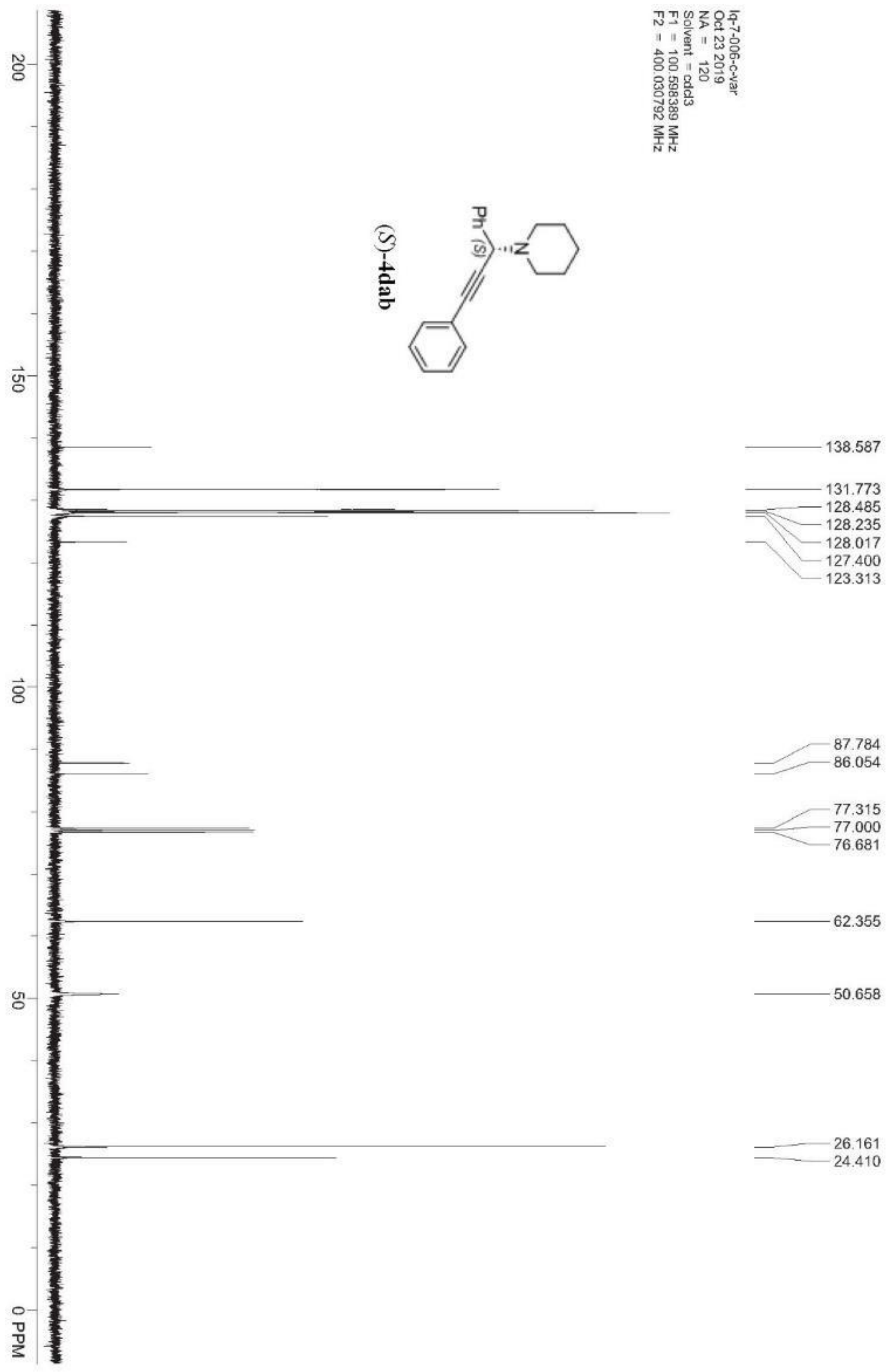

^{13}C NMR (400 MHz, $CDCl_3$) spectrum for (*S*)-4dab

Empower 3 SOFTWARE

Injection Summary Report

SAMPLE INFORMATION

Sample Name:	lq-7-006-oj-h-99-1-0.5-214	Acquired By:	System
Sample Type:	Unknown	Sample Set Name:	
Vial:	1	Acq. Method Set:	HPLC
Injection #:	4	Processing Method:	Default
Injection Volume:	2.00 ul	Channel Name:	W2489 ChA
Run Time:	20.0 Minutes	Proc. Chnl. Descr.:	W2489 ChA 214nm
Date Acquired:	10/20/2019 1:24:40 PM CST		
Date Processed:	10/21/2019 1:40:07 PM CST		

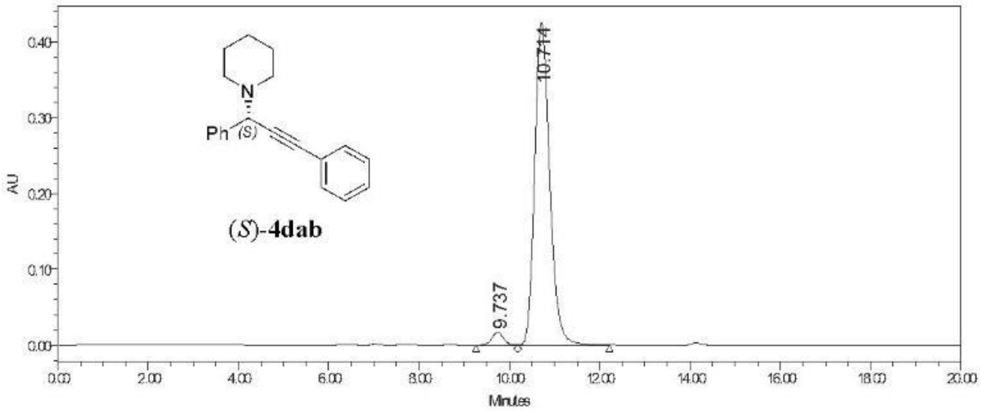

Channel: W2489 ChA; Processed Channel: W2489 ChA 214nm; Result Id: 5683; Processing Method: Defaul

Processed Channel Descr.: W2489 ChA 214nm

	Processed Channel Descr.	RT	Area	%Area	Height
1	W2489 ChA 214nm	9.737	300191	2.98	17420
2	W2489 ChA 214nm	10.714	9783893	97.02	425529

Reported by User: System
Report Method: Injection Summary Report
Report Method ID: 1639 1639
Page: 1 of 1

Project Name: HPLC
Date Printed:
10/21/2019
1:42:00 PM PRC

HPLC spectrum for (*S*)-4dab

Empower 3 SOFTWARE

Injection Summary Report

SAMPLE INFORMATION

Sample Name:	lq-7-005-oj-h-99-1-0.5-214	Acquired By:	System
Sample Type:	Unknown	Sample Set Name:	
Vial:	1	Acq. Method Set:	HPLC
Injection #:	2	Processing Method:	Default
Injection Volume:	3.00 ul	Channel Name:	W2489 ChA
Run Time:	30.0 Minutes	Proc. Chnl. Descr.:	W2489 ChA 214nm
Date Acquired:	10/20/2019 12:35:18 PM CST		
Date Processed:	10/21/2019 1:38:40 PM CST		

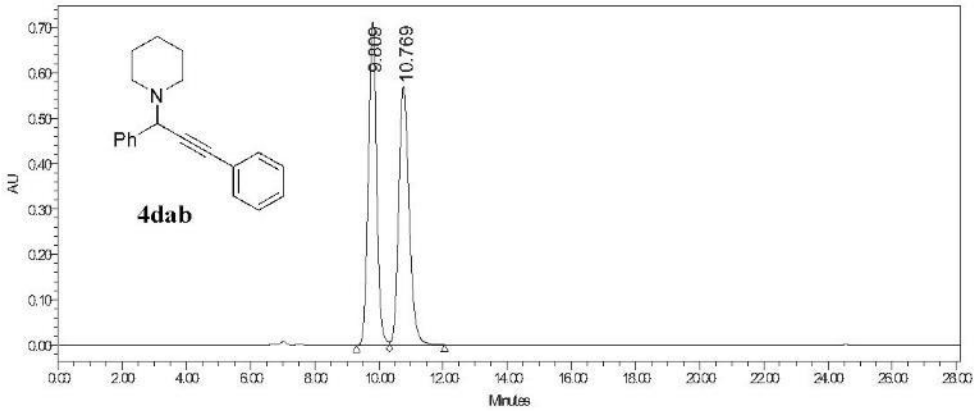

Processed Channel Descr.: W2489 ChA 214nm

	Processed Channel Descr.	RT	Area	% Area	Height
1	W2489 ChA 214nm	9.809	12675653	49.75	712184
2	W2489 ChA 214nm	10.769	12803803	50.25	569656

Reported by User: System
Report Method: Injection Summary Report
Report Method ID: 1639 1639
Page: 1 of 1

Project Name: HPLC
Date Printed:
10/21/2019
1:40:59 PM PRC

HPLC spectrum for (±)-4dab

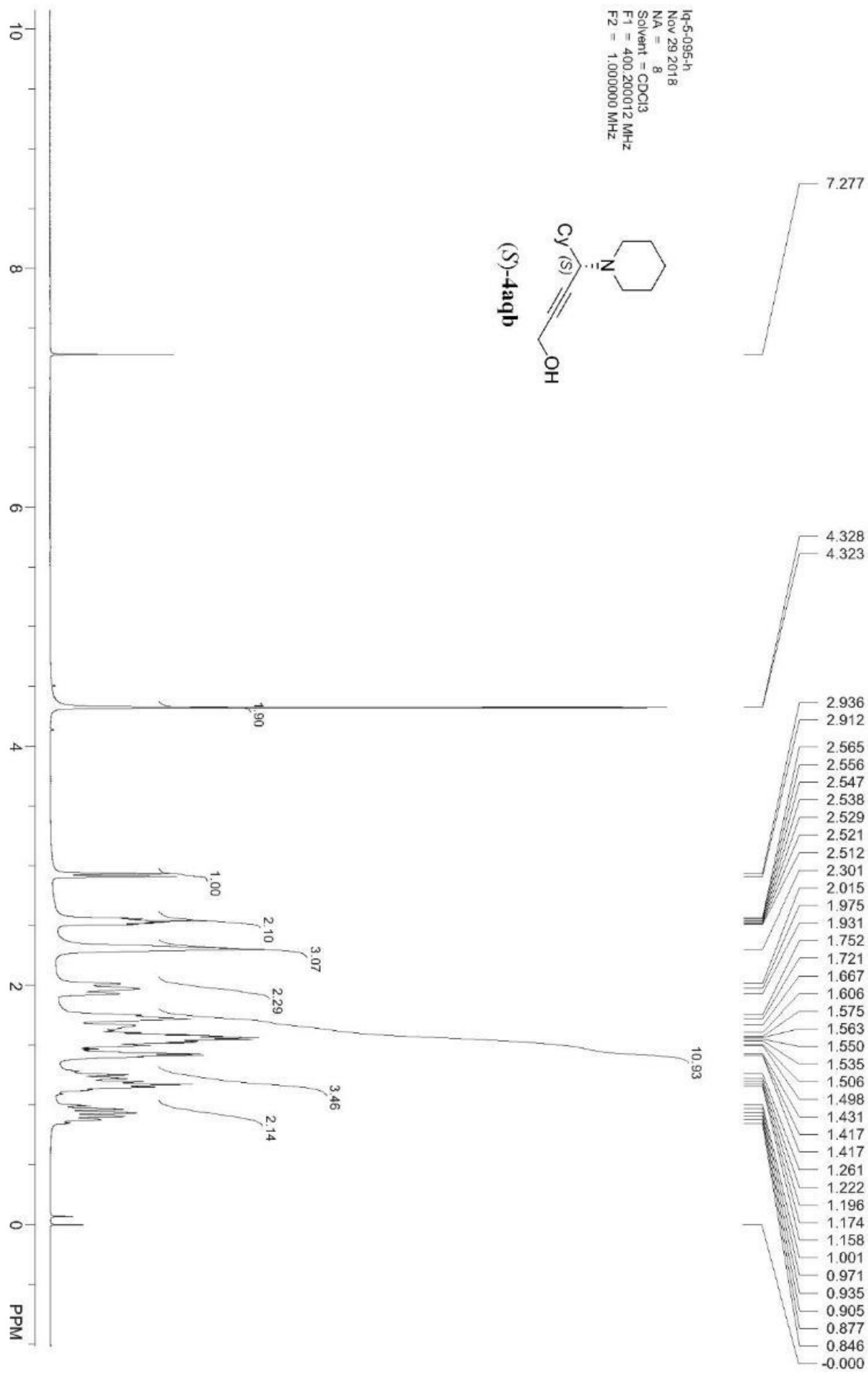

^{1}H NMR (400 MHz, CDCl3) spectrum for (*S*)-4aqb

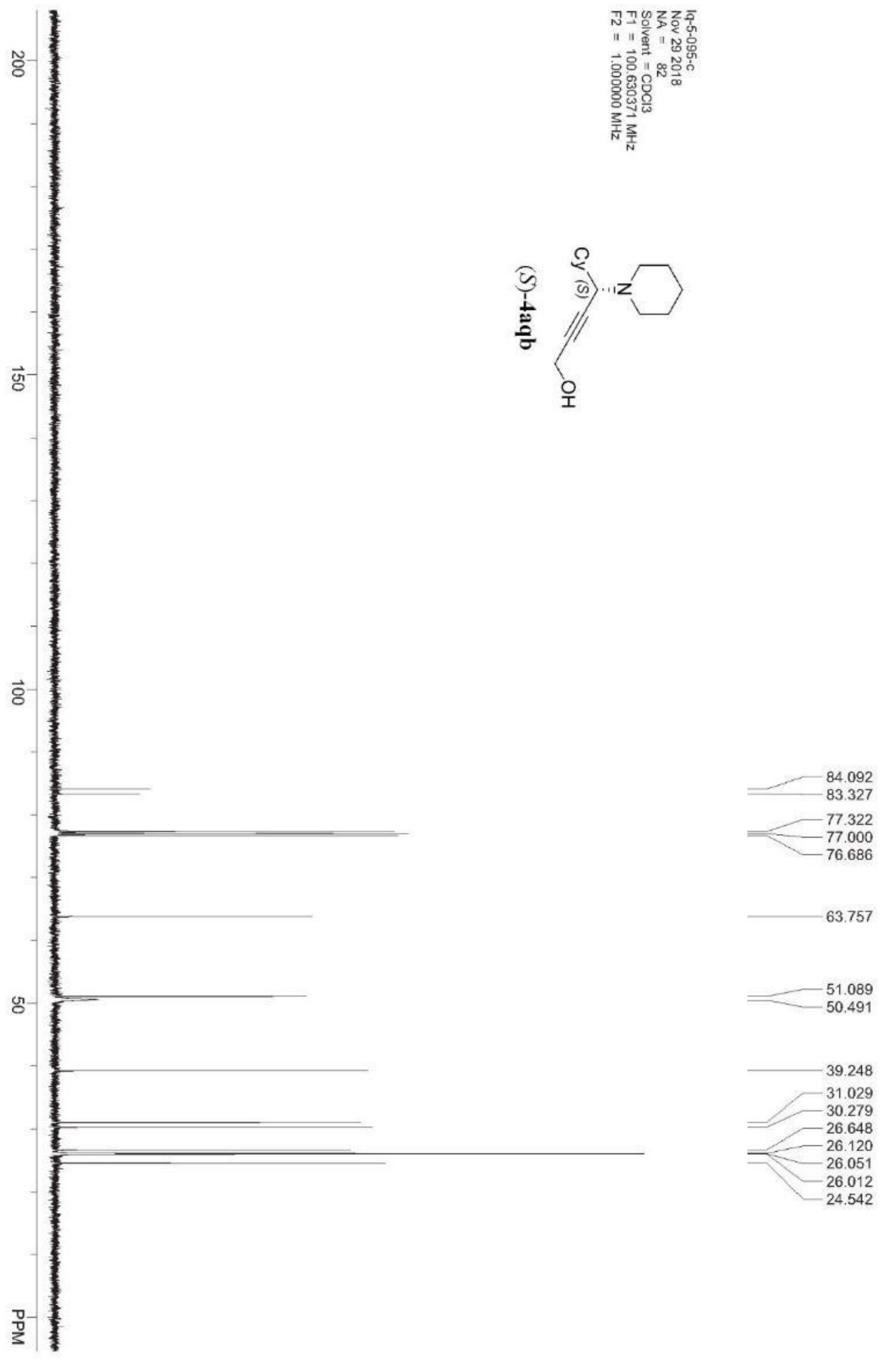

^{13}C NMR (400 MHz, $CDCl_3$) spectrum for (*S*)-4aqb

Empower 3 SOFTWARE

Injection Summary Report

SAMPLE INFORMATION

Sample Name:	lq-5-095-ad-h-99-1-1.0-214	Acquired By:	System
Sample Type:	Unknown	Sample Set Name:	
Vial:	1	Acq. Method Set:	HPLC
Injection #:	1	Processing Method:	20181124
Injection Volume:	5.00 ul	Channel Name:	W2489 ChA
Run Time:	40.0 Minutes	Proc. Chnl. Descr.:	W2489 ChA 214nm
Date Acquired:	11/30/2018 3:28:50 AM CST		
Date Processed:	11/30/2018 4:23:44 AM CST		

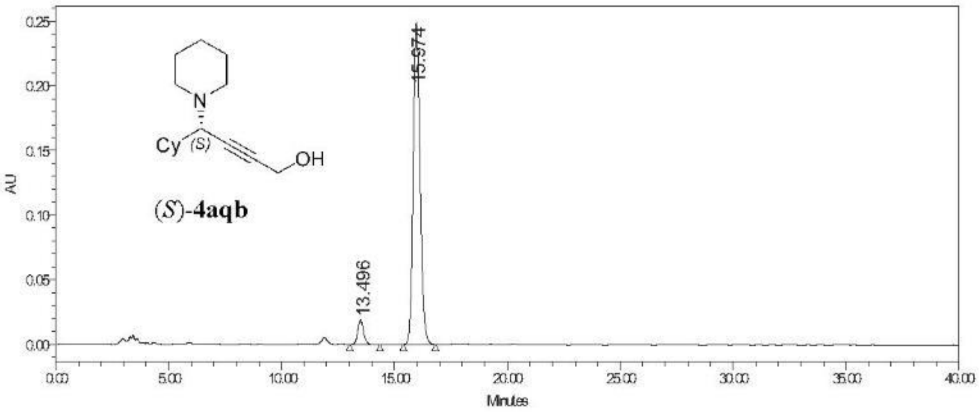

Channel: W2489 ChA; Processed Channel: W2489 ChA 214nm; Result Id: 1274; Processing Method: 2018112

Processed Channel Descr.: W2489 ChA 214nm

	Processed Channel Descr.	RT	Area	%Area	Height
1	W2489 ChA 214nm	13.496	334949	5.99	18911
2	W2489 ChA 214nm	15.974	5260531	94.01	249295

Reported by User: System
Report Method: Injection Summary Report
Report Method ID: 1007 1007
Page: 1 of 1

Project Name: HPLC
Date Printed:
11/30/2018
5:22:40 AM PRC

HPLC spectrum for (*S*)-4aqb

Empower 3 SOFTWARE

Injection Summary Report

SAMPLE INFORMATION

Sample Name:	lq-5-088-ad-h-99-1-1.0-214	Acquired By:	System
Sample Type:	Unknown	Sample Set Name:	
Vial:	1	Acq. Method Set:	HPLC
Injection #:	1	Processing Method:	20181124
Injection Volume:	5.00 ul	Channel Name:	W2489 ChA
Run Time:	40.0 Minutes	Proc. Chnl. Descr.:	W2489 ChA 214nm
Date Acquired:	11/30/2018 4:44:58 AM CST		
Date Processed:	11/30/2018 5:23:43 AM CST		

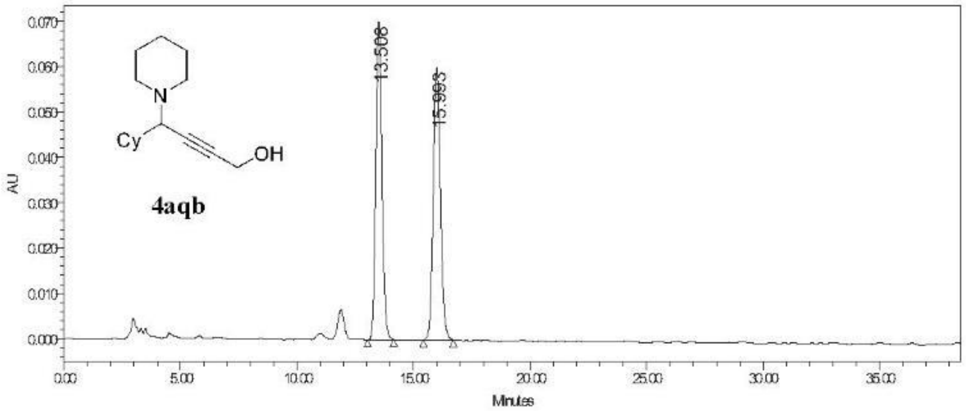

Channel: W2489 ChA; Processed Channel: W2489 ChA 214nm; Result Id: 1275; Processing Method: 2018112[illegible]

Processed Channel Descr.: W2489 ChA 214nm

	Processed Channel Descr.	RT	Area	%Area	Height
1	W2489 ChA 214nm	13.508	1279023	49.80	70184
2	W2489 ChA 214nm	15.993	1289039	50.20	60090

Reported by User: System
Report Method: Injection Summary Report
Report Method ID: 1007 1007
Page: 1 of 1

Project Name: HPLC
Date Printed:
11/30/2018
5:24:02 AM PRC

HPLC spectrum for (±)-4aqb

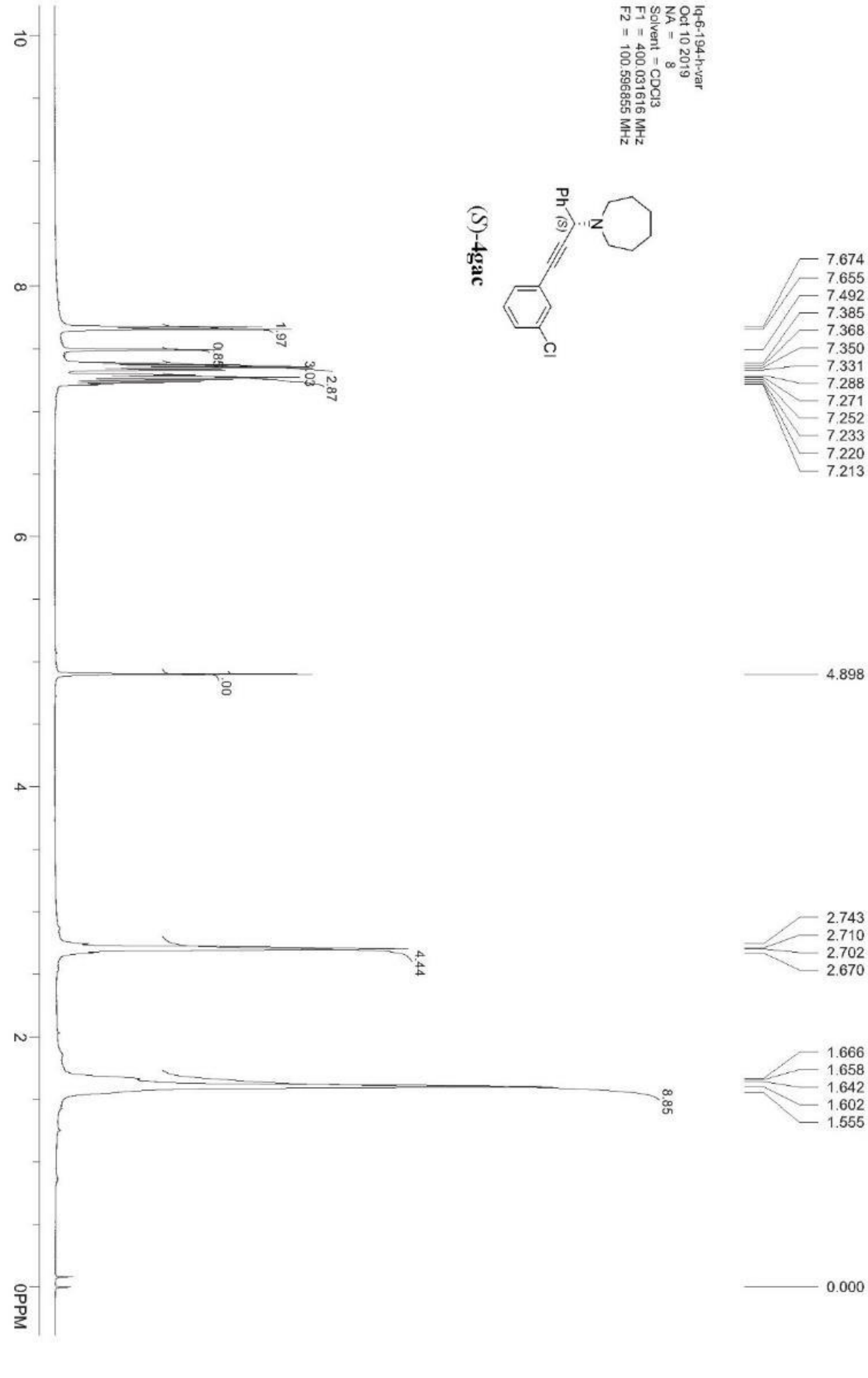

^{1}H NMR (400 MHz, CDCl3) spectrum for (*S*)**-4gac**

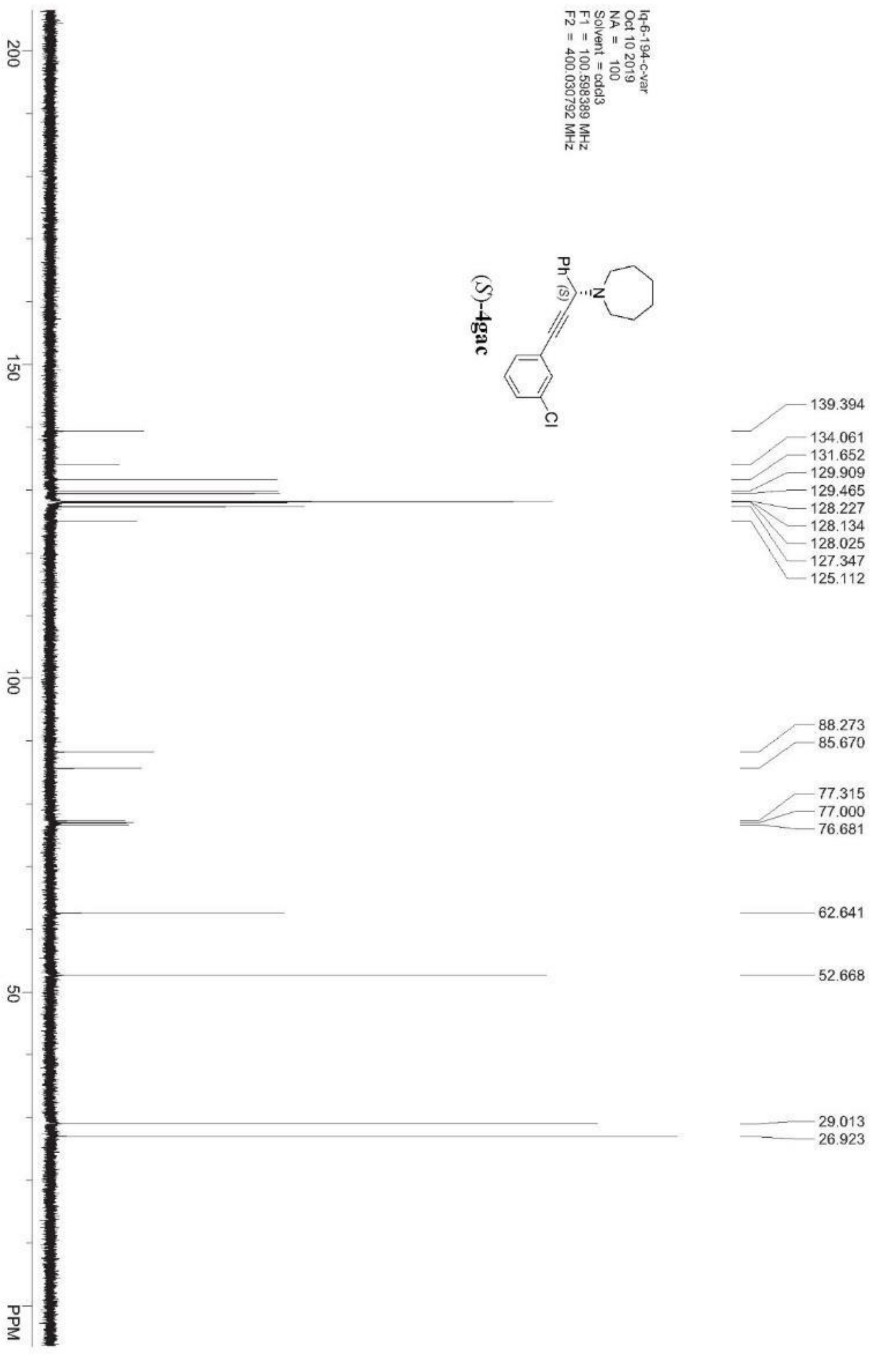

^{13}C NMR (400 MHz, $CDCl_3$) spectrum for (*S*)**-4gac**

Empower 3 SOFTWARE

Injection Summary Report

SAMPLE INFORMATION

Sample Name:	lq-6-194-ad-h-100-0-0.2-214	Acquired By:	System
Sample Type:	Unknown	Sample Set Name:	
Vial:	1	Acq. Method Set:	HPLC
Injection #:	2	Processing Method:	Default
Injection Volume:	2.00 ul	Channel Name:	W2489 ChA
Run Time:	50.0 Minutes	Proc. Chnl. Descr.:	W2489 ChA 214nm
Date Acquired:	10/9/2019 12:36:38 PM CST		
Date Processed:	10/10/2019 5:55:17 AM CST		

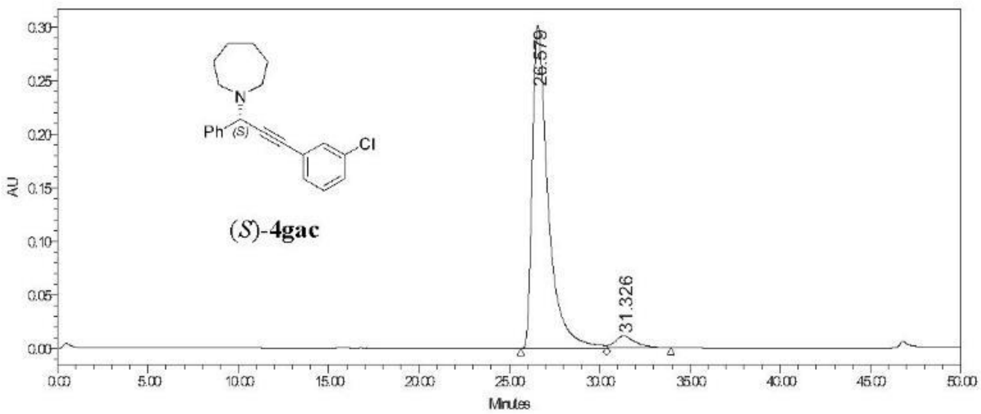

Processed Channel Descr.: W2489 ChA 214nm

	Processed Channel Descr.	RT	Area	% Area	Height
1	W2489 ChA 214nm	26.579	18351667	95.39	301224
2	W2489 ChA 214nm	31.326	886980	4.61	11235

Reported by User: System
Report Method: Injection Summary Report
Report Method ID: 1639 1639
Page: 1 of 1

Project Name: HPLC
Date Printed:
10/10/2019
5:57:32 AM PRC

HPLC spectrum for (*S*)-4gac

Empower 3 SOFTWARE

Injection Summary Report

SAMPLE INFORMATION

Sample Name:	lq-6-192-ad-h-100-0-0.2-214	Acquired By:	System
Sample Type:	Unknown	Sample Set Name:	
Vial:	1	Acq. Method Set:	HPLC
Injection #:	1	Processing Method:	Default
Injection Volume:	2.00 ul	Channel Name:	W2489 ChA
Run Time:	60.0 Minutes	Proc. Chnl. Descr.:	W2489 ChA 214nm
Date Acquired:	10/9/2019 11:42:35 AM CST		
Date Processed:	10/10/2019 5:56:02 AM CST		

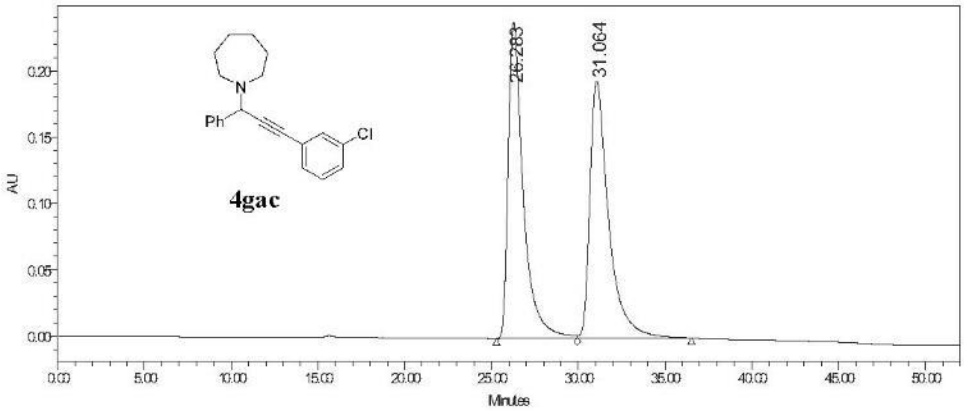

Channel: W2489 ChA; Processed Channel: W2489 ChA 214nm; Result Id: 5334; Processing Method: Defaul

Processed Channel Descr.: W2489 ChA 214nm

	Processed Channel Descr.	RT	Area	% Area	Height
1	W2489 ChA 214nm	26.283	14368629	49.77	238301
2	W2489 ChA 214nm	31.064	14500293	50.23	193631

Reported by User: System
Report Method: Injection Summary Report
Report Method ID: 1639 1639
Page: 1 of 1

Project Name: HPLC
Date Printed:
10/10/2019
5:56:17 AM PRC

HPLC spectrum for (±)-4gac

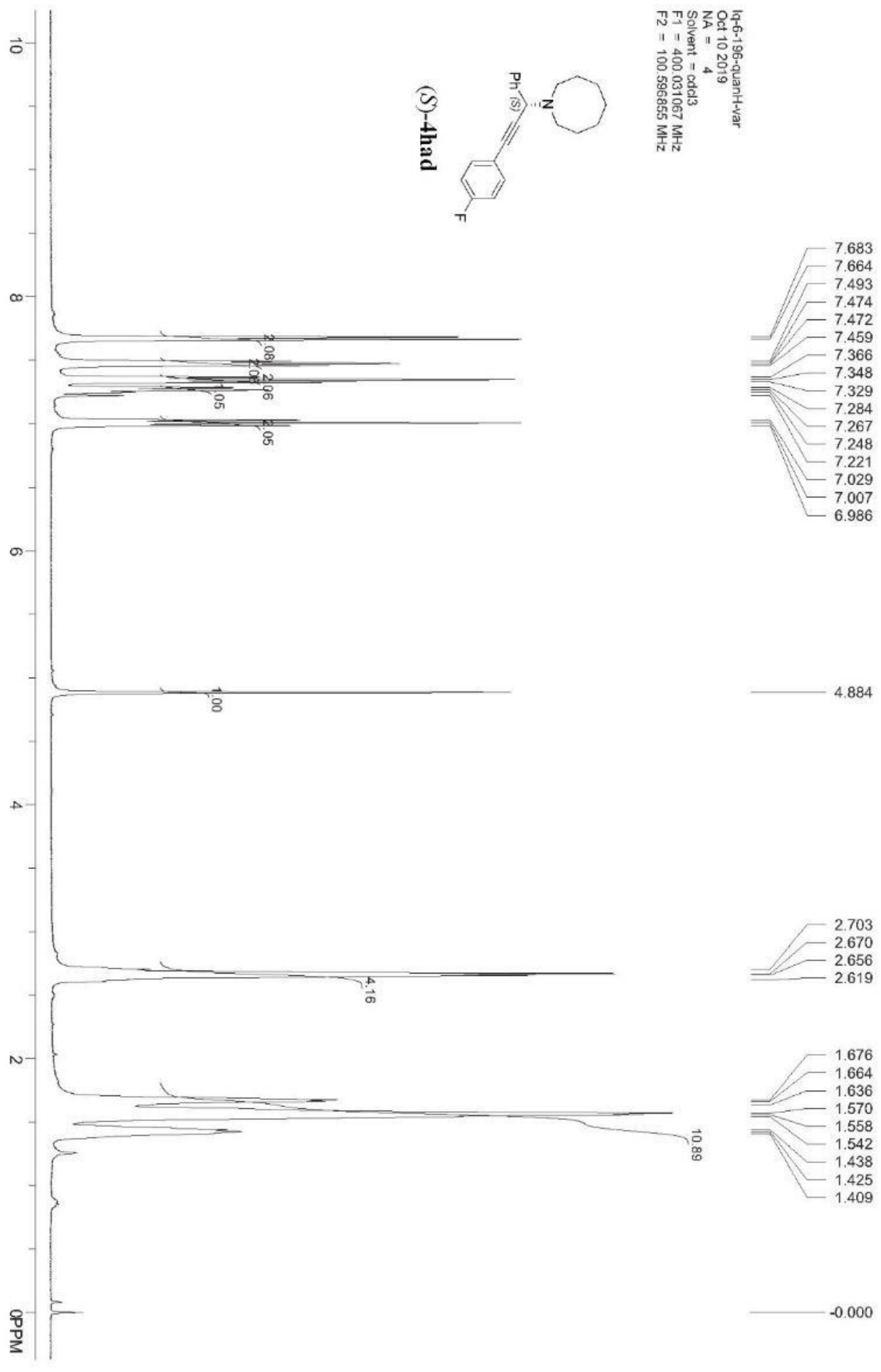

^{1}H NMR (400 MHz, CDCl3) spectrum for (*S*)-4had

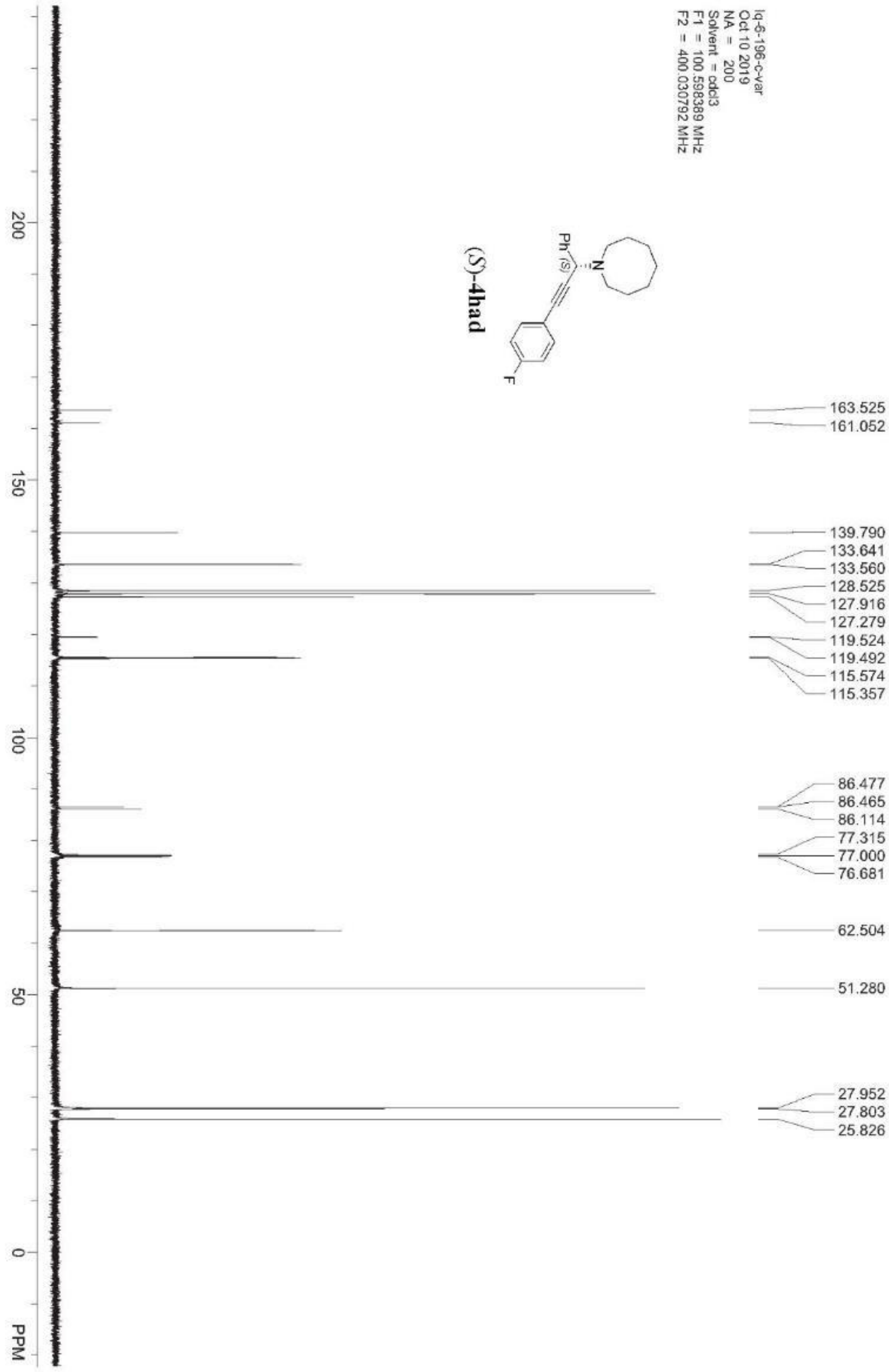

^{13}C NMR (400 MHz, CDCl3) spectrum for (*S*)-4had

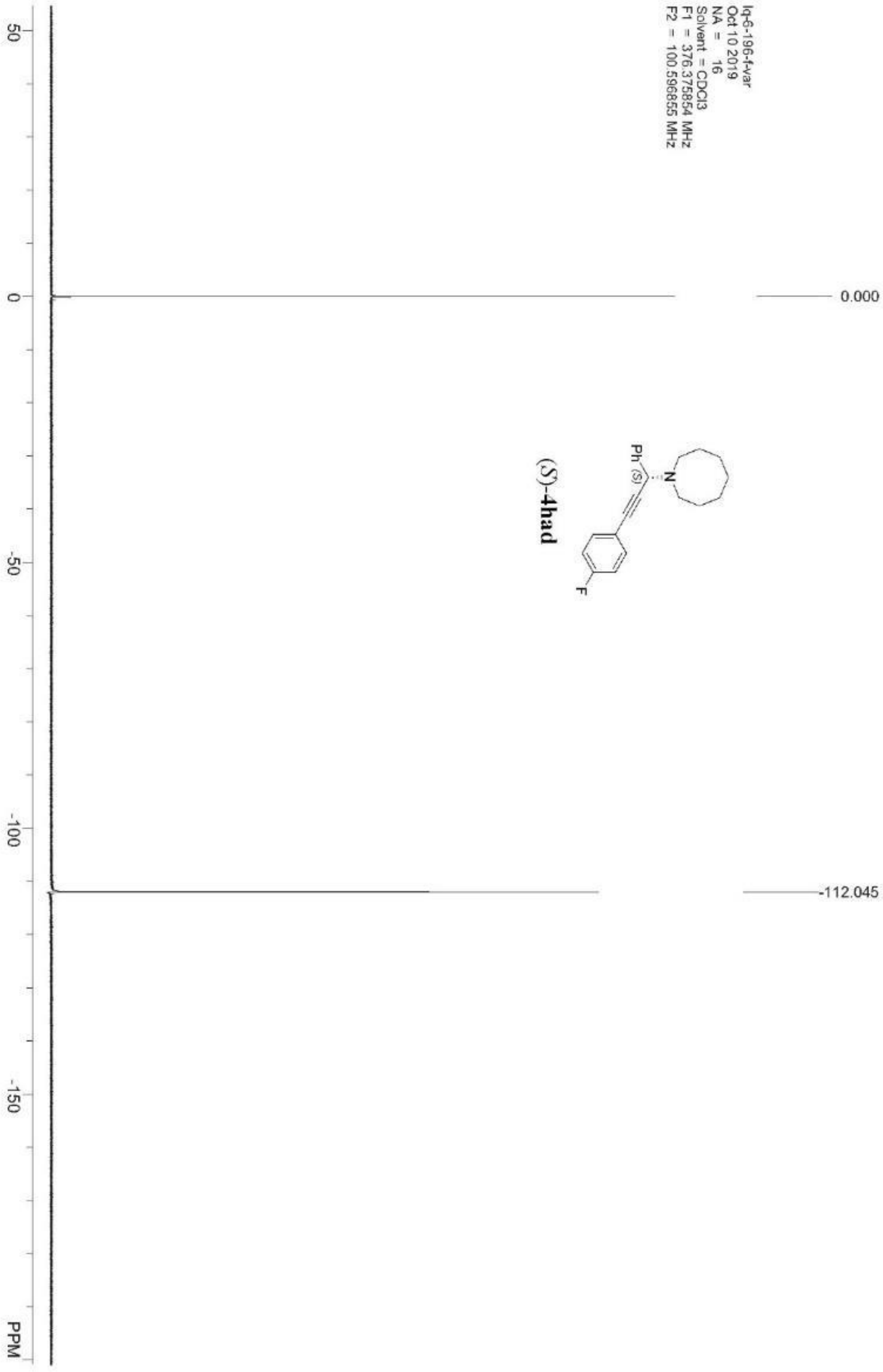

^{19}F NMR (376 MHz, CDCl3) spectrum for (*S*)-4had (CFCl$_3$ was used as the internal standard)

Empower™ 3 SOFTWARE

Injection Summary Report

SAMPLE INFORMATION

Sample Name:	lq-6-196-ad-h-100-0-0.2-214	Acquired By:	System
Sample Type:	Unknown	Sample Set Name:	
Vial:	1	Acq. Method Set:	HPLC
Injection #:	1	Processing Method:	Default
Injection Volume:	2.00 ul	Channel Name:	W2489 ChA
Run Time:	50.0 Minutes	Proc. Chnl. Descr.:	W2489 ChA 214nm
Date Acquired:	10/11/2019 1:00:17 AM CST		
Date Processed:	10/11/2019 1:51:47 AM CST		

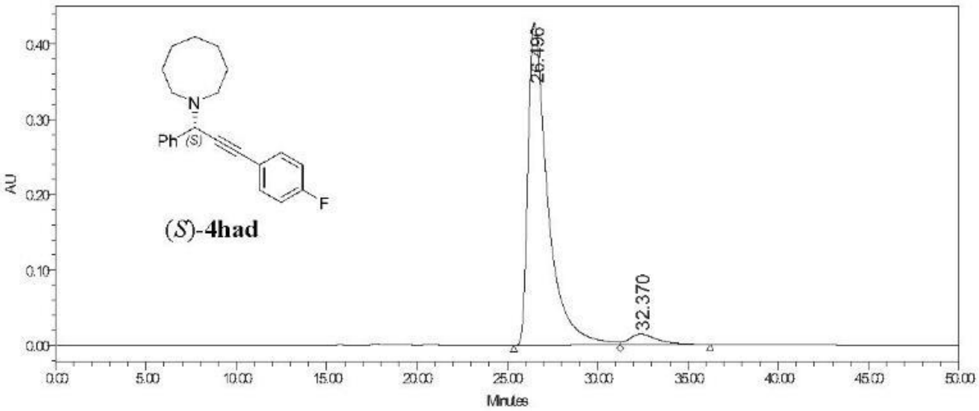

Channel: W2489 ChA; Processed Channel: W2489 ChA 214nm; Result Id: 5370; Processing Method: Defaul

Processed Channel Descr.: W2489 ChA 214nm

	Processed Channel Descr.	RT	Area	% Area	Height
1	W2489 ChA 214nm	26.496	32405941	95.38	428624
2	W2489 ChA 214nm	32.370	1570106	4.62	14252

Reported by User: System
Report Method: Injection Summary Report
Report Method ID: 1639 1639
Page: 1 of 1

Project Name: HPLC
Date Printed:
10/11/2019
5:43:01 AM PRC

HPLC spectrum for (*S*)-4had

Empower 3 SOFTWARE

Injection Summary Report

SAMPLE INFORMATION

Sample Name:	lq-6-195-ad-h-100-0-0.2-214	Acquired By:	System
Sample Type:	Unknown	Sample Set Name:	
Vial:	1	Acq. Method Set:	HPLC
Injection #:	4	Processing Method:	Default
Injection Volume:	4.00 ul	Channel Name:	W2489 ChA
Run Time:	50.0 Minutes	Proc. Chnl. Descr.:	W2489 ChA 214nm
Date Acquired:	10/11/2019 4:34:33 AM CST		
Date Processed:	10/11/2019 5:43:54 AM CST		

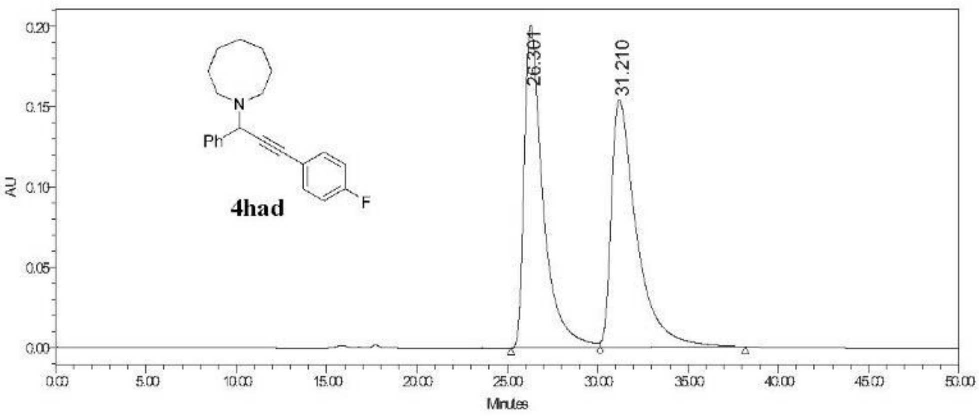

Processed Channel Descr.: W2489 ChA 214nm

	Processed Channel Descr.	RT	Area	% Area	Height
1	W2489 ChA 214nm	26.301	14583218	49.29	200777
2	W2489 ChA 214nm	31.210	15001791	50.71	154214

Reported by User: System
Report Method: Injection Summary Report
Report Method ID: 1639 1639
Page: 1 of 1

Project Name: HPLC
Date Printed:
10/11/2019
5:45:00 AM PRC

HPLC spectrum for (±)-4had

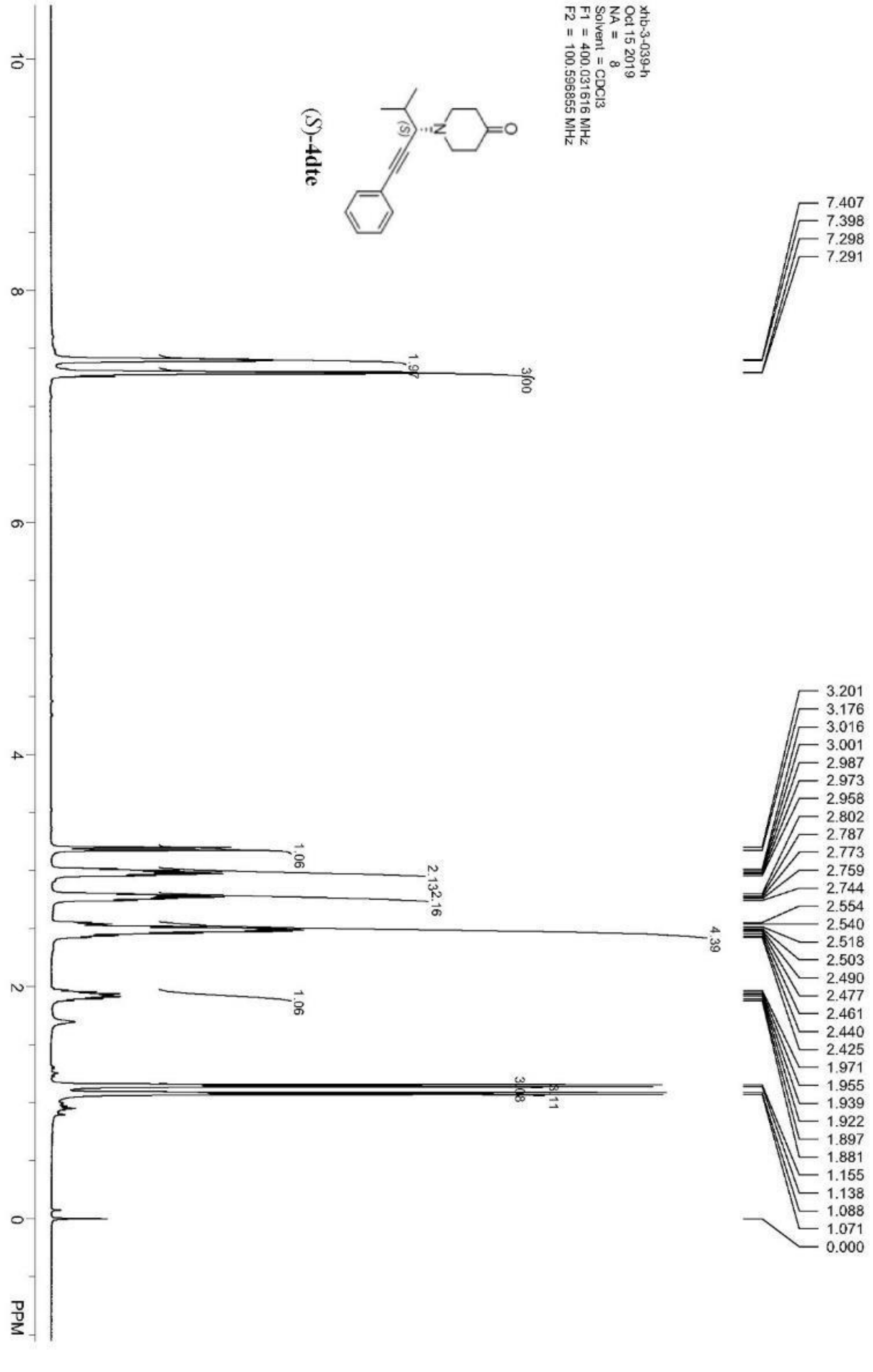

^{1}H NMR (400 MHz, CDCl3) spectrum for (*S*)**-4dte**

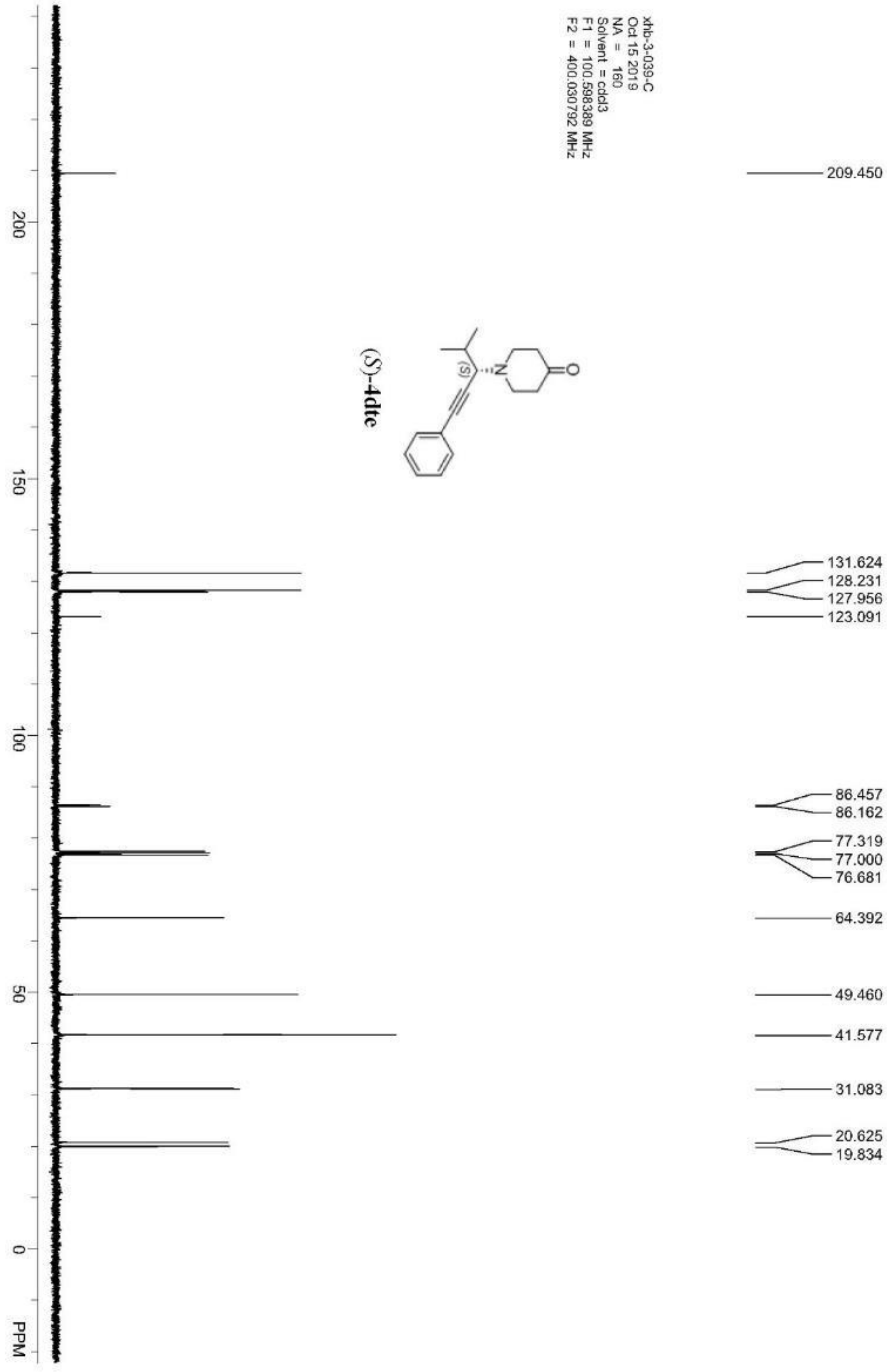

^{13}C NMR (400 MHz, $CDCl_3$) spectrum for (*S*)-4dte

Empower 3 SOFTWARE

Injection Summary Report

SAMPLE INFORMATION

Sample Name:	xhb-3-39-oj-h-99-1-0.5-214	Acquired By:	System
Sample Type:	Unknown	Sample Set Name:	
Vial:	1	Acq. Method Set:	HPLC
Injection #:	3	Processing Method:	LC PQ
Injection Volume:	7.50 ul	Channel Name:	W2489 ChA
Run Time:	45.0 Minutes	Proc. Chnl. Descr.:	W2489 ChA 214nm
Date Acquired:	10/15/2019 7:43:50 AM CST		
Date Processed:	10/15/2019 8:34:18 AM CST		

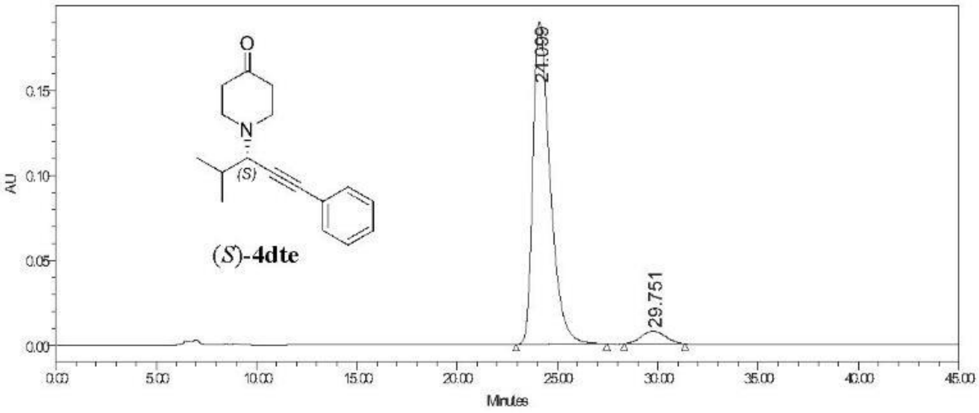

Processed Channel Descr.: W2489 ChA 214nm

	Processed Channel Descr.	RT	Area	% Area	Height
1	W2489 ChA 214nm	24.099	11248758	94.81	189339
2	W2489 ChA 214nm	29.751	615965	5.19	7376

Reported by User: System
Report Method: Injection Summary Report
Report Method ID: 1639 1639
Page: 1 of 1

Project Name: HPLC
Date Printed:
10/15/2019
10:48:09 AM PRC

HPLC spectrum for (*S*)-4dte

Empower™ 3 SOFTWARE

Injection Summary Report

SAMPLE INFORMATION

Sample Name:	xhb-3-32-oj-h-99-1-0.5-214	Acquired By:	System
Sample Type:	Unknown	Sample Set Name:	
Vial:	1	Acq. Method Set:	HPLC
Injection #:	4	Processing Method:	LC PQ
Injection Volume:	5.00 ul	Channel Name:	W2489 ChA
Run Time:	45.0 Minutes	Proc. Chnl. Descr.:	W2489 ChA 214nm
Date Acquired:	10/15/2019 9:55:19 AM CST		
Date Processed:	10/15/2019 10:47:41 AM CST		

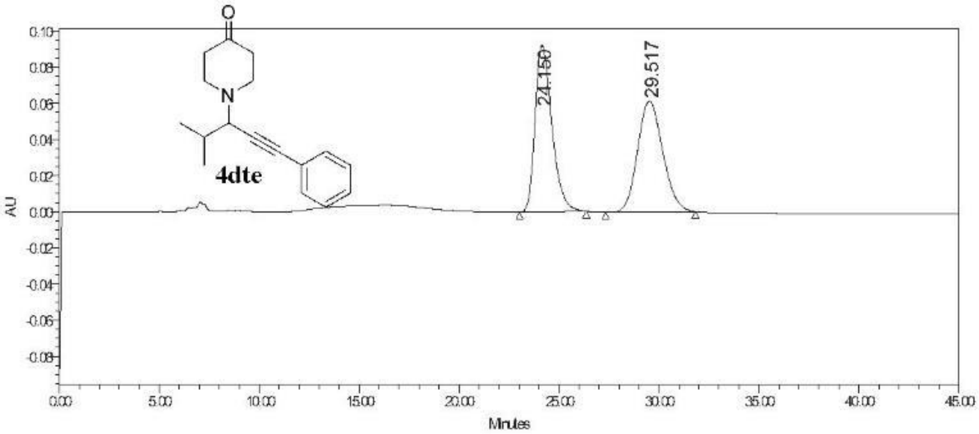

Processed Channel Descr.: W2489 ChA 214nm

	Processed Channel Descr.	RT	Area	%Area	Height
1	W2489 ChA 214nm	24.150	5377252	50.10	92254
2	W2489 ChA 214nm	29.517	5356113	49.90	61305

Reported by User: System
Report Method: Injection Summary Report
Report Method ID: 1639 1639
Page: 1 of 1

Project Name: HPLC
Date Printed:
10/15/2019
10:48:50 AM PRC

HPLC spectrum for (±)-4dte

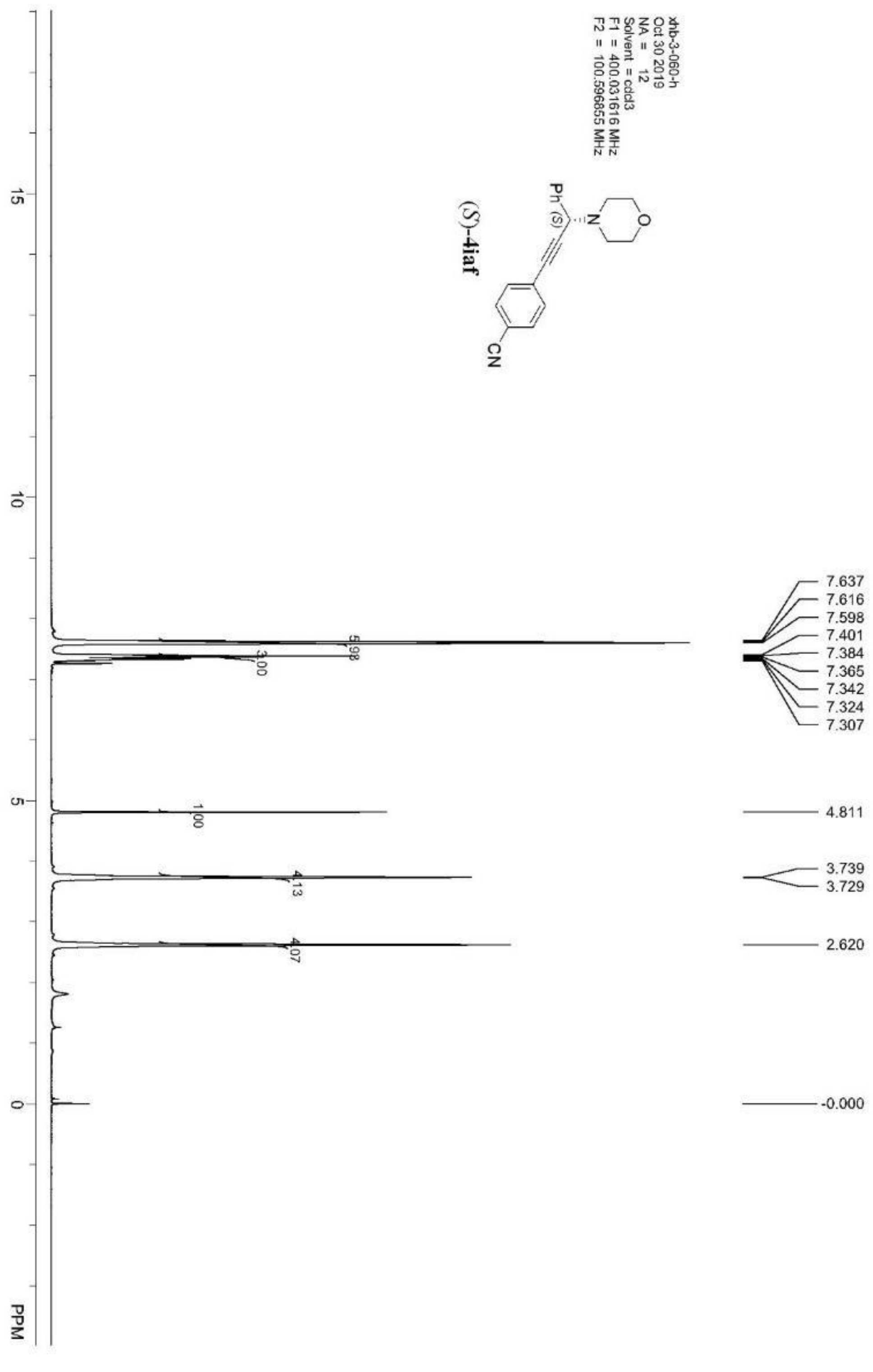

^{1}H NMR (400 MHz, CDCl3) spectrum for (*S*)-**4iaf**

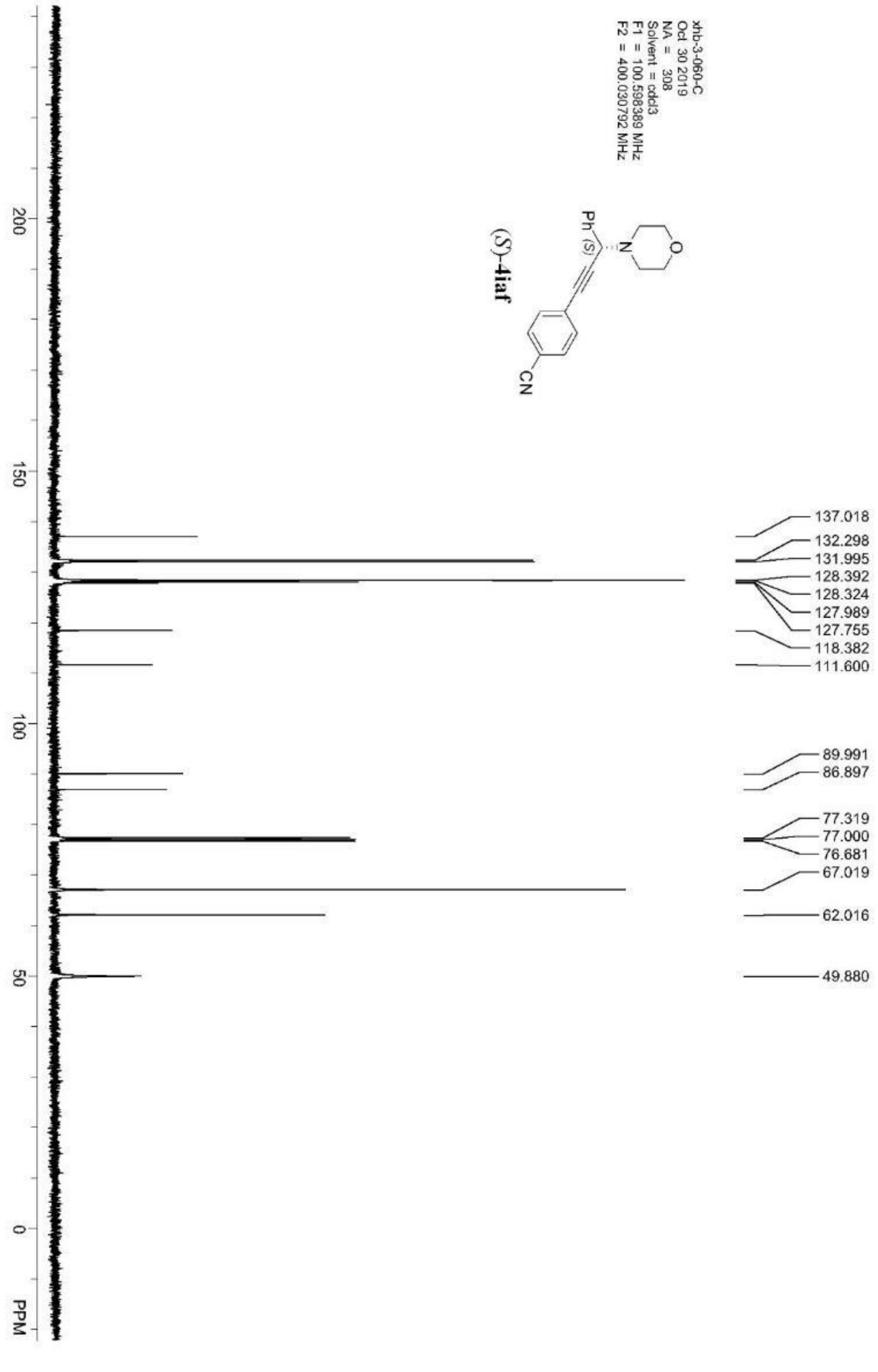

^{13}C NMR (400 MHz, $CDCl_3$) spectrum for (*S*)-4iaf

Empower 3

Injection Summary Report

SAMPLE INFORMATION

Sample Name:	xhb-3-060-od-h-100-1-1-214	Acquired By:	System
Sample Type:	Unknown	Sample Set Name:	
Vial:	1	Acq. Method Set:	HPLC
Injection #:	2	Processing Method:	LCPQ
Injection Volume:	2.00 ul	Channel Name:	W2489 ChA
Run Time:	40.0 Minutes	Proc. Chnl. Descr.:	W2489 ChA 214nm
Date Acquired:	11/3/2019 2:49:14 AM CST		
Date Processed:	11/3/2019 3:27:28 AM CST		

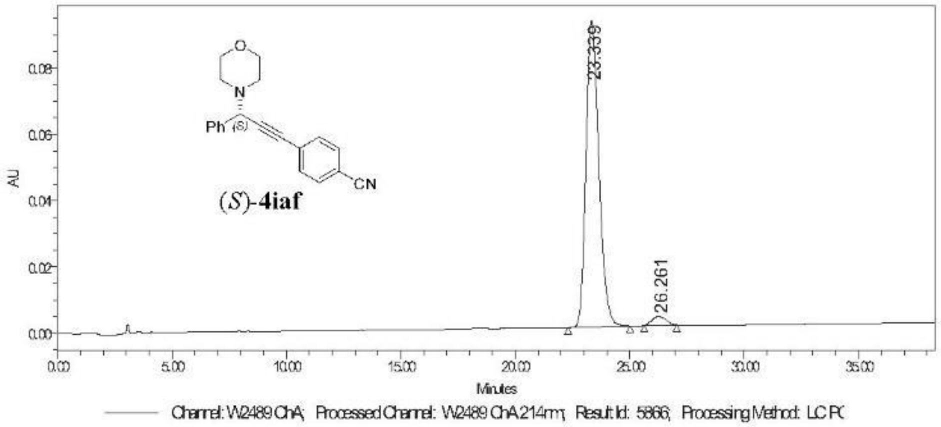

Processed Channel Descr.: W2489 ChA 214nm

	Processed Channel Descr.	RT	Area	% Area	Height
1	W2489 ChA 214nm	23.339	3608326	96.98	92262
2	W2489 ChA 214nm	26.261	112434	3.02	2720

Reported by User: System
Report Method: Injection Summary Report
Report Method ID: 1639 1639
Page: 1 of 1

Project Name: HPLC
Date Printed:
11/3/2019
4:20:06 AM PRC

HPLC spectrum for (*S*)-4iaf

Empower 3 SOFTWARE

Injection Summary Report

SAMPLE INFORMATION

Sample Name:	xhb-3-031-od-h-100-1-1-214	Acquired By:	System
Sample Type:	Unknown	Sample Set Name:	
Vial:	1	Acq. Method Set:	HPLC
Injection #:	3	Processing Method:	LCPQ
Injection Volume:	5.00 ul	Channel Name:	W2489 ChA
Run Time:	40.0 Minutes	Proc. Chnl. Descr.:	W2489 ChA 214nm
Date Acquired:	11/3/2019 3:28:37 AM CST		
Date Processed:	11/3/2019 4:19:47 AM CST		

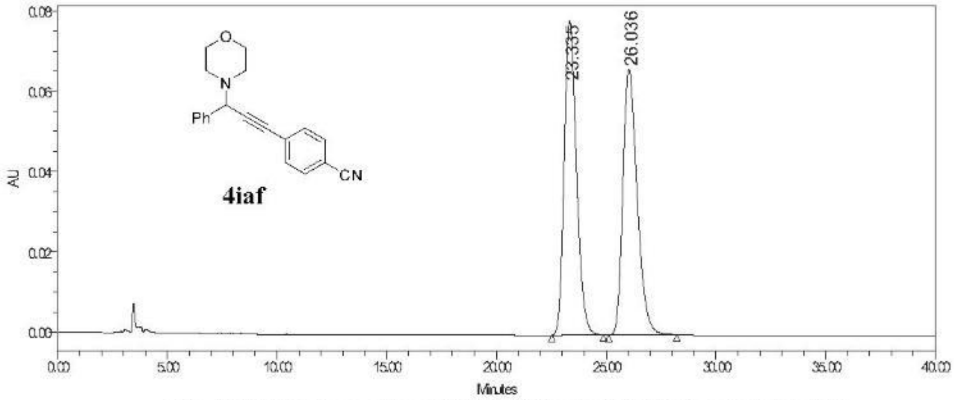

Channel: W2489 ChA; Processed Channel: W2489 ChA 214nm; Result Id: 5870; Processing Method: LC PC

Processed Channel Descr.: W2489 ChA 214nm

	Processed Channel Descr.	RT	Area	%Area	Height
1	W2489 ChA 214nm	23.335	3028384	50.37	78080
2	W2489 ChA 214nm	26.036	2983859	49.63	66040

Reported by User: System
Report Method: Injection Summary Report
Report Method ID: 1639 1639
Page: 1 of 1

Project Name: HPLC
Date Printed:
11/3/2019
4:20:37 AM PRC

HPLC spectrum for (±)-4iaf

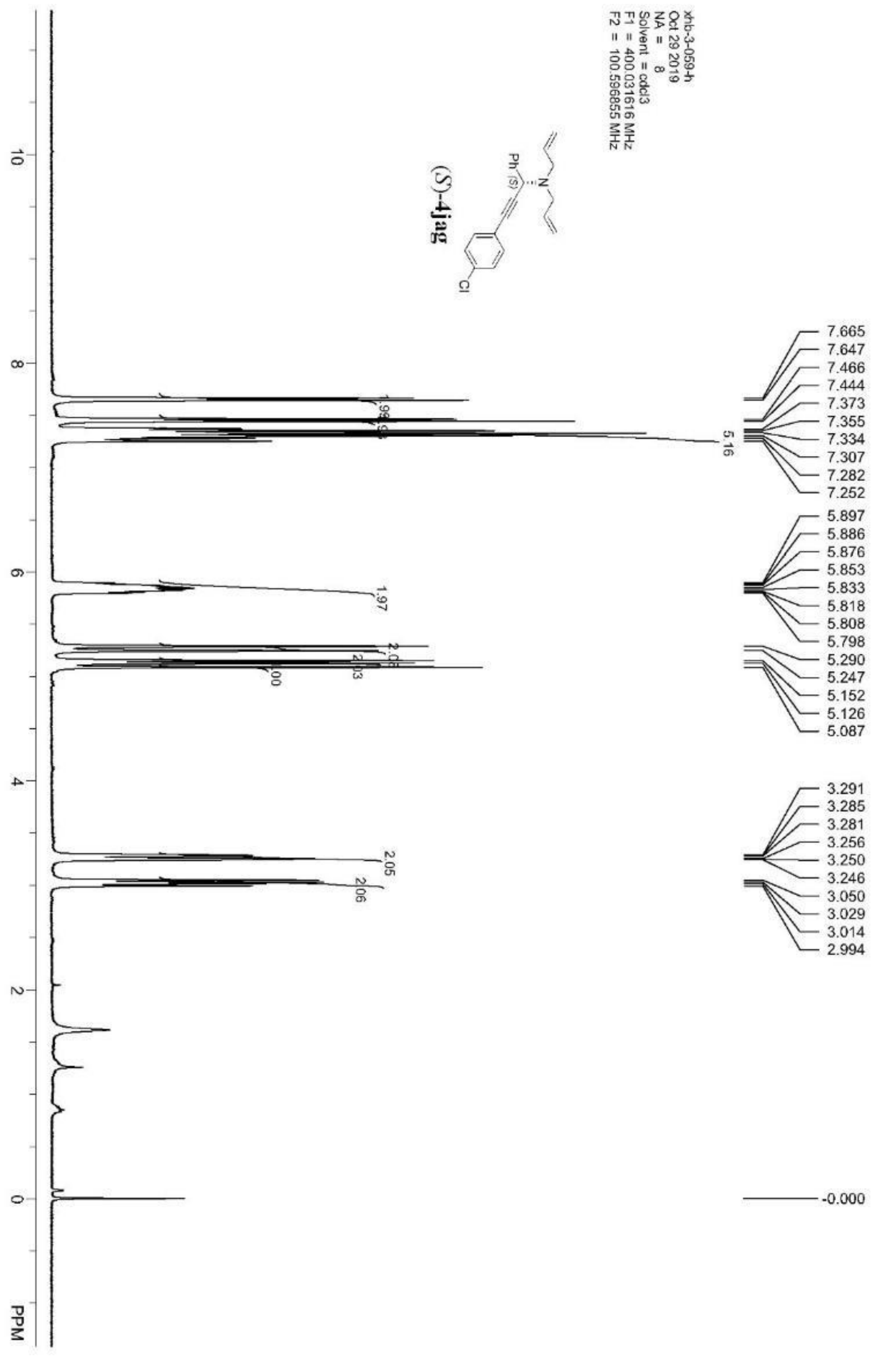

^{1}H NMR (400 MHz, CDCl3) spectrum for (*S*)**-4jag**

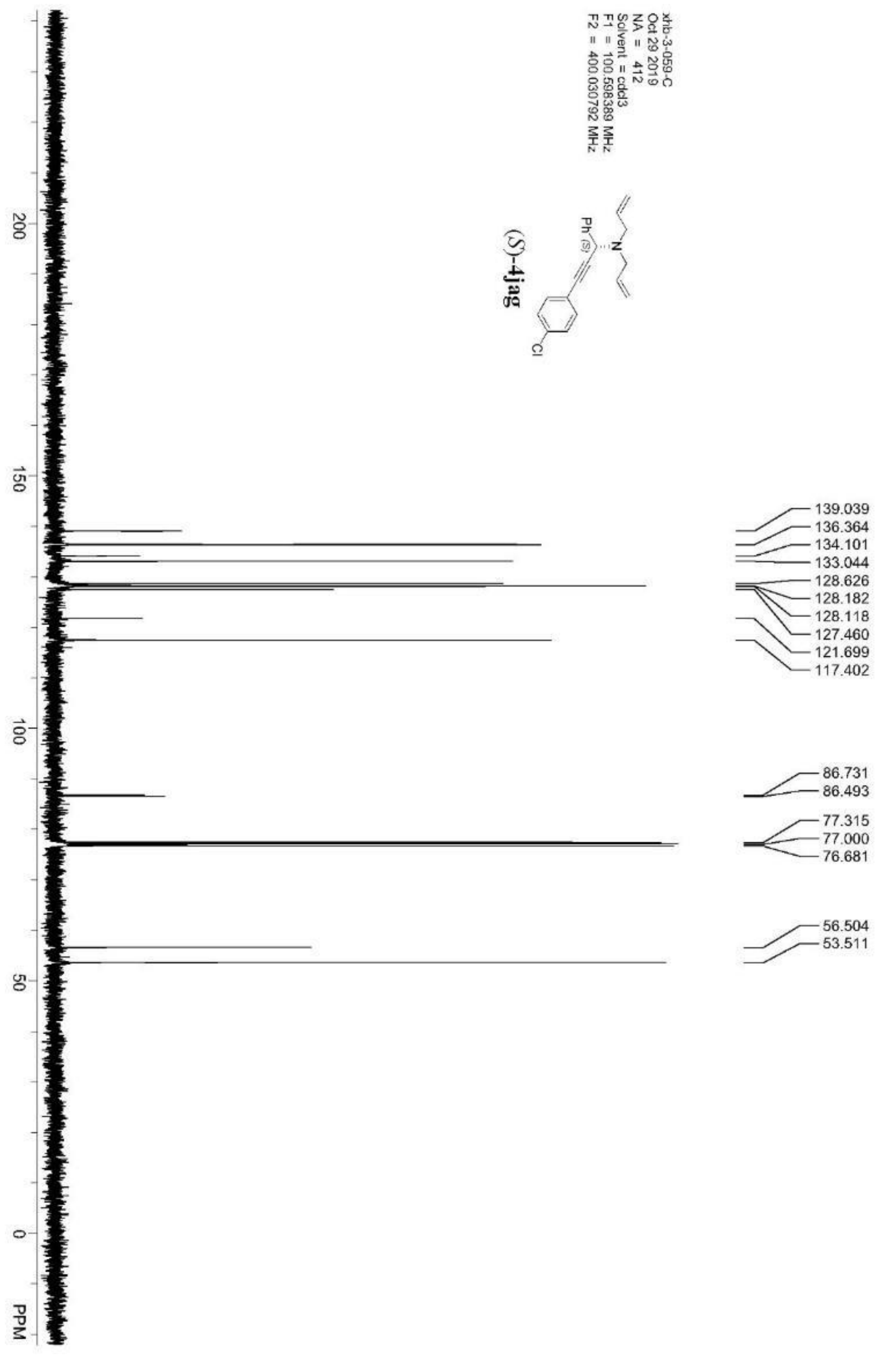

^{13}C NMR (400 MHz, CDCl$_3$) spectrum for (*S*)-4jag

Empower 3 SOFTWARE

Injection Summary Report

SAMPLE INFORMATION

Sample Name:	xhb-3-059-odh-100-0-0.7-214	Acquired By:	System
Sample Type:	Unknown	Sample Set Name:	
Vial:	1	Acq. Method Set:	HPLC
Injection #:	3	Processing Method:	LC PQ
Injection Volume:	2.50 ul	Channel Name:	W2489 ChA
Run Time:	30.0 Minutes	Proc. Chnl. Descr.:	W2489 ChA 214nm
Date Acquired:	10/30/2019 12:58:05 PM CST		
Date Processed:	10/30/2019 1:17:43 PM CST		

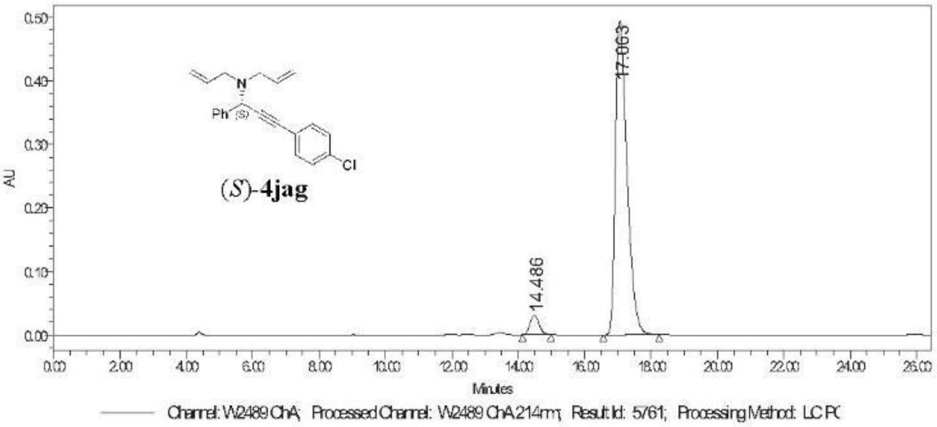

Processed Channel Descr.: W2489 ChA 214nm

	Processed Channel Descr.	RT	Area	%Area	Height
1	W2489 ChA 214nm	14.486	566336	4.54	30333
2	W2489 ChA 214nm	17.063	11895223	95.46	494251

Reported by User: System
Report Method: Injection Summary Report
Report Method ID: 1639 1639
Page: 1 of 1

Project Name: HPLC
Date Printed:
11/3/2019
4:20:56 AM PRC

HPLC spectrum for (*S*)-4jag

Empower 3 SOFTWARE

Injection Summary Report

SAMPLE INFORMATION

Sample Name:	xhb-3-036-od-h-100-0-0.7-214	Acquired By:	System
Sample Type:	Unknown	Sample Set Name:	
Vial:	1	Acq. Method Set:	HPLC
Injection #:	4	Processing Method:	LCPQ
Injection Volume:	5.00 ul	Channel Name:	W2489 ChA
Run Time:	30.0 Minutes	Proc. Chnl. Descr.:	W2489 ChA 214nm
Date Acquired:	10/30/2019 1:25:22 PM CST		
Date Processed:	10/30/2019 1:50:32 PM CST		

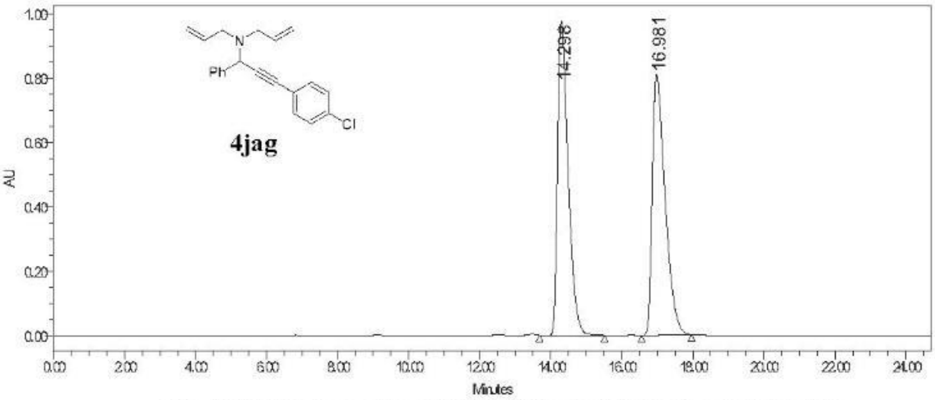

Processed Channel Descr.: W2489 ChA 214nm

	Processed Channel Descr.	RT	Area	%Area	Height
1	W2489 ChA 214nm	14.298	20122686	49.81	977791
2	W2489 ChA 214nm	16.981	20275072	50.19	811496

Reported by User: System
Report Method: Injection Summary Report
Report Method ID: 1639 1639
Page: 1 of 1

Project Name: HPLC
Date Printed:
11/3/2019
4:21:23 AM PRC

HPLC spectrum for (±)-4jag

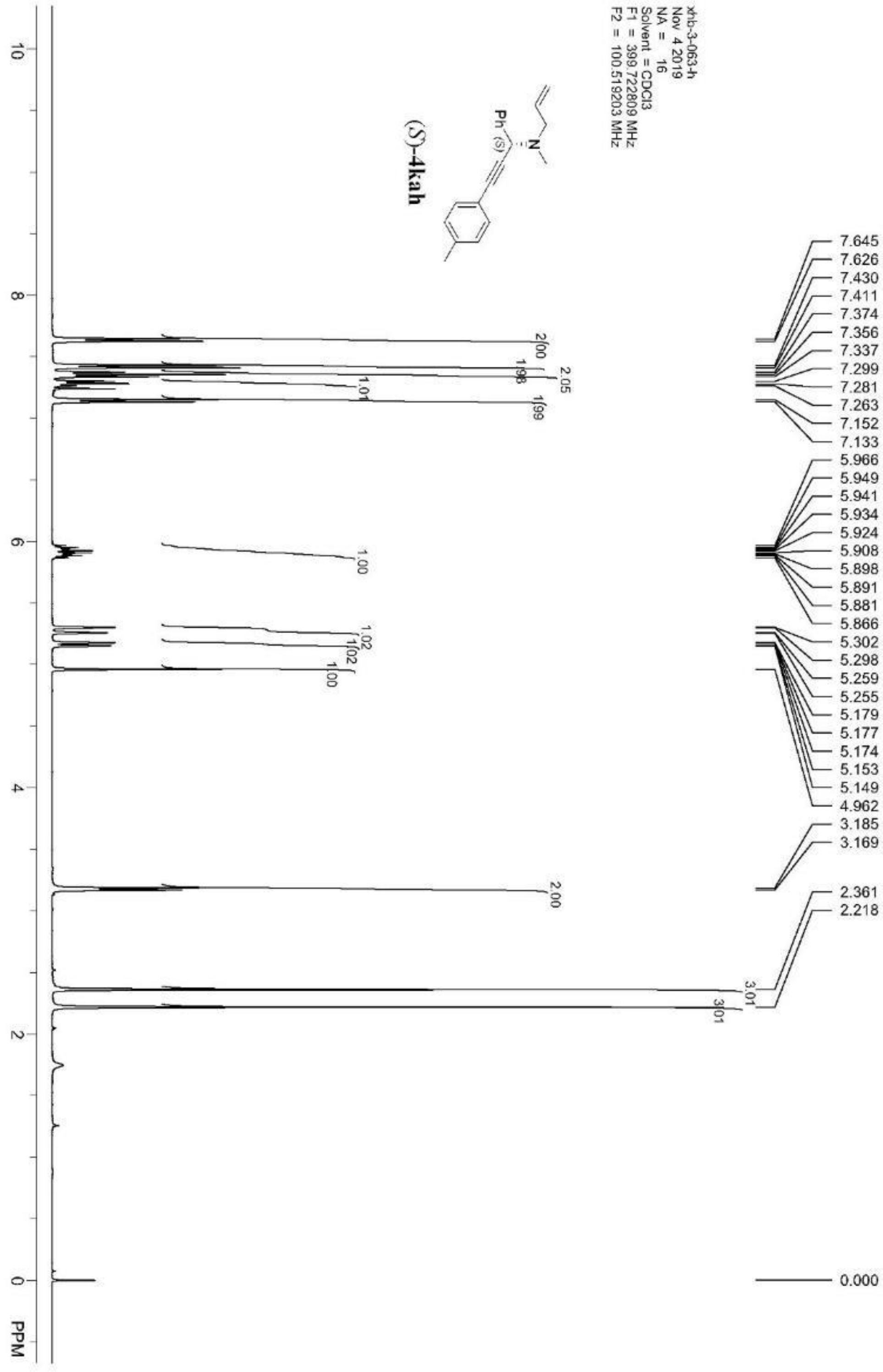

^{1}H NMR (400 MHz, CDCl3) spectrum for (*S*)-4kah

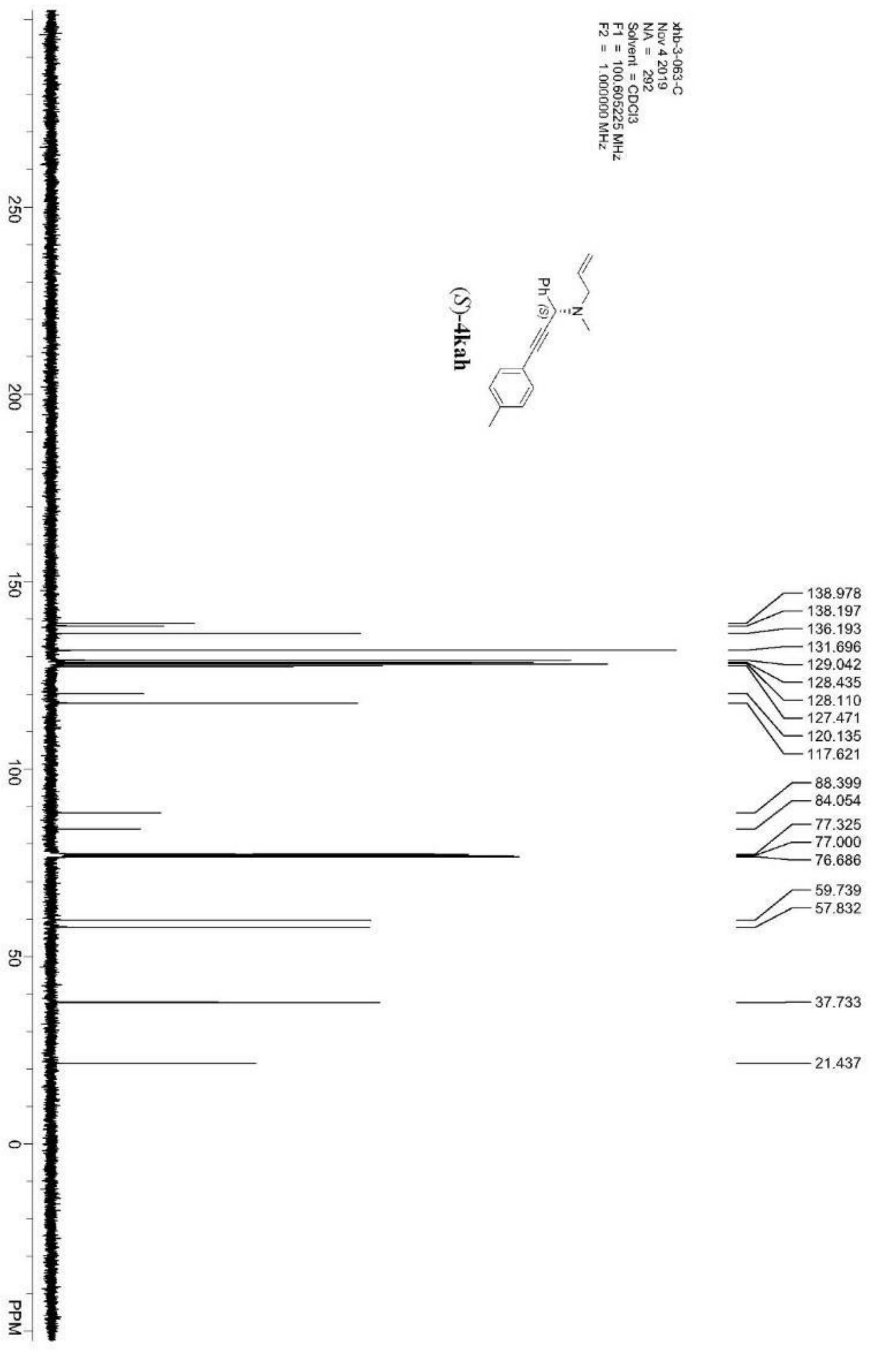

^{13}C NMR (400 MHz, CDCl$_3$) spectrum for (*S*)-4kah

Empower 3 SOFTWARE

Injection Summary Report

SAMPLE INFORMATION

Sample Name:	xhb-3-063-od-h-100-0-0.7-214	Acquired By:	System
Sample Type:	Unknown	Sample Set Name:	
Vial:	1	Acq. Method Set:	HPLC
Injection #:	2	Processing Method:	LCPQ
Injection Volume:	2.50 ul	Channel Name:	W2489 ChA
Run Time:	30.0 Minutes	Proc. Chnl. Descr.:	W2489 ChA 214nm
Date Acquired:	11/5/2019 5:45:06 AM CST		
Date Processed:	11/6/2019 11:16:04 AM CST		

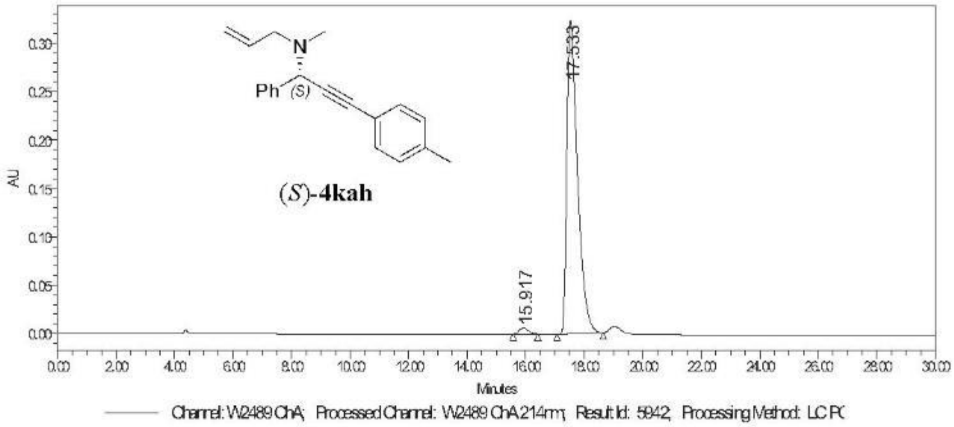

Processed Channel Descr.: W2489 ChA 214nm

	Processed Channel Descr.	RT	Area	%Area	Height
1	W2489 ChA 214nm	15.917	130283	1.57	6279
2	W2489 ChA 214nm	17.533	8145133	98.43	323128

Reported by User: System
Report Method: Injection Summary Report
Report Method ID: 1639 1639
Page: 1 of 1

Project Name: HPLC
Date Printed:
11/6/2019
11:16:58 AM PRC

HPLC spectrum for (*S*)-4kah

Empower™ 3 SOFTWARE

Injection Summary Report

SAMPLE INFORMATION

Sample Name:	xhb-3-040-od-h-100-0-0.7-214	Acquired By:	System
Sample Type:	Unknown	Sample Set Name:	
Vial:	1	Acq. Method Set:	HPLC
Injection #:	3	Processing Method:	LC PQ
Injection Volume:	5.00 ul	Channel Name:	W2489 ChA
Run Time:	30.0 Minutes	Proc. Chnl. Descr.:	W2489 ChA 214nm
Date Acquired:	11/5/2019 6:32:48 AM CST		
Date Processed:	11/6/2019 11:16:37 AM CST		

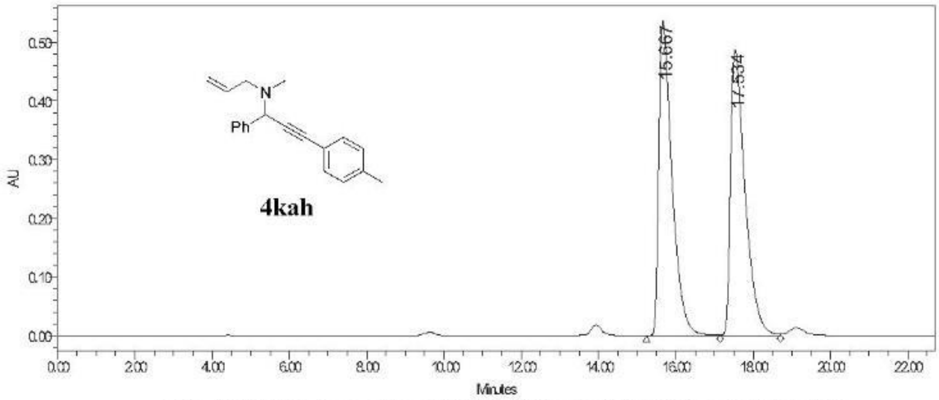

Processed Channel Descr.: W2489 ChA 214nm

	Processed Channel Descr.	RT	Area	%Area	Height
1	W2489 ChA 214nm	15.667	12620323	49.87	536326
2	W2489 ChA 214nm	17.534	12683997	50.13	487318

Reported by User: System
Report Method: Injection Summary Report
Report Method ID: 1639 1639
Page: 1 of 1

Project Name: HPLC
Date Printed:
11/6/2019
11:17:29 AM PRC

HPLC spectrum for (±)-4kah

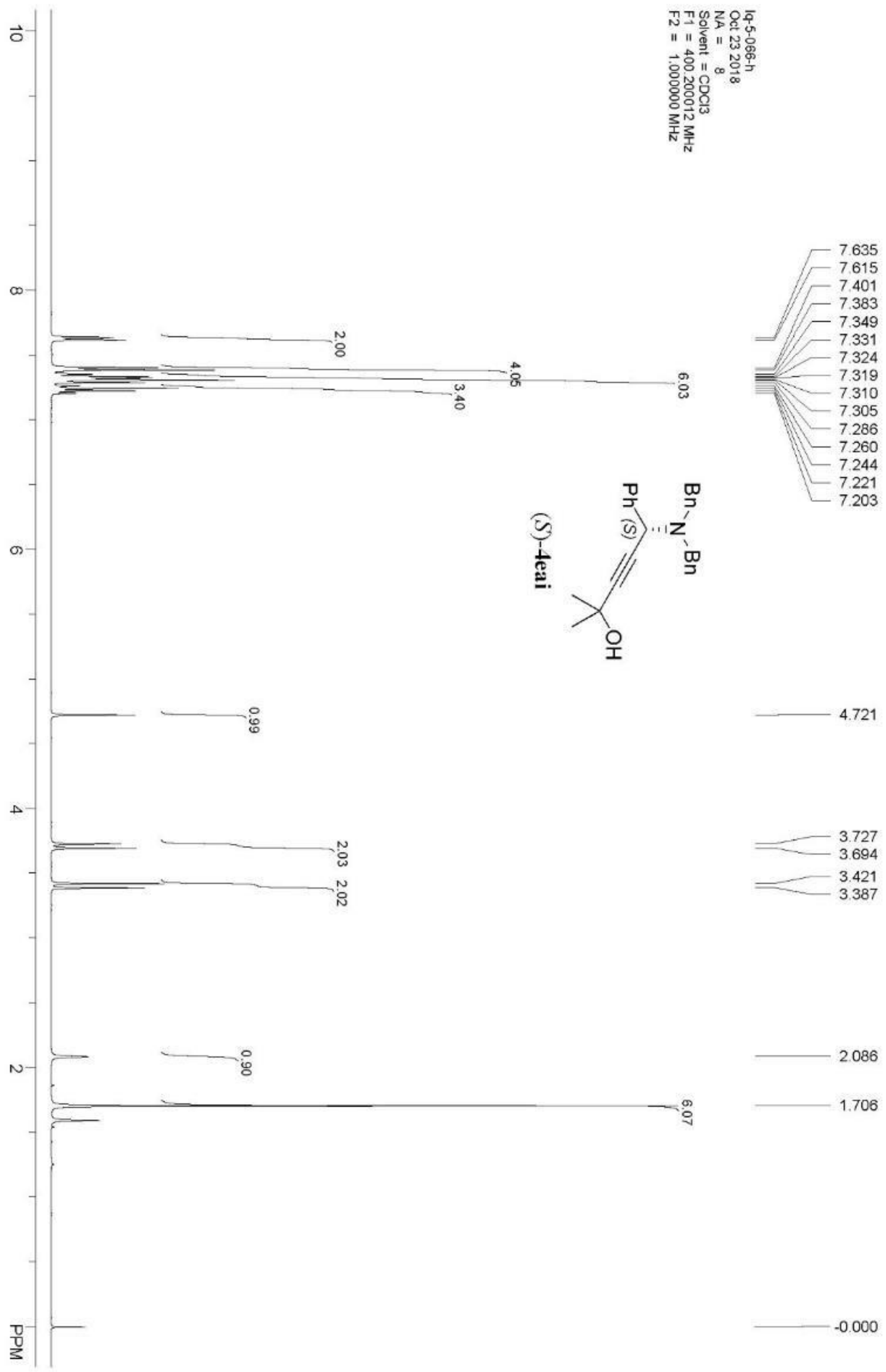

^{1}H NMR (400 MHz, CDCl3) spectrum for (*S*)-4eai

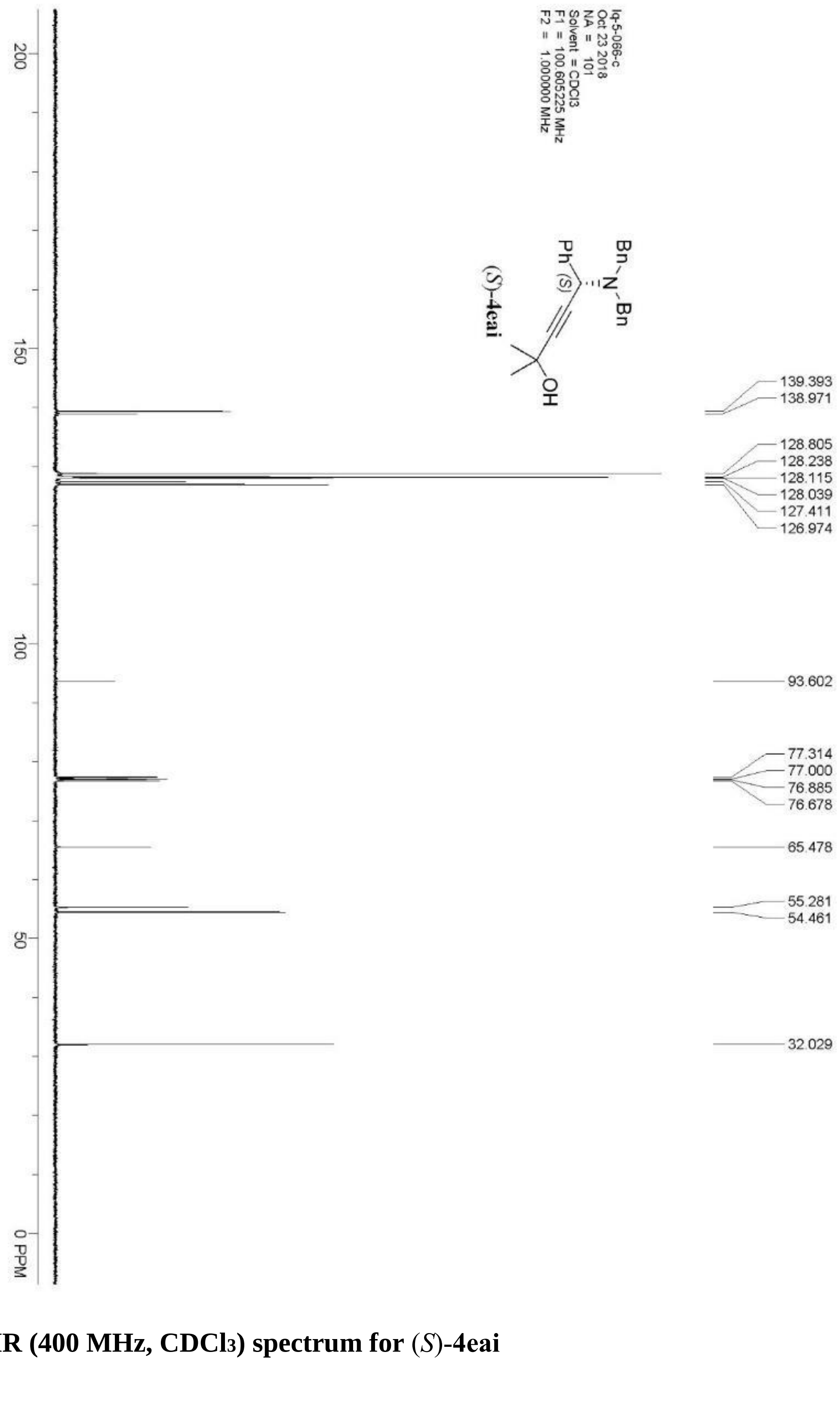

^{13}C NMR (400 MHz, CDCl$_3$) spectrum for (*S*)-4eai

1q-5-066

实验时间：2018-10-23, 15:33:21　　　　报告时间：2018-10-23, 16:07:04
谱图文件:D:\data\slf\lq\2018-10-23-2\lq-5-066-od-h-100+1-1.2-214.org

实验内容简介：
OD-H　100+1
214nm　1.2ml/min

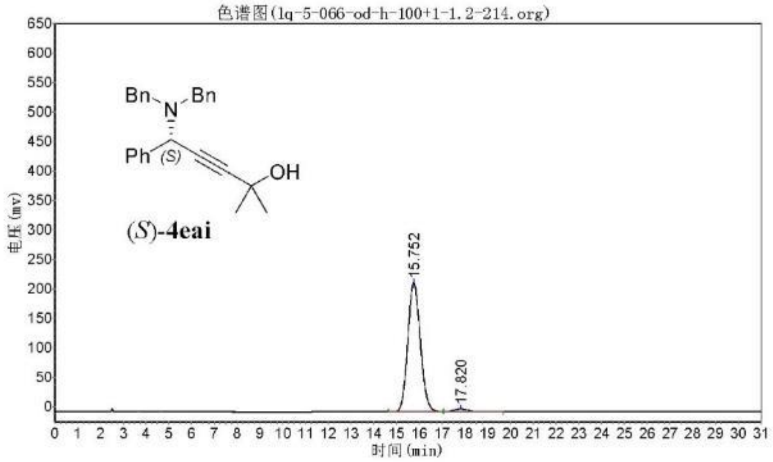

分析结果表

峰号	峰名	保留时间	峰高	峰面积	含量
1		15.752	218754.781	8386064.000	97.4258
2		17.820	4628.918	221578.141	2.5742
**总计**			223383.700	8607642.141	100.0000

HPLC spectrum for (*S*)-4eai

xhb-1-086

实验时间：2018-10-23, 15:01:00　　　　　　　　　　　　报告时间：2018-10-23, 15:35:09
谱图文件:D:\data\slf\lq\2018-10-23-2\xhb-1-086-od-h-100+1-1.2-214.org

实验内容简介：
OD-H　100+1
214nm　1.2ml/min

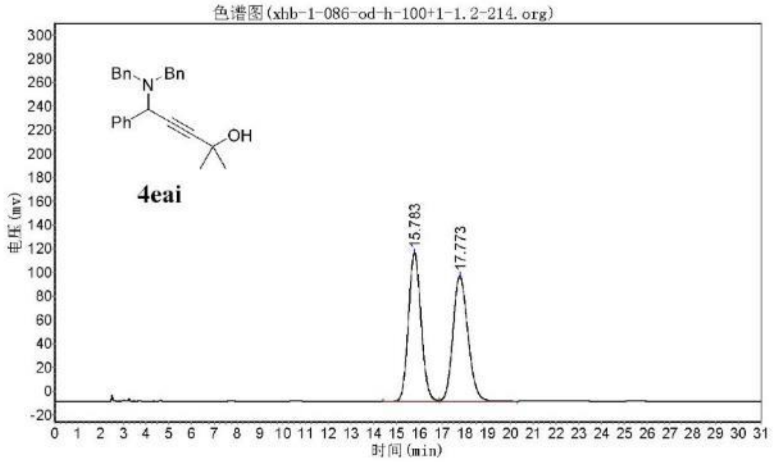

分析结果表

峰号	峰名	保留时间	峰高	峰面积	含量
1		15.783	125317.836	4817742.500	49.8258
2		17.773	105896.242	4851421.500	50.1742
总计			231214.078	9669164.000	100.0000

HPLC spectrum for (±)-4eai

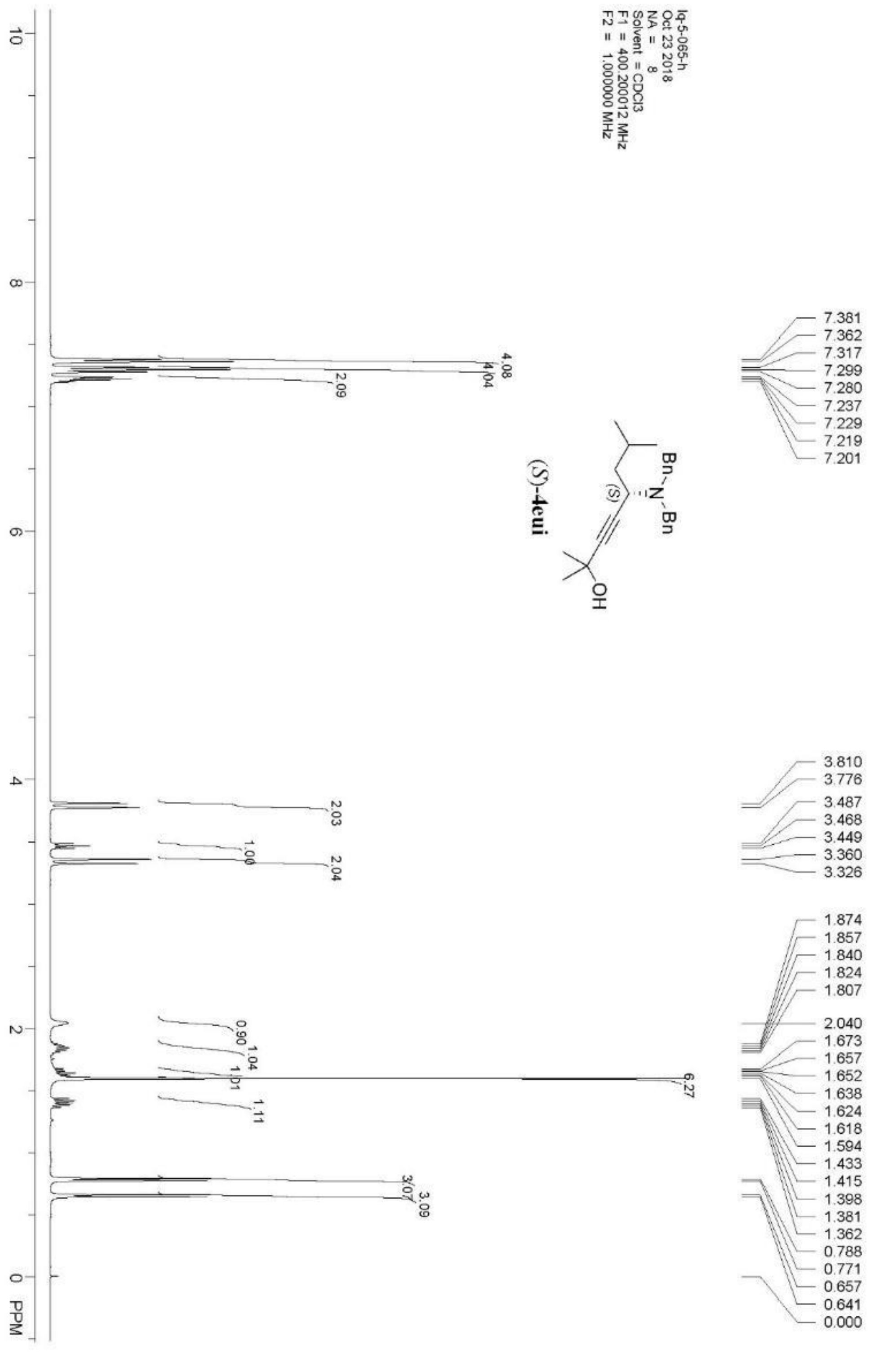

^{1}H NMR (400 MHz, CDCl3) spectrum for (*S*)**-4eui**

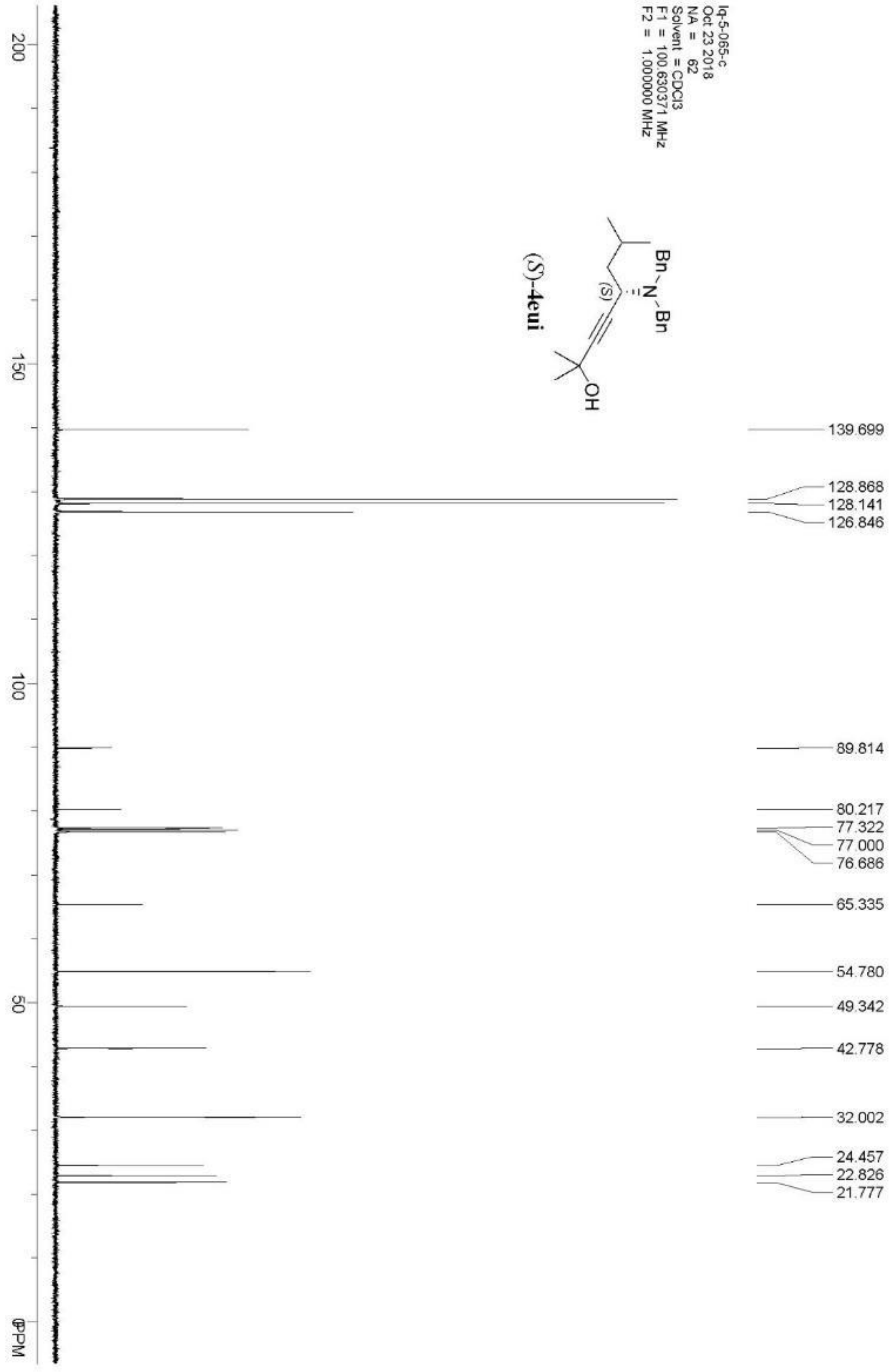

^{13}C NMR (400 MHz, CDCl$_3$) spectrum for (*S*)-4eui

1q-5-065

实验时间：2018-10-23, 13:24:32 报告时间：2018-10-23, 13:30:57
谱图文件:D:\data\slf\lq\2018-10-23\lq-5-065-ad-h-98+2-1-214-2.mdy

实验内容简介：
AD-H 98:2
214nm 1.0ml/min

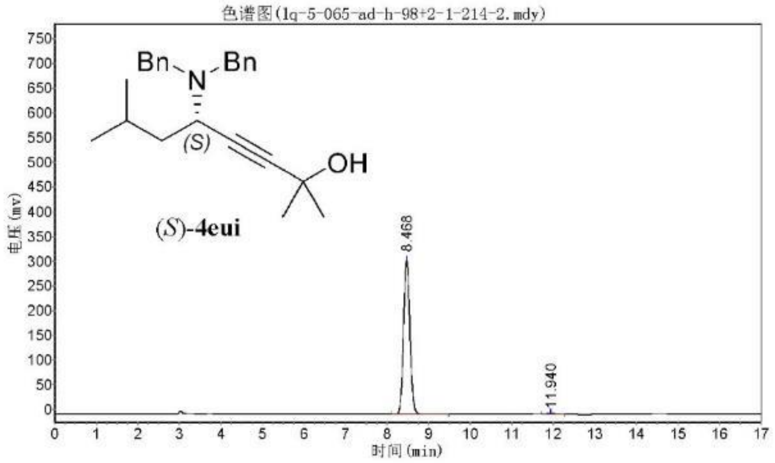

分析结果表

峰号	峰名	保留时间	峰高	峰面积	含量
1		8.468	310649.719	3291174.500	98.7207
2		11.940	2706.676	42648.496	1.2793
**总计**			313356.395	3333822.996	100.0000

HPLC spectrum for (*S*)-4eui

xhb-1-087

实验时间：2018-10-23, 11:18:54　　　　　　　　　　　　报告时间：2018-10-23, 13:29:08
谱图文件:D:\data\slf\lq\2018-10-23\xhb-1-087-ad-h-98+2-1-214-2.org

实验内容简介：
AD-H　98:2
214nm　1.0ml/min

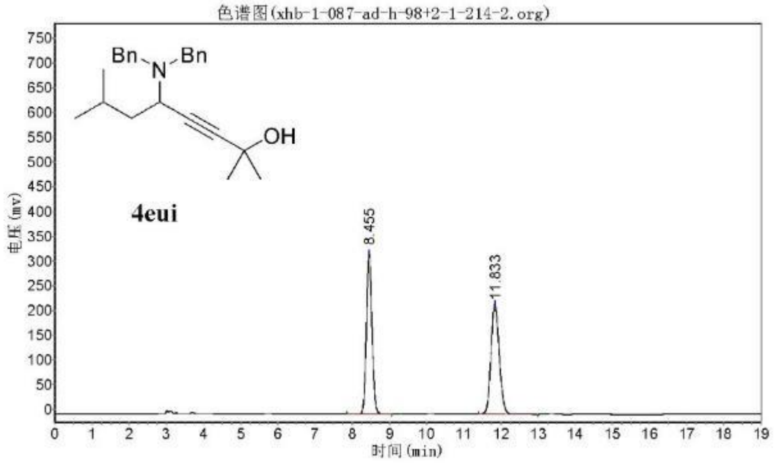

分析结果表

峰号	峰名	保留时间	峰高	峰面积	含量
1		8.455	323698.281	3419639.750	49.7918
2		11.833	221653.875	3448241.750	50.2082
总计			545352.156	6867881.500	100.0000

HPLC spectrum for (±)-4eui

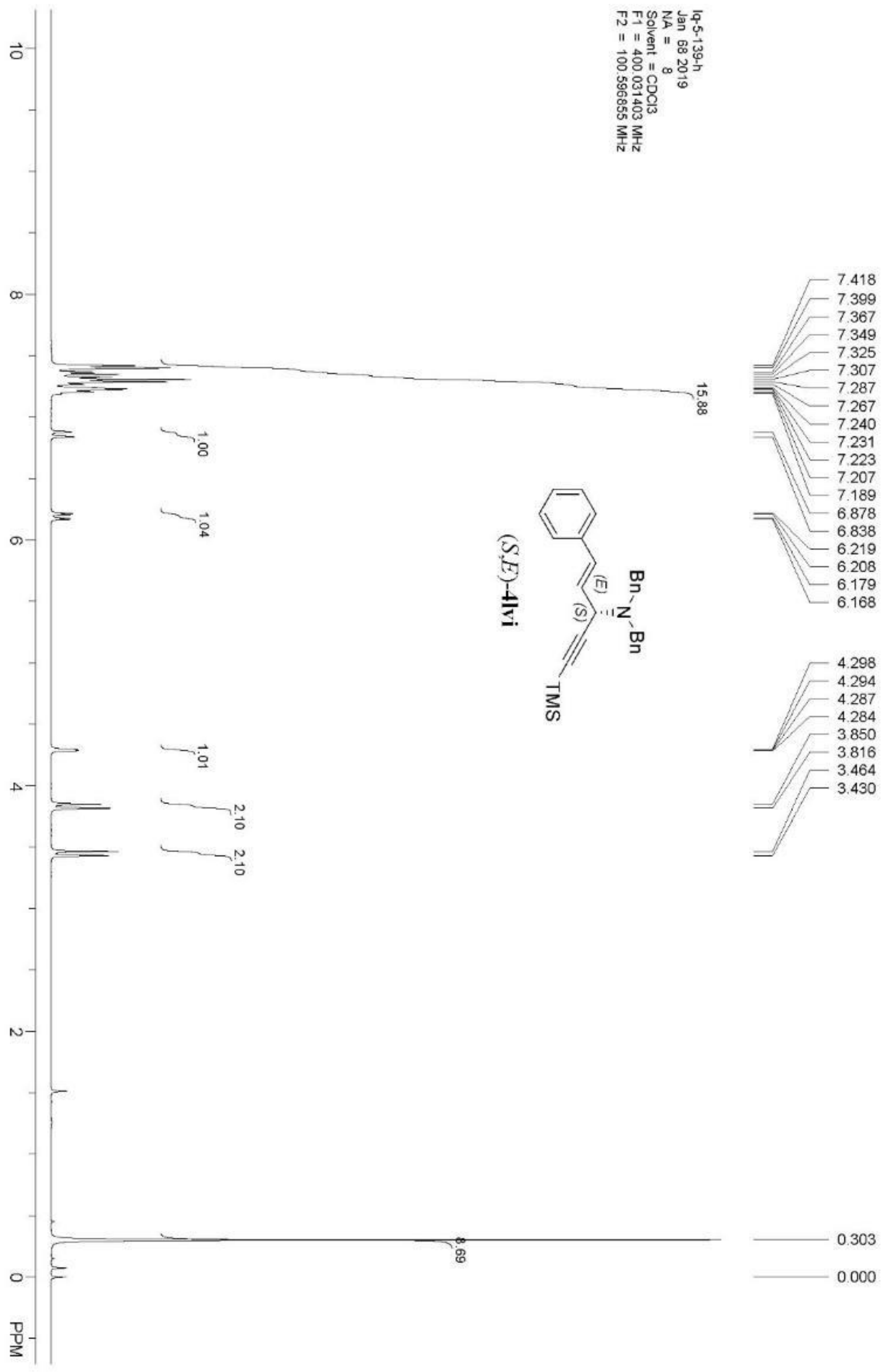

^{1}H NMR (400 MHz, CDCl3) spectrum for (*S*,*E*)-**4lvi**

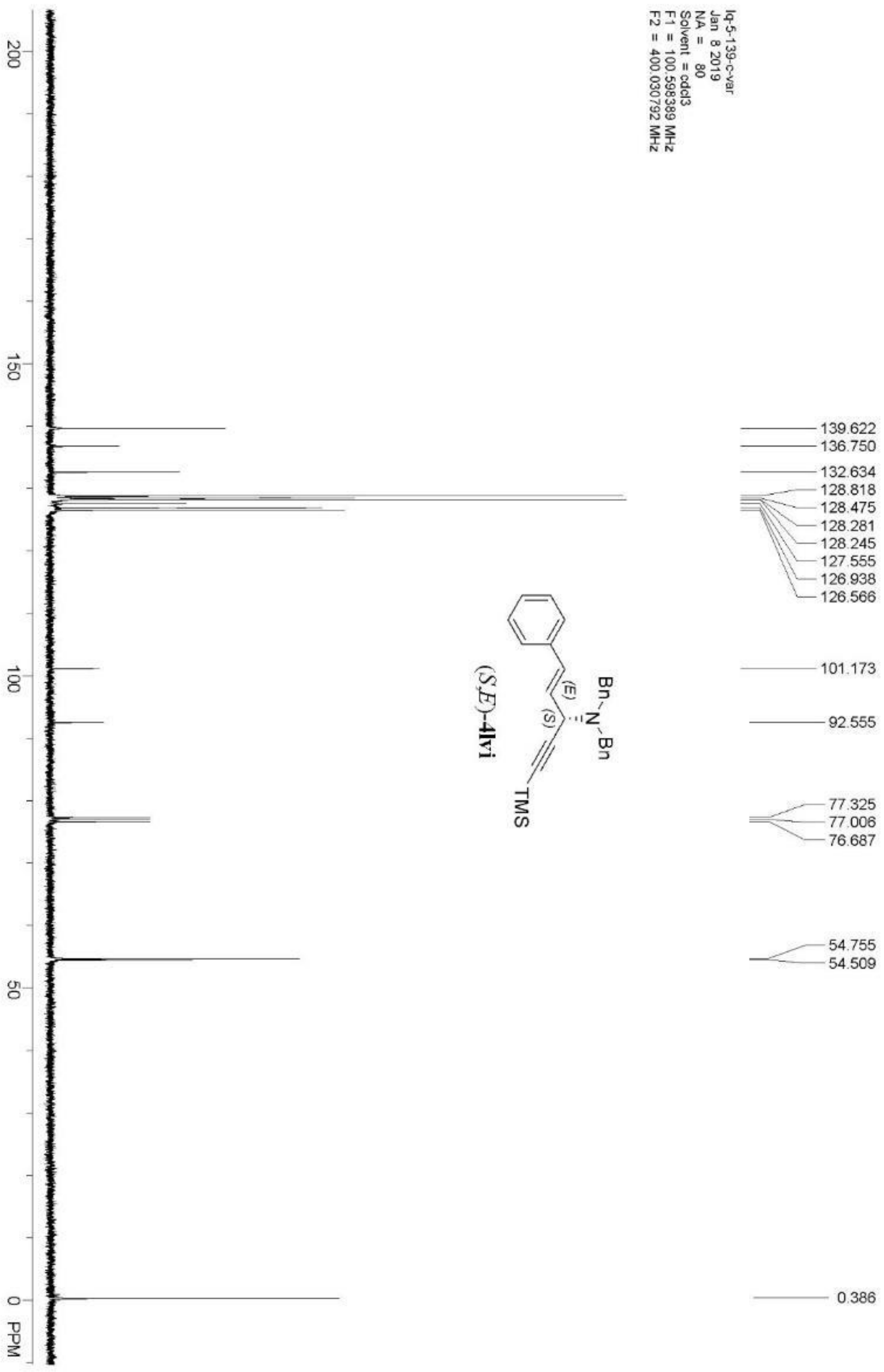

^{13}C NMR (400 MHz, CDCl$_3$) spectrum for (*S*,*E*)-4lvi

Empower 3 SOFTWARE

Injection Summary Report

SAMPLE INFORMATION

Sample Name:	lq-5-139-od-h-100-0-0.2-214	Acquired By:	System
Sample Type:	Unknown	Sample Set Name:	
Vial:	1	Acq. Method Set:	HPLC
Injection #:	2	Processing Method:	Default
Injection Volume:	5.00 ul	Channel Name:	W2489 ChA
Run Time:	120.0 Minutes	Proc. Chnl. Descr.:	W2489 ChA 214nm
Date Acquired:	1/6/2019 11:54:03 AM CST		
Date Processed:	1/6/2019 1:23:15 PM CST		

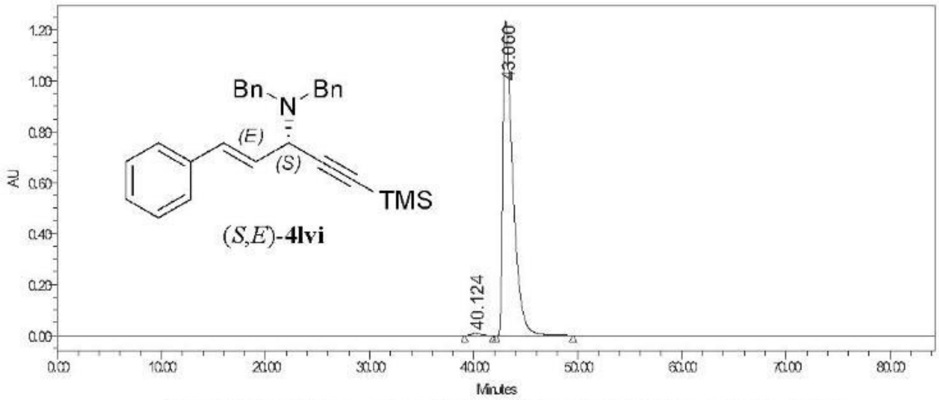

Channel: W2489 ChA; Processed Channel: W2489 ChA 214nm; Result Id: 2247; Processing Method: Defaul

Processed Channel Descr.: W2489 ChA 214nm

	Processed Channel Descr.	RT	Area	%Area	Height
1	W2489 ChA 214nm	40.124	525338	0.64	9159
2	W2489 ChA 214nm	43.060	81526652	99.36	1234201

Reported by User: System
Report Method: Injection Summary Report
Report Method ID: 1639 1639
Page: 1 of 1

Project Name: HPLC
Date Printed:
1/6/2019
3:18:53 PM PRC

HPLC spectrum for (*S*,*E*)-4lvi

Empower 3 SOFTWARE

Injection Summary Report

SAMPLE INFORMATION

Sample Name:	lq-5-136-od-h-100-0-0.2-214	Acquired By:	System
Sample Type:	Unknown	Sample Set Name:	
Vial:	1	Acq. Method Set:	HPLC
Injection #:	4	Processing Method:	Default
Injection Volume:	5.00 ul	Channel Name:	W2489 ChA
Run Time:	120.0 Minutes	Proc. Chnl. Descr.:	W2489 ChA 214nm
Date Acquired:	1/6/2019 1:54:05 PM CST		
Date Processed:	1/6/2019 3:20:18 PM CST		

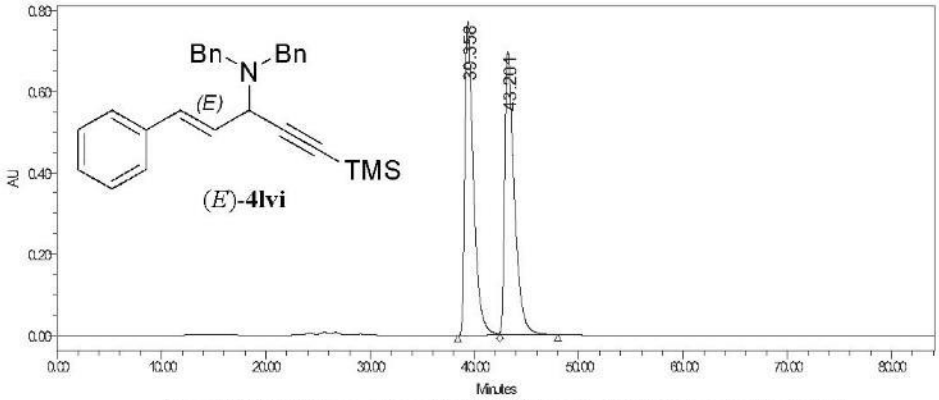

Processed Channel Descr.: W2489 ChA 214nm

	Processed Channel Descr.	RT	Area	%Area	Height
1	W2489 ChA 214nm	39.358	44183502	49.92	772961
2	W2489 ChA 214nm	43.201	44319975	50.08	697151

Reported by User: System
Report Method: Injection Summary Report
Report Method ID: 1639 1639
Page: 1 of 1

Project Name: HPLC
Date Printed:
1/6/2019
3:20:28 PM PRC

HPLC spectrum for (*E*)-4lvi

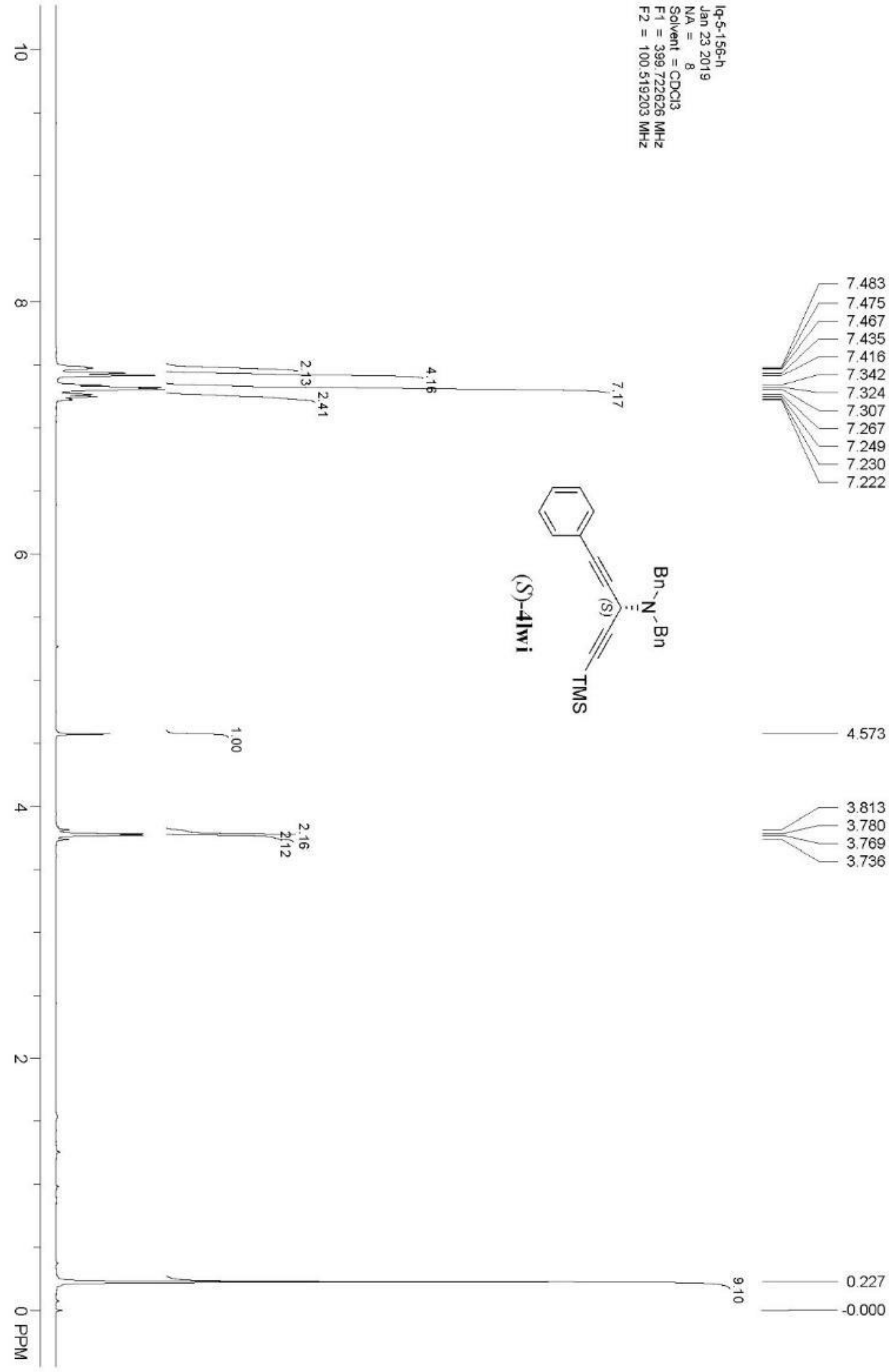

^{1}H NMR (400 MHz, CDCl3) spectrum for (*S*)**-4lwi**

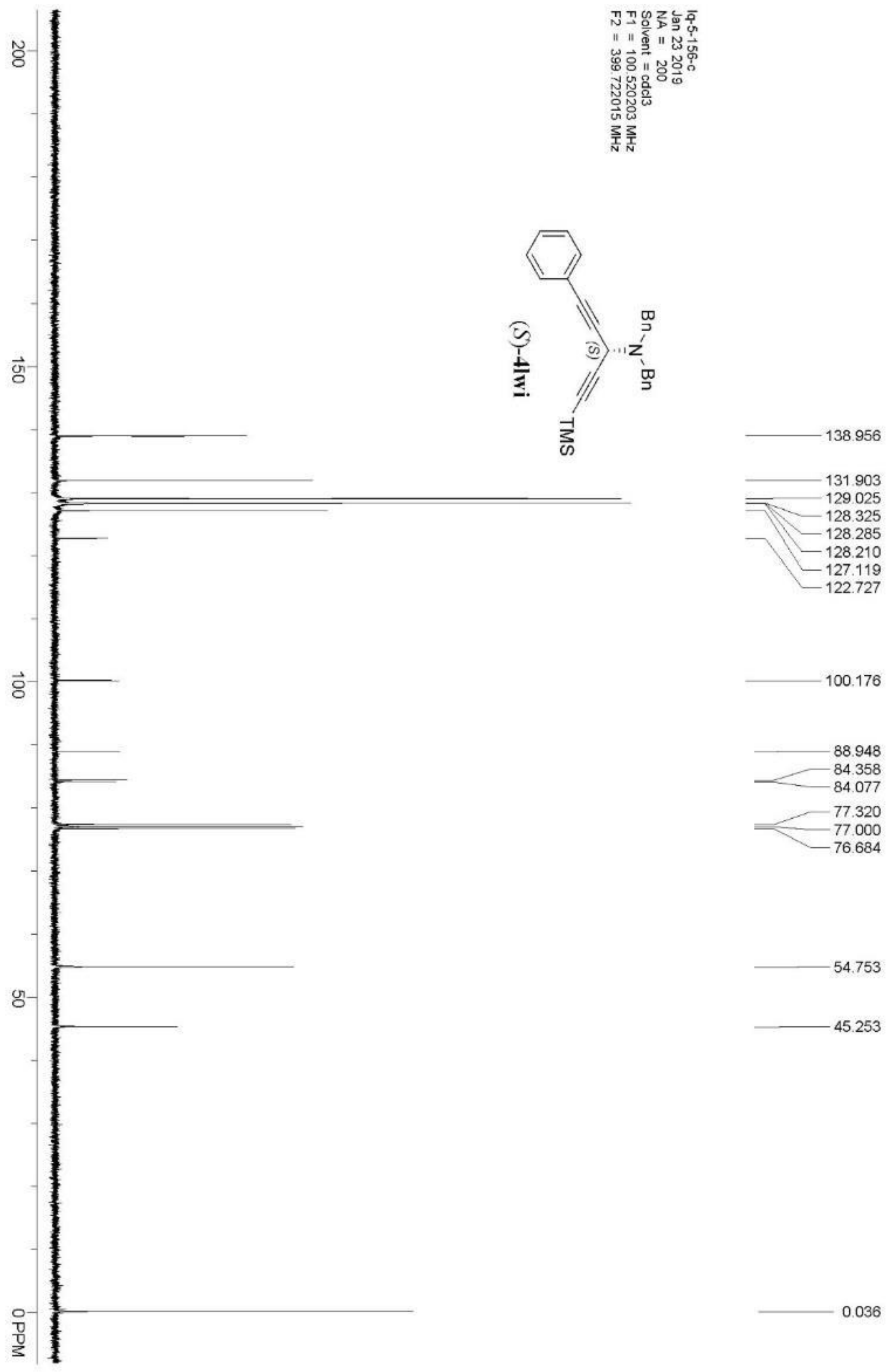

^{13}C NMR (400 MHz, CDCl3) spectrum for (*S*)-4lwi

Empower 3 SOFTWARE

Injection Summary Report

SAMPLE INFORMATION

Sample Name:	lq-5-156-od-h-100-0-0.5-214	Acquired By:	System
Sample Type:	Unknown	Sample Set Name:	
Vial:	1	Acq. Method Set:	HPLC
Injection #:	2	Processing Method:	Default
Injection Volume:	3.00 ul	Channel Name:	W2489 ChA
Run Time:	40.0 Minutes	Proc. Chnl. Descr.:	W2489 ChA 214nm
Date Acquired:	1/22/2019 1:40:03 PM CST		
Date Processed:	1/22/2019 2:15:55 PM CST		

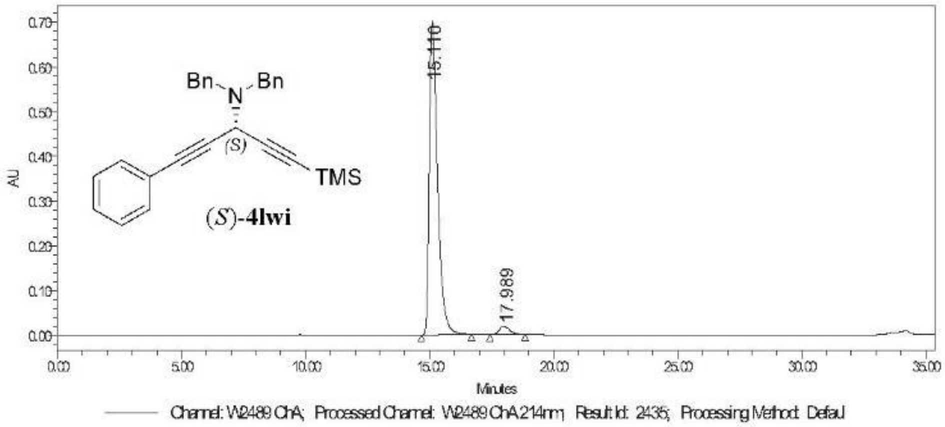

Processed Channel Descr.: W2489 ChA 214nm

	Processed Channel Descr.	RT	Area	%Area	Height
1	W2489 ChA 214nm	15.110	16216886	96.78	702211
2	W2489 ChA 214nm	17.989	539462	3.22	18601

Reported by User: System
Report Method: Injection Summary Report
Report Method ID: 1639 1639
Page: 1 of 1

Project Name: HPLC
Date Printed:
1/24/2019
3:12:14 AM PRC

HPLC spectrum for (*S*)-4lwi

Empower™ 3 SOFTWARE

Injection Summary Report

SAMPLE INFORMATION

Sample Name:	lq-5-138-od-h-100-0-0.5-214	Acquired By:	System
Sample Type:	Unknown	Sample Set Name:	
Vial:	1	Acq. Method Set:	HPLC
Injection #:	3	Processing Method:	Default
Injection Volume:	3.00 ul	Channel Name:	W2489 ChA
Run Time:	40.0 Minutes	Proc. Chnl. Descr.:	W2489 ChA 214nm
Date Acquired:	1/22/2019 2:16:25 PM CST		
Date Processed:	1/22/2019 3:00:55 PM CST		

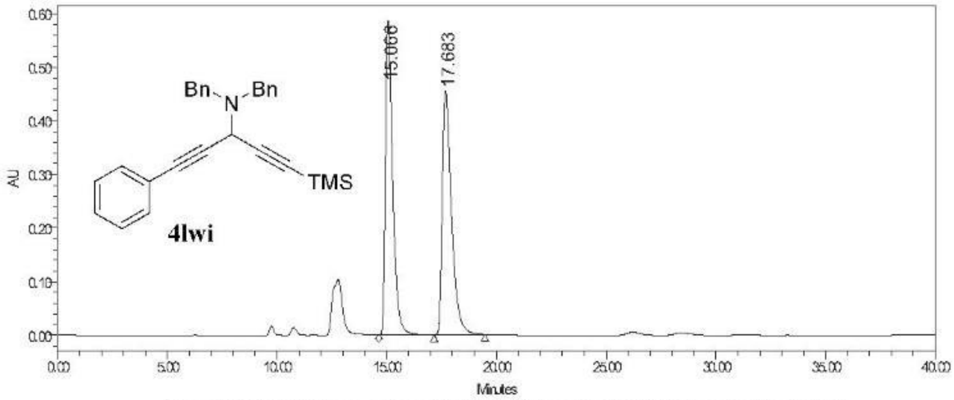

Channel: W2489 ChA; Processed Channel: W2489 ChA 214nm; Result Id: 2439; Processing Method: Defaul

Processed Channel Descr.: W2489 ChA 214nm

	Processed Channel Descr.	RT	Area	%Area	Height
1	W2489 ChA 214nm	15.066	13417513	50.19	585719
2	W2489 ChA 214nm	17.683	13313944	49.81	456367

Reported by User: System
Report Method: Injection Summary Report
Report Method ID: 1639 1639
Page: 1 of 1

Project Name: HPLC
Date Printed:
1/24/2019
6:49:13 AM PRC

HPLC spectrum for (±)-4lwi

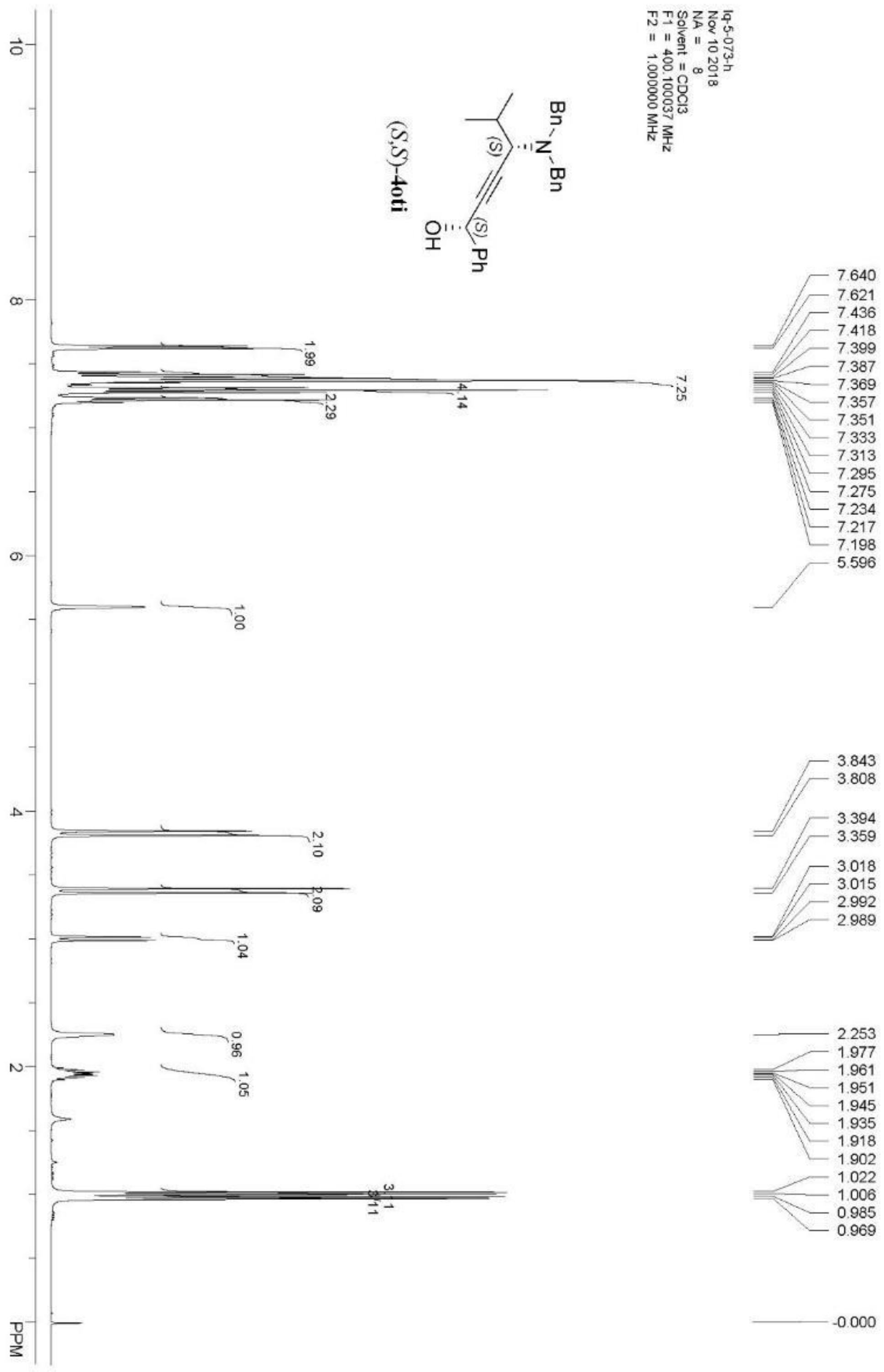

^{1}H NMR (400 MHz, CDCl3) spectrum for (*S*,*S*)**-4oti**

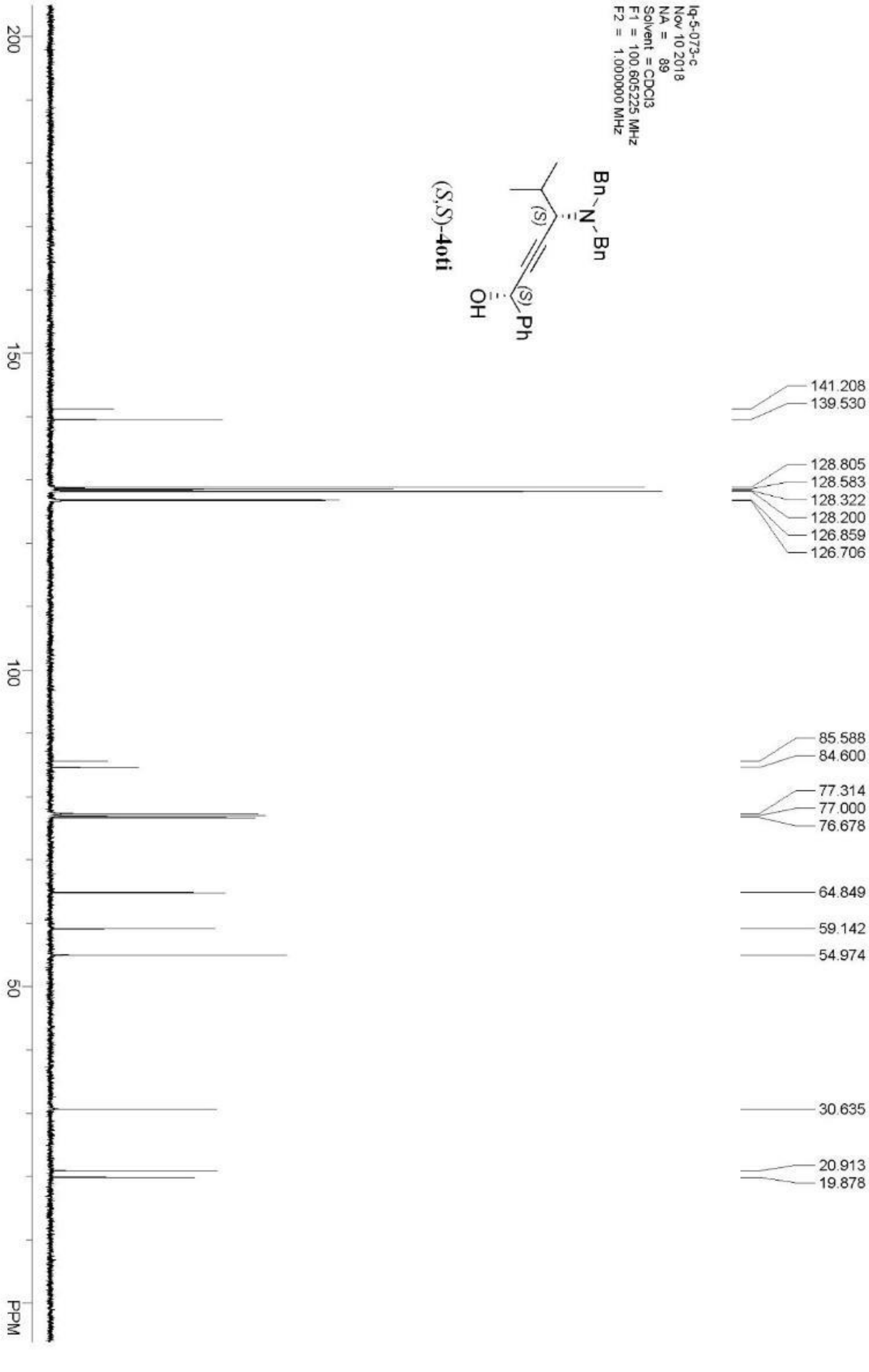

^{13}C NMR (400 MHz, CDCl$_3$) spectrum for (*S*,*S*)-4oti

1q-5-073

实验时间：2018-11-06, 23:46:45　　　　报告时间：2018-11-07, 0:32:19
谱图文件:D:\data\slf\1q\2018-11-06\1q-5-073-od-h-95-5-0.5-214-2.org

实验内容简介：
OD-H　　95+5
0.5ml/min　214nm

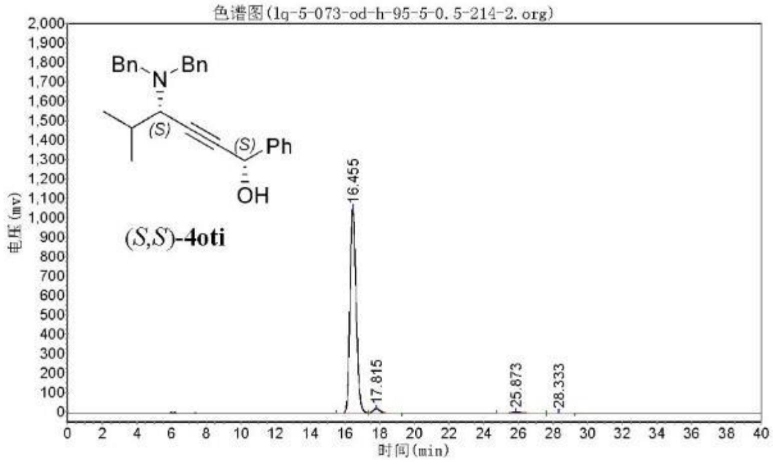

分析结果表

峰号	峰名	保留时间	峰高	峰面积	含量
1		16.455	1055444.375	26230332.000	96.5800
2		17.815	22861.410	651502.688	2.3988
3		25.873	5607.155	270427.813	0.9957
4		28.333	127.627	6899.591	0.0254
总计			1084040.568	27159162.091	100.0000

HPLC spectrum for (*S*,*S*)-4oti

xhb-1-094

实验时间：2018-11-06,20:30:16 报告时间：2018-11-06,21:27:02
谱图文件:D:\data\slf\lq\2018-11-06\xhb-1-094-od-h-95-5-0.5-214.org

实验内容简介：
OD-H 95+5
0.5ml/min 214nm

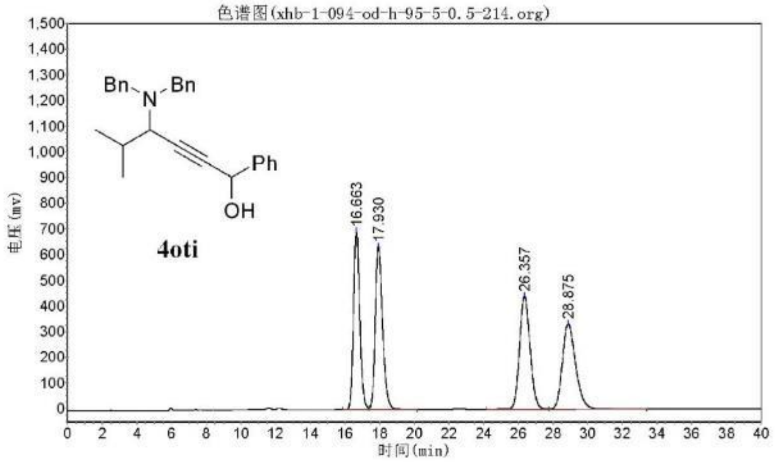

分析结果表

峰号	峰名	保留时间	峰高	峰面积	含量
1		16.663	692755.500	17448750.000	24.1373
2		17.930	632441.375	18556568.000	25.6698
3		26.357	440318.656	18571336.000	25.6902
4		28.875	332774.344	17712846.000	24.5027
**总计**			2098289.875	72289500.000	100.0000

HPLC spectrum for (±)-4oti

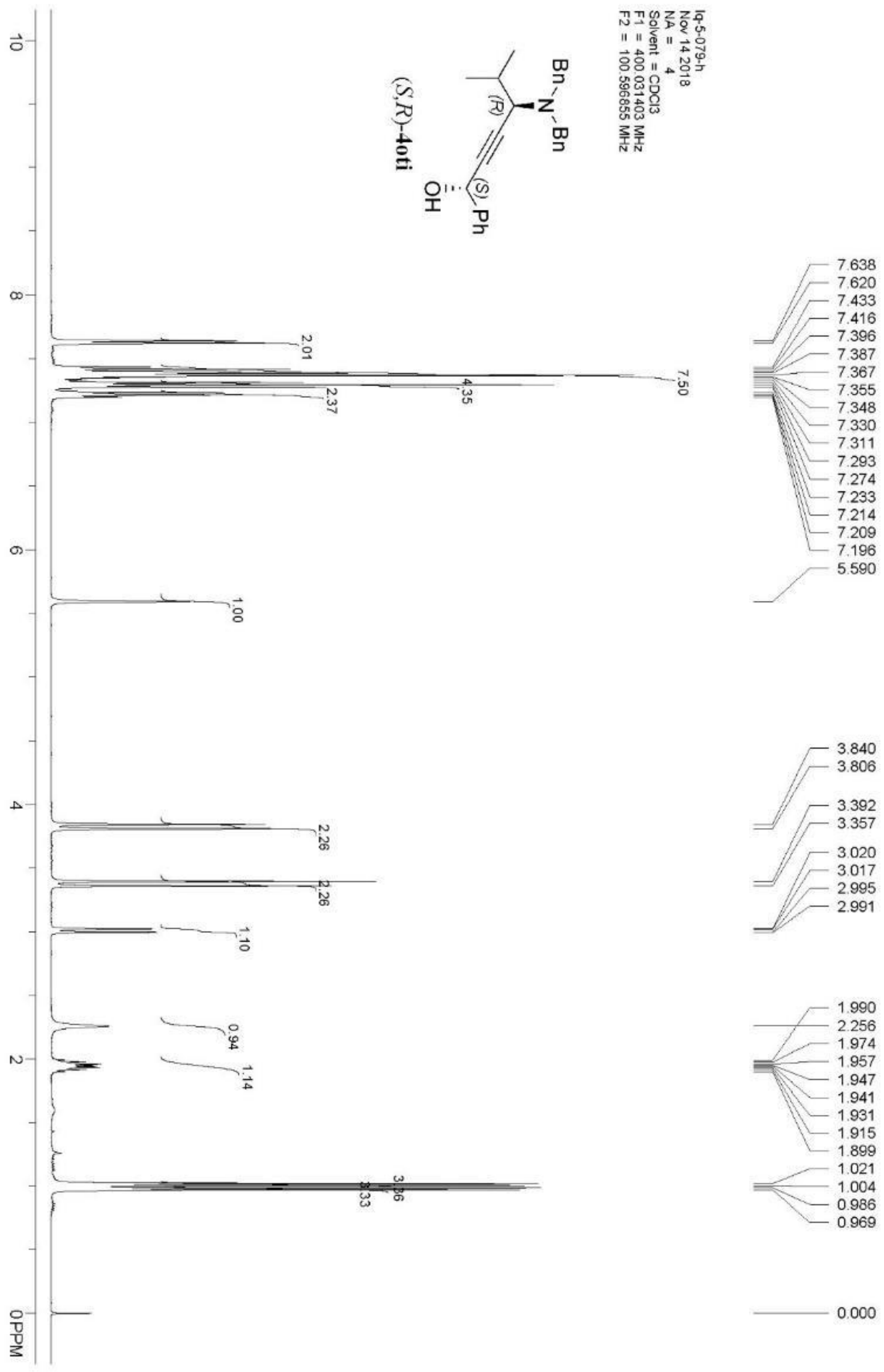

^{1}H NMR (400 MHz, CDCl3) spectrum for (*S*,*R*)**-4oti**

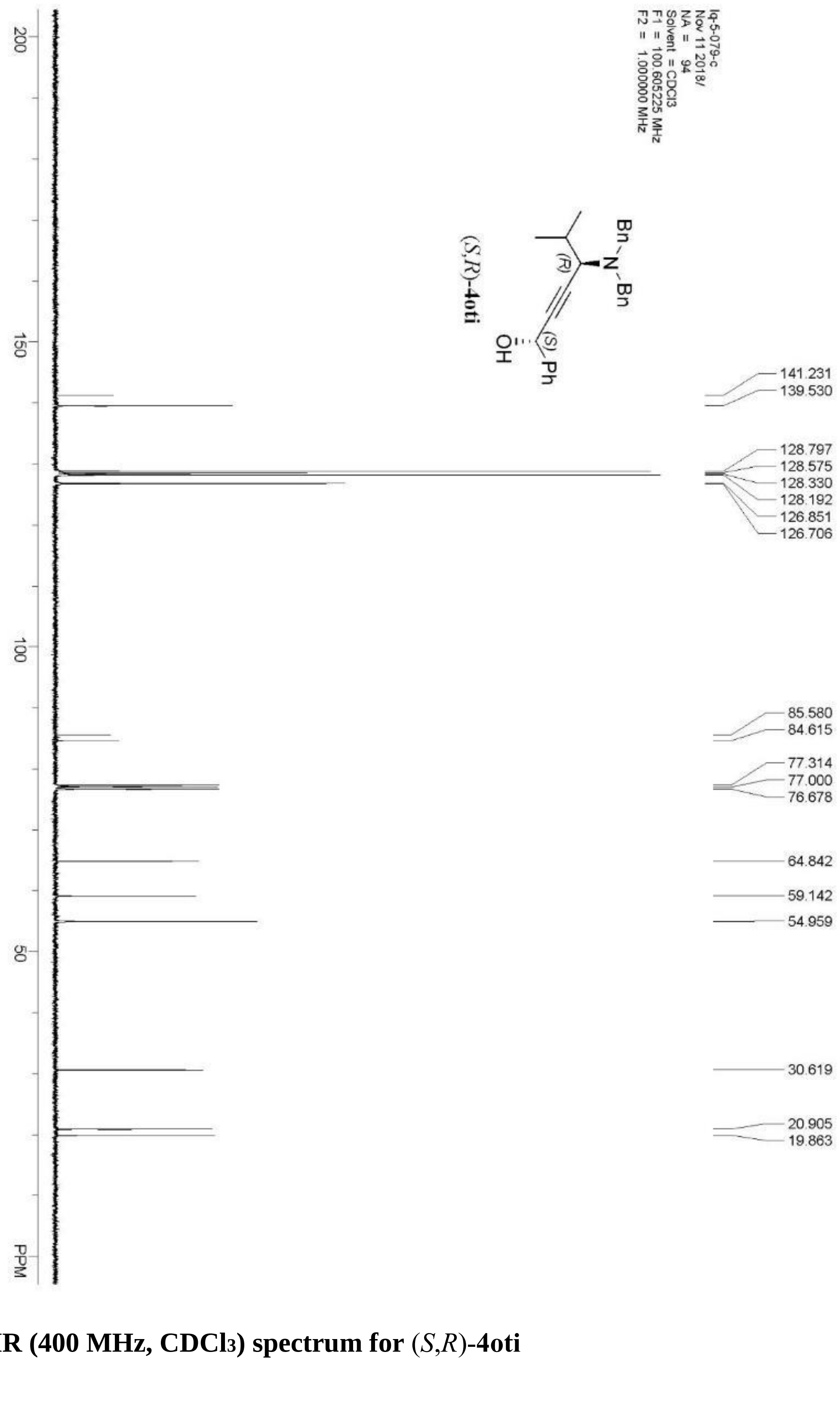

^{13}C NMR (400 MHz, $CDCl_3$) spectrum for (*S*,*R*)-4oti

lq-5-079

实验时间：2018/11/12, 15:36:22　　　　报告时间：2018/11/12, 16:25:05
谱图文件:D:\data\slf\lq\2018-11-12\lq-5-079-OD-H-95+5-0.5-214-2.org

实验内容简介：
OD-H　95:5
214nm　0.5ml/min

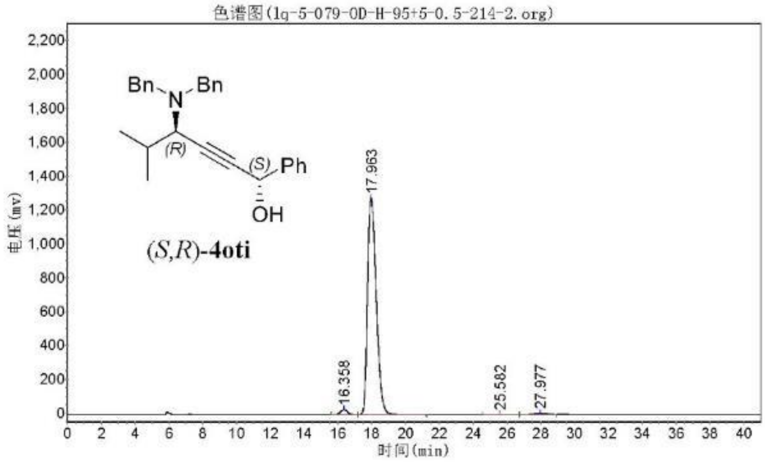

分析结果表

峰号	峰名	保留时间	峰高	峰面积	含量
1		16.358	29017.451	811359.500	1.7254
2		17.963	1276106.125	45792368.000	97.3778
3		25.582	258.156	16192.309	0.0344
4		27.977	6854.787	405556.188	0.8624
总计			1312236.519	47025475.996	100.0000

HPLC spectrum for (*S*,*R*)-4oti

xhb-1-094

实验时间：2018/11/12, 14:12:40　　　　报告时间：2018/11/12, 16:21:00
谱图文件:D:\data\slf\lq\2018-11-12\xhb-1-094-OD-H-95+5-0.5-214.org

实验内容简介：
OD-H　95:5
214nm　0.5ml/min

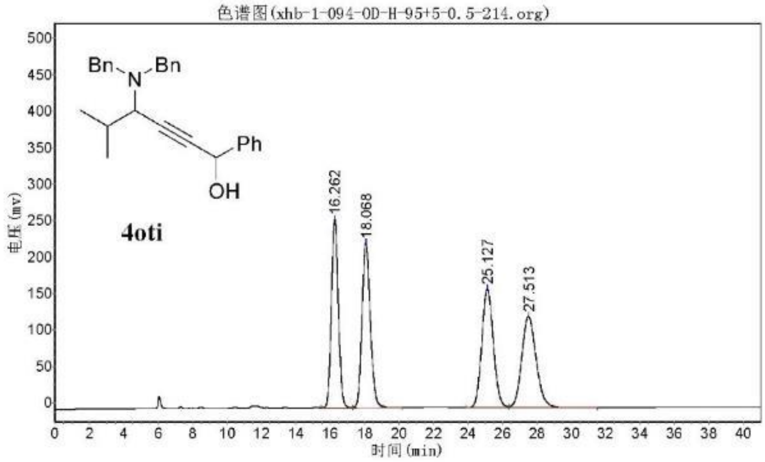

分析结果表

峰号	峰名	保留时间	峰高	峰面积	含量
1		16.262	258340.188	7219212.500	24.2741
2		18.068	225544.844	7632276.500	25.6630
3		25.127	162805.734	7627264.000	25.6461
4		27.513	124881.055	7261655.500	24.4168
总计			771571.820	29740408.500	100.0000

HPLC spectrum for (±)-4oti

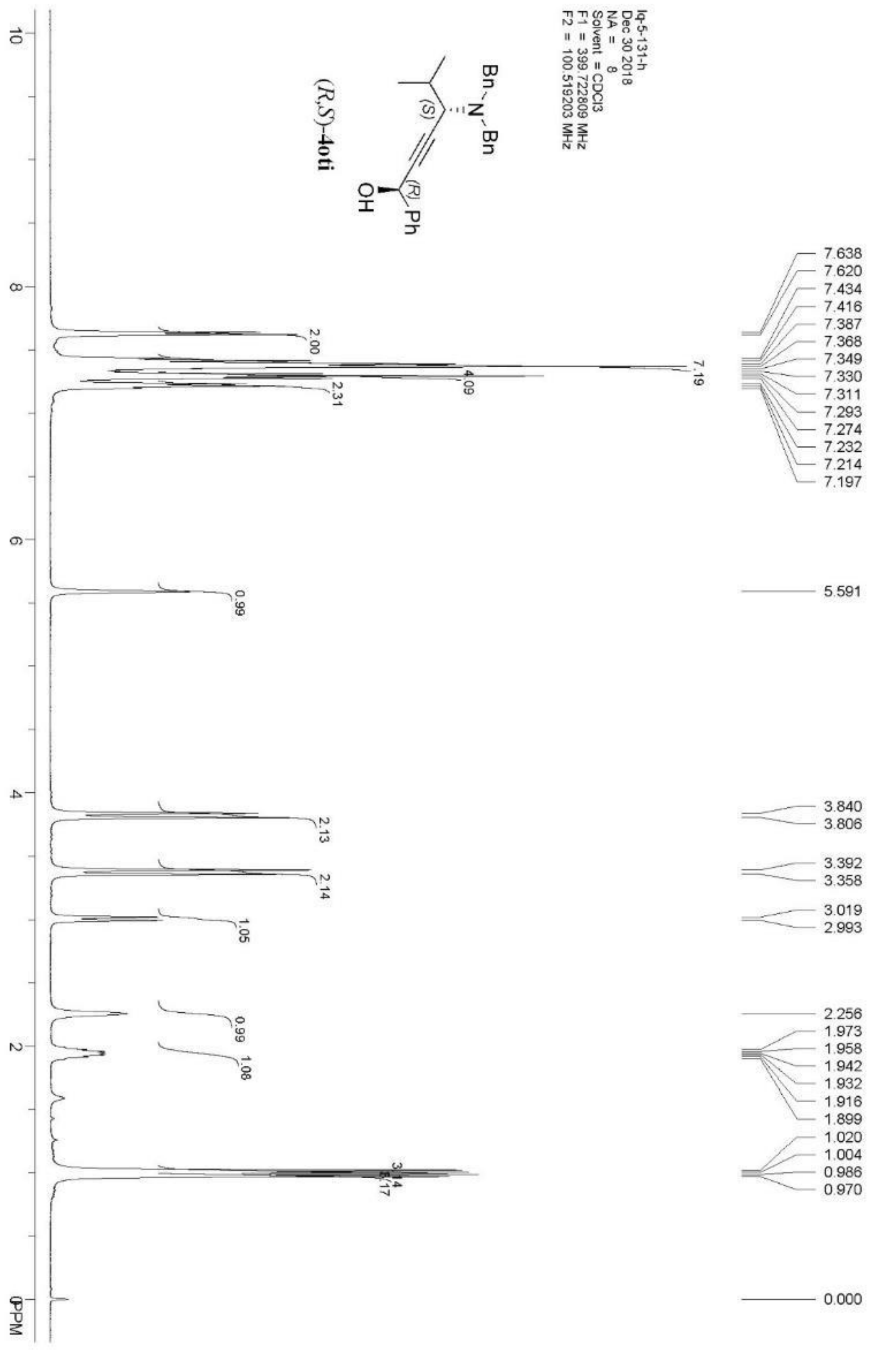

^{1}H NMR (400 MHz, CDCl3) spectrum for (*R*,*S*)-**4oti**

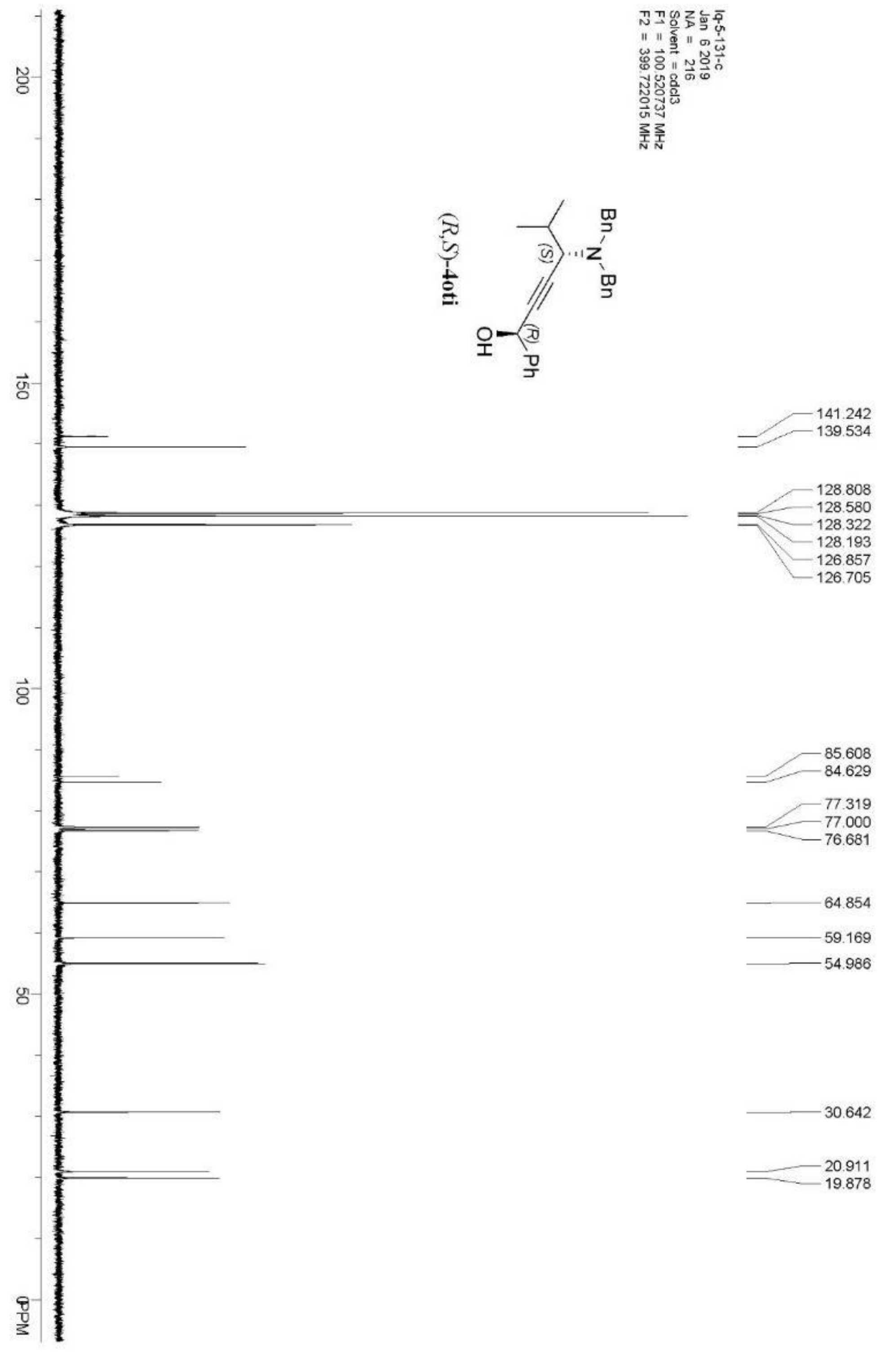

^{13}C NMR (400 MHz, CDCl$_3$) spectrum for (*R*,*S*)-4oti

Empower 3 SOFTWARE

Injection Summary Report

SAMPLE INFORMATION

Sample Name:	lq-5-131-od-h-95-5-0.5-214	Acquired By:	System
Sample Type:	Unknown	Sample Set Name:	
Vial:	1	Acq. Method Set:	HPLC
Injection #:	2	Processing Method:	Default
Injection Volume:	5.00 ul	Channel Name:	W2489 ChA
Run Time:	60.0 Minutes	Proc. Chnl. Descr.:	W2489 ChA 214nm
Date Acquired:	1/3/2019 2:45:26 AM CST		
Date Processed:	1/3/2019 3:57:10 AM CST		

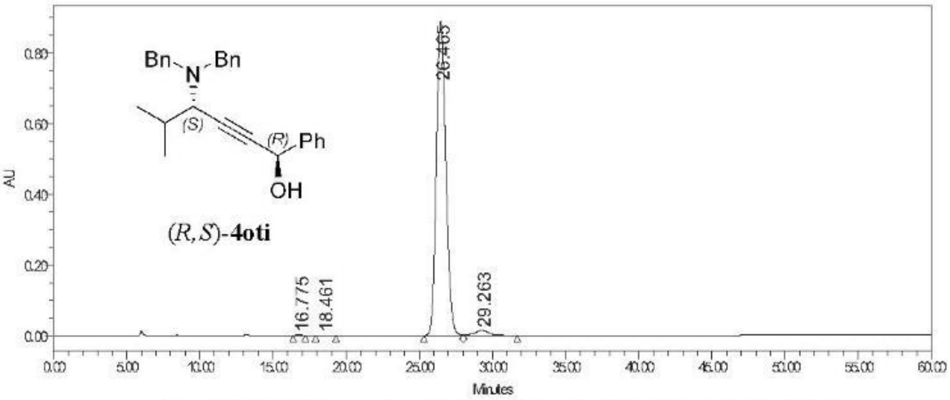

Processed Channel Descr.: W2489 ChA 214nm

	Processed Channel Descr.	RT	Area	%Area	Height
1	W2489 ChA 214nm	16.775	49392	0.12	2039
2	W2489 ChA 214nm	18.461	23997	0.06	787
3	W2489 ChA 214nm	26.465	40154806	97.52	890086
4	W2489 ChA 214nm	29.263	949569	2.31	14070

Reported by User: System
Report Method: Injection Summary Report
Report Method ID: 1639 1639
Page: 1 of 1

Project Name: HPLC
Date Printed:
1/3/2019
4:59:22 AM PRC

HPLC spectrum for (*R*,*S*)-4oti

Empower™ 3 SOFTWARE

Injection Summary Report

SAMPLE INFORMATION

Sample Name:	xhb-1-094-od-h-95-5-0.5-214	Acquired By:	System
Sample Type:	Unknown	Sample Set Name:	
Vial:	1	Acq. Method Set:	HPLC
Injection #:	1	Processing Method:	Default
Injection Volume:	5.00 ul	Channel Name:	W2489 ChA
Run Time:	60.0 Minutes	Proc. Chnl. Descr.:	W2489 ChA 214nm
Date Acquired:	1/3/2019 1:22:29 AM CST		
Date Processed:	1/3/2019 2:46:28 AM CST		

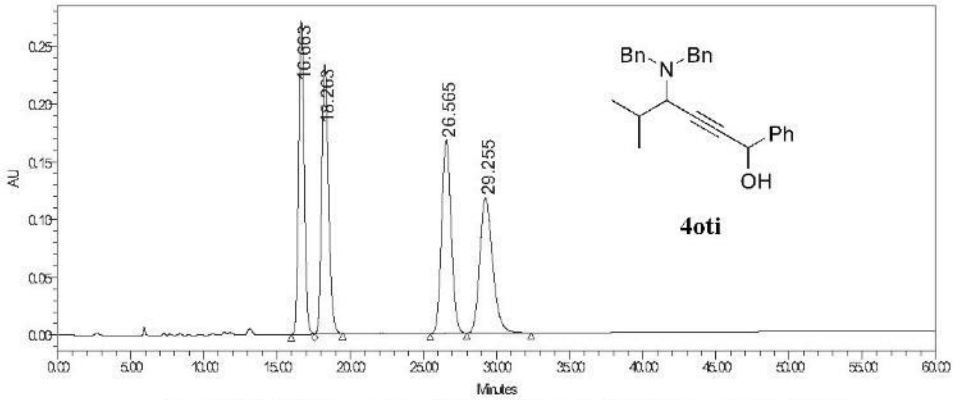

Channel: W2489 ChA; Processed Channel: W2489 ChA 214nm; Result Id: 2169; Processing Method: Defaul

Processed Channel Descr.: W2489 ChA 214nm

	Processed Channel Descr.	RT	Area	%Area	Height
1	W2489 ChA 214nm	16.663	7201446	24.41	271350
2	W2489 ChA 214nm	18.263	7548313	25.59	233444
3	W2489 ChA 214nm	26.565	7602965	25.77	167146
4	W2489 ChA 214nm	29.255	7144870	24.22	116821

Reported by User: System
Report Method: Injection Summary Report
Report Method ID: 1639 1639
Page: 1 of 1

Project Name: HPLC
Date Printed:
1/3/2019
3:12:25 AM PRC

HPLC spectrum for (±)-4oti

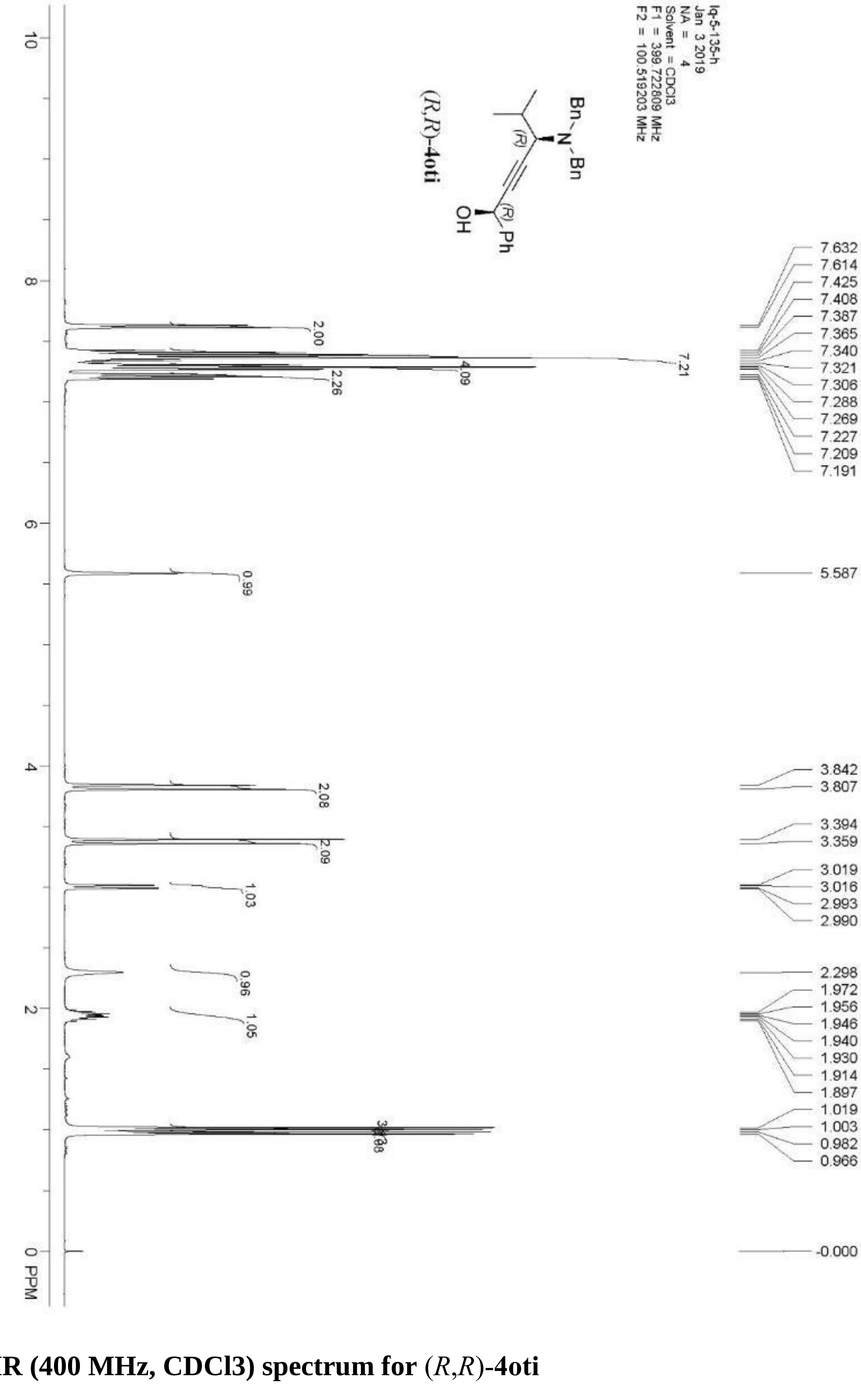

^{1}H NMR (400 MHz, CDCl3) spectrum for (*R*,*R*)**-4oti**

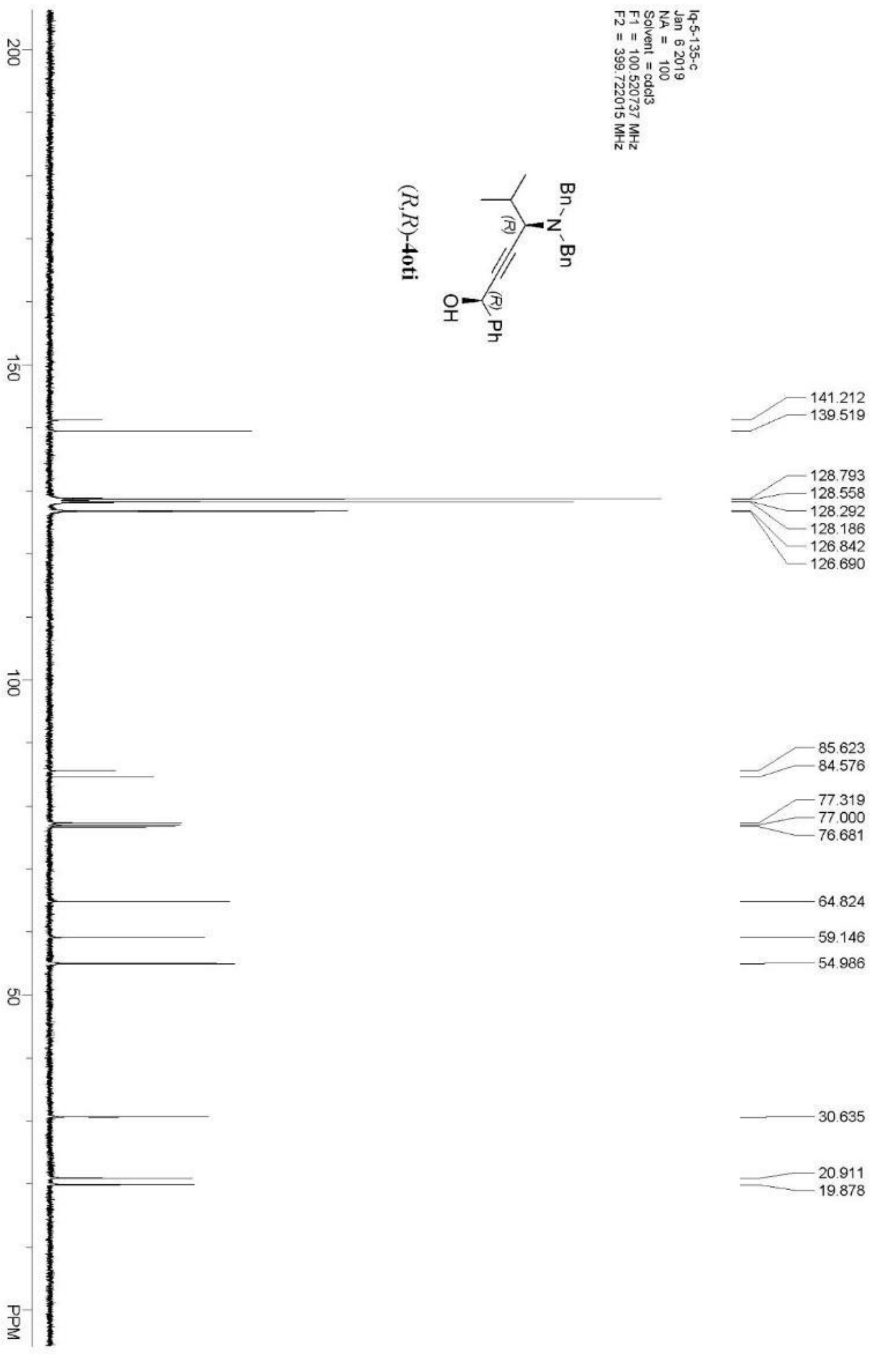

^{13}C NMR (400 MHz, $CDCl_3$) spectrum for (*R*,*R*)**-4oti**

Empower 3

Injection Summary Report

SAMPLE INFORMATION

Sample Name:	lq-5-135-od-h-95-5-0.5-214	Acquired By:	System
Sample Type:	Unknown	Sample Set Name:	
Vial:	1	Acq. Method Set:	HPLC
Injection #:	1	Processing Method:	Default
Injection Volume:	5.00 ul	Channel Name:	W2489 ChA
Run Time:	60.0 Minutes	Proc. Chnl. Descr.:	W2489 ChA 214nm
Date Acquired:	1/4/2019 10:56:25 AM CST		
Date Processed:	1/4/2019 1:01:29 PM CST		

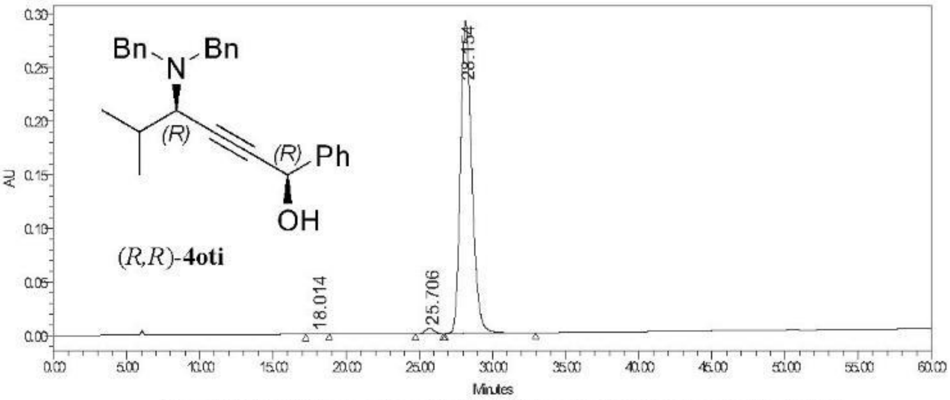

Processed Channel Descr.: W2489 ChA 214nm

	Processed Channel Descr.	RT	Area	%Area	Height
1	W2489 ChA 214nm	18.014	12106	0.08	413
2	W2489 ChA 214nm	25.706	203448	1.28	5001
3	W2489 ChA 214nm	28.154	15617138	98.64	290868

Reported by User: System
Report Method: Injection Summary Report
Report Method ID: 1639 1639
Page: 1 of 1

Project Name: HPLC
Date Printed:
1/4/2019
1:02:25 PM PRC

HPLC spectrum for (*R*,*R*)-4oti

Empower™ 3 SOFTWARE

Injection Summary Report

SAMPLE INFORMATION

Sample Name:	xhb-1-094-od-h-95-5-0.5-214	Acquired By:	System
Sample Type:	Unknown	Sample Set Name:	
Vial:	1	Acq. Method Set:	HPLC
Injection #:	2	Processing Method:	Default
Injection Volume:	5.00 ul	Channel Name:	W2489 ChA
Run Time:	60.0 Minutes	Proc. Chnl. Descr.:	W2489 ChA 214nm
Date Acquired:	1/4/2019 11:57:08 AM CST		
Date Processed:	1/4/2019 12:58:41 PM CST		

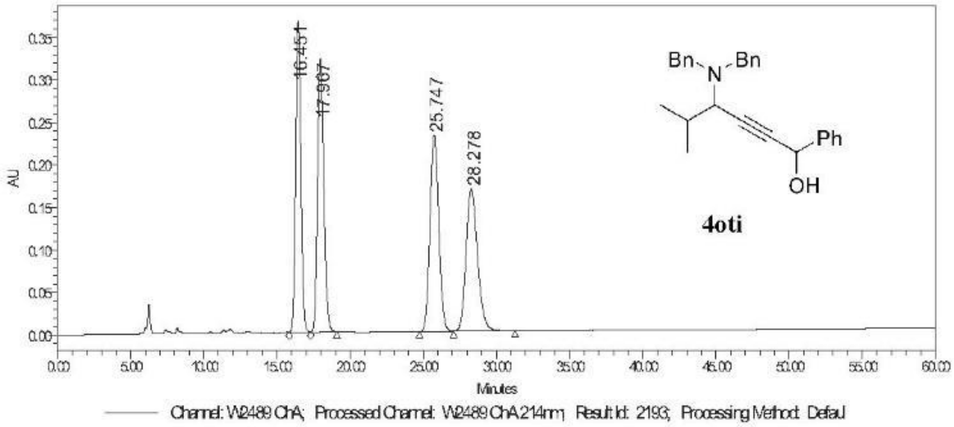

4oti

Processed Channel Descr.: W2489 ChA 214nm

	Processed Channel Descr.	RT	Area	%Area	Height
1	W2489 ChA 214nm	16.451	9054415	24.35	366121
2	W2489 ChA 214nm	17.967	9503704	25.56	321878
3	W2489 ChA 214nm	25.747	9577646	25.75	230467
4	W2489 ChA 214nm	28.278	9052054	24.34	167283

Reported by User: System
Report Method: Injection Summary Report
Report Method ID: 1639 1639
Page: 1 of 1

Project Name: HPLC
Date Printed:
1/4/2019
12:58:53 PM PRC

HPLC spectrum for (±)-4oti

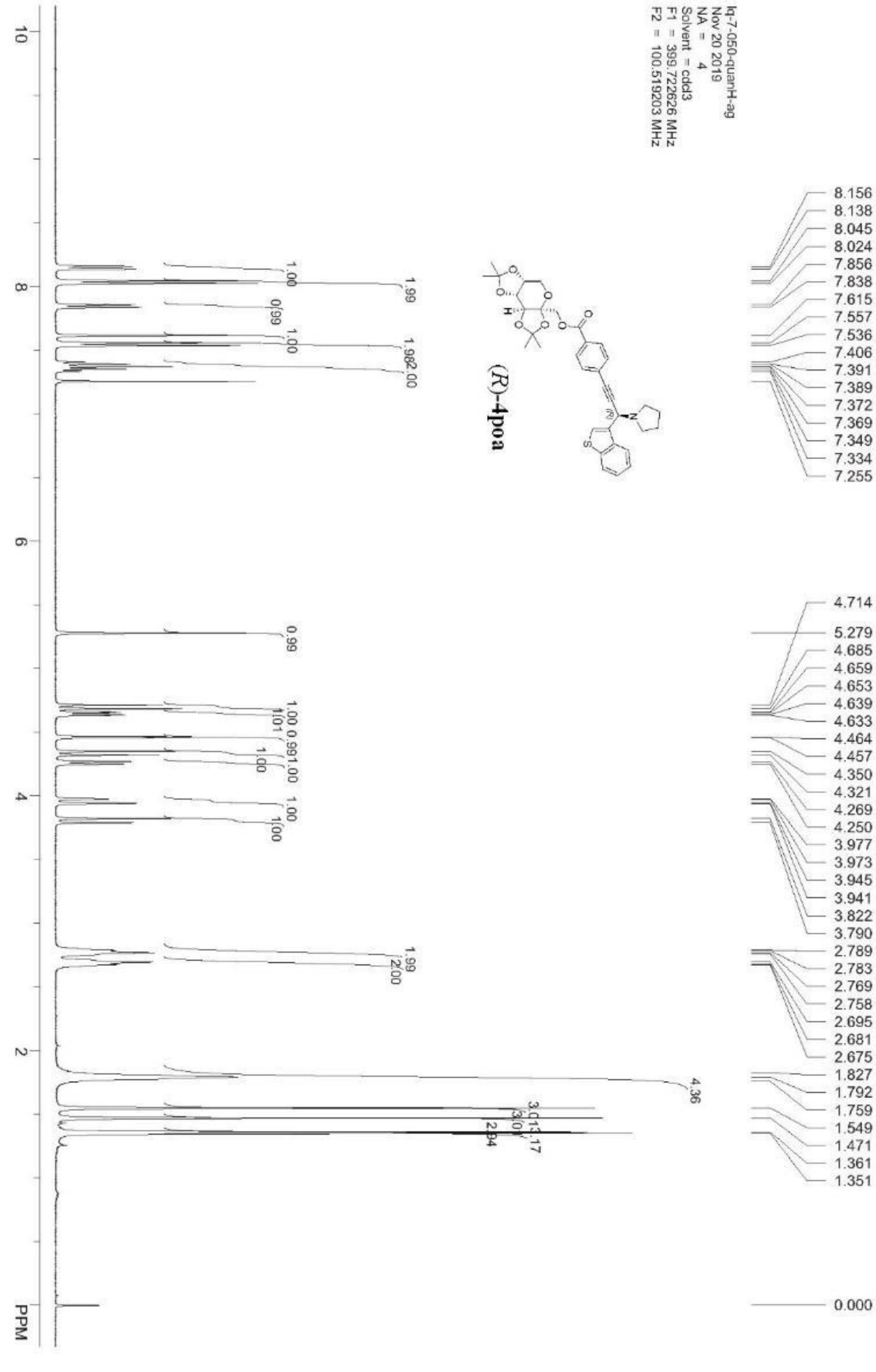

^{1}H NMR (400 MHz, CDCl3) spectrum for (*R*)**-4poa**

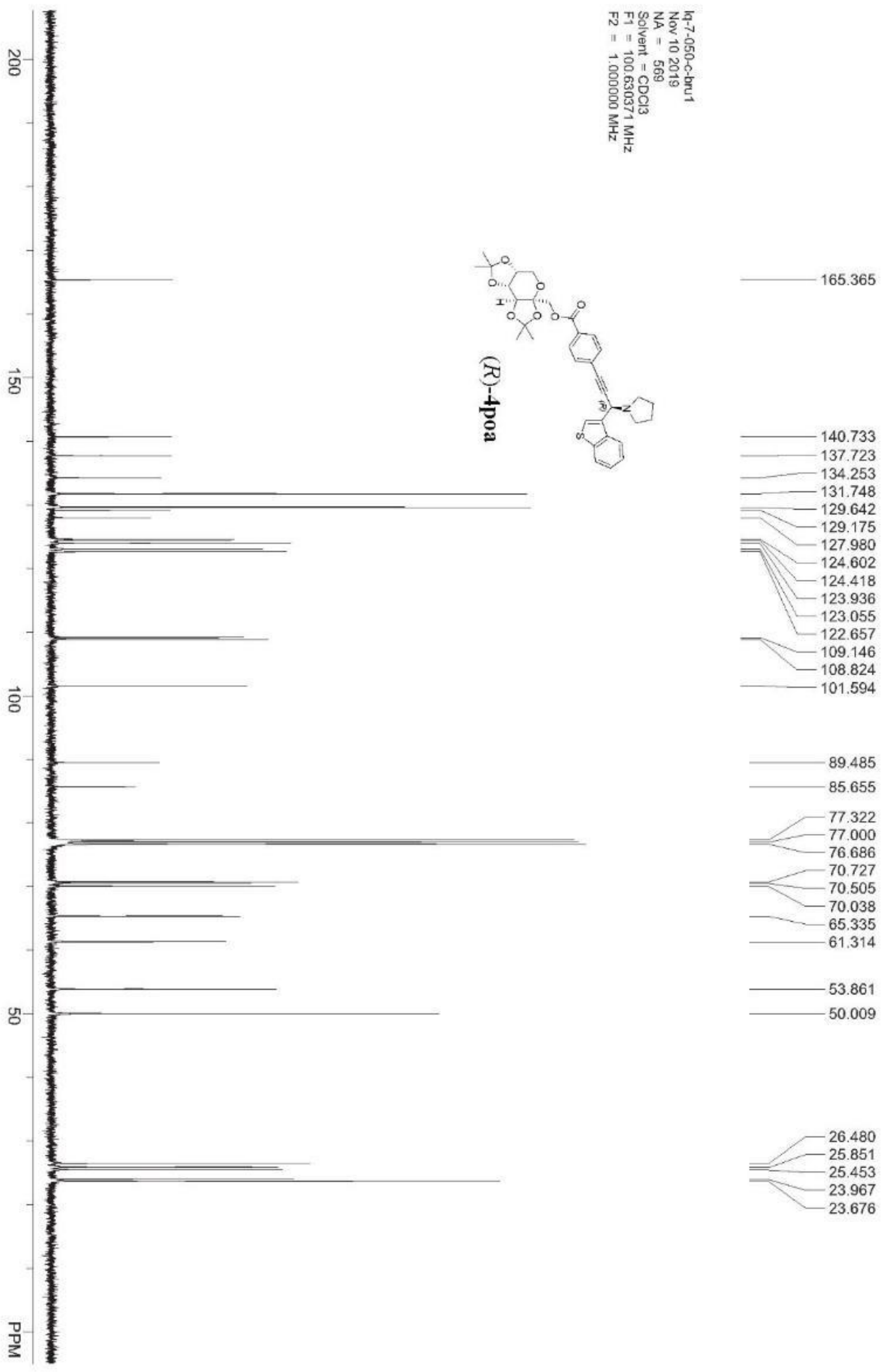

^{13}C NMR (400 MHz, CDCl$_3$) spectrum for (*R*)-4poa

Empower™ 3 SOFTWARE

Injection Summary Report

SAMPLE INFORMATION

Sample Name:	lq-7-050-cz-h-95-5-1-214	Acquired By:	System
Sample Type:	Unknown	Sample Set Name:	
Vial:	1	Acq. Method Set:	HPLC
Injection #:	3	Processing Method:	Default
Injection Volume:	2.00 ul	Channel Name:	W2489 ChA
Run Time:	24.0 Minutes	Proc. Chnl. Descr.:	W2489 ChA 214nm
Date Acquired:	11/20/2019 11:52:01 PM CST		
Date Processed:	11/21/2019 12:38:44 AM CST		

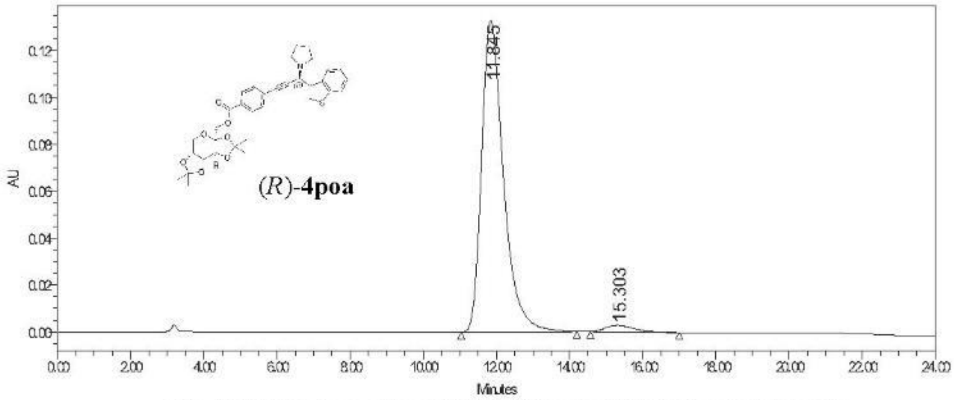

Channel: W2489 ChA; Processed Channel: W2489 ChA 214nm; Result Id: 6115; Processing Method: Defaul

Processed Channel Descr.: W2489 ChA 214nm

	Processed Channel Descr.	RT	Area	% Area	Height
1	W2489 ChA 214nm	11.845	5491963	97.35	132596
2	W2489 ChA 214nm	15.303	149530	2.65	2742

Reported by User: System
Report Method: Injection Summary Report
Report Method ID: 1639 1639
Page: 1 of 1

Project Name: HPLC
Date Printed:
11/21/2019
12:48:26 AM PRC

HPLC spectrum for (*R*)-4poa

Empower™ 3 SOFTWARE

Injection Summary Report

SAMPLE INFORMATION

Sample Name:	lq-7-047-oz-h-95-5-1-214	Acquired By:	System
Sample Type:	Unknown	Sample Set Name:	
Vial:	1	Acq. Method Set:	HPLC
Injection #:	4	Processing Method:	Default
Injection Volume:	5.00 ul	Channel Name:	W2489 ChA
Run Time:	24.0 Minutes	Proc. Chnl. Descr.:	W2489 ChA 214nm
Date Acquired:	11/21/2019 12:22:58 AM CST		
Date Processed:	11/21/2019 12:47:31 AM CST		

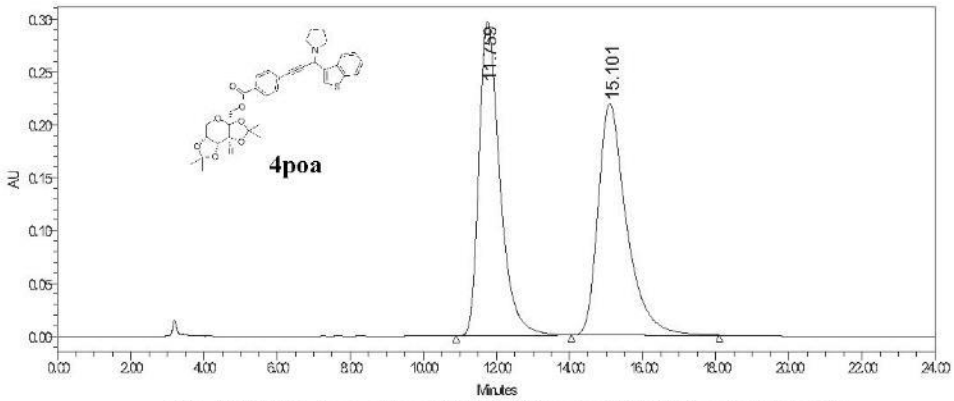

Processed Channel Descr.: W2489 ChA 214nm

	Processed Channel Descr.	RT	Area	%Area	Height
1	W2489 ChA 214nm	11.759	11767019	50.02	296364
2	W2489 ChA 214nm	15.101	11759671	49.98	218731

Reported by User: System
Report Method: Injection Summary Report
Report Method ID: 1639 1639
Page: 1 of 1

Project Name: HPLC
Date Printed:
11/21/2019
12:47:52 AM PRC

HPLC spectrum for (±)-4poa

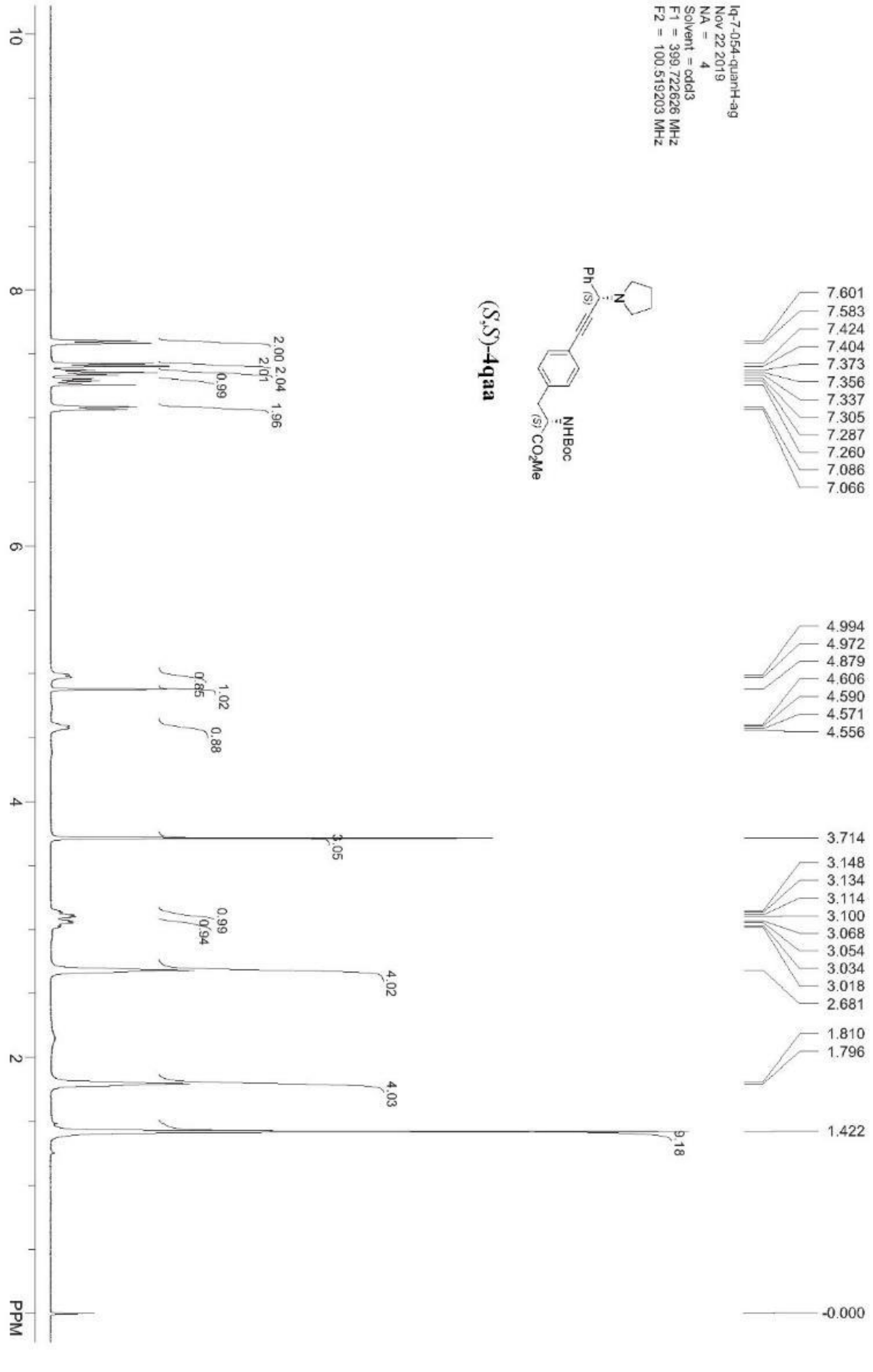

^{1}H NMR (400 MHz, CDCl3) spectrum for (*S*,*S*)**-4qaa**

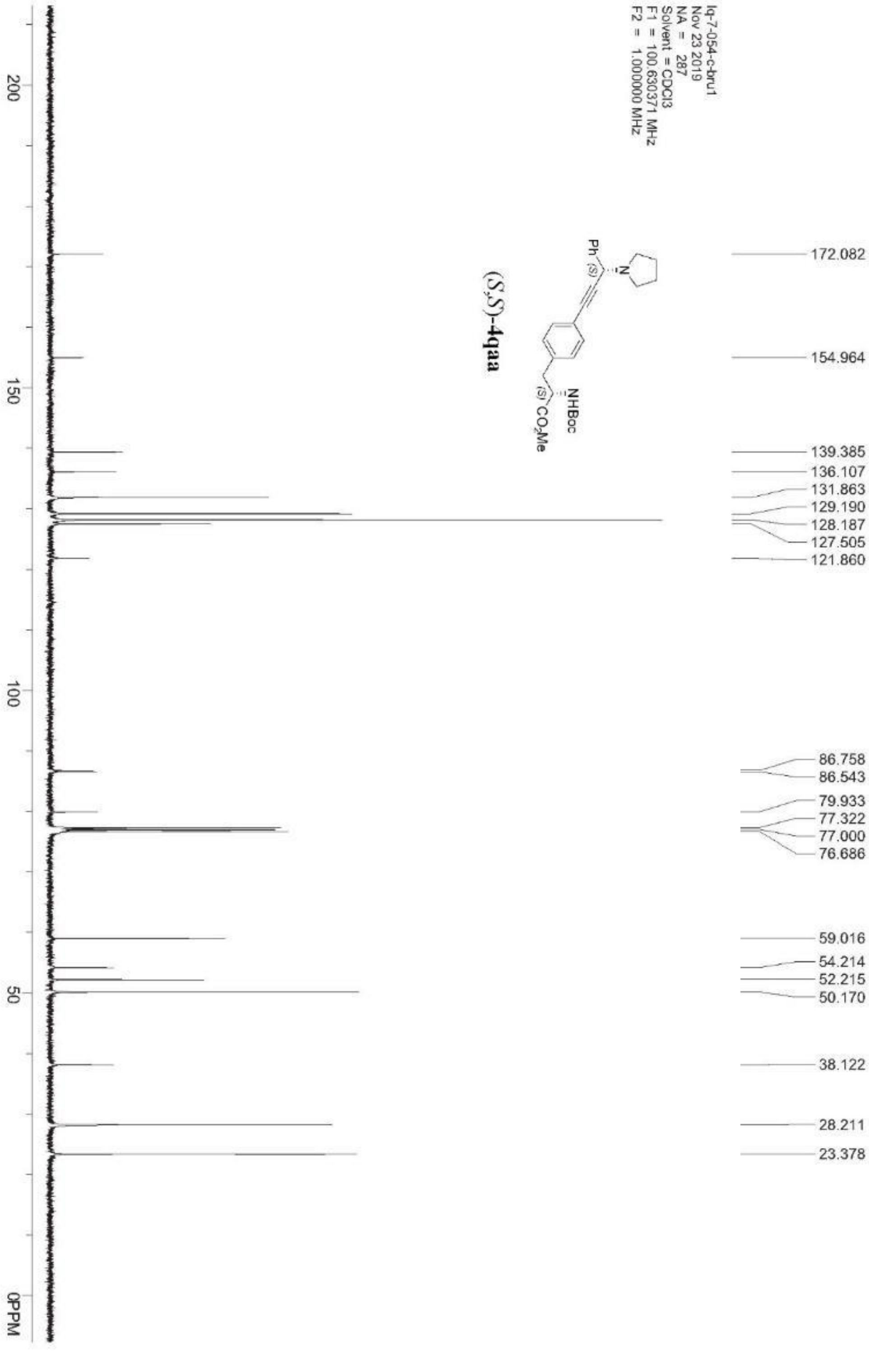

^{13}C NMR (400 MHz, CDCl$_3$) spectrum for (*S*,*S*)**-4qaa**

Empower 3 SOFTWARE

Injection Summary Report

SAMPLE INFORMATION

Sample Name:	lq-7-054-oj-h-95-5-1-214	Acquired By:	System
Sample Type:	Unknown	Sample Set Name:	
Vial:	1	Acq. Method Set:	HPLC
Injection #:	4	Processing Method:	Default
Injection Volume:	4.00 ul	Channel Name:	W2489 ChA
Run Time:	30.0 Minutes	Proc. Chnl. Descr.:	W2489 ChA 214nm
Date Acquired:	11/24/2019 7:43:50 AM CST		
Date Processed:	11/24/2019 8:33:15 AM CST		

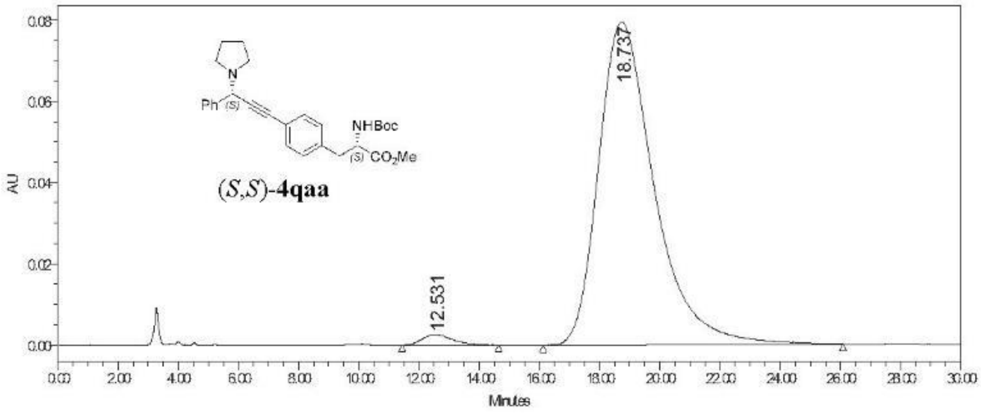

Channel: W2489 ChA; Processed Channel: W2489 ChA 214nm; Result Id: 6142; Processing Method: Defaul

Processed Channel Descr.: W2489 ChA 214nm

	Processed Channel Descr.	RT	Area	% Area	Height
1	W2489 ChA 214nm	12.531	187119	1.80	2668
2	W2489 ChA 214nm	18.737	10204204	98.20	79499

Reported by User: System
Report Method: Injection Summary Report
Report Method ID: 1639 1639
Page: 1 of 1

Project Name: HPLC
Date Printed:
11/24/2019
8:33:48 AM PRC

HPLC spectrum for (*S*,*S*)-4qaa

Empower 3 SOFTWARE

Injection Summary Report

SAMPLE INFORMATION

Sample Name:	lq-7-049-oj-h-95-5-1-214	Acquired By:	System
Sample Type:	Unknown	Sample Set Name:	
Vial:	1	Acq. Method Set:	HPLC
Injection #:	1	Processing Method:	Default
Injection Volume:	5.00 ul	Channel Name:	W2489 ChA
Run Time:	30.0 Minutes	Proc. Chnl. Descr.:	W2489 ChA 214nm
Date Acquired:	11/24/2019 5:52:05 AM CST		
Date Processed:	11/24/2019 8:31:31 AM CST		

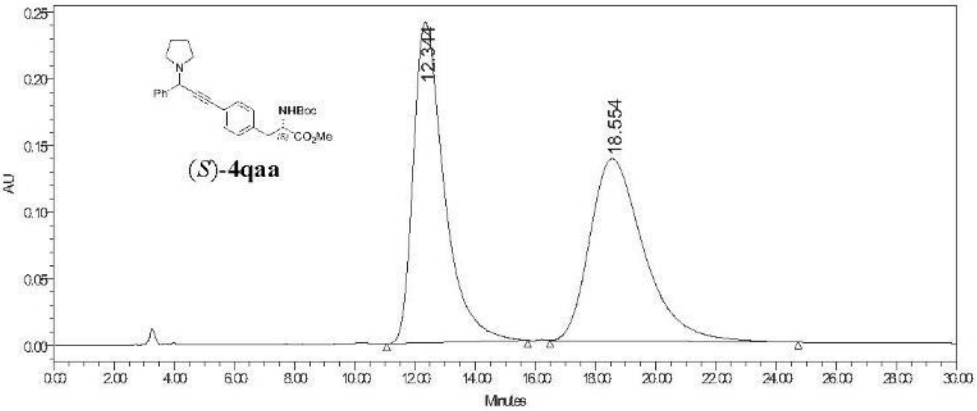

Processed Channel Descr.: W2489 ChA 214nm

	Processed Channel Descr.	RT	Area	% Area	Height
1	W2489 ChA 214nm	12.344	17102591	50.17	240722
2	W2489 ChA 214nm	18.554	16988626	49.83	136596

Reported by User: System
Report Method: Injection Summary Report
Report Method ID: 1639 1639
Page: 1 of 1

Project Name: HPLC
Date Printed:
11/24/2019
8:34:34 AM PRC

HPLC spectrum for (±)-4qaa

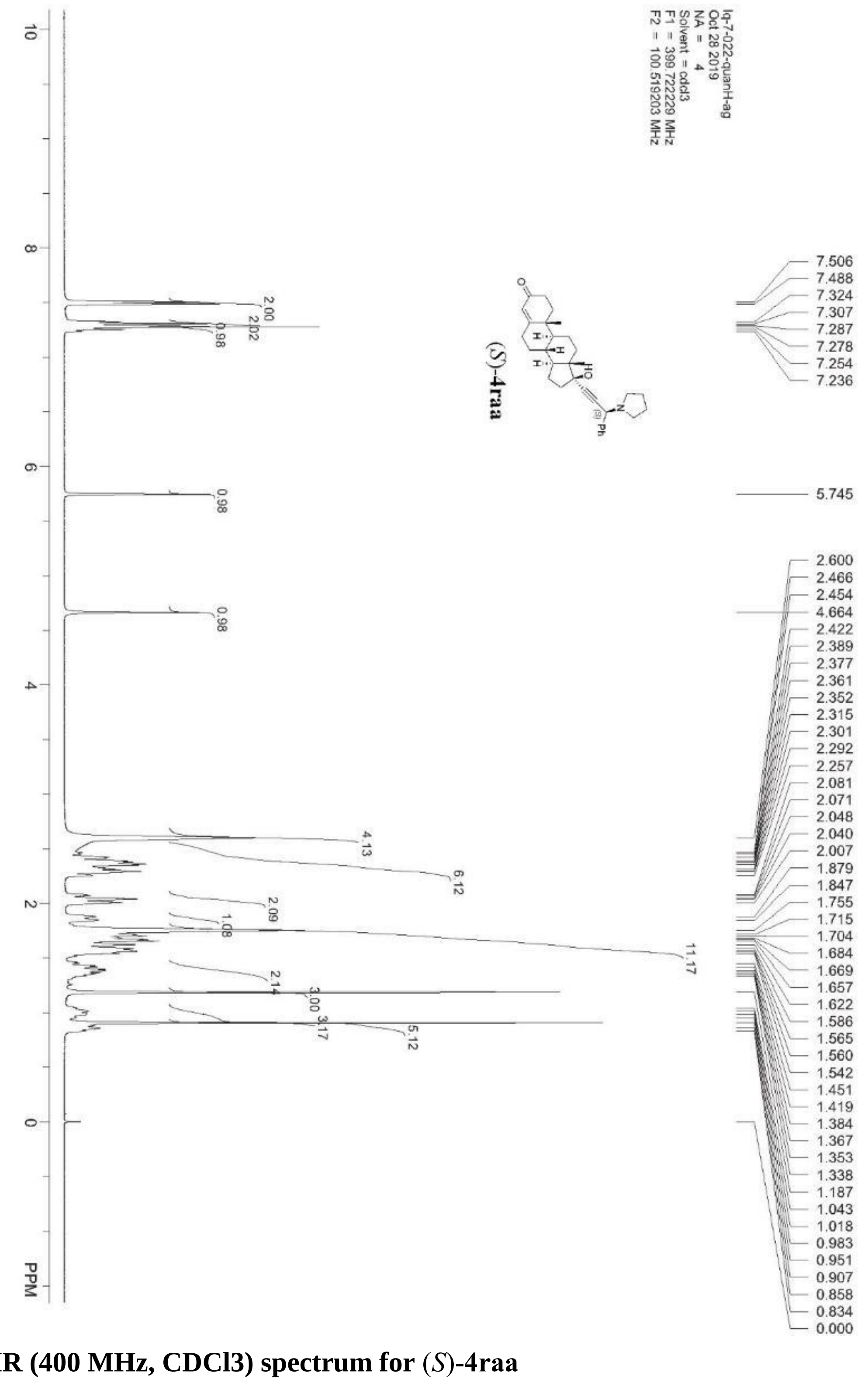

^{1}H NMR (400 MHz, CDCl3) spectrum for (*S*)-4raa

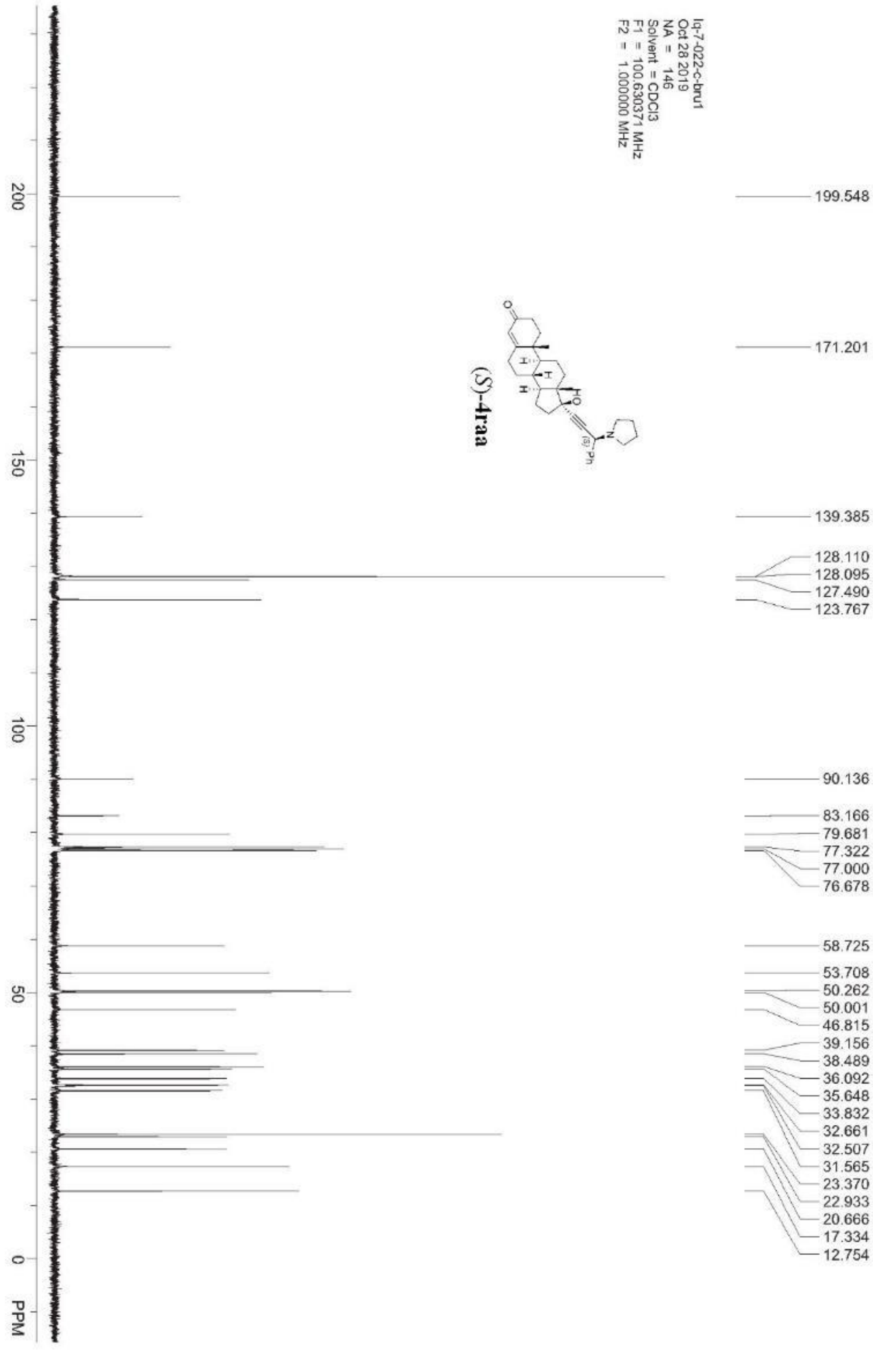

^{13}C NMR (400 MHz, CDCl$_3$) spectrum for (*S*)-4raa

Empower 3 SOFTWARE

Injection Summary Report

SAMPLE INFORMATION

Sample Name:	lq-7-022-ad-h-95-5-1-214	Acquired By:	System
Sample Type:	Unknown	Sample Set Name:	
Vial:	1	Acq. Method Set:	HPLC
Injection #:	2	Processing Method:	Default
Injection Volume:	5.00 ul	Channel Name:	W2489 ChA
Run Time:	80.0 Minutes	Proc. Chnl. Descr.:	W2489 ChA 214nm
Date Acquired:	11/2/2019 9:59:25 AM CST		
Date Processed:	11/2/2019 2:10:36 PM CST		

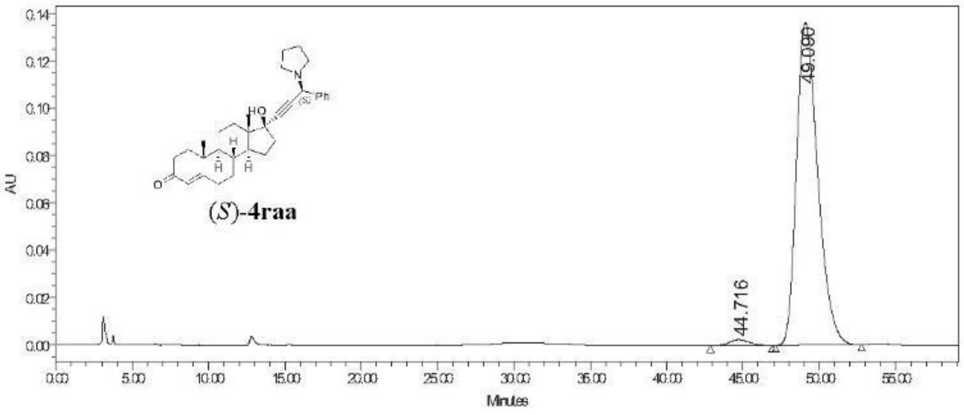

Channel: W2489 ChA; Processed Channel: W2489 ChA 214nm; Result Id: 5853; Processing Method: Defaul

Processed Channel Descr.: W2489 ChA 214nm

	Processed Channel Descr.	RT	Area	% Area	Height
1	W2489 ChA 214nm	44.716	214191	1.60	2468
2	W2489 ChA 214nm	49.090	13134275	98.40	136174

Reported by User: System
Report Method: Injection Summary Report
Report Method ID: 1639 1639
Page: 1 of 1

Project Name: HPLC
Date Printed:
11/2/2019
2:11:02 PM PRC

HPLC spectrum for (*S*)-4raa

Empower 3 SOFTWARE

Injection Summary Report

SAMPLE INFORMATION

Sample Name:	lq-7-015-ad-h-95-5-1-214	Acquired By:	System
Sample Type:	Unknown	Sample Set Name:	
Vial:	1	Acq. Method Set:	HPLC
Injection #:	2	Processing Method:	Default
Injection Volume:	10.00 ul	Channel Name:	W2489 ChA
Run Time:	60.0 Minutes	Proc. Chnl. Descr.:	W2489 ChA 214nm
Date Acquired:	11/2/2019 1:58:41 AM CST		
Date Processed:	11/2/2019 2:09:15 PM CST		

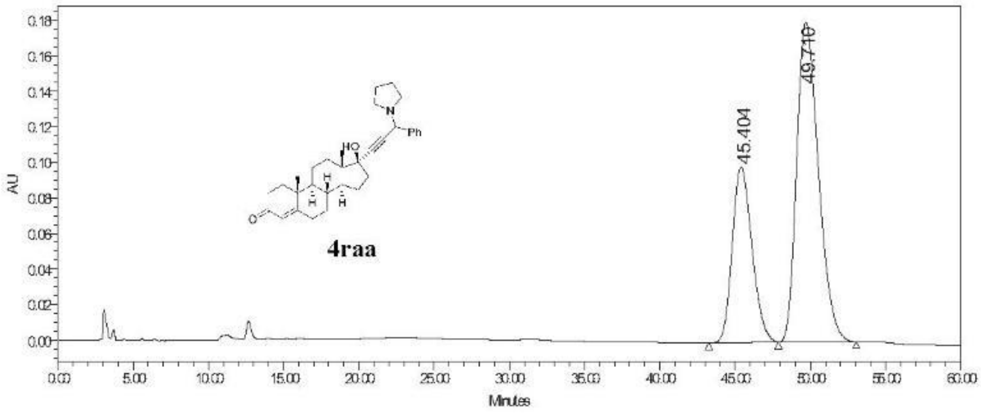

Channel: W2489 ChA; Processed Channel: W2489 ChA 214nm; Result Id: 5851; Processing Method: Defaul

Processed Channel Descr.: W2489 ChA 214nm

	Processed Channel Descr.	RT	Area	% Area	Height
1	W2489 ChA 214nm	45.404	8959945	33.18	98737
2	W2489 ChA 214nm	49.710	1804111	66.82	179649

Reported by User: System
Report Method: Injection Summary Report
Report Method ID: 1639 1639
Page: 1 of 1

Project Name: HPLC
Date Printed:
11/2/2019
2:11:33 PM PRC

HPLC spectrum for (±)-4raa

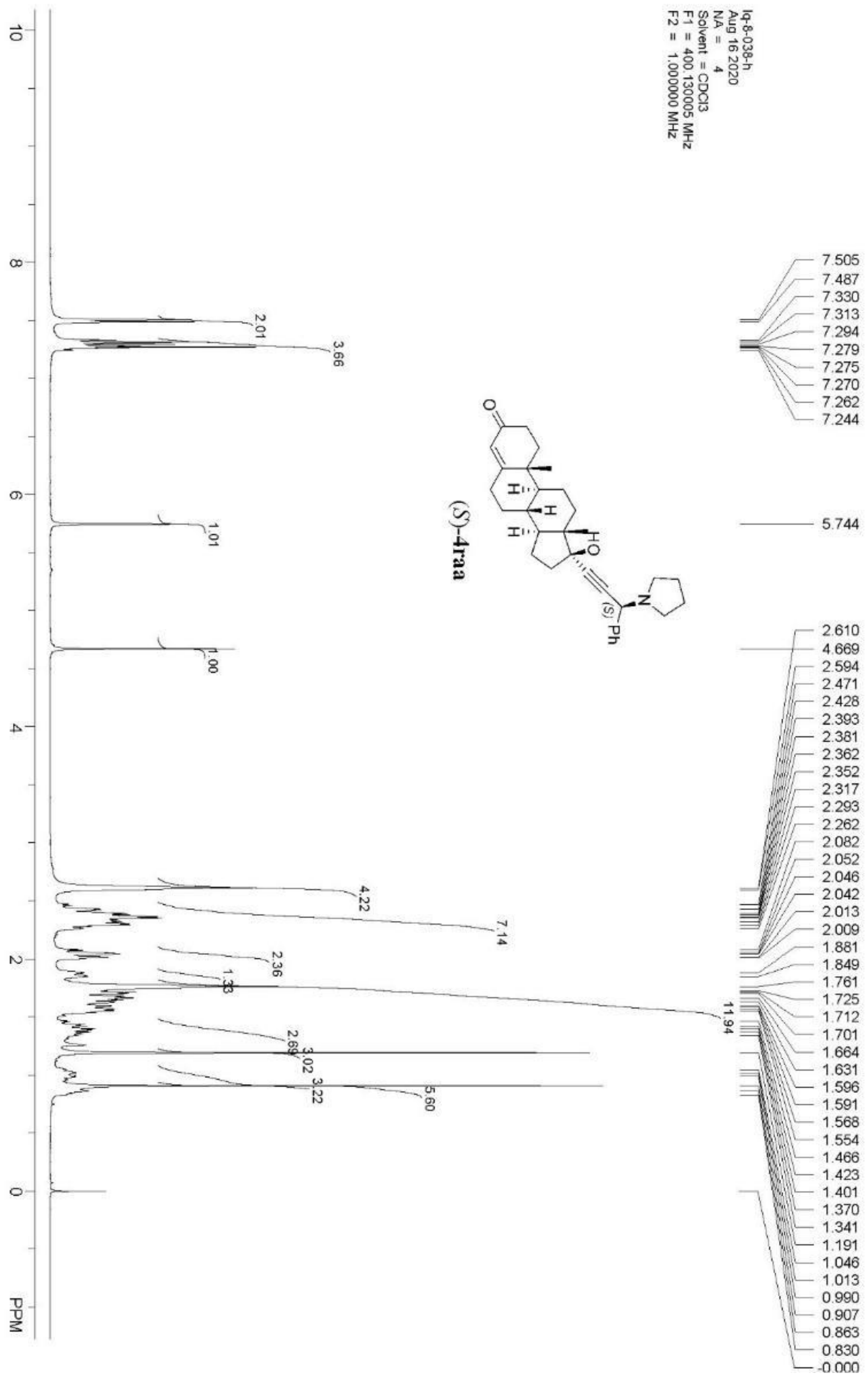

^{1}H NMR (400 MHz, CDCl3) spectrum for (*S*)-4raa

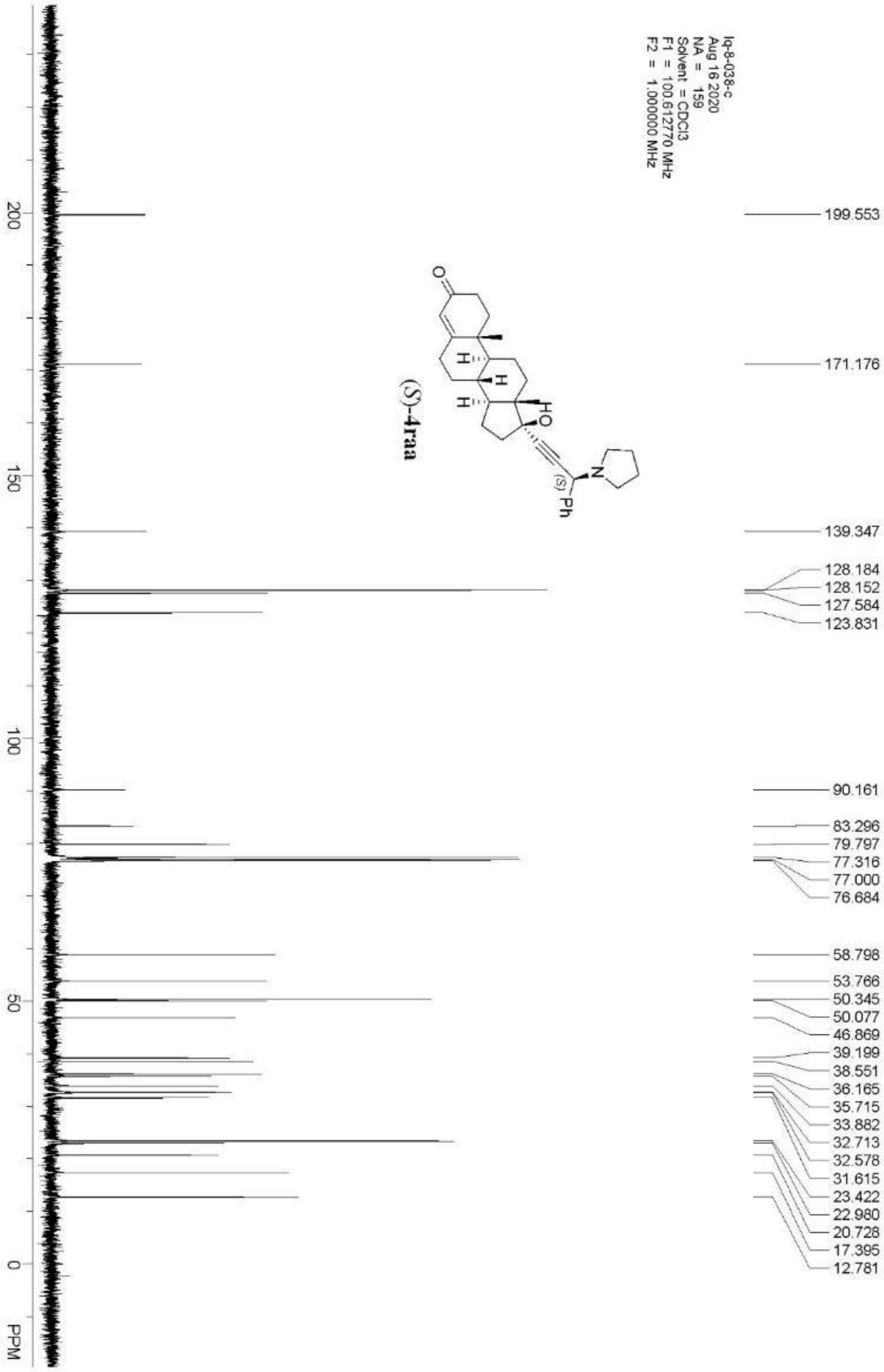

^{13}C NMR (400 MHz, CDCl3) spectrum for (*S*)-4raa

Empower 3 SOFTWARE

Injection Summary Report

SAMPLE INFORMATION

Sample Name:	lq-8-038-cz-h-80-20-0.7-214	Acquired By:	System
Sample Type:	Unknown	Sample Set Name:	
Vial:	1	Acq. Method Set:	HPLC
Injection #:	1	Processing Method:	Default
Injection Volume:	10.00 ul	Channel Name:	W2489 ChA
Run Time:	30.0 Minutes	Proc. Chnl. Descr.:	W2489 ChA 214nm
Date Acquired:	8/16/2020 6:15:57 AM CST		
Date Processed:	8/17/2020 11:04:02 AM CST		

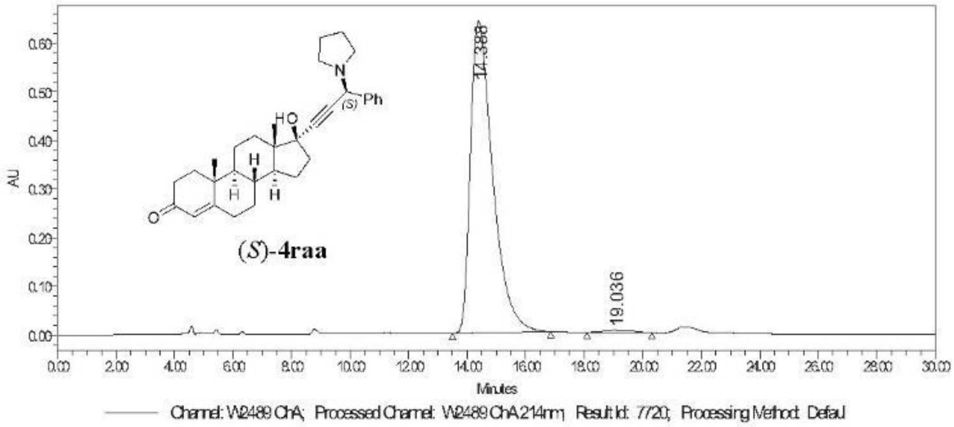

Processed Channel Descr.: W2489 ChA 214nm

	Processed Channel Descr.	RT	Area	%Area	Height
1	W2489 ChA 214nm	14.388	33836564	99.01	641401
2	W2489 ChA 214nm	19.036	337847	0.99	4673

Reported by User: System
Report Method: Injection Summary Report
Report Method ID: 6865 6865
Page: 1 of 1

Project Name: HPLC
Date Printed:
8/17/2020
11:05:11 AM PRC

HPLC spectrum for (*S*)-4raa

Empower 3 SOFTWARE

Injection Summary Report

SAMPLE INFORMATION

Sample Name:	lq-7-015-oz-h-80-20-0.7-214	Acquired By:	System
Sample Type:	Unknown	Sample Set Name:	
Vial:	1	Acq. Method Set:	HPLC
Injection #:	2	Processing Method:	Default
Injection Volume:	10.00 ul	Channel Name:	W2489 ChA
Run Time:	30.0 Minutes	Proc. Chnl. Descr.:	W2489 ChA 214nm
Date Acquired:	8/16/2020 5:34:34 AM CST		
Date Processed:	8/16/2020 6:04:38 AM CST		

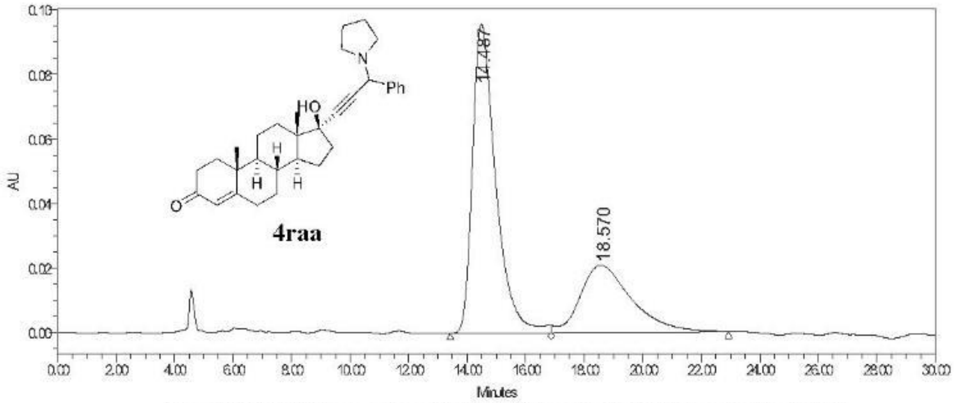

Processed Channel Descr.: W2489 ChA 214nm

	Processed Channel Descr.	RT	Area	% Area	Height
1	W2489 ChA 214nm	14.487	5293723	67.65	95839
2	W2489 ChA 214nm	18.570	2531893	32.35	20882

Reported by User: System
Report Method: Injection Summary Report
Report Method ID: 6865 6865
Page: 1 of 1

Project Name: HPLC
Date Printed:
8/17/2020
11:02:42 AM PRC

HPLC spectrum for (±)-4raa

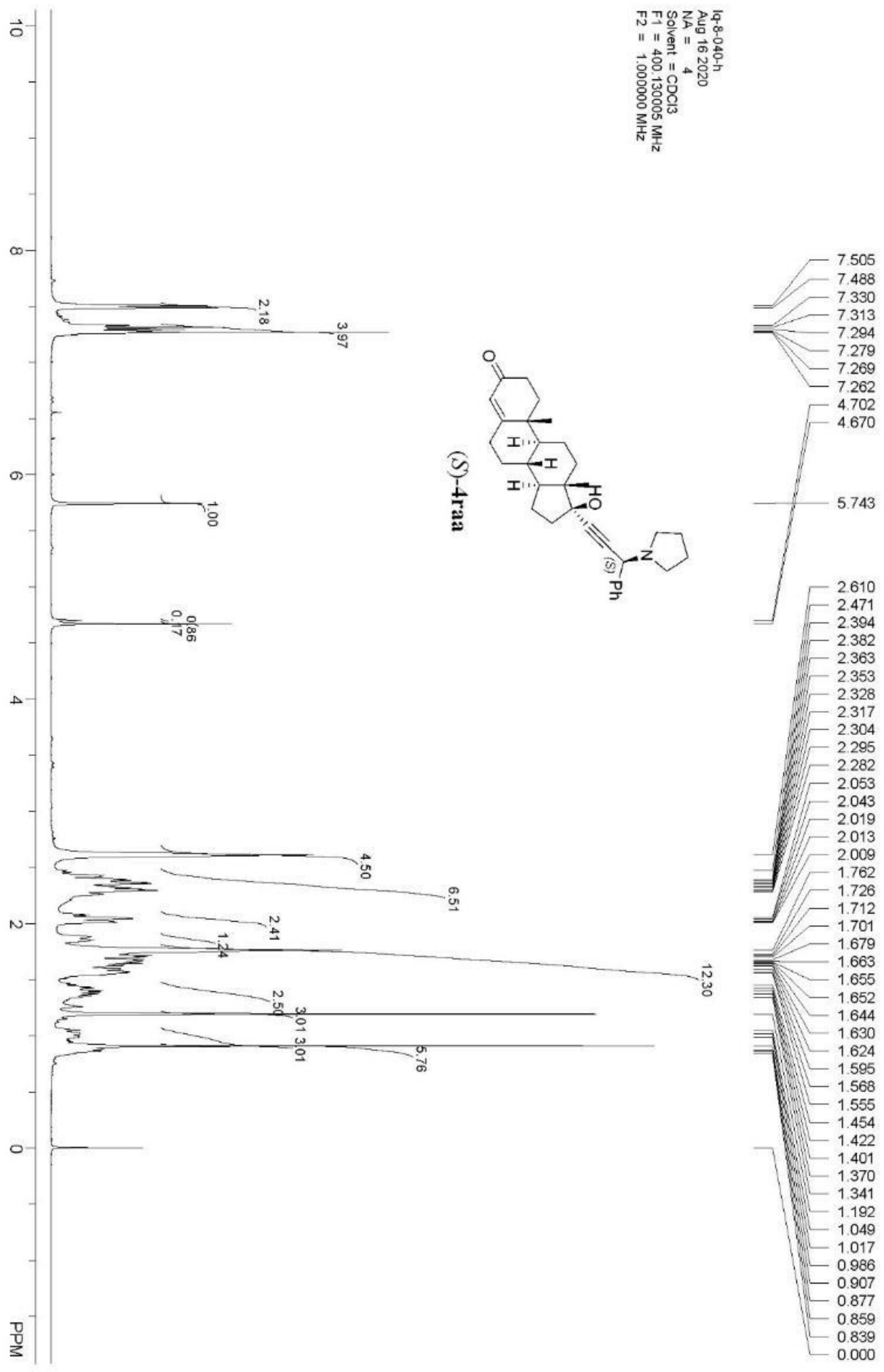

^{1}H NMR (400 MHz, CDCl3) spectrum for (*S*)-4raa

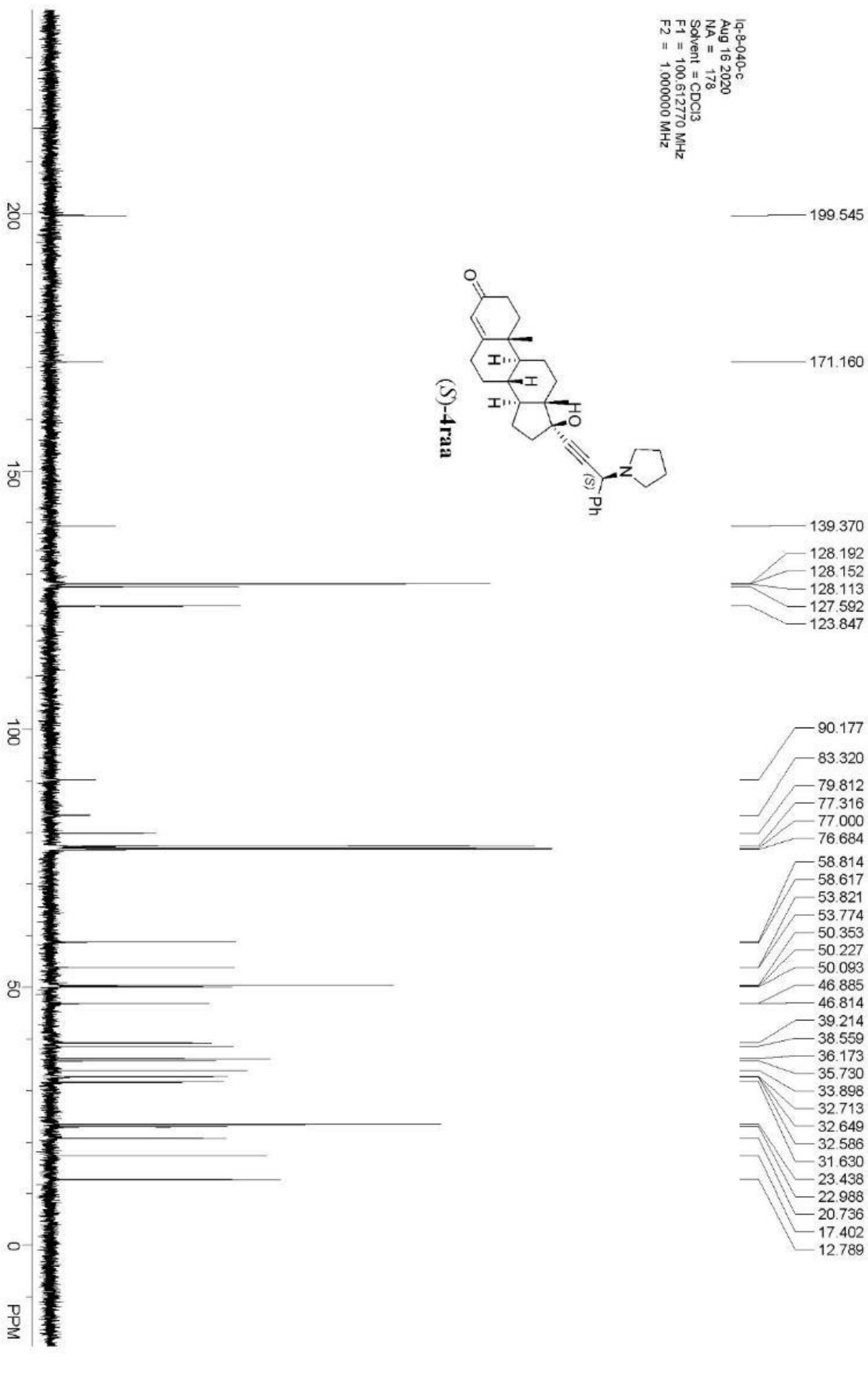

^{13}C NMR (400 MHz, CDCl$_3$) spectrum for (*S*)-4raa

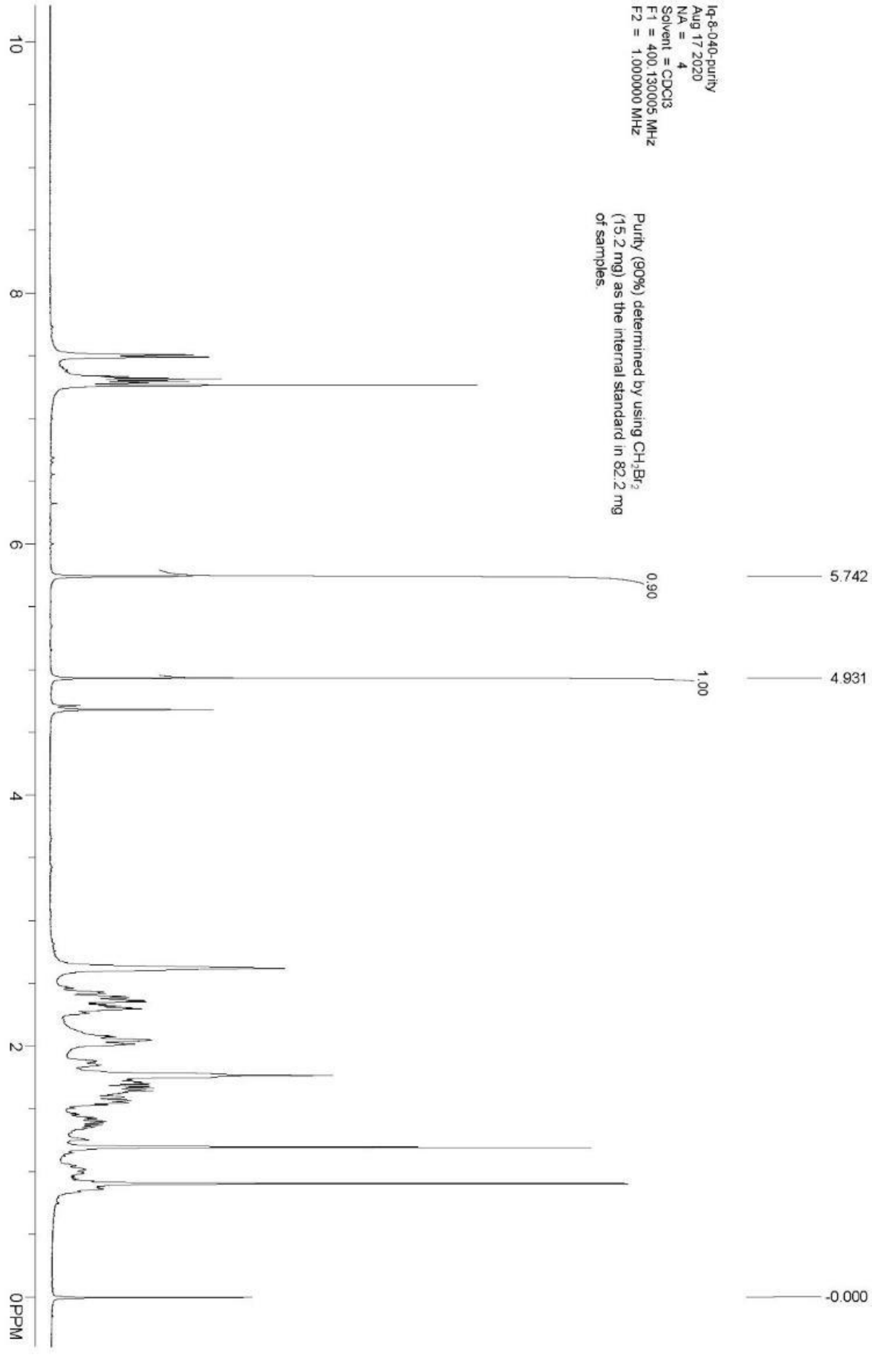

^{1}H NMR (400 MHz, CDCl3) spectrum for (*S*)-4raa

Empower 3 SOFTWARE

Injection Summary Report

SAMPLE INFORMATION

Sample Name:	lq-8-040-oz-h-80-20-0.7-214	Acquired By:	System
Sample Type:	Unknown	Sample Set Name:	
Vial:	1	Acq. Method Set:	HPLC
Injection #:	4	Processing Method:	Default
Injection Volume:	10.00 ul	Channel Name:	W2489 ChA
Run Time:	30.0 Minutes	Proc. Chnl. Descr.:	W2489 ChA 214nm
Date Acquired:	8/18/2020 4:33:50 AM CST		
Date Processed:	8/18/2020 4:56:56 AM CST		

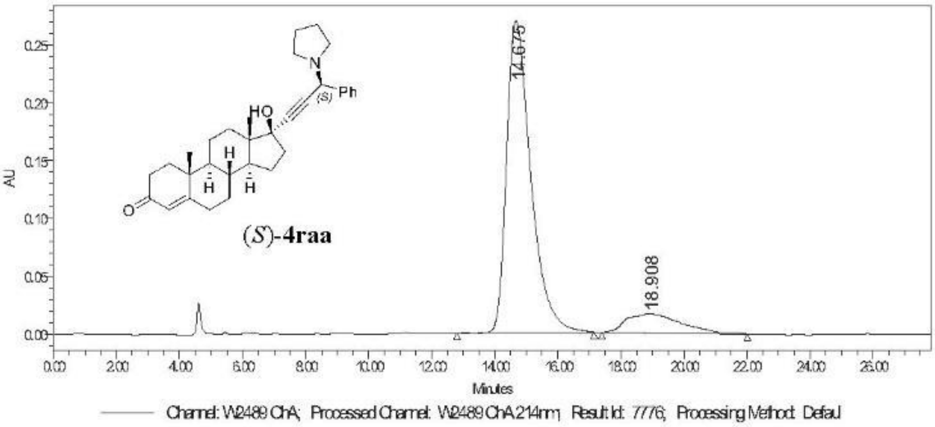

Processed Channel Descr.: W2489 ChA 214nm

	Processed Channel Descr.	RT	Area	%Area	Height
1	W2489 ChA 214nm	14.675	14372157	87.64	269227
2	W2489 ChA 214nm	18.908	2027840	12.36	16511

Reported by User: System
Report Method: Injection Summary Report
Report Method ID: 6865 6865
Page: 1 of 1

Project Name: HPLC
Date Printed:
8/18/2020
6:12:39 AM PRC

HPLC spectrum for (*S*)-4raa

Empower 3 SOFTWARE

Injection Summary Report

SAMPLE INFORMATION

Sample Name:	lq-7-015-oz-h-80-20-0.7-214	Acquired By:	System
Sample Type:	Unknown	Sample Set Name:	
Vial:	1	Acq. Method Set:	HPLC
Injection #:	6	Processing Method:	Default
Injection Volume:	10.00 ul	Channel Name:	W2489 ChA
Run Time:	28.0 Minutes	Proc. Chnl. Descr.:	W2489 ChA 214nm
Date Acquired:	8/18/2020 5:33:35 AM CST		
Date Processed:	8/18/2020 6:11:53 AM CST		

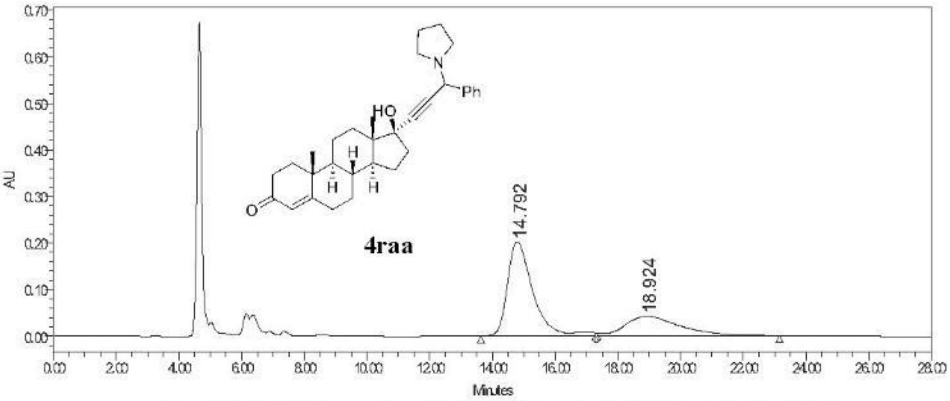

Channel: W2489 ChA; Processed Channel: W2489 ChA 214nm; Result Id: 7786; Processing Method: Defaul

Processed Channel Descr.: W2489 ChA 214nm

	Processed Channel Descr.	RT	Area	%Area	Height
1	W2489 ChA 214nm	14.792	11288026	68.51	202329
2	W2489 ChA 214nm	18.924	5187301	31.49	42308

Reported by User: System
Report Method: Injection Summary Report
Report Method ID: 6865 6865
Page: 1 of 1

Project Name: HPLC
Date Printed:
8/18/2020
6:13:29 AM PRC

HPLC spectrum for (±)-4raa

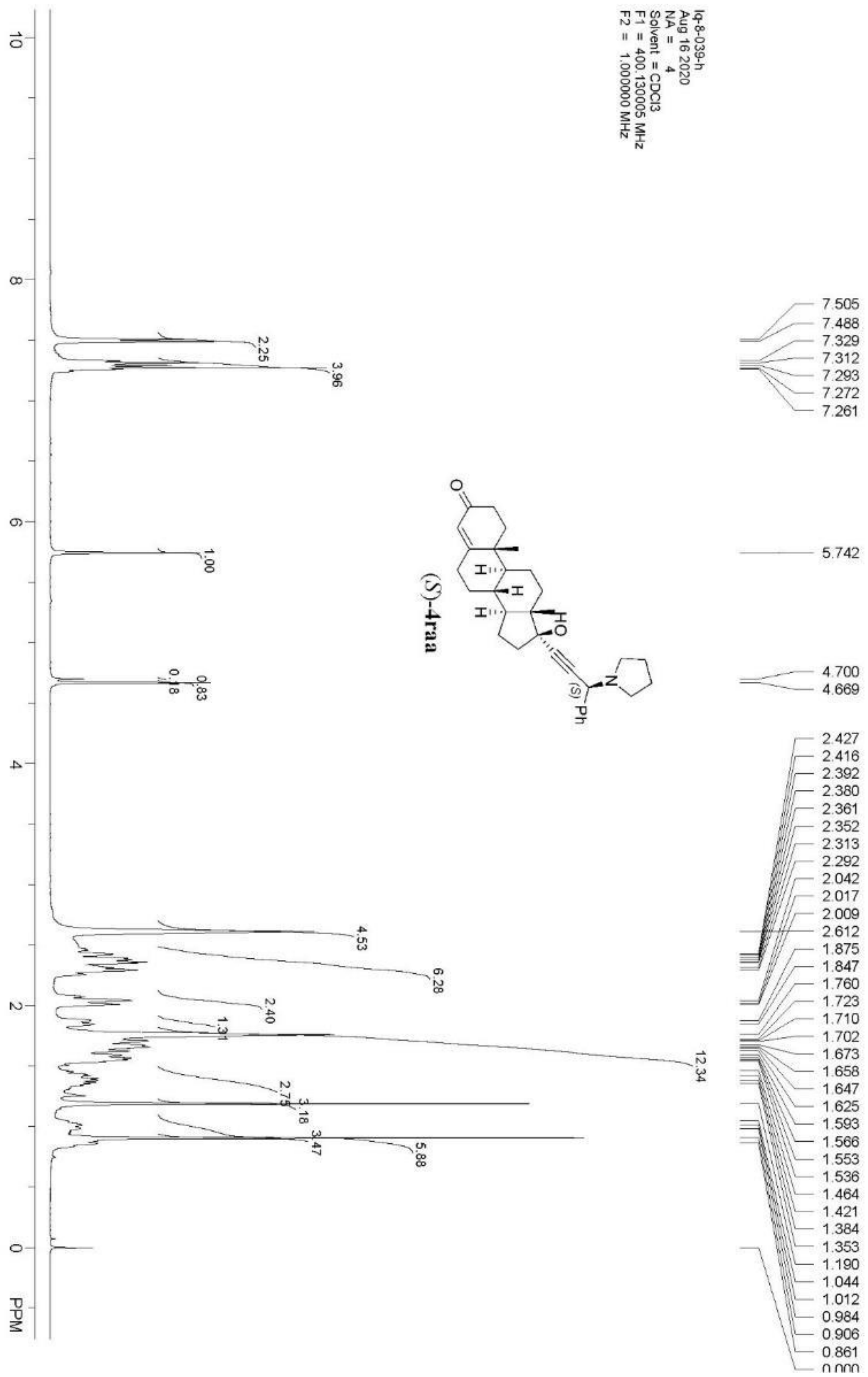

^{1}H NMR (400 MHz, CDCl3) spectrum for (*S*)-4raa

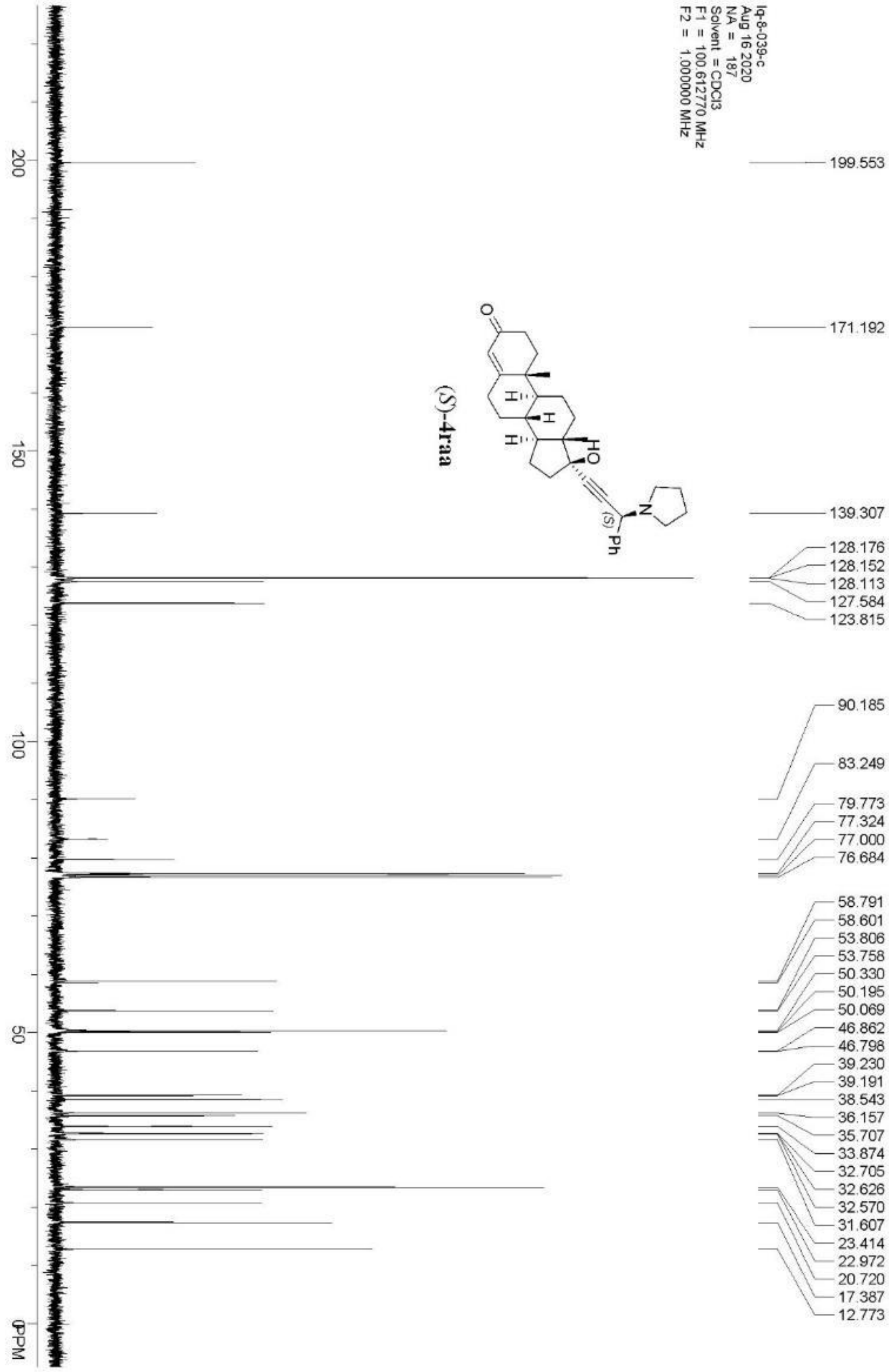

^{13}C NMR (400 MHz, CDCl$_3$) spectrum for (*S*)-4raa

Empower 3 SOFTWARE

Injection Summary Report

SAMPLE INFORMATION

Sample Name:	lq-8-039-oz-h-80-20-0.7-214	Acquired By:	System
Sample Type:	Unknown	Sample Set Name:	
Vial:	1	Acq. Method Set:	HPLC
Injection #:	5	Processing Method:	Default
Injection Volume:	10.00 ul	Channel Name:	W2489 ChA
Run Time:	30.0 Minutes	Proc. Chnl. Descr.:	W2489 ChA 214nm
Date Acquired:	8/17/2020 2:08:03 PM CST		
Date Processed:	8/17/2020 2:59:08 PM CST		

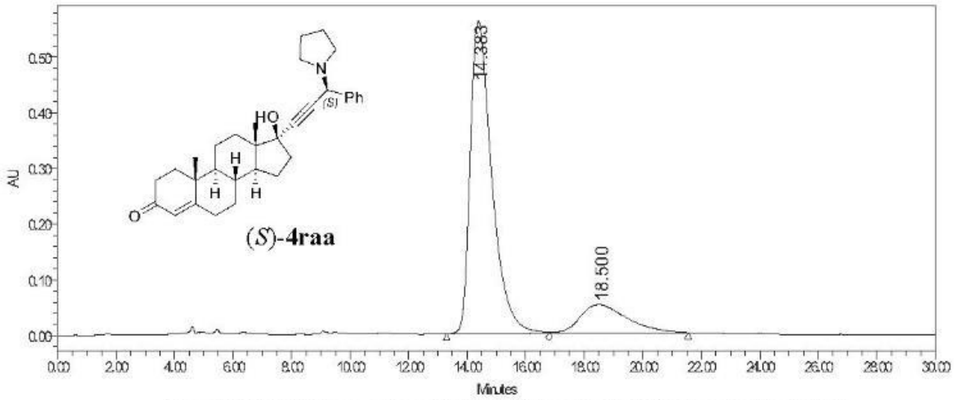

Processed Channel Descr.: W2489 ChA 214nm

	Processed Channel Descr.	RT	Area	%Area	Height
1	W2489 ChA 214nm	14.383	28677103	83.45	561043
2	W2489 ChA 214nm	18.500	5686648	16.55	51309

Reported by User: System
Report Method: Injection Summary Report
Report Method ID: 6865 6865
Page: 1 of 1

Project Name: HPLC
Date Printed:
8/17/2020
3:23:14 PM PRC

HPLC spectrum for (*S*)-4raa

Empower 3 SOFTWARE

Injection Summary Report

SAMPLE INFORMATION

Sample Name:	lq-7-015-oz-h-80-20-0.7-214	Acquired By:	System
Sample Type:	Unknown	Sample Set Name:	
Vial:	1	Acq. Method Set:	HPLC
Injection #:	1	Processing Method:	Default
Injection Volume:	5.00 ul	Channel Name:	W2489 ChA
Run Time:	30.0 Minutes	Proc. Chnl. Descr.:	W2489 ChA 214nm
Date Acquired:	8/17/2020 11:07:41 AM CST		
Date Processed:	8/17/2020 11:37:25 AM CST		

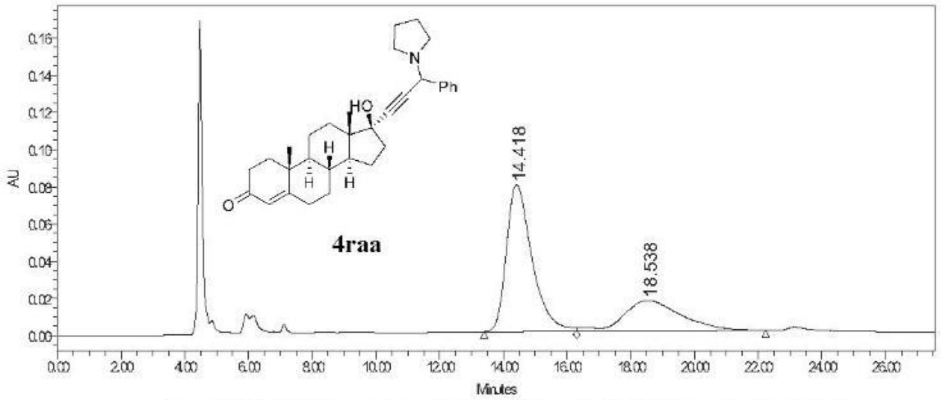

Processed Channel Descr.: W2489 ChA 214nm

	Processed Channel Descr.	RT	Area	%Area	Height
1	W2489 ChA 214nm	14.418	4392298	67.89	78985
2	W2489 ChA 214nm	18.538	2077407	32.11	16416

Reported by User: System
Report Method: Injection Summary Report
Report Method ID: 6865 6865
Page: 1 of 1

Project Name: HPLC
Date Printed:
8/17/2020
11:37:42 AM PRC

HPLC spectrum for (±)-4raa

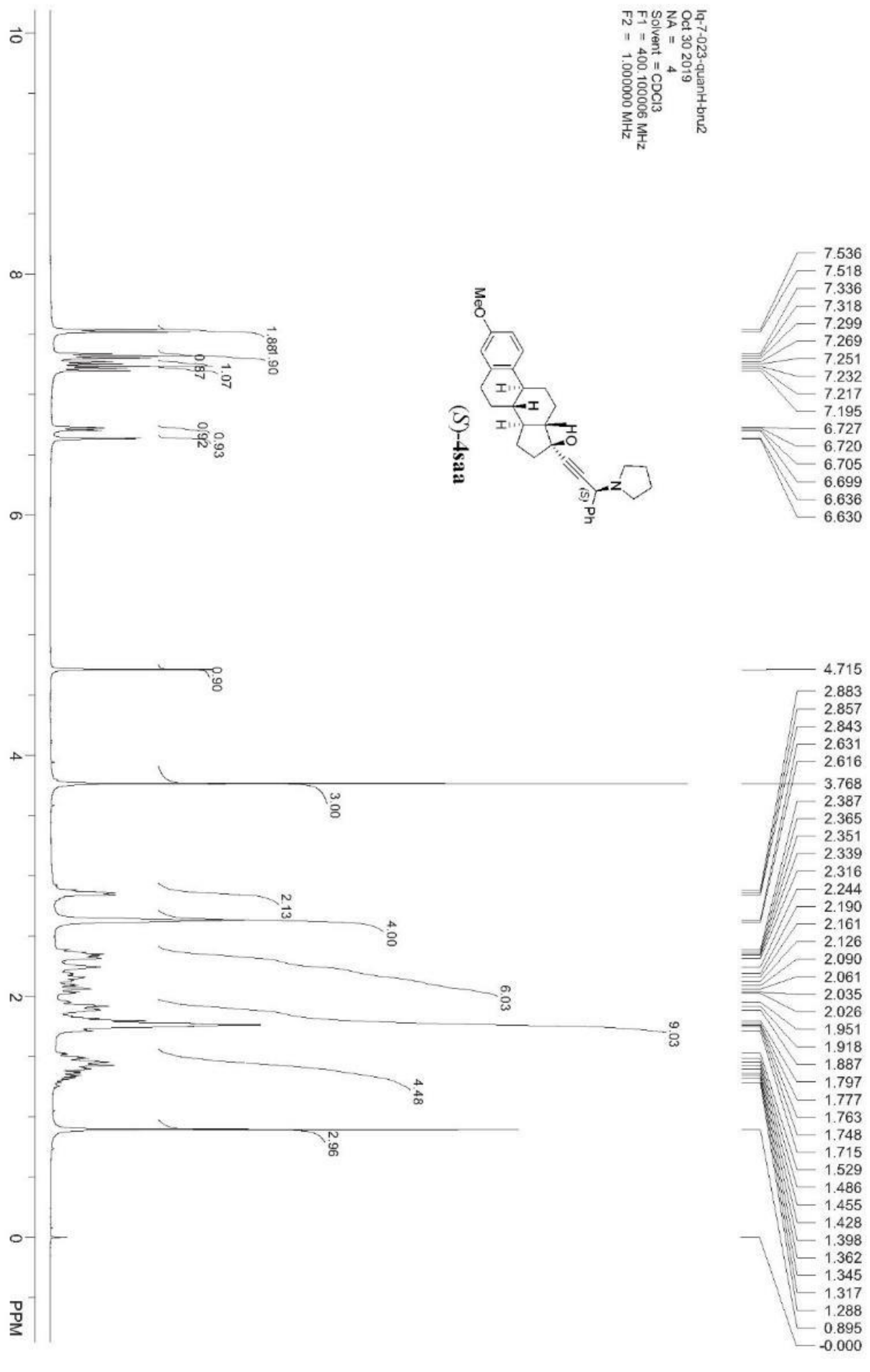

^{1}H NMR (400 MHz, CDCl3) spectrum for (*S*)-**4saa**

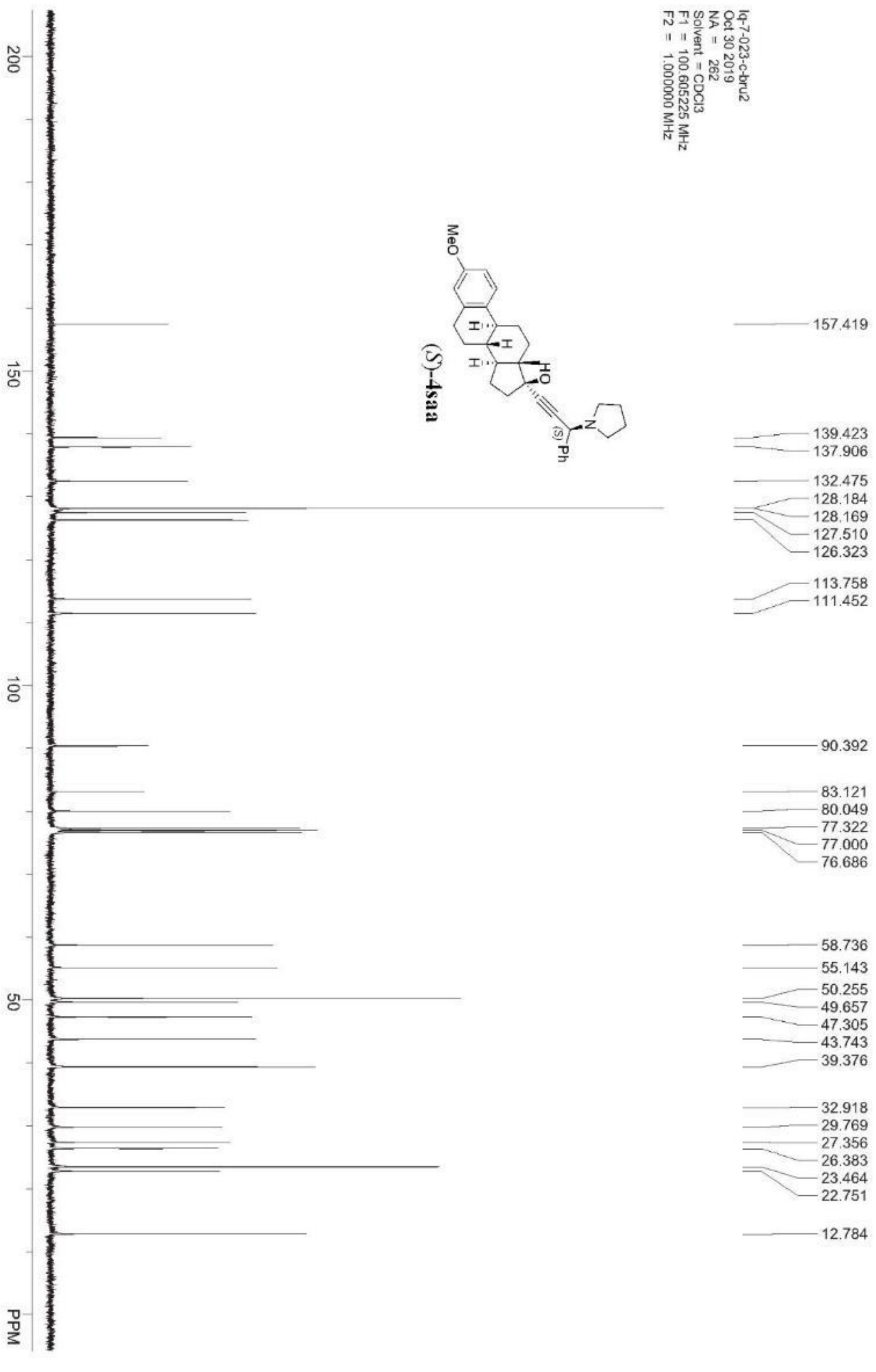

^{13}C NMR (400 MHz, $CDCl_3$) spectrum for (*S*)-4saa

Empower 3 SOFTWARE

Injection Summary Report

SAMPLE INFORMATION

Sample Name:	lq-7-023-ad-h-95-5-1-214	Acquired By:	System
Sample Type:	Unknown	Sample Set Name:	
Vial:	1	Acq. Method Set:	HPLC
Injection #:	2	Processing Method:	Default
Injection Volume:	2.50 ul	Channel Name:	W2489 ChA
Run Time:	20.0 Minutes	Proc. Chnl. Descr.:	W2489 ChA 214nm
Date Acquired:	11/2/2019 1:32:30 PM CST		
Date Processed:	11/2/2019 2:06:53 PM CST		

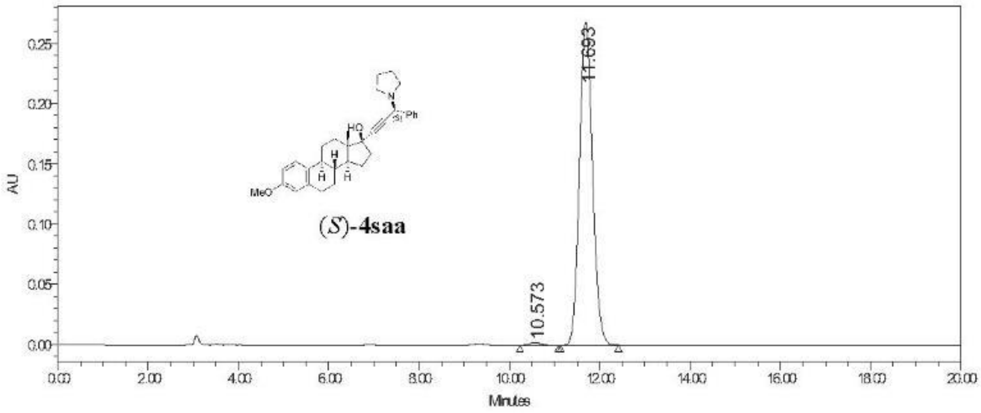

Processed Channel Descr.: W2489 ChA 214nm

	Processed Channel Descr.	RT	Area	%Area	Height
1	W2489 ChA 214nm	10.573	38844	0.72	2122
2	W2489 ChA 214nm	11.693	5372090	99.28	268326

Reported by User: System
Report Method: Injection Summary Report
Report Method ID: 1639 1639
Page: 1 of 1

Project Name: HPLC
Date Printed:
11/2/2019
2:07:07 PM PRC

HPLC spectrum for (*S*)-4saa

Empower™ 3 SOFTWARE

Injection Summary Report

SAMPLE INFORMATION

Sample Name:	lq-7-020-ad-h-95-5-1-214	Acquired By:	System
Sample Type:	Unknown	Sample Set Name:	
Vial:	1	Acq. Method Set:	HPLC
Injection #:	1	Processing Method:	Default
Injection Volume:	5.00 ul	Channel Name:	W2489 ChA
Run Time:	120.0 Minutes	Proc. Chnl. Descr.:	W2489 ChA 214nm
Date Acquired:	11/2/2019 11:25:05 AM CST		
Date Processed:	11/2/2019 2:06:14 PM CST		

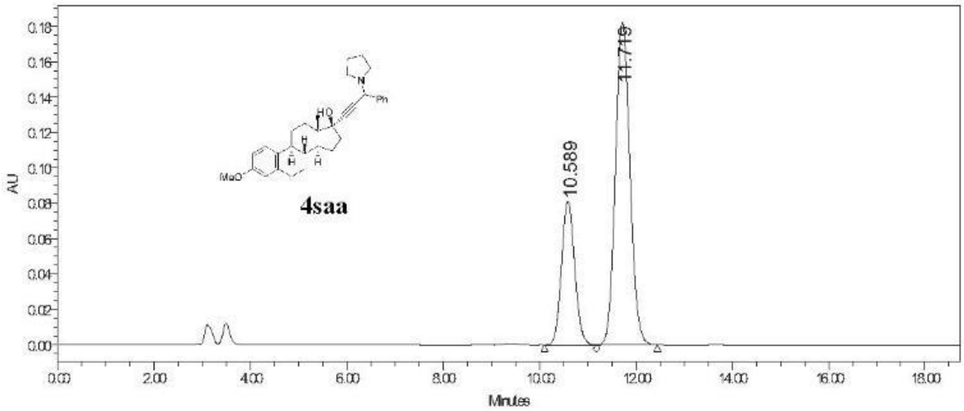

Channel: W2489 ChA; Processed Channel: W2489 ChA 214nm; Result Id: 5847; Processing Method: Defaul

Processed Channel Descr.: W2489 ChA 214nm

	Processed Channel Descr.	RT	Area	%Area	Height
1	W2489 ChA 214nm	10.589	1541259	28.81	81430
2	W2489 ChA 214nm	11.719	3808215	71.19	182503

Reported by User: System
Report Method: Injection Summary Report
Report Method ID: 1639 1639
Page: 1 of 1

Project Name: HPLC
Date Printed:
11/2/2019
2:07:50 PM PRC

HPLC spectrum for (±)-4saa

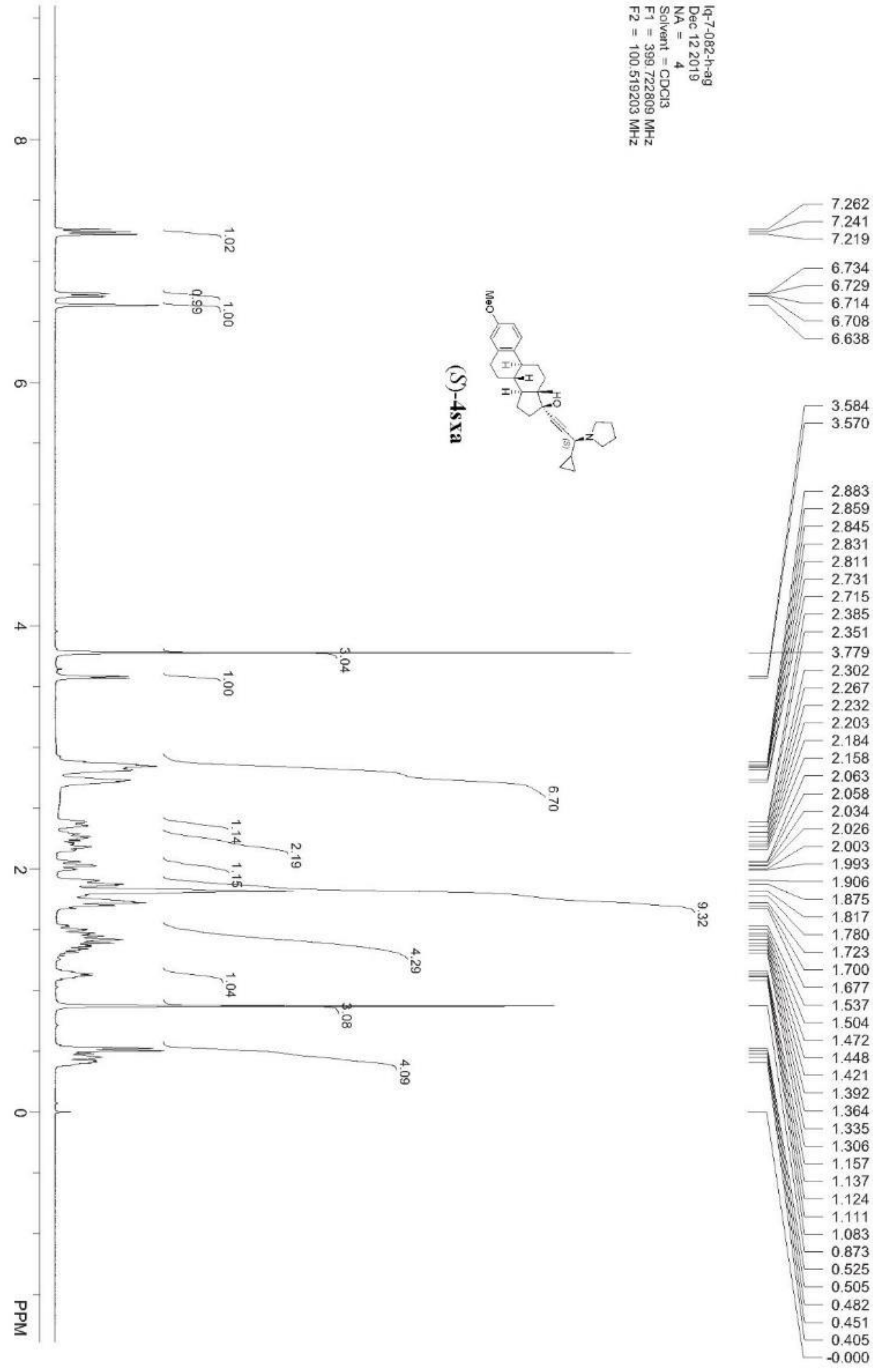

^{1}H NMR (400 MHz, CDCl3) spectrum for (*S*)**-4sxa**

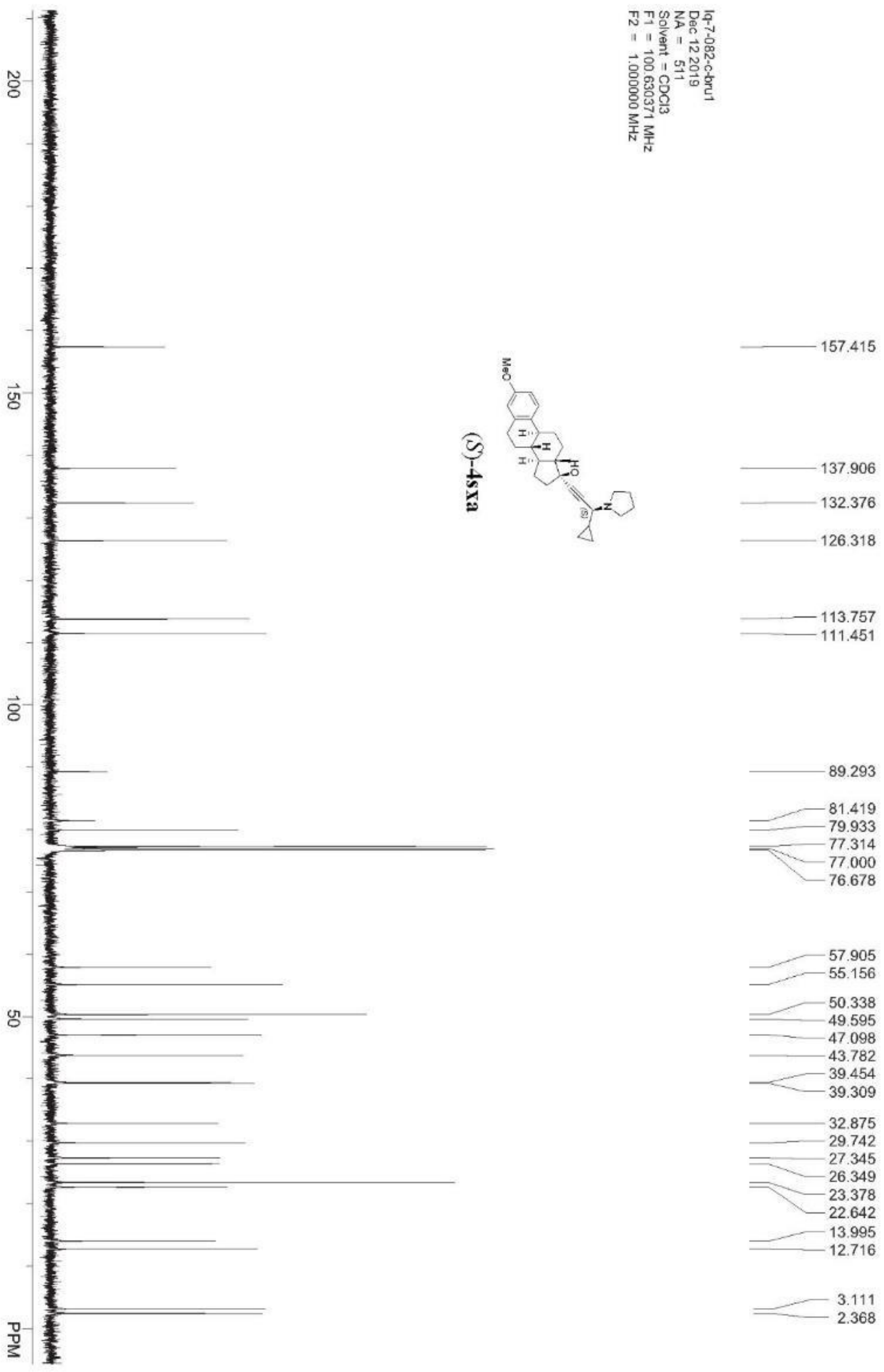

^{13}C NMR (400 MHz, CDCl$_3$) spectrum for (*S*)-4sxa

Empower 3 SOFTWARE

Injection Summary Report

SAMPLE INFORMATION

Sample Name:	lq-7-082-ad-h-90-10-0.5-214	Acquired By:	System
Sample Type:	Unknown	Sample Set Name:	
Vial:	1	Acq. Method Set:	HPLC
Injection #:	1	Processing Method:	Default
Injection Volume:	5.00 ul	Channel Name:	W2489 ChA
Run Time:	25.0 Minutes	Proc. Chnl. Descr.:	W2489 ChA 214nm
Date Acquired:	12/13/2019 11:27:18 AM CST		
Date Processed:	12/13/2019 1:20:29 PM CST		

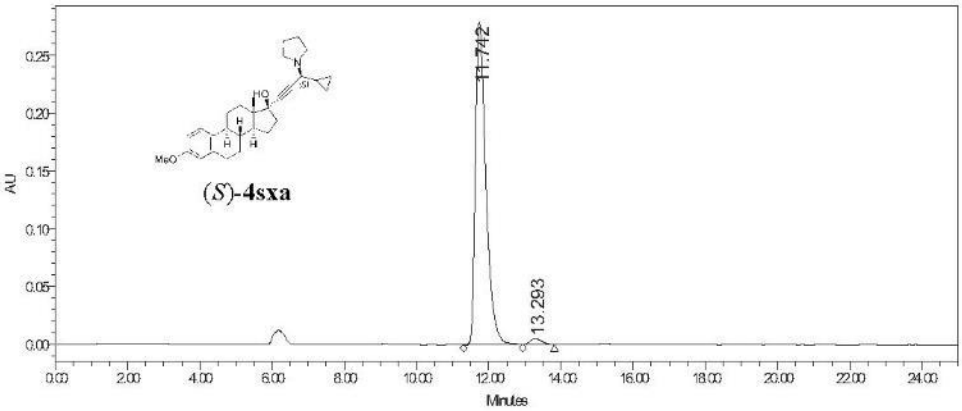

Channel: W2489 ChA; Processed Channel: W2489 ChA 214nm; Result Id: 6394; Processing Method: Defaul

Processed Channel Descr.: W2489 ChA 214nm

	Processed Channel Descr.	RT	Area	%Area	Height
1	W2489 ChA 214nm	11.742	5632740	98.07	279410
2	W2489 ChA 214nm	13.293	110652	1.93	5016

Reported by User: System
Report Method: Injection Summary Report
Report Method ID: 1639 1639
Page: 1 of 1

Project Name: HPLC
Date Printed:
12/13/2019
1:20:39 PM PRC

HPLC spectrum for (*S*)-4sxa

Empower 3 SOFTWARE

Injection Summary Report

SAMPLE INFORMATION

Sample Name:	lq-7-074-ad-h-90-10-0.5-214	Acquired By:	System
Sample Type:	Unknown	Sample Set Name:	
Vial:	1	Acq. Method Set:	HPLC
Injection #:	4	Processing Method:	Default
Injection Volume:	5.00 ul	Channel Name:	W2489 ChA
Run Time:	20.0 Minutes	Proc. Chnl. Descr.:	W2489 ChA 214nm
Date Acquired:	12/13/2019 12:46:33 PM CST		
Date Processed:	12/13/2019 1:19:46 PM CST		

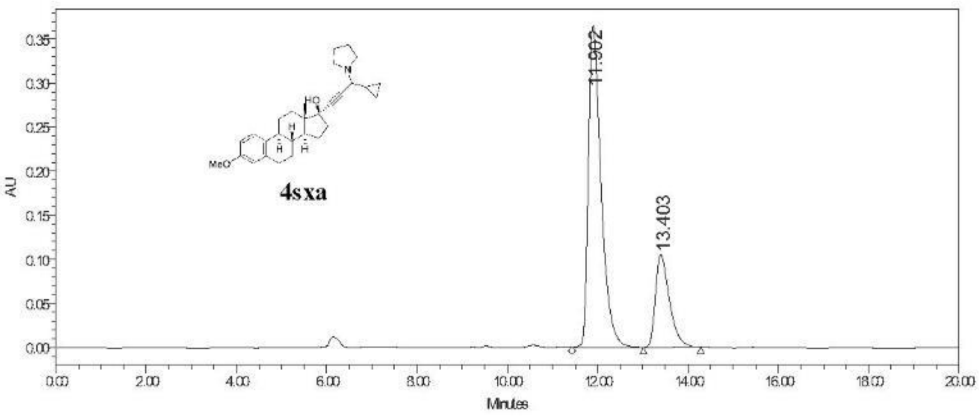

Processed Channel Descr.: W2489 ChA 214nm

	Processed Channel Descr.	RT	Area	%Area	Height
1	W2489 ChA 214nm	11.902	7127543	76.08	364883
2	W2489 ChA 214nm	13.403	2241392	23.92	104772

Reported by User: System
Report Method: Injection Summary Report
Report Method ID: 1639 1639
Page: 1 of 1

Project Name: HPLC
Date Printed:
12/13/2019
1:21:13 PM PRC

HPLC spectrum for (±)-4sxa

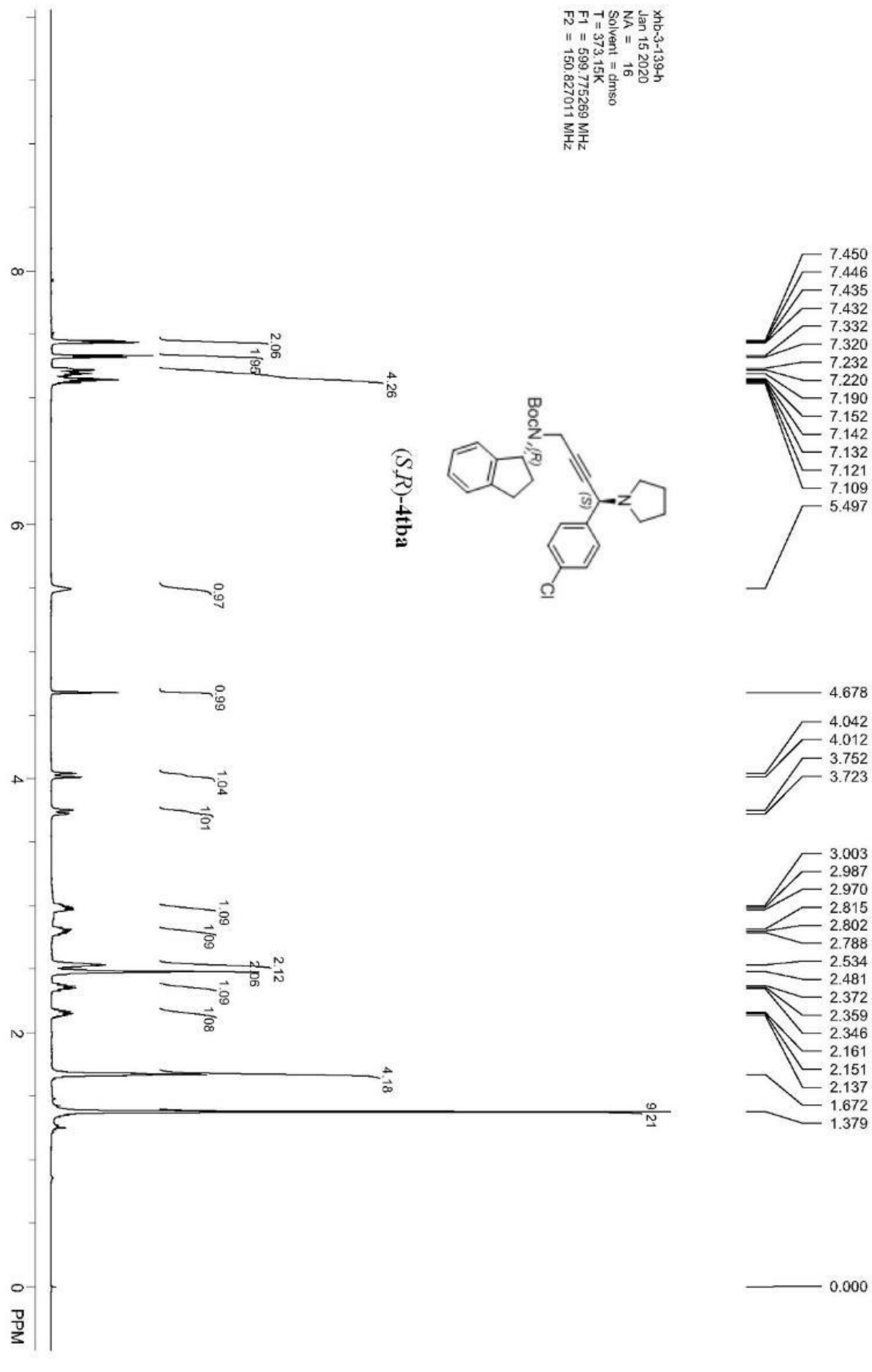

^{1}H NMR (400 MHz, d_6-DMSO, 373 K) spectrum for (*S*,*R*)**-4tba**

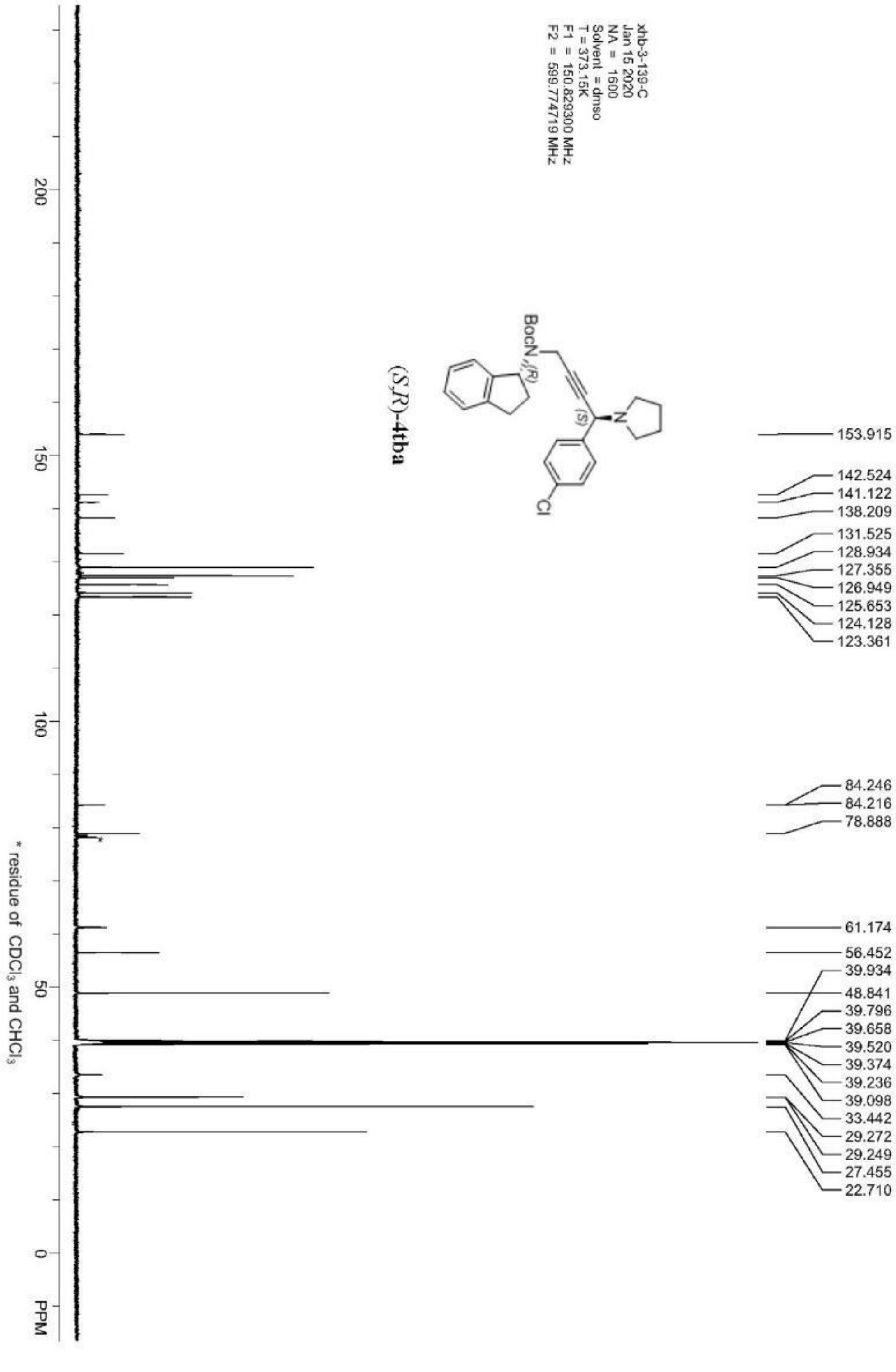

^{13}C NMR (400 MHz, *d*$_6$-DMSO, 373 K) spectrum for (*S*,*R*)**-4tba**

Empower 3 SOFTWARE

Injection Summary Report

SAMPLE INFORMATION

Sample Name:	xhb-3-139-ad-h-100-1-1-214	Acquired By:	System
Sample Type:	Unknown	Sample Set Name:	
Vial:	1	Acq. Method Set:	HPLC
Injection #:	2	Processing Method:	LC PQ
Injection Volume:	2.50 ul	Channel Name:	W2489 ChA
Run Time:	30.0 Minutes	Proc. Chnl. Descr.:	W2489 ChA 214nm
Date Acquired:	1/11/2020 11:06:15 AM CST		
Date Processed:	1/11/2020 11:32:05 AM CST		

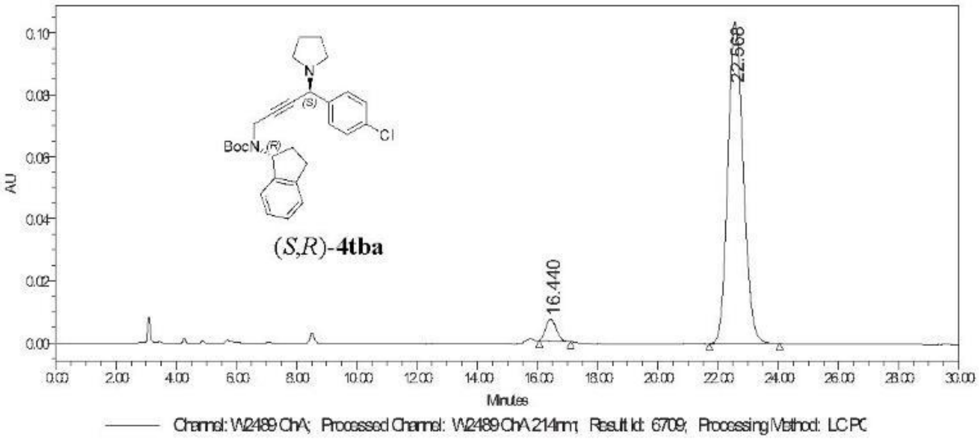

Processed Channel Descr.: W2489 ChA 214nm

	Processed Channel Descr.	RT	Area	%Area	Height
1	W2489 ChA 214nm	16.440	170638	4.42	7036
2	W2489 ChA 214nm	22.568	3689510	95.58	103662

HPLC spectrum for (*S*,*R*)-4tba

Empower™ 3 SOFTWARE

Injection Summary Report

SAMPLE INFORMATION

Sample Name:	xhb-3-138-ad-h-100-1-1-214	Acquired By:	System
Sample Type:	Unknown	Sample Set Name:	
Vial:	1	Acq. Method Set:	HPLC
Injection #:	3	Processing Method:	LC PQ
Injection Volume:	5.00 ul	Channel Name:	W2489 ChA
Run Time:	30.0 Minutes	Proc. Chnl. Descr.:	W2489 ChA 214nm
Date Acquired:	1/11/2020 11:36:37 AM CST		
Date Processed:	1/19/2020 11:37:33 AM CST		

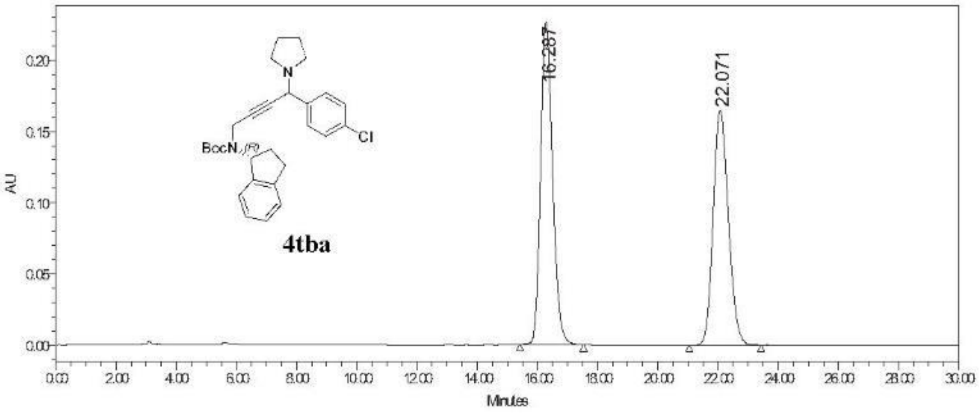

Channel: W2489 ChA; Processed Channel: W2489 ChA 214nm; Result Id: 6760; Processing Method: LC PC

Processed Channel Descr.: W2489 ChA 214nm

	Processed Channel Descr.	RT	Area	%Area	Height
1	W2489 ChA 214nm	16.287	6235110	51.88	226515
2	W2489 ChA 214nm	22.071	5782298	48.12	164884

Reported by User: System
Report Method: Injection Summary Report
Report Method ID: 1639 1639
Page: 1 of 1

Project Name: HPLC
Date Printed:
1/19/2020
11:37:54 AM PRC

HPLC spectrum for (*R*)-4tba

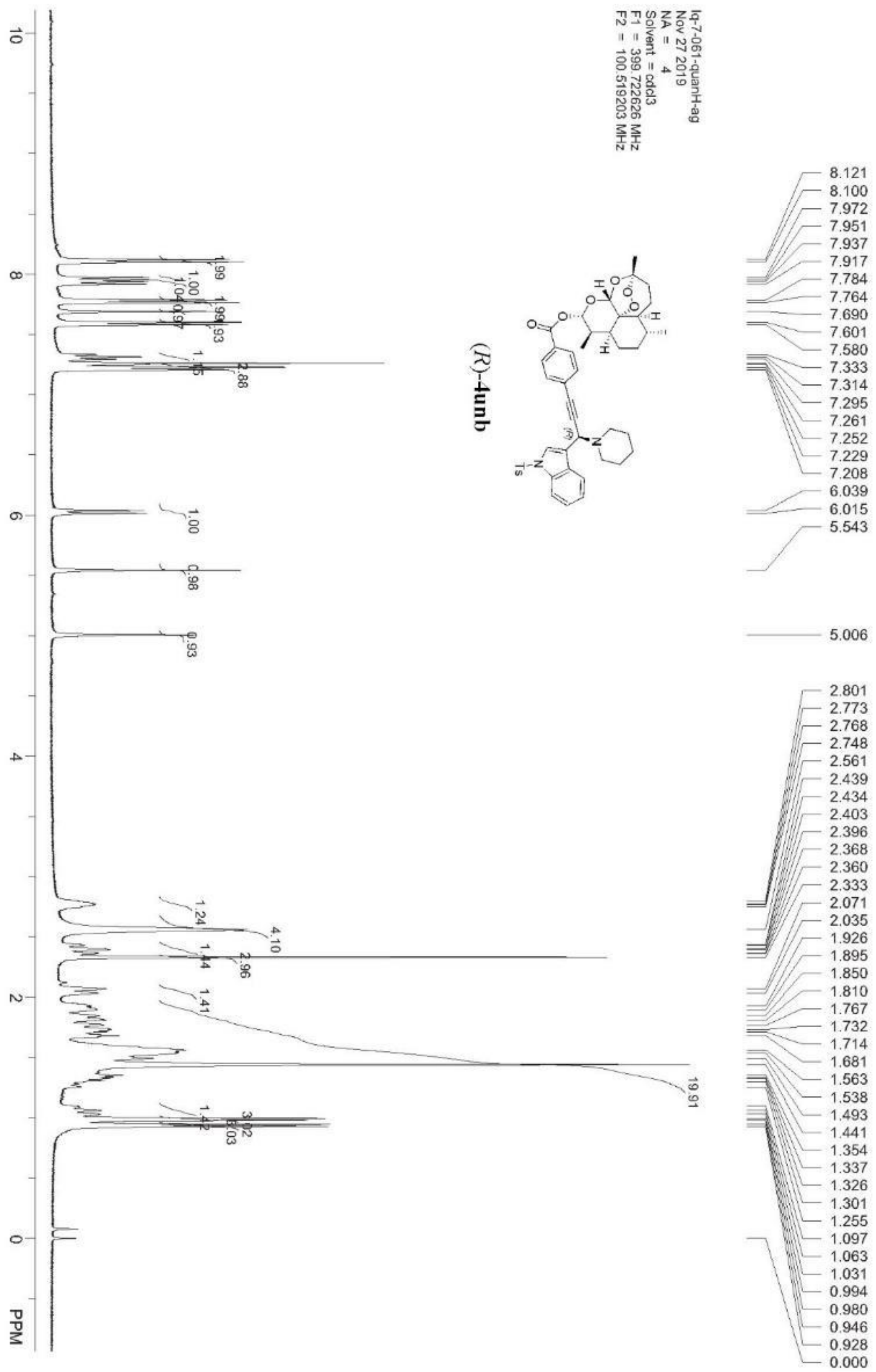

^{1}H NMR (400 MHz, CDCl3) spectrum for (*R*)**-4unb**

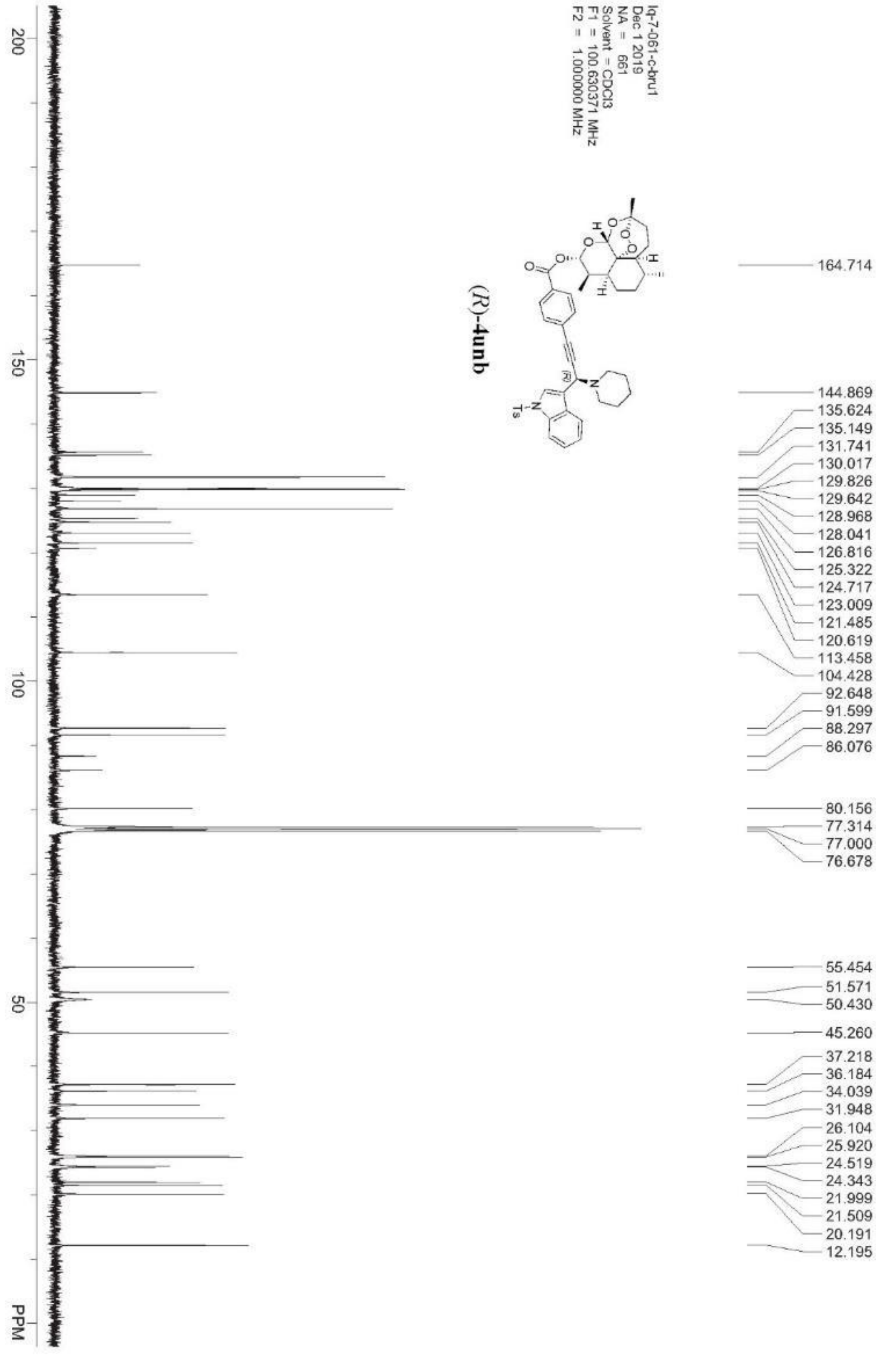

^{13}C NMR (400 MHz, CDCl$_3$) spectrum for (*R*)-4unb

Empower™ 3 SOFTWARE

Injection Summary Report

SAMPLE INFORMATION

Sample Name:	lq-7-061-od-h-90-10-1-214	Acquired By:	System
Sample Type:	Unknown	Sample Set Name:	
Vial:	1	Acq. Method Set:	HPLC
Injection #:	3	Processing Method:	Default
Injection Volume:	4.00 ul	Channel Name:	W2489 ChA
Run Time:	80.0 Minutes	Proc. Chnl. Descr.:	W2489 ChA 214nm
Date Acquired:	12/6/2019 3:12:28 PM CST		
Date Processed:	12/7/2019 12:58:22 PM CST		

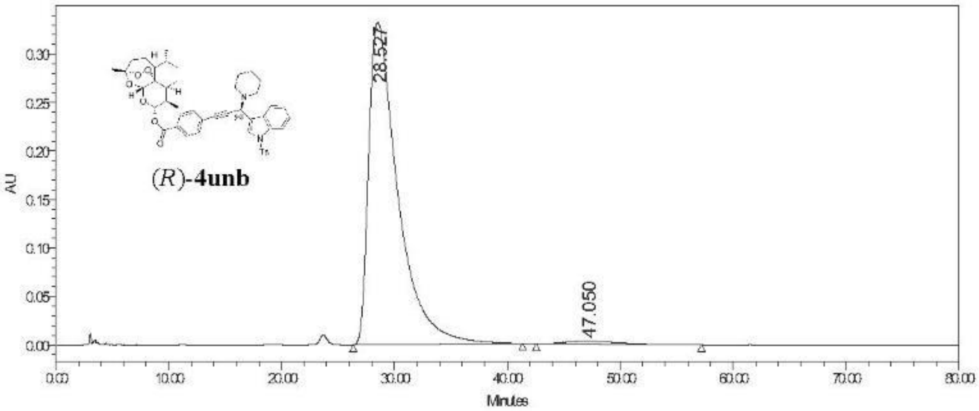

Processed Channel Descr.: W2489 ChA 214nm

	Processed Channel Descr.	RT	Area	% Area	Height
1	W2489 ChA 214nm	28.527	59697145	98.30	332683
2	W2489 ChA 214nm	47.050	1034798	1.70	3165

Reported by User: System
Report Method: Injection Summary Report
Report Method ID: 1639 1639
Page: 1 of 1

Project Name: HPLC
Date Printed:
12/7/2019
1:00:21 PM PRC

HPLC spectrum for (*R*)-4unb

Empower™ 3 SOFTWARE

Injection Summary Report

SAMPLE INFORMATION

Sample Name:	lq-7-061-066-od-h-90-10-1-214	Acquired By:	System
Sample Type:	Unknown	Sample Set Name:	
Vial:	1	Acq. Method Set:	HPLC
Injection #:	1	Processing Method:	Default
Injection Volume:	5.00 ul	Channel Name:	W2489 ChA
Run Time:	90.0 Minutes	Proc. Chnl. Descr.:	W2489 ChA 214nm
Date Acquired:	12/6/2019 11:15:53 PM CST		
Date Processed:	12/7/2019 12:57:41 PM CST		

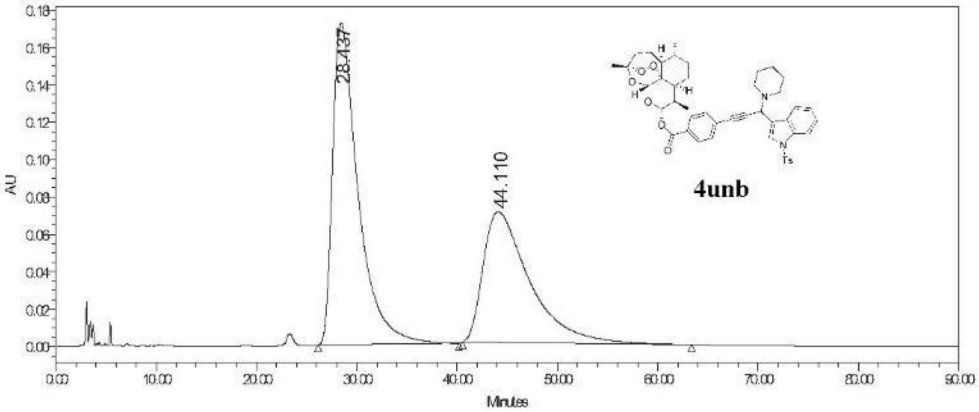

Channel: W2489 ChA; Processed Channel: W2489 ChA 214nm; Result Id: 6303; Processing Method: Defaul

Processed Channel Descr.: W2489 ChA 214nm

	Processed Channel Descr.	RT	Area	% Area	Height
1	W2489 ChA 214nm	28.437	30324204	57.33	172080
2	W2489 ChA 214nm	44.110	22574040	42.67	69958

Reported by User: System
Report Method: Injection Summary Report
Report Method ID: 1639 1639
Page: 1 of 1

Project Name: HPLC
Date Printed:
12/7/2019
12:59:55 PM PRC

HPLC spectrum for (±)-4unb

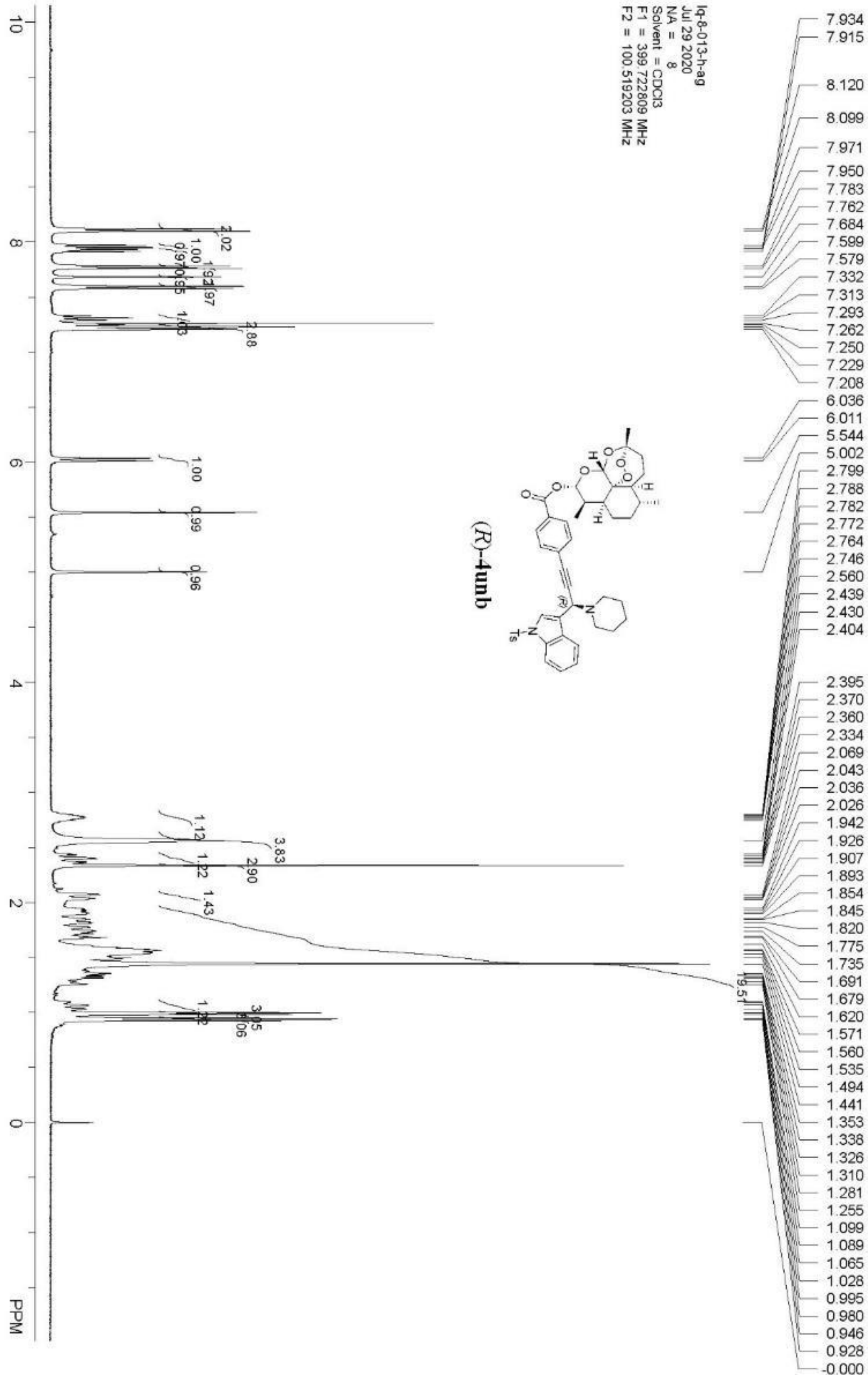

^{1}H NMR (400 MHz, CDCl3) spectrum for *(R)*-**4unb**

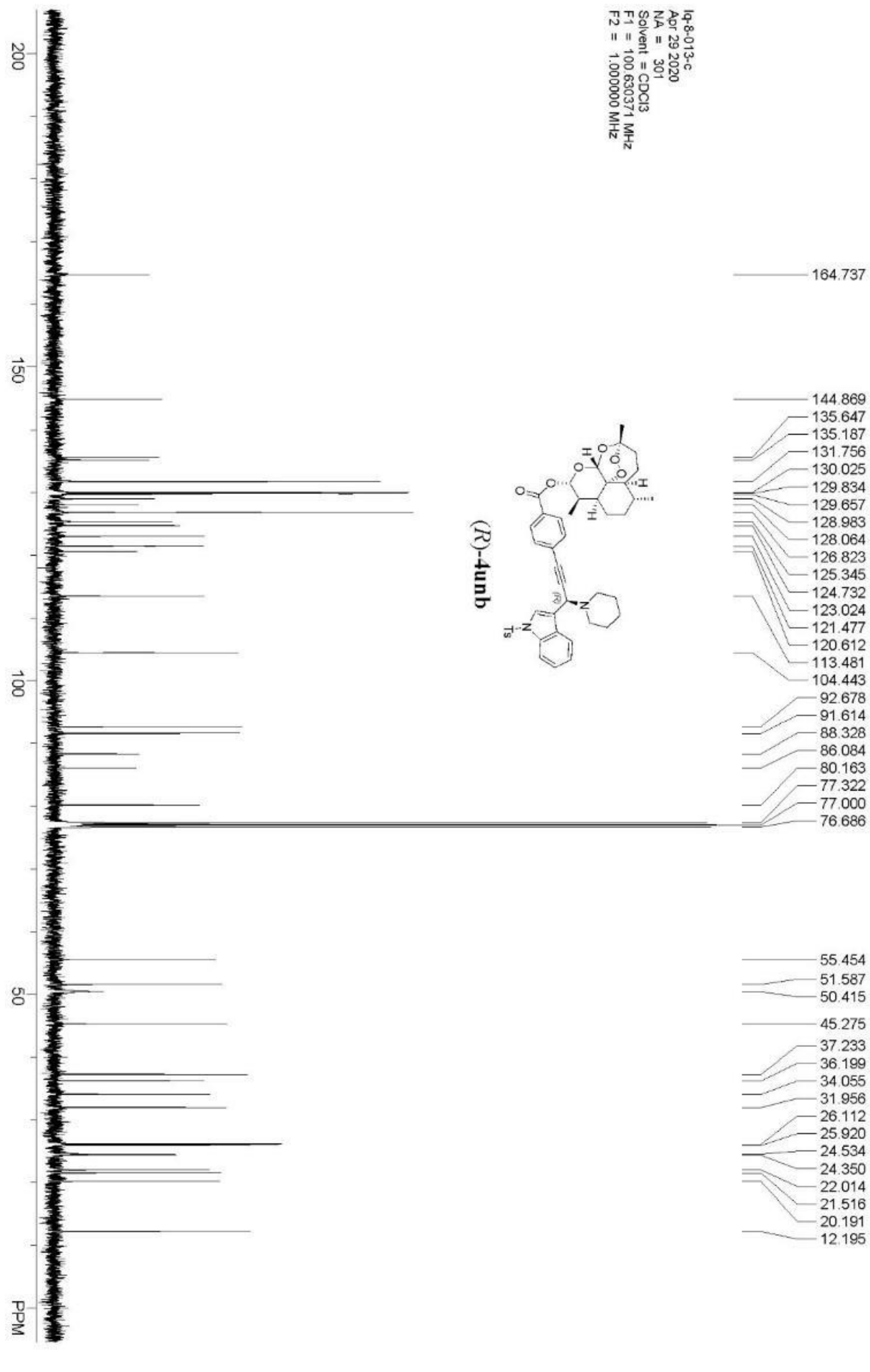

^{13}C NMR (400 MHz, CDCl$_3$) spectrum for (*R*)-4unb

Empower 3 SOFTWARE

Injection Summary Report

SAMPLE INFORMATION

Sample Name:	lq-8-013-od-h-90-10-1-214	Acquired By:	System
Sample Type:	Unknown	Sample Set Name:	
Vial:	1	Acq. Method Set:	HPLC
Injection #:	1	Processing Method:	Default
Injection Volume:	5.00 ul	Channel Name:	W2489 ChA
Run Time:	90.0 Minutes	Proc. Chnl. Descr.:	W2489 ChA 214nm
Date Acquired:	7/29/2020 5:47:33 AM CST		
Date Processed:	7/29/2020 11:00:24 AM CST		

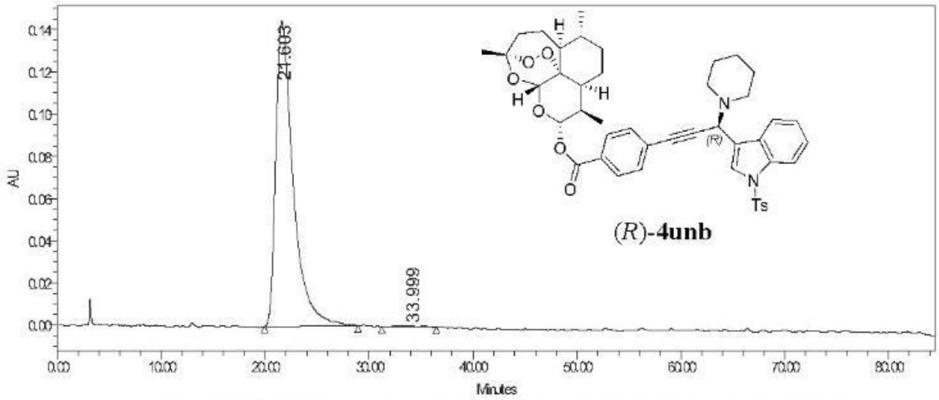

Channel: W2489 ChA; Processed Channel: W2489 ChA 214nm; Result Id: 7442; Processing Method: Defaul

Processed Channel Descr.: W2489 ChA 214nm

	Processed Channel Descr.	RT	Area	%Area	Height
1	W2489 ChA 214nm	21.603	16792912	99.37	144404
2	W2489 ChA 214nm	33.999	105638	0.63	691

Reported by User: System
Report Method: Injection Summary Report
Report Method ID: 6865 6865
Page: 1 of 1

Project Name: HPLC
Date Printed:
7/30/2020
2:09:44 AM PRC

HPLC spectrum for (*R*)-4unb

Empower 3

Injection Summary Report

SAMPLE INFORMATION

Sample Name:	lq-8-013-mixed-od-h-90-10-1-214	Acquired By:	System
Sample Type:	Unknown	Sample Set Name:	
Vial:	1	Acq. Method Set:	HPLC
Injection #:	1	Processing Method:	Default
Injection Volume:	10.00 ul	Channel Name:	W2489 ChA
Run Time:	90.0 Minutes	Proc. Chnl. Descr.:	W2489 ChA 214nm
Date Acquired:	7/29/2020 4:27:22 AM CST		
Date Processed:	7/29/2020 9:48:18 AM CST		

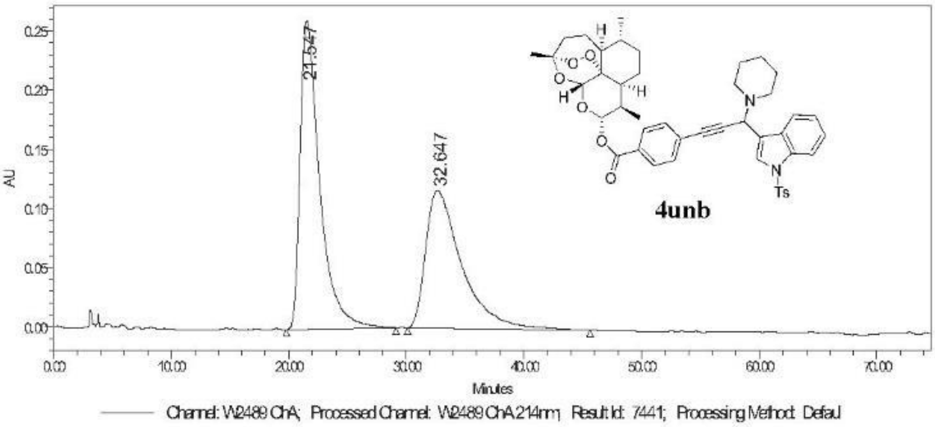

Processed Channel Descr.: W2489 ChA 214nm

	Processed Channel Descr.	RT	Area	%Area	Height
1	W2489 ChA 214nm	21.547	30142474	54.94	260508
2	W2489 ChA 214nm	32.647	24723288	45.06	116717

Reported by User: System
Report Method: Injection Summary Report
Report Method ID: 6865 6865
Page: 1 of 1

Project Name: HPLC
Date Printed:
7/30/2020
2:09:22 AM PRC

HPLC spectrum for (±)-4unb

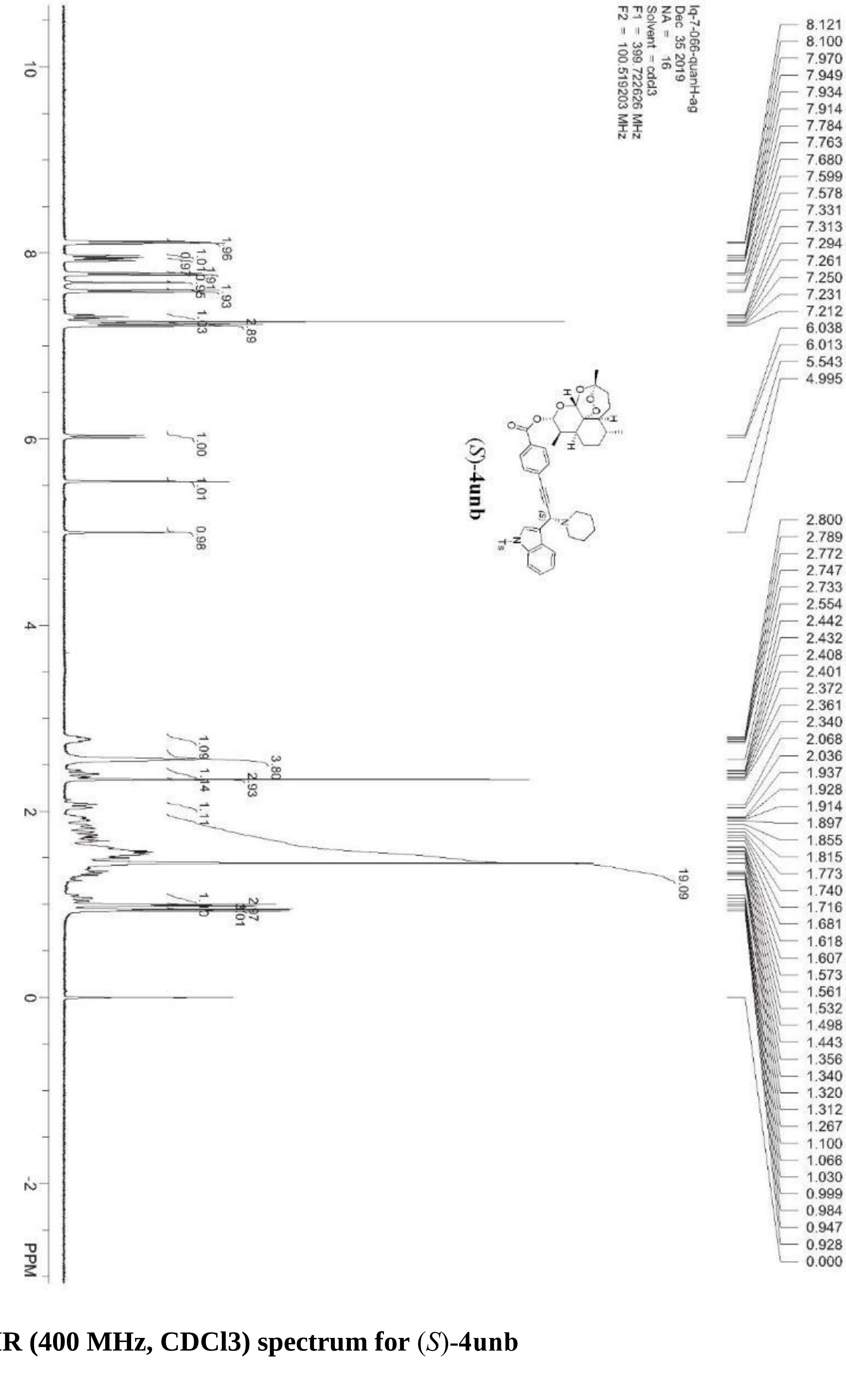

^{1}H NMR (400 MHz, CDCl3) spectrum for (*S*)-4unb

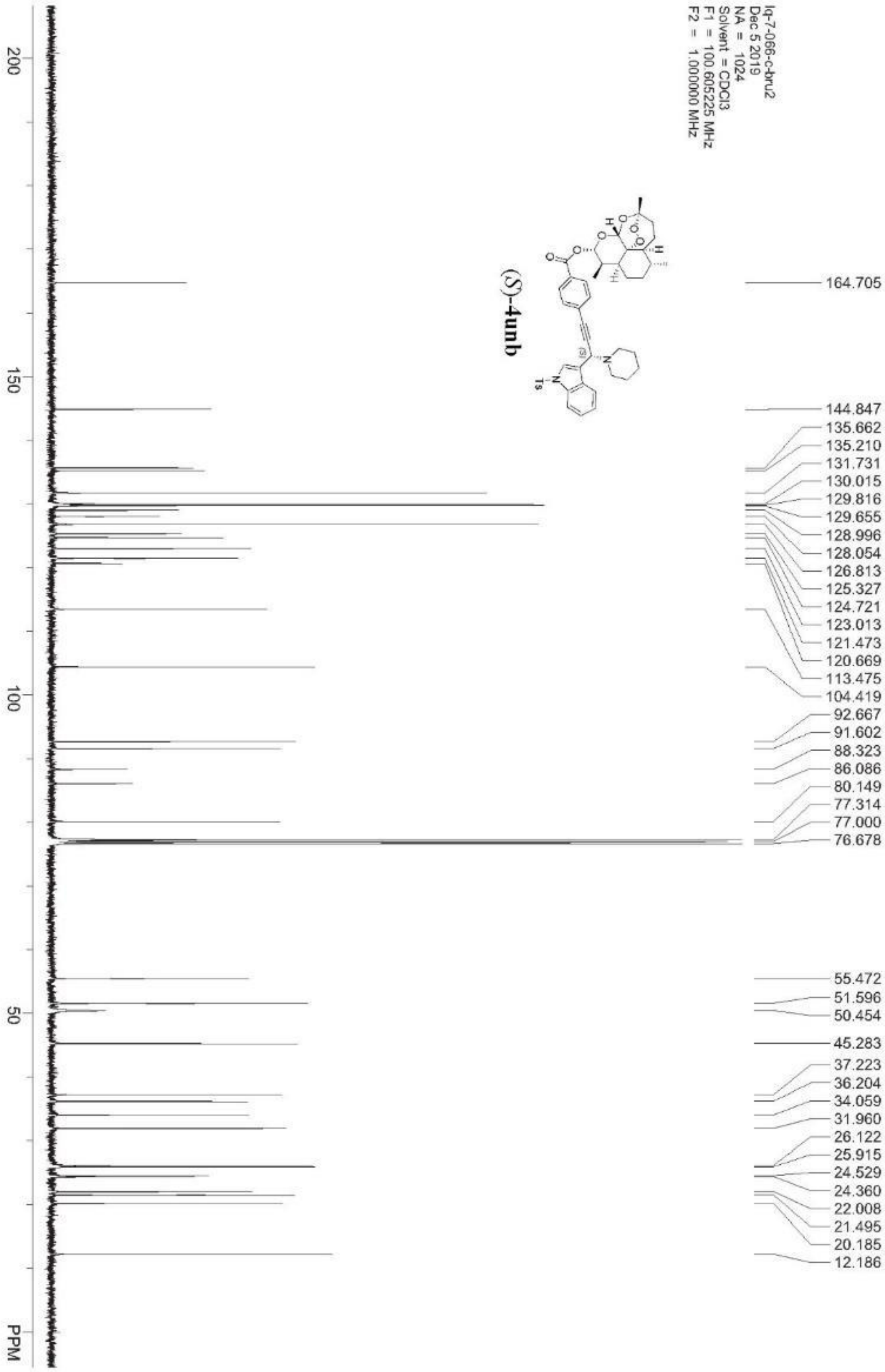

^{13}C NMR (400 MHz, CDCl$_3$) spectrum for (*S*)-4unb

Empower 3 SOFTWARE

Injection Summary Report

SAMPLE INFORMATION

Sample Name:	lq-7-066-od-h-90-10-1-214	Acquired By:	System
Sample Type:	Unknown	Sample Set Name:	
Vial:	1	Acq. Method Set:	HPLC
Injection #:	2	Processing Method:	Default
Injection Volume:	7.50 ul	Channel Name:	W2489 ChA
Run Time:	80.0 Minutes	Proc. Chnl. Descr.:	W2489 ChA 214nm
Date Acquired:	12/6/2019 1:43:03 PM CST		
Date Processed:	12/7/2019 12:58:57 PM CST		

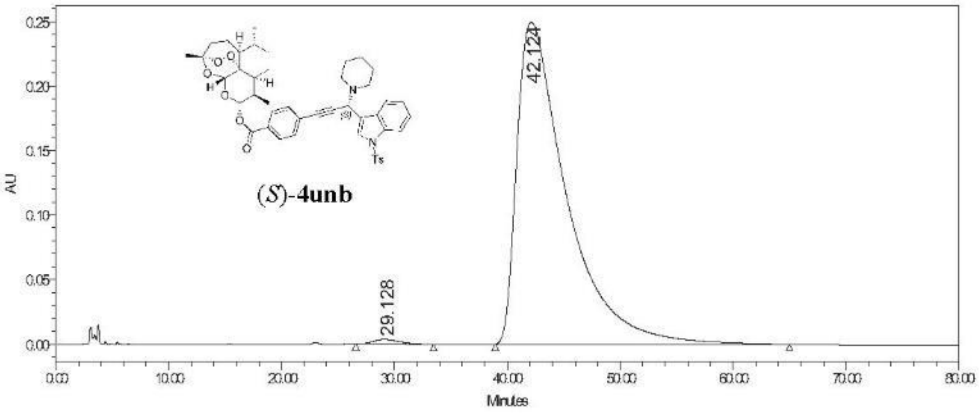

Processed Channel Descr.: W2489 ChA 214nm

	Processed Channel Descr.	RT	Area	% Area	Height
1	W2489 ChA 214nm	29.128	547797	0.71	3527
2	W2489 ChA 214nm	42.124	76083351	99.29	249568

Reported by User: System
Report Method: Injection Summary Report
Report Method ID: 1639 1639
Page: 1 of 1

Project Name: HPLC
Date Printed:
12/7/2019
1:00:32 PM PRC

HPLC spectrum for (*S*)-4unb

Empower™ 3 SOFTWARE

Injection Summary Report

SAMPLE INFORMATION

Sample Name:	lq-7-061-066-od-h-90-10-1-214	Acquired By:	System
Sample Type:	Unknown	Sample Set Name:	
Vial:	1	Acq. Method Set:	HPLC
Injection #:	1	Processing Method:	Default
Injection Volume:	5.00 ul	Channel Name:	W2489 ChA
Run Time:	90.0 Minutes	Proc. Chnl. Descr.:	W2489 ChA 214nm
Date Acquired:	12/6/2019 11:15:53 PM CST		
Date Processed:	12/7/2019 12:57:41 PM CST		

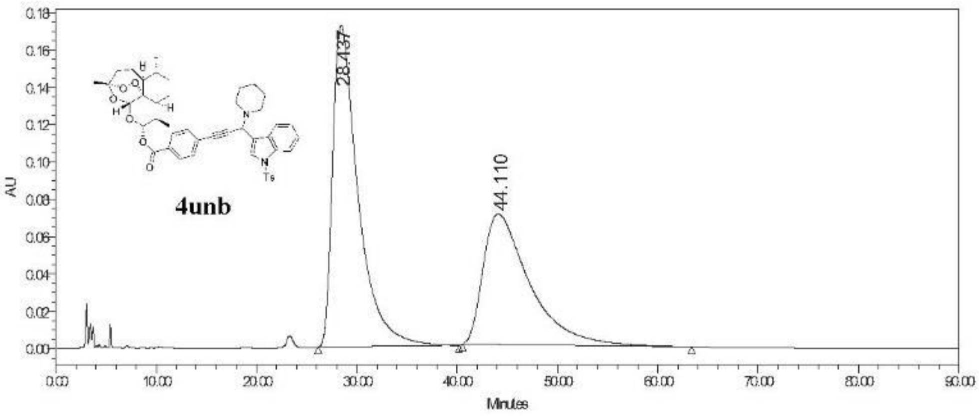

Channel: W2489 ChA; Processed Channel: W2489 ChA 214nm; Result Id: 6303; Processing Method: Defaul

Processed Channel Descr.: W2489 ChA 214nm

	Processed Channel Descr.	RT	Area	% Area	Height
1	W2489 ChA 214nm	28.437	30324204	57.33	172080
2	W2489 ChA 214nm	44.110	22574040	42.67	69958

HPLC spectrum for (±)-4unb

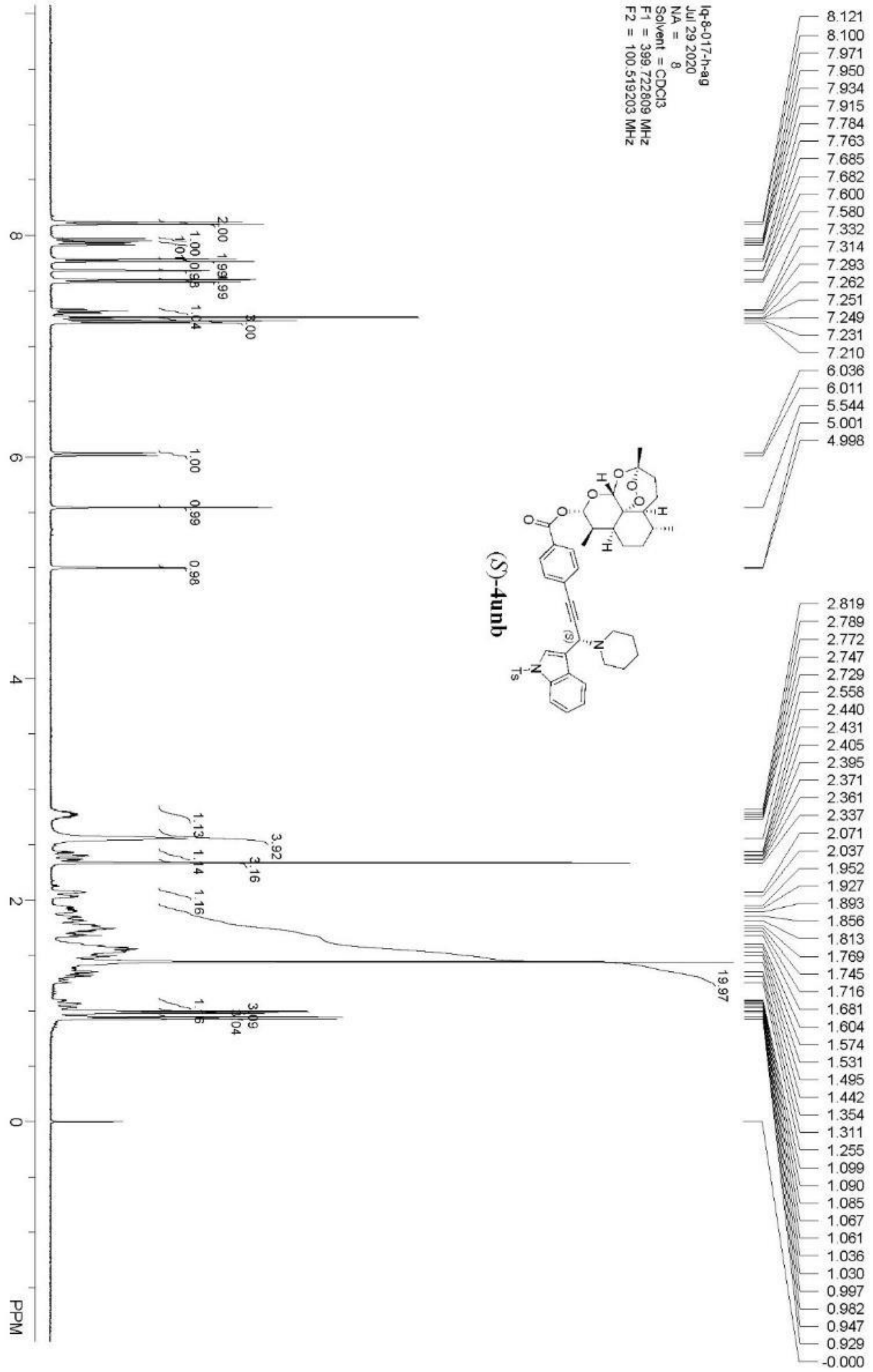

^{1}H NMR (400 MHz, CDCl3) spectrum for (*S*)-4unb

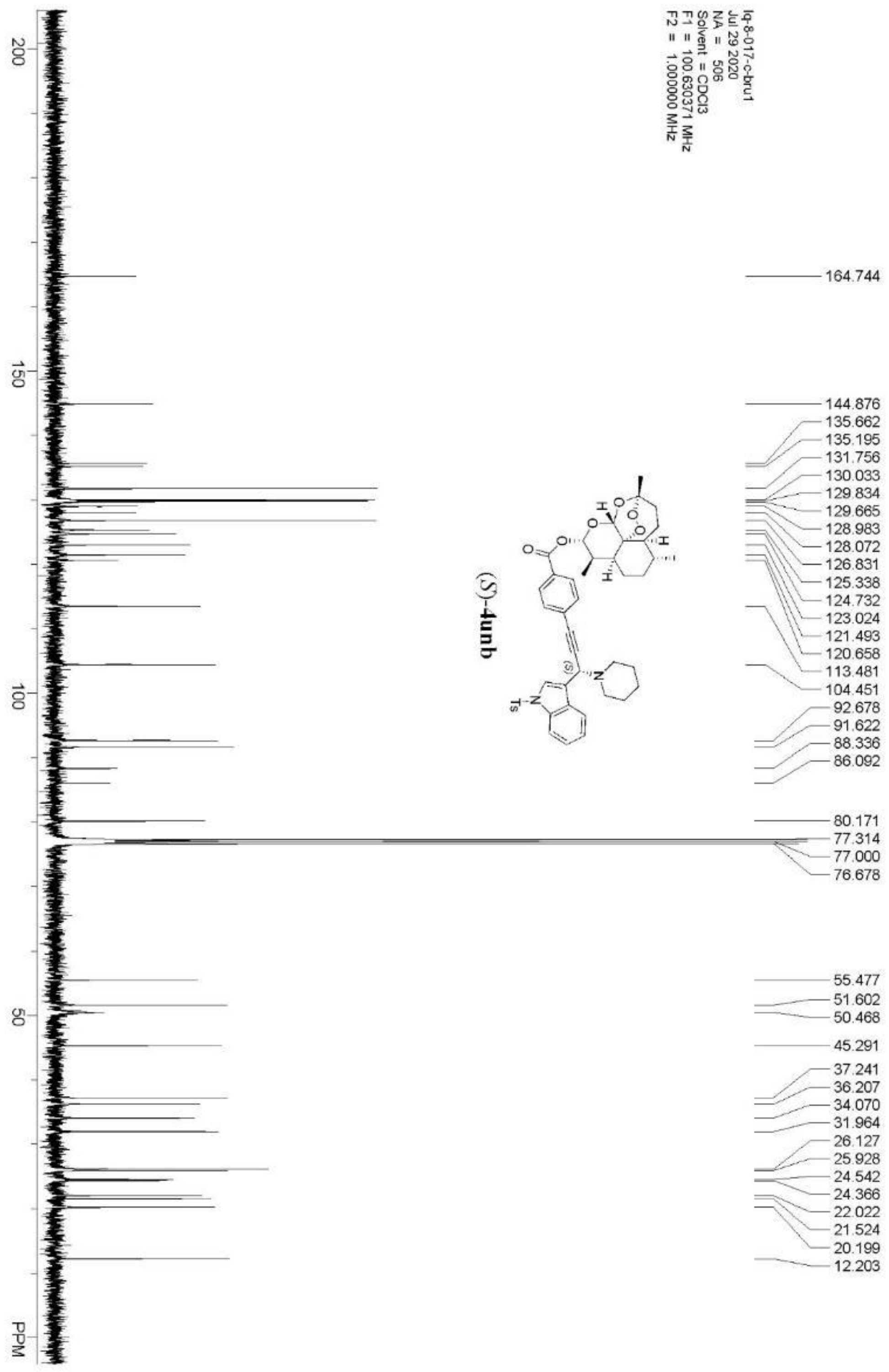

^{13}C NMR (400 MHz, CDCl3) spectrum for (*S*)-4unb

Empower™ 3 SOFTWARE

Injection Summary Report

SAMPLE INFORMATION

Sample Name:	lq-8-017-od-h-90-10-1-214	Acquired By:	System
Sample Type:	Unknown	Sample Set Name:	
Vial:	1	Acq. Method Set:	HPLC
Injection #:	2	Processing Method:	Default
Injection Volume:	10.00 ul	Channel Name:	W2489 ChA
Run Time:	90.0 Minutes	Proc. Chnl. Descr.:	W2489 ChA 214nm
Date Acquired:	7/29/2020 8:21:48 AM CST		
Date Processed:	7/29/2020 9:47:13 AM CST		

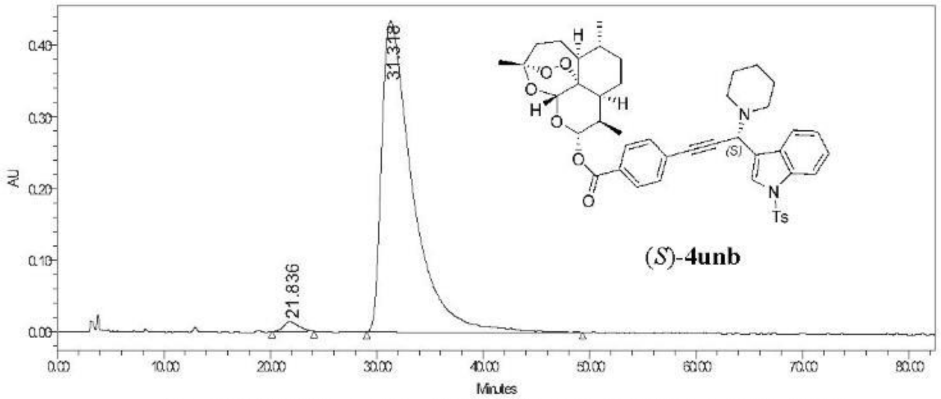

Channel: W2489 ChA; Processed Channel: W2489 ChA 214nm; Result Id: 7440; Processing Method: Default

Processed Channel Descr.: W2489 ChA 214nm

	Processed Channel Descr.	RT	Area	%Area	Height
1	W2489 ChA 214nm	21.836	1303906	1.46	12997
2	W2489 ChA 214nm	31.318	87878212	98.54	433718

Reported by User: System
Report Method: Injection Summary Report
Report Method ID: 6865 6865
Page: 1 of 1

Project Name: HPLC
Date Printed:
7/30/2020
2:08:36 AM PRC

HPLC spectrum for (*S*)-4unb

Empower 3 SOFTWARE

Injection Summary Report

SAMPLE INFORMATION

Sample Name:	lq-8-013-mixed-od-h-90-10-1-214	Acquired By:	System
Sample Type:	Unknown	Sample Set Name:	
Vial:	1	Acq. Method Set:	HPLC
Injection #:	1	Processing Method:	Default
Injection Volume:	10.00 ul	Channel Name:	W2489 ChA
Run Time:	90.0 Minutes	Proc. Chnl. Descr.:	W2489 ChA 214nm
Date Acquired:	7/29/2020 4:27:22 AM CST		
Date Processed:	7/29/2020 9:48:18 AM CST		

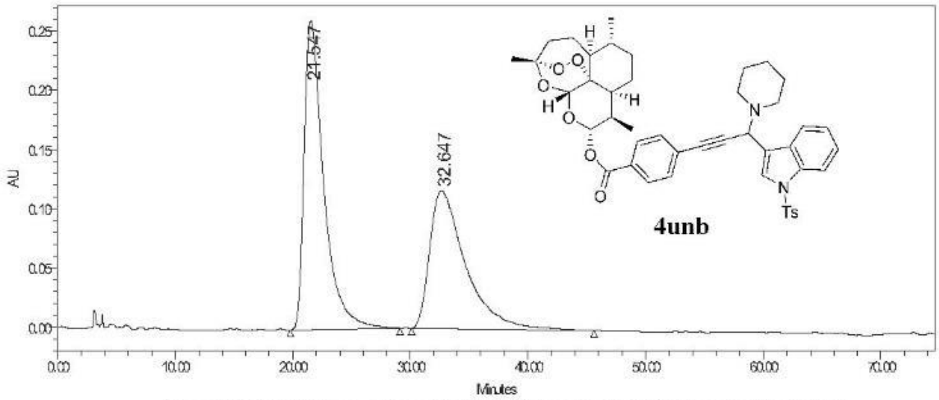

Channel: W2489 ChA; Processed Channel: W2489 ChA 214nm; Result Id: 7441; Processing Method: Defaul

Processed Channel Descr.: W2489 ChA 214nm

	Processed Channel Descr.	RT	Area	%Area	Height
1	W2489 ChA 214nm	21.547	30142474	54.94	260508
2	W2489 ChA 214nm	32.647	24723288	45.06	116717

Reported by User: System
Report Method: Injection Summary Report
Report Method ID: 6865 6865
Page: 1 of 1

Project Name: HPLC
Date Printed:
7/30/2020
2:09:22 AM PRC

HPLC spectrum for (±)-4unb

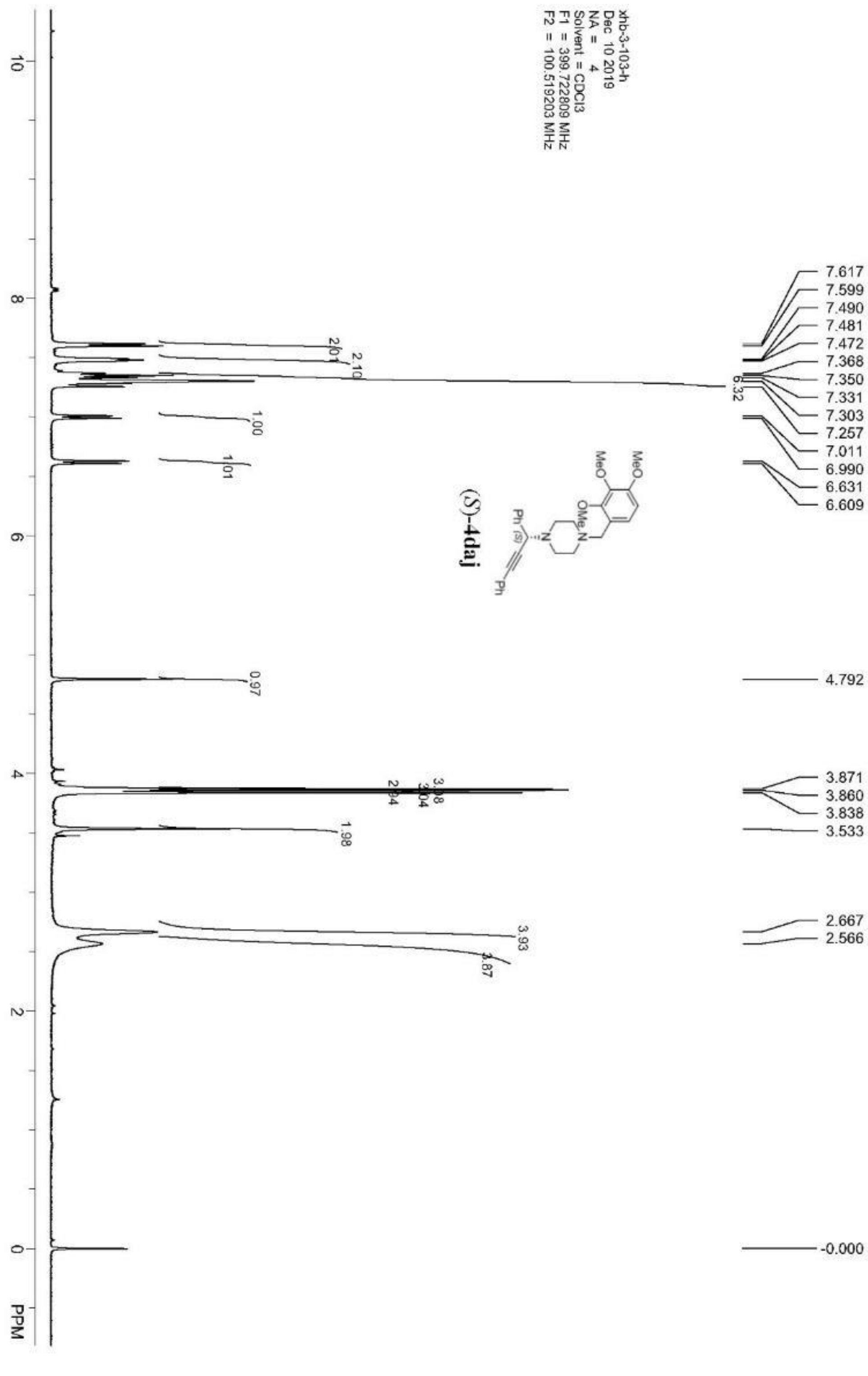

¹H NMR (400 MHz, CDCl3) spectrum for (*S*)**-4daj**

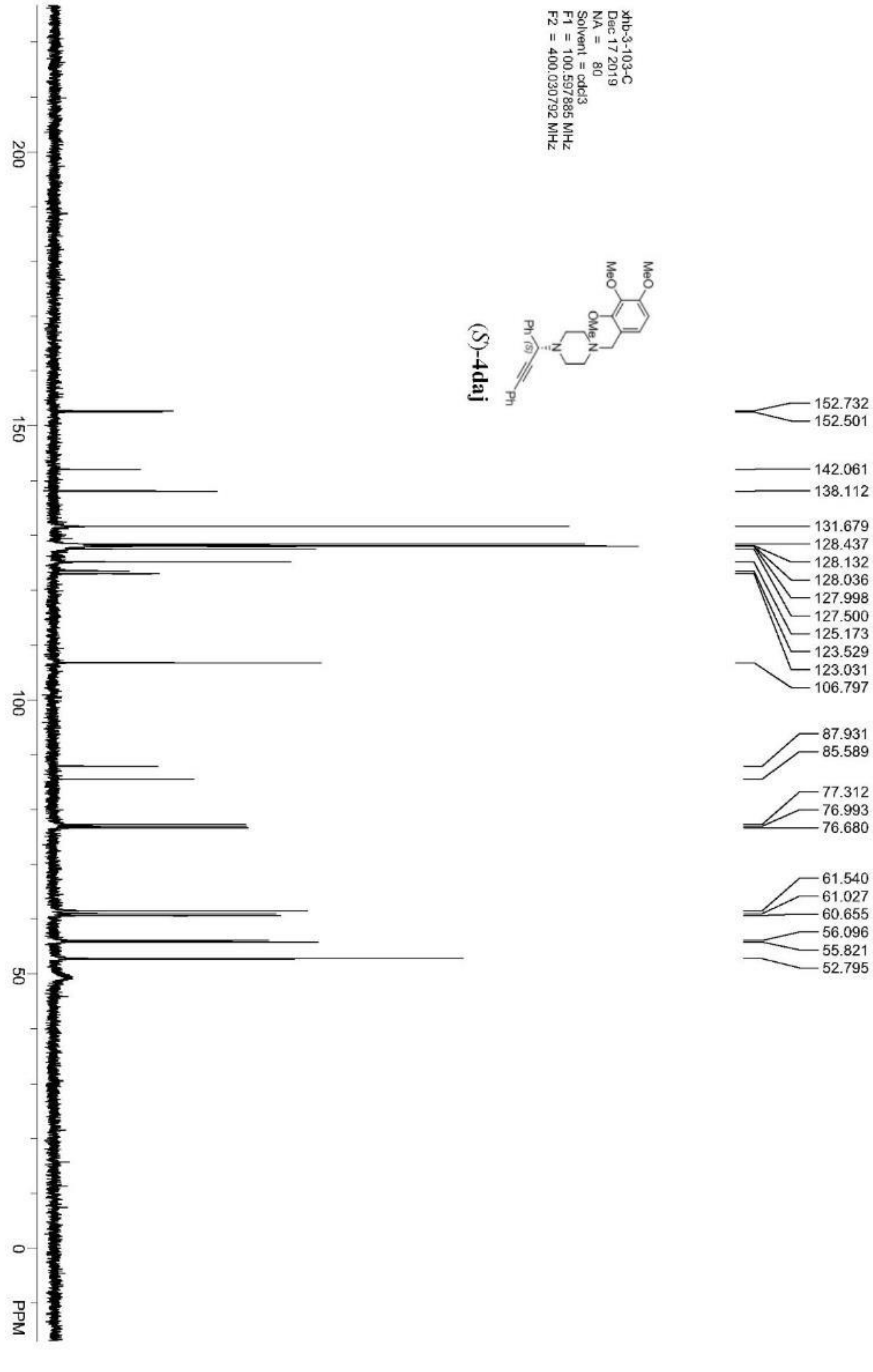

^{13}C NMR (400 MHz, CDCl$_3$) spectrum for (*S*)-4daj

Empower 3 SOFTWARE

Injection Summary Report

SAMPLE INFORMATION

Sample Name:	xhb-3-103-cj-h-80-20-0.7-214	Acquired By:	System
Sample Type:	Unknown	Sample Set Name:	
Vial:	1	Acq. Method Set:	HPLC
Injection #:	1	Processing Method:	LC PQ
Injection Volume:	5.00 ul	Channel Name:	W2489 ChA
Run Time:	40.0 Minutes	Proc. Chnl. Descr.:	W2489 ChA 214nm
Date Acquired:	12/10/2019 1:49:21 AM CST		
Date Processed:	12/10/2019 2:36:37 AM CST		

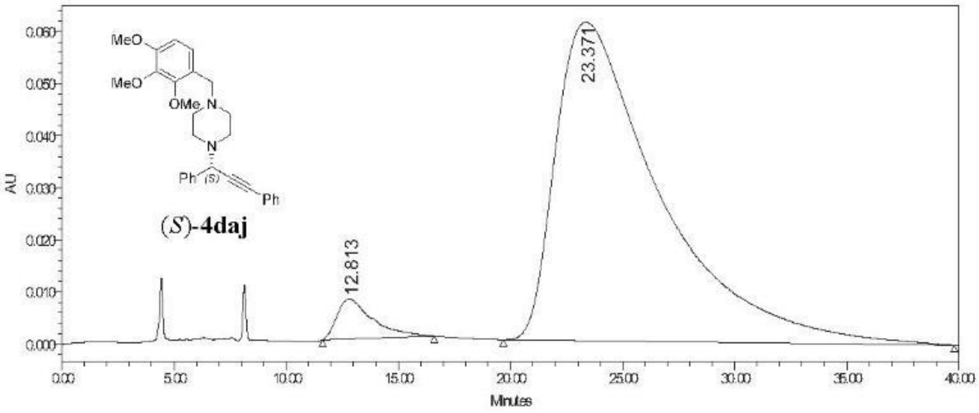

Processed Channel Descr.: W2489 ChA 214nm

	Processed Channel Descr.	RT	Area	% Area	Height
1	W2489 ChA 214nm	12.813	843404	4.09	7658
2	W2489 ChA 214nm	23.371	19756053	95.91	61177

Reported by User: System
Report Method: Injection Summary Report
Report Method ID: 1639 1639
Page: 1 of 1

Project Name: HPLC
Date Printed:
12/19/2019
1:00:46 AM PRC

HPLC spectrum for (*S*)-4daj

Empower 3 SOFTWARE

Injection Summary Report

SAMPLE INFORMATION

Sample Name:	xhb-3-096-cj-h-80-20-0.7-214	Acquired By:	System
Sample Type:	Unknown	Sample Set Name:	
Vial:	1	Acq. Method Set:	HPLC
Injection #:	3	Processing Method:	LC PQ
Injection Volume:	2.50 ul	Channel Name:	W2489 ChA
Run Time:	50.0 Minutes	Proc. Chnl. Descr.:	W2489 ChA 214nm
Date Acquired:	12/10/2019 5:32:55 AM CST		
Date Processed:	12/10/2019 6:22:02 AM CST		

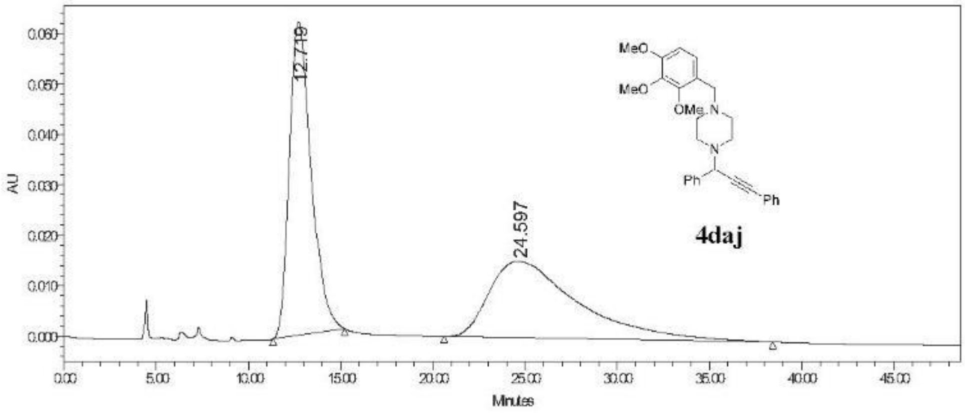

Processed Channel Descr.: W2489 ChA 214nm

	Processed Channel Descr.	RT	Area	%Area	Height
1	W2489 ChA 214nm	12.719	5092455	50.18	62021
2	W2489 ChA 214nm	24.597	5055202	49.82	15105

Reported by User: System
Report Method: Injection Summary Report
Report Method ID: 1639 1639
Page: 1 of 1

Project Name: HPLC
Date Printed:
12/19/2019
1:00:16 AM PRC

HPLC spectrum for (±)-4daj

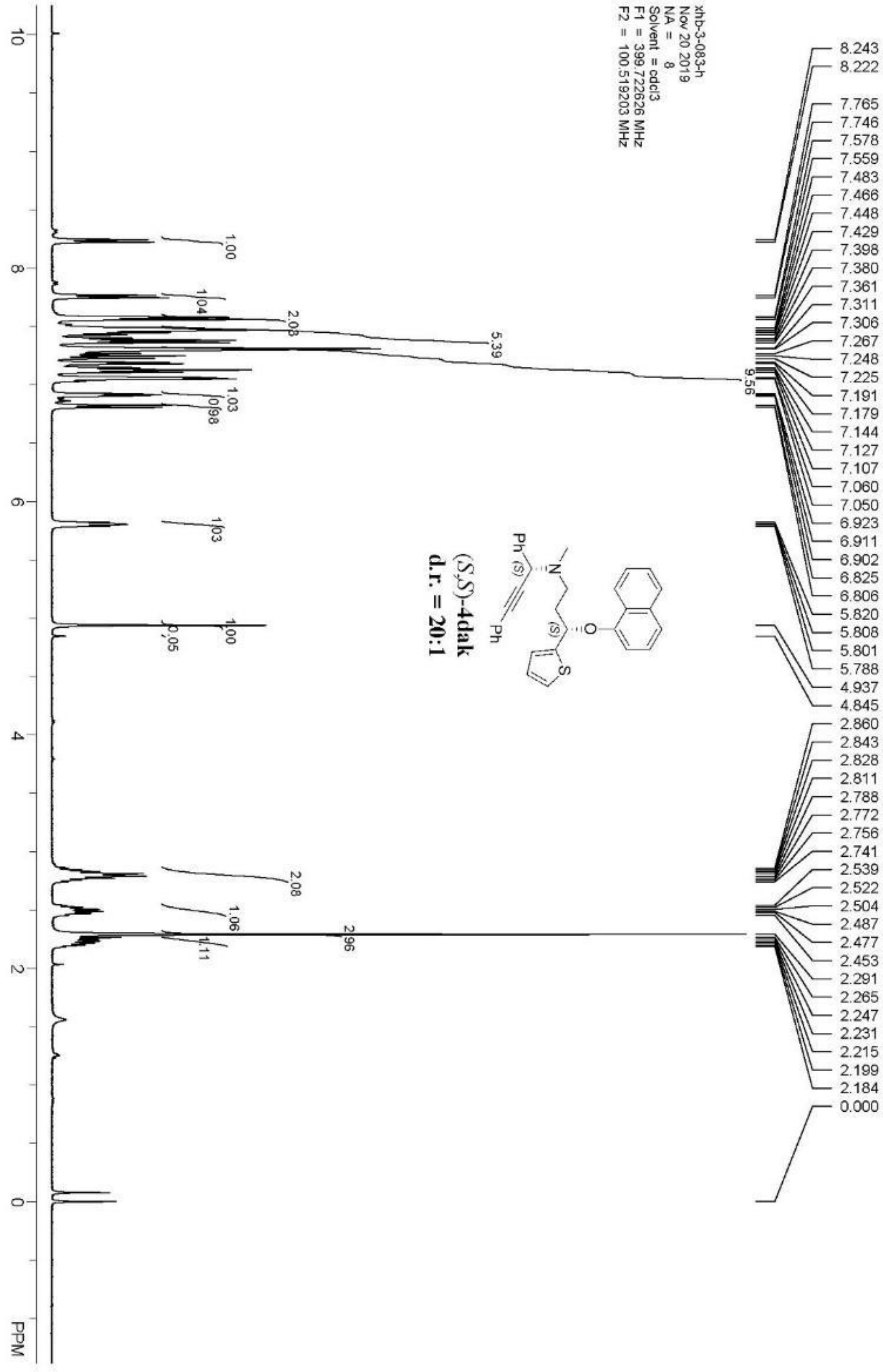

^{1}H NMR (400 MHz, CDCl3) spectrum for (*S*,*S*)-4dak

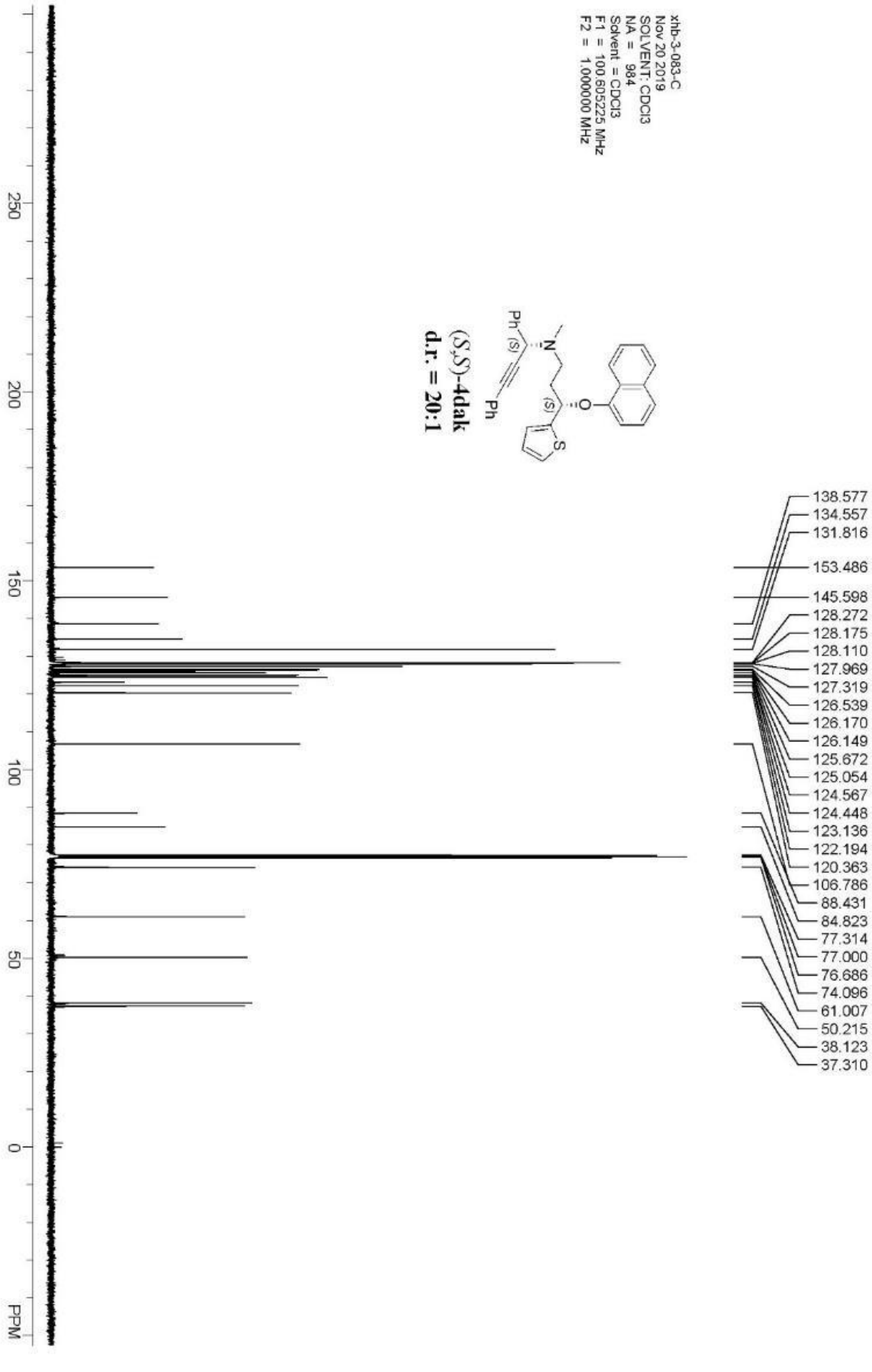

^{13}C NMR (400 MHz, CDCl3) spectrum for (*S*,*S*)-4dak

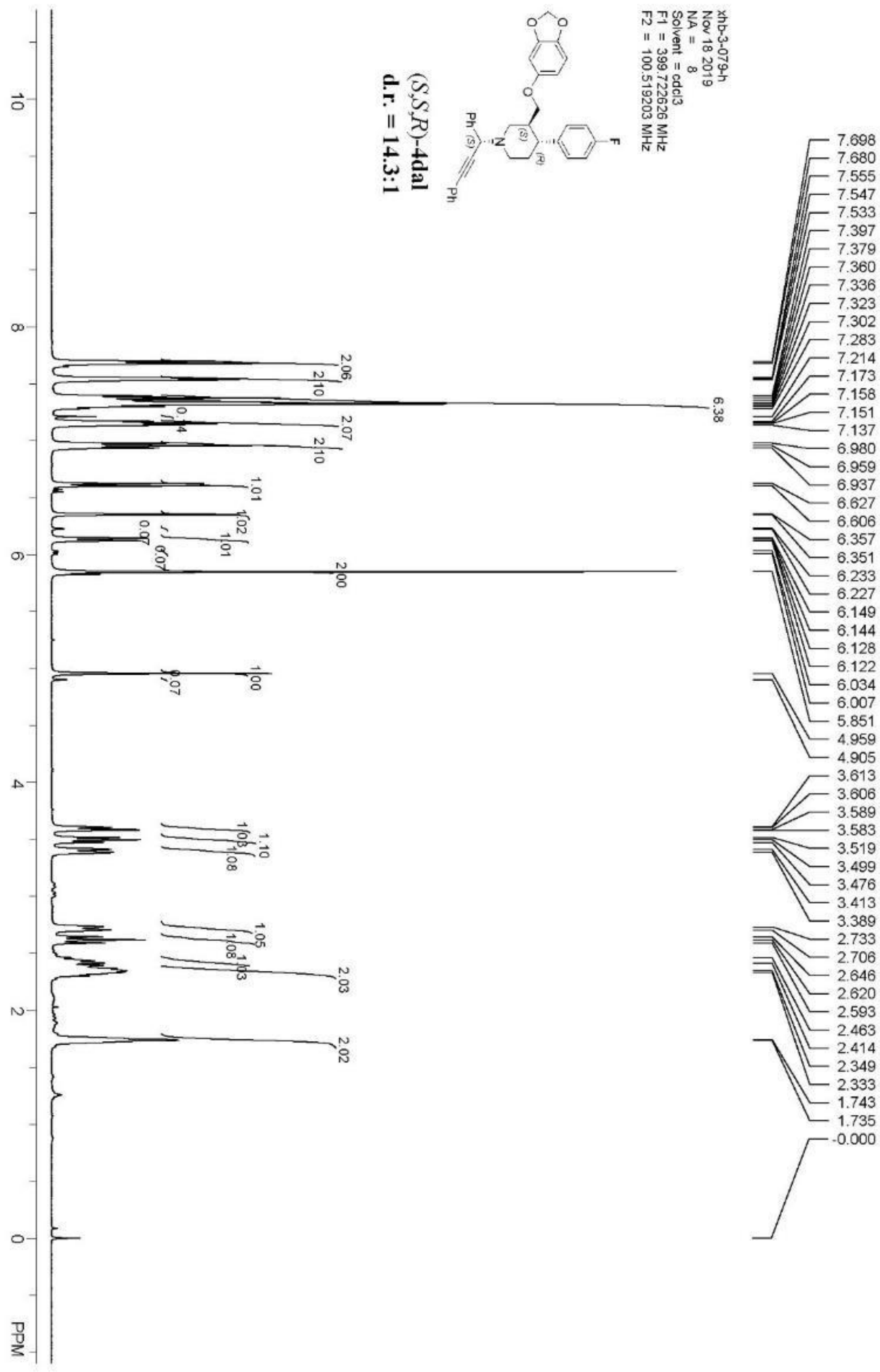

^{1}H NMR (400 MHz, CDCl3) spectrum for (*S*,*S*)**-4dal**

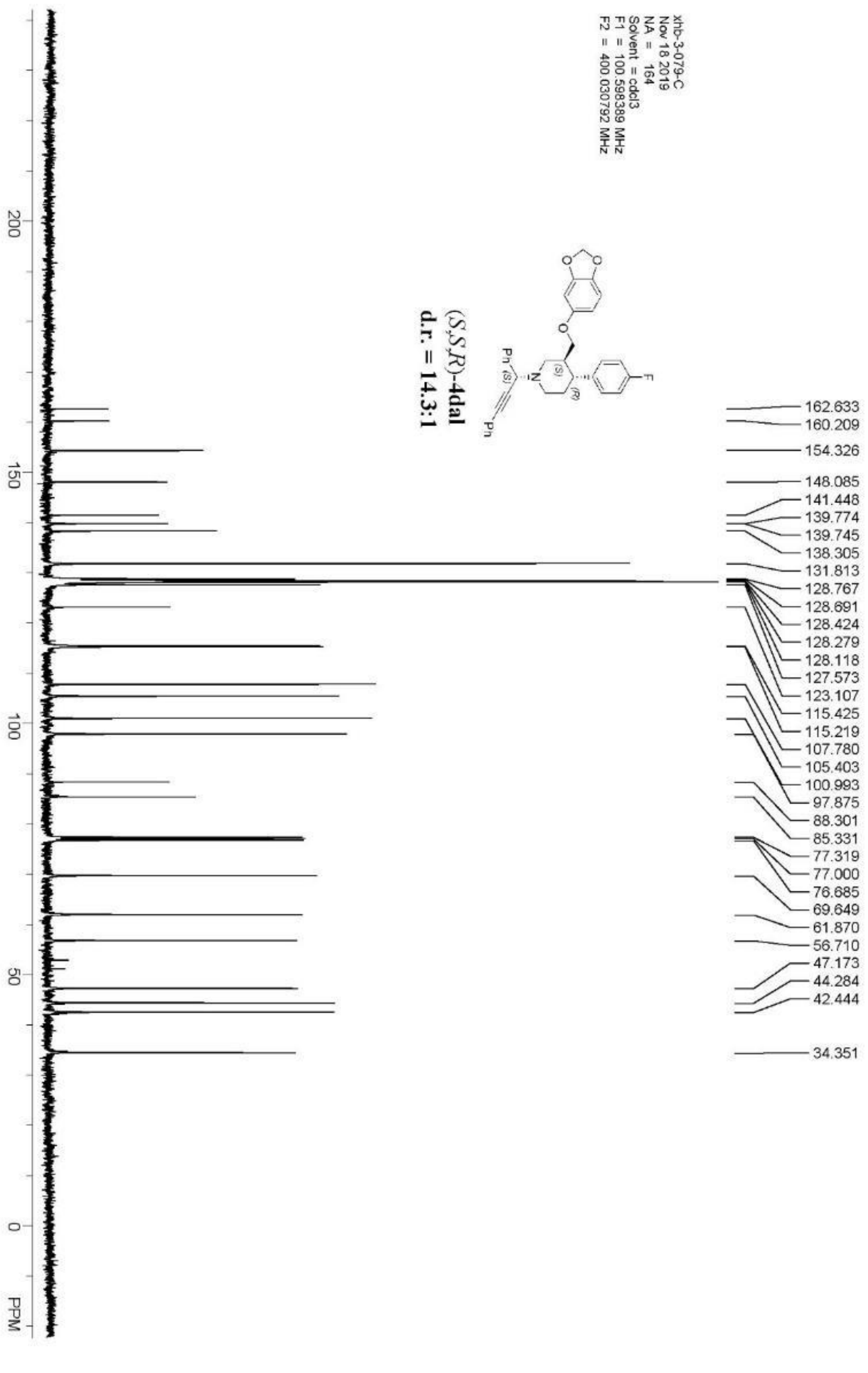

^{13}C NMR (400 MHz, CDCl$_3$) spectrum for (*S*,*S*)-4dal

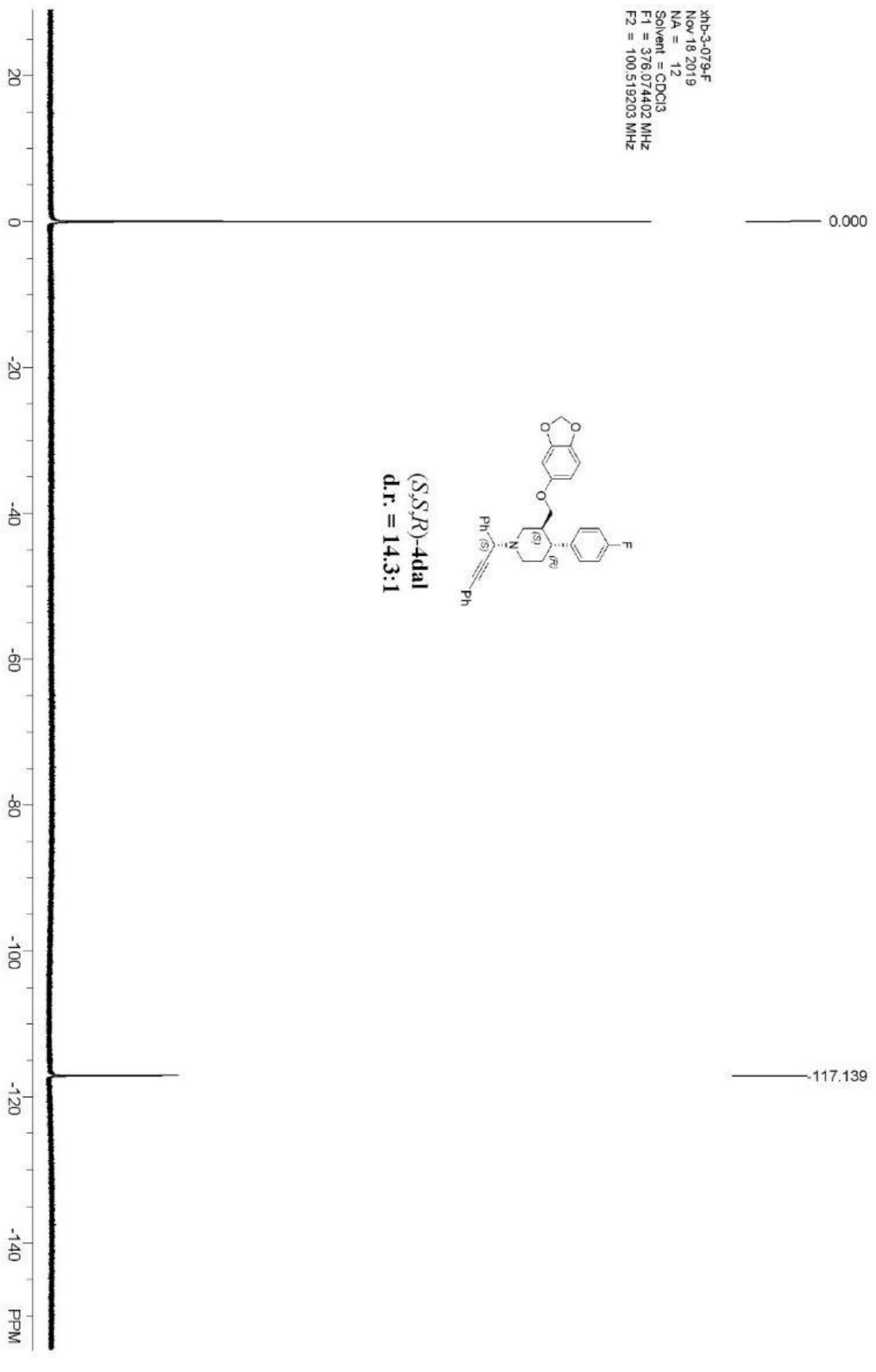

^{19}F NMR (376 MHz, $CDCl_3$) spectrum for (*S*,*S*)-4dal ($CFCl_3$ was used as the internal standard)

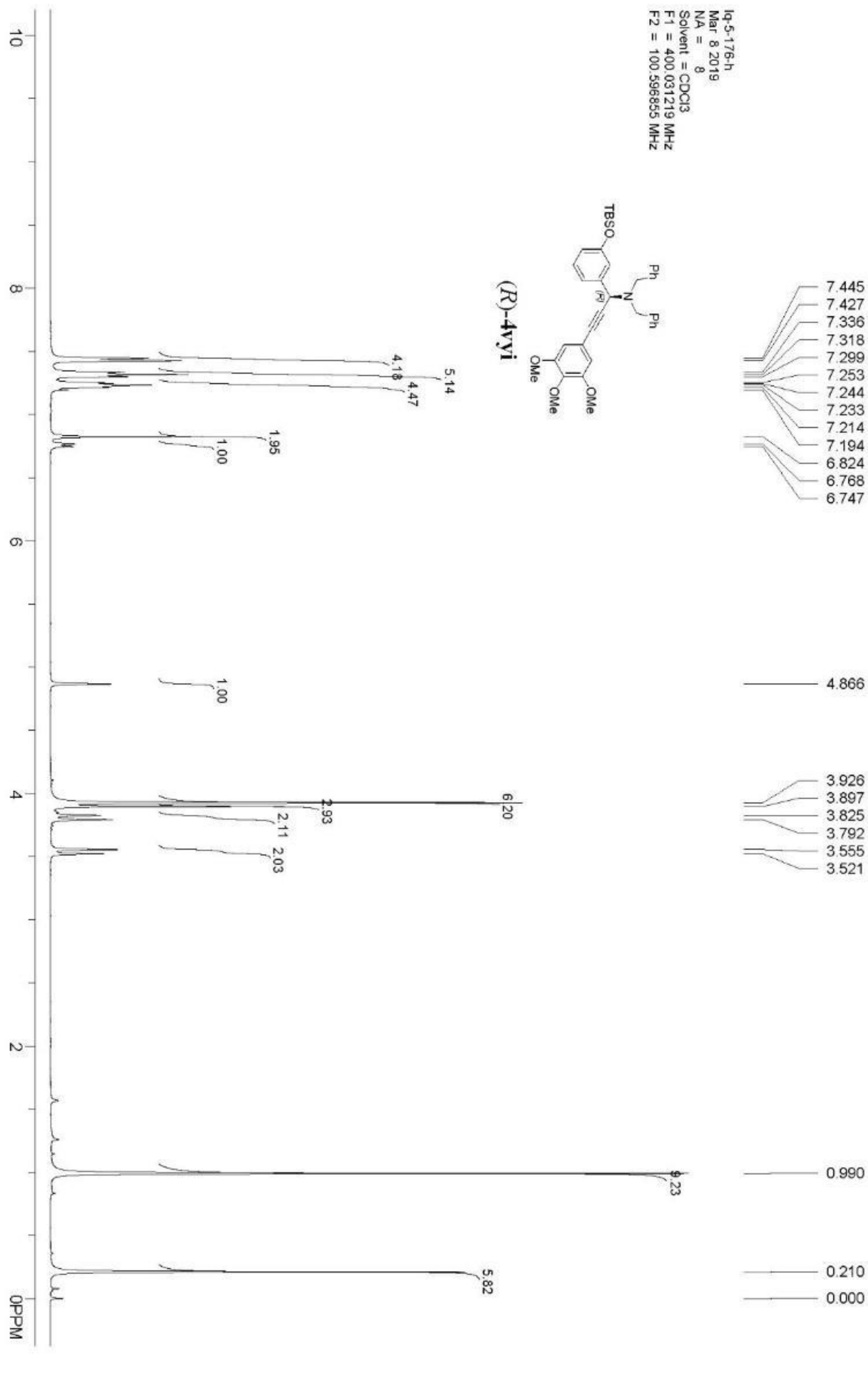

^{1}H NMR (400 MHz, CDCl3) spectrum for (*R*)**-4vyi**

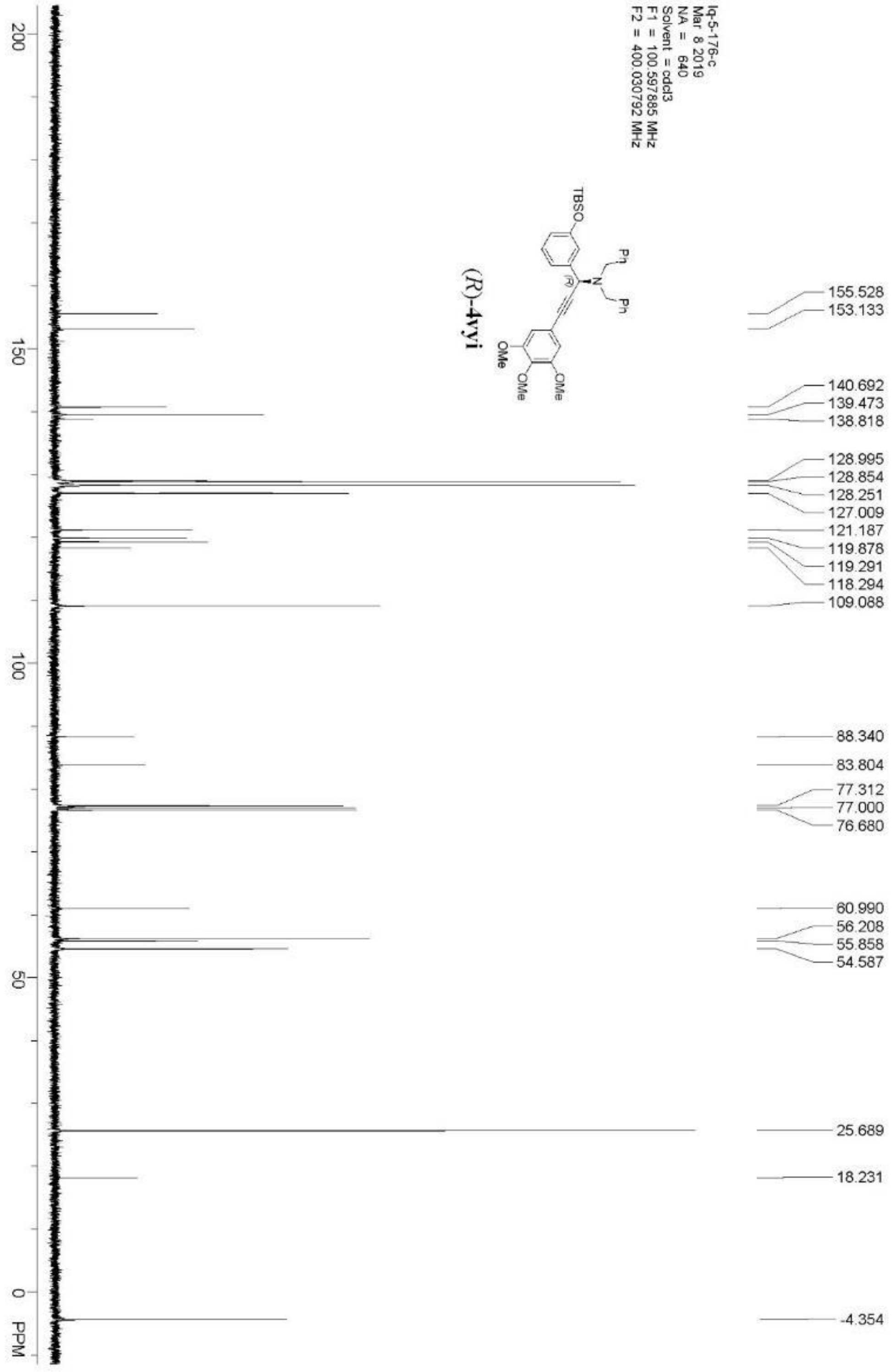

^{13}C NMR (400 MHz, CDCl3) spectrum for (*R*)**-4vyi**

Empower 3

Injection Summary Report

SAMPLE INFORMATION

Sample Name:	lq-5-176-ad-h-99-1-0.5-214	Acquired By:	System
Sample Type:	Unknown	Sample Set Name:	
Vial:	1	Acq. Method Set:	HPLC
Injection #:	2	Processing Method:	Default
Injection Volume:	3.00 ul	Channel Name:	W2489 ChA
Run Time:	120.0 Minutes	Proc. Chnl. Descr.:	W2489 ChA 214nm
Date Acquired:	3/7/2019 1:40:01 AM CST		
Date Processed:	3/7/2019 3:41:31 AM CST		

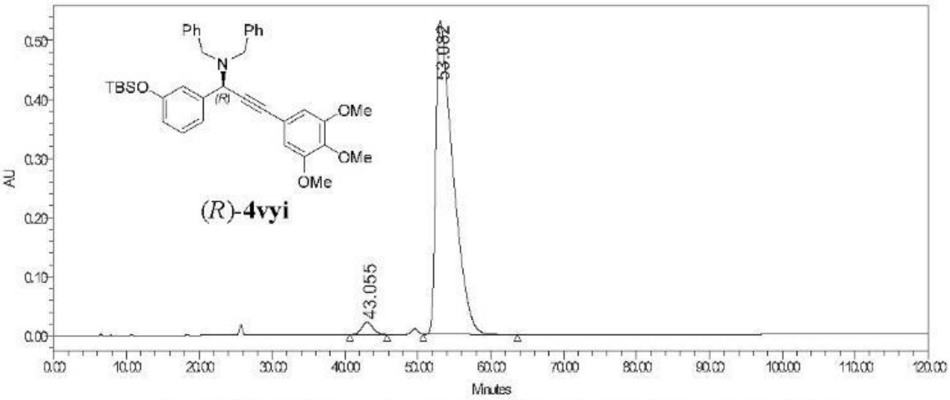

Channel: W2489 ChA; Processed Channel: W2489 ChA 214nm; Result Id: 2726; Processing Method: Default

Processed Channel Descr.: W2489 ChA 214nm

	Processed Channel Descr.	RT	Area	%Area	Height
1	W2489 ChA 214nm	43.055	2205967	2.49	20309
2	W2489 ChA 214nm	53.082	86511621	97.51	529946

Reported by User: System
Report Method: Injection Summary Report
Report Method ID: 1639 1639
Page: 1 of 1

Project Name: HPLC
Date Printed:
3/7/2019
5:29:28 AM PRC

HPLC spectrum for (*R*)-4vyi

Empower 3 SOFTWARE

Injection Summary Report

SAMPLE INFORMATION

Sample Name:	lq-5-157-ad-h-99-1-0.5-214	Acquired By:	System
Sample Type:	Unknown	Sample Set Name:	
Vial:	1	Acq. Method Set:	HPLC
Injection #:	3	Processing Method:	Default
Injection Volume:	5.00 ul	Channel Name:	W2489 ChA
Run Time:	120.0 Minutes	Proc. Chnl. Descr.:	W2489 ChA 214nm
Date Acquired:	3/7/2019 3:43:16 AM CST		
Date Processed:	3/7/2019 5:29:14 AM CST		

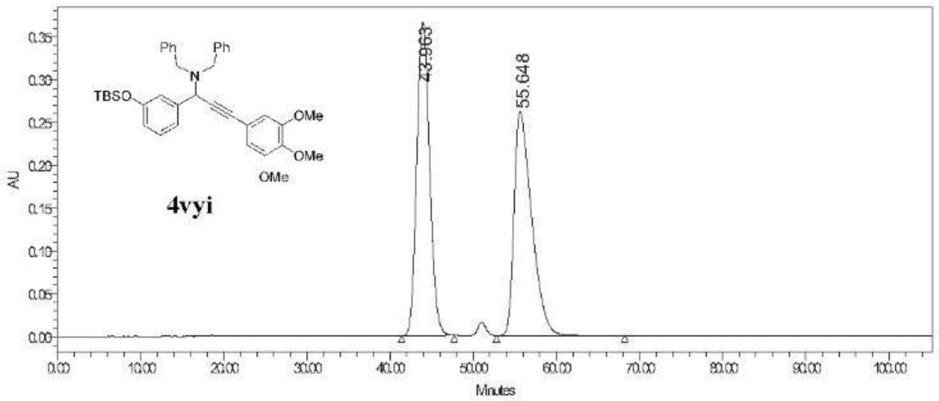

Processed Channel Descr.: W2489 ChA 214nm

	Processed Channel Descr.	RT	Area	%Area	Height
1	W2489 ChA 214nm	43.963	39216994	50.51	365302
2	W2489 ChA 214nm	55.648	38423500	49.49	261150

Reported by User: System
Report Method: Injection Summary Report
Report Method ID: 1639 1639
Page: 1 of 1

Project Name: HPLC
Date Printed:
3/7/2019
5:29:57 AM PRC

HPLC spectrum for (±)-4vyi

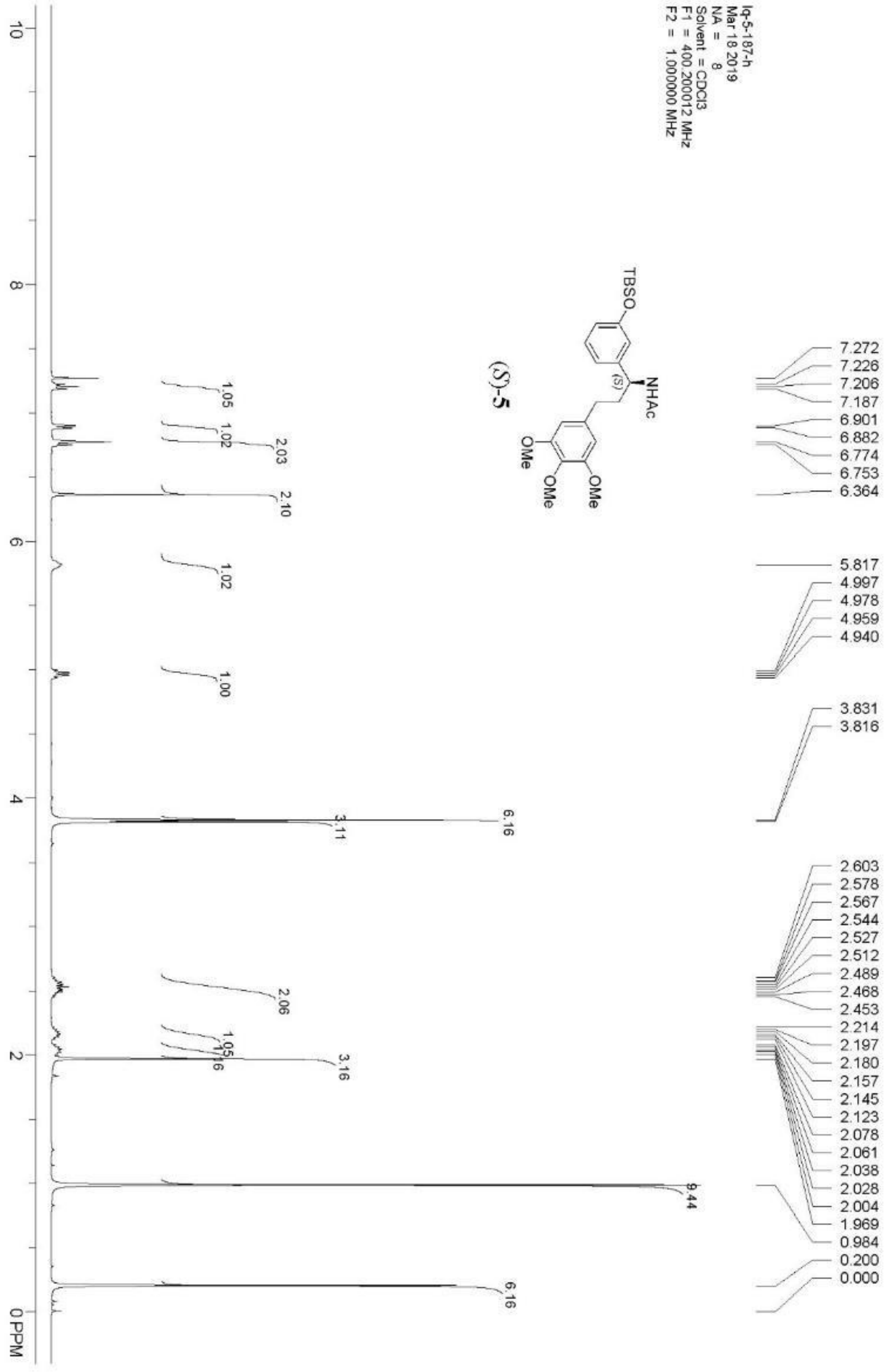

^{1}H NMR (400 MHz, CDCl3) spectrum for (*R*)**-4vyi**

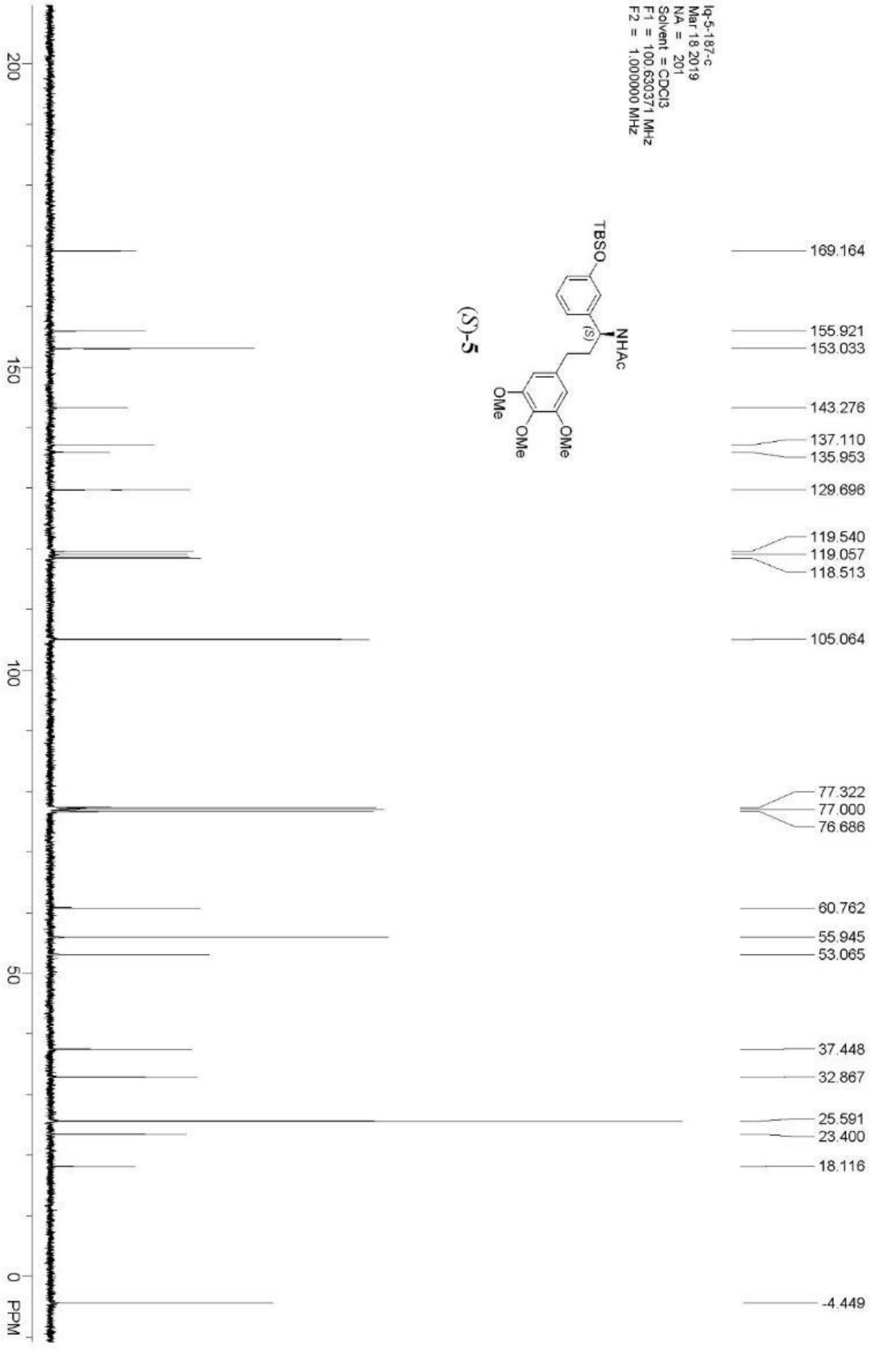

^{13}C NMR (400 MHz, $CDCl_3$) spectrum for (*S*)-5

Empower 3 SOFTWARE

Injection Summary Report

SAMPLE INFORMATION

Sample Name:	lq-5-187-od-h-80-20-0.5-214	Acquired By:	System
Sample Type:	Unknown	Sample Set Name:	
Vial:	1	Acq. Method Set:	HPLC
Injection #:	2	Processing Method:	Default
Injection Volume:	5.00 ul	Channel Name:	W2489 ChA
Run Time:	30.0 Minutes	Proc. Chnl. Descr.:	W2489 ChA 214nm
Date Acquired:	3/19/2019 1:18:37 AM CST		
Date Processed:	3/19/2019 1:47:43 AM CST		

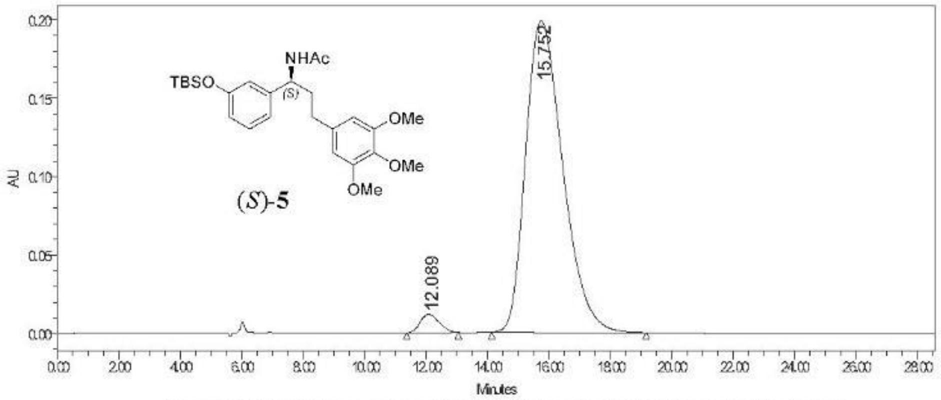

Processed Channel Descr.: W2489 ChA 214nm

	Processed Channel Descr.	RT	Area	%Area	Height
1	W2489 ChA 214nm	12.089	498460	2.90	11713
2	W2489 ChA 214nm	15.752	16671113	97.10	198304

Reported by User: System
Report Method: Injection Summary Report
Report Method ID: 1639 1639
Page: 1 of 1

Project Name: HPLC
Date Printed:
3/19/2019
1:50:10 AM PRC

HPLC spectrum for (*S*)-5

Empower 3 SOFTWARE

Injection Summary Report

SAMPLE INFORMATION

Sample Name:	lq-5-165-od-h-80-20-0.5-214	Acquired By:	System
Sample Type:	Unknown	Sample Set Name:	
Vial:	1	Acq. Method Set:	HPLC
Injection #:	4	Processing Method:	Default
Injection Volume:	3.00 ul	Channel Name:	W2489 ChA
Run Time:	30.0 Minutes	Proc. Chnl. Descr.:	W2489 ChA 214nm
Date Acquired:	3/19/2019 2:30:33 AM CST		
Date Processed:	3/19/2019 3:17:07 AM CST		

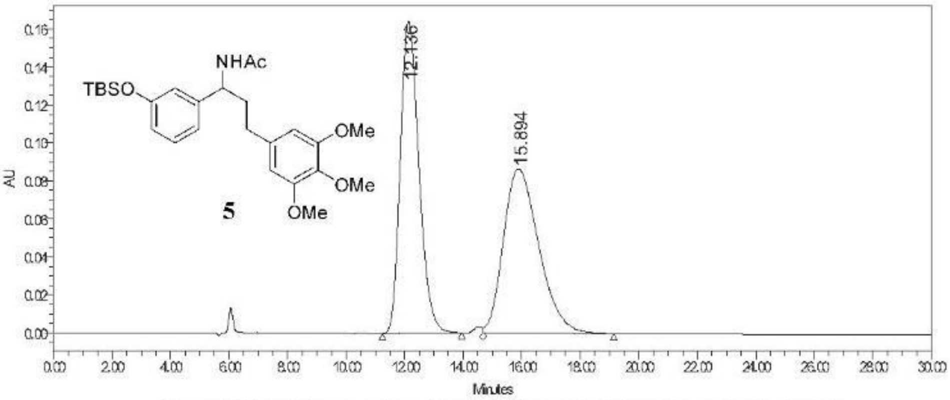

Channel: W2489 ChA; Processed Channel: W2489 ChA 214nm; Result Id: 2774; Processing Method: Defaul

Processed Channel Descr.: W2489 ChA 214nm

	Processed Channel Descr.	RT	Area	% Area	Height
1	W2489 ChA 214nm	12.136	7241809	49.99	164257
2	W2489 ChA 214nm	15.894	7243699	50.01	86408

Reported by User: System
Report Method: Injection Summary Report
Report Method ID: 1639 1639
Page: 1 of 1

Project Name: HPLC
Date Printed:
3/19/2019
3:17:44 AM PRC

HPLC spectrum for (±)-5

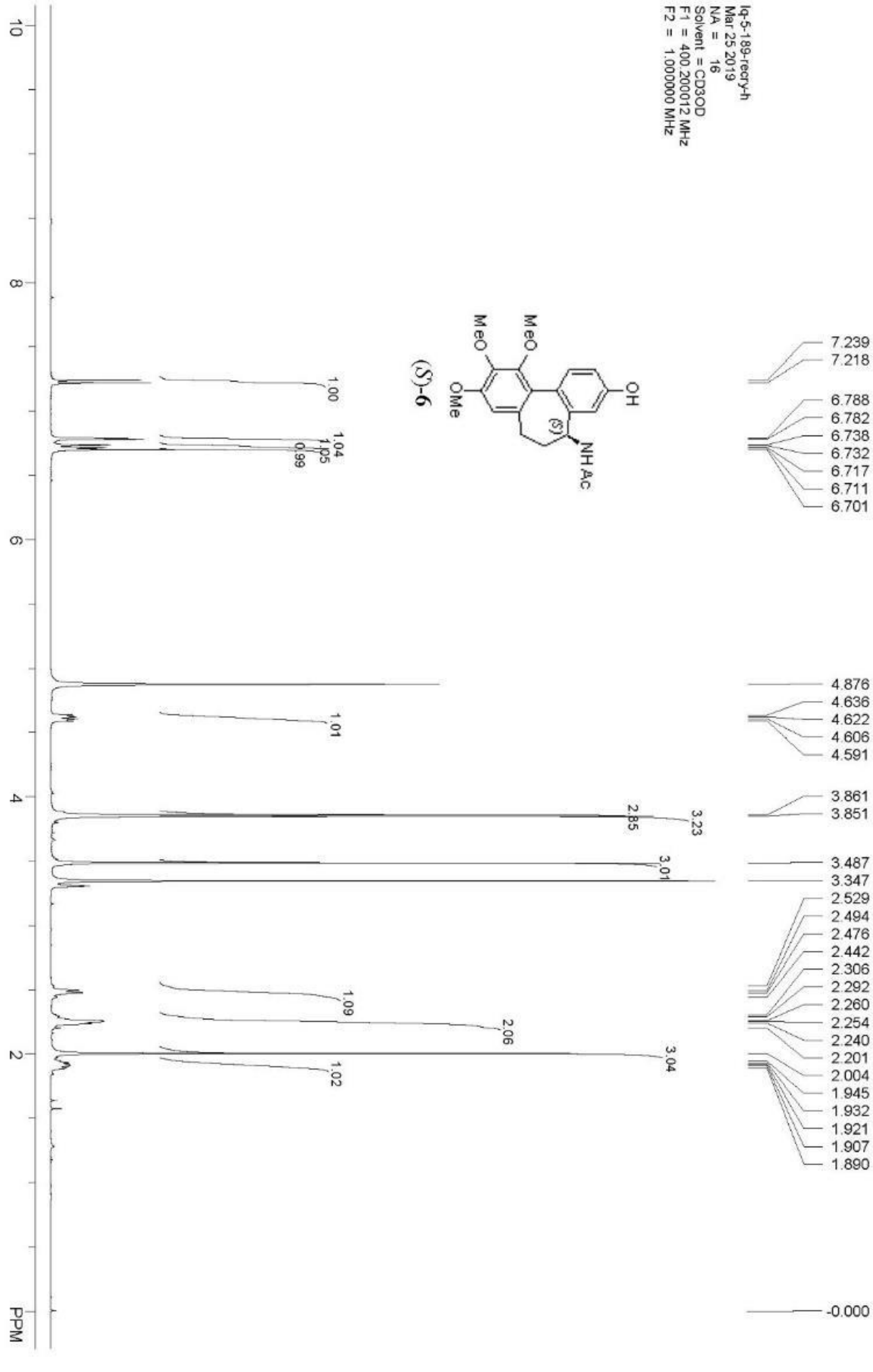

^{1}H NMR (400 MHz, CD_3OD) spectrum for (*S*)-5

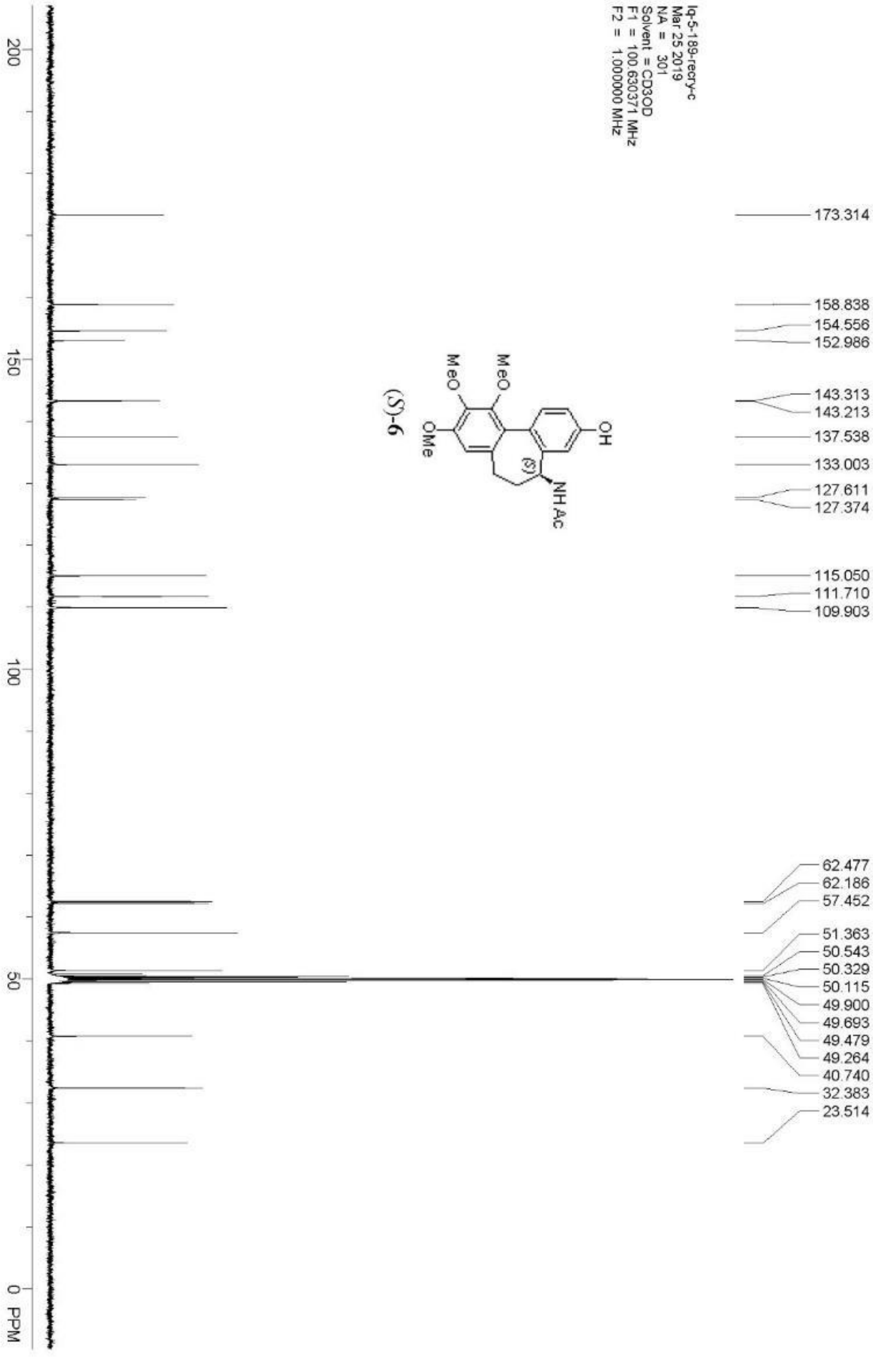

^{13}C NMR (400 MHz, CD_3OD) spectrum for (*S*)-6

Empower 3 SOFTWARE

Injection Summary Report

SAMPLE INFORMATION

Sample Name:	lq-5-189-re-od-h-80-20-1.0-214	Acquired By:	System
Sample Type:	Unknown	Sample Set Name:	
Vial:	1	Acq. Method Set:	HPLC
Injection #:	2	Processing Method:	xjz01
Injection Volume:	3.00 ul	Channel Name:	W2489 ChA
Run Time:	40.0 Minutes	Proc. Chnl. Descr.:	W2489 ChA 214nm
Date Acquired:	3/26/2019 2:23:28 PM CST		
Date Processed:	4/24/2019 1:57:39 PM CST		

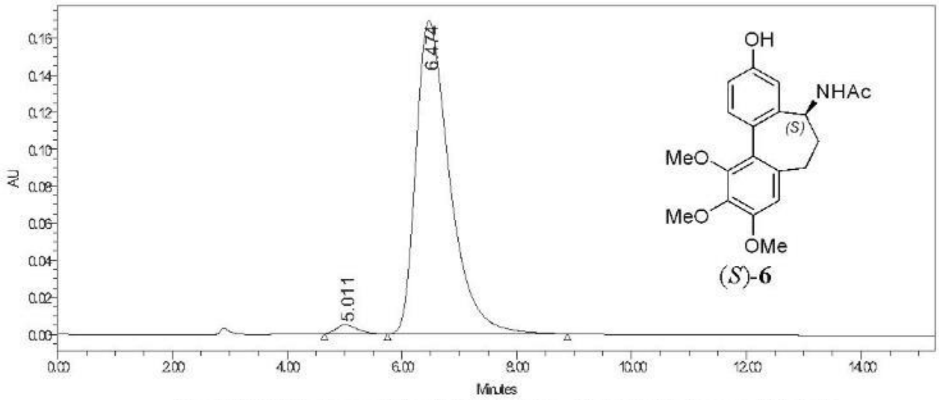

Channel: W2489 ChA; Processed Channel: W2489 ChA 214nm; Result Id: 2976; Processing Method: xjz01

Processed Channel Descr.: W2489 ChA 214nm

	Processed Channel Descr.	RT	Area	% Area	Height
1	W2489 ChA 214nm	5.011	120221	1.71	4679
2	W2489 ChA 214nm	6.474	6898485	98.29	169021

Reported by User: System
Report Method: Injection Summary Report
Report Method ID: 1639 1639
Page: 1 of 1

Project Name: HPLC
Date Printed:
4/24/2019
1:58:48 PM PRC

HPLC spectrum for (*S*)-6

Empower™ 3 SOFTWARE

Injection Summary Report

SAMPLE INFORMATION

Sample Name:	lq-5-184-od-h-80-20-1.0-214	Acquired By:	System
Sample Type:	Unknown	Sample Set Name:	
Vial:	1	Acq. Method Set:	HPLC
Injection #:	3	Processing Method:	Default
Injection Volume:	3.00 ul	Channel Name:	W2489 ChA
Run Time:	20.0 Minutes	Proc. Chnl. Descr.:	W2489 ChA 214nm
Date Acquired:	3/26/2019 2:45:40 PM CST		
Date Processed:	3/26/2019 3:03:50 PM CST		

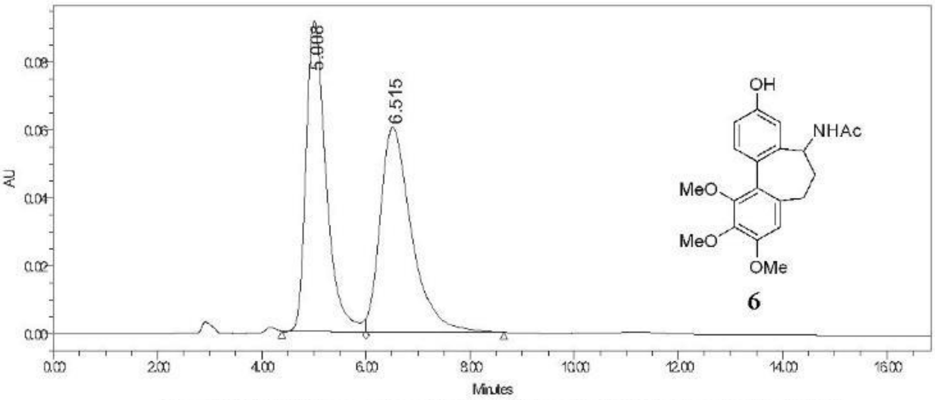

Channel: W2489 ChA; Processed Channel: W2489 ChA 214nm; Result Id: 2855; Processing Method: Defaul

Processed Channel Descr.: W2489 ChA 214nm

	Processed Channel Descr.	RT	Area	% Area	Height
1	W2489 ChA 214nm	5.008	2450831	49.47	91502
2	W2489 ChA 214nm	6.515	2503515	50.53	60252

Reported by User: System
Report Method: Injection Summary Report
Report Method ID: 1639 1639
Page: 1 of 1

Project Name: HPLC
Date Printed:
3/26/2019
3:04:08 PM PRC

HPLC spectrum for (±)-6

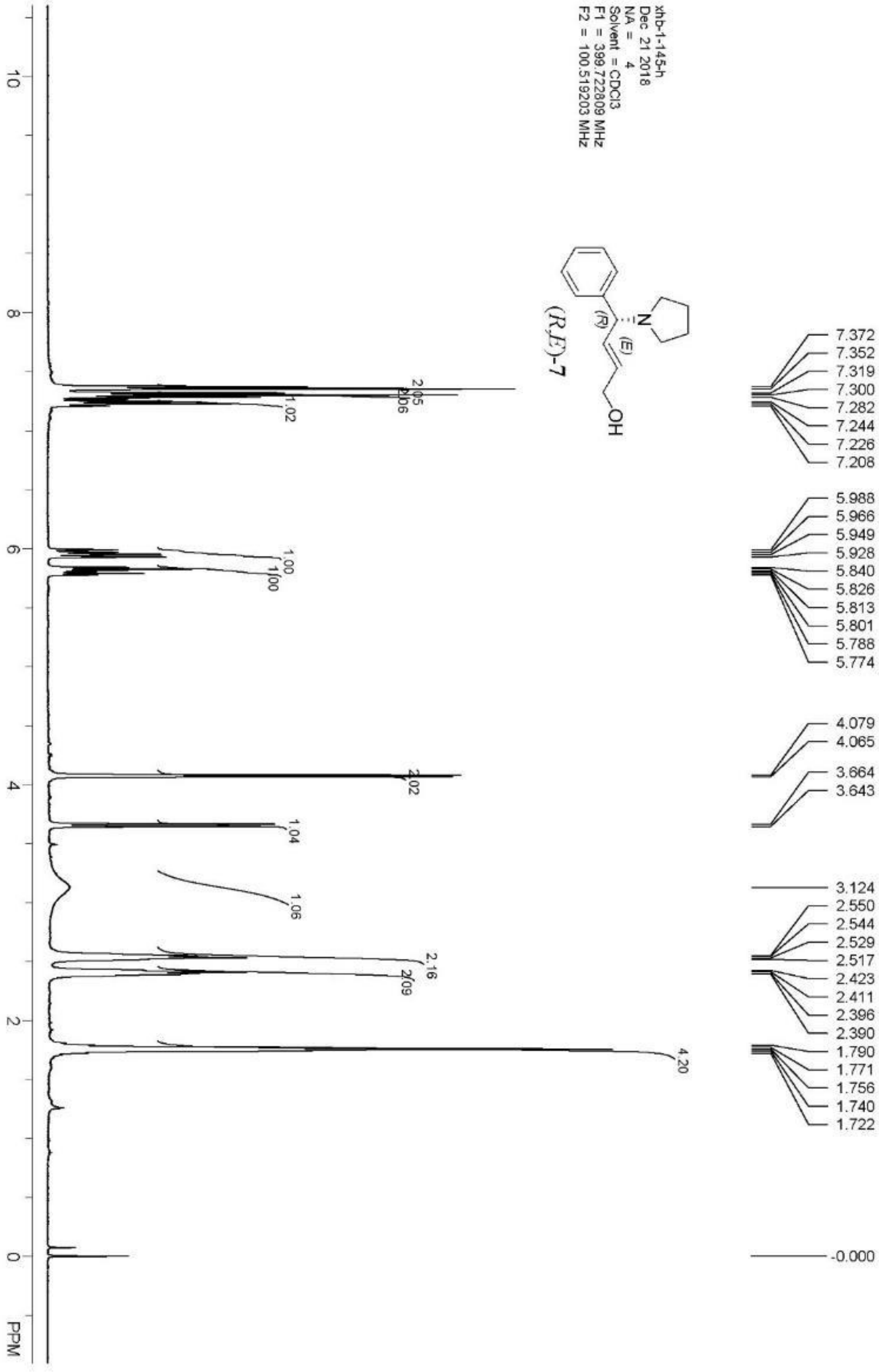

^{1}H NMR (400 MHz, CDCl3) spectrum for (*R*,*E*)-**7**

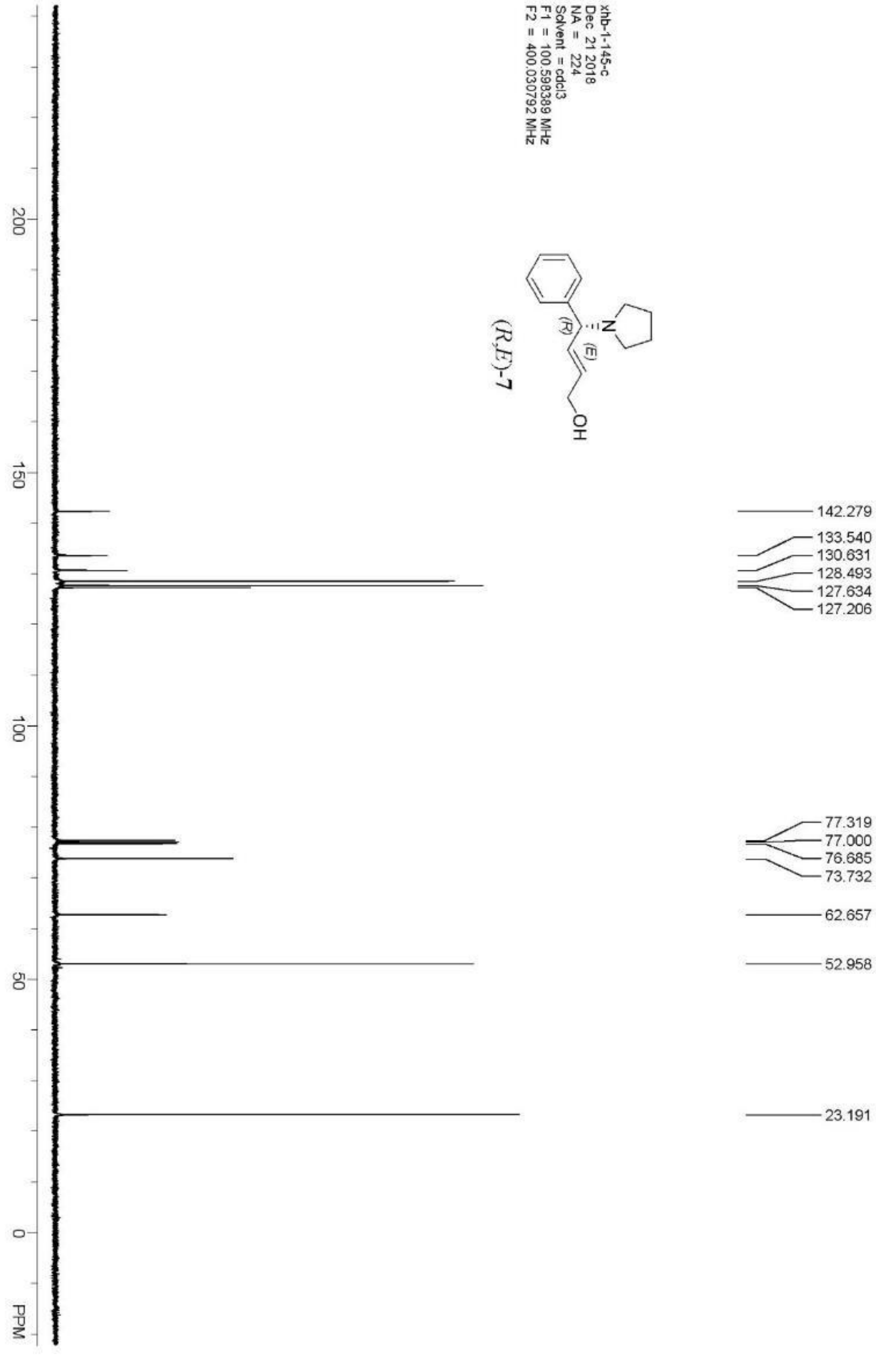

^{13}C NMR (400 MHz, $CDCl_3$) spectrum for (*R*,*E*)-**7**

Empower 3 SOFTWARE

Injection Summary Report

SAMPLE INFORMATION

Sample Name:	xhb-1-145-od-h-98-2-1.0-214	Acquired By:	System
Sample Type:	Unknown	Sample Set Name:	
Vial:	1	Acq. Method Set:	HPLC
Injection #:	3	Processing Method:	20181124
Injection Volume:	5.00 ul	Channel Name:	W2489 ChA
Run Time:	60.0 Minutes	Proc. Chnl. Descr.:	W2489 ChA 214nm
Date Acquired:	12/22/2018 4:33:22 AM CST		
Date Processed:	12/22/2018 5:14:13 AM CST		

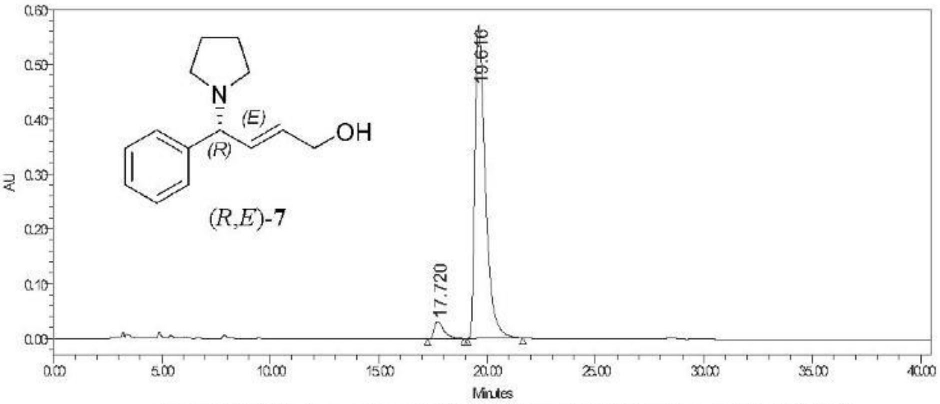

Channel: W2489 ChA; Processed Channel: W2489 ChA 214nm; Result Id: 1892; Processing Method: 2018112[illegible]

Processed Channel Descr.: W2489 ChA 214nm

	Processed Channel Descr.	RT	Area	%Area	Height
1	W2489 ChA 214nm	17.720	956357	4.78	31524
2	W2489 ChA 214nm	19.616	19040945	95.22	570900

Reported by User: System
Report Method: Injection Summary Report
Report Method ID: 1639 1639
Page: 1 of 1

Project Name: HPLC
Date Printed:
12/22/2018
9:05:45 AM PRC

HPLC spectrum for (*R*,*E*)-7

Empower 3 SOFTWARE

Injection Summary Report

SAMPLE INFORMATION

Sample Name:	xhb-1-137-od-h-98-2-1.0-214	Acquired By:	System
Sample Type:	Unknown	Sample Set Name:	
Vial:	1	Acq. Method Set:	HPLC
Injection #:	2	Processing Method:	20181124
Injection Volume:	3.00 ul	Channel Name:	W2489 ChA
Run Time:	60.0 Minutes	Proc. Chnl. Descr.:	W2489 ChA 214nm
Date Acquired:	12/22/2018 3:42:58 AM CST		
Date Processed:	12/22/2018 4:50:05 AM CST		

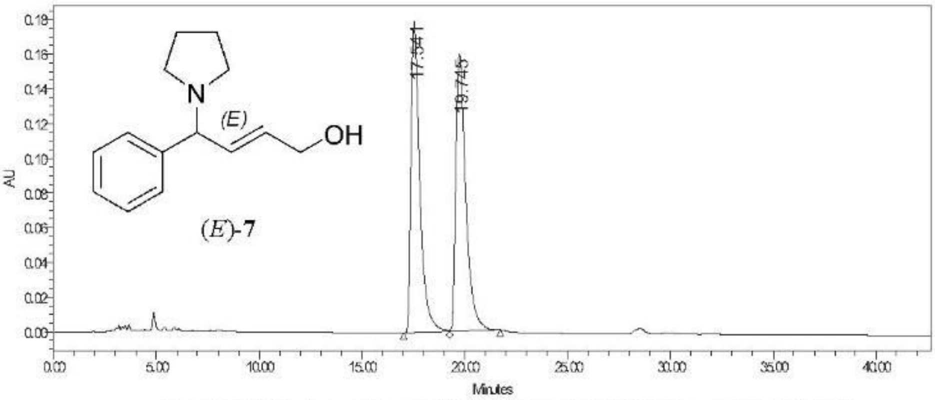

Processed Channel Descr.: W2489 ChA 214nm

	Processed Channel Descr.	RT	Area	%Area	Height
1	W2489 ChA 214nm	17.541	5333873	50.02	179538
2	W2489 ChA 214nm	19.745	5328564	49.98	159425

Reported by User: System
Report Method: Injection Summary Report
Report Method ID: 1639 1639
Page: 1 of 1

Project Name: HPLC
Date Printed:
12/22/2018
4:50:32 AM PRC

HPLC spectrum for (*E*)-7

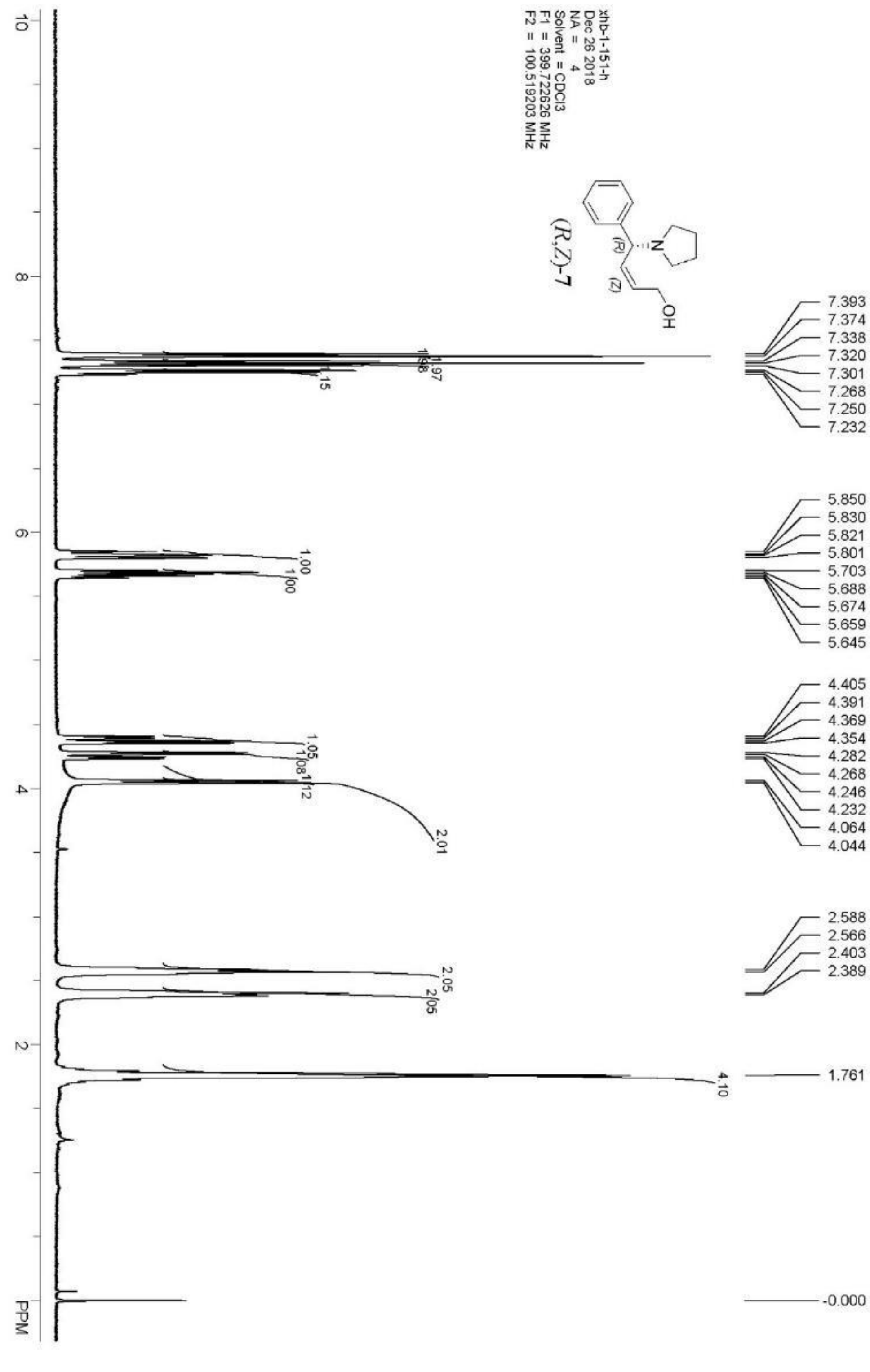

^{1}H NMR (400 MHz, CDCl3) spectrum for (*R*,*Z*)-**7**

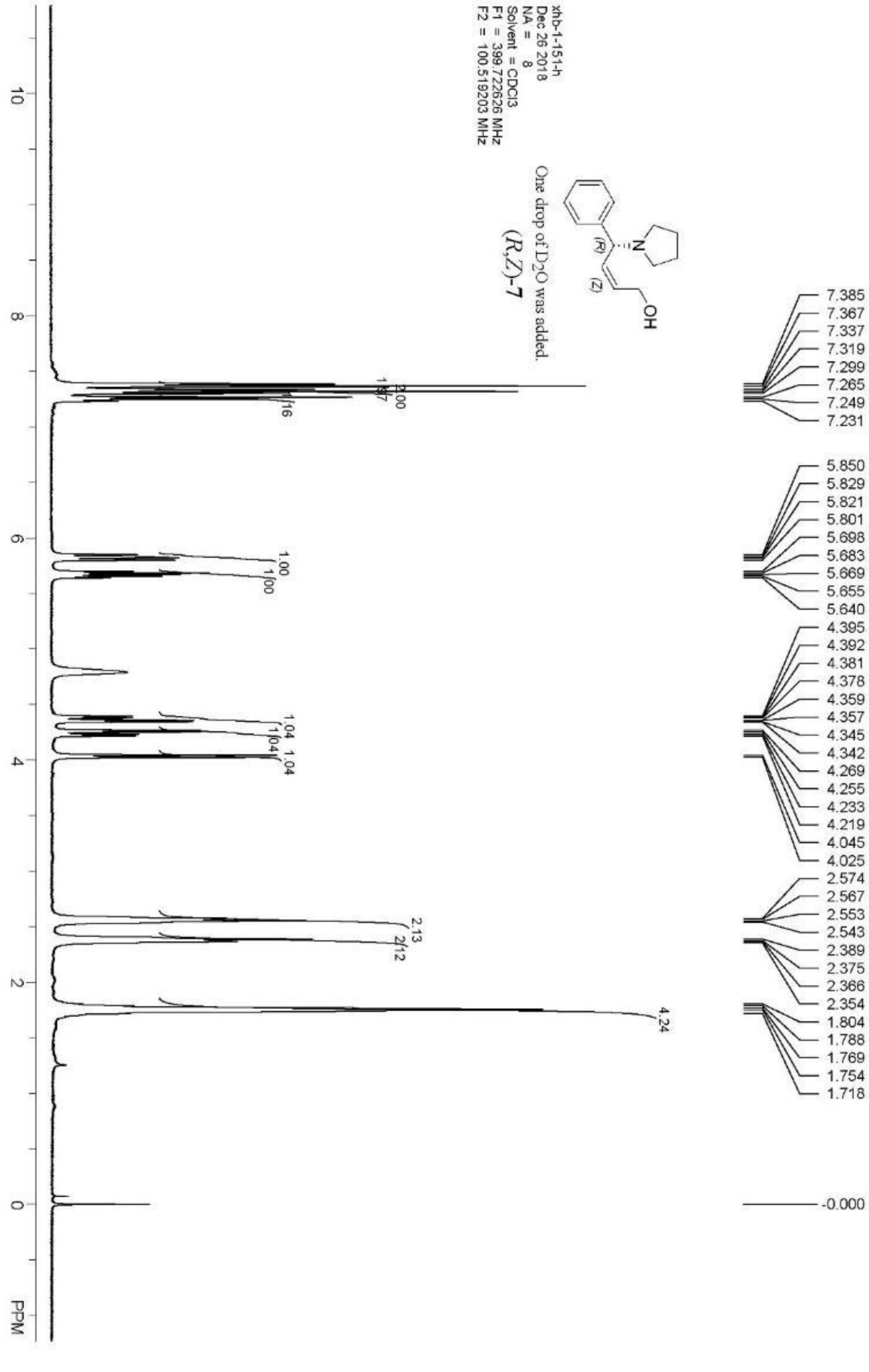

^{1}H NMR (400 MHz, CDCl3) spectrum for (*R*,*Z*)-**7**

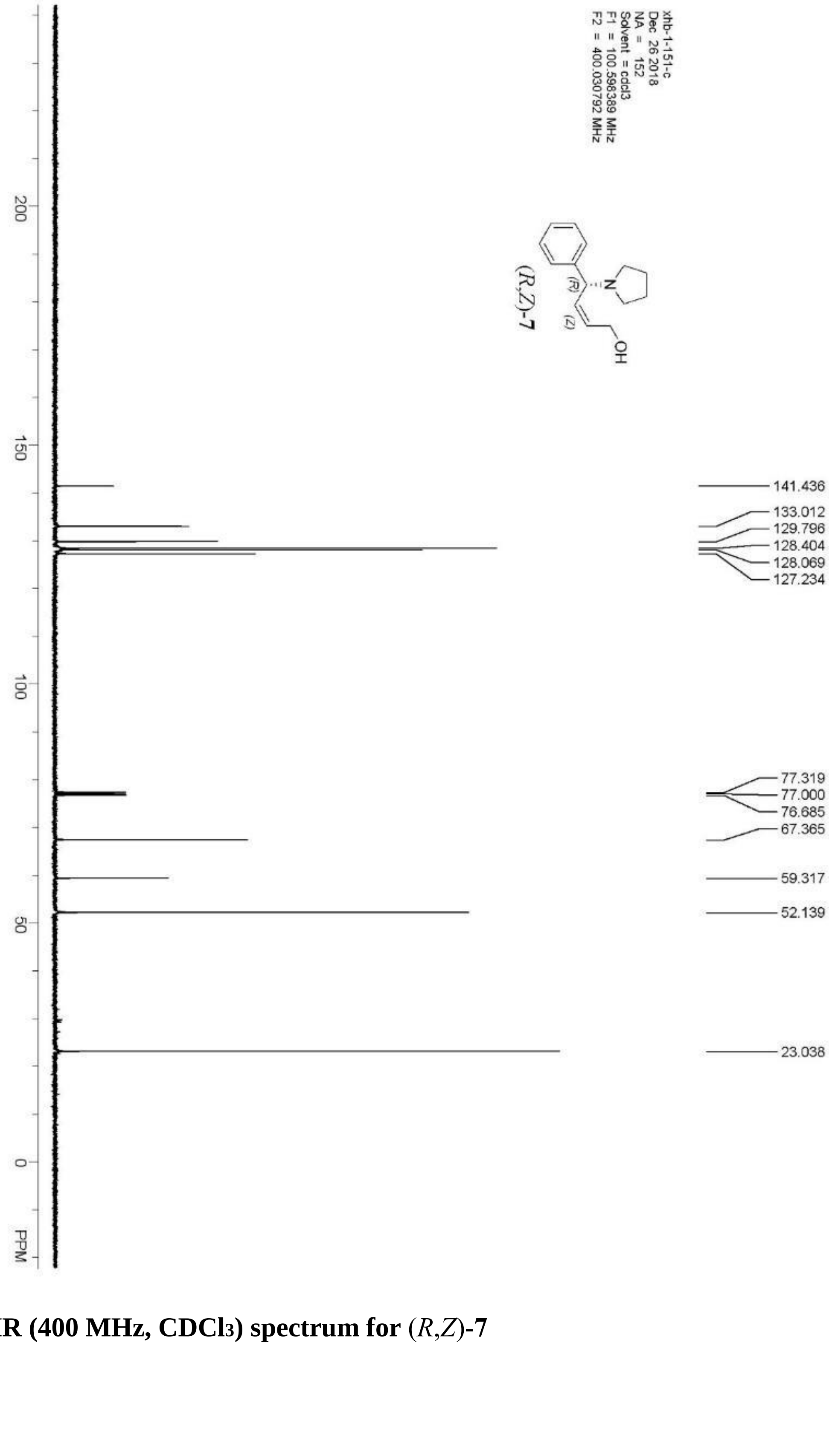

^{13}C NMR (400 MHz, CDCl$_3$) spectrum for (*R*,*Z*)**-7**

Empower™ 3 SOFTWARE

Injection Summary Report

SAMPLE INFORMATION

Sample Name:	xhb-1-151-oj-h-95-5-0.5-214	Acquired By:	System
Sample Type:	Unknown	Sample Set Name:	
Vial:	1	Acq. Method Set:	HPLC
Injection #:	3	Processing Method:	Default
Injection Volume:	5.00 ul	Channel Name:	W2489 ChA
Run Time:	60.0 Minutes	Proc. Chnl. Descr.:	W2489 ChA 214nm
Date Acquired:	12/27/2018 4:20:27 PM CST		
Date Processed:	12/27/2018 4:58:35 PM CST		

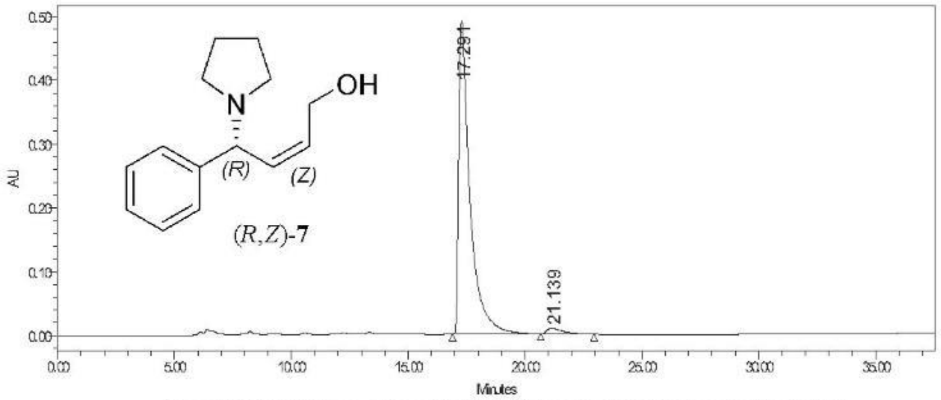

Channel: W2489 ChA; Processed Channel: W2489 ChA 214nm; Result Id: 2071; Processing Method: Default

Processed Channel Descr.: W2489 ChA 214nm

	Processed Channel Descr.	RT	Area	%Area	Height
1	W2489 ChA 214nm	17.291	16381249	97.39	491064
2	W2489 ChA 214nm	21.139	439507	2.61	9166

Reported by User: System
Report Method: Injection Summary Report
Report Method ID: 1639 1639
Page: 1 of 1

Project Name: HPLC
Date Printed:
12/28/2018
2:09:56 AM PRC

HPLC spectrum for (*R*,*Z*)-7

Empower 3 SOFTWARE

Injection Summary Report

SAMPLE INFORMATION

Sample Name:	xhb-1-146-oj-h-95-5-0.5-214	Acquired By:	System
Sample Type:	Unknown	Sample Set Name:	
Vial:	1	Acq. Method Set:	HPLC
Injection #:	2	Processing Method:	Default
Injection Volume:	5.00 ul	Channel Name:	W2489 ChA
Run Time:	60.0 Minutes	Proc. Chnl. Descr.:	W2489 ChA 214nm
Date Acquired:	12/27/2018 3:29:43 PM CST		
Date Processed:	12/27/2018 4:29:06 PM CST		

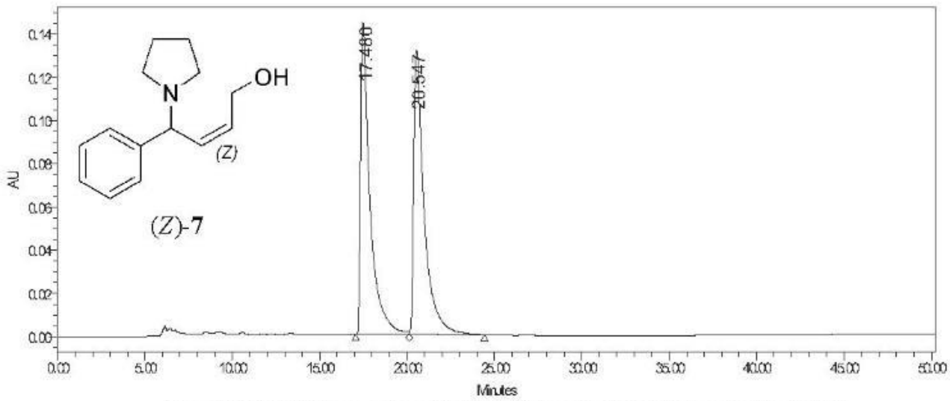

Channel: W2489 ChA; Processed Channel: W2489 ChA 214nm; Result Id: 2070; Processing Method: Defaul

Processed Channel Descr.: W2489 ChA 214nm

	Processed Channel Descr.	RT	Area	%Area	Height
1	W2489 ChA 214nm	17.480	5396565	49.93	144229
2	W2489 ChA 214nm	20.547	5412074	50.07	131603

Reported by User: System
Report Method: Injection Summary Report
Report Method ID: 1639 1639
Page: 1 of 1

Project Name: HPLC
Date Printed:
12/28/2018
2:09:25 AM PRC

HPLC spectrum for (*Z*)-7

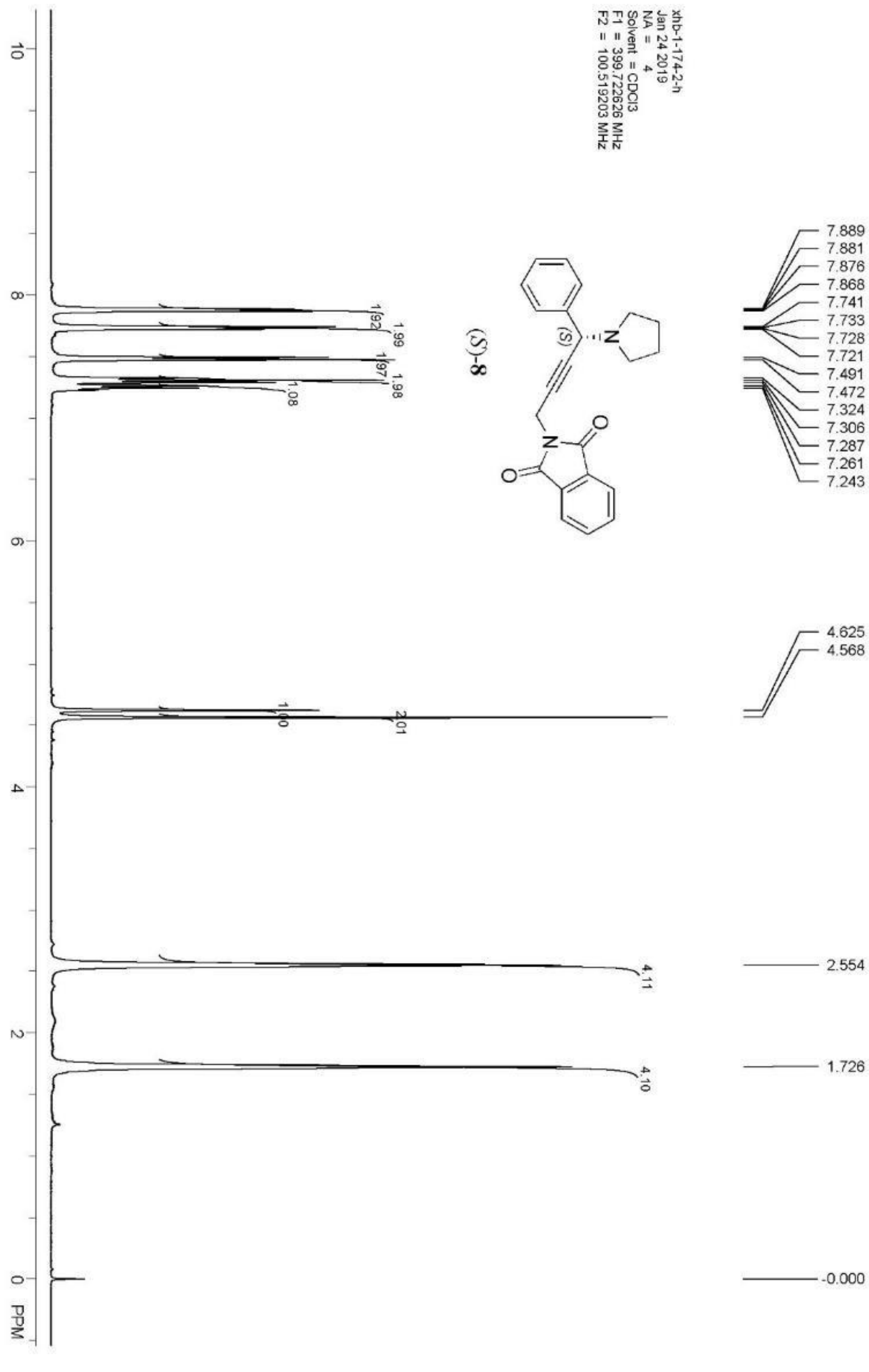

^{1}H NMR (400 MHz, CDCl3) spectrum for (*S*)-**8**

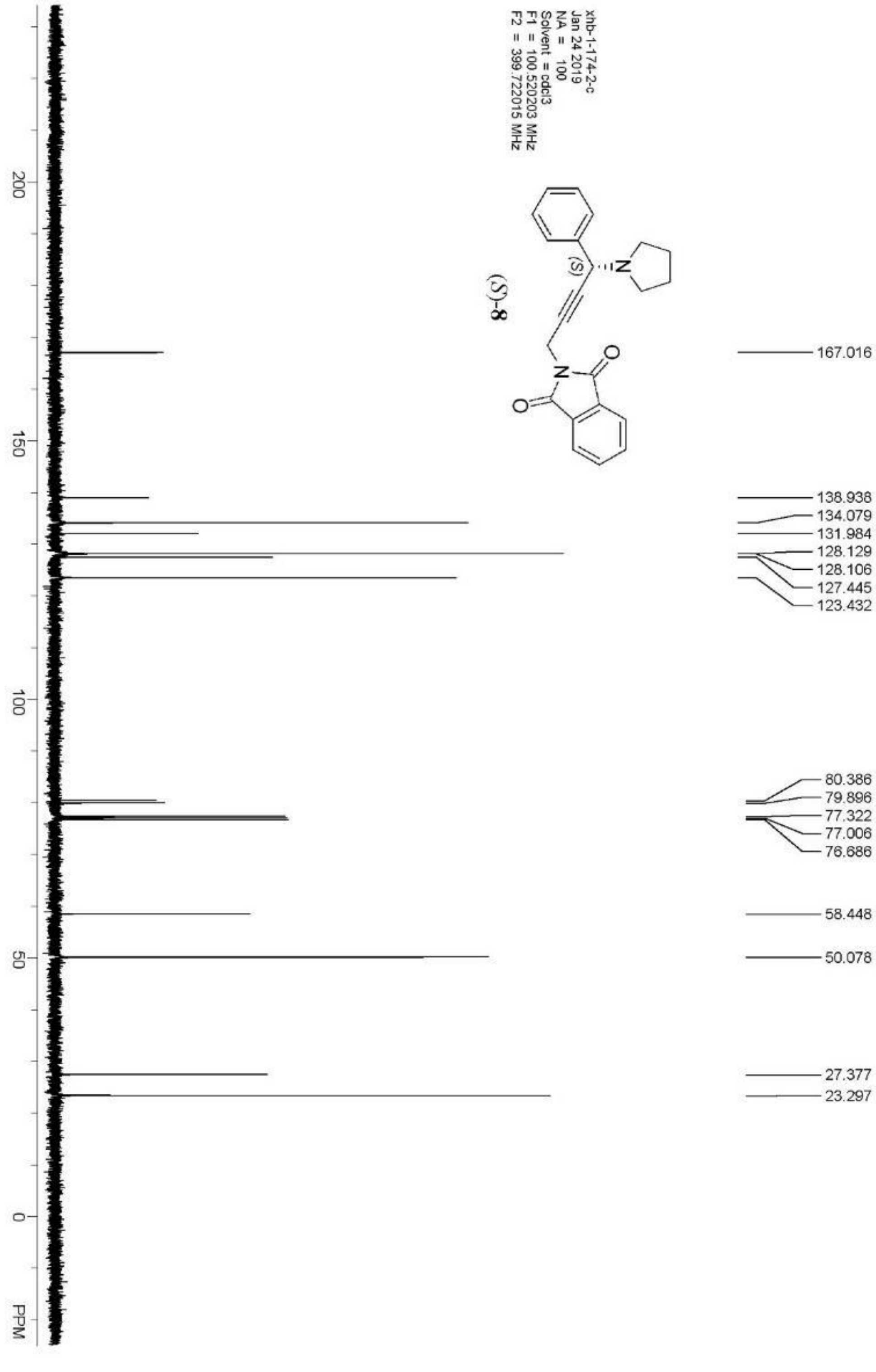

^{13}C NMR (400 MHz, CDCl$_3$) spectrum for (*S*)-8

Empower 3 SOFTWARE

Injection Summary Report

SAMPLE INFORMATION

Sample Name:	xhb-1-174-2-od-h-98-2-0.7-214	Acquired By:	System
Sample Type:	Unknown	Sample Set Name:	
Vial:	1	Acq. Method Set:	HPLC
Injection #:	2	Processing Method:	Default
Injection Volume:	5.00 ul	Channel Name:	W2489 ChA
Run Time:	60.0 Minutes	Proc. Chnl. Descr.:	W2489 ChA 214nm
Date Acquired:	1/25/2019 3:57:48 AM CST		
Date Processed:	1/25/2019 5:16:27 AM CST		

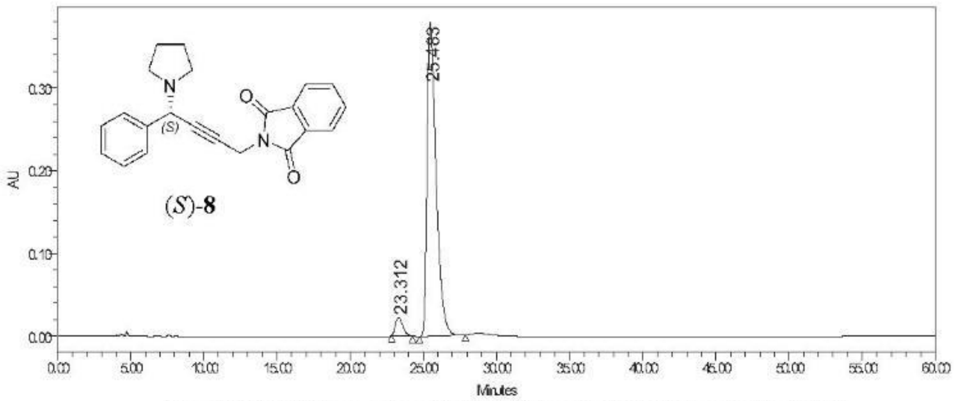

Processed Channel Descr.: W2489 ChA 214nm

	Processed Channel Descr.	RT	Area	%Area	Height
1	W2489 ChA 214nm	23.312	756054	4.48	21775
2	W2489 ChA 214nm	25.483	16123780	95.52	378760

Reported by User: System
Report Method: Injection Summary Report
Report Method ID: 1639 1639
Page: 1 of 1

Project Name: HPLC
Date Printed:
1/25/2019
5:16:53 AM PRC

HPLC spectrum for (*S*)-8

Empower 3 SOFTWARE

Injection Summary Report

SAMPLE INFORMATION

Sample Name:	xhb-1-172-od-h-98-2-0.7-214	Acquired By:	System
Sample Type:	Unknown	Sample Set Name:	
Vial:	1	Acq. Method Set:	HPLC
Injection #:	1	Processing Method:	Default
Injection Volume:	5.00 ul	Channel Name:	W2489 ChA
Run Time:	60.0 Minutes	Proc. Chnl. Descr.:	W2489 ChA 214nm
Date Acquired:	1/25/2019 3:11:40 AM CST		
Date Processed:	1/25/2019 3:54:21 AM CST		

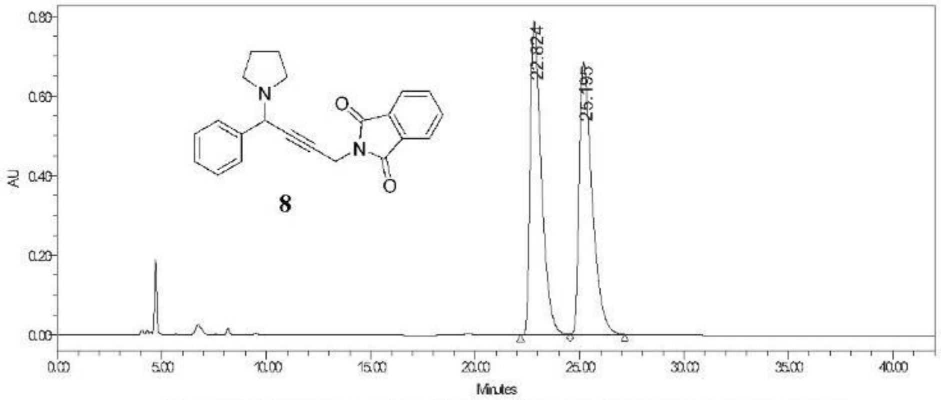

Channel: W2489 ChA; Processed Channel: W2489 ChA 214nm; Result Id: 2483; Processing Method: Defaul

Processed Channel Descr.: W2489 ChA 214nm

	Processed Channel Descr.	RT	Area	%Area	Height
1	W2489 ChA 214nm	22.824	29267549	49.97	788509
2	W2489 ChA 214nm	25.195	29297340	50.03	685202

Reported by User: System
Report Method: Injection Summary Report
Report Method ID: 1639 1639
Page: 1 of 1

Project Name: HPLC
Date Printed:
1/25/2019
3:54:42 AM PRC

HPLC spectrum for (±)-8

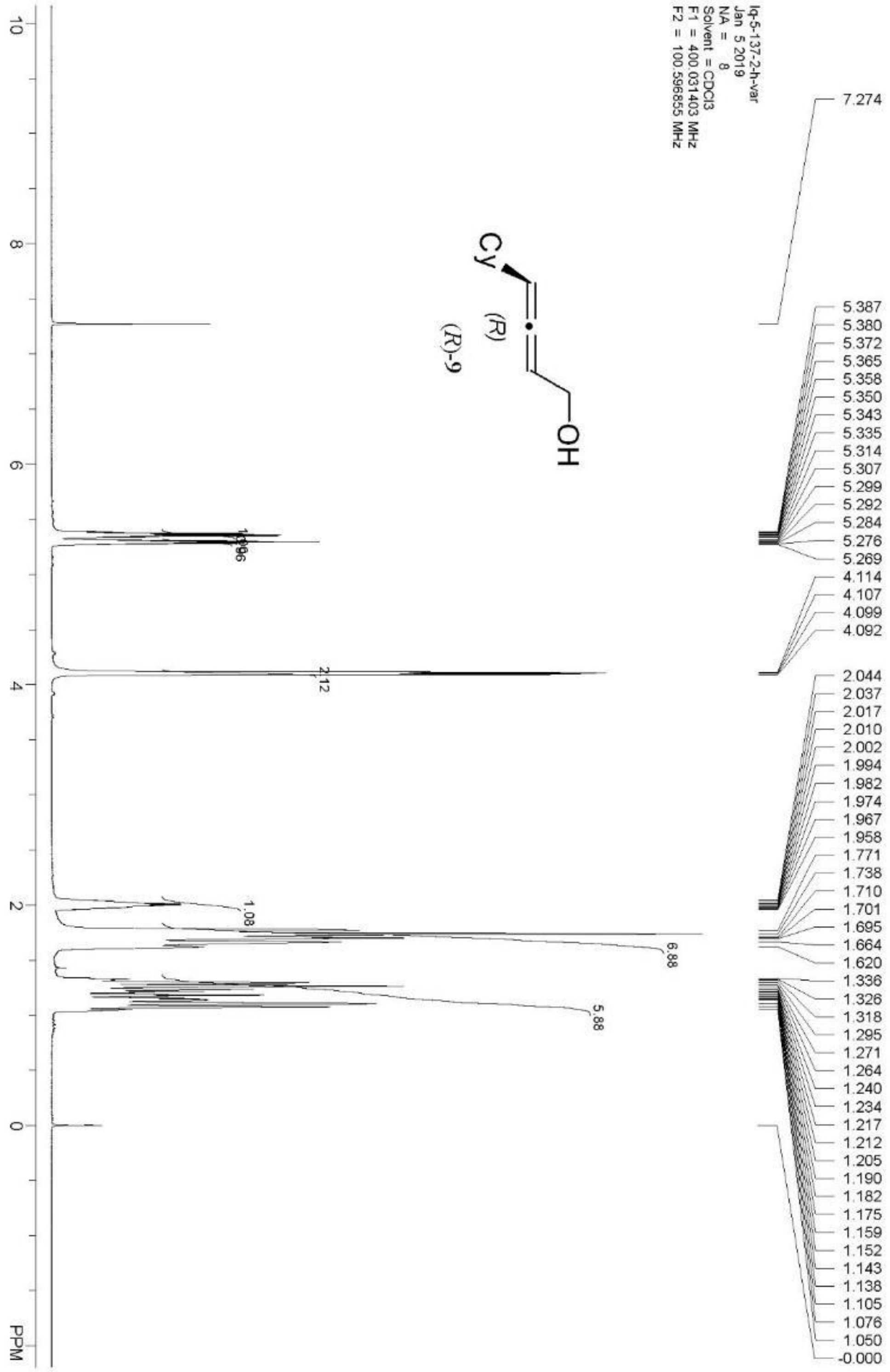

^{1}H NMR (400 MHz, CDCl3) spectrum for

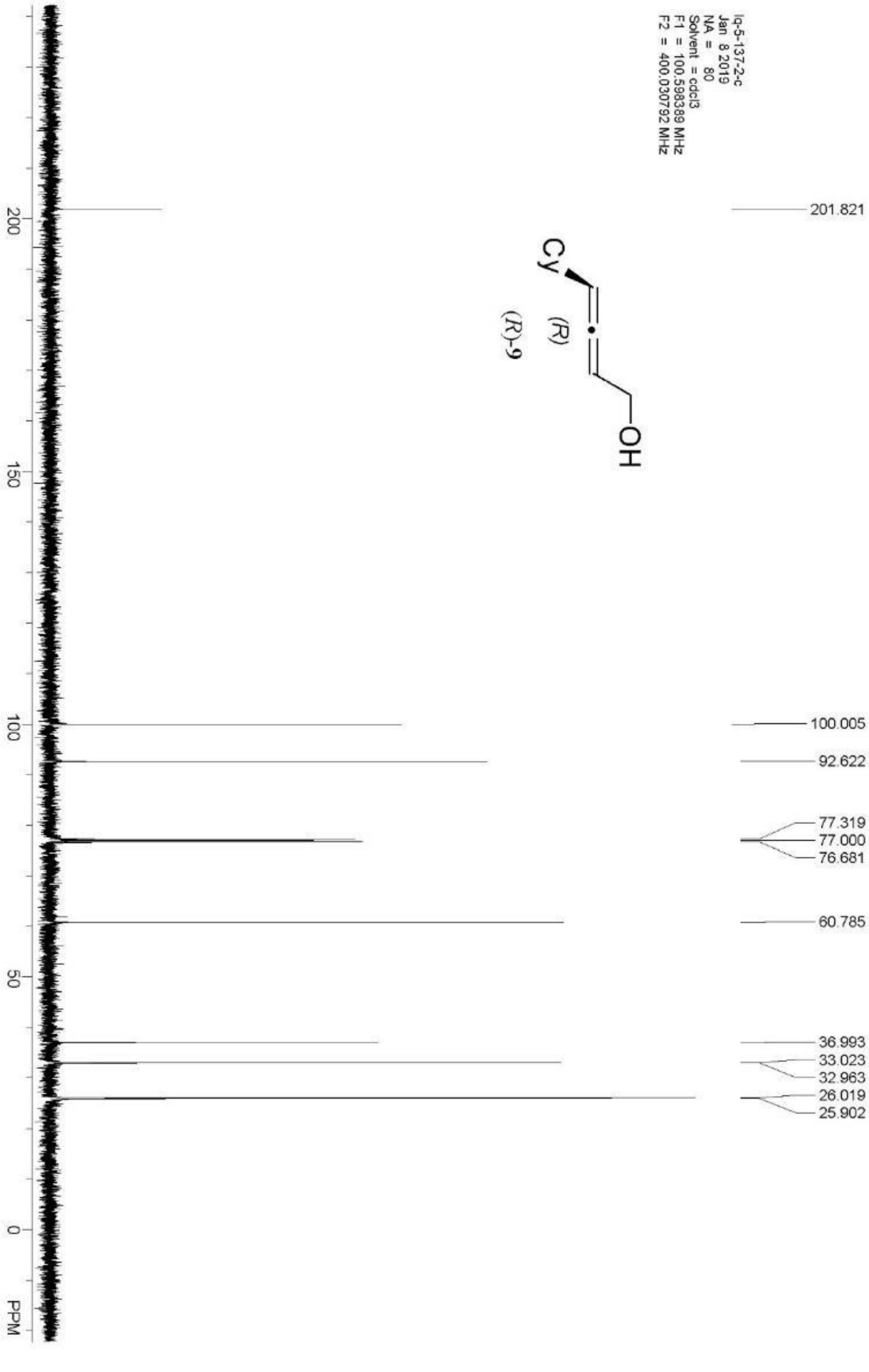

^{13}C NMR (400 MHz, CDCl$_3$) spectrum for (*R*)-9

Empower 3 SOFTWARE

Injection Summary Report

SAMPLE INFORMATION

Sample Name:	lq-5-137-2-as-h98-2-1.0-214	Acquired By:	System
Sample Type:	Unknown	Sample Set Name:	
Vial:	1	Acq. Method Set:	HPLC
Injection #:	4	Processing Method:	Default
Injection Volume:	10.00 ul	Channel Name:	W2489 ChA
Run Time:	40.0 Minutes	Proc. Chnl. Descr.:	W2489 ChA 214nm
Date Acquired:	1/6/2019 8:28:20 AM CST		
Date Processed:	1/6/2019 8:52:37 AM CST		

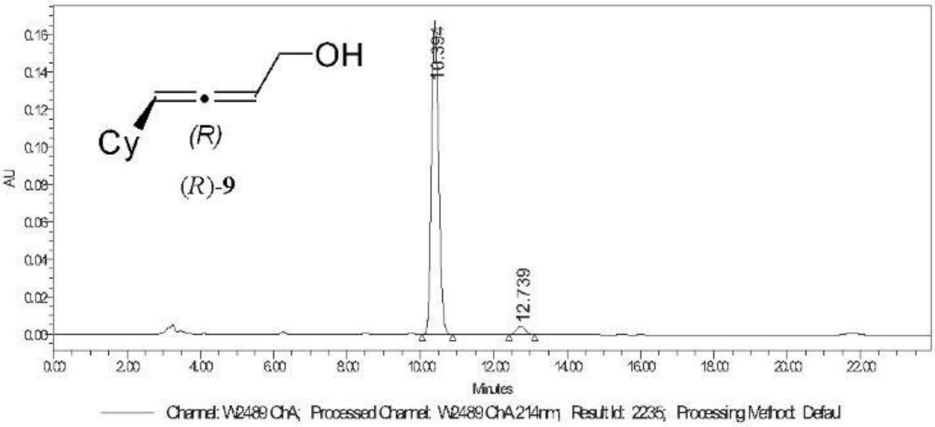

Processed Channel Descr.: W2489 ChA 214nm

	Processed Channel Descr.	RT	Area	%Area	Height
1	W2489 ChA 214nm	10.394	2273328	97.07	167524
2	W2489 ChA 214nm	12.739	68716	2.93	4299

Reported by User: System
Report Method: Injection Summary Report
Report Method ID: 1639 1639
Page: 1 of 1

Project Name: HPLC
Date Printed:
1/6/2019
8:53:21 AM PRC

HPLC spectrum for (*R*)-9

Empower™ 3 SOFTWARE

Injection Summary Report

SAMPLE INFORMATION

Sample Name:	lq-2-114-as-h-98-2-1.0-214	Acquired By:	System
Sample Type:	Unknown	Sample Set Name:	
Vial:	1	Acq. Method Set:	HPLC
Injection #:	5	Processing Method:	Default
Injection Volume:	10.00 ul	Channel Name:	W2489 ChA
Run Time:	40.0 Minutes	Proc. Chnl. Descr.:	W2489 ChA 214nm
Date Acquired:	1/6/2019 8:55:33 AM CST		
Date Processed:	1/6/2019 9:25:45 AM CST		

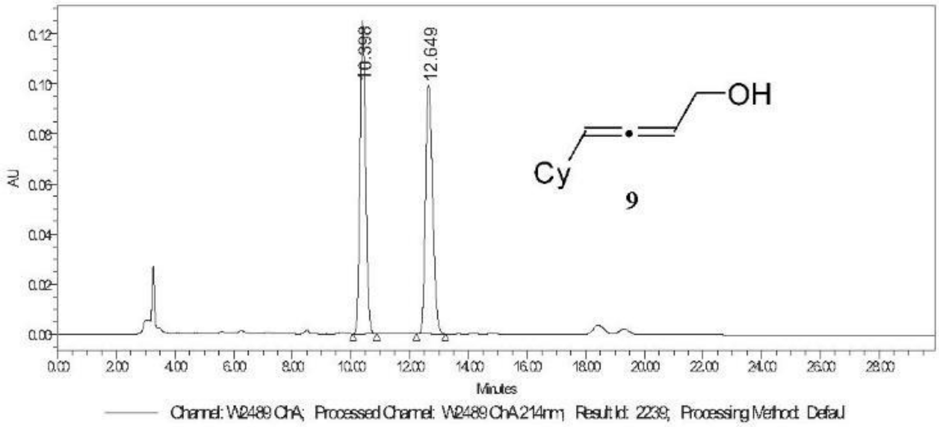

Processed Channel Descr.: W2489 ChA 214nm

	Processed Channel Descr.	RT	Area	%Area	Height
1	W2489 ChA 214nm	10.398	1676197	49.99	124958
2	W2489 ChA 214nm	12.649	1677054	50.01	99400

Reported by User: System
Report Method: Injection Summary Report
Report Method ID: 1639 1639
Page: 1 of 1

Project Name: HPLC
Date Printed:
1/6/2019
9:25:57 AM PRC

HPLC spectrum for (±)-9

Supplementary References

1. Danjo, H., Mitani, N., Muraki, Y., Kawahata, M., Azumaya, I., Yamaguchi, K. & Miyazawa, T. Tris(spiroborate)-type anionic nanocycles. *Chem.-Asian J.* **7**, 1529-1532 (2012).
2. Rokade, B. V. & Guiry, P. J. Enantioselective catalytic asymmetric A^3 coupling with phosphino-imidazoline ligands. *J. Org. Chem.* **84**, 5763-5772 (2019).
3. Fan, W. & Ma, S. An easily removable stereo-dictating group for enantioselective synthesis of propargylic amines. *Chem. Commun.* **49**, 10175-10175 (2013).
4. Zhao, C. & Seidel, D. Enantioselective A^3 reactions of secondary amines with a Cu(I)/acid-thiourea catalyst combination. *J. Am. Chem. Soc.* **137**, 4650-4653 (2015).
5. Aschwanden, P., Stephenson, C. R. J. & Carreira, E. M. Highly enantioselective access to primary propargylamines: 4-piperidinone as a convenient protecting group. *Org. Lett.* **8**, 2437-2440 (2006).
6. Gommermann, N. & Knochel, P. Practical highly enantioselective synthesis of terminal propargylamines. An expeditious synthesis of (*S*)-(+)-coniine. *Chem. Commun.*, 2324-2325 (2004).
7. Paioti, P. H. S., Abboud, K. A. & Aponick, A. Catalytic enantioselective synthesis of amino skipped diynes. *J. Am. Chem. Soc.* **138**, 2150-2153 (2016).
8. Xu, D., Li, Z. & Ma, S. Novozym-435-catalyzed enzymatic separation of racemic propargylic alcohols. A facile route to optically active terminal aryl propargylic alcohols. *Tetrahedron Lett.* **44**, 6343-6346 (2003).
9. Santandrea, J., Minozzi, C., Cruché, C. & Collins, S. K. Photochemical dual-catalytic synthesis of alkynyl sulfides. *Angew. Chem. Int. Ed.* **56**, 12255-12259 (2017).
10. Wu, T. R. & Chong, J. M. Asymmetric synthesis of propargylamides via 3,3'-disubstituted binaphthol-modified alkynylboronates. *Org. Lett.* **8**, 15-18 (2006).
11. Besong, G., Jarowicki, K., Kocienski, P. J., Sliwinski, E. & Boyle, F. T. Synthesis of (*S*)-(-)-*N*-acetylcolchinol using intramolecular biaryl oxidative coupling. *Org. Biomol. Chem.* **4**, 2193-2207 (2006).
12. Sawyer, J. S. & Macdonald, T. L. Total synthesis of (±)-*N*-acetylcolchinol. *Tetrahedron Lett.* **29**, 4839-4842 (1988).
13. Čech, J. & Šantavý, F. The effect of hydrogen peroxide in alkaline medium on colchiceine. *Collect. Czech. Chem. Commun.* **14**, 532-539 (1949).
14. Nicolaus, N., Reball, J., Sitnikov, N., Velder, J., Termath, A., Fedorov, A. Y. & Schmalz, H.-G. A convenient entry to new C-7-modified colchicinoids through azide alkyne [3+2] cycloaddition: Application of ring-contractive rearrangements. *Heterocycles* **82**, 1585-1585 (2010).
15. Fernholz, H. Űber die umlagerung des colchicins mit natriumalkoholat und die struktur des ringes C. *Justus Liebigs Ann. Chem.* **568**, 63-72 (1950).
16. Besong, G., Billen, D., Dager, I., Kocienski, P., Sliwinski, E., Tai, L. R. & Boyle, F. T. A synthesis of (a*R*,7*S*)-(-)-*N*-acetylcolchinol and its conjugate with a cyclic RGD peptide. *Tetrahedron* **64**, 4700-4710 (2008).
17. Courtois, G. & Miginiac, P. Action d'organometalliques fonctionnels sur les gem-aminoethers et les sels d'immonium. Ii. Synthese d'amines γ-fonctionnelles α-acetyleniques, α-ethyleniques z ou e et saturees. *Bull. Soc. Chim. Fr.* **2**, 21-27 (1983).

18. Brown, C. A. & Ahuja, V. K. "P-2 nickel" catalyst with ethylenediamine, a novel system for highly stereospecific reduction of alkynes to *cis*-olefins. *J. Chem. Soc., Chem. Commun.*, 553-554 (1973).
19. Mitsunobu, O. & Yamada, M. Preparation of esters of carboxylic and phosphoric acidviaquaternary phosphonium salts. *Bull. Chem. Soc. Jpn.* **40**, 2380-2382 (1967).
20. Mitsunobu, O. The use of diethyl azodicarboxylate and triphenylphosphine in synthesis and transformation of natural products. *Synthesis*, 1-28 (1981).
21. Ye, J., Fan, W. & Ma, S. *tert*-Butyldimethylsilyl-directed highly enantioselective approach to axially chiral α-allenols. *Chem.-Eur. J.* **19**, 716-720 (2013).
22. Ye, J. & Ma, S. Preparation of (*R*)-4-cyclohexyl-2,3-butadien-1-ol. *Org. Synth.* **91**, 233-247 (2014).
23. Paioti, P. H. S., Abboud, K. A. & Aponick, A. Incorporation of axial chirality into phosphino-imidazoline ligands for enantioselective catalysis. *ACS Catal.* **7**, 2133-2138 (2017).
24. Zhang, J.-T., Wang, H.-Y., Zhu, W., Cai, T.-T. & Guo, Y.-L. Solvent-assisted electrospray ionization for direct analysis of various compounds (complex) from low/nonpolar solvents and eluents. *Anal. Chem.* **86**, 8937-8942 (2014).
25. Frisch, M. J.; Trucks, G. W.; Schlegel, H. B.; Scuseria, G. E.; Robb, M. A.; Cheeseman, J. R.; Scalmani, G.; Barone, V.; Mennucci, B.; Petersson, G. A.; Nakatsuji, H.; Caricato, M.; Li, X.; Hratchian, H. P.; Izmaylov, A. F.; Bloino, J.; Zheng, G.; Sonnenberg, J. L.; Hada, M.; Ehara, M.; Toyota, K.; Fukuda, R.; Hasegawa, J.; Ishida, M.; Nakajima, T.; Honda, Y.; Kitao, O.; Nakai, H.; Vreven, T.; Montgomery, J. A., Jr.; J. E, P.; Ogliaro, F.; Bearpark, M.; Heyd, J. J.; Brothers, E.; Kudin, K. N.; Staroverov, V. N.; Keith, T.; Kobayashi, R.; Normand, J.; Raghavachari, K.; Rendell, A.; Burant, J. C.; Iyengar, S. S.; Tomasi, J.; Cossi, M.; Rega, N.; Millam, J. M.; Klene, M.; Knox, J. E.; Cross, J. B.; Bakken, V.; Adamo, C.; Jaramillo, J.; Gomperts, R.; Stratmann, R. E.; Yazyev, O.; Austin, A. J.; Cammi, R.; Pomelli, C.; Ochterski, J. W.; Martin, R. L.; Morokuma, K.; Zakrzewski, V. G.; Voth, G. A.; Salvador, P.; Dannenberg, J. J.; Dapprich, S.; Daniels, A. D.; Farkas, O.; Foresman, J. B.; Ortiz, J. V.; Cioslowski, J.; Fox, D. J. Gaussian 09, Revision D.01, Gaussian, Inc., Wallingford, CT, **2009**.
26. (a) Zhao, Y.; Truhlar, D. G. Density functionals with broad applicability in chemistry. *Acc. Chem. Res.* **41**, 157-167 (2008). (b) Zhao, Y. Truhlar, D. G. The M06 suite of density functionals for main group thermochemistry, thermochemical kinetics, noncovalent interactions, excited states, and transition elements: two new functionals and systematic testing of four M06-class functionals and 12 other functionals. *Theor. Chem. Acc.* 120, 215-241 (2008).
27. (a) Fukui, K. The path of chemical reactions - the IRC approach. *Acc. Chem. Res.* **14**, 363-368 (1981); (b) Dunning, Jr. T. H.; Hay, P. J. "Gaussian basis sets for molecular calculations" in *Methods of electronic structure theory* (vol. 3 of *Modern Theoretical Chemistry*), H. F. Schaefer, Ed. (Plenum Press, New York, 1977), pp. 1-27.
28. Schwerdtfeger, P. The pseudopotential approximation in electronic structure theory. *ChemPhysChem* **12**, 3143-3155 (2011).
29. (a) Hay, P. J.; Wadt, W. R. *Ab initio effective* core potentials for molecular calculations. Potentials for the transition metal atoms Sc to Hg. *J. Chem. Phys.* **82**, 270-283 (1985); (b) Wadt, W. R.; Hay, P. J. *Ab initio effective* core potentials for molecular calculations. Potentials for main group elements Na to Bi. *J. Chem. Phys.* **82**, 284-298 (1985); (c) Hay, P. J.; Wadt, W. R. *Ab initio effective* core potentials for molecular calculations. Potentials for K to Au including the outermost

core orbitals. *J. Chem. Phys.* **82**, 299-310 (1985).
30. Andrae, D.; Häußermann, U.; Dolg, M.; Stoll H.; Preuß, H. Energy-adjusted *ab initio* pseudopotentials for the second and third row transition elements. *Theor. Chim. Acta* **77**, 123-141 (1990).
31. Marenich, A. V.; Cramer, C. J.; Truhlar, D. G. Universal solvation model based on solute electron density and on a continuum model of the solvent defined by the bulk dielectric constant and atomic surface tensions. *J. Phys. Chem.* B, **113**, 6378-6396 (2009).
